# Decoding Non-Coding RNA Regulators in DITRA: From Genomic Insights to Potential Biomarkers and Therapeutic Targets

**DOI:** 10.3390/genes16070753

**Published:** 2025-06-27

**Authors:** Sofia Spanou, Athena Andreou, Katerina Gioti, Dimitrios Chaniotis, Apostolos Beloukas, Louis Papageorgiou, Trias Thireou

**Affiliations:** 1 Laboratory of Genetics, Department of Biotechnology, Agricultural University of Athens, 11855 Athens, Greece; sofiaecb@gmail.com (S.S.); aandre@aua.gr (A.A.); 2 Department of Biomedical Sciences, School of Health Sciences, University of West Attica, 12243 Egaleo, Greecedchaniotis@uniwa.gr (D.C.); abeloukas@uniwa.gr (A.B.); 3 National AIDS Reference Centre of Southern Greece, School of Public Health, University of West Attica, 11521 Athens, Greece

**Keywords:** Interleukin family, functional genomics, genetics, epigenetics, therapeutic targets, biomarkers

## Abstract

Background: Deficiency of *IL-36* Receptor Antagonist (DITRA) is a rare monogenic autoinflammatory disease, characterized by dysregulation of *IL-36* signaling and phenotypically classified as a subtype of generalized pustular psoriasis. Objectives: This study aimed to explore the role of potentially coding and non-coding RNAs (ncRNAs) in the *IL36RN* interactome to identify putative pathogenic mechanisms, biomarkers, and therapeutic targets for DITRA. Methods: A systems biology approach was applied using the STRING database to construct the IL36RN protein–protein interaction network. Key ncRNA interactions were identified using RNAInter. The networks were visualized and analyzed with Cytoscape v3 and the CytoHubba plugin to identify central nodes and interaction hubs. Pathway enrichment analysis was then performed to determine the biological relevance of candidate ncRNAs and genes. Results: Analysis identified thirty-eight ncRNAs interacting with the IL36RN network, including six lncRNAs and thirty-two miRNAs. Of these, thirty-three were associated with key DITRA-related signaling pathways, while five remain to be validated. Additionally, seven protein-coding genes were highlighted, with three (*TINCR*, *PLEKHA1*, and *HNF4A*) directly implicated in biological pathways related to DITRA. Many of the identified ncRNAs have prior associations with immune-mediated diseases, including psoriasis, supporting their potential relevance in DITRA pathogenesis. Conclusions: This study provides novel insights into the ncRNA-mediated regulation of *IL36RN* and its network in the context of DITRA. The findings support the potential utility of specific ncRNAs and genes, such as *TINCR*, *PLEKHA1*, and *HNF4A*, as key genomic elements warrant further functional characterization to confirm their mechanistic roles and may inform biomarker discovery and targeted therapeutic development in DITRA.

## 1. Introduction

Psoriasis is a chronic, proliferative disease that causes the overactivation of the immune system and thus the rapid multiplication of skin cells. Both men and women are affected at any age, but most cases of psoriasis occur between the ages of 15 and 20 years of age, with a second peak occurring between the ages of 55 to 60 years. The cause of the disease is a mix of environmental and genetic factors. Many patients have a family history of psoriasis and certain genes have been potentially identified as contributors to its development. Most of these genes play a critical role in the function of the immune system. Scaly and inflamed patches of skin on the scalp, elbows or knees are often the most common symptoms that mostly go through cycles, lasting for weeks or months, followed by periods of remission. Generally, winter aggravates the symptoms whereas summer improves them. Treatment is formed based on the type of psoriasis and the severity of the condition but there is no cure yet. Psoriatic patients are often at risk of other sever conditions, such as psoriatic arthritis and infections, while stress, alcohol, smoking, obesity and hypocalcemia appear to be triggering factors [1,2,3].

### 1.1. Psoriasis Subtypes

Psoriasis has several subtypes that are categorized based on the different symptoms, the patient’s age of the disease appearance, the body part that is affected and the cause of its development. The most common subtype that affects 85–90% of patients is plaque psoriasis (psoriasis vulgaris) and presents as erythematous plaques—dry skin lesions—with silvery scales, mostly over the elbows, knees, scalp and back [2]. Guttate psoriasis (eruptive psoriasis) is the second most common subtype but the one with the best prognosis. It commonly affects children or young adults after upper respiratory tract infection with the streptococcal organism. Symptoms presented are erythematous and scaly raindrop-shaped lesions typically on the torso or limbs [1,2,3]. Most probably, this subtype disappears after a few weeks, but some patients develop plaque psoriasis [2]. Inverse psoriasis (flexural/intertriginous psoriasis) is often confused with dermatophyte infection as both affect the same sites: groins, armpits, intergluteal region and inframammary region. Psoriasis symptoms appear as smooth, erythematous, and sharply demarcated patches. About 21–30% of psoriatic patients develop inverse psoriasis, mostly obese patients [4].

The less common types of psoriasis are erythrodermic and pustular. Erythrodermic is an extreme form of psoriasis, potentially life-threatening. It affects more than 90% of the body surface area and it occurs in 1–2% of psoriatic patients [4]. Skin shows intense redness and discoloration, having a similar appearance to that of a burn. In a large area of the body, the skin is detached, while the scales are not small, but resemble leaves. This type of psoriasis disturbs the normal balance of body temperature and fluids and causes an increase in heart rate, large fluctuations in body temperature, severe itching and pain. It can be caused by severe sunburn, consumption of certain medications such as corticosteroids, stress, infection or alcoholism [5].PP is a clinically heterogeneous group of inflammatory skin diseases that includes different subtypes and often occurs in patients with psoriasis vulgaris [6,7]. The genetic background of PP is thought to be predominantly monogenic and recent genetic discoveries have led to the development of targeted biological drugs for the treatment of this condition [6]. The subtypes of PP are divided into two categories, generalized pustular psoriasis (GPP) and localized pustular psoriasis (LPP). GPP’s subtypes are von Zumbusch, annular, exanthematic and PP of pregnancy (PPP), while LPP’s subtypes are acrodermatitis continua of Hallopeau (ACH) and palmoplantar psoriasis(PaP) [7].

### 1.2. Anosology of Psoriasis

Psoriasis is considered both an autoimmune and autoinflammatory disease. Psoriatic skin activates both branches of the immune system, the innate and adaptive, depending on the type of disease. The loss of immunity against autologous tissues is caused by the presence of autoreactive T and B cells, while both genetic and environmental factors are responsible for the onset of the disease. Depending on the type of disease, inflammation occurs in certain areas, while lack of self-reactive T cells and high levels of autoantibody titres are observed [8]. Each type of psoriasis represents a different balance between autoimmune and autoinflammatory immune processes, and they have been correlated with several other autoimmune and autoinflammatory disorders [9,10,11]. Chronic plaque psoriasis is characterized by adaptive immunity, whereas PP, both generalized and localized, is characterized by innate immunity and auto-inflammatory responses. Erythrodermic and inverse psoriasis are more closely related to autoimmune than to autoinflammatory diseases and the immune response is based on activation of the adaptive immune system. Guttate psoriasis is considered primarily an autoimmune disease, as it is often triggered by bacterial infections and involves an immune response mediated mainly by T cells of the adaptive immune system [9].

### 1.3. IL36RN Mutation as the Cause of DITRA

As previously mentioned, GPP often occurs in people who are already suffering from plaque psoriasis. Studies have highlighted IL-36 receptor antagonist (*IL36Ra*) deficiency as one of the main causes of GPP in patients who have not developed psoriasis previously. *IL36Ra* is an antagonist of three cytokines that belong in the interleukin-1 family: interleukin-36α, interleukin-36β and interleukin-36γ, which activate several proinflammatory signaling pathways, such as the nuclear factor-κB and mitogen-activated protein kinase; thus, *IL36Ra* plays an important role in controlling the inflammatory response in the skin [12,13]. The absence or dysfunction of this antagonist is a rare genetic disorder and causes DITRA [14]. Specifically, DITRA is an autoinflammatory disease with an autosomal recessive inheritance pattern that manifests with recurrent episodes of pustular fever, leukocytosis and increased inflammatory markers and is caused by mutations in the *IL36RN* gene, located in the chromosome 2q14.1 [15]. DITRA has been associated with GPP, ACH and PPP.

### 1.4. Molecular Pathway of DITRA

**Figure 1:**
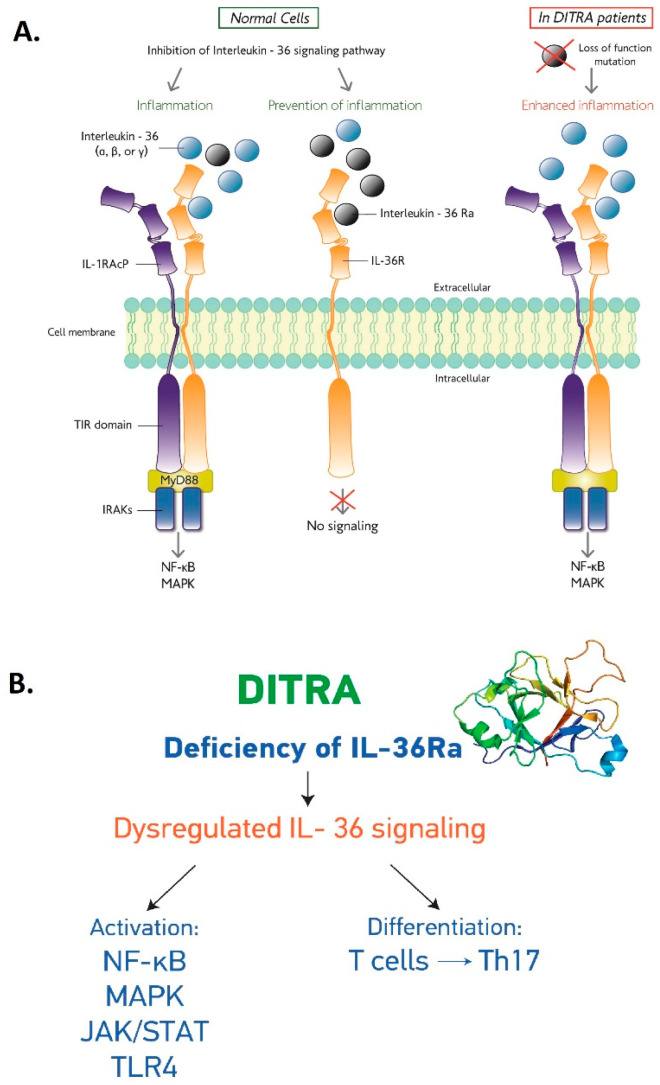
(**A**) Molecular pathway of DITRA. (**B**) Biomolecules in IL-36Ra interactome.

*IL36Ra* is a cytokine, which is encoded by the IL36RN gene, and its deficiency is one of the main causes of DITRA disease. It is a natural antagonist of the three IL-36 agonists, *IL-36α*, *IL-36β* and *IL-36γ*, and binds to IL1RL2 receptor (*IL-36R*, *IL-1Rrp2*, *IL1Rrp2*), inhibiting signaling and immune response [16]. *IL-36Ra* is involved in the interleukin 36 pathway, which regulates the activation of immune cells (macrophages, T cells, dendritic cells and neutrophils) (Figure 1) [17]. In normal cells, IL36Ra binds to the interleukin-36 receptor and inhibits the signaling of proinflammatory cytokines *IL-36α*, *IL-36β* and *IL-36γ.* Immune response is formed by preventing the activation of pathways that lead to inflammation. In contrast, when there is a mutation in *IL-36Ra*, the three IL-36 agonists are not blocked by the antagonist and bind to the interleukin-36 receptor, leading to the recruitment of the interleukin-1 receptor helper protein (Figure 1). This recruitment activates the nuclear factor-κB (NF-κB) and mitogen-activated protein kinases (MAPKs), initiating cell signaling (Figure 1). The IL-36Ra deficiency results in the impairment of this regulatory mechanism and the uncontrolled activation of the interleukin-36 signaling pathway, contributing to the inflammatory manifestations observed in DITRA, as increased neutrophil production leads to the development of pustules, the main symptom of PP [9,12,14,18].

### 1.5. ncRNAs in DITRA

MicroRNAs are small, non-coding RNA molecules that play a crucial role in the post-transcriptional regulation of gene expression. They function by binding to the 3′ UTRs of target mRNAs, leading to either degradation of the mRNA or inhibition of its translation. This mechanism allows miRNAs to regulate various cellular processes, such as keratinocyte proliferation, differentiation, and influence the immune response, which are particularly relevant in the pathogenesis of conditions like psoriasis [19]. Long-non-coding RNAs are a type of RNA molecules that are longer than 200 nucleotides and do not encode proteins. They are important regulatory molecules that influence gene expression and cellular processes. Specifically, they are found to act as scaffolds for protein complexes, influencing the transcription of involved target genes. They also function as endogenous sponges for miRNAs, thereby regulating the availability of these miRNAs to target mRNAs. In the psoriatic skin, certain lncRNAs are found up-regulated, resulting in keratinocyte hyperproliferation. Since microRNAs and lncRNAs regulate various cellular processes, their differential expression patterns may indicate their potential role as biomarkers and therapeutic targets [20].Thus, the aim of the present study was the investigation of macromolecular interaction networks contributing to DITRA pathogenesis, focusing on the impact of ncRNAs on *IL36RN* interactome towards identifying potential biomarkers, risk factors and therapeutic targets.

## 2. Materials and Methods

### 2.1. Data Collection and Selection of Non-Coding RNAs

An extensive literature review on PubMed identified *IL36RN* as the gene responsible for the pathogenesis of DITRA, when mutated from 1999 to January 2025. Mutations in this gene lead to a deficiency of *IL36Ra*, resulting in chronic inflammation and a hyper-responsive immune response [12]. *IL36RN* protein–protein interactions were collected from STRING DB using default settings and visualized with the open source software platform Cytoscape [21,22]. RNAInter v4.0, which includes information from six different databases, was also used to extract RNA–RNA interaction [23,24]. The resulting network was analyzed using cytoHubba to identify hub nodes and explore relevant pathways and important macromolecules [24,25]. cytoHubba is a Cytoscape plugin that ranks network nodes by calculating 12 topological analysis indices, using both local-based and global-based methods and thus capturing different network features [25,26]. The intersection of the top 100 nodes ranked by each method was calculated and the non-coding RNAs with a frequency of 7/12 or greater were selected for further investigation.

### 2.2. Non-Coding RNA Evaluation

Enrichment analysis was performed for the two groups of selected lncRNAs and miRNAs, using the online server RNAenrich [27]. RNAenrich can analyze various RNA types and includes a plethora of experimentally validated RNA–target interactions. The GO (Gene Ontology) Biological Pathways and the Reactome Pathway Knowledgebase 2024 terms with the lowest pAdj that were previously associated with DITRA or psoriasis were determined [28,29,30,31]. Moreover, the targets of each ncRNA and the GO Biological Processes were identified by searching RNAenrich and associated GeneCards version 5.23 [27,28,30]. Last but not least, all the extracted non-coding targets were evaluated for theircorrelation with DITRA, psoriasis and dermatitis using publications from PubMed.

## 3. Results

**Figure 2:**
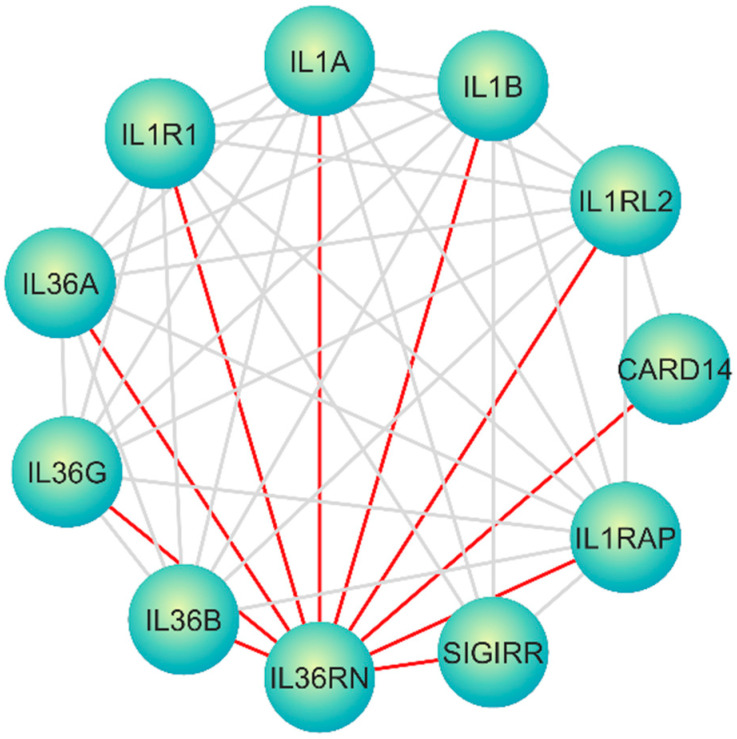
Interaction network from STRING DB visualized in Cytoscape, illustrating the complex interactions and functional connections between interleukin receptors and related molecules.

The ultimate goal of the present study was the identification of new candidate non-coding targets using the *IL36RN* interactome. Based on the literature search on PubMed, they have been identified in 14 studies that present pathogenic mutations in *IL36RN* associated with the manifestation of DITRA [7,32,33,34,35,36]. The *IL36RN* interactome has been extracted from the STRING database [22] and internal correlations with other macromolecules have been extracted from the RNAInter databases (Supplementary Table S1) [24]. Based on the results, genes as well as other genomic functional regions are identified in the main interaction network (Table 1 and Supplementary Table S1). Afterwards, the major interaction network with key role molecules has been analyzed and visualized using Cytoscape (Supplementary Data S1, Figure 2) [23]. Some of the genes that perform complex interactions and functional connections with *IL36RN* are *IL36A*, *IL36B*, *IL36G*, *IL1A*, *IL1B*, *IL1R1*, *IL1RL2*, *IL1RAP*, *CARD14*, and *SIGIRR* (Figure 2). Since the network contained 522 nodes, cytoHubba of Cytoscape was used to perform calculations towards identifying the hub nodes in the network (Supplementary Data S1). All 12 methods of cytoHubba were applied [26,30] and the top 100 nodes from each algorithm were selected, resulting in a total of 109 macromolecules across 12 lists (Supplementary Table S2). Afterwards, the frequency of appearance of each node in the 12 lists was calculated and a threshold of ≥7 was set. Based on the results, 84 macromolecules with significant impact on *IL36RN* interactome network have been recognized, including 6 lncRNAs, 32 microRNAs and 46 genes (Supplementary Table S3).

Validation analysis of the methodology output has been performed using the final outcome of genes and non-coding RNA targets in order to study the success of identifying key functional genomic regions involved in the DITRA. For this purpose, results from other biological databases such as the Malacards database were combined [37,38]. At the gene level, 65% of the genes described in Malacards with a score greater than 5.3 also appear to have a correlation with DITRA based on the results of the described work including *IL36RN*, *CARD14*, *IL1RL2*, *IL36B*, *IL36G*, *IL1RAP*, *TNF*, *IL36A* and *IL1A* (Supplementary Table S4). The remaining genes including *AP1S3*, *IL17A*, *TNIP1*, *SERPINA3* and *LCE3B*, although described in the Malacards database, do not appear to be documented by a sufficient number of publications. Regarding other functional genomic regions that appear to participate in the course of DITRA manifestation, and specifically in non-coding regions, a literature review was conducted given that there is no biological database that associates non-coding regions with diseases.

Evaluation analysis discoveredseven novel candidate genes including *CSDE1*, *FNBP4*, *HNF4A*, *MECP2*, *PLEKHA1*, *TNPO2* and *TINCR*, and 38 candidate non-coding RNA targets that may play an important role in DITRA manifestation (Supplementary Table S4). The RNAenrich, MalaCards and PubMed databases were used to annotate the candidate genes and non-coding RNAs with specific knowledge such as related biological targets, biological pathways and diseases [24,28,37]. Based on the annotated information, three genes (*FNBP4, PLEKHA1* and *TINCR*), and 33 non-coding regions appear to be related to key biological pathways of DITRA progression (Supplementary Table S4). Moreover, all the suggested genomic regions have been correlated with psoriasis and other autoimmune diseases except for one gene (*TNPO2*) and one non-coding region (Supplementary Table S4). All proposed genomic regions were evaluated for their candidate contribution as biomarkers and therapeutic targets (Supplementary Table S5). The analysis indicated that 44 genetic and non-coding RNA (ncRNA) biomarkers and pharmacological targets were associated with a wide range of diseases. Among them, approximately 93% are identified as pharmacological targets, while all (100%) are classified as biomarkers. The most prominent contribution is observed in cancer, where nearly all entries are implicated in tumorigenesis. Notable representation is also observed in psoriasis and autoimmune diseases, particularly through lncRNAs such as *TINCR*, *MALAT1*, and *NORAD*, as well as miRNAs like miR-17-5p, miR-26b-5p, and let-7i-5p. These findings highlight the molecular complexity and recurring involvement of specific ncRNAs across multiple pathologies, offering multiple avenues for diagnostic and therapeutic exploitation.

**Figure 3:**
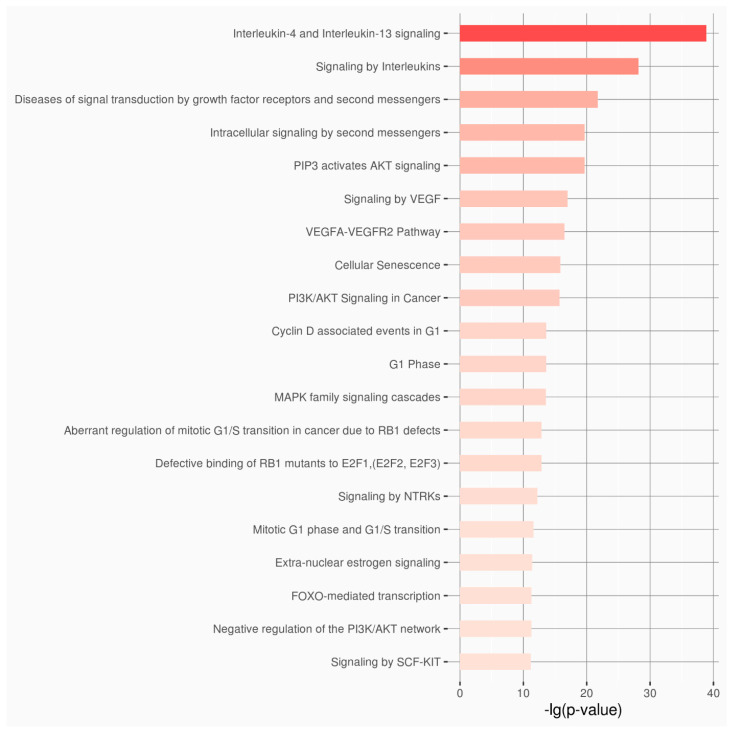
Top 20 highly enriched Reactome pathways for the 32 selected microRNAs.

**Figure 4:**
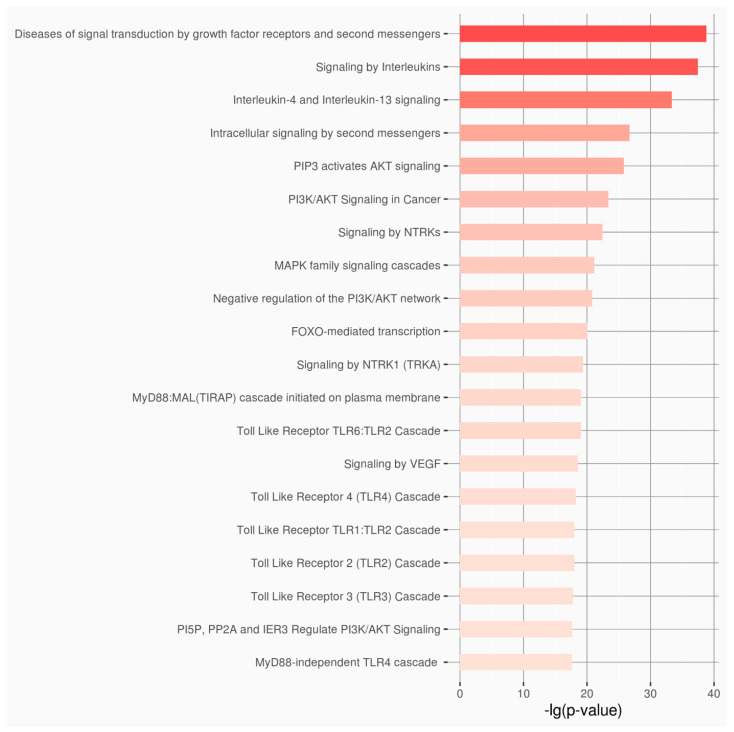
Top 20 highly enriched Reactome pathways for the 6 selected lncRNAs.

Two enrichment analyses were performed for the dataset of the lncRNAs and the dataset of the microRNA using the extracted information from the RNAenrich, and 5600 and 4361 biological pathways, respectively, were correlated (Supplementary Tables S6 and S7). Based on the results from the top 20 biological pathways of each dataset, there is a clear correlation with DITRA, psoriasis and dermatitis diseases (Figure 3 and Figure 4).

## 4. Discussion

### 4.1. Genetic and Epigenetic Context of DITRA and Psoriasis

Psoriasis is a multifactorial inflammatory skin disorder influenced by complex interactions between genetic predisposition and environmental triggers. Heritability estimates exceed 60%, and genome-wide association studies (GWASs) have identified at least 65 susceptibility loci. In contrast, Deficiency of Interleukin-36 Receptor Antagonist (DITRA) represents a monogenic autoinflammatory condition with a complex pathophysiology that remains incompletely understood linked to IL-36 signaling dysregulation [7]. While the *IL36RN* gene is central to disease development, recent advances in transcriptomics and interactome analyses have revealed that genes and non-coding RNAs (ncRNAs)—including microRNAs, long non-coding RNAs, and circular RNAs—play crucial roles in fine-tuning inflammatory pathways and interactome dynamics [39,40].

DITRA follows an autosomal recessive inheritance pattern and manifests as a subtype of generalized pustular psoriasis (GPP) in the absence of preceding plaque-type psoriasis. While psoriasis pathogenesis involves a complex genomic and epigenomic landscape, DITRA provides a more tractable model for exploring the effects of singular genetic defects and their regulatory modulation by epigenetic factors. Epigenetic regulation—including DNA methylation, histone modifications, and non-coding RNAs (ncRNAs)—is increasingly recognized as a critical layer of gene expression control, particularly in immune-mediated diseases such as psoriasis. It has been observed that certain epigenetic modifications are involved in psoriasis, including numerous differentially expressed ncRNAs playing a crucial regulatory role at the gene level [41].In the present study, the macromolecular interaction networks contributing to DITRA pathogenesis were investigated, focusing on the impact of genes and ncRNAs on *IL36Ra* interactome towards identifying potential biomarkers, risk factors and therapeutic targets.

### 4.2. Functional Implications of the Newly Discovered Genes in IL36RN Regulation

In this study, we propose seven genes of interest that may be implicated in DITRA (Deficiency of the IL-36 Receptor Antagonist), based on their involvement in psoriasis-associated pathways and relevant biological functions. These genes emerged through integrative analysis and are connected to signaling or regulatory processes already known to be dysregulated in psoriasis. *CSDE1* is associated with the C-MYC transcriptional repression pathway and functions in translational control and stress granule assembly; it also interacts with *TRIM33*, a known NF-κB regulator in psoriatic inflammation [42]. *FNBP4*, though without a defined pathway, is localized to nuclear specks and is elevated in psoriatic patients [43]. *HNF4A* participates in key signaling cascades such as AMPK, TGF-β, ERK, and MAPK, all of which are relevant to keratinocyte regulation and DITRA pathophysiology [44]. *MECP2*, an epigenetic regulator, affects transcription and immune signaling pathways such as ALK and GABA and has been linked to autoimmune responses in psoriasis [44]. *PLEKHA1* is involved in CD28/T-cell and PI3K signaling, both of which are central to DITRA and psoriatic inflammation [45]. *TNPO2*, although largely unexplored in dermatology, participates in nuclear transport and AKT signaling, which may intersect with immune regulatory mechanisms—this gene represents an uncharted but potentially important target [46]. Lastly, *TINCR*, currently annotated as a gene, has also been suggested to function as an LNC, although its exact classification remains under investigation. It plays a crucial role in epidermal differentiation by stabilizing mRNAs of key structural proteins and is involved in the activation of MAPK and Wnt/β-catenin signaling pathways—processes relevant to both psoriasis and potentially to DITRA pathophysiology [47,48]. Although the association of these genes with DITRA remains hypothetical, their roles in psoriasis and key inflammatory or differentiation pathways suggest a compelling rationale for further investigation.

### 4.3. Functional Implications of lncRNAs in IL36RN Regulation

Our analysis identified 38 ncRNAs included in *IL36RN* interactome, 6 lncRNAs (*MALAT1*, *NEAT1*, *SNHG16*, *TUG1*, *MIR17HG*, *NORAD*) and 32 microRNAs. The proposed ncRNAs for the pathogenesis of DITRA interfere with interleukin signaling pathways as expected, since DITRA has an autoimmune and autoinflammatory background. Numerous intracellular signaling pathways are also affected leading to probable changes in cell metabolism, proliferation, migration and cell death, mechanisms that possibly affect the overall behavior of epithelial and immune cells of DITRA patients. For the top 25 signaling pathways, enrichment analysis revealed that 56% (13/25) are related to psoriasis and 28% (7/25) are related to DITRA regarding the lncRNAs, whereas 72% (18/25) are related to psoriasis and only 16% (4/25) are related to DITRA regarding the miRNAs. This difference between the two diseases may be attributed to the fact that DITRA has an autosomal recessive pattern of inheritance, whereas psoriasis is the result of genetic and environmental factors combination; however, the low percentage of miRNA interference in DITRA intracellular signaling pathways needs to be further investigated. Interestingly, PI3K/AKT signaling pathway seems to be associated with both psoriasis and DITRA, with no previous data published; thus, in vitro or in vivo studies are proposed to unveil its possible implication in psoriasis and/or DITRA pathogenesis.

Thirty-three out of the thirty-eight lncRNAs are proposed for their possible association with DITRA, yet data for miR-30e-5p, let-7d-5p, miR-301a-3p, miR-301b-3p, miR-495-3p cannot confirm their possible implication in DITRA pathogenesis and subsequent studies have been proposed. Most lncRNAshave previously been reported to be correlated with psoriasis. LncRNA *MALAT1* was found to be upregulated in psoriasis [40,49]. By interacting with NF-κB, it inhibits lps-induced maturation of DCs, reduces T cell proliferation, and induces the generation of Treg cells [49,50]. LncRNA *MALAT1* is also associated with tolerance functions of DCs and induction of immune tolerance [41]. LncRNA *NEAT1* is also upregulated in 3 mm deep psoriatic lesion tissue samples and is positively correlated with the expression levels of inflammatory factors, including *IL-6*, *IL-8*, *TNF-α*, *IL-17*, and *IL-22* [51]. On the other hand, the lncRNA *NEAT1* is expressed at low levels in the skin tissues of patients with psoriasis. The upregulation of *NEAT1* is reported to result in the targeted mediation of downstream miR-3194-5p to increase Galectin-7 expression, which subsequently inhibits the activity of psoriasis HaCat cells and exhibits therapeutic effects [52]. LncRNA *TUG1* is also downregulated in psoriasis patients. The down-regulation of TUG-1 is associated with both up-regulation of miRNA-377 and down-regulation PPAR-γ, which may contribute to the inflammatory cascade responses and chronicity that characterize psoriasis [53]. MIR17HG is a precursor RNA for the mir17-92 cluster, which haspreviously been reported to promote the proliferation and the chemokine production of keratinocytes, being implicated in psoriasis pathogenesis [54]. LncRNA *NORAD* also engages in psoriasis by binding to miR-26a to regulate keratinocyte proliferation [51]. lncRNA *SNHG16* has been found to regulate miR-146a/b in other inflammation-related diseases and it is hypothesized that it is the upstream factor affecting miR-146a/b in psoriasis [55].

### 4.4. miRNAs in Inflammation and Autoimmunity

Concerning miRNAs, it has been identified that hsa-miR-19a-3p has great potential to become a biomarker for psoriasis because it binds to the mRNA of seven genes (*CAST*, *TSC1*, *SPATA2*, *ERAP1*, *TNIP1*, *ERBB3* and *SDC4*) thatare associated with psoriasis [56]. MiRNA hsa-let-7c-5p is found to be upregulated in psoriasis patients [57]. However, another study found MiRNA hsa-let-7c-5p to be downregulated in psoriasis. MiRNA hsa-let-7c-5p targeted *CDKN1A* and *CDC25A*, *STAT3*, *IL1B* genes and JAK/STAT3 signaling pathways, causing perturbations in cell cycle progression, migration and Th17 cell differentiation [58]. Similar results were also found in another study concerning the development of psoriatic lesions, which showed a significant trend towards a 2-fold decrease in hsa-let-7c-5p compared to the corresponding non-lesional skin [59]. Likewise, miRNAs hsa-let-7a-5p, hsa-let-7b-5p, hsa-let-7d-5p and hsa-let-7i-5p have been associated with psoriasis being either upregulated or downregulated [57,59].

Furthermore, hsa-miR-106a-5p and miR-17-5p show a significant trend of increasing in lesional skin of psosiaris patients [59]. Hhsa-miR-106a-5p, which is part of the in vitro CD4 T cell-derived miRNA signature, is upregulated in serum from psoriasis patients and it is specific in Th1/Th17 cell-derived extracellular vesicles (EVs) in vitro [60]. hsa-miR-122-5p and hsa-miR-124-3p are found upregulated and downregulated, respectively, in psoriatic lesions and keratinocytes targeting *SPRY2* and *FGFR2* gene expression, which causes alterations in keratinocyte proliferation and migration [61,62]. hsa-miR-30e-5p, hsa-miR-106b-5p, hsa-miR-26b-5p and hsa-miR-130a- 3p are also related to psoriasis, with hsa-miR-30e-5p, hsa-miR-106b-5p and hsa-miR-130a- 3p being found significantly down-regulated in psoriasis patients’ blood [57]. For hsa-miR-186-5p, data correlate its down-regulation with psoriatic arthritis [63]. Interestingly, even though precursors hsa-miR-301, hsa-miR-302 and hsa-miR-495 are associated with psoriasis, there areno clear data to show the implication of hsa-miR-301a-3p, hsa-miR-301b-3p and hsa-miR-495-3p supporting a role in DITRA pathogenesis, highlighting novel candidates for further investigation.

### 4.5. Clinical and Experimental Relevance

Keratinocytes expressing *IL-36* and their antagonist *IL-36RN* are known to induce the expression of inflammatory cytokines and chemokines and are potent sources of macrophage, T cell, and neutrophil chemokines, while *IL-36* regulates keratinocyte- and endothelial cell-mediated inflammatory response, plays an important role in innate immune response and is important in effector T-cell differentiation [21]. Given the fact that *IL-36* regulates the function of both non-immune cells and immune cells, the ncRNAs mentioned above, which appear to be differentially expressed in keratinocytes, immune cells and serum of psoriasis patients, may serve as accessible biomarkers, and their regulatory roles suggest therapeutic potential as well, particularly in modulating IL-36-dependent inflammation.

However, for several candidate miRNAs (e.g., miR-30e-5p, miR-106b-5p, miR-130a-3p, miR-186-5p), their role in DITRA remains speculative and necessitates in vitro and in vivo validation. Similarly, lncRNAs with indirect or inconsistent involvement in psoriatic pathways need targeted functional studies.

### 4.6. A Molecular Interaction Network Perspective

In this context, computational and bioinformatics analyses offer unique and essential contributions to unraveling the layers of regulatory complexity of several diseases including in DITRA [64]. Specifically, mapping and analyzing the *IL36RN* interactome and identifying potential ncRNA-mediated regulatory interactions can 1. elucidate novel layers of post-transcriptional regulation, providing mechanistic insights that go beyond protein-coding genes [65]; 2. highlight potential therapeutic targets and biomarkers, as ncRNAs increasingly emerge as key regulators of inflammatory signaling [39]; 3.integrate disparate experimental findings into a cohesive, systems-level framework that better reflects the in vivo biology of DITRA [66].

We must drive forward future experimental efforts, prioritizing ncRNA targets or interactions for validation in cellular or animal models. Although this study does not generate new experimental data, it leverages robust computational approaches to mine existing data and build an integrated network view of *IL36RN* regulation in DITRA. Such integrative analyses are crucial in the current era of high-throughput biology, where the accumulation of data outpaces our ability to experimentally validate every hypothesis [66]. By offering a systematic and reproducible computational framework, this work fills a critical knowledge gap and provides a roadmap for targeted experimental studies to validate key regulatory interactions in DITRA. Therefore, even in the absence of new laboratory data, this computational dissection of the *IL36RN* interactome and its non-coding RNA regulators in DITRA represents a significant and necessary step towards a deeper understanding of the disease’s molecular underpinnings and the identification of novel avenues for therapeutic intervention.

## 5. Conclusions

Deciphering the regulatory mechanisms of the human genome has long posed a significant challenge, particularly with regard to the functional roles of non-coding DNA sequences. Ongoing research aims to link phenotypic manifestations, such as disease states, with gene activity and intracellular signaling pathways. Despite considerable progress, establishing definitive correlations remains difficult in complex, multifactorial diseases like psoriasis, which are influenced by both genetic and environmental components. However, monogenic disorders such as DITRA provide a more tractable model for such investigations.

In this study, we identified specific epigenetic regulators—namely, non-coding RNAs (ncRNAs)—that may contribute to the pathogenesis of DITRA. Many of the ncRNAs highlighted here have previously been implicated in inflammatory conditions including psoriasis, rheumatoid arthritis, and atopic dermatitis. Notably, we also uncovered potentially novel ncRNAs not yet associated with these disorders. A total of 38 non-coding RNAs were identified within the extended IL36RN interactome, comprising 6lncRNAs and 32 miRNAs. Of these, 5remain to be discovered and 33 ncRNAs were mapped to signaling pathways functionally relevant to DITRA pathogenesis, including *IL-36*, *NF-κB*, and PI3K/AKT signaling. Additionally, seven protein-coding genes emerged as central nodes, among which *TINCR*, *PLEKHA1*, and *HNF4A* demonstrated direct enrichment in DITRA-related biological processes and gene ontology terms. Several candidate ncRNAs, while unlinked to DITRA in prior studies, show regulatory interactions consistent with immune-mediated skin inflammation. Many of the identified ncRNAs have prior associations with immune-mediated diseases, including psoriasis, arthritis and dermatitis supporting their potential relevance in DITRA pathogenesis. Further research is warranted to elucidate the roles of these ncRNAs in DITRA and to explore their potential as biomarkers or therapeutic targets. Our findings contribute to a deeper understanding of the genetic and epigenetic mechanisms underlying this rare autoinflammatory condition.

## Figures and Tables

**Table 1:** Major macromolecules categories in the main interaction network.

Category of Functional Genomic Regions	Total
miRNA	333
Genes	120
lncRNA	36
Pseudogenes	9
snRNA	6
rRNA	3
eRNA	2
Unknown Regions	2
snoRNA	1

## Data Availability

All data and materials are provided as supplementary files.

## Supplementary Materials

The following supporting information can be downloaded at https://www.mdpi.com/article/10.3390/genes16070753/s1: Supplementary Table S1: The main interaction network analyzed using Cytoscape; Supplementary Table S2: Output of the cytoHubba 12 algorithms for all network nodes; Supplementary Table S3: Frequency of the most important nodes in the 100 top hits of each cytoHubba method; Supplementary Table S4: The proposed genomic regions of genes and non-coding RNAs that may relate toDITRA; Supplementary Table S5: The proposed genomic regions as candidate biomarkers and pharmacological targets; Supplementary Table S6:Enrichment analysis for the 6 selected lncRNAs; Supplementary Table S7: Enrichment analysis for the 32 selected microRNAs. Supplementary Data S1: The output file of Cytoscape.

## Abbreviations

The following abbreviations are used in this manuscript:

DITRA	Deficiency of Interleukin-36 Receptor Antagonist
GPP	Generalized Pustular Psoriasis
ACH	Hallopeau’s Continuous Acrodermatitis
IL36Ra	IL-36 Receptor Antagonist
LPP	Localized Pustular Psoriasis
MAPKs	Mitogen-activated Protein Kinases
NF-kB	Nuclear Factor-Κb
PaP	Palmoplantar Psoriasis
PP	Pustular Psoriasis
PPP	Pustular Psoriasis of Pregnancy
