# Supplementary material for: Decoding Non-Coding RNA Regulators in DITRA: From Genomic Insights to Potential Biomarkers and Therapeutic Targets
Source: Genes (Basel). 2025 Jun 27;16(7):753. doi: 10.3390/genes16070753 (PMC12295128; doi:10.3390/genes16070753)
Supplement: Supplementary file 1 [file genes-16-00753-s001.zip › Supplementary-TableS1.pdf]

**Node_A**	**Node_B**
CARD14	IL36RN
CARD14	IL1RL2
IL1A	IL2
IL1A	IL1RL1
IL1A	IL36G
IL1A	IL1RN
IL1A	IL36A
IL1A	IL36RN
IL1A	IL36B
IL1A	SIGIRR
IL1A	IL1RL2
IL1A	IRAK1
IL1A	IL1RAP
IL1A	IL1R2
IL1A	IL1B
IL1A	IL1R1
IL1B	IL2
IL1B	IL1RL1
IL1B	IL36G
IL1B	IL1RN
IL1B	IL36A
IL1B	IL36RN
IL1B	IL36B
IL1B	SIGIRR
IL1B	IL1RL2
IL1B	IRAK1
IL1B	IL1R2
IL1B	IL1R1
IL1B	IL1RAP
IL1R1	IL2
IL1R1	IL1RL1
IL1R1	IL36G
IL1R1	IL1RN
IL1R1	IL36A
IL1R1	IL36RN
IL1R1	IL36B
IL1R1	IL1RL2
IL1R1	IL1RAP
IL1R1	IL1R2
IL1R1	IRAK1
IL1R1	SIGIRR
IL1R2	IL2

IL1R2	IL1RL1
IL1R2	IL36G
IL1R2	IL1RN
IL1R2	IL36RN
IL1R2	IL1RAP
IL1R2	SIGIRR
IL1RAP	IL1RL1
IL1RAP	IL36G
IL1RAP	IL1RN
IL1RAP	IL36A
IL1RAP	IL36RN
IL1RAP	IL36B
IL1RAP	IL1RL2
IL1RAP	SIGIRR
IL1RAP	IRAK1
IL1RL1	IL2
IL1RL1	IL36RN
IL1RL1	IL36G
IL1RL1	IL1RL2
IL1RL1	IL1RN
IL1RL1	IRAK1
IL1RL2	IL36G
IL1RL2	IL1RN
IL1RL2	IL36A
IL1RL2	IL36RN
IL1RL2	IL36B
IL1RN	IL2
IL1RN	IL36A
IL1RN	IL36RN
IL1RN	IL36B
IL2	IRAK1
IL2	IL36B
IL2	IL36RN
IL36A	IL36G
IL36A	IL36B
IL36A	IL36RN
IL36B	IL36G
IL36B	IL36RN
IL36G	IL36RN
IL36RN	SIGIRR
IRAK1	SIGIRR
28S_RRNA	IRAK1
AC005477	IL1A

AC078899.1	IL1A
AC245014.3	IL1A
ACO1	IL1A
AFAP1-AS1	IL1A
AFAP1-AS1	IL1B
AFAP1-AS1	IL1RL1
AL049794	IL1RAP
ANCR	IL1B
ANCR	IL1R2
ANCR	IL1RN
ANCR	IL36G
ANCR	IL36RN
AR	CARD14
AR	IL1A
AR	IL1B
AR	IL1R1
AR	IL1R2
AR	IL1RAP
AR	IL1RL1
AR	IL1RL2
AR	IL1RN
AR	IL2
AR	IL36A
AR	IL36B
AR	IL36G
AR	IL36RN
AR	IRAK1
AR	SIGIRR
ARHGEF10	IL1A
ARL6	IL1R2
ATP8	IL1A
BANCR	IL2
BEND4	IL1A
BNC1	IL1A
C17orf58	IL1A
C5orf60	IL1A
CARD14	AC091045.1
CARD14	PPM1K
CARD14	RNR2
CARD14	SAMD4B
CARD14	TFAP2A-AS2
CARM1	IL1A
CAT2	IL36RN

CAT6	IL36RN
CCDC124	IL1A
CCDC168	IL1R1
CCDC89	IL1RAP
CDCP1	IL1A
CHMP1B	IL1A
CHMP3	IL1A
CISTR	IL1A
CISTR	IL1B
CLIP1	IL1A
CRNDE	IL36B
CSDE1	IL1A
DEPDC1B	IL36A
DMXL2	IL1A
DRAIC	IL2
DRAIC	IL36RN
EEF1A1P9	IL1A
EML2	IRAK1
ERCC4	IL1A
ETS1-AS1	IL1RL2
FAM214B	IL1A
FENDRR	IL1B
FENDRR	SIGIRR
FNBP4	IL1A
FZR1	IRAK1
GDF2	IL1A
GPR61	IRAK1
GRB10	IL1A
GTF2F1	IL1A
GTF2H2	IL1RAP
HID1	IL1A
HP1BP3	IL1A
IL10RB-DT	IL1A
IL1A	AL353746.1
IL1A	INTS3
IL1A	KRT6A
IL1A	L3MBTL2
IL1A	LOC101928012
IL1A	MBD1
IL1A	MROH1
IL1A	MTND1P23
IL1A	MTND4P12
IL1A	NARS2

IL1A	ND1
IL1A	ND2
IL1A	ND4
IL1A	ND5
IL1A	NEAT1
IL1A	NME1-NME2
IL1A	NRCP
IL1A	OPCML-IT1
IL1A	PCDH19
IL1A	PLCG1
IL1A	PLEKHA1
IL1A	PLPP7
IL1A	POLR2A
IL1A	PPP2R3A
IL1A	PRMT2
IL1A	PRPF31
IL1A	PRRG3
IL1A	PSMB5
IL1A	PTCH1
IL1A	RABAC1
IL1A	RAD51-AS1
IL1A	RBM28
IL1A	RBMS1
IL1A	RN7SKP119
IL1A	RNA5-8SN3
IL1A	RNA5-8SP6
IL1A	RNA5SP298
IL1A	RNU1-106P
IL1A	RNU1-138P
IL1A	RNU1-148P
IL1A	RNVU1-32
IL1A	RNVU1-7
IL1A	SENCR
IL1A	SLC12A2
IL1A	SLC35C2
IL1A	SNORA73B
IL1A	SOWAHB
IL1A	SRPRA
IL1A	SRRM2
IL1A	SSH1
IL1A	STS
IL1A	TBRG4
IL1A	TEX101

IL1A	TG
IL1A	TMEM201
IL1A	TMTC2
IL1A	TNFRSF14
IL1A	TNPO2
IL1A	U1
IL1A	USP38
IL1A	ZIC3
IL1A	ZNF318
IL1A	ZNF354B
IL1B	IL1beta-RBT46
IL1B	IL1beta-eRNA
IL1B	MALAT1
IL1B	NRCP
IL1B	PLAUR
IL1B	SBF2-AS1
IL1B	lnrCXCR4
IL1R1	LOC101928266
IL1R1	ND2
IL1R1	SENCR
IL1R1	SENP5
IL1R1	TUG1
IL1R2	lnrCXCR4
IL1RAP	AC005183.1
IL1RAP	LTBP1
IL1RAP	lincZFP161
IL1RL1	MAGI1
IL1RL1	MAGI1-IT1
IL1RL1	NDUFV3
IL1RL1	OR8A1
IL1RN	MPPE1
IL1RN	NORAD
IL1RN	TINCR
IL1RN	ZMYND11
IL2	NDE1
IL2	TFAP2A-AS2
IL2	lnrCXCR4
IL36A	AC053481.3
IL36B	NORAD
IL36B	SBF2-AS1
IL36RN	AL158829.1
IL36RN	KHSRP
IRAK1	MECP2

IRAK1	MIR17HG
IRAK1	NSRP1
IRAK1	PSD4
IRAK1	RNU4-2
IRAK1	RPL30
IRAK1	SNHG16
IRAK1	SOX9
IRAK1	TIMP2
IRAK1	TINCR
IRAK1	TPPP
IRAK1	TUG1
IRAK1	hsa-miR-326
IRAK1	hsa-miR-330-3p
IRAK1	hsa-miR-370
IRAK1	hsa-miR-371a-3p
IRAK1	hsa-miR-6893-3p
IRAK1	lincMTX2
IRAK1	lnrCXCR4
LOC107987013	IL36RN
SBF2-AS1	SIGIRR
SIGIRR	TENM1
SIGIRR	TUG1
SIGIRR	lincTNS1
SIGIRR	lincZFP161
hcmv-miR-US25-1-5p	CARD14
hcmv-miR-US25-1-5p	IL1A
hcmv-miR-US25-1-5p	IL1B
hcmv-miR-US25-1-5p	IL1R1
hcmv-miR-US25-1-5p	IL1R2
hcmv-miR-US25-1-5p	IL1RAP
hcmv-miR-US25-1-5p	IL1RL1
hcmv-miR-US25-1-5p	IL1RL2
hcmv-miR-US25-1-5p	IL1RN
hcmv-miR-US25-1-5p	IL2

hcmv-miR-US25-1-5p	IL36A
hcmv-miR-US25-1-5p	IL36B
hcmv-miR-US25-1-5p	IL36G
hcmv-miR-US25-1-5p	IL36RN
hcmv-miR-US25-1-5p	IRAK1
hcmv-miR-US25-1-5p	SIGIRR
hsa-let-7a-5p	IL1R1
hsa-let-7a-5p	IL1RN
hsa-let-7b-5p	IL1RN
hsa-let-7c-5p	IL1RN
hsa-let-7d-3p	IL1RN
hsa-let-7d-5p	IL1R1
hsa-let-7d-5p	IL1RN
hsa-let-7e-5p	IL1R1
hsa-let-7e-5p	IL1RN
hsa-let-7f-2-3p	IL1RN
hsa-let-7f-5p	IL1R1
hsa-let-7f-5p	IL1RN
hsa-let-7g-5p	IL1R1
hsa-let-7g-5p	IL1RN
hsa-let-7i-5p	IL1RN
hsa-let-7i-5p	IL2
hsa-miR-1-3p	IL1R1
hsa-miR-1-3p	IL1RAP
hsa-miR-1-3p	IL1RN
hsa-miR-101-3p	IL1B
hsa-miR-101-3p	IL1RAP
hsa-miR-103a-2-5p	IL1RAP
hsa-miR-103a-3p	IL1RAP
hsa-miR-103a-3p	IL1RL1
hsa-miR-103a-3p	IL1RN
hsa-miR-105-3p	IL1RN
hsa-miR-105-5p	IL1RAP
hsa-miR-105-5p	IL1RL1
hsa-miR-105-5p	IL1RN
hsa-miR-106a-3p	IL1RAP
hsa-miR-106a-5p	IL1B
hsa-miR-106a-5p	IL1RAP

hsa-miR-106b-3p	IL1RAP
hsa-miR-106b-5p	IL1RAP
hsa-miR-107	IL1RAP
hsa-miR-107	IL1RL1
hsa-miR-107	IL1RN
hsa-miR-1179	IL1R1
hsa-miR-1179	IL1RAP
hsa-miR-1180-3p	IRAK1
hsa-miR-1185-5p	IL1A
hsa-miR-1185-5p	IRAK1
hsa-miR-1193	IL1R1
hsa-miR-1197	IL1R1
hsa-miR-1197	IRAK1
hsa-miR-1205	IL1RN
hsa-miR-1207-3p	SIGIRR
hsa-miR-122-3p	IL1RN
hsa-miR-122-5p	IL1A
hsa-miR-122-5p	IL1R1
hsa-miR-122-5p	IL1RL1
hsa-miR-122-5p	IL1RN
hsa-miR-1224-5p	IRAK1
hsa-miR-1225-3p	IL1RN
hsa-miR-1226-3p	IRAK1
hsa-miR-1228-3p	IL1RAP
hsa-miR-1229-3p	IL1RL2
hsa-miR-1231	IRAK1
hsa-miR-1237-3p	IRAK1
hsa-miR-124-3p	CARD14
hsa-miR-124-3p	IL1B
hsa-miR-124-3p	IL1R1
hsa-miR-124-3p	IL1RAP
hsa-miR-1249-3p	IL1RAP
hsa-miR-1252-5p	IL1R1
hsa-miR-1252-5p	IL1RAP
hsa-miR-125a-3p	IL1A
hsa-miR-125a-3p	IRAK1
hsa-miR-125a-5p	IL1RN
hsa-miR-125a-5p	IRAK1
hsa-miR-125b-5p	IRAK1
hsa-miR-1270	IL1RN
hsa-miR-1271-5p	IL1A
hsa-miR-1271-5p	IRAK1
hsa-miR-1273f	IL36B

hsa-miR-1275	IRAK1
hsa-miR-1278	IL1RN
hsa-miR-128-3p	IL1RAP
hsa-miR-1285-3p	IL1RAP
hsa-miR-1286	IL1RN
hsa-miR-1289	IL1RN
hsa-miR-1297	IL1A
hsa-miR-1297	IL1RAP
hsa-miR-130a-3p	IL1RAP
hsa-miR-130b-3p	IL1RAP
hsa-miR-132-3p	IL1A
hsa-miR-132-3p	IRAK1
hsa-miR-133a-3p	IRAK1
hsa-miR-134-5p	IRAK1
hsa-miR-135a-5p	IL1R1
hsa-miR-135a-5p	IL1RAP
hsa-miR-135b-5p	IL1R1
hsa-miR-140-5p	IL1A
hsa-miR-140-5p	IL1RAP
hsa-miR-141-3p	IL1RAP
hsa-miR-141-3p	IL1RN
hsa-miR-142-3p	IL1A
hsa-miR-143-3p	IL1RAP
hsa-miR-144-3p	IL1RAP
hsa-miR-146a-3p	IRAK1
hsa-miR-146a-5p	IL1RAP
hsa-miR-146a-5p	IL1RL2
hsa-miR-146b-3p	IRAK1
hsa-miR-146b-5p	IL1B
hsa-miR-146b-5p	IL1RAP
hsa-miR-146b-5p	IL1RL2
hsa-miR-146b-5p	IRAK1
hsa-miR-147a	IL1RL1
hsa-miR-149-3p	SIGIRR
hsa-miR-149-5p	IL1A
hsa-miR-149-5p	SIGIRR
hsa-miR-150-3p	IRAK1
hsa-miR-150-5p	IRAK1
hsa-miR-152-3p	IL1A
hsa-miR-152-3p	IRAK1
hsa-miR-153-3p	IL1RAP
hsa-miR-155-5p	IL2
hsa-miR-16-1-3p	IL36RN

hsa-miR-16-5p	IL36RN
hsa-miR-17-3p	IL1RAP
hsa-miR-17-5p	IL1RAP
hsa-miR-17-5p	IRAK1
hsa-miR-181a-5p	IL1A
hsa-miR-181a-5p	IL1RAP
hsa-miR-181b-5p	IL1A
hsa-miR-181b-5p	IL1RAP
hsa-miR-181c-5p	IL1A
hsa-miR-181c-5p	IL1RAP
hsa-miR-181c-5p	IL2
hsa-miR-181d-3p	IL1R1
hsa-miR-181d-5p	IL1A
hsa-miR-181d-5p	IL1RAP
hsa-miR-182-3p	IL1RAP
hsa-miR-182-5p	IL1RAP
hsa-miR-184	IL1R1
hsa-miR-186-5p	IL1A
hsa-miR-186-5p	IL1RAP
hsa-miR-186-5p	IL36RN
hsa-miR-18a-5p	IL1RAP
hsa-miR-18b-3p	IL1RAP
hsa-miR-18b-5p	IL1RAP
hsa-miR-1909-3p	SIGIRR
hsa-miR-190a-5p	IL1A
hsa-miR-190a-5p	IL1RAP
hsa-miR-190b-5p	IL1A
hsa-miR-190b-5p	IL1RAP
hsa-miR-191-5p	IL1A
hsa-miR-1914-3p	IRAK1
hsa-miR-1915-3p	SIGIRR
hsa-miR-192-5p	IL1R1
hsa-miR-192-5p	IL1RAP
hsa-miR-192-5p	IRAK1
hsa-miR-193b-3p	IL1A
hsa-miR-193b-3p	IL1R1
hsa-miR-193b-3p	IRAK1
hsa-miR-196a-5p	IL1A
hsa-miR-197-3p	IL1R1
hsa-miR-197-3p	IL36RN
hsa-miR-1976	IL1RAP
hsa-miR-198	IL1RAP
hsa-miR-198	IL1RN

hsa-miR-199a-3p	IL1RAP
hsa-miR-19a-3p	IL1A
hsa-miR-19a-3p	IL1R1
hsa-miR-19a-3p	IL1RAP
hsa-miR-19b-1-5p	IL1R1
hsa-miR-19b-1-5p	IL1RAP
hsa-miR-19b-3p	IL1A
hsa-miR-19b-3p	IL1R1
hsa-miR-19b-3p	IL1RAP
hsa-miR-200a-3p	IL1B
hsa-miR-200a-3p	IL1RAP
hsa-miR-200b-3p	IL1B
hsa-miR-200c-3p	IL1B
hsa-miR-203a-3p	IL1A
hsa-miR-204-5p	IL1B
hsa-miR-204-5p	IL1R1
hsa-miR-204-5p	IL1RAP
hsa-miR-204-5p	IRAK1
hsa-miR-205-5p	IL1A
hsa-miR-205-5p	IL1R1
hsa-miR-2054	IL1RAP
hsa-miR-206	IL1R1
hsa-miR-20a-3p	IL1RAP
hsa-miR-20a-5p	IL1RAP
hsa-miR-20b-3p	IL1RAP
hsa-miR-20b-5p	IL1RAP
hsa-miR-21-5p	IL1B
hsa-miR-21-5p	IL1RAP
hsa-miR-21-5p	IRAK1
hsa-miR-211-5p	IL1B
hsa-miR-211-5p	IL1R1
hsa-miR-211-5p	IRAK1
hsa-miR-212-3p	IL1A
hsa-miR-214-3p	IL1R1
hsa-miR-214-3p	IL1RAP
hsa-miR-214-3p	IRAK1
hsa-miR-215-5p	IL1R1
hsa-miR-215-5p	IL1RAP
hsa-miR-215-5p	IRAK1
hsa-miR-216a-3p	IL1RL1
hsa-miR-218-2-3p	IL1RN
hsa-miR-218-5p	IL36G
hsa-miR-221-3p	IL1RAP

hsa-miR-222-3p	IL1RAP
hsa-miR-222-3p	IRAK1
hsa-miR-2355-5p	IL1RL1
hsa-miR-23a-3p	IL1RAP
hsa-miR-23b-3p	IL1RAP
hsa-miR-23c	IL1B
hsa-miR-24-1-5p	IL1A
hsa-miR-24-2-5p	IL1A
hsa-miR-24-3p	IL1A
hsa-miR-24-3p	IL1B
hsa-miR-24-3p	IL1R1
hsa-miR-26b-5p	CARD14
hsa-miR-26b-5p	IL1A
hsa-miR-26b-5p	IL1R2
hsa-miR-26b-5p	IL1RAP
hsa-miR-26b-5p	IL1RL1
hsa-miR-26b-5p	IL36RN
hsa-miR-27a-3p	IL1RAP
hsa-miR-27b-3p	IL1RAP
hsa-miR-296-3p	IL1RAP
hsa-miR-29a-3p	IL1RAP
hsa-miR-29b-2-5p	IL1RAP
hsa-miR-29b-3p	IL1RAP
hsa-miR-29c-3p	IL1RAP
hsa-miR-301a-3p	IL1RAP
hsa-miR-301b-3p	IL1RAP
hsa-miR-30a-5p	CARD14
hsa-miR-30a-5p	IL1A
hsa-miR-30b-3p	IL1RL1
hsa-miR-30b-5p	CARD14
hsa-miR-30b-5p	IL1A
hsa-miR-30c-5p	CARD14
hsa-miR-30c-5p	IL1A
hsa-miR-30d-5p	CARD14
hsa-miR-30d-5p	IL1A
hsa-miR-30e-5p	CARD14
hsa-miR-30e-5p	IL1A
hsa-miR-31-3p	IL1RAP
hsa-miR-31-5p	IL1R1
hsa-miR-31-5p	IL1RAP
hsa-miR-31-5p	IRAK1
hsa-miR-3116	IL1RN
hsa-miR-3118	IRAK1

hsa-miR-3120-3p	IRAK1
hsa-miR-3133	IL1RN
hsa-miR-3158-3p	IL1RN
hsa-miR-3163	IL1RAP
hsa-miR-3183	IL1RAP
hsa-miR-3183	IL1RN
hsa-miR-3198	IL1RN
hsa-miR-3199	IL1RN
hsa-miR-3199	IRAK1
hsa-miR-323b-5p	IL1RAP
hsa-miR-324-3p	IL1RAP
hsa-miR-324-3p	IRAK1
hsa-miR-325	IL1RAP
hsa-miR-328	IRAK1
hsa-miR-328-3p	IL1RAP
hsa-miR-328-3p	IRAK1
hsa-miR-330-5p	IRAK1
hsa-miR-335-5p	IL1A
hsa-miR-335-5p	IL36RN
hsa-miR-335-5p	SIGIRR
hsa-miR-339-3p	IRAK1
hsa-miR-339-5p	IRAK1
hsa-miR-339-5p	SIGIRR
hsa-miR-33a-3p	IRAK1
hsa-miR-33a-5p	IL1RAP
hsa-miR-342-3p	IL1RN
hsa-miR-346	IRAK1
hsa-miR-34a-5p	IL1A
hsa-miR-34a-5p	IL1RAP
hsa-miR-34b-3p	IRAK1
hsa-miR-34c-3p	IL1RN
hsa-miR-34c-5p	IL1R1
hsa-miR-34c-5p	IL1RN
hsa-miR-3529-3p	IRAK1
hsa-miR-361-3p	IL1RL2
hsa-miR-363-3p	IL1RAP
hsa-miR-365a-3p	IL1A
hsa-miR-365a-3p	IL1RAP
hsa-miR-365a-3p	IRAK1
hsa-miR-3666	IL1RAP
hsa-miR-3667-3p	IL1R1
hsa-miR-371a-3p	IL1RAP
hsa-miR-373-3p	IL1RAP

hsa-miR-373-3p	IRAK1
hsa-miR-374a-5p	IL1A
hsa-miR-374b-5p	IL1A
hsa-miR-375-3p	IL1RAP
hsa-miR-376c-3p	IL1RAP
hsa-miR-378a-5p	IL1R1
hsa-miR-381-3p	IRAK1
hsa-miR-382-3p	IRAK1
hsa-miR-382-5p	IL1R1
hsa-miR-421	IL1RAP
hsa-miR-422a	IL1RAP
hsa-miR-423-3p	IRAK1
hsa-miR-425-5p	IL1RAP
hsa-miR-4253	IL1RAP
hsa-miR-4256	IRAK1
hsa-miR-4262	IL1A
hsa-miR-4267	IL1RL2
hsa-miR-4269	IL1RN
hsa-miR-4277	IL1RAP
hsa-miR-4282	IL1RAP
hsa-miR-4284	IL1R1
hsa-miR-4287	IL1RN
hsa-miR-4288	IRAK1
hsa-miR-4292	IRAK1
hsa-miR-4294	IL1RN
hsa-miR-4295	IL1RAP
hsa-miR-4297	IRAK1
hsa-miR-4301	IL1RL2
hsa-miR-4309	IL1RN
hsa-miR-431-5p	IL1RAP
hsa-miR-4310	IL1RAP
hsa-miR-432-3p	IL1RAP
hsa-miR-432-5p	IL1R1
hsa-miR-4326	IL1RAP
hsa-miR-4329	IL1RN
hsa-miR-448	IL1RAP
hsa-miR-449a	IL1A
hsa-miR-449a	IL1R1
hsa-miR-449a	IL1RAP
hsa-miR-452-3p	IL1R1
hsa-miR-454-3p	IL1RAP
hsa-miR-4635	IL1RAP
hsa-miR-4695-3p	CARD14

hsa-miR-4695-5p	IL1RL1
hsa-miR-4732-5p	IL36B
hsa-miR-484	IL2
hsa-miR-485-5p	IL1RAP
hsa-miR-485-5p	IRAK1
hsa-miR-490-3p	IL1RAP
hsa-miR-490-3p	IL1RN
hsa-miR-490-3p	IRAK1
hsa-miR-491-5p	IL1R1
hsa-miR-492	IL1RL1
hsa-miR-492	IL1RN
hsa-miR-494-3p	IL1A
hsa-miR-494-3p	IL1RAP
hsa-miR-495-3p	IL1RAP
hsa-miR-499a-5p	IL1RAP
hsa-miR-500b-3p	IL1RL2
hsa-miR-503-3p	IL2
hsa-miR-505-3p	IL1RN
hsa-miR-508-3p	IL1A
hsa-miR-508-3p	IL36B
hsa-miR-509-3-5p	IL1RN
hsa-miR-509-3p	IL1RN
hsa-miR-510-3p	IL1RAP
hsa-miR-511-5p	IL1R1
hsa-miR-511-5p	IRAK1
hsa-miR-513a-5p	IL1RAP
hsa-miR-514a-3p	IL1RN
hsa-miR-514b-3p	IL1RN
hsa-miR-515-3p	IL1RN
hsa-miR-516b-3p	IL1RN
hsa-miR-518c-5p	IRAK1
hsa-miR-519a-3p	IL1RAP
hsa-miR-519b-3p	IL1RAP
hsa-miR-519c-3p	IL1RAP
hsa-miR-519d-3p	IL1RAP
hsa-miR-519e-3p	IL1RN
hsa-miR-520a-3p	IRAK1
hsa-miR-524-3p	IL1RL1
hsa-miR-526b-3p	IL1RAP
hsa-miR-542-3p	IL1RAP
hsa-miR-543	IL1A
hsa-miR-543	IL1RAP
hsa-miR-548c-3p	IL1RAP

hsa-miR-548c-3p	IL1RN
hsa-miR-548d-5p	IL1B
hsa-miR-548k	IL1RN
hsa-miR-548t-5p	IL1RAP
hsa-miR-548u	IL1RN
hsa-miR-548v	IRAK1
hsa-miR-556-3p	IL1RN
hsa-miR-561-3p	IL1RL1
hsa-miR-569	IRAK1
hsa-miR-573	IL1RN
hsa-miR-578	IL1B
hsa-miR-580-3p	IL1RN
hsa-miR-584-5p	IL1RL1
hsa-miR-589-5p	IRAK1
hsa-miR-590-3p	IL1A
hsa-miR-590-3p	IL1R1
hsa-miR-590-3p	IL1RAP
hsa-miR-590-5p	IL1RAP
hsa-miR-591	IL1RL2
hsa-miR-591	IRAK1
hsa-miR-612	IL1RAP
hsa-miR-613	IL1R1
hsa-miR-617	IRAK1
hsa-miR-618	IL1RAP
hsa-miR-619-3p	IL1RN
hsa-miR-620	IL1RN
hsa-miR-625-5p	IRAK1
hsa-miR-628-5p	IL1R1
hsa-miR-629-3p	IL1RN
hsa-miR-632	IRAK1
hsa-miR-641	IRAK1
hsa-miR-6511a-3p	IL1RL2
hsa-miR-6511b-3p	IL1RL2
hsa-miR-670-3p	IRAK1
hsa-miR-6716-5p	IL36B
hsa-miR-6733-3p	IRAK1
hsa-miR-6784-3p	IL1RL2
hsa-miR-6832-5p	IL36B
hsa-miR-6862-3p	IL1RL2
hsa-miR-6868-5p	IL1RL1
hsa-miR-7153-5p	IRAK1
hsa-miR-744-3p	IRAK1
hsa-miR-744-5p	IRAK1

hsa-miR-767-3p	IL1RAP
hsa-miR-767-3p	IRAK1
hsa-miR-874-3p	IL1A
hsa-miR-874-3p	IL1RAP
hsa-miR-877-3p	IL1B
hsa-miR-887-3p	IL1B
hsa-miR-920	IL1B
hsa-miR-924	IL1RAP
hsa-miR-92a-1-5p	IL1A
hsa-miR-92a-1-5p	SIGIRR
hsa-miR-92a-3p	IRAK1
hsa-miR-93-3p	IL1RAP
hsa-miR-93-3p	IRAK1
hsa-miR-93-5p	IL1RAP
hsa-miR-96-3p	IL1R1
hsa-miR-96-5p	IL1A
hsa-mir-1226	IRAK1
hsa-mir-1226-3p	IRAK1
hsa-mir-1228	IL1RAP
hsa-mir-132	IRAK1
hsa-mir-142-3p	IRAK1
hsa-mir-149	SIGIRR
hsa-mir-16-1	IL36RN
hsa-mir-181a/181b/18	IL1A
hsa-mir-193b	IRAK1
hsa-mir-193b-3p	IRAK1
hsa-mir-222	IRAK1
hsa-mir-30a/30b/30c/	IL1A
hsa-mir-328	IRAK1
hsa-mir-4508	CARD14
hsa-mir-488-3p	IL1B
hsa-mir-744	IRAK1
hsa-mir-744-3p	IRAK1
hsa-mir-93	IRAK1
hsa-mir-93-3p	IRAK1
kshv-miR-K12-11-3p	IL1RAP
kshv-miR-K12-3-3p	IL1RAP
kshv-miR-K12-4-3p	IL1RAP
kshv-miR-K12-9-3p	IRAK1
hsa-miR-142-3p	IRAK1
hsa-miR-146a-5p	IRAK1
IL1B	ATF3

IL1B	CHI3L1
IL1B	CXCL8
IL1B	HNF4A
IL1B	IL10
TNF	IL1B
hsa-miR-31-5p	IL2
IL1B	IL6
IL2	SH3BP4
hsa-miR-101-3p	MALAT1
hsa-miR-101-3p	NEAT1
hsa-miR-101-3p	SOX9
hsa-miR-124-3p	SOX9
hsa-miR-30c-5p	SOX9
hsa-miR-30e-5p	SOX9
28S_RRNA	ATF3
28S_RRNA	CARM1
28S_RRNA	CHMP1B
28S_RRNA	CSDE1
28S_RRNA	KHSRP
28S_RRNA	NEAT1
28S_RRNA	POLR2A
28S_RRNA	RPL30
28S_RRNA	SRRM2
28S_RRNA	TBRG4
28S_RRNA	TIMP2
AC245014.3	FNBP4
AC245014.3	NEAT1
AC245014.3	RNU1-106P
AC245014.3	TENM1
AC245014.3	U1
ACO1	AR
ACO1	CISTR
ACO1	RAD51-AS1
ACO1	SENCR
AFAP1-AS1	ATF3
AFAP1-AS1	C17orf58
AFAP1-AS1	CDCP1
AFAP1-AS1	CXCL8
AFAP1-AS1	HNF4A
AFAP1-AS1	LTBP1
AFAP1-AS1	PLAUR
AFAP1-AS1	TFAP2A-AS2
AL353746.1	U1

ANCR	SOX9
ANCR	TIMP2
ANCR	TINCR
AR	AR
AR	ARHGEF10
AR	ARL6
AR	ATF3
AR	BEND4
AR	BNC1
AR	C17orf58
AR	CCDC124
AR	CCDC89
AR	CDCP1
AR	CHI3L1
AR	CHMP1B
AR	CLIP1
AR	CRNDE
AR	CSDE1
AR	CXCL8
AR	DEPDC1B
AR	DMXL2
AR	DRAIC
AR	EML2
AR	ERCC4
AR	FAM214B
AR	FNBP4
AR	FZR1
AR	GDF2
AR	GPR61
AR	GRB10
AR	GTF2F1
AR	GTF2H2
AR	HID1
AR	HNF4A
AR	HP1BP3
AR	IL10
AR	IL6
AR	INTS3
AR	KHSRP
AR	KRT6A
AR	L3MBTL2
AR	LTBP1
AR	MAGI1

AR	MALAT1
AR	MBD1
AR	MECP2
AR	MPPE1
AR	NARS2
AR	ND2
AR	ND5
AR	NDE1
AR	NDUFV3
AR	NEAT1
AR	NSRP1
AR	PCDH19
AR	PLAUR
AR	PLCG1
AR	PLEKHA1
AR	PLPP7
AR	POLR2A
AR	PPM1K
AR	PPP2R3A
AR	PRMT2
AR	PRPF31
AR	PRRG3
AR	PSD4
AR	PSMB5
AR	PTCH1
AR	RABAC1
AR	RBM28
AR	RBMS1
AR	RPL30
AR	SAMD4B
AR	SENP5
AR	SH3BP4
AR	SLC12A2
AR	SLC35C2
AR	SNHG16
AR	SOX9
AR	SRPRA
AR	SRRM2
AR	STS
AR	TBRG4
AR	TENM1
AR	TEX101
AR	TFAP2A-AS2

AR	TG
AR	TIMP2
AR	TINCR
AR	TMEM201
AR	TMTC2
AR	TNF
AR	TNFRSF14
AR	TNPO2
AR	TPPP
AR	TUG1
AR	USP38
AR	ZIC3
AR	ZMYND11
AR	ZNF318
AR	ZNF354B
AR	lincMTX2
ARHGEF10	CISTR
ARHGEF10	MALAT1
ARHGEF10	NORAD
ARHGEF10	RAD51-AS1
ARHGEF10	TINCR
ARHGEF10	TUG1
ARHGEF10	lnrCXCR4
ARL6	CRNDE
ARL6	lincMTX2
ARL6	lincTNS1
ATF3	CISTR
ATF3	CRNDE
ATF3	FENDRR
ATF3	MALAT1
ATF3	MIR17HG
ATF3	NORAD
ATF3	RAD51-AS1
ATF3	SNHG16
ATF3	hsa-miR-296-3p
ATF3	hsa-miR-33a-3p
ATP8	MALAT1
ATP8	ND1
ATP8	hsa-let-7g-5p
BANCR	CSDE1
BANCR	HID1
BANCR	NORAD
BANCR	RNA5-8SN3

BANCR	SOX9
BEND4	KHSRP
BNC1	CISTR
BNC1	CRNDE
BNC1	MALAT1
BNC1	lincTNS1
BNC1	lincZFP161
C17orf58	CISTR
C17orf58	FENDRR
C17orf58	TUG1
C5orf60	DRAIC
CARM1	FENDRR
CARM1	MALAT1
CARM1	MIR17HG
CARM1	SNHG16
CARM1	TFAP2A-AS2
CAT2	LTBP1
CAT2	MTND1P23
CAT2	RNU1-138P
CAT2	ZIC3
CCDC124	CRNDE
CCDC168	TFAP2A-AS2
CCDC89	NORAD
CCDC89	TUG1
CDCP1	CISTR
CDCP1	FENDRR
CDCP1	MALAT1
CDCP1	SBF2-AS1
CDCP1	SNHG16
CDCP1	TFAP2A-AS2
CDCP1	lincTNS1
CDCP1	lincZFP161
CHI3L1	SBF2-AS1
CHI3L1	lnrCXCR4
CHMP1B	FENDRR
CHMP1B	MALAT1
CHMP1B	TFAP2A-AS2
CHMP1B	hsa-miR-425-5p
CHMP1B	hsa-mir-181a/181b/18
CHMP1B	lincMTX2
CHMP3	RAD51-AS1
CHMP3	SNHG16

CHMP3	TINCR
CISTR	CSDE1
CISTR	DMXL2
CISTR	FAM214B
CISTR	FNBP4
CISTR	FZR1
CISTR	GTF2F1
CISTR	KHSRP
CISTR	MPPE1
CISTR	NDE1
CISTR	PLCG1
CISTR	PLEKHA1
CISTR	PRMT2
CISTR	PSMB5
CISTR	PTCH1
CISTR	SLC35C2
CISTR	SOX9
CISTR	SRPRA
CISTR	SRRM2
CISTR	SSH1
CISTR	TIMP2
CISTR	TNPO2
CLIP1	NORAD
CRNDE	CRNDE
CRNDE	EML2
CRNDE	FAM214B
CRNDE	GRB10
CRNDE	HP1BP3
CRNDE	IL6
CRNDE	L3MBTL2
CRNDE	NARS2
CRNDE	NDE1
CRNDE	NEAT1
CRNDE	PLAUR
CRNDE	PLCG1
CRNDE	PPP2R3A
CRNDE	PRPF31
CRNDE	PSMB5
CRNDE	RBM28
CRNDE	SLC35C2
CRNDE	SNHG16
CRNDE	TBRG4
CRNDE	TMTC2

CRNDE	USP38
CSDE1	FENDRR
CSDE1	MIR17HG
CSDE1	NSRP1
CSDE1	RNA5-8SN3
CSDE1	SNHG16
CSDE1	hsa-miR-144-3p
CSDE1	hsa-miR-16-1-3p
CSDE1	hsa-miR-330-3p
CSDE1	hsa-miR-371a-3p
CSDE1	hsa-miR-378a-5p
CSDE1	hsa-miR-422a
CSDE1	hsa-miR-425-5p
CSDE1	hsa-miR-431-5p
CSDE1	hsa-miR-503-3p
CSDE1	lincTNS1
CSDE1	lincZFP161
CXCL8	IL1beta-RBT46
CXCL8	IL1beta-eRNA
CXCL8	MALAT1
CXCL8	NEAT1
CXCL8	NRCP
CXCL8	RAD51-AS1
CXCL8	SENCR
CXCL8	SNHG16
CXCL8	lincTNS1
DEPDC1B	MALAT1
DEPDC1B	SBF2-AS1
DEPDC1B	TUG1
DEPDC1B	lincMTX2
DMXL2	NEAT1
DMXL2	hsa-miR-132-3p
DMXL2	hsa-miR-135b-5p
DMXL2	hsa-miR-141-3p
DMXL2	hsa-miR-149-3p
DMXL2	hsa-miR-30b-3p
DMXL2	hsa-miR-365a-3p
DMXL2	hsa-mir-181a/181b/18
DMXL2	hsa-mir-30a/30b/30c/
DRAIC	PLPP7
DRAIC	SNORA73B
DRAIC	TNFRSF14

EML2	SBF2-AS1
EML2	TFAP2A-AS2
EML2	TUG1
EML2	lincMTX2
ERCC4	TFAP2A-AS2
ERCC4	lincTNS1
FAM214B	RAD51-AS1
FAM214B	TFAP2A-AS2
FAM214B	lincMTX2
FENDRR	FENDRR
FENDRR	L3MBTL2
FENDRR	MALAT1
FENDRR	MPPE1
FENDRR	MROH1
FENDRR	NARS2
FENDRR	NORAD
FENDRR	NSRP1
FENDRR	PPM1K
FENDRR	PPP2R3A
FENDRR	PRPF31
FENDRR	PSMB5
FENDRR	SNHG16
FENDRR	SRRM2
FENDRR	TBRG4
FENDRR	TIMP2
FENDRR	USP38
FENDRR	ZMYND11
FENDRR	lincMTX2
FNBP4	MALAT1
FNBP4	TINCR
FNBP4	hsa-miR-23c
FZR1	TNPO2
GDF2	RNA5-8SN3
GDF2	U1
GPR61	lnrCXCR4
GRB10	MALAT1
GRB10	NORAD
GRB10	SBF2-AS1
GRB10	TUG1
GRB10	hsa-miR-182-3p
GRB10	hsa-miR-328
GRB10	hsa-miR-96-5p
GRB10	lincMTX2

GTF2F1	MALAT1
GTF2F1	NSRP1
GTF2F1	TINCR
HID1	TFAP2A-AS2
HNF4A	NORAD
HNF4A	TNPO2
HNF4A	lnrCXCR4
HP1BP3	U1
HP1BP3	lincMTX2
IL10	NORAD
IL10	TFAP2A-AS2
IL10	hsa-miR-328
IL10	lnrCXCR4
IL6	MALAT1
IL6	NORAD
IL6	RAD51-AS1
IL6	SBF2-AS1
IL6	SENCR
INTS3	MIR17HG
INTS3	RAD51-AS1
INTS3	RNU1-106P
INTS3	RNVU1-32
KHSRP	MIR17HG
KHSRP	TFAP2A-AS2
KHSRP	TINCR
KHSRP	TUG1
KHSRP	hsa-miR-378a-5p
KHSRP	hsa-miR-422a
KHSRP	lincTNS1
KRT6A	ND2
KRT6A	RNR2
KRT6A	TINCR
KRT6A	TUG1
L3MBTL2	NDE1
L3MBTL2	SENCR
L3MBTL2	lincTNS1
LTBP1	MIR17HG
LTBP1	RAD51-AS1
LTBP1	TFAP2A-AS2
LTBP1	TINCR
MAGI1	TFAP2A-AS2
MAGI1	TINCR
MAGI1	TNPO2

MALAT1	MALAT1
MALAT1	ND1
MALAT1	ND2
MALAT1	ND4
MALAT1	ND5
MALAT1	NDE1
MALAT1	NEAT1
MALAT1	PLAUR
MALAT1	PPM1K
MALAT1	PRMT2
MALAT1	RNA5-8SN3
MALAT1	RNR2
MALAT1	RNU1-106P
MALAT1	RNU1-138P
MALAT1	RNVU1-32
MALAT1	SSH1
MALAT1	TEX101
MALAT1	TMTC2
MALAT1	TNPO2
MALAT1	USP38
MALAT1	ZNF354B
MALAT1	hsa-let-7b-5p
MALAT1	hsa-let-7d-3p
MALAT1	hsa-let-7f-5p
MALAT1	hsa-let-7g-5p
MALAT1	hsa-let-7i-5p
MALAT1	hsa-miR-106a-3p
MALAT1	hsa-miR-1249-3p
MALAT1	hsa-miR-132-3p
MALAT1	hsa-miR-134-5p
MALAT1	hsa-miR-135b-5p
MALAT1	hsa-miR-146a-3p
MALAT1	hsa-miR-149-3p
MALAT1	hsa-miR-16-1-3p
MALAT1	hsa-miR-182-3p
MALAT1	hsa-miR-18b-5p
MALAT1	hsa-miR-215-5p
MALAT1	hsa-miR-221-3p
MALAT1	hsa-miR-23a-3p
MALAT1	hsa-miR-296-3p
MALAT1	hsa-miR-30b-3p
MALAT1	hsa-miR-30b-5p
MALAT1	hsa-miR-3118

MALAT1	hsa-miR-326
MALAT1	hsa-miR-328
MALAT1	hsa-miR-339-5p
MALAT1	hsa-miR-33a-3p
MALAT1	hsa-miR-346
MALAT1	hsa-miR-365a-3p
MALAT1	hsa-miR-370
MALAT1	hsa-miR-371a-3p
MALAT1	hsa-miR-373-3p
MALAT1	hsa-miR-376c-3p
MALAT1	hsa-miR-378a-5p
MALAT1	hsa-miR-381-3p
MALAT1	hsa-miR-382-5p
MALAT1	hsa-miR-422a
MALAT1	hsa-miR-425-5p
MALAT1	hsa-miR-4262
MALAT1	hsa-miR-4295
MALAT1	hsa-miR-449a
MALAT1	hsa-miR-452-3p
MALAT1	hsa-miR-490-3p
MALAT1	hsa-miR-495-3p
MALAT1	hsa-miR-503-3p
MALAT1	hsa-miR-515-3p
MALAT1	hsa-miR-526b-3p
MALAT1	hsa-miR-542-3p
MALAT1	hsa-miR-543
MALAT1	hsa-miR-548c-3p
MALAT1	hsa-miR-590-3p
MALAT1	hsa-miR-6893-3p
MALAT1	hsa-miR-7153-5p
MALAT1	hsa-miR-877-3p
MALAT1	hsa-mir-181a/181b/18
MALAT1	hsa-mir-222
MALAT1	hsa-mir-30a/30b/30c/
MALAT1	hsa-mir-488-3p
MALAT1	lincMTX2
MBD1	RAD51-AS1
MBD1	hsa-miR-149-3p
MBD1	hsa-miR-16-1-3p
MBD1	hsa-miR-182-3p
MBD1	hsa-miR-328
MBD1	hsa-miR-503-3p

MBD1	hsa-miR-96-5p
MECP2	MIR17HG
MECP2	SNHG16
MIR17HG	ND2
MIR17HG	ND4
MIR17HG	NDUFV3
MIR17HG	PLCG1
MIR17HG	POLR2A
MIR17HG	PRMT2
MIR17HG	RABAC1
MIR17HG	RPL30
MIR17HG	SRPRA
MIR17HG	SRRM2
MIR17HG	USP38
MPPE1	TFAP2A-AS2
MROH1	TFAP2A-AS2
MROH1	lincTNS1
MTND4P12	ND4
MTND4P12	NEAT1
MTND4P12	ZNF318
MTND4P12	hsa-let-7d-3p
MTND4P12	hsa-miR-16-1-3p
MTND4P12	hsa-miR-373-3p
NARS2	RAD51-AS1
NARS2	TNPO2
NARS2	lincTNS1
NARS2	lincZFP161
ND1	ND4
ND1	RNR2
ND1	RNVU1-7
ND1	TEX101
ND2	ND4
ND2	ND5
ND2	NEAT1
ND2	NORAD
ND2	RNR2
ND2	TEX101
ND4	ND5
ND4	NEAT1
ND4	NORAD
ND4	RAD51-AS1
ND4	RNR2
ND4	ZNF318

ND5	NEAT1
ND5	NORAD
ND5	NSRP1
ND5	RAD51-AS1
ND5	RNR2
ND5	SRRM2
ND5	STS
NDE1	NSRP1
NDE1	POLR2A
NDE1	PRPF31
NDE1	SRPRA
NDE1	TINCR
NDE1	TUG1
NDE1	lincMTX2
NDE1	lincTNS1
NDUFV3	NORAD
NDUFV3	RNR2
NEAT1	NORAD
NEAT1	RNA5-8SN3
NEAT1	RNR2
NEAT1	RNU1-106P
NEAT1	RNU1-138P
NEAT1	RNU1-148P
NEAT1	RNVU1-32
NEAT1	RNVU1-7
NEAT1	SENP5
NEAT1	TINCR
NEAT1	hsa-let-7d-3p
NEAT1	hsa-miR-106a-3p
NEAT1	hsa-miR-1249-3p
NEAT1	hsa-miR-125b-5p
NEAT1	hsa-miR-130b-3p
NEAT1	hsa-miR-132-3p
NEAT1	hsa-miR-134-5p
NEAT1	hsa-miR-135b-5p
NEAT1	hsa-miR-141-3p
NEAT1	hsa-miR-143-3p
NEAT1	hsa-miR-144-3p
NEAT1	hsa-miR-146a-3p
NEAT1	hsa-miR-146b-5p
NEAT1	hsa-miR-149-3p
NEAT1	hsa-miR-16-1-3p
NEAT1	hsa-miR-182-3p

NEAT1	hsa-miR-184
NEAT1	hsa-miR-18b-5p
NEAT1	hsa-miR-205-5p
NEAT1	hsa-miR-221-3p
NEAT1	hsa-miR-23c
NEAT1	hsa-miR-296-3p
NEAT1	hsa-miR-30b-3p
NEAT1	hsa-miR-3118
NEAT1	hsa-miR-326
NEAT1	hsa-miR-328
NEAT1	hsa-miR-330-3p
NEAT1	hsa-miR-33a-3p
NEAT1	hsa-miR-346
NEAT1	hsa-miR-3666
NEAT1	hsa-miR-370
NEAT1	hsa-miR-371a-3p
NEAT1	hsa-miR-373-3p
NEAT1	hsa-miR-376c-3p
NEAT1	hsa-miR-378a-5p
NEAT1	hsa-miR-381-3p
NEAT1	hsa-miR-382-5p
NEAT1	hsa-miR-422a
NEAT1	hsa-miR-425-5p
NEAT1	hsa-miR-4262
NEAT1	hsa-miR-4295
NEAT1	hsa-miR-431-5p
NEAT1	hsa-miR-452-3p
NEAT1	hsa-miR-454-3p
NEAT1	hsa-miR-490-3p
NEAT1	hsa-miR-495-3p
NEAT1	hsa-miR-503-3p
NEAT1	hsa-miR-526b-3p
NEAT1	hsa-miR-542-3p
NEAT1	hsa-miR-543
NEAT1	hsa-miR-590-3p
NEAT1	hsa-miR-6893-3p
NEAT1	hsa-miR-7153-5p
NEAT1	hsa-miR-877-3p
NEAT1	hsa-miR-96-5p
NEAT1	hsa-mir-181a/181b/18
NEAT1	hsa-mir-222
NEAT1	hsa-mir-30a/30b/30c/

NEAT1	hsa-mir-488-3p
NEAT1	lincMTX2
NME1-NME2	NSRP1
NME1-NME2	RAD51-AS1
NME1-NME2	SNHG16
NME1-NME2	hsa-miR-149-3p
NORAD	NORAD
NORAD	OR8A1
NORAD	POLR2A
NORAD	RNR2
NORAD	SOX9
NORAD	SRRM2
NORAD	TFAP2A-AS2
NORAD	TINCR
NORAD	TNF
NRCP	PLEKHA1
NRCP	SLC12A2
NRCP	TNF
NSRP1	PSMB5
NSRP1	RABAC1
NSRP1	SAMD4B
NSRP1	SRPRA
NSRP1	lincTNS1
PCDH19	TFAP2A-AS2
PLAUR	TUG1
PLAUR	lincMTX2
PLCG1	RAD51-AS1
PLCG1	RNA5-8SN3
PLCG1	TFAP2A-AS2
PLCG1	lincMTX2
PLEKHA1	SNHG16
PLEKHA1	TFAP2A-AS2
PLEKHA1	lincMTX2
PLPP7	lincMTX2
POLR2A	RNU1-106P
POLR2A	RNVU1-32
POLR2A	TIMP2
POLR2A	TINCR
PPM1K	TFAP2A-AS2
PPM1K	TUG1
PPM1K	lincTNS1
PPM1K	lincZFP161
PPP2R3A	SNHG16

PPP2R3A	TINCR
PRMT2	RAD51-AS1
PRMT2	RNA5-8SN3
PRPF31	TFAP2A-AS2
PRPF31	TUG1
PSD4	RNA5-8SN3
PSMB5	RAD51-AS1
PSMB5	SNHG16
PTCH1	SNHG16
PTCH1	TFAP2A-AS2
PTCH1	hsa-miR-125b-5p
PTCH1	hsa-miR-134-5p
PTCH1	hsa-miR-3118
PTCH1	hsa-miR-370
PTCH1	hsa-miR-490-3p
PTCH1	hsa-miR-6893-3p
PTCH1	lnrCXCR4
RABAC1	TFAP2A-AS2
RAD51-AS1	RNU4-2
RAD51-AS1	RNVU1-7
RAD51-AS1	SH3BP4
RAD51-AS1	SRPRA
RAD51-AS1	TIMP2
RAD51-AS1	TNF
RAD51-AS1	ZNF354B
RAD51-AS1	lnrCXCR4
RBM28	lincMTX2
RBMS1	hsa-miR-143-3p
RBMS1	hsa-miR-18b-5p
RBMS1	hsa-miR-490-3p
RBMS1	hsa-mir-488-3p
RBMS1	lincTNS1
RBMS1	lincZFP161
RN7SKP119	TIMP2
RNA5-8SN3	AC091045.1
RNA5-8SN3	RNA5-8SP6
RNA5-8SN3	STS
RNA5-8SN3	TEX101
RNA5-8SN3	TNPO2
RNA5-8SN3	TPPP
RNA5-8SP6	SSH1
RNA5-8SP6	STS
RNA5SP298	TEX101

RNR2	AC091045.1
RNR2	RNU1-106P
RNR2	SAMD4B
RNR2	TEX101
RNR2	TNPO2
RNU1-106P	RNU1-148P
RNU1-106P	RNVU1-7
RNU1-106P	SRRM2
RNU1-106P	TPPP
RNU1-138P	SENCR
RNU1-148P	RNVU1-32
RNU1-148P	U1
RNU4-2	U1
RNVU1-32	RNVU1-7
RNVU1-7	TEX101
RNVU1-7	TNPO2
RNVU1-7	U1
RPL30	TFAP2A-AS2
RPL30	hsa-miR-1197
RPL30	hsa-miR-1249-3p
RPL30	hsa-miR-134-5p
RPL30	hsa-miR-149-3p
RPL30	hsa-miR-16-1-3p
RPL30	hsa-miR-184
RPL30	hsa-miR-205-5p
RPL30	hsa-miR-23a-3p
RPL30	hsa-miR-23c
RPL30	hsa-miR-3118
RPL30	hsa-miR-326
RPL30	hsa-miR-328
RPL30	hsa-miR-330-3p
RPL30	hsa-miR-365a-3p
RPL30	hsa-miR-371a-3p
RPL30	hsa-miR-376c-3p
RPL30	hsa-miR-382-5p
RPL30	hsa-miR-449a
RPL30	hsa-miR-495-3p
RPL30	hsa-miR-503-3p
RPL30	hsa-miR-542-3p
SAMD4B	SBF2-AS1
SAMD4B	SNHG16
SAMD4B	hsa-miR-376c-3p
SBF2-AS1	SLC12A2

SBF2-AS1	TIMP2
SBF2-AS1	hsa-miR-134-5p
SBF2-AS1	hsa-miR-141-3p
SBF2-AS1	hsa-miR-16-1-3p
SBF2-AS1	hsa-miR-3118
SBF2-AS1	hsa-miR-330-3p
SBF2-AS1	hsa-miR-339-5p
SBF2-AS1	hsa-miR-33a-3p
SBF2-AS1	hsa-miR-495-3p
SBF2-AS1	hsa-miR-503-3p
SBF2-AS1	hsa-mir-181a/181b/18
SBF2-AS1	lincMTX2
SENP5	SNHG16
SENP5	TFAP2A-AS2
SENP5	hsa-let-7d-3p
SENP5	hsa-miR-328
SH3BP4	SNHG16
SH3BP4	lincZFP161
SLC12A2	SNHG16
SLC35C2	SNHG16
SLC35C2	hsa-miR-134-5p
SLC35C2	hsa-miR-296-3p
SLC35C2	hsa-miR-3118
SLC35C2	hsa-miR-326
SLC35C2	hsa-miR-330-3p
SLC35C2	hsa-miR-370
SLC35C2	hsa-miR-381-3p
SLC35C2	hsa-miR-613
SLC35C2	hsa-miR-877-3p
SLC35C2	lincMTX2
SNHG16	SSH1
SNHG16	STS
SNHG16	TINCR
SNHG16	TMEM201
SNHG16	TNPO2
SNHG16	TUG1
SNHG16	USP38
SNHG16	ZMYND11
SNHG16	ZNF354B
SNHG16	hsa-miR-106a-3p
SNHG16	hsa-miR-146a-3p
SNHG16	hsa-miR-182-3p

SNHG16	hsa-miR-23a-3p
SNHG16	hsa-miR-23c
SNHG16	hsa-miR-30b-3p
SNHG16	hsa-miR-382-5p
SNHG16	hsa-miR-526b-3p
SNHG16	hsa-miR-7153-5p
SNHG16	hsa-miR-96-5p
SNHG16	lincMTX2
SNHG16	lincTNS1
SNORA73B	TFAP2A-AS2
SNORA73B	hsa-miR-215-5p
SNORA73B	hsa-miR-330-3p
SOX9	TUG1
SRPRA	TINCR
SRPRA	lincMTX2
SRRM2	RNVU1-32
SRRM2	SSH1
SRRM2	TINCR
SRRM2	hsa-miR-130b-3p
SRRM2	hsa-miR-141-3p
SRRM2	hsa-miR-146a-3p
SRRM2	hsa-miR-146b-5p
SRRM2	hsa-miR-18b-5p
SRRM2	hsa-miR-205-5p
SRRM2	hsa-miR-296-3p
SRRM2	hsa-miR-326
SRRM2	hsa-miR-328
SRRM2	hsa-miR-330-3p
SRRM2	hsa-miR-339-5p
SRRM2	hsa-miR-3666
SRRM2	hsa-miR-370
SRRM2	hsa-miR-371a-3p
SRRM2	hsa-miR-373-3p
SRRM2	hsa-miR-381-3p
SRRM2	hsa-miR-422a
SRRM2	hsa-miR-4295
SRRM2	hsa-miR-449a
SRRM2	hsa-miR-452-3p
SRRM2	hsa-miR-454-3p
SRRM2	hsa-miR-526b-3p
SRRM2	hsa-miR-7153-5p
SRRM2	hsa-miR-877-3p
SRRM2	hsa-mir-488-3p

STS	TEX101
STS	TFAP2A-AS2
TENM1	TNPO2
TENM1	lnrCXCR4
TEX101	TFAP2A-AS2
TEX101	TPPP
TEX101	U1
TEX101	lnrCXCR4
TFAP2A-AS2	ZIC3
TFAP2A-AS2	ZNF354B
TIMP2	TINCR
TIMP2	TNPO2
TINCR	TINCR
TINCR	TNPO2
TINCR	ZNF318
TMTC2	lincMTX2
TNFRSF14	lnrCXCR4
TNPO2	U1
TNPO2	lincMTX2
TPPP	RNVU1-32
TUG1	TUG1
TUG1	USP38
TUG1	ZMYND11
TUG1	hsa-let-7b-5p
TUG1	hsa-let-7d-3p
TUG1	hsa-let-7g-5p
TUG1	hsa-let-7i-5p
TUG1	hsa-miR-106a-3p
TUG1	hsa-miR-130b-3p
TUG1	hsa-miR-135b-5p
TUG1	hsa-miR-141-3p
TUG1	hsa-miR-149-3p
TUG1	hsa-miR-16-1-3p
TUG1	hsa-miR-18b-5p
TUG1	hsa-miR-205-5p
TUG1	hsa-miR-215-5p
TUG1	hsa-miR-23a-3p
TUG1	hsa-miR-23c
TUG1	hsa-miR-328
TUG1	hsa-miR-330-3p
TUG1	hsa-miR-33a-3p
TUG1	hsa-miR-365a-3p
TUG1	hsa-miR-3666

TUG1	hsa-miR-370
TUG1	hsa-miR-371a-3p
TUG1	hsa-miR-425-5p
TUG1	hsa-miR-4262
TUG1	hsa-miR-4295
TUG1	hsa-miR-452-3p
TUG1	hsa-miR-454-3p
TUG1	hsa-miR-503-3p
TUG1	hsa-miR-526b-3p
TUG1	hsa-miR-542-3p
TUG1	hsa-miR-543
TUG1	hsa-miR-613
TUG1	hsa-miR-877-3p
TUG1	hsa-mir-181a/181b/18
TUG1	hsa-mir-222
TUG1	hsa-mir-488-3p
TUG1	lincTNS1
U1	ZNF318
U1	ZNF354B
USP38	lincMTX2
USP38	lincTNS1
hsa-let-7a-5p	AC053481.3
hsa-let-7a-5p	BEND4
hsa-let-7a-5p	CDCP1
hsa-let-7a-5p	CLIP1
hsa-let-7a-5p	CSDE1
hsa-let-7a-5p	CXCL8
hsa-let-7a-5p	DMXL2
hsa-let-7a-5p	FAM214B
hsa-let-7a-5p	FZR1
hsa-let-7a-5p	GRB10
hsa-let-7a-5p	IL10
hsa-let-7a-5p	IL6
hsa-let-7a-5p	LTBP1
hsa-let-7a-5p	MBD1
hsa-let-7a-5p	MECP2
hsa-let-7a-5p	MTND4P12
hsa-let-7a-5p	NARS2
hsa-let-7a-5p	ND1
hsa-let-7a-5p	ND2
hsa-let-7a-5p	ND5
hsa-let-7a-5p	PCDH19

hsa-let-7a-5p	PLAUR
hsa-let-7a-5p	POLR2A
hsa-let-7a-5p	PRPF31
hsa-let-7a-5p	PTCH1
hsa-let-7a-5p	RBM28
hsa-let-7a-5p	RBMS1
hsa-let-7a-5p	RPL30
hsa-let-7a-5p	SAMD4B
hsa-let-7a-5p	SENP5
hsa-let-7a-5p	SLC12A2
hsa-let-7a-5p	SLC35C2
hsa-let-7a-5p	SNHG16
hsa-let-7a-5p	SSH1
hsa-let-7a-5p	TINCR
hsa-let-7a-5p	TNF
hsa-let-7a-5p	TNPO2
hsa-let-7a-5p	USP38
hsa-let-7a-5p	ZMYND11
hsa-let-7a-5p	ZNF318
hsa-let-7b-5p	AC053481.3
hsa-let-7b-5p	AR
hsa-let-7b-5p	BEND4
hsa-let-7b-5p	CDCP1
hsa-let-7b-5p	CLIP1
hsa-let-7b-5p	CSDE1
hsa-let-7b-5p	CXCL8
hsa-let-7b-5p	FAM214B
hsa-let-7b-5p	FZR1
hsa-let-7b-5p	GRB10
hsa-let-7b-5p	HNF4A
hsa-let-7b-5p	IL10
hsa-let-7b-5p	KHSRP
hsa-let-7b-5p	LTBP1
hsa-let-7b-5p	MBD1
hsa-let-7b-5p	MECP2
hsa-let-7b-5p	MTND4P12
hsa-let-7b-5p	NARS2
hsa-let-7b-5p	ND1
hsa-let-7b-5p	ND2
hsa-let-7b-5p	ND4
hsa-let-7b-5p	ND5
hsa-let-7b-5p	NEAT1
hsa-let-7b-5p	PCDH19

hsa-let-7b-5p	PLAUR
hsa-let-7b-5p	PLEKHA1
hsa-let-7b-5p	POLR2A
hsa-let-7b-5p	PRPF31
hsa-let-7b-5p	PTCH1
hsa-let-7b-5p	RBM28
hsa-let-7b-5p	RBMS1
hsa-let-7b-5p	SAMD4B
hsa-let-7b-5p	SENP5
hsa-let-7b-5p	SLC12A2
hsa-let-7b-5p	SLC35C2
hsa-let-7b-5p	SNHG16
hsa-let-7b-5p	SOX9
hsa-let-7b-5p	SSH1
hsa-let-7b-5p	TINCR
hsa-let-7b-5p	TMEM201
hsa-let-7b-5p	TNF
hsa-let-7b-5p	TNPO2
hsa-let-7b-5p	USP38
hsa-let-7b-5p	ZMYND11
hsa-let-7b-5p	ZNF318
hsa-let-7b-5p	ZNF354B
hsa-let-7c-5p	AC053481.3
hsa-let-7c-5p	AR
hsa-let-7c-5p	BEND4
hsa-let-7c-5p	CARM1
hsa-let-7c-5p	CDCP1
hsa-let-7c-5p	CLIP1
hsa-let-7c-5p	CSDE1
hsa-let-7c-5p	CXCL8
hsa-let-7c-5p	DMXL2
hsa-let-7c-5p	FAM214B
hsa-let-7c-5p	FZR1
hsa-let-7c-5p	GRB10
hsa-let-7c-5p	IL6
hsa-let-7c-5p	LTBP1
hsa-let-7c-5p	MBD1
hsa-let-7c-5p	MECP2
hsa-let-7c-5p	MTND4P12
hsa-let-7c-5p	NARS2
hsa-let-7c-5p	ND1
hsa-let-7c-5p	ND2
hsa-let-7c-5p	ND4

hsa-let-7c-5p	ND5
hsa-let-7c-5p	NEAT1
hsa-let-7c-5p	PCDH19
hsa-let-7c-5p	PLAUR
hsa-let-7c-5p	POLR2A
hsa-let-7c-5p	PPM1K
hsa-let-7c-5p	PRPF31
hsa-let-7c-5p	PTCH1
hsa-let-7c-5p	RBM28
hsa-let-7c-5p	RBMS1
hsa-let-7c-5p	RNR2
hsa-let-7c-5p	SAMD4B
hsa-let-7c-5p	SENP5
hsa-let-7c-5p	SLC12A2
hsa-let-7c-5p	SLC35C2
hsa-let-7c-5p	SNHG16
hsa-let-7c-5p	SSH1
hsa-let-7c-5p	TINCR
hsa-let-7c-5p	TMEM201
hsa-let-7c-5p	TNF
hsa-let-7c-5p	TNPO2
hsa-let-7c-5p	USP38
hsa-let-7c-5p	ZMYND11
hsa-let-7c-5p	ZNF318
hsa-let-7c-5p	ZNF354B
hsa-let-7d-3p	CARM1
hsa-let-7d-3p	CSDE1
hsa-let-7d-3p	MAGI1
hsa-let-7d-3p	PCDH19
hsa-let-7d-3p	PPM1K
hsa-let-7d-3p	RBMS1
hsa-let-7d-3p	SNHG16
hsa-let-7d-5p	AC053481.3
hsa-let-7d-5p	BEND4
hsa-let-7d-5p	CDCP1
hsa-let-7d-5p	CSDE1
hsa-let-7d-5p	CXCL8
hsa-let-7d-5p	DMXL2
hsa-let-7d-5p	FAM214B
hsa-let-7d-5p	FNBP4
hsa-let-7d-5p	FZR1
hsa-let-7d-5p	GRB10
hsa-let-7d-5p	HNF4A

hsa-let-7d-5p	IL10
hsa-let-7d-5p	IL6
hsa-let-7d-5p	LTBP1
hsa-let-7d-5p	MECP2
hsa-let-7d-5p	MTND4P12
hsa-let-7d-5p	NARS2
hsa-let-7d-5p	NEAT1
hsa-let-7d-5p	PCDH19
hsa-let-7d-5p	PLAUR
hsa-let-7d-5p	POLR2A
hsa-let-7d-5p	PRPF31
hsa-let-7d-5p	PTCH1
hsa-let-7d-5p	RBM28
hsa-let-7d-5p	RBMS1
hsa-let-7d-5p	SENP5
hsa-let-7d-5p	SLC12A2
hsa-let-7d-5p	SLC35C2
hsa-let-7d-5p	SNHG16
hsa-let-7d-5p	SOX9
hsa-let-7d-5p	SSH1
hsa-let-7d-5p	STS
hsa-let-7d-5p	TMEM201
hsa-let-7d-5p	TNF
hsa-let-7d-5p	TNPO2
hsa-let-7d-5p	USP38
hsa-let-7d-5p	ZNF318
hsa-let-7d-5p	ZNF354B
hsa-let-7e-5p	AC053481.3
hsa-let-7e-5p	ACO1
hsa-let-7e-5p	BEND4
hsa-let-7e-5p	CARM1
hsa-let-7e-5p	CDCP1
hsa-let-7e-5p	CLIP1
hsa-let-7e-5p	CSDE1
hsa-let-7e-5p	CXCL8
hsa-let-7e-5p	DMXL2
hsa-let-7e-5p	ERCC4
hsa-let-7e-5p	FAM214B
hsa-let-7e-5p	FZR1
hsa-let-7e-5p	GRB10
hsa-let-7e-5p	HNF4A
hsa-let-7e-5p	IL10
hsa-let-7e-5p	IL6

hsa-let-7e-5p	LTBP1
hsa-let-7e-5p	MBD1
hsa-let-7e-5p	MECP2
hsa-let-7e-5p	MTND4P12
hsa-let-7e-5p	NARS2
hsa-let-7e-5p	ND1
hsa-let-7e-5p	ND2
hsa-let-7e-5p	ND4
hsa-let-7e-5p	ND5
hsa-let-7e-5p	NEAT1
hsa-let-7e-5p	PCDH19
hsa-let-7e-5p	PLAUR
hsa-let-7e-5p	POLR2A
hsa-let-7e-5p	PRPF31
hsa-let-7e-5p	PTCH1
hsa-let-7e-5p	RBM28
hsa-let-7e-5p	RBMS1
hsa-let-7e-5p	SAMD4B
hsa-let-7e-5p	SENP5
hsa-let-7e-5p	SLC35C2
hsa-let-7e-5p	SNHG16
hsa-let-7e-5p	SSH1
hsa-let-7e-5p	TINCR
hsa-let-7e-5p	TNF
hsa-let-7e-5p	TNPO2
hsa-let-7e-5p	USP38
hsa-let-7e-5p	ZNF318
hsa-let-7e-5p	ZNF354B
hsa-let-7f-2-3p	BEND4
hsa-let-7f-2-3p	CSDE1
hsa-let-7f-2-3p	DEPDC1B
hsa-let-7f-2-3p	FNBP4
hsa-let-7f-2-3p	IL10
hsa-let-7f-2-3p	MECP2
hsa-let-7f-2-3p	PTCH1
hsa-let-7f-2-3p	SENP5
hsa-let-7f-2-3p	SNHG16
hsa-let-7f-2-3p	TNF
hsa-let-7f-2-3p	USP38
hsa-let-7f-2-3p	ZNF318
hsa-let-7f-5p	AC053481.3
hsa-let-7f-5p	BEND4
hsa-let-7f-5p	CDCP1

hsa-let-7f-5p	CSDE1
hsa-let-7f-5p	CXCL8
hsa-let-7f-5p	DMXL2
hsa-let-7f-5p	ERCC4
hsa-let-7f-5p	FAM214B
hsa-let-7f-5p	FZR1
hsa-let-7f-5p	GRB10
hsa-let-7f-5p	IL10
hsa-let-7f-5p	IL6
hsa-let-7f-5p	LTBP1
hsa-let-7f-5p	MBD1
hsa-let-7f-5p	MECP2
hsa-let-7f-5p	MTND4P12
hsa-let-7f-5p	NARS2
hsa-let-7f-5p	ND2
hsa-let-7f-5p	ND4
hsa-let-7f-5p	ND5
hsa-let-7f-5p	NEAT1
hsa-let-7f-5p	PCDH19
hsa-let-7f-5p	PLAUR
hsa-let-7f-5p	PLEKHA1
hsa-let-7f-5p	PRPF31
hsa-let-7f-5p	PTCH1
hsa-let-7f-5p	RBM28
hsa-let-7f-5p	RBMS1
hsa-let-7f-5p	SAMD4B
hsa-let-7f-5p	SENP5
hsa-let-7f-5p	SLC12A2
hsa-let-7f-5p	SNHG16
hsa-let-7f-5p	SSH1
hsa-let-7f-5p	TG
hsa-let-7f-5p	TINCR
hsa-let-7f-5p	TMTC2
hsa-let-7f-5p	TNF
hsa-let-7f-5p	TNPO2
hsa-let-7f-5p	TUG1
hsa-let-7f-5p	USP38
hsa-let-7f-5p	ZNF318
hsa-let-7f-5p	ZNF354B
hsa-let-7g-5p	AC053481.3
hsa-let-7g-5p	BEND4
hsa-let-7g-5p	CLIP1
hsa-let-7g-5p	CSDE1

hsa-let-7g-5p	CXCL8
hsa-let-7g-5p	DMXL2
hsa-let-7g-5p	ERCC4
hsa-let-7g-5p	FAM214B
hsa-let-7g-5p	FZR1
hsa-let-7g-5p	GRB10
hsa-let-7g-5p	HNF4A
hsa-let-7g-5p	IL10
hsa-let-7g-5p	IL6
hsa-let-7g-5p	LTBP1
hsa-let-7g-5p	MBD1
hsa-let-7g-5p	MECP2
hsa-let-7g-5p	MTND4P12
hsa-let-7g-5p	NARS2
hsa-let-7g-5p	ND2
hsa-let-7g-5p	NEAT1
hsa-let-7g-5p	PCDH19
hsa-let-7g-5p	PLAUR
hsa-let-7g-5p	PLEKHA1
hsa-let-7g-5p	POLR2A
hsa-let-7g-5p	PRPF31
hsa-let-7g-5p	PTCH1
hsa-let-7g-5p	RBM28
hsa-let-7g-5p	RBMS1
hsa-let-7g-5p	SAMD4B
hsa-let-7g-5p	SENP5
hsa-let-7g-5p	SLC12A2
hsa-let-7g-5p	SNHG16
hsa-let-7g-5p	SSH1
hsa-let-7g-5p	TINCR
hsa-let-7g-5p	TMEM201
hsa-let-7g-5p	TMTC2
hsa-let-7g-5p	TNF
hsa-let-7g-5p	USP38
hsa-let-7g-5p	ZNF318
hsa-let-7g-5p	ZNF354B
hsa-let-7i-5p	AC053481.3
hsa-let-7i-5p	AR
hsa-let-7i-5p	BEND4
hsa-let-7i-5p	CDCP1
hsa-let-7i-5p	CLIP1
hsa-let-7i-5p	CSDE1
hsa-let-7i-5p	CXCL8

hsa-let-7i-5p	DEPDC1B
hsa-let-7i-5p	ERCC4
hsa-let-7i-5p	FAM214B
hsa-let-7i-5p	FZR1
hsa-let-7i-5p	GRB10
hsa-let-7i-5p	HNF4A
hsa-let-7i-5p	IL10
hsa-let-7i-5p	IL6
hsa-let-7i-5p	LTBP1
hsa-let-7i-5p	MBD1
hsa-let-7i-5p	MECP2
hsa-let-7i-5p	MTND4P12
hsa-let-7i-5p	NARS2
hsa-let-7i-5p	NEAT1
hsa-let-7i-5p	PCDH19
hsa-let-7i-5p	PLAUR
hsa-let-7i-5p	PLCG1
hsa-let-7i-5p	POLR2A
hsa-let-7i-5p	PRPF31
hsa-let-7i-5p	PTCH1
hsa-let-7i-5p	RBM28
hsa-let-7i-5p	RBMS1
hsa-let-7i-5p	SAMD4B
hsa-let-7i-5p	SENP5
hsa-let-7i-5p	SLC35C2
hsa-let-7i-5p	SNHG16
hsa-let-7i-5p	SSH1
hsa-let-7i-5p	TBRG4
hsa-let-7i-5p	TINCR
hsa-let-7i-5p	TMEM201
hsa-let-7i-5p	TNF
hsa-let-7i-5p	TNPO2
hsa-let-7i-5p	USP38
hsa-let-7i-5p	ZNF318
hsa-let-7i-5p	ZNF354B
hsa-miR-1-3p	CCDC124
hsa-miR-1-3p	CDCP1
hsa-miR-1-3p	CXCL8
hsa-miR-1-3p	HP1BP3
hsa-miR-1-3p	IL6
hsa-miR-1-3p	KHSRP
hsa-miR-1-3p	MAGI1
hsa-miR-1-3p	MECP2

hsa-miR-1-3p	NARS2
hsa-miR-1-3p	ND1
hsa-miR-1-3p	NEAT1
hsa-miR-1-3p	PLCG1
hsa-miR-1-3p	PLEKHA1
hsa-miR-1-3p	PPP2R3A
hsa-miR-1-3p	PRMT2
hsa-miR-1-3p	SAMD4B
hsa-miR-1-3p	SENCR
hsa-miR-1-3p	SLC12A2
hsa-miR-1-3p	SOX9
hsa-miR-1-3p	TIMP2
hsa-miR-1-3p	TNPO2
hsa-miR-1-3p	TPPP
hsa-miR-1-3p	ZMYND11
hsa-miR-1-3p	ZNF318
hsa-miR-101-3p	ARHGEF10
hsa-miR-101-3p	ATF3
hsa-miR-101-3p	BEND4
hsa-miR-101-3p	CARM1
hsa-miR-101-3p	CHMP3
hsa-miR-101-3p	CLIP1
hsa-miR-101-3p	CSDE1
hsa-miR-101-3p	CXCL8
hsa-miR-101-3p	DEPDC1B
hsa-miR-101-3p	DMXL2
hsa-miR-101-3p	EEF1A1P9
hsa-miR-101-3p	FNBP4
hsa-miR-101-3p	GRB10
hsa-miR-101-3p	GTF2H2
hsa-miR-101-3p	KHSRP
hsa-miR-101-3p	LTBP1
hsa-miR-101-3p	MAGI1
hsa-miR-101-3p	MECP2
hsa-miR-101-3p	MPPE1
hsa-miR-101-3p	NDUFV3
hsa-miR-101-3p	PLCG1
hsa-miR-101-3p	PLEKHA1
hsa-miR-101-3p	POLR2A
hsa-miR-101-3p	PPM1K
hsa-miR-101-3p	PTCH1
hsa-miR-101-3p	RABAC1
hsa-miR-101-3p	RBMS1

hsa-miR-101-3p	SH3BP4
hsa-miR-101-3p	SLC12A2
hsa-miR-101-3p	SSH1
hsa-miR-101-3p	TMEM201
hsa-miR-101-3p	TMTC2
hsa-miR-101-3p	TNPO2
hsa-miR-101-3p	USP38
hsa-miR-101-3p	ZNF318
hsa-miR-103a-2-5p	CARM1
hsa-miR-103a-2-5p	CLIP1
hsa-miR-103a-2-5p	DEPDC1B
hsa-miR-103a-2-5p	KHSRP
hsa-miR-103a-2-5p	LTBP1
hsa-miR-103a-2-5p	PTCH1
hsa-miR-103a-2-5p	TNPO2
hsa-miR-103a-2-5p	USP38
hsa-miR-103a-3p	ACO1
hsa-miR-103a-3p	ARHGEF10
hsa-miR-103a-3p	ATF3
hsa-miR-103a-3p	CARM1
hsa-miR-103a-3p	CHI3L1
hsa-miR-103a-3p	CHMP3
hsa-miR-103a-3p	CLIP1
hsa-miR-103a-3p	CSDE1
hsa-miR-103a-3p	DEPDC1B
hsa-miR-103a-3p	EEF1A1P9
hsa-miR-103a-3p	FNBP4
hsa-miR-103a-3p	GRB10
hsa-miR-103a-3p	GTF2F1
hsa-miR-103a-3p	HID1
hsa-miR-103a-3p	HNF4A
hsa-miR-103a-3p	IL6
hsa-miR-103a-3p	KHSRP
hsa-miR-103a-3p	MBD1
hsa-miR-103a-3p	MECP2
hsa-miR-103a-3p	NEAT1
hsa-miR-103a-3p	NSRP1
hsa-miR-103a-3p	PLCG1
hsa-miR-103a-3p	PLEKHA1
hsa-miR-103a-3p	PRPF31
hsa-miR-103a-3p	RABAC1
hsa-miR-103a-3p	SENP5
hsa-miR-103a-3p	SH3BP4

hsa-miR-103a-3p	SLC12A2
hsa-miR-103a-3p	SLC35C2
hsa-miR-103a-3p	SOX9
hsa-miR-103a-3p	SRPRA
hsa-miR-103a-3p	SRRM2
hsa-miR-103a-3p	SSH1
hsa-miR-103a-3p	TIMP2
hsa-miR-103a-3p	TMEM201
hsa-miR-103a-3p	TNPO2
hsa-miR-103a-3p	USP38
hsa-miR-103a-3p	ZMYND11
hsa-miR-105-3p	CLIP1
hsa-miR-105-3p	PLEKHA1
hsa-miR-105-3p	RBMS1
hsa-miR-105-3p	SLC12A2
hsa-miR-105-3p	SOX9
hsa-miR-105-3p	TNPO2
hsa-miR-105-5p	ARL6
hsa-miR-105-5p	ATF3
hsa-miR-105-5p	BNC1
hsa-miR-105-5p	CDCP1
hsa-miR-105-5p	CLIP1
hsa-miR-105-5p	DMXL2
hsa-miR-105-5p	EEF1A1P9
hsa-miR-105-5p	GRB10
hsa-miR-105-5p	L3MBTL2
hsa-miR-105-5p	LTBP1
hsa-miR-105-5p	MAGI1
hsa-miR-105-5p	MALAT1
hsa-miR-105-5p	MECP2
hsa-miR-105-5p	NDE1
hsa-miR-105-5p	NORAD
hsa-miR-105-5p	PLCG1
hsa-miR-105-5p	PLEKHA1
hsa-miR-105-5p	PPM1K
hsa-miR-105-5p	PTCH1
hsa-miR-105-5p	RBMS1
hsa-miR-105-5p	SAMD4B
hsa-miR-105-5p	SH3BP4
hsa-miR-105-5p	SLC12A2
hsa-miR-105-5p	SNHG16
hsa-miR-105-5p	SOX9
hsa-miR-105-5p	TIMP2

hsa-miR-105-5p	TMTC2
hsa-miR-105-5p	TNPO2
hsa-miR-105-5p	TUG1
hsa-miR-105-5p	USP38
hsa-miR-105-5p	ZNF354B
hsa-miR-106a-3p	CSDE1
hsa-miR-106a-3p	FNBP4
hsa-miR-106a-3p	LTBP1
hsa-miR-106a-3p	PLEKHA1
hsa-miR-106a-3p	PSMB5
hsa-miR-106a-3p	TIMP2
hsa-miR-106a-3p	ZIC3
hsa-miR-106a-3p	ZNF354B
hsa-miR-106a-5p	ARHGEF10
hsa-miR-106a-5p	ARL6
hsa-miR-106a-5p	BEND4
hsa-miR-106a-5p	C17orf58
hsa-miR-106a-5p	CARM1
hsa-miR-106a-5p	CCDC168
hsa-miR-106a-5p	CDCP1
hsa-miR-106a-5p	CHMP3
hsa-miR-106a-5p	CSDE1
hsa-miR-106a-5p	CXCL8
hsa-miR-106a-5p	FNBP4
hsa-miR-106a-5p	GRB10
hsa-miR-106a-5p	HP1BP3
hsa-miR-106a-5p	IL10
hsa-miR-106a-5p	IL6
hsa-miR-106a-5p	L3MBTL2
hsa-miR-106a-5p	MAGI1
hsa-miR-106a-5p	MALAT1
hsa-miR-106a-5p	MECP2
hsa-miR-106a-5p	MIR17HG
hsa-miR-106a-5p	NDUFV3
hsa-miR-106a-5p	NEAT1
hsa-miR-106a-5p	NORAD
hsa-miR-106a-5p	PCDH19
hsa-miR-106a-5p	PLEKHA1
hsa-miR-106a-5p	PPP2R3A
hsa-miR-106a-5p	PSMB5
hsa-miR-106a-5p	RBMS1
hsa-miR-106a-5p	RNU1-148P
hsa-miR-106a-5p	SENP5

hsa-miR-106a-5p	SH3BP4
hsa-miR-106a-5p	SLC12A2
hsa-miR-106a-5p	SNHG16
hsa-miR-106a-5p	SOX9
hsa-miR-106a-5p	SRRM2
hsa-miR-106a-5p	SSH1
hsa-miR-106a-5p	STS
hsa-miR-106a-5p	TENM1
hsa-miR-106a-5p	TINCR
hsa-miR-106a-5p	TMTC2
hsa-miR-106a-5p	TNF
hsa-miR-106a-5p	TPPP
hsa-miR-106a-5p	TUG1
hsa-miR-106a-5p	ZNF318
hsa-miR-106a-5p	ZNF354B
hsa-miR-106b-3p	CSDE1
hsa-miR-106b-3p	FNBP4
hsa-miR-106b-3p	MAGI1
hsa-miR-106b-3p	SH3BP4
hsa-miR-106b-3p	TIMP2
hsa-miR-106b-5p	AR
hsa-miR-106b-5p	ARHGEF10
hsa-miR-106b-5p	ATF3
hsa-miR-106b-5p	CARM1
hsa-miR-106b-5p	CHMP3
hsa-miR-106b-5p	CSDE1
hsa-miR-106b-5p	CXCL8
hsa-miR-106b-5p	FNBP4
hsa-miR-106b-5p	GRB10
hsa-miR-106b-5p	HNF4A
hsa-miR-106b-5p	HP1BP3
hsa-miR-106b-5p	IL6
hsa-miR-106b-5p	MALAT1
hsa-miR-106b-5p	MECP2
hsa-miR-106b-5p	NEAT1
hsa-miR-106b-5p	NORAD
hsa-miR-106b-5p	PLEKHA1
hsa-miR-106b-5p	PPP2R3A
hsa-miR-106b-5p	PRRG3
hsa-miR-106b-5p	RBMS1
hsa-miR-106b-5p	RNU1-148P
hsa-miR-106b-5p	SENP5
hsa-miR-106b-5p	SH3BP4

hsa-miR-106b-5p	SNHG16
hsa-miR-106b-5p	SSH1
hsa-miR-106b-5p	TIMP2
hsa-miR-106b-5p	TINCR
hsa-miR-106b-5p	TNF
hsa-miR-106b-5p	TPPP
hsa-miR-106b-5p	TUG1
hsa-miR-106b-5p	ZMYND11
hsa-miR-106b-5p	ZNF318
hsa-miR-106b-5p	ZNF354B
hsa-miR-107	ACO1
hsa-miR-107	ARHGEF10
hsa-miR-107	ATF3
hsa-miR-107	CARM1
hsa-miR-107	CDCP1
hsa-miR-107	CHI3L1
hsa-miR-107	CHMP3
hsa-miR-107	CLIP1
hsa-miR-107	CSDE1
hsa-miR-107	DEPDC1B
hsa-miR-107	EEF1A1P9
hsa-miR-107	FNBP4
hsa-miR-107	GRB10
hsa-miR-107	GTF2F1
hsa-miR-107	HID1
hsa-miR-107	HNF4A
hsa-miR-107	IL6
hsa-miR-107	KHSRP
hsa-miR-107	MBD1
hsa-miR-107	MECP2
hsa-miR-107	NEAT1
hsa-miR-107	NSRP1
hsa-miR-107	PLCG1
hsa-miR-107	PLEKHA1
hsa-miR-107	PRPF31
hsa-miR-107	PSMB5
hsa-miR-107	RABAC1
hsa-miR-107	SENP5
hsa-miR-107	SH3BP4
hsa-miR-107	SLC12A2
hsa-miR-107	SLC35C2
hsa-miR-107	SOX9
hsa-miR-107	SRPRA

hsa-miR-107	SRRM2
hsa-miR-107	SSH1
hsa-miR-107	STS
hsa-miR-107	TMEM201
hsa-miR-107	TNPO2
hsa-miR-107	USP38
hsa-miR-107	ZMYND11
hsa-miR-107	hsa-let-7a-5p
hsa-miR-107	hsa-let-7b-5p
hsa-miR-107	hsa-let-7c-5p
hsa-miR-107	hsa-let-7d-5p
hsa-miR-107	hsa-let-7e-5p
hsa-miR-107	hsa-let-7f-5p
hsa-miR-107	hsa-let-7g-5p
hsa-miR-107	hsa-let-7i-5p
hsa-miR-1179	ARHGEF10
hsa-miR-1179	ATF3
hsa-miR-1179	CDCP1
hsa-miR-1179	CHMP1B
hsa-miR-1179	CSDE1
hsa-miR-1179	FZR1
hsa-miR-1179	GTF2H2
hsa-miR-1179	HNF4A
hsa-miR-1179	LTBP1
hsa-miR-1179	MAGI1
hsa-miR-1179	MALAT1
hsa-miR-1179	MBD1
hsa-miR-1179	MPPE1
hsa-miR-1179	NEAT1
hsa-miR-1179	PCDH19
hsa-miR-1179	PLCG1
hsa-miR-1179	PLEKHA1
hsa-miR-1179	POLR2A
hsa-miR-1179	PPM1K
hsa-miR-1179	RBMS1
hsa-miR-1179	SLC12A2
hsa-miR-1179	SNHG16
hsa-miR-1179	SOX9
hsa-miR-1179	SRRM2
hsa-miR-1179	STS
hsa-miR-1179	USP38
hsa-miR-1179	ZMYND11
hsa-miR-1180-3p	AR

hsa-miR-1180-3p	HNF4A
hsa-miR-1180-3p	KHSRP
hsa-miR-1180-3p	ND5
hsa-miR-1180-3p	SNHG16
hsa-miR-1180-3p	TNPO2
hsa-miR-1185-5p	BEND4
hsa-miR-1185-5p	CHMP3
hsa-miR-1185-5p	HID1
hsa-miR-1185-5p	MECP2
hsa-miR-1185-5p	MTND4P12
hsa-miR-1185-5p	NARS2
hsa-miR-1185-5p	NEAT1
hsa-miR-1185-5p	TIMP2
hsa-miR-1185-5p	TNPO2
hsa-miR-1185-5p	USP38
hsa-miR-1193	EEF1A1P9
hsa-miR-1193	HNF4A
hsa-miR-1193	L3MBTL2
hsa-miR-1193	MTND4P12
hsa-miR-1193	NME1-NME2
hsa-miR-1193	PSMB5
hsa-miR-1193	PTCH1
hsa-miR-1193	RABAC1
hsa-miR-1193	SH3BP4
hsa-miR-1193	SNHG16
hsa-miR-1193	SRRM2
hsa-miR-1193	STS
hsa-miR-1193	TINCR
hsa-miR-1193	TNPO2
hsa-miR-1197	AR
hsa-miR-1197	CHMP3
hsa-miR-1197	CSDE1
hsa-miR-1197	EML2
hsa-miR-1197	FAM214B
hsa-miR-1197	HNF4A
hsa-miR-1197	KHSRP
hsa-miR-1197	LTBP1
hsa-miR-1197	MAGI1
hsa-miR-1197	MAGI1-IT1
hsa-miR-1197	MECP2
hsa-miR-1197	NEAT1
hsa-miR-1197	PLCG1
hsa-miR-1197	POLR2A

hsa-miR-1197	RABAC1
hsa-miR-1197	SAMD4B
hsa-miR-1197	SLC12A2
hsa-miR-1197	SRPRA
hsa-miR-1197	SSH1
hsa-miR-1197	ZMYND11
hsa-miR-1205	ACO1
hsa-miR-1205	AR
hsa-miR-1205	NDE1
hsa-miR-1205	RPL30
hsa-miR-1205	SNHG16
hsa-miR-1207-3p	GRB10
hsa-miR-1207-3p	MPPE1
hsa-miR-1207-3p	RBMS1
hsa-miR-1207-3p	SNHG16
hsa-miR-1207-3p	SRPRA
hsa-miR-1207-3p	TNPO2
hsa-miR-122-5p	AC078899.1
hsa-miR-122-5p	ACO1
hsa-miR-122-5p	ARHGEF10
hsa-miR-122-5p	CHMP3
hsa-miR-122-5p	CXCL8
hsa-miR-122-5p	FAM214B
hsa-miR-122-5p	GRB10
hsa-miR-122-5p	GTF2F1
hsa-miR-122-5p	GTF2H2
hsa-miR-122-5p	HNF4A
hsa-miR-122-5p	HP1BP3
hsa-miR-122-5p	IL6
hsa-miR-122-5p	INTS3
hsa-miR-122-5p	MALAT1
hsa-miR-122-5p	MECP2
hsa-miR-122-5p	NEAT1
hsa-miR-122-5p	PLCG1
hsa-miR-122-5p	PPP2R3A
hsa-miR-122-5p	PRMT2
hsa-miR-122-5p	PSD4
hsa-miR-122-5p	PSMB5
hsa-miR-122-5p	PTCH1
hsa-miR-122-5p	SAMD4B
hsa-miR-122-5p	SH3BP4
hsa-miR-122-5p	SLC35C2
hsa-miR-122-5p	SNHG16

hsa-miR-122-5p	SOX9
hsa-miR-122-5p	SRPRA
hsa-miR-122-5p	SRRM2
hsa-miR-122-5p	TIMP2
hsa-miR-122-5p	TNPO2
hsa-miR-122-5p	ZNF354B
hsa-miR-1224-5p	ACO1
hsa-miR-1224-5p	ATF3
hsa-miR-1224-5p	CDCP1
hsa-miR-1224-5p	DMXL2
hsa-miR-1224-5p	ERCC4
hsa-miR-1224-5p	GRB10
hsa-miR-1224-5p	HID1
hsa-miR-1224-5p	INTS3
hsa-miR-1224-5p	NEAT1
hsa-miR-1224-5p	PCDH19
hsa-miR-1224-5p	PLCG1
hsa-miR-1224-5p	PRPF31
hsa-miR-1224-5p	SAMD4B
hsa-miR-1224-5p	SOX9
hsa-miR-1224-5p	SRPRA
hsa-miR-1224-5p	STS
hsa-miR-1224-5p	TIMP2
hsa-miR-1224-5p	TMEM201
hsa-miR-1224-5p	TNPO2
hsa-miR-1224-5p	TUG1
hsa-miR-1224-5p	USP38
hsa-miR-1224-5p	ZIC3
hsa-miR-1225-3p	CDCP1
hsa-miR-1225-3p	CHMP3
hsa-miR-1225-3p	TNPO2
hsa-miR-1225-3p	ZMYND11
hsa-miR-1226-3p	CSDE1
hsa-miR-1226-3p	HNF4A
hsa-miR-1226-3p	NDUFV3
hsa-miR-1226-3p	SAMD4B
hsa-miR-1226-3p	SNHG16
hsa-miR-1228-3p	HNF4A
hsa-miR-1228-3p	HP1BP3
hsa-miR-1229-3p	CSDE1
hsa-miR-1229-3p	MALAT1
hsa-miR-1231	ACO1
hsa-miR-1231	HNF4A

hsa-miR-1237-3p	ACO1
hsa-miR-1237-3p	AR
hsa-miR-1237-3p	HNF4A
hsa-miR-1237-3p	PSMB5
hsa-miR-1237-3p	SRPRA
hsa-miR-124-3p	ACO1
hsa-miR-124-3p	ARHGEF10
hsa-miR-124-3p	CDCP1
hsa-miR-124-3p	CHMP1B
hsa-miR-124-3p	CLIP1
hsa-miR-124-3p	CSDE1
hsa-miR-124-3p	CXCL8
hsa-miR-124-3p	DEPDC1B
hsa-miR-124-3p	DMXL2
hsa-miR-124-3p	FAM214B
hsa-miR-124-3p	FNBP4
hsa-miR-124-3p	GRB10
hsa-miR-124-3p	HNF4A
hsa-miR-124-3p	IL10
hsa-miR-124-3p	IL6
hsa-miR-124-3p	LTBP1
hsa-miR-124-3p	MAGI1
hsa-miR-124-3p	MECP2
hsa-miR-124-3p	MROH1
hsa-miR-124-3p	PLEKHA1
hsa-miR-124-3p	PSD4
hsa-miR-124-3p	PSMB5
hsa-miR-124-3p	RBM28
hsa-miR-124-3p	RBMS1
hsa-miR-124-3p	SAMD4B
hsa-miR-124-3p	SH3BP4
hsa-miR-124-3p	SLC12A2
hsa-miR-124-3p	SNHG16
hsa-miR-124-3p	SRRM2
hsa-miR-124-3p	SSH1
hsa-miR-124-3p	TENM1
hsa-miR-124-3p	TMEM201
hsa-miR-124-3p	TMTC2
hsa-miR-124-3p	TNPO2
hsa-miR-124-3p	USP38
hsa-miR-124-3p	ZNF318
hsa-miR-1249-3p	HNF4A
hsa-miR-1249-3p	PTCH1

hsa-miR-1249-3p	RBMS1
hsa-miR-1249-3p	SH3BP4
hsa-miR-1249-3p	TMTC2
hsa-miR-1252-5p	ACO1
hsa-miR-1252-5p	ARHGEF10
hsa-miR-1252-5p	CDCP1
hsa-miR-1252-5p	CHMP3
hsa-miR-1252-5p	CLIP1
hsa-miR-1252-5p	GRB10
hsa-miR-1252-5p	HID1
hsa-miR-1252-5p	HNF4A
hsa-miR-1252-5p	HP1BP3
hsa-miR-1252-5p	IL6
hsa-miR-1252-5p	KHSRP
hsa-miR-1252-5p	LTBP1
hsa-miR-1252-5p	MALAT1
hsa-miR-1252-5p	MECP2
hsa-miR-1252-5p	MIR17HG
hsa-miR-1252-5p	NDE1
hsa-miR-1252-5p	PSD4
hsa-miR-1252-5p	RPL30
hsa-miR-1252-5p	SAMD4B
hsa-miR-1252-5p	SH3BP4
hsa-miR-1252-5p	SRPRA
hsa-miR-1252-5p	TENM1
hsa-miR-1252-5p	TIMP2
hsa-miR-1252-5p	TNPO2
hsa-miR-125a-3p	CARM1
hsa-miR-125a-3p	CDCP1
hsa-miR-125a-3p	CHMP3
hsa-miR-125a-3p	CLIP1
hsa-miR-125a-3p	CSDE1
hsa-miR-125a-3p	FAM214B
hsa-miR-125a-3p	FNBP4
hsa-miR-125a-3p	HP1BP3
hsa-miR-125a-3p	IL10
hsa-miR-125a-3p	IL6
hsa-miR-125a-3p	INTS3
hsa-miR-125a-3p	MBD1
hsa-miR-125a-3p	NARS2
hsa-miR-125a-3p	NORAD
hsa-miR-125a-3p	RBM28
hsa-miR-125a-3p	RPL30

hsa-miR-125a-3p	SLC35C2
hsa-miR-125a-3p	SRRM2
hsa-miR-125a-3p	hsa-miR-1249-3p
hsa-miR-125a-5p	CARM1
hsa-miR-125a-5p	CSDE1
hsa-miR-125a-5p	GRB10
hsa-miR-125a-5p	HID1
hsa-miR-125a-5p	HNF4A
hsa-miR-125a-5p	IL10
hsa-miR-125a-5p	MALAT1
hsa-miR-125a-5p	MBD1
hsa-miR-125a-5p	NORAD
hsa-miR-125a-5p	SH3BP4
hsa-miR-125a-5p	TNPO2
hsa-miR-125b-5p	CARM1
hsa-miR-125b-5p	CCDC124
hsa-miR-125b-5p	CHMP3
hsa-miR-125b-5p	CSDE1
hsa-miR-125b-5p	GRB10
hsa-miR-125b-5p	HID1
hsa-miR-125b-5p	KHSRP
hsa-miR-125b-5p	MBD1
hsa-miR-125b-5p	SRRM2
hsa-miR-125b-5p	TNF
hsa-miR-125b-5p	ZMYND11
hsa-miR-1270	CSDE1
hsa-miR-1270	FNBP4
hsa-miR-1270	SRRM2
hsa-miR-1271-5p	ACO1
hsa-miR-1271-5p	AR
hsa-miR-1271-5p	CHMP1B
hsa-miR-1271-5p	HNF4A
hsa-miR-1271-5p	KHSRP
hsa-miR-1271-5p	MAGI1
hsa-miR-1271-5p	MALAT1
hsa-miR-1271-5p	MECP2
hsa-miR-1271-5p	NARS2
hsa-miR-1271-5p	NME1-NME2
hsa-miR-1271-5p	PPP2R3A
hsa-miR-1271-5p	SH3BP4
hsa-miR-1271-5p	SLC12A2
hsa-miR-1273f	AR
hsa-miR-1273f	HNF4A

hsa-miR-1273f	MALAT1
hsa-miR-1273f	NDUFV3
hsa-miR-1273f	PLEKHA1
hsa-miR-1273f	SNHG16
hsa-miR-1275	AR
hsa-miR-1275	SLC35C2
hsa-miR-1275	SRRM2
hsa-miR-1275	TMTC2
hsa-miR-1278	AR
hsa-miR-1278	HNF4A
hsa-miR-1278	MTND4P12
hsa-miR-1278	PLEKHA1
hsa-miR-1278	PSMB5
hsa-miR-1278	SENP5
hsa-miR-1278	USP38
hsa-miR-1278	hsa-miR-101-3p
hsa-miR-128-3p	AR
hsa-miR-128-3p	ARL6
hsa-miR-128-3p	BNC1
hsa-miR-128-3p	CDCP1
hsa-miR-128-3p	DEPDC1B
hsa-miR-128-3p	GRB10
hsa-miR-128-3p	GTF2H2
hsa-miR-128-3p	HP1BP3
hsa-miR-128-3p	KHSRP
hsa-miR-128-3p	LTBP1
hsa-miR-128-3p	NARS2
hsa-miR-128-3p	NEAT1
hsa-miR-128-3p	PLEKHA1
hsa-miR-128-3p	PTCH1
hsa-miR-128-3p	RBMS1
hsa-miR-128-3p	SENP5
hsa-miR-128-3p	SNHG16
hsa-miR-128-3p	SOX9
hsa-miR-128-3p	SRPRA
hsa-miR-128-3p	SSH1
hsa-miR-128-3p	TBRG4
hsa-miR-128-3p	TIMP2
hsa-miR-128-3p	TMTC2
hsa-miR-128-3p	TPPP
hsa-miR-128-3p	TUG1
hsa-miR-128-3p	USP38
hsa-miR-128-3p	hsa-miR-106b-5p

hsa-miR-1285-3p	SNHG16
hsa-miR-1285-3p	SRPRA
hsa-miR-1285-3p	TMTC2
hsa-miR-1286	NDE1
hsa-miR-1286	TMEM201
hsa-miR-1286	ZMYND11
hsa-miR-1289	FNBP4
hsa-miR-1297	BEND4
hsa-miR-1297	C17orf58
hsa-miR-1297	CARM1
hsa-miR-1297	DEPDC1B
hsa-miR-1297	DMXL2
hsa-miR-1297	FAM214B
hsa-miR-1297	GRB10
hsa-miR-1297	IL6
hsa-miR-1297	LTBP1
hsa-miR-1297	MALAT1
hsa-miR-1297	MECP2
hsa-miR-1297	MPPE1
hsa-miR-1297	PCDH19
hsa-miR-1297	PTCH1
hsa-miR-1297	RBMS1
hsa-miR-1297	SENP5
hsa-miR-1297	SLC12A2
hsa-miR-1297	TUG1
hsa-miR-130a-3p	AR
hsa-miR-130a-3p	ATF3
hsa-miR-130a-3p	CHMP3
hsa-miR-130a-3p	CLIP1
hsa-miR-130a-3p	CSDE1
hsa-miR-130a-3p	ERCC4
hsa-miR-130a-3p	FNBP4
hsa-miR-130a-3p	GRB10
hsa-miR-130a-3p	HNF4A
hsa-miR-130a-3p	KHSRP
hsa-miR-130a-3p	LTBP1
hsa-miR-130a-3p	MECP2
hsa-miR-130a-3p	MIR17HG
hsa-miR-130a-3p	PLEKHA1
hsa-miR-130a-3p	PSMB5
hsa-miR-130a-3p	RNVU1-7
hsa-miR-130a-3p	SLC12A2
hsa-miR-130a-3p	TENM1

hsa-miR-130a-3p	TIMP2
hsa-miR-130a-3p	TNF
hsa-miR-130a-3p	ZMYND11
hsa-miR-130a-3p	ZNF354B
hsa-miR-130b-3p	AR
hsa-miR-130b-3p	CARM1
hsa-miR-130b-3p	CHMP3
hsa-miR-130b-3p	CLIP1
hsa-miR-130b-3p	CSDE1
hsa-miR-130b-3p	ERCC4
hsa-miR-130b-3p	FNBP4
hsa-miR-130b-3p	GRB10
hsa-miR-130b-3p	HNF4A
hsa-miR-130b-3p	LTBP1
hsa-miR-130b-3p	MECP2
hsa-miR-130b-3p	MIR17HG
hsa-miR-130b-3p	MPPE1
hsa-miR-130b-3p	PLEKHA1
hsa-miR-130b-3p	PPM1K
hsa-miR-130b-3p	PPP2R3A
hsa-miR-130b-3p	PSMB5
hsa-miR-130b-3p	RNVU1-7
hsa-miR-130b-3p	SLC12A2
hsa-miR-130b-3p	TENM1
hsa-miR-130b-3p	TIMP2
hsa-miR-130b-3p	TMEM201
hsa-miR-130b-3p	TNF
hsa-miR-130b-3p	ZMYND11
hsa-miR-130b-3p	ZNF354B
hsa-miR-130b-3p	hsa-miR-130a-3p
hsa-miR-132-3p	ARHGEF10
hsa-miR-132-3p	CHMP1B
hsa-miR-132-3p	CSDE1
hsa-miR-132-3p	GTF2H2
hsa-miR-132-3p	HNF4A
hsa-miR-132-3p	IL10
hsa-miR-132-3p	MAGI1
hsa-miR-132-3p	MECP2
hsa-miR-132-3p	NARS2
hsa-miR-132-3p	POLR2A
hsa-miR-132-3p	PTCH1
hsa-miR-132-3p	RBMS1
hsa-miR-132-3p	SNHG16

hsa-miR-132-3p	TUG1
hsa-miR-132-3p	USP38
hsa-miR-133a-3p	C17orf58
hsa-miR-133a-3p	CDCP1
hsa-miR-133a-3p	CHMP3
hsa-miR-133a-3p	MALAT1
hsa-miR-133a-3p	MECP2
hsa-miR-133a-3p	RBMS1
hsa-miR-133a-3p	SH3BP4
hsa-miR-133a-3p	TINCR
hsa-miR-133a-3p	TMEM201
hsa-miR-133a-3p	TNPO2
hsa-miR-133a-3p	hsa-miR-1286
hsa-miR-134-5p	CDCP1
hsa-miR-134-5p	FNBP4
hsa-miR-134-5p	HNF4A
hsa-miR-134-5p	HP1BP3
hsa-miR-134-5p	PLEKHA1
hsa-miR-134-5p	PSMB5
hsa-miR-134-5p	TNPO2
hsa-miR-134-5p	hsa-miR-128-3p
hsa-miR-135a-5p	ARL6
hsa-miR-135a-5p	ATF3
hsa-miR-135a-5p	CSDE1
hsa-miR-135a-5p	IL6
hsa-miR-135a-5p	INTS3
hsa-miR-135a-5p	NDUFV3
hsa-miR-135a-5p	RBMS1
hsa-miR-135a-5p	SSH1
hsa-miR-135a-5p	STS
hsa-miR-135a-5p	TNPO2
hsa-miR-135a-5p	ZMYND11
hsa-miR-135b-5p	AR
hsa-miR-135b-5p	ARL6
hsa-miR-135b-5p	ATF3
hsa-miR-135b-5p	HNF4A
hsa-miR-135b-5p	IL6
hsa-miR-135b-5p	INTS3
hsa-miR-135b-5p	NDUFV3
hsa-miR-135b-5p	SSH1
hsa-miR-135b-5p	STS
hsa-miR-135b-5p	TNPO2
hsa-miR-135b-5p	ZMYND11

hsa-miR-140-5p	ATF3
hsa-miR-140-5p	CHMP3
hsa-miR-140-5p	CSDE1
hsa-miR-140-5p	ERCC4
hsa-miR-140-5p	HNF4A
hsa-miR-140-5p	IL6
hsa-miR-140-5p	MALAT1
hsa-miR-140-5p	PPM1K
hsa-miR-140-5p	PPP2R3A
hsa-miR-140-5p	SNHG16
hsa-miR-140-5p	SOX9
hsa-miR-141-3p	AR
hsa-miR-141-3p	ATF3
hsa-miR-141-3p	BEND4
hsa-miR-141-3p	CARM1
hsa-miR-141-3p	CSDE1
hsa-miR-141-3p	DEPDC1B
hsa-miR-141-3p	GRB10
hsa-miR-141-3p	GTF2H2
hsa-miR-141-3p	HNF4A
hsa-miR-141-3p	IL10
hsa-miR-141-3p	LTBP1
hsa-miR-141-3p	MALAT1
hsa-miR-141-3p	NME1-NME2
hsa-miR-141-3p	PPM1K
hsa-miR-141-3p	PTCH1
hsa-miR-141-3p	RBM28
hsa-miR-141-3p	RBMS1
hsa-miR-141-3p	SH3BP4
hsa-miR-141-3p	SLC12A2
hsa-miR-141-3p	SNHG16
hsa-miR-141-3p	SOX9
hsa-miR-141-3p	TMTC2
hsa-miR-141-3p	ZMYND11
hsa-miR-141-3p	hsa-miR-107
hsa-miR-142-3p	ARHGEF10
hsa-miR-142-3p	CHMP3
hsa-miR-142-3p	CLIP1
hsa-miR-142-3p	CSDE1
hsa-miR-142-3p	FNBP4
hsa-miR-142-3p	GRB10
hsa-miR-142-3p	IL10
hsa-miR-142-3p	IL6

hsa-miR-142-3p	KHSRP
hsa-miR-142-3p	MAGI1
hsa-miR-142-3p	MECP2
hsa-miR-142-3p	POLR2A
hsa-miR-142-3p	PSMB5
hsa-miR-142-3p	PTCH1
hsa-miR-142-3p	RBMS1
hsa-miR-142-3p	SLC12A2
hsa-miR-142-3p	SNHG16
hsa-miR-142-3p	SOX9
hsa-miR-142-3p	STS
hsa-miR-142-3p	TUG1
hsa-miR-143-3p	ARL6
hsa-miR-143-3p	CARM1
hsa-miR-143-3p	CLIP1
hsa-miR-143-3p	CSDE1
hsa-miR-143-3p	DEPDC1B
hsa-miR-143-3p	HNF4A
hsa-miR-143-3p	KHSRP
hsa-miR-143-3p	LTBP1
hsa-miR-143-3p	PLEKHA1
hsa-miR-143-3p	PPP2R3A
hsa-miR-143-3p	PTCH1
hsa-miR-143-3p	SH3BP4
hsa-miR-143-3p	SRRM2
hsa-miR-143-3p	STS
hsa-miR-143-3p	TNF
hsa-miR-143-3p	TNPO2
hsa-miR-143-3p	ZMYND11
hsa-miR-143-3p	ZNF318
hsa-miR-144-3p	ARHGEF10
hsa-miR-144-3p	CRNDE
hsa-miR-144-3p	DMXL2
hsa-miR-144-3p	GTF2H2
hsa-miR-144-3p	HNF4A
hsa-miR-144-3p	HP1BP3
hsa-miR-144-3p	IL6
hsa-miR-144-3p	MAGI1
hsa-miR-144-3p	MALAT1
hsa-miR-144-3p	MECP2
hsa-miR-144-3p	PPM1K
hsa-miR-144-3p	PPP2R3A
hsa-miR-144-3p	PTCH1

hsa-miR-144-3p	RPL30
hsa-miR-144-3p	SH3BP4
hsa-miR-144-3p	SLC12A2
hsa-miR-144-3p	SOX9
hsa-miR-144-3p	TMEM201
hsa-miR-144-3p	TMTC2
hsa-miR-144-3p	USP38
hsa-miR-144-3p	ZNF318
hsa-miR-144-3p	hsa-miR-142-3p
hsa-miR-146a-3p	CSDE1
hsa-miR-146a-3p	PLEKHA1
hsa-miR-146a-3p	TNPO2
hsa-miR-146a-3p	USP38
hsa-miR-146a-5p	CXCL8
hsa-miR-146a-5p	FNBP4
hsa-miR-146a-5p	GTF2F1
hsa-miR-146a-5p	HNF4A
hsa-miR-146a-5p	HP1BP3
hsa-miR-146a-5p	IL6
hsa-miR-146a-5p	MPPE1
hsa-miR-146a-5p	PLAUR
hsa-miR-146a-5p	SAMD4B
hsa-miR-146a-5p	SNHG16
hsa-miR-146a-5p	TMTC2
hsa-miR-146a-5p	TNPO2
hsa-miR-146b-3p	HP1BP3
hsa-miR-146b-3p	NDUFV3
hsa-miR-146b-3p	TNPO2
hsa-miR-146b-3p	USP38
hsa-miR-146b-3p	ZNF354B
hsa-miR-146b-5p	AFAP1-AS1
hsa-miR-146b-5p	AR
hsa-miR-146b-5p	BEND4
hsa-miR-146b-5p	CXCL8
hsa-miR-146b-5p	DEPDC1B
hsa-miR-146b-5p	FNBP4
hsa-miR-146b-5p	GTF2F1
hsa-miR-146b-5p	HNF4A
hsa-miR-146b-5p	HP1BP3
hsa-miR-146b-5p	IL10
hsa-miR-146b-5p	IL6
hsa-miR-146b-5p	MALAT1
hsa-miR-146b-5p	MPPE1

hsa-miR-146b-5p	SAMD4B
hsa-miR-146b-5p	SNHG16
hsa-miR-146b-5p	TNPO2
hsa-miR-147a	AR
hsa-miR-147a	CDCP1
hsa-miR-147a	HNF4A
hsa-miR-147a	STS
hsa-miR-147a	TNPO2
hsa-miR-149-3p	CARM1
hsa-miR-149-3p	CHMP1B
hsa-miR-149-3p	CLIP1
hsa-miR-149-3p	CSDE1
hsa-miR-149-3p	KHSRP
hsa-miR-149-3p	PLEKHA1
hsa-miR-149-3p	PPM1K
hsa-miR-149-3p	RBMS1
hsa-miR-149-3p	SRPRA
hsa-miR-149-3p	SRRM2
hsa-miR-149-3p	TIMP2
hsa-miR-149-3p	ZNF318
hsa-miR-149-5p	ARHGEF10
hsa-miR-149-5p	ATF3
hsa-miR-149-5p	CARM1
hsa-miR-149-5p	CLIP1
hsa-miR-149-5p	HNF4A
hsa-miR-149-5p	IL6
hsa-miR-149-5p	INTS3
hsa-miR-149-5p	KHSRP
hsa-miR-149-5p	MECP2
hsa-miR-149-5p	PCDH19
hsa-miR-149-5p	SBF2-AS1
hsa-miR-149-5p	SRPRA
hsa-miR-149-5p	TBRG4
hsa-miR-149-5p	TNF
hsa-miR-149-5p	TNPO2
hsa-miR-149-5p	ZMYND11
hsa-miR-149-5p	ZNF318
hsa-miR-150-3p	ACO1
hsa-miR-150-3p	PLCG1
hsa-miR-150-3p	PLEKHA1
hsa-miR-150-3p	TNPO2
hsa-miR-150-5p	ACO1
hsa-miR-150-5p	CLIP1

hsa-miR-150-5p	CSDE1
hsa-miR-150-5p	GTF2H2
hsa-miR-150-5p	HNF4A
hsa-miR-150-5p	MAGI1
hsa-miR-150-5p	MBD1
hsa-miR-150-5p	NDUFV3
hsa-miR-150-5p	PLCG1
hsa-miR-150-5p	PLEKHA1
hsa-miR-150-5p	PPP2R3A
hsa-miR-150-5p	RABAC1
hsa-miR-150-5p	TNF
hsa-miR-150-5p	TNPO2
hsa-miR-150-5p	hsa-miR-125a-5p
hsa-miR-150-5p	hsa-miR-143-3p
hsa-miR-152-3p	CDCP1
hsa-miR-152-3p	CHMP3
hsa-miR-152-3p	CLIP1
hsa-miR-152-3p	FNBP4
hsa-miR-152-3p	LTBP1
hsa-miR-152-3p	MBD1
hsa-miR-152-3p	MECP2
hsa-miR-152-3p	PTCH1
hsa-miR-152-3p	SENP5
hsa-miR-152-3p	TBRG4
hsa-miR-152-3p	TNF
hsa-miR-152-3p	USP38
hsa-miR-152-3p	hsa-miR-122-5p
hsa-miR-152-3p	hsa-miR-134-5p
hsa-miR-153-3p	ATF3
hsa-miR-153-3p	CSDE1
hsa-miR-153-3p	DMXL2
hsa-miR-153-3p	FNBP4
hsa-miR-153-3p	IL6
hsa-miR-153-3p	MAGI1
hsa-miR-153-3p	MIR17HG
hsa-miR-153-3p	PLEKHA1
hsa-miR-153-3p	PPM1K
hsa-miR-153-3p	PTCH1
hsa-miR-153-3p	SH3BP4
hsa-miR-153-3p	SSH1
hsa-miR-153-3p	TMTC2
hsa-miR-153-3p	TUG1
hsa-miR-153-3p	ZMYND11

hsa-miR-153-3p	ZNF354B
hsa-miR-155-5p	ATF3
hsa-miR-155-5p	CSDE1
hsa-miR-155-5p	CXCL8
hsa-miR-155-5p	ERCC4
hsa-miR-155-5p	GRB10
hsa-miR-155-5p	HNF4A
hsa-miR-155-5p	MALAT1
hsa-miR-155-5p	MECP2
hsa-miR-155-5p	MIR17HG
hsa-miR-155-5p	PLEKHA1
hsa-miR-155-5p	PSMB5
hsa-miR-155-5p	SH3BP4
hsa-miR-155-5p	SLC35C2
hsa-miR-155-5p	SOX9
hsa-miR-155-5p	TMTC2
hsa-miR-155-5p	USP38
hsa-miR-155-5p	ZIC3
hsa-miR-155-5p	ZMYND11
hsa-miR-16-1-3p	ACO1
hsa-miR-16-1-3p	CARM1
hsa-miR-16-1-3p	CLIP1
hsa-miR-16-1-3p	FNBP4
hsa-miR-16-1-3p	HP1BP3
hsa-miR-16-1-3p	POLR2A
hsa-miR-16-1-3p	PPM1K
hsa-miR-16-1-3p	RBMS1
hsa-miR-16-1-3p	SLC12A2
hsa-miR-16-1-3p	SRPRA
hsa-miR-16-1-3p	USP38
hsa-miR-16-1-3p	ZNF318
hsa-miR-16-5p	ACO1
hsa-miR-16-5p	AR
hsa-miR-16-5p	CARM1
hsa-miR-16-5p	CHMP3
hsa-miR-16-5p	CSDE1
hsa-miR-16-5p	FNBP4
hsa-miR-16-5p	GRB10
hsa-miR-16-5p	IL6
hsa-miR-16-5p	MBD1
hsa-miR-16-5p	MECP2
hsa-miR-16-5p	PLEKHA1
hsa-miR-16-5p	POLR2A

hsa-miR-16-5p	PSMB5
hsa-miR-16-5p	PTCH1
hsa-miR-16-5p	RBMS1
hsa-miR-16-5p	SENP5
hsa-miR-16-5p	SH3BP4
hsa-miR-16-5p	SLC12A2
hsa-miR-16-5p	SNHG16
hsa-miR-16-5p	SOX9
hsa-miR-16-5p	SRPRA
hsa-miR-16-5p	TMEM201
hsa-miR-16-5p	TNF
hsa-miR-16-5p	USP38
hsa-miR-16-5p	ZNF318
hsa-miR-16-5p	hsa-miR-106b-5p
hsa-miR-17-3p	C17orf58
hsa-miR-17-3p	CARM1
hsa-miR-17-3p	CSDE1
hsa-miR-17-3p	FNBP4
hsa-miR-17-3p	GRB10
hsa-miR-17-3p	MALAT1
hsa-miR-17-3p	NDUFV3
hsa-miR-17-3p	POLR2A
hsa-miR-17-3p	PRMT2
hsa-miR-17-3p	TIMP2
hsa-miR-17-3p	USP38
hsa-miR-17-5p	ARHGEF10
hsa-miR-17-5p	ARL6
hsa-miR-17-5p	ATF3
hsa-miR-17-5p	CSDE1
hsa-miR-17-5p	CXCL8
hsa-miR-17-5p	FNBP4
hsa-miR-17-5p	HNF4A
hsa-miR-17-5p	HP1BP3
hsa-miR-17-5p	IL6
hsa-miR-17-5p	L3MBTL2
hsa-miR-17-5p	MECP2
hsa-miR-17-5p	ND2
hsa-miR-17-5p	ND4
hsa-miR-17-5p	PPP2R3A
hsa-miR-17-5p	PSMB5
hsa-miR-17-5p	RBMS1
hsa-miR-17-5p	SENP5
hsa-miR-17-5p	SH3BP4

hsa-miR-17-5p	SNHG16
hsa-miR-17-5p	SRRM2
hsa-miR-17-5p	SSH1
hsa-miR-17-5p	TNF
hsa-miR-17-5p	TPPP
hsa-miR-17-5p	TUG1
hsa-miR-17-5p	USP38
hsa-miR-17-5p	ZNF318
hsa-miR-17-5p	ZNF354B
hsa-miR-17-5p	hsa-miR-149-5p
hsa-miR-17-5p	hsa-miR-155-5p
hsa-miR-181a-5p	ACO1
hsa-miR-181a-5p	AFAP1-AS1
hsa-miR-181a-5p	CARM1
hsa-miR-181a-5p	CDCP1
hsa-miR-181a-5p	CHMP1B
hsa-miR-181a-5p	CHMP3
hsa-miR-181a-5p	CLIP1
hsa-miR-181a-5p	CRNDE
hsa-miR-181a-5p	CSDE1
hsa-miR-181a-5p	DMXL2
hsa-miR-181a-5p	FNBP4
hsa-miR-181a-5p	GRB10
hsa-miR-181a-5p	IL10
hsa-miR-181a-5p	KHSRP
hsa-miR-181a-5p	MBD1
hsa-miR-181a-5p	MECP2
hsa-miR-181a-5p	ND2
hsa-miR-181a-5p	NEAT1
hsa-miR-181a-5p	PLEKHA1
hsa-miR-181a-5p	PPP2R3A
hsa-miR-181a-5p	RBMS1
hsa-miR-181a-5p	SLC35C2
hsa-miR-181a-5p	TNF
hsa-miR-181a-5p	ZMYND11
hsa-miR-181b-5p	ACO1
hsa-miR-181b-5p	ARL6
hsa-miR-181b-5p	CARM1
hsa-miR-181b-5p	CDCP1
hsa-miR-181b-5p	CHMP1B
hsa-miR-181b-5p	CHMP3
hsa-miR-181b-5p	CLIP1
hsa-miR-181b-5p	CSDE1

hsa-miR-181b-5p	CXCL8
hsa-miR-181b-5p	DEPDC1B
hsa-miR-181b-5p	DMXL2
hsa-miR-181b-5p	FNBP4
hsa-miR-181b-5p	GRB10
hsa-miR-181b-5p	IL10
hsa-miR-181b-5p	KHSRP
hsa-miR-181b-5p	MBD1
hsa-miR-181b-5p	MECP2
hsa-miR-181b-5p	NDE1
hsa-miR-181b-5p	PLEKHA1
hsa-miR-181b-5p	PPP2R3A
hsa-miR-181b-5p	PTCH1
hsa-miR-181b-5p	RBMS1
hsa-miR-181b-5p	SLC35C2
hsa-miR-181b-5p	SRRM2
hsa-miR-181b-5p	STS
hsa-miR-181b-5p	TNF
hsa-miR-181b-5p	ZMYND11
hsa-miR-181c-5p	ACO1
hsa-miR-181c-5p	CARM1
hsa-miR-181c-5p	CDCP1
hsa-miR-181c-5p	CHMP1B
hsa-miR-181c-5p	CHMP3
hsa-miR-181c-5p	CLIP1
hsa-miR-181c-5p	CSDE1
hsa-miR-181c-5p	DMXL2
hsa-miR-181c-5p	FNBP4
hsa-miR-181c-5p	GRB10
hsa-miR-181c-5p	HNF4A
hsa-miR-181c-5p	IL10
hsa-miR-181c-5p	KHSRP
hsa-miR-181c-5p	MBD1
hsa-miR-181c-5p	MECP2
hsa-miR-181c-5p	PLEKHA1
hsa-miR-181c-5p	PPP2R3A
hsa-miR-181c-5p	RBMS1
hsa-miR-181c-5p	SLC35C2
hsa-miR-181c-5p	TNF
hsa-miR-181c-5p	ZMYND11
hsa-miR-181d-5p	ACO1
hsa-miR-181d-5p	ARL6
hsa-miR-181d-5p	CARM1

hsa-miR-181d-5p	CDCP1
hsa-miR-181d-5p	CHMP1B
hsa-miR-181d-5p	CHMP3
hsa-miR-181d-5p	CLIP1
hsa-miR-181d-5p	CSDE1
hsa-miR-181d-5p	CXCL8
hsa-miR-181d-5p	DEPDC1B
hsa-miR-181d-5p	DMXL2
hsa-miR-181d-5p	FNBP4
hsa-miR-181d-5p	GRB10
hsa-miR-181d-5p	HNF4A
hsa-miR-181d-5p	HP1BP3
hsa-miR-181d-5p	IL10
hsa-miR-181d-5p	KHSRP
hsa-miR-181d-5p	LTBP1
hsa-miR-181d-5p	MBD1
hsa-miR-181d-5p	MECP2
hsa-miR-181d-5p	NEAT1
hsa-miR-181d-5p	PLEKHA1
hsa-miR-181d-5p	PPP2R3A
hsa-miR-181d-5p	RBMS1
hsa-miR-181d-5p	SLC35C2
hsa-miR-181d-5p	STS
hsa-miR-181d-5p	TNF
hsa-miR-181d-5p	ZMYND11
hsa-miR-181d-5p	hsa-miR-128-3p
hsa-miR-181d-5p	hsa-miR-181a-5p
hsa-miR-181d-5p	hsa-miR-181b-5p
hsa-miR-181d-5p	hsa-miR-181c-5p
hsa-miR-182-3p	ACO1
hsa-miR-182-3p	CARM1
hsa-miR-182-3p	MECP2
hsa-miR-182-3p	SH3BP4
hsa-miR-182-3p	SLC12A2
hsa-miR-182-5p	ARHGEF10
hsa-miR-182-5p	C17orf58
hsa-miR-182-5p	CARM1
hsa-miR-182-5p	CHMP1B
hsa-miR-182-5p	DMXL2
hsa-miR-182-5p	HNF4A
hsa-miR-182-5p	HP1BP3
hsa-miR-182-5p	KHSRP
hsa-miR-182-5p	MAGI1

hsa-miR-182-5p	MECP2
hsa-miR-182-5p	NME1-NME2
hsa-miR-182-5p	PLEKHA1
hsa-miR-182-5p	PPP2R3A
hsa-miR-182-5p	PTCH1
hsa-miR-182-5p	RBM28
hsa-miR-182-5p	SH3BP4
hsa-miR-182-5p	SNHG16
hsa-miR-184	AR
hsa-miR-184	CARM1
hsa-miR-184	CSDE1
hsa-miR-184	PLEKHA1
hsa-miR-184	RBMS1
hsa-miR-184	SENP5
hsa-miR-184	SRRM2
hsa-miR-184	TNPO2
hsa-miR-184	hsa-miR-1237-3p
hsa-miR-186-5p	ACO1
hsa-miR-186-5p	ARHGEF10
hsa-miR-186-5p	ARL6
hsa-miR-186-5p	C17orf58
hsa-miR-186-5p	CHMP3
hsa-miR-186-5p	CLIP1
hsa-miR-186-5p	CRNDE
hsa-miR-186-5p	CSDE1
hsa-miR-186-5p	DEPDC1B
hsa-miR-186-5p	DMXL2
hsa-miR-186-5p	HNF4A
hsa-miR-186-5p	HP1BP3
hsa-miR-186-5p	KHSRP
hsa-miR-186-5p	MAGI1
hsa-miR-186-5p	ND5
hsa-miR-186-5p	NME1-NME2
hsa-miR-186-5p	NSRP1
hsa-miR-186-5p	PLEKHA1
hsa-miR-186-5p	RBM28
hsa-miR-186-5p	SLC12A2
hsa-miR-186-5p	SNHG16
hsa-miR-186-5p	SOX9
hsa-miR-186-5p	SRRM2
hsa-miR-186-5p	STS
hsa-miR-186-5p	TENM1
hsa-miR-186-5p	TMEM201

hsa-miR-186-5p	TNPO2
hsa-miR-186-5p	USP38
hsa-miR-186-5p	ZMYND11
hsa-miR-186-5p	hsa-miR-1179
hsa-miR-18a-5p	CLIP1
hsa-miR-18a-5p	CSDE1
hsa-miR-18a-5p	CXCL8
hsa-miR-18a-5p	FENDRR
hsa-miR-18a-5p	HNF4A
hsa-miR-18a-5p	L3MBTL2
hsa-miR-18a-5p	MBD1
hsa-miR-18a-5p	MECP2
hsa-miR-18a-5p	PSMB5
hsa-miR-18a-5p	RABAC1
hsa-miR-18a-5p	SH3BP4
hsa-miR-18a-5p	ZNF318
hsa-miR-18b-3p	CSDE1
hsa-miR-18b-3p	PLCG1
hsa-miR-18b-3p	TIMP2
hsa-miR-18b-5p	CLIP1
hsa-miR-18b-5p	CSDE1
hsa-miR-18b-5p	CXCL8
hsa-miR-18b-5p	GRB10
hsa-miR-18b-5p	L3MBTL2
hsa-miR-18b-5p	MBD1
hsa-miR-18b-5p	MECP2
hsa-miR-18b-5p	PSMB5
hsa-miR-18b-5p	SH3BP4
hsa-miR-18b-5p	SOX9
hsa-miR-18b-5p	ZNF318
hsa-miR-1909-3p	CARM1
hsa-miR-1909-3p	HNF4A
hsa-miR-1909-3p	SENP5
hsa-miR-1909-3p	TNPO2
hsa-miR-190a-5p	AR
hsa-miR-190a-5p	CSDE1
hsa-miR-190a-5p	DEPDC1B
hsa-miR-190a-5p	DMXL2
hsa-miR-190a-5p	HNF4A
hsa-miR-190a-5p	HP1BP3
hsa-miR-190a-5p	IL6
hsa-miR-190a-5p	PLEKHA1
hsa-miR-190a-5p	PTCH1

hsa-miR-190a-5p	RBMS1
hsa-miR-190a-5p	SAMD4B
hsa-miR-190a-5p	SNHG16
hsa-miR-190a-5p	SOX9
hsa-miR-190a-5p	STS
hsa-miR-190a-5p	USP38
hsa-miR-190a-5p	ZMYND11
hsa-miR-190b-5p	ACO1
hsa-miR-190b-5p	AR
hsa-miR-190b-5p	CSDE1
hsa-miR-190b-5p	DEPDC1B
hsa-miR-190b-5p	DMXL2
hsa-miR-190b-5p	HNF4A
hsa-miR-190b-5p	HP1BP3
hsa-miR-190b-5p	IL6
hsa-miR-190b-5p	PLEKHA1
hsa-miR-190b-5p	PTCH1
hsa-miR-190b-5p	RBMS1
hsa-miR-190b-5p	SAMD4B
hsa-miR-190b-5p	SNHG16
hsa-miR-190b-5p	SOX9
hsa-miR-190b-5p	STS
hsa-miR-190b-5p	USP38
hsa-miR-190b-5p	ZMYND11
hsa-miR-191-5p	HNF4A
hsa-miR-191-5p	LTBP1
hsa-miR-191-5p	MECP2
hsa-miR-191-5p	TIMP2
hsa-miR-191-5p	TNPO2
hsa-miR-1914-3p	PLCG1
hsa-miR-1914-3p	RBMS1
hsa-miR-1914-3p	SRRM2
hsa-miR-1914-3p	TNPO2
hsa-miR-1915-3p	HNF4A
hsa-miR-1915-3p	TNPO2
hsa-miR-1915-3p	TUG1
hsa-miR-1915-3p	hsa-miR-18a-5p
hsa-miR-1915-3p	hsa-miR-18b-5p
hsa-miR-192-5p	AR
hsa-miR-192-5p	ARHGEF10
hsa-miR-192-5p	C17orf58
hsa-miR-192-5p	ERCC4
hsa-miR-192-5p	HNF4A

hsa-miR-192-5p	HP1BP3
hsa-miR-192-5p	NDE1
hsa-miR-192-5p	PLEKHA1
hsa-miR-192-5p	RBMS1
hsa-miR-192-5p	SH3BP4
hsa-miR-192-5p	TMTC2
hsa-miR-192-5p	ZMYND11
hsa-miR-193b-3p	AR
hsa-miR-193b-3p	CARM1
hsa-miR-193b-3p	CLIP1
hsa-miR-193b-3p	CSDE1
hsa-miR-193b-3p	DMXL2
hsa-miR-193b-3p	HNF4A
hsa-miR-193b-3p	HP1BP3
hsa-miR-193b-3p	L3MBTL2
hsa-miR-193b-3p	MPPE1
hsa-miR-193b-3p	NEAT1
hsa-miR-193b-3p	PLAUR
hsa-miR-193b-3p	PRPF31
hsa-miR-193b-3p	PSD4
hsa-miR-193b-3p	PTCH1
hsa-miR-193b-3p	SENP5
hsa-miR-193b-3p	SH3BP4
hsa-miR-193b-3p	SNHG16
hsa-miR-193b-3p	ZNF318
hsa-miR-196a-5p	ACO1
hsa-miR-196a-5p	BEND4
hsa-miR-196a-5p	CDCP1
hsa-miR-196a-5p	CSDE1
hsa-miR-196a-5p	DEPDC1B
hsa-miR-196a-5p	DMXL2
hsa-miR-196a-5p	HP1BP3
hsa-miR-196a-5p	KHSRP
hsa-miR-196a-5p	LTBP1
hsa-miR-196a-5p	MECP2
hsa-miR-196a-5p	ND4
hsa-miR-196a-5p	ND5
hsa-miR-196a-5p	PCDH19
hsa-miR-196a-5p	SAMD4B
hsa-miR-196a-5p	SNHG16
hsa-miR-196a-5p	SOX9
hsa-miR-196a-5p	TENM1
hsa-miR-196a-5p	TUG1

hsa-miR-196a-5p	USP38
hsa-miR-196a-5p	ZNF354B
hsa-miR-197-3p	CSDE1
hsa-miR-197-3p	DEPDC1B
hsa-miR-197-3p	HNF4A
hsa-miR-197-3p	LTBP1
hsa-miR-197-3p	MBD1
hsa-miR-197-3p	NDUFV3
hsa-miR-197-3p	TIMP2
hsa-miR-197-3p	ZNF318
hsa-miR-1976	ACO1
hsa-miR-1976	GTF2H2
hsa-miR-1976	HNF4A
hsa-miR-1976	MPPE1
hsa-miR-1976	NDUFV3
hsa-miR-1976	PPM1K
hsa-miR-1976	SRRM2
hsa-miR-1976	TMEM201
hsa-miR-198	HNF4A
hsa-miR-198	PCDH19
hsa-miR-198	PTCH1
hsa-miR-198	SNHG16
hsa-miR-198	SOX9
hsa-miR-199a-3p	ARHGEF10
hsa-miR-199a-3p	ATF3
hsa-miR-199a-3p	CLIP1
hsa-miR-199a-3p	DEPDC1B
hsa-miR-199a-3p	MBD1
hsa-miR-199a-3p	MECP2
hsa-miR-199a-3p	NDUFV3
hsa-miR-199a-3p	PPM1K
hsa-miR-199a-3p	SH3BP4
hsa-miR-199a-3p	SLC12A2
hsa-miR-199a-3p	TUG1
hsa-miR-199a-3p	ZMYND11
hsa-miR-19a-3p	ACO1
hsa-miR-19a-3p	ARL6
hsa-miR-19a-3p	CDCP1
hsa-miR-19a-3p	CHMP3
hsa-miR-19a-3p	CLIP1
hsa-miR-19a-3p	CSDE1
hsa-miR-19a-3p	DMXL2
hsa-miR-19a-3p	ERCC4

hsa-miR-19a-3p	FNBP4
hsa-miR-19a-3p	GRB10
hsa-miR-19a-3p	GTF2H2
hsa-miR-19a-3p	HNF4A
hsa-miR-19a-3p	IL6
hsa-miR-19a-3p	MIR17HG
hsa-miR-19a-3p	NDE1
hsa-miR-19a-3p	PCDH19
hsa-miR-19a-3p	PRMT2
hsa-miR-19a-3p	RBMS1
hsa-miR-19a-3p	SH3BP4
hsa-miR-19a-3p	SLC12A2
hsa-miR-19a-3p	SNHG16
hsa-miR-19a-3p	SOX9
hsa-miR-19a-3p	STS
hsa-miR-19a-3p	TNF
hsa-miR-19a-3p	TNPO2
hsa-miR-19a-3p	ZIC3
hsa-miR-19a-3p	ZMYND11
hsa-miR-19a-3p	hsa-miR-130a-3p
hsa-miR-19a-3p	hsa-miR-130b-3p
hsa-miR-19a-3p	hsa-miR-153-3p
hsa-miR-19b-1-5p	CLIP1
hsa-miR-19b-1-5p	CSDE1
hsa-miR-19b-1-5p	FNBP4
hsa-miR-19b-1-5p	MALAT1
hsa-miR-19b-1-5p	MIR17HG
hsa-miR-19b-1-5p	RBMS1
hsa-miR-19b-1-5p	RPL30
hsa-miR-19b-1-5p	SOX9
hsa-miR-19b-1-5p	TNF
hsa-miR-19b-1-5p	TNPO2
hsa-miR-19b-1-5p	ZIC3
hsa-miR-19b-3p	ACO1
hsa-miR-19b-3p	ARL6
hsa-miR-19b-3p	CDCP1
hsa-miR-19b-3p	CHMP3
hsa-miR-19b-3p	CLIP1
hsa-miR-19b-3p	DMXL2
hsa-miR-19b-3p	ERCC4
hsa-miR-19b-3p	FNBP4
hsa-miR-19b-3p	GRB10
hsa-miR-19b-3p	GTF2H2

hsa-miR-19b-3p	NDE1
hsa-miR-19b-3p	PRMT2
hsa-miR-19b-3p	RBMS1
hsa-miR-19b-3p	SH3BP4
hsa-miR-19b-3p	SLC12A2
hsa-miR-19b-3p	SNHG16
hsa-miR-19b-3p	SOX9
hsa-miR-19b-3p	SRRM2
hsa-miR-19b-3p	STS
hsa-miR-19b-3p	TNF
hsa-miR-19b-3p	TNPO2
hsa-miR-19b-3p	ZMYND11
hsa-miR-200a-3p	AR
hsa-miR-200a-3p	ATF3
hsa-miR-200a-3p	BEND4
hsa-miR-200a-3p	CARM1
hsa-miR-200a-3p	CLIP1
hsa-miR-200a-3p	CSDE1
hsa-miR-200a-3p	DEPDC1B
hsa-miR-200a-3p	GRB10
hsa-miR-200a-3p	GTF2H2
hsa-miR-200a-3p	HNF4A
hsa-miR-200a-3p	IL10
hsa-miR-200a-3p	LTBP1
hsa-miR-200a-3p	NME1-NME2
hsa-miR-200a-3p	PPM1K
hsa-miR-200a-3p	RBM28
hsa-miR-200a-3p	RBMS1
hsa-miR-200a-3p	SH3BP4
hsa-miR-200a-3p	SLC12A2
hsa-miR-200a-3p	SOX9
hsa-miR-200a-3p	ZMYND11
hsa-miR-200a-3p	hsa-miR-107
hsa-miR-200a-3p	hsa-miR-141-3p
hsa-miR-200b-3p	ACO1
hsa-miR-200b-3p	AR
hsa-miR-200b-3p	ARHGEF10
hsa-miR-200b-3p	ARL6
hsa-miR-200b-3p	CARM1
hsa-miR-200b-3p	CHMP1B
hsa-miR-200b-3p	CLIP1
hsa-miR-200b-3p	DEPDC1B
hsa-miR-200b-3p	ERCC4

hsa-miR-200b-3p	FNBP4
hsa-miR-200b-3p	GRB10
hsa-miR-200b-3p	HNF4A
hsa-miR-200b-3p	HP1BP3
hsa-miR-200b-3p	IL6
hsa-miR-200b-3p	LTBP1
hsa-miR-200b-3p	MAGI1
hsa-miR-200b-3p	MALAT1
hsa-miR-200b-3p	PLCG1
hsa-miR-200b-3p	PTCH1
hsa-miR-200b-3p	RBMS1
hsa-miR-200b-3p	SLC12A2
hsa-miR-200b-3p	TIMP2
hsa-miR-200b-3p	TNPO2
hsa-miR-200b-3p	USP38
hsa-miR-200b-3p	ZMYND11
hsa-miR-200c-3p	AR
hsa-miR-200c-3p	ARHGEF10
hsa-miR-200c-3p	ARL6
hsa-miR-200c-3p	CARM1
hsa-miR-200c-3p	CHMP1B
hsa-miR-200c-3p	CLIP1
hsa-miR-200c-3p	DEPDC1B
hsa-miR-200c-3p	ERCC4
hsa-miR-200c-3p	FNBP4
hsa-miR-200c-3p	GRB10
hsa-miR-200c-3p	HNF4A
hsa-miR-200c-3p	HP1BP3
hsa-miR-200c-3p	LTBP1
hsa-miR-200c-3p	MAGI1
hsa-miR-200c-3p	MECP2
hsa-miR-200c-3p	PLCG1
hsa-miR-200c-3p	PTCH1
hsa-miR-200c-3p	SLC12A2
hsa-miR-200c-3p	TIMP2
hsa-miR-200c-3p	USP38
hsa-miR-203a-3p	ARHGEF10
hsa-miR-203a-3p	BEND4
hsa-miR-203a-3p	CDCP1
hsa-miR-203a-3p	CHMP3
hsa-miR-203a-3p	CLIP1
hsa-miR-203a-3p	CSDE1
hsa-miR-203a-3p	CXCL8

hsa-miR-203a-3p	DMXL2
hsa-miR-203a-3p	FZR1
hsa-miR-203a-3p	HNF4A
hsa-miR-203a-3p	HP1BP3
hsa-miR-203a-3p	IL10
hsa-miR-203a-3p	IL6
hsa-miR-203a-3p	INTS3
hsa-miR-203a-3p	LTBP1
hsa-miR-203a-3p	MAGI1
hsa-miR-203a-3p	MALAT1
hsa-miR-203a-3p	MECP2
hsa-miR-203a-3p	PCDH19
hsa-miR-203a-3p	PPM1K
hsa-miR-203a-3p	PTCH1
hsa-miR-203a-3p	RBM28
hsa-miR-203a-3p	RBMS1
hsa-miR-203a-3p	SH3BP4
hsa-miR-203a-3p	SLC12A2
hsa-miR-203a-3p	SNHG16
hsa-miR-203a-3p	SOX9
hsa-miR-203a-3p	SRPRA
hsa-miR-203a-3p	TIMP2
hsa-miR-203a-3p	TMTC2
hsa-miR-203a-3p	TNF
hsa-miR-203a-3p	ZMYND11
hsa-miR-203a-3p	ZNF318
hsa-miR-204-5p	BANCR
hsa-miR-204-5p	CHMP3
hsa-miR-204-5p	CLIP1
hsa-miR-204-5p	CSDE1
hsa-miR-204-5p	HP1BP3
hsa-miR-204-5p	IL10
hsa-miR-204-5p	INTS3
hsa-miR-204-5p	LTBP1
hsa-miR-204-5p	MAGI1
hsa-miR-204-5p	MBD1
hsa-miR-204-5p	MECP2
hsa-miR-204-5p	NARS2
hsa-miR-204-5p	NEAT1
hsa-miR-204-5p	PLAUR
hsa-miR-204-5p	PLCG1
hsa-miR-204-5p	RBMS1
hsa-miR-204-5p	SH3BP4

hsa-miR-204-5p	TMTC2
hsa-miR-204-5p	TPPP
hsa-miR-204-5p	TUG1
hsa-miR-205-5p	AR
hsa-miR-205-5p	CLIP1
hsa-miR-205-5p	CSDE1
hsa-miR-205-5p	CXCL8
hsa-miR-205-5p	DMXL2
hsa-miR-205-5p	ERCC4
hsa-miR-205-5p	HNF4A
hsa-miR-205-5p	HP1BP3
hsa-miR-205-5p	L3MBTL2
hsa-miR-205-5p	MAGI1
hsa-miR-205-5p	MALAT1
hsa-miR-205-5p	NARS2
hsa-miR-205-5p	PRMT2
hsa-miR-205-5p	PTCH1
hsa-miR-205-5p	STS
hsa-miR-205-5p	TMEM201
hsa-miR-205-5p	ZMYND11
hsa-miR-205-5p	hsa-miR-204-5p
hsa-miR-2054	CSDE1
hsa-miR-2054	HNF4A
hsa-miR-2054	SRPRA
hsa-miR-206	HP1BP3
hsa-miR-206	KHSRP
hsa-miR-206	NARS2
hsa-miR-206	PLCG1
hsa-miR-206	PLEKHA1
hsa-miR-206	PPP2R3A
hsa-miR-206	PRMT2
hsa-miR-206	SLC12A2
hsa-miR-206	SOX9
hsa-miR-206	STS
hsa-miR-206	TNPO2
hsa-miR-206	TPPP
hsa-miR-206	ZMYND11
hsa-miR-20a-3p	CSDE1
hsa-miR-20a-3p	FNBP4
hsa-miR-20a-3p	SRRM2
hsa-miR-20a-3p	TIMP2
hsa-miR-20a-5p	ARHGEF10
hsa-miR-20a-5p	CSDE1

hsa-miR-20a-5p	CXCL8
hsa-miR-20a-5p	FNBP4
hsa-miR-20a-5p	HNF4A
hsa-miR-20a-5p	HP1BP3
hsa-miR-20a-5p	IL6
hsa-miR-20a-5p	INTS3
hsa-miR-20a-5p	L3MBTL2
hsa-miR-20a-5p	MALAT1
hsa-miR-20a-5p	MECP2
hsa-miR-20a-5p	PPP2R3A
hsa-miR-20a-5p	RBMS1
hsa-miR-20a-5p	RPL30
hsa-miR-20a-5p	SENP5
hsa-miR-20a-5p	SH3BP4
hsa-miR-20a-5p	SNHG16
hsa-miR-20a-5p	SRRM2
hsa-miR-20a-5p	SSH1
hsa-miR-20a-5p	TIMP2
hsa-miR-20a-5p	TNF
hsa-miR-20a-5p	TPPP
hsa-miR-20a-5p	TUG1
hsa-miR-20a-5p	ZNF318
hsa-miR-20a-5p	ZNF354B
hsa-miR-20a-5p	hsa-miR-106a-5p
hsa-miR-20b-3p	CSDE1
hsa-miR-20b-3p	FNBP4
hsa-miR-20b-3p	PCDH19
hsa-miR-20b-3p	SLC12A2
hsa-miR-20b-3p	SOX9
hsa-miR-20b-3p	TIMP2
hsa-miR-20b-5p	ARHGEF10
hsa-miR-20b-5p	CSDE1
hsa-miR-20b-5p	CXCL8
hsa-miR-20b-5p	FNBP4
hsa-miR-20b-5p	HP1BP3
hsa-miR-20b-5p	IL6
hsa-miR-20b-5p	L3MBTL2
hsa-miR-20b-5p	MALAT1
hsa-miR-20b-5p	MECP2
hsa-miR-20b-5p	PPP2R3A
hsa-miR-20b-5p	RBMS1
hsa-miR-20b-5p	SENP5
hsa-miR-20b-5p	SH3BP4

hsa-miR-20b-5p	SNHG16
hsa-miR-20b-5p	SRRM2
hsa-miR-20b-5p	SSH1
hsa-miR-20b-5p	TIMP2
hsa-miR-20b-5p	TNF
hsa-miR-20b-5p	TPPP
hsa-miR-20b-5p	TUG1
hsa-miR-20b-5p	ZNF318
hsa-miR-20b-5p	ZNF354B
hsa-miR-21-5p	ACO1
hsa-miR-21-5p	AR
hsa-miR-21-5p	ARHGEF10
hsa-miR-21-5p	ARL6
hsa-miR-21-5p	HNF4A
hsa-miR-21-5p	HP1BP3
hsa-miR-21-5p	IL10
hsa-miR-21-5p	INTS3
hsa-miR-21-5p	LTBP1
hsa-miR-21-5p	MALAT1
hsa-miR-21-5p	NEAT1
hsa-miR-21-5p	RBMS1
hsa-miR-21-5p	TNF
hsa-miR-21-5p	TUG1
hsa-miR-21-5p	hsa-miR-132-3p
hsa-miR-211-5p	AR
hsa-miR-211-5p	CHMP3
hsa-miR-211-5p	CLIP1
hsa-miR-211-5p	HNF4A
hsa-miR-211-5p	HP1BP3
hsa-miR-211-5p	IL10
hsa-miR-211-5p	INTS3
hsa-miR-211-5p	LTBP1
hsa-miR-211-5p	MAGI1
hsa-miR-211-5p	MBD1
hsa-miR-211-5p	MECP2
hsa-miR-211-5p	PLCG1
hsa-miR-211-5p	RBMS1
hsa-miR-211-5p	TMTC2
hsa-miR-211-5p	TPPP
hsa-miR-212-3p	AR
hsa-miR-212-3p	ARHGEF10
hsa-miR-212-3p	CSDE1
hsa-miR-212-3p	FNBP4

hsa-miR-212-3p	GTF2H2
hsa-miR-212-3p	HNF4A
hsa-miR-212-3p	MAGI1
hsa-miR-212-3p	NARS2
hsa-miR-212-3p	PRPF31
hsa-miR-212-3p	PTCH1
hsa-miR-212-3p	RBMS1
hsa-miR-212-3p	SNHG16
hsa-miR-212-3p	USP38
hsa-miR-212-3p	hsa-miR-134-5p
hsa-miR-212-3p	hsa-miR-18a-5p
hsa-miR-212-3p	hsa-miR-18b-5p
hsa-miR-212-3p	hsa-miR-21-5p
hsa-miR-214-3p	ACO1
hsa-miR-214-3p	ARHGEF10
hsa-miR-214-3p	DMXL2
hsa-miR-214-3p	FENDRR
hsa-miR-214-3p	FNBP4
hsa-miR-214-3p	HNF4A
hsa-miR-214-3p	MALAT1
hsa-miR-214-3p	MIR17HG
hsa-miR-214-3p	NEAT1
hsa-miR-214-3p	NSRP1
hsa-miR-214-3p	PCDH19
hsa-miR-214-3p	PLAUR
hsa-miR-214-3p	POLR2A
hsa-miR-214-3p	PSMB5
hsa-miR-214-3p	SH3BP4
hsa-miR-214-3p	SLC12A2
hsa-miR-214-3p	SLC35C2
hsa-miR-214-3p	SNHG16
hsa-miR-214-3p	TIMP2
hsa-miR-214-3p	TMTC2
hsa-miR-214-3p	TUG1
hsa-miR-214-3p	hsa-let-7d-5p
hsa-miR-215-5p	C17orf58
hsa-miR-215-5p	ERCC4
hsa-miR-215-5p	HNF4A
hsa-miR-215-5p	HP1BP3
hsa-miR-215-5p	NDE1
hsa-miR-215-5p	PCDH19
hsa-miR-215-5p	PLEKHA1
hsa-miR-215-5p	SH3BP4

hsa-miR-215-5p	TMTC2
hsa-miR-215-5p	ZMYND11
hsa-miR-215-5p	hsa-miR-192-5p
hsa-miR-216a-3p	DEPDC1B
hsa-miR-216a-3p	DMXL2
hsa-miR-216a-3p	SRPRA
hsa-miR-216a-3p	TNPO2
hsa-miR-216a-3p	TPPP
hsa-miR-218-5p	ARL6
hsa-miR-218-5p	BEND4
hsa-miR-218-5p	CCDC89
hsa-miR-218-5p	CDCP1
hsa-miR-218-5p	CSDE1
hsa-miR-218-5p	FNBP4
hsa-miR-218-5p	HP1BP3
hsa-miR-218-5p	MALAT1
hsa-miR-218-5p	MECP2
hsa-miR-218-5p	NEAT1
hsa-miR-218-5p	PLCG1
hsa-miR-218-5p	POLR2A
hsa-miR-218-5p	PTCH1
hsa-miR-218-5p	SH3BP4
hsa-miR-218-5p	SLC12A2
hsa-miR-218-5p	SLC35C2
hsa-miR-218-5p	SSH1
hsa-miR-218-5p	TIMP2
hsa-miR-218-5p	hsa-miR-107
hsa-miR-221-3p	AR
hsa-miR-221-3p	ARHGEF10
hsa-miR-221-3p	ATF3
hsa-miR-221-3p	CHMP3
hsa-miR-221-3p	CLIP1
hsa-miR-221-3p	CSDE1
hsa-miR-221-3p	ERCC4
hsa-miR-221-3p	GRB10
hsa-miR-221-3p	GTF2H2
hsa-miR-221-3p	KHSRP
hsa-miR-221-3p	MAGI1
hsa-miR-221-3p	MECP2
hsa-miR-221-3p	PSMB5
hsa-miR-221-3p	RBMS1
hsa-miR-221-3p	SH3BP4
hsa-miR-221-3p	TNF

hsa-miR-221-3p	TNPO2
hsa-miR-221-3p	TUG1
hsa-miR-221-3p	USP38
hsa-miR-221-3p	ZMYND11
hsa-miR-221-3p	hsa-miR-19a-3p
hsa-miR-222-3p	AR
hsa-miR-222-3p	ARHGEF10
hsa-miR-222-3p	ATF3
hsa-miR-222-3p	CHMP3
hsa-miR-222-3p	CLIP1
hsa-miR-222-3p	CSDE1
hsa-miR-222-3p	DEPDC1B
hsa-miR-222-3p	ERCC4
hsa-miR-222-3p	GRB10
hsa-miR-222-3p	GTF2H2
hsa-miR-222-3p	MAGI1
hsa-miR-222-3p	MECP2
hsa-miR-222-3p	MROH1
hsa-miR-222-3p	PLCG1
hsa-miR-222-3p	PSMB5
hsa-miR-222-3p	RBMS1
hsa-miR-222-3p	SENP5
hsa-miR-222-3p	SH3BP4
hsa-miR-222-3p	SRRM2
hsa-miR-222-3p	TIMP2
hsa-miR-222-3p	ZMYND11
hsa-miR-2355-5p	HNF4A
hsa-miR-2355-5p	MBD1
hsa-miR-2355-5p	SLC12A2
hsa-miR-2355-5p	TNPO2
hsa-miR-23a-3p	ACO1
hsa-miR-23a-3p	C17orf58
hsa-miR-23a-3p	CSDE1
hsa-miR-23a-3p	CXCL8
hsa-miR-23a-3p	FNBP4
hsa-miR-23a-3p	HNF4A
hsa-miR-23a-3p	HP1BP3
hsa-miR-23a-3p	IL6
hsa-miR-23a-3p	KHSRP
hsa-miR-23a-3p	MAGI1
hsa-miR-23a-3p	NDUFV3
hsa-miR-23a-3p	NEAT1
hsa-miR-23a-3p	PCDH19

hsa-miR-23a-3p	PLEKHA1
hsa-miR-23a-3p	PPM1K
hsa-miR-23a-3p	PRMT2
hsa-miR-23a-3p	RNA5-8SP6
hsa-miR-23a-3p	SH3BP4
hsa-miR-23a-3p	SLC12A2
hsa-miR-23a-3p	SRPRA
hsa-miR-23a-3p	STS
hsa-miR-23a-3p	ZMYND11
hsa-miR-23b-3p	ACO1
hsa-miR-23b-3p	ATF3
hsa-miR-23b-3p	C17orf58
hsa-miR-23b-3p	CSDE1
hsa-miR-23b-3p	FNBP4
hsa-miR-23b-3p	HNF4A
hsa-miR-23b-3p	HP1BP3
hsa-miR-23b-3p	IL6
hsa-miR-23b-3p	KHSRP
hsa-miR-23b-3p	MAGI1
hsa-miR-23b-3p	MALAT1
hsa-miR-23b-3p	NDUFV3
hsa-miR-23b-3p	PCDH19
hsa-miR-23b-3p	PLAUR
hsa-miR-23b-3p	PLEKHA1
hsa-miR-23b-3p	PPM1K
hsa-miR-23b-3p	PRMT2
hsa-miR-23b-3p	RNA5-8SP6
hsa-miR-23b-3p	SH3BP4
hsa-miR-23b-3p	SLC12A2
hsa-miR-23b-3p	SRPRA
hsa-miR-23b-3p	STS
hsa-miR-23b-3p	ZMYND11
hsa-miR-23c	HNF4A
hsa-miR-23c	KHSRP
hsa-miR-23c	MAGI1
hsa-miR-23c	MALAT1
hsa-miR-23c	NDUFV3
hsa-miR-23c	PCDH19
hsa-miR-23c	PPM1K
hsa-miR-23c	RNA5-8SP6
hsa-miR-23c	SLC12A2
hsa-miR-23c	STS
hsa-miR-24-1-5p	IL6

hsa-miR-24-1-5p	SRPRA
hsa-miR-24-1-5p	ZIC3
hsa-miR-24-2-5p	SRPRA
hsa-miR-24-2-5p	ZIC3
hsa-miR-24-3p	ACO1
hsa-miR-24-3p	ATF3
hsa-miR-24-3p	CDCP1
hsa-miR-24-3p	CLIP1
hsa-miR-24-3p	DMXL2
hsa-miR-24-3p	KHSRP
hsa-miR-24-3p	L3MBTL2
hsa-miR-24-3p	MAGI1
hsa-miR-24-3p	RABAC1
hsa-miR-24-3p	SRPRA
hsa-miR-24-3p	TNF
hsa-miR-24-3p	TNPO2
hsa-miR-24-3p	TUG1
hsa-miR-26b-5p	ATF3
hsa-miR-26b-5p	BEND4
hsa-miR-26b-5p	BNC1
hsa-miR-26b-5p	C17orf58
hsa-miR-26b-5p	CARM1
hsa-miR-26b-5p	CHMP3
hsa-miR-26b-5p	DEPDC1B
hsa-miR-26b-5p	DMXL2
hsa-miR-26b-5p	FAM214B
hsa-miR-26b-5p	GRB10
hsa-miR-26b-5p	HNF4A
hsa-miR-26b-5p	IL10
hsa-miR-26b-5p	IL6
hsa-miR-26b-5p	LTBP1
hsa-miR-26b-5p	MALAT1
hsa-miR-26b-5p	MPPE1
hsa-miR-26b-5p	NARS2
hsa-miR-26b-5p	PCDH19
hsa-miR-26b-5p	PLEKHA1
hsa-miR-26b-5p	PPP2R3A
hsa-miR-26b-5p	PRRG3
hsa-miR-26b-5p	PTCH1
hsa-miR-26b-5p	RBMS1
hsa-miR-26b-5p	SENP5
hsa-miR-26b-5p	SLC12A2
hsa-miR-26b-5p	SRPRA

hsa-miR-26b-5p	SRRM2
hsa-miR-26b-5p	SSH1
hsa-miR-26b-5p	TPPP
hsa-miR-26b-5p	TUG1
hsa-miR-27a-3p	ACO1
hsa-miR-27a-3p	ATF3
hsa-miR-27a-3p	BEND4
hsa-miR-27a-3p	CARM1
hsa-miR-27a-3p	DEPDC1B
hsa-miR-27a-3p	DMXL2
hsa-miR-27a-3p	GRB10
hsa-miR-27a-3p	GTF2H2
hsa-miR-27a-3p	HNF4A
hsa-miR-27a-3p	IL10
hsa-miR-27a-3p	KHSRP
hsa-miR-27a-3p	L3MBTL2
hsa-miR-27a-3p	LTBP1
hsa-miR-27a-3p	PPM1K
hsa-miR-27a-3p	SENP5
hsa-miR-27a-3p	SLC12A2
hsa-miR-27a-3p	SNHG16
hsa-miR-27a-3p	SOX9
hsa-miR-27a-3p	SRRM2
hsa-miR-27a-3p	SSH1
hsa-miR-27a-3p	TMTC2
hsa-miR-27a-3p	ZMYND11
hsa-miR-27a-3p	ZNF318
hsa-miR-27b-3p	ACO1
hsa-miR-27b-3p	ATF3
hsa-miR-27b-3p	BEND4
hsa-miR-27b-3p	CARM1
hsa-miR-27b-3p	DEPDC1B
hsa-miR-27b-3p	FNBP4
hsa-miR-27b-3p	GRB10
hsa-miR-27b-3p	GTF2H2
hsa-miR-27b-3p	HNF4A
hsa-miR-27b-3p	IL10
hsa-miR-27b-3p	KHSRP
hsa-miR-27b-3p	L3MBTL2
hsa-miR-27b-3p	LTBP1
hsa-miR-27b-3p	PPM1K
hsa-miR-27b-3p	PSMB5
hsa-miR-27b-3p	SENP5

hsa-miR-27b-3p	SLC12A2
hsa-miR-27b-3p	SNHG16
hsa-miR-27b-3p	SOX9
hsa-miR-27b-3p	SRPRA
hsa-miR-27b-3p	SRRM2
hsa-miR-27b-3p	SSH1
hsa-miR-27b-3p	TMTC2
hsa-miR-27b-3p	ZMYND11
hsa-miR-27b-3p	ZNF318
hsa-miR-296-3p	AR
hsa-miR-296-3p	FZR1
hsa-miR-296-3p	IL10
hsa-miR-296-3p	MBD1
hsa-miR-296-3p	ND2
hsa-miR-296-3p	ND4
hsa-miR-296-3p	ND5
hsa-miR-296-3p	NDE1
hsa-miR-296-3p	PTCH1
hsa-miR-296-3p	SENP5
hsa-miR-296-3p	SH3BP4
hsa-miR-296-3p	SRPRA
hsa-miR-296-3p	hsa-miR-150-5p
hsa-miR-29a-3p	ACO1
hsa-miR-29a-3p	ARHGEF10
hsa-miR-29a-3p	CSDE1
hsa-miR-29a-3p	FNBP4
hsa-miR-29a-3p	HNF4A
hsa-miR-29a-3p	PLEKHA1
hsa-miR-29a-3p	PRMT2
hsa-miR-29a-3p	RBMS1
hsa-miR-29a-3p	SLC12A2
hsa-miR-29a-3p	TMEM201
hsa-miR-29a-3p	ZNF318
hsa-miR-29b-2-5p	ACO1
hsa-miR-29b-2-5p	CSDE1
hsa-miR-29b-2-5p	FZR1
hsa-miR-29b-2-5p	PRMT2
hsa-miR-29b-2-5p	SRPRA
hsa-miR-29b-2-5p	ZNF318
hsa-miR-29b-3p	ARHGEF10
hsa-miR-29b-3p	CSDE1
hsa-miR-29b-3p	HP1BP3
hsa-miR-29b-3p	PLEKHA1

hsa-miR-29b-3p	SLC12A2
hsa-miR-29b-3p	SOX9
hsa-miR-29b-3p	SSH1
hsa-miR-29b-3p	TMEM201
hsa-miR-29b-3p	TNF
hsa-miR-29b-3p	TUG1
hsa-miR-29c-3p	ACO1
hsa-miR-29c-3p	AR
hsa-miR-29c-3p	ARHGEF10
hsa-miR-29c-3p	CSDE1
hsa-miR-29c-3p	HNF4A
hsa-miR-29c-3p	PLEKHA1
hsa-miR-29c-3p	PRMT2
hsa-miR-29c-3p	SLC12A2
hsa-miR-29c-3p	TMEM201
hsa-miR-29c-3p	TUG1
hsa-miR-29c-3p	ZNF318
hsa-miR-301a-3p	AR
hsa-miR-301a-3p	ATF3
hsa-miR-301a-3p	CHMP3
hsa-miR-301a-3p	CLIP1
hsa-miR-301a-3p	CSDE1
hsa-miR-301a-3p	ERCC4
hsa-miR-301a-3p	FNBP4
hsa-miR-301a-3p	GRB10
hsa-miR-301a-3p	HNF4A
hsa-miR-301a-3p	LTBP1
hsa-miR-301a-3p	MECP2
hsa-miR-301a-3p	MIR17HG
hsa-miR-301a-3p	PRPF31
hsa-miR-301a-3p	PSMB5
hsa-miR-301a-3p	RNVU1-7
hsa-miR-301a-3p	SH3BP4
hsa-miR-301a-3p	SLC12A2
hsa-miR-301a-3p	TENM1
hsa-miR-301a-3p	TIMP2
hsa-miR-301a-3p	TNF
hsa-miR-301a-3p	ZMYND11
hsa-miR-301a-3p	ZNF354B
hsa-miR-301a-3p	hsa-miR-130a-3p
hsa-miR-301a-3p	hsa-miR-130b-3p
hsa-miR-301a-3p	hsa-miR-19a-3p
hsa-miR-301b-3p	AR

hsa-miR-301b-3p	ATF3
hsa-miR-301b-3p	CHMP3
hsa-miR-301b-3p	CLIP1
hsa-miR-301b-3p	CSDE1
hsa-miR-301b-3p	ERCC4
hsa-miR-301b-3p	FNBP4
hsa-miR-301b-3p	GRB10
hsa-miR-301b-3p	HNF4A
hsa-miR-301b-3p	HP1BP3
hsa-miR-301b-3p	LTBP1
hsa-miR-301b-3p	MECP2
hsa-miR-301b-3p	MIR17HG
hsa-miR-301b-3p	PSMB5
hsa-miR-301b-3p	RNVU1-7
hsa-miR-301b-3p	SH3BP4
hsa-miR-301b-3p	SLC12A2
hsa-miR-301b-3p	TENM1
hsa-miR-301b-3p	TIMP2
hsa-miR-301b-3p	TNF
hsa-miR-301b-3p	ZMYND11
hsa-miR-301b-3p	ZNF354B
hsa-miR-301b-3p	hsa-miR-130a-3p
hsa-miR-301b-3p	hsa-miR-130b-3p
hsa-miR-301b-3p	hsa-miR-19a-3p
hsa-miR-301b-3p	hsa-miR-301a-3p
hsa-miR-30a-5p	AR
hsa-miR-30a-5p	ARL6
hsa-miR-30a-5p	CDCP1
hsa-miR-30a-5p	CSDE1
hsa-miR-30a-5p	DEPDC1B
hsa-miR-30a-5p	DMXL2
hsa-miR-30a-5p	ERCC4
hsa-miR-30a-5p	GRB10
hsa-miR-30a-5p	HNF4A
hsa-miR-30a-5p	HP1BP3
hsa-miR-30a-5p	MECP2
hsa-miR-30a-5p	NARS2
hsa-miR-30a-5p	NSRP1
hsa-miR-30a-5p	PCDH19
hsa-miR-30a-5p	PLCG1
hsa-miR-30a-5p	POLR2A
hsa-miR-30a-5p	PTCH1
hsa-miR-30a-5p	RBMS1

hsa-miR-30a-5p	SENP5
hsa-miR-30a-5p	SLC35C2
hsa-miR-30a-5p	SOX9
hsa-miR-30a-5p	SRPRA
hsa-miR-30a-5p	SSH1
hsa-miR-30a-5p	STS
hsa-miR-30a-5p	TIMP2
hsa-miR-30a-5p	ZMYND11
hsa-miR-30a-5p	ZNF318
hsa-miR-30b-3p	CHMP1B
hsa-miR-30b-3p	CSDE1
hsa-miR-30b-3p	FZR1
hsa-miR-30b-3p	KHSRP
hsa-miR-30b-3p	PCDH19
hsa-miR-30b-3p	PLCG1
hsa-miR-30b-3p	PLEKHA1
hsa-miR-30b-3p	POLR2A
hsa-miR-30b-3p	SOX9
hsa-miR-30b-3p	SRPRA
hsa-miR-30b-3p	ZNF318
hsa-miR-30b-5p	AR
hsa-miR-30b-5p	ARL6
hsa-miR-30b-5p	CSDE1
hsa-miR-30b-5p	DEPDC1B
hsa-miR-30b-5p	ERCC4
hsa-miR-30b-5p	GRB10
hsa-miR-30b-5p	HNF4A
hsa-miR-30b-5p	HP1BP3
hsa-miR-30b-5p	MECP2
hsa-miR-30b-5p	NARS2
hsa-miR-30b-5p	PCDH19
hsa-miR-30b-5p	PLCG1
hsa-miR-30b-5p	POLR2A
hsa-miR-30b-5p	PTCH1
hsa-miR-30b-5p	SENP5
hsa-miR-30b-5p	SLC35C2
hsa-miR-30b-5p	SRPRA
hsa-miR-30b-5p	SSH1
hsa-miR-30b-5p	STS
hsa-miR-30b-5p	TIMP2
hsa-miR-30b-5p	ZMYND11
hsa-miR-30b-5p	ZNF318
hsa-miR-30b-5p	hsa-miR-30a-5p

hsa-miR-30c-5p	AR
hsa-miR-30c-5p	ARL6
hsa-miR-30c-5p	CSDE1
hsa-miR-30c-5p	DEPDC1B
hsa-miR-30c-5p	ERCC4
hsa-miR-30c-5p	GRB10
hsa-miR-30c-5p	HP1BP3
hsa-miR-30c-5p	MALAT1
hsa-miR-30c-5p	MECP2
hsa-miR-30c-5p	NARS2
hsa-miR-30c-5p	PCDH19
hsa-miR-30c-5p	PLCG1
hsa-miR-30c-5p	POLR2A
hsa-miR-30c-5p	PTCH1
hsa-miR-30c-5p	SENP5
hsa-miR-30c-5p	SLC35C2
hsa-miR-30c-5p	SRPRA
hsa-miR-30c-5p	SSH1
hsa-miR-30c-5p	STS
hsa-miR-30c-5p	TIMP2
hsa-miR-30c-5p	ZMYND11
hsa-miR-30c-5p	ZNF318
hsa-miR-30c-5p	hsa-miR-30b-5p
hsa-miR-30d-5p	AR
hsa-miR-30d-5p	ARL6
hsa-miR-30d-5p	CHMP3
hsa-miR-30d-5p	CSDE1
hsa-miR-30d-5p	DEPDC1B
hsa-miR-30d-5p	ERCC4
hsa-miR-30d-5p	GRB10
hsa-miR-30d-5p	HNF4A
hsa-miR-30d-5p	HP1BP3
hsa-miR-30d-5p	MALAT1
hsa-miR-30d-5p	MECP2
hsa-miR-30d-5p	NARS2
hsa-miR-30d-5p	PCDH19
hsa-miR-30d-5p	PLCG1
hsa-miR-30d-5p	POLR2A
hsa-miR-30d-5p	PTCH1
hsa-miR-30d-5p	SAMD4B
hsa-miR-30d-5p	SENP5
hsa-miR-30d-5p	SLC35C2
hsa-miR-30d-5p	SRPRA

hsa-miR-30d-5p	SSH1
hsa-miR-30d-5p	STS
hsa-miR-30d-5p	TIMP2
hsa-miR-30d-5p	ZMYND11
hsa-miR-30d-5p	ZNF318
hsa-miR-30d-5p	hsa-miR-16-5p
hsa-miR-30d-5p	hsa-miR-30b-5p
hsa-miR-30e-5p	AR
hsa-miR-30e-5p	ARL6
hsa-miR-30e-5p	CSDE1
hsa-miR-30e-5p	DEPDC1B
hsa-miR-30e-5p	ERCC4
hsa-miR-30e-5p	GRB10
hsa-miR-30e-5p	HNF4A
hsa-miR-30e-5p	HP1BP3
hsa-miR-30e-5p	MALAT1
hsa-miR-30e-5p	MBD1
hsa-miR-30e-5p	MECP2
hsa-miR-30e-5p	NARS2
hsa-miR-30e-5p	NSRP1
hsa-miR-30e-5p	PCDH19
hsa-miR-30e-5p	PLCG1
hsa-miR-30e-5p	POLR2A
hsa-miR-30e-5p	PSMB5
hsa-miR-30e-5p	PTCH1
hsa-miR-30e-5p	SENP5
hsa-miR-30e-5p	SLC35C2
hsa-miR-30e-5p	SRPRA
hsa-miR-30e-5p	SSH1
hsa-miR-30e-5p	STS
hsa-miR-30e-5p	TIMP2
hsa-miR-30e-5p	ZMYND11
hsa-miR-30e-5p	ZNF318
hsa-miR-30e-5p	hsa-miR-30a-5p
hsa-miR-30e-5p	hsa-miR-30b-5p
hsa-miR-30e-5p	hsa-miR-30c-5p
hsa-miR-30e-5p	hsa-miR-30d-5p
hsa-miR-31-3p	KHSRP
hsa-miR-31-3p	PPM1K
hsa-miR-31-3p	RBMS1
hsa-miR-31-3p	SSH1
hsa-miR-31-5p	AR
hsa-miR-31-5p	ARHGEF10

hsa-miR-31-5p	CLIP1
hsa-miR-31-5p	CSDE1
hsa-miR-31-5p	GRB10
hsa-miR-31-5p	HNF4A
hsa-miR-31-5p	KHSRP
hsa-miR-31-5p	PLEKHA1
hsa-miR-31-5p	POLR2A
hsa-miR-31-5p	RBMS1
hsa-miR-31-5p	SH3BP4
hsa-miR-31-5p	SNHG16
hsa-miR-31-5p	SRPRA
hsa-miR-31-5p	SRRM2
hsa-miR-31-5p	SSH1
hsa-miR-31-5p	TINCR
hsa-miR-31-5p	hsa-miR-190a-5p
hsa-miR-3116	ACO1
hsa-miR-3116	ARHGEF10
hsa-miR-3116	CLIP1
hsa-miR-3116	MALAT1
hsa-miR-3116	NDE1
hsa-miR-3116	NDUFV3
hsa-miR-3118	FNBP4
hsa-miR-3118	PLEKHA1
hsa-miR-3118	hsa-miR-212-3p
hsa-miR-3120-3p	DEPDC1B
hsa-miR-3120-3p	HNF4A
hsa-miR-3120-3p	MIR17HG
hsa-miR-3120-3p	RBMS1
hsa-miR-3120-3p	TUG1
hsa-miR-3120-3p	ZMYND11
hsa-miR-3133	AR
hsa-miR-3133	BNC1
hsa-miR-3133	HNF4A
hsa-miR-3133	KHSRP
hsa-miR-3133	RBMS1
hsa-miR-3133	SNHG16
hsa-miR-3133	SRRM2
hsa-miR-3133	TIMP2
hsa-miR-3133	TNPO2
hsa-miR-3158-3p	LTBP1
hsa-miR-3158-3p	TIMP2
hsa-miR-3163	AR
hsa-miR-3163	ATF3

hsa-miR-3163	BEND4
hsa-miR-3163	C17orf58
hsa-miR-3163	CLIP1
hsa-miR-3163	CSDE1
hsa-miR-3163	FNBP4
hsa-miR-3163	HNF4A
hsa-miR-3163	HP1BP3
hsa-miR-3163	PLEKHA1
hsa-miR-3163	POLR2A
hsa-miR-3163	PPM1K
hsa-miR-3163	RBMS1
hsa-miR-3163	SAMD4B
hsa-miR-3163	SNHG16
hsa-miR-3163	SOX9
hsa-miR-3183	PSMB5
hsa-miR-3183	hsa-miR-134-5p
hsa-miR-3183	hsa-miR-3118
hsa-miR-3198	SRRM2
hsa-miR-3199	CARM1
hsa-miR-3199	GTF2H2
hsa-miR-3199	SNHG16
hsa-miR-3199	TNPO2
hsa-miR-323b-5p	CHMP3
hsa-miR-323b-5p	CSDE1
hsa-miR-323b-5p	GRB10
hsa-miR-323b-5p	HID1
hsa-miR-323b-5p	MIR17HG
hsa-miR-323b-5p	PLEKHA1
hsa-miR-323b-5p	SNHG16
hsa-miR-323b-5p	SOX9
hsa-miR-323b-5p	TMEM201
hsa-miR-324-3p	ACO1
hsa-miR-324-3p	CARM1
hsa-miR-324-3p	GTF2H2
hsa-miR-324-3p	MAGI1
hsa-miR-324-3p	MBD1
hsa-miR-324-3p	MIR17HG
hsa-miR-324-3p	NDUFV3
hsa-miR-324-3p	NSRP1
hsa-miR-324-3p	PCDH19
hsa-miR-324-3p	SRRM2
hsa-miR-324-3p	TPPP
hsa-miR-325	CHMP3

hsa-miR-325	HNF4A
hsa-miR-325	PSMB5
hsa-miR-325	RBMS1
hsa-miR-325	SOX9
hsa-miR-325	STS
hsa-miR-325	TNPO2
hsa-miR-325	hsa-miR-128-3p
hsa-miR-326	AR
hsa-miR-326	CSDE1
hsa-miR-326	EML2
hsa-miR-326	HNF4A
hsa-miR-326	INTS3
hsa-miR-326	MECP2
hsa-miR-326	SAMD4B
hsa-miR-326	SH3BP4
hsa-miR-326	SNORA73B
hsa-miR-326	SRPRA
hsa-miR-326	STS
hsa-miR-326	TIMP2
hsa-miR-326	TNPO2
hsa-miR-326	TUG1
hsa-miR-326	ZMYND11
hsa-miR-328-3p	CDCP1
hsa-miR-328-3p	GRB10
hsa-miR-328-3p	HNF4A
hsa-miR-328-3p	MAGI1
hsa-miR-328-3p	MECP2
hsa-miR-328-3p	NDUFV3
hsa-miR-328-3p	PCDH19
hsa-miR-328-3p	PLEKHA1
hsa-miR-328-3p	SH3BP4
hsa-miR-328-3p	TMTC2
hsa-miR-328-3p	TUG1
hsa-miR-328-3p	hsa-miR-184
hsa-miR-330-3p	ACO1
hsa-miR-330-3p	CLIP1
hsa-miR-330-3p	HNF4A
hsa-miR-330-3p	MALAT1
hsa-miR-330-3p	MIR17HG
hsa-miR-330-3p	PCDH19
hsa-miR-330-3p	PPM1K
hsa-miR-330-3p	SLC12A2
hsa-miR-330-3p	SRPRA

hsa-miR-330-3p	TNPO2
hsa-miR-330-3p	ZNF318
hsa-miR-330-3p	hsa-miR-218-5p
hsa-miR-330-5p	AR
hsa-miR-330-5p	INTS3
hsa-miR-330-5p	MALAT1
hsa-miR-330-5p	SAMD4B
hsa-miR-330-5p	SH3BP4
hsa-miR-330-5p	SNORA73B
hsa-miR-330-5p	SRPRA
hsa-miR-330-5p	STS
hsa-miR-330-5p	TIMP2
hsa-miR-330-5p	ZMYND11
hsa-miR-335-5p	AR
hsa-miR-335-5p	BNC1
hsa-miR-335-5p	CDCP1
hsa-miR-335-5p	CXCL8
hsa-miR-335-5p	FAM214B
hsa-miR-335-5p	FNBP4
hsa-miR-335-5p	GRB10
hsa-miR-335-5p	HID1
hsa-miR-335-5p	HNF4A
hsa-miR-335-5p	IL6
hsa-miR-335-5p	KHSRP
hsa-miR-335-5p	LTBP1
hsa-miR-335-5p	MAGI1
hsa-miR-335-5p	MROH1
hsa-miR-335-5p	PLAUR
hsa-miR-335-5p	PLEKHA1
hsa-miR-335-5p	PPP2R3A
hsa-miR-335-5p	SLC12A2
hsa-miR-335-5p	SOX9
hsa-miR-335-5p	SRPRA
hsa-miR-335-5p	TEX101
hsa-miR-335-5p	TMTC2
hsa-miR-335-5p	TNF
hsa-miR-335-5p	TUG1
hsa-miR-335-5p	hsa-miR-143-3p
hsa-miR-335-5p	hsa-miR-192-5p
hsa-miR-339-3p	SNHG16
hsa-miR-339-5p	ATF3
hsa-miR-339-5p	CHMP1B
hsa-miR-339-5p	HNF4A

hsa-miR-339-5p	INTS3
hsa-miR-339-5p	LTBP1
hsa-miR-339-5p	MECP2
hsa-miR-339-5p	NEAT1
hsa-miR-339-5p	SNHG16
hsa-miR-33a-3p	CSDE1
hsa-miR-33a-3p	DMXL2
hsa-miR-33a-3p	HP1BP3
hsa-miR-33a-3p	RPL30
hsa-miR-33a-3p	SENP5
hsa-miR-33a-3p	SLC12A2
hsa-miR-33a-3p	SOX9
hsa-miR-33a-3p	ZMYND11
hsa-miR-33a-5p	BEND4
hsa-miR-33a-5p	CHMP1B
hsa-miR-33a-5p	CSDE1
hsa-miR-33a-5p	DEPDC1B
hsa-miR-33a-5p	DMXL2
hsa-miR-33a-5p	ERCC4
hsa-miR-33a-5p	HNF4A
hsa-miR-33a-5p	MBD1
hsa-miR-33a-5p	SLC12A2
hsa-miR-33a-5p	SOX9
hsa-miR-33a-5p	SRRM2
hsa-miR-33a-5p	STS
hsa-miR-33a-5p	USP38
hsa-miR-33a-5p	hsa-miR-17-5p
hsa-miR-33a-5p	hsa-miR-20a-5p
hsa-miR-342-3p	AR
hsa-miR-342-3p	ATF3
hsa-miR-342-3p	BEND4
hsa-miR-342-3p	CHMP1B
hsa-miR-342-3p	CSDE1
hsa-miR-342-3p	DMXL2
hsa-miR-342-3p	GRB10
hsa-miR-342-3p	HNF4A
hsa-miR-342-3p	L3MBTL2
hsa-miR-342-3p	NEAT1
hsa-miR-342-3p	POLR2A
hsa-miR-342-3p	PPM1K
hsa-miR-342-3p	SENP5
hsa-miR-342-3p	SNHG16
hsa-miR-342-3p	SOX9

hsa-miR-342-3p	TMEM201
hsa-miR-342-3p	TMTC2
hsa-miR-346	ACO1
hsa-miR-346	CARM1
hsa-miR-346	HNF4A
hsa-miR-346	MIR17HG
hsa-miR-346	RPL30
hsa-miR-346	hsa-miR-18a-5p
hsa-miR-34a-5p	AR
hsa-miR-34a-5p	ARL6
hsa-miR-34a-5p	ATF3
hsa-miR-34a-5p	BEND4
hsa-miR-34a-5p	GRB10
hsa-miR-34a-5p	GTF2F1
hsa-miR-34a-5p	HP1BP3
hsa-miR-34a-5p	IL10
hsa-miR-34a-5p	LTBP1
hsa-miR-34a-5p	MAGI1
hsa-miR-34a-5p	MALAT1
hsa-miR-34a-5p	MECP2
hsa-miR-34a-5p	NEAT1
hsa-miR-34a-5p	PLCG1
hsa-miR-34a-5p	PLEKHA1
hsa-miR-34a-5p	PPP2R3A
hsa-miR-34a-5p	PSD4
hsa-miR-34a-5p	SLC12A2
hsa-miR-34a-5p	SRPRA
hsa-miR-34a-5p	STS
hsa-miR-34a-5p	TMEM201
hsa-miR-34a-5p	TNF
hsa-miR-34a-5p	TPPP
hsa-miR-34a-5p	TUG1
hsa-miR-34a-5p	ZMYND11
hsa-miR-34a-5p	ZNF318
hsa-miR-34a-5p	hsa-let-7g-5p
hsa-miR-34a-5p	hsa-miR-1229-3p
hsa-miR-34a-5p	hsa-miR-1271-5p
hsa-miR-34a-5p	hsa-miR-144-3p
hsa-miR-34a-5p	hsa-miR-20a-5p
hsa-miR-34a-5p	hsa-miR-330-3p
hsa-miR-34b-3p	AR
hsa-miR-34b-3p	KHSRP
hsa-miR-34b-3p	MALAT1

hsa-miR-34b-3p	POLR2A
hsa-miR-34b-3p	RABAC1
hsa-miR-34b-3p	RBM28
hsa-miR-34b-3p	SNHG16
hsa-miR-34b-3p	SRPRA
hsa-miR-34b-3p	TMTC2
hsa-miR-34b-3p	TUG1
hsa-miR-34c-3p	CHMP3
hsa-miR-34c-3p	GTF2F1
hsa-miR-34c-3p	INTS3
hsa-miR-34c-5p	AR
hsa-miR-34c-5p	ARHGEF10
hsa-miR-34c-5p	ARL6
hsa-miR-34c-5p	ATF3
hsa-miR-34c-5p	BEND4
hsa-miR-34c-5p	CARM1
hsa-miR-34c-5p	GRB10
hsa-miR-34c-5p	GTF2F1
hsa-miR-34c-5p	HNF4A
hsa-miR-34c-5p	INTS3
hsa-miR-34c-5p	MECP2
hsa-miR-34c-5p	NEAT1
hsa-miR-34c-5p	PLEKHA1
hsa-miR-34c-5p	PPP2R3A
hsa-miR-34c-5p	PSD4
hsa-miR-34c-5p	PTCH1
hsa-miR-34c-5p	SLC12A2
hsa-miR-34c-5p	SRPRA
hsa-miR-34c-5p	TMEM201
hsa-miR-34c-5p	TPPP
hsa-miR-34c-5p	TUG1
hsa-miR-34c-5p	ZMYND11
hsa-miR-34c-5p	hsa-let-7g-5p
hsa-miR-34c-5p	hsa-miR-1229-3p
hsa-miR-34c-5p	hsa-miR-330-3p
hsa-miR-34c-5p	hsa-miR-335-5p
hsa-miR-3529-3p	TIMP2
hsa-miR-361-3p	PSMB5
hsa-miR-361-3p	RBMS1
hsa-miR-361-3p	SLC12A2
hsa-miR-361-3p	SRRM2
hsa-miR-361-3p	ZNF354B
hsa-miR-363-3p	ARHGEF10

hsa-miR-363-3p	CARM1
hsa-miR-363-3p	CHMP3
hsa-miR-363-3p	CXCL8
hsa-miR-363-3p	DMXL2
hsa-miR-363-3p	FNBP4
hsa-miR-363-3p	FZR1
hsa-miR-363-3p	HP1BP3
hsa-miR-363-3p	MALAT1
hsa-miR-363-3p	MECP2
hsa-miR-363-3p	PLEKHA1
hsa-miR-363-3p	RBM28
hsa-miR-363-3p	SH3BP4
hsa-miR-363-3p	SLC12A2
hsa-miR-363-3p	SRPRA
hsa-miR-363-3p	STS
hsa-miR-363-3p	TPPP
hsa-miR-363-3p	ZNF354B
hsa-miR-363-3p	hsa-miR-130a-3p
hsa-miR-363-3p	hsa-miR-130b-3p
hsa-miR-363-3p	hsa-miR-301a-3p
hsa-miR-363-3p	hsa-miR-301b-3p
hsa-miR-363-3p	hsa-miR-34a-5p
hsa-miR-365a-3p	ACO1
hsa-miR-365a-3p	CDCP1
hsa-miR-365a-3p	IL6
hsa-miR-365a-3p	LTBP1
hsa-miR-365a-3p	NEAT1
hsa-miR-365a-3p	NSRP1
hsa-miR-365a-3p	SENP5
hsa-miR-365a-3p	SRRM2
hsa-miR-365a-3p	TMTC2
hsa-miR-365a-3p	ZMYND11
hsa-miR-365a-3p	hsa-miR-130a-3p
hsa-miR-3666	AR
hsa-miR-3666	CHMP3
hsa-miR-3666	CLIP1
hsa-miR-3666	GRB10
hsa-miR-3666	HNF4A
hsa-miR-3666	MECP2
hsa-miR-3666	PSMB5
hsa-miR-3666	RNVU1-7
hsa-miR-3666	TENM1
hsa-miR-3666	TIMP2

hsa-miR-3666	TNF
hsa-miR-3666	ZNF354B
hsa-miR-3666	hsa-miR-130b-3p
hsa-miR-3666	hsa-miR-19a-3p
hsa-miR-3666	hsa-miR-301a-3p
hsa-miR-3666	hsa-miR-301b-3p
hsa-miR-371a-3p	DEPDC1B
hsa-miR-371a-3p	HNF4A
hsa-miR-371a-3p	INTS3
hsa-miR-371a-3p	MAGI1
hsa-miR-371a-3p	PLEKHA1
hsa-miR-371a-3p	SNHG16
hsa-miR-371a-3p	STS
hsa-miR-373-3p	ACO1
hsa-miR-373-3p	ARHGEF10
hsa-miR-373-3p	C17orf58
hsa-miR-373-3p	CHMP3
hsa-miR-373-3p	CSDE1
hsa-miR-373-3p	CXCL8
hsa-miR-373-3p	DEPDC1B
hsa-miR-373-3p	ERCC4
hsa-miR-373-3p	HNF4A
hsa-miR-373-3p	HP1BP3
hsa-miR-373-3p	IL6
hsa-miR-373-3p	L3MBTL2
hsa-miR-373-3p	MAGI1
hsa-miR-373-3p	MECP2
hsa-miR-373-3p	MIR17HG
hsa-miR-373-3p	NARS2
hsa-miR-373-3p	PLEKHA1
hsa-miR-373-3p	POLR2A
hsa-miR-373-3p	PPP2R3A
hsa-miR-373-3p	PSMB5
hsa-miR-373-3p	SH3BP4
hsa-miR-373-3p	SLC12A2
hsa-miR-373-3p	SLC35C2
hsa-miR-373-3p	SNHG16
hsa-miR-373-3p	SSH1
hsa-miR-373-3p	TBRG4
hsa-miR-373-3p	TG
hsa-miR-373-3p	TMTC2
hsa-miR-373-3p	TNF
hsa-miR-373-3p	hsa-miR-106a-5p

hsa-miR-373-3p	hsa-miR-20a-5p
hsa-miR-374a-5p	ARL6
hsa-miR-374a-5p	CCDC89
hsa-miR-374a-5p	CHMP1B
hsa-miR-374a-5p	CSDE1
hsa-miR-374a-5p	CXCL8
hsa-miR-374a-5p	DEPDC1B
hsa-miR-374a-5p	FNBP4
hsa-miR-374a-5p	GTF2H2
hsa-miR-374a-5p	HNF4A
hsa-miR-374a-5p	HP1BP3
hsa-miR-374a-5p	IL10
hsa-miR-374a-5p	IL6
hsa-miR-374a-5p	KHSRP
hsa-miR-374a-5p	LTBP1
hsa-miR-374a-5p	MBD1
hsa-miR-374a-5p	MECP2
hsa-miR-374a-5p	PLAUR
hsa-miR-374a-5p	PLEKHA1
hsa-miR-374a-5p	PPM1K
hsa-miR-374a-5p	SLC12A2
hsa-miR-374a-5p	SOX9
hsa-miR-374a-5p	TMTC2
hsa-miR-374a-5p	TUG1
hsa-miR-374a-5p	USP38
hsa-miR-374a-5p	ZNF318
hsa-miR-374a-5p	hsa-miR-2355-5p
hsa-miR-374a-5p	hsa-miR-373-3p
hsa-miR-374b-5p	ARL6
hsa-miR-374b-5p	CCDC89
hsa-miR-374b-5p	CHMP1B
hsa-miR-374b-5p	CSDE1
hsa-miR-374b-5p	CXCL8
hsa-miR-374b-5p	DEPDC1B
hsa-miR-374b-5p	ERCC4
hsa-miR-374b-5p	HNF4A
hsa-miR-374b-5p	HP1BP3
hsa-miR-374b-5p	IL10
hsa-miR-374b-5p	IL6
hsa-miR-374b-5p	KHSRP
hsa-miR-374b-5p	LTBP1
hsa-miR-374b-5p	MBD1
hsa-miR-374b-5p	MECP2

hsa-miR-374b-5p	NSRP1
hsa-miR-374b-5p	PLAUR
hsa-miR-374b-5p	PLEKHA1
hsa-miR-374b-5p	PPM1K
hsa-miR-374b-5p	SLC12A2
hsa-miR-374b-5p	SOX9
hsa-miR-374b-5p	TMTC2
hsa-miR-374b-5p	USP38
hsa-miR-374b-5p	ZNF318
hsa-miR-374b-5p	hsa-miR-2355-5p
hsa-miR-374b-5p	hsa-miR-373-3p
hsa-miR-375-3p	AR
hsa-miR-375-3p	HNF4A
hsa-miR-375-3p	KHSRP
hsa-miR-375-3p	L3MBTL2
hsa-miR-375-3p	MAGI1
hsa-miR-375-3p	MALAT1
hsa-miR-375-3p	MIR17HG
hsa-miR-375-3p	POLR2A
hsa-miR-375-3p	SH3BP4
hsa-miR-375-3p	TINCR
hsa-miR-375-3p	USP38
hsa-miR-375-3p	ZIC3
hsa-miR-376c-3p	ARL6
hsa-miR-376c-3p	CLIP1
hsa-miR-376c-3p	CXCL8
hsa-miR-376c-3p	DEPDC1B
hsa-miR-376c-3p	DMXL2
hsa-miR-376c-3p	ERCC4
hsa-miR-376c-3p	FNBP4
hsa-miR-376c-3p	HNF4A
hsa-miR-376c-3p	HP1BP3
hsa-miR-376c-3p	IL6
hsa-miR-376c-3p	PCDH19
hsa-miR-376c-3p	PLEKHA1
hsa-miR-376c-3p	PPM1K
hsa-miR-376c-3p	PRRG3
hsa-miR-376c-3p	PSMB5
hsa-miR-376c-3p	PTCH1
hsa-miR-376c-3p	RBMS1
hsa-miR-376c-3p	SLC35C2
hsa-miR-376c-3p	ZNF354B
hsa-miR-378a-5p	NDE1

hsa-miR-378a-5p	NDUFV3
hsa-miR-378a-5p	SRRM2
hsa-miR-381-3p	AR
hsa-miR-381-3p	CLIP1
hsa-miR-381-3p	CSDE1
hsa-miR-381-3p	DMXL2
hsa-miR-381-3p	FNBP4
hsa-miR-381-3p	GRB10
hsa-miR-381-3p	HNF4A
hsa-miR-381-3p	IL6
hsa-miR-381-3p	KHSRP
hsa-miR-381-3p	LTBP1
hsa-miR-381-3p	MAGI1
hsa-miR-381-3p	PCDH19
hsa-miR-381-3p	PLAUR
hsa-miR-381-3p	PLEKHA1
hsa-miR-381-3p	PTCH1
hsa-miR-381-3p	RBM28
hsa-miR-381-3p	RBMS1
hsa-miR-381-3p	SOX9
hsa-miR-381-3p	TUG1
hsa-miR-381-3p	ZMYND11
hsa-miR-381-3p	ZNF318
hsa-miR-382-3p	HP1BP3
hsa-miR-382-3p	NDUFV3
hsa-miR-382-3p	PPM1K
hsa-miR-382-5p	C17orf58
hsa-miR-382-5p	DMXL2
hsa-miR-382-5p	HNF4A
hsa-miR-382-5p	MECP2
hsa-miR-382-5p	MIR17HG
hsa-miR-382-5p	PLEKHA1
hsa-miR-382-5p	PPM1K
hsa-miR-382-5p	RBMS1
hsa-miR-382-5p	SRRM2
hsa-miR-382-5p	TNPO2
hsa-miR-382-5p	TUG1
hsa-miR-382-5p	ZNF318
hsa-miR-421	AR
hsa-miR-421	CHMP1B
hsa-miR-421	CSDE1
hsa-miR-421	DMXL2
hsa-miR-421	FNBP4

hsa-miR-421	GRB10
hsa-miR-421	HNF4A
hsa-miR-421	HP1BP3
hsa-miR-421	IL10
hsa-miR-421	NARS2
hsa-miR-421	PLCG1
hsa-miR-421	POLR2A
hsa-miR-421	SENP5
hsa-miR-421	SNHG16
hsa-miR-421	SOX9
hsa-miR-421	SRPRA
hsa-miR-421	SSH1
hsa-miR-421	TIMP2
hsa-miR-421	hsa-miR-21-5p
hsa-miR-421	hsa-miR-31-5p
hsa-miR-422a	ACO1
hsa-miR-422a	ARHGEF10
hsa-miR-422a	ATF3
hsa-miR-422a	BEND4
hsa-miR-422a	C17orf58
hsa-miR-422a	CARM1
hsa-miR-422a	DMXL2
hsa-miR-422a	GTF2F1
hsa-miR-422a	HNF4A
hsa-miR-422a	L3MBTL2
hsa-miR-422a	MECP2
hsa-miR-422a	PLEKHA1
hsa-miR-422a	SENP5
hsa-miR-422a	SRPRA
hsa-miR-422a	TIMP2
hsa-miR-422a	USP38
hsa-miR-423-3p	ACO1
hsa-miR-423-3p	AR
hsa-miR-423-3p	HNF4A
hsa-miR-423-3p	INTS3
hsa-miR-423-3p	MECP2
hsa-miR-423-3p	NDE1
hsa-miR-423-3p	PSMB5
hsa-miR-423-3p	SRPRA
hsa-miR-425-5p	AR
hsa-miR-425-5p	ARL6
hsa-miR-425-5p	C17orf58
hsa-miR-425-5p	EEF1A1P9

hsa-miR-425-5p	FZR1
hsa-miR-425-5p	MBD1
hsa-miR-425-5p	MECP2
hsa-miR-425-5p	MIR17HG
hsa-miR-425-5p	PPM1K
hsa-miR-425-5p	PTCH1
hsa-miR-425-5p	RABAC1
hsa-miR-425-5p	TIMP2
hsa-miR-425-5p	TMTC2
hsa-miR-425-5p	ZNF318
hsa-miR-425-5p	hsa-miR-2355-5p
hsa-miR-4253	FAM214B
hsa-miR-4253	HNF4A
hsa-miR-4256	AR
hsa-miR-4256	CCDC124
hsa-miR-4256	HNF4A
hsa-miR-4256	SNHG16
hsa-miR-4262	CARM1
hsa-miR-4262	CHMP1B
hsa-miR-4262	CHMP3
hsa-miR-4262	CLIP1
hsa-miR-4262	CSDE1
hsa-miR-4262	DMXL2
hsa-miR-4262	FNBP4
hsa-miR-4262	GRB10
hsa-miR-4262	KHSRP
hsa-miR-4262	MECP2
hsa-miR-4262	PLEKHA1
hsa-miR-4262	PPP2R3A
hsa-miR-4262	PSD4
hsa-miR-4262	TNF
hsa-miR-4262	ZMYND11
hsa-miR-4262	hsa-miR-181d-5p
hsa-miR-4267	ATF3
hsa-miR-4267	HNF4A
hsa-miR-4267	PSD4
hsa-miR-4267	ZMYND11
hsa-miR-4269	CSDE1
hsa-miR-4269	HNF4A
hsa-miR-4277	ATF3
hsa-miR-4277	CSDE1
hsa-miR-4277	HNF4A
hsa-miR-4277	INTS3

hsa-miR-4277	KHSRP
hsa-miR-4277	PPM1K
hsa-miR-4277	RBMS1
hsa-miR-4277	SNHG16
hsa-miR-4282	CSDE1
hsa-miR-4282	DEPDC1B
hsa-miR-4282	FNBP4
hsa-miR-4282	HP1BP3
hsa-miR-4282	PLEKHA1
hsa-miR-4282	PRMT2
hsa-miR-4282	RBMS1
hsa-miR-4282	RPL30
hsa-miR-4282	SLC12A2
hsa-miR-4282	SNHG16
hsa-miR-4282	SRPRA
hsa-miR-4282	TNPO2
hsa-miR-4284	RABAC1
hsa-miR-4284	TNPO2
hsa-miR-4287	CLIP1
hsa-miR-4287	HNF4A
hsa-miR-4287	LTBP1
hsa-miR-4287	PPM1K
hsa-miR-4287	PSMB5
hsa-miR-4287	RBM28
hsa-miR-4288	ACO1
hsa-miR-4288	AR
hsa-miR-4288	HNF4A
hsa-miR-4288	MALAT1
hsa-miR-4292	AR
hsa-miR-4292	HNF4A
hsa-miR-4292	SNHG16
hsa-miR-4292	SRRM2
hsa-miR-4292	TNPO2
hsa-miR-4292	ZNF354B
hsa-miR-4294	HNF4A
hsa-miR-4294	MIR17HG
hsa-miR-4294	TIMP2
hsa-miR-4295	AR
hsa-miR-4295	ATF3
hsa-miR-4295	BEND4
hsa-miR-4295	CHMP3
hsa-miR-4295	CLIP1
hsa-miR-4295	CSDE1

hsa-miR-4295	FNBP4
hsa-miR-4295	GRB10
hsa-miR-4295	HNF4A
hsa-miR-4295	LTBP1
hsa-miR-4295	MECP2
hsa-miR-4295	MIR17HG
hsa-miR-4295	PSMB5
hsa-miR-4295	RBMS1
hsa-miR-4295	RNVU1-7
hsa-miR-4295	TENM1
hsa-miR-4295	TIMP2
hsa-miR-4295	TNF
hsa-miR-4295	ZNF354B
hsa-miR-4295	hsa-miR-130b-3p
hsa-miR-4295	hsa-miR-19a-3p
hsa-miR-4295	hsa-miR-301a-3p
hsa-miR-4295	hsa-miR-301b-3p
hsa-miR-4297	CARM1
hsa-miR-4297	HNF4A
hsa-miR-4297	LTBP1
hsa-miR-4297	MAGI1
hsa-miR-4297	SNHG16
hsa-miR-4301	HNF4A
hsa-miR-4301	MECP2
hsa-miR-4301	TMTC2
hsa-miR-4301	TNPO2
hsa-miR-4301	ZNF354B
hsa-miR-4309	HNF4A
hsa-miR-431-5p	ATF3
hsa-miR-431-5p	CXCL8
hsa-miR-431-5p	HNF4A
hsa-miR-431-5p	MPPE1
hsa-miR-431-5p	SNHG16
hsa-miR-431-5p	SRRM2
hsa-miR-431-5p	TBRG4
hsa-miR-4310	ATF3
hsa-miR-4310	FZR1
hsa-miR-4310	PLEKHA1
hsa-miR-432-3p	ATF3
hsa-miR-432-3p	L3MBTL2
hsa-miR-432-3p	PSMB5
hsa-miR-432-3p	TNPO2
hsa-miR-432-5p	CDCP1

hsa-miR-432-5p	CHMP3
hsa-miR-432-5p	CSDE1
hsa-miR-432-5p	HNF4A
hsa-miR-432-5p	IL10
hsa-miR-432-5p	MECP2
hsa-miR-432-5p	PPM1K
hsa-miR-432-5p	SH3BP4
hsa-miR-432-5p	SNHG16
hsa-miR-432-5p	TMEM201
hsa-miR-432-5p	TNPO2
hsa-miR-4326	SNHG16
hsa-miR-4326	SRRM2
hsa-miR-4329	HNF4A
hsa-miR-4329	SNHG16
hsa-miR-4329	TMTC2
hsa-miR-448	ACO1
hsa-miR-448	CHMP3
hsa-miR-448	CLIP1
hsa-miR-448	CSDE1
hsa-miR-448	DMXL2
hsa-miR-448	FNBP4
hsa-miR-448	HP1BP3
hsa-miR-448	IL6
hsa-miR-448	L3MBTL2
hsa-miR-448	MAGI1
hsa-miR-448	MALAT1
hsa-miR-448	MECP2
hsa-miR-448	MPPE1
hsa-miR-448	PLEKHA1
hsa-miR-448	PTCH1
hsa-miR-448	RBMS1
hsa-miR-448	SH3BP4
hsa-miR-448	SLC12A2
hsa-miR-448	TNPO2
hsa-miR-448	ZMYND11
hsa-miR-448	ZNF354B
hsa-miR-448	hsa-miR-1278
hsa-miR-449a	ARL6
hsa-miR-449a	ATF3
hsa-miR-449a	BEND4
hsa-miR-449a	CHI3L1
hsa-miR-449a	GRB10
hsa-miR-449a	GTF2F1

hsa-miR-449a	HNF4A
hsa-miR-449a	HP1BP3
hsa-miR-449a	INTS3
hsa-miR-449a	MECP2
hsa-miR-449a	NEAT1
hsa-miR-449a	PLCG1
hsa-miR-449a	PLEKHA1
hsa-miR-449a	PPP2R3A
hsa-miR-449a	SAMD4B
hsa-miR-449a	SLC12A2
hsa-miR-449a	SRPRA
hsa-miR-449a	STS
hsa-miR-449a	TMEM201
hsa-miR-449a	TPPP
hsa-miR-449a	TUG1
hsa-miR-449a	ZMYND11
hsa-miR-449a	ZNF318
hsa-miR-449a	hsa-let-7g-5p
hsa-miR-449a	hsa-miR-1229-3p
hsa-miR-449a	hsa-miR-20a-5p
hsa-miR-449a	hsa-miR-330-3p
hsa-miR-452-3p	ACO1
hsa-miR-452-3p	CDCP1
hsa-miR-452-3p	L3MBTL2
hsa-miR-452-3p	MAGI1
hsa-miR-452-3p	PPM1K
hsa-miR-452-3p	RPL30
hsa-miR-452-3p	SRPRA
hsa-miR-452-3p	TMTC2
hsa-miR-454-3p	AR
hsa-miR-454-3p	CHMP3
hsa-miR-454-3p	CLIP1
hsa-miR-454-3p	CSDE1
hsa-miR-454-3p	ERCC4
hsa-miR-454-3p	FNBP4
hsa-miR-454-3p	GRB10
hsa-miR-454-3p	GTF2H2
hsa-miR-454-3p	HNF4A
hsa-miR-454-3p	LTBP1
hsa-miR-454-3p	MECP2
hsa-miR-454-3p	MIR17HG
hsa-miR-454-3p	PLEKHA1
hsa-miR-454-3p	POLR2A

hsa-miR-454-3p	PSMB5
hsa-miR-454-3p	RNVU1-7
hsa-miR-454-3p	SH3BP4
hsa-miR-454-3p	SLC12A2
hsa-miR-454-3p	TENM1
hsa-miR-454-3p	TIMP2
hsa-miR-454-3p	TNF
hsa-miR-454-3p	ZMYND11
hsa-miR-454-3p	ZNF354B
hsa-miR-454-3p	hsa-miR-130b-3p
hsa-miR-454-3p	hsa-miR-19a-3p
hsa-miR-454-3p	hsa-miR-301a-3p
hsa-miR-454-3p	hsa-miR-301b-3p
hsa-miR-454-3p	hsa-miR-363-3p
hsa-miR-4635	ATF3
hsa-miR-4635	HNF4A
hsa-miR-4635	MPPE1
hsa-miR-4635	PLCG1
hsa-miR-4635	TPPP
hsa-miR-4695-3p	PPM1K
hsa-miR-4695-5p	CDCP1
hsa-miR-484	CSDE1
hsa-miR-484	HNF4A
hsa-miR-484	KHSRP
hsa-miR-484	L3MBTL2
hsa-miR-484	NDUFV3
hsa-miR-484	PCDH19
hsa-miR-484	POLR2A
hsa-miR-484	PRPF31
hsa-miR-484	PSMB5
hsa-miR-484	SRPRA
hsa-miR-485-5p	ACO1
hsa-miR-485-5p	CDCP1
hsa-miR-485-5p	CHMP1B
hsa-miR-485-5p	CSDE1
hsa-miR-485-5p	HID1
hsa-miR-485-5p	L3MBTL2
hsa-miR-485-5p	MPPE1
hsa-miR-485-5p	NEAT1
hsa-miR-485-5p	PLEKHA1
hsa-miR-485-5p	PSD4
hsa-miR-485-5p	RABAC1
hsa-miR-485-5p	SLC12A2

hsa-miR-485-5p	SNHG16
hsa-miR-485-5p	TMEM201
hsa-miR-485-5p	TNPO2
hsa-miR-485-5p	ZMYND11
hsa-miR-485-5p	hsa-miR-20a-5p
hsa-miR-490-3p	ACO1
hsa-miR-490-3p	CHMP3
hsa-miR-490-3p	HNF4A
hsa-miR-490-3p	MIR17HG
hsa-miR-490-3p	SNHG16
hsa-miR-490-3p	TNPO2
hsa-miR-490-3p	TUG1
hsa-miR-490-3p	ZMYND11
hsa-miR-490-3p	hsa-miR-152-3p
hsa-miR-490-3p	hsa-miR-421
hsa-miR-491-5p	AR
hsa-miR-491-5p	CDCP1
hsa-miR-491-5p	FAM214B
hsa-miR-491-5p	HP1BP3
hsa-miR-491-5p	LTBP1
hsa-miR-491-5p	MECP2
hsa-miR-491-5p	SAMD4B
hsa-miR-491-5p	SH3BP4
hsa-miR-491-5p	SOX9
hsa-miR-491-5p	SRPRA
hsa-miR-491-5p	SRRM2
hsa-miR-491-5p	hsa-miR-122-5p
hsa-miR-492	HNF4A
hsa-miR-492	HP1BP3
hsa-miR-494-3p	ACO1
hsa-miR-494-3p	ARHGEF10
hsa-miR-494-3p	ARL6
hsa-miR-494-3p	ATF3
hsa-miR-494-3p	C17orf58
hsa-miR-494-3p	CARM1
hsa-miR-494-3p	CHMP1B
hsa-miR-494-3p	CLIP1
hsa-miR-494-3p	CSDE1
hsa-miR-494-3p	DMXL2
hsa-miR-494-3p	ERCC4
hsa-miR-494-3p	FNBP4
hsa-miR-494-3p	GTF2F1
hsa-miR-494-3p	HNF4A

hsa-miR-494-3p	HP1BP3
hsa-miR-494-3p	IL10
hsa-miR-494-3p	NARS2
hsa-miR-494-3p	PLEKHA1
hsa-miR-494-3p	RBMS1
hsa-miR-494-3p	SLC12A2
hsa-miR-494-3p	SNHG16
hsa-miR-494-3p	SOX9
hsa-miR-494-3p	SRRM2
hsa-miR-494-3p	TMEM201
hsa-miR-494-3p	TMTC2
hsa-miR-494-3p	TNF
hsa-miR-494-3p	USP38
hsa-miR-494-3p	ZIC3
hsa-miR-494-3p	hsa-miR-33a-5p
hsa-miR-495-3p	ACO1
hsa-miR-495-3p	ARHGEF10
hsa-miR-495-3p	C17orf58
hsa-miR-495-3p	CHMP1B
hsa-miR-495-3p	CSDE1
hsa-miR-495-3p	DMXL2
hsa-miR-495-3p	GRB10
hsa-miR-495-3p	HNF4A
hsa-miR-495-3p	HP1BP3
hsa-miR-495-3p	IL10
hsa-miR-495-3p	INTS3
hsa-miR-495-3p	KHSRP
hsa-miR-495-3p	LTBP1
hsa-miR-495-3p	MIR17HG
hsa-miR-495-3p	NARS2
hsa-miR-495-3p	POLR2A
hsa-miR-495-3p	PPM1K
hsa-miR-495-3p	PTCH1
hsa-miR-495-3p	RBM28
hsa-miR-495-3p	SAMD4B
hsa-miR-495-3p	SENP5
hsa-miR-495-3p	SH3BP4
hsa-miR-495-3p	SLC12A2
hsa-miR-495-3p	SNHG16
hsa-miR-495-3p	SRRM2
hsa-miR-495-3p	TIMP2
hsa-miR-495-3p	TMEM201
hsa-miR-495-3p	TNPO2

hsa-miR-495-3p	TUG1
hsa-miR-495-3p	USP38
hsa-miR-495-3p	ZMYND11
hsa-miR-495-3p	ZNF318
hsa-miR-495-3p	hsa-miR-421
hsa-miR-499a-5p	AR
hsa-miR-499a-5p	DEPDC1B
hsa-miR-499a-5p	ERCC4
hsa-miR-499a-5p	HNF4A
hsa-miR-499a-5p	HP1BP3
hsa-miR-499a-5p	INTS3
hsa-miR-499a-5p	LTBP1
hsa-miR-499a-5p	MAGI1
hsa-miR-499a-5p	MECP2
hsa-miR-499a-5p	PLEKHA1
hsa-miR-499a-5p	PTCH1
hsa-miR-499a-5p	SENP5
hsa-miR-499a-5p	SLC12A2
hsa-miR-499a-5p	SRPRA
hsa-miR-500b-3p	CHMP3
hsa-miR-500b-3p	PTCH1
hsa-miR-503-3p	IL10
hsa-miR-505-3p	ACO1
hsa-miR-505-3p	BEND4
hsa-miR-505-3p	FZR1
hsa-miR-505-3p	GRB10
hsa-miR-505-3p	HNF4A
hsa-miR-505-3p	MAGI1
hsa-miR-505-3p	MECP2
hsa-miR-505-3p	RBM28
hsa-miR-505-3p	SNHG16
hsa-miR-505-3p	SRPRA
hsa-miR-505-3p	SRRM2
hsa-miR-505-3p	TMEM201
hsa-miR-505-3p	TUG1
hsa-miR-505-3p	ZMYND11
hsa-miR-505-3p	hsa-miR-149-5p
hsa-miR-505-3p	hsa-miR-21-5p
hsa-miR-505-3p	hsa-miR-494-3p
hsa-miR-508-3p	CDCP1
hsa-miR-508-3p	CLIP1
hsa-miR-508-3p	FNBP4
hsa-miR-508-3p	HID1

hsa-miR-508-3p	HNF4A
hsa-miR-508-3p	MALAT1
hsa-miR-508-3p	MECP2
hsa-miR-508-3p	PLEKHA1
hsa-miR-508-3p	POLR2A
hsa-miR-508-3p	PPM1K
hsa-miR-508-3p	SNHG16
hsa-miR-508-3p	ZNF318
hsa-miR-509-3p	ARHGEF10
hsa-miR-509-3p	C17orf58
hsa-miR-509-3p	CLIP1
hsa-miR-509-3p	GRB10
hsa-miR-509-3p	MALAT1
hsa-miR-509-3p	MECP2
hsa-miR-509-3p	PPP2R3A
hsa-miR-509-3p	PSMB5
hsa-miR-509-3p	SH3BP4
hsa-miR-509-3p	hsa-miR-17-5p
hsa-miR-510-3p	RABAC1
hsa-miR-511-5p	C17orf58
hsa-miR-511-5p	CARM1
hsa-miR-511-5p	DEPDC1B
hsa-miR-511-5p	FNBP4
hsa-miR-511-5p	HP1BP3
hsa-miR-511-5p	PLEKHA1
hsa-miR-511-5p	RBMS1
hsa-miR-511-5p	SH3BP4
hsa-miR-511-5p	TNPO2
hsa-miR-511-5p	hsa-miR-17-5p
hsa-miR-511-5p	hsa-miR-181a-5p
hsa-miR-511-5p	hsa-miR-181b-5p
hsa-miR-511-5p	hsa-miR-181c-5p
hsa-miR-511-5p	hsa-miR-181d-5p
hsa-miR-511-5p	hsa-miR-4262
hsa-miR-513a-5p	KHSRP
hsa-miR-513a-5p	PPM1K
hsa-miR-513a-5p	SAMD4B
hsa-miR-513a-5p	SLC12A2
hsa-miR-513a-5p	SNHG16
hsa-miR-513a-5p	TMTC2
hsa-miR-514a-3p	LTBP1
hsa-miR-514b-3p	CARM1
hsa-miR-514b-3p	LTBP1

hsa-miR-514b-3p	PLEKHA1
hsa-miR-515-3p	GRB10
hsa-miR-515-3p	MIR17HG
hsa-miR-515-3p	SLC12A2
hsa-miR-515-3p	TUG1
hsa-miR-515-3p	hsa-miR-17-5p
hsa-miR-516b-3p	ATF3
hsa-miR-516b-3p	CSDE1
hsa-miR-516b-3p	FNBP4
hsa-miR-516b-3p	PLCG1
hsa-miR-516b-3p	SH3BP4
hsa-miR-518c-5p	ARHGEF10
hsa-miR-518c-5p	CLIP1
hsa-miR-518c-5p	INTS3
hsa-miR-518c-5p	MECP2
hsa-miR-519a-3p	CLIP1
hsa-miR-519a-3p	CSDE1
hsa-miR-519a-3p	CXCL8
hsa-miR-519a-3p	FNBP4
hsa-miR-519a-3p	MECP2
hsa-miR-519a-3p	RBMS1
hsa-miR-519a-3p	SLC35C2
hsa-miR-519a-3p	SNHG16
hsa-miR-519a-3p	TIMP2
hsa-miR-519b-3p	CLIP1
hsa-miR-519b-3p	CSDE1
hsa-miR-519b-3p	CXCL8
hsa-miR-519b-3p	FNBP4
hsa-miR-519b-3p	HNF4A
hsa-miR-519b-3p	L3MBTL2
hsa-miR-519b-3p	MECP2
hsa-miR-519b-3p	RBMS1
hsa-miR-519b-3p	SLC35C2
hsa-miR-519b-3p	TIMP2
hsa-miR-519c-3p	CLIP1
hsa-miR-519c-3p	CSDE1
hsa-miR-519c-3p	FNBP4
hsa-miR-519c-3p	HNF4A
hsa-miR-519c-3p	L3MBTL2
hsa-miR-519c-3p	MECP2
hsa-miR-519c-3p	RBMS1
hsa-miR-519c-3p	SH3BP4
hsa-miR-519c-3p	SLC35C2

hsa-miR-519c-3p	TIMP2
hsa-miR-519d-3p	ARHGEF10
hsa-miR-519d-3p	CSDE1
hsa-miR-519d-3p	CXCL8
hsa-miR-519d-3p	FNBP4
hsa-miR-519d-3p	HNF4A
hsa-miR-519d-3p	HP1BP3
hsa-miR-519d-3p	IL6
hsa-miR-519d-3p	L3MBTL2
hsa-miR-519d-3p	MECP2
hsa-miR-519d-3p	PLEKHA1
hsa-miR-519d-3p	PPP2R3A
hsa-miR-519d-3p	SENP5
hsa-miR-519d-3p	SH3BP4
hsa-miR-519d-3p	SNHG16
hsa-miR-519d-3p	SRRM2
hsa-miR-519d-3p	SSH1
hsa-miR-519d-3p	TIMP2
hsa-miR-519d-3p	TNF
hsa-miR-519d-3p	TPPP
hsa-miR-519d-3p	ZNF318
hsa-miR-519d-3p	ZNF354B
hsa-miR-519e-3p	CXCL8
hsa-miR-519e-3p	GRB10
hsa-miR-519e-3p	HNF4A
hsa-miR-519e-3p	MIR17HG
hsa-miR-519e-3p	PLEKHA1
hsa-miR-519e-3p	PPM1K
hsa-miR-519e-3p	SLC12A2
hsa-miR-520a-3p	ARHGEF10
hsa-miR-520a-3p	BNC1
hsa-miR-520a-3p	CHMP3
hsa-miR-520a-3p	CSDE1
hsa-miR-520a-3p	CXCL8
hsa-miR-520a-3p	ERCC4
hsa-miR-520a-3p	FNBP4
hsa-miR-520a-3p	HNF4A
hsa-miR-520a-3p	HP1BP3
hsa-miR-520a-3p	L3MBTL2
hsa-miR-520a-3p	MAGI1
hsa-miR-520a-3p	MECP2
hsa-miR-520a-3p	MIR17HG
hsa-miR-520a-3p	RBMS1

hsa-miR-520a-3p	SLC35C2
hsa-miR-520a-3p	SNHG16
hsa-miR-520a-3p	SOX9
hsa-miR-520a-3p	SRRM2
hsa-miR-520a-3p	TMTC2
hsa-miR-520a-3p	TNF
hsa-miR-520a-3p	TNPO2
hsa-miR-520a-3p	hsa-miR-106a-5p
hsa-miR-520a-3p	hsa-miR-132-3p
hsa-miR-520a-3p	hsa-miR-20a-5p
hsa-miR-520a-3p	hsa-miR-212-3p
hsa-miR-520a-3p	hsa-miR-374a-5p
hsa-miR-520a-3p	hsa-miR-374b-5p
hsa-miR-524-3p	ATF3
hsa-miR-524-3p	CSDE1
hsa-miR-524-3p	FNBP4
hsa-miR-524-3p	HNF4A
hsa-miR-524-3p	HP1BP3
hsa-miR-524-3p	KHSRP
hsa-miR-524-3p	POLR2A
hsa-miR-524-3p	SLC12A2
hsa-miR-524-3p	SOX9
hsa-miR-524-3p	hsa-miR-106a-5p
hsa-miR-524-3p	hsa-miR-106b-5p
hsa-miR-524-3p	hsa-miR-17-5p
hsa-miR-524-3p	hsa-miR-204-5p
hsa-miR-524-3p	hsa-miR-20a-5p
hsa-miR-524-3p	hsa-miR-20b-5p
hsa-miR-524-3p	hsa-miR-211-5p
hsa-miR-524-3p	hsa-miR-373-3p
hsa-miR-524-3p	hsa-miR-519d-3p
hsa-miR-524-3p	hsa-miR-520a-3p
hsa-miR-526b-3p	ACO1
hsa-miR-526b-3p	CSDE1
hsa-miR-526b-3p	FNBP4
hsa-miR-526b-3p	L3MBTL2
hsa-miR-526b-3p	MECP2
hsa-miR-526b-3p	SLC12A2
hsa-miR-526b-3p	TBRG4
hsa-miR-526b-3p	TIMP2
hsa-miR-526b-3p	TNPO2
hsa-miR-526b-3p	USP38
hsa-miR-526b-3p	ZNF354B

hsa-miR-542-3p	ARL6
hsa-miR-542-3p	ATF3
hsa-miR-542-3p	CARM1
hsa-miR-542-3p	CSDE1
hsa-miR-542-3p	L3MBTL2
hsa-miR-542-3p	PLEKHA1
hsa-miR-542-3p	SLC12A2
hsa-miR-542-3p	SNHG16
hsa-miR-542-3p	SRRM2
hsa-miR-542-3p	hsa-miR-130a-3p
hsa-miR-542-3p	hsa-miR-23a-3p
hsa-miR-543	ACO1
hsa-miR-543	ARHGEF10
hsa-miR-543	CHMP1B
hsa-miR-543	CLIP1
hsa-miR-543	CSDE1
hsa-miR-543	DEPDC1B
hsa-miR-543	DMXL2
hsa-miR-543	GRB10
hsa-miR-543	HNF4A
hsa-miR-543	HP1BP3
hsa-miR-543	IL10
hsa-miR-543	INTS3
hsa-miR-543	PLEKHA1
hsa-miR-543	PTCH1
hsa-miR-543	RBMS1
hsa-miR-543	SAMD4B
hsa-miR-543	SLC12A2
hsa-miR-543	SLC35C2
hsa-miR-543	TNF
hsa-miR-543	USP38
hsa-miR-543	ZIC3
hsa-miR-548c-3p	ACO1
hsa-miR-548c-3p	AR
hsa-miR-548c-3p	CLIP1
hsa-miR-548c-3p	CSDE1
hsa-miR-548c-3p	FNBP4
hsa-miR-548c-3p	HNF4A
hsa-miR-548c-3p	LTBP1
hsa-miR-548c-3p	MIR17HG
hsa-miR-548c-3p	PLEKHA1
hsa-miR-548c-3p	POLR2A
hsa-miR-548c-3p	PTCH1

hsa-miR-548c-3p	RBMS1
hsa-miR-548c-3p	SAMD4B
hsa-miR-548c-3p	SLC12A2
hsa-miR-548c-3p	SNHG16
hsa-miR-548c-3p	TNPO2
hsa-miR-548c-3p	TPPP
hsa-miR-548c-3p	ZIC3
hsa-miR-548c-3p	hsa-miR-19a-3p
hsa-miR-548c-3p	hsa-miR-19b-3p
hsa-miR-548d-5p	CLIP1
hsa-miR-548d-5p	KHSRP
hsa-miR-548k	BEND4
hsa-miR-548k	FNBP4
hsa-miR-548k	HNF4A
hsa-miR-548k	PSMB5
hsa-miR-548k	SRPRA
hsa-miR-548t-5p	CSDE1
hsa-miR-548t-5p	FNBP4
hsa-miR-548t-5p	GRB10
hsa-miR-548t-5p	HNF4A
hsa-miR-548t-5p	KHSRP
hsa-miR-548t-5p	MAGI1
hsa-miR-548t-5p	SAMD4B
hsa-miR-548t-5p	SNHG16
hsa-miR-548t-5p	TIMP2
hsa-miR-548t-5p	USP38
hsa-miR-548u	DEPDC1B
hsa-miR-548u	GRB10
hsa-miR-548u	HNF4A
hsa-miR-548u	NDUFV3
hsa-miR-548u	PPM1K
hsa-miR-548u	SNHG16
hsa-miR-548u	TNPO2
hsa-miR-548v	HNF4A
hsa-miR-548v	MIR17HG
hsa-miR-548v	PLCG1
hsa-miR-548v	SRPRA
hsa-miR-548v	hsa-miR-19a-3p
hsa-miR-548v	hsa-miR-19b-3p
hsa-miR-556-3p	LTBP1
hsa-miR-556-3p	PPM1K
hsa-miR-556-3p	PSMB5
hsa-miR-556-3p	SLC12A2

hsa-miR-556-3p	ZMYND11
hsa-miR-561-3p	CSDE1
hsa-miR-561-3p	FNBP4
hsa-miR-561-3p	HNF4A
hsa-miR-561-3p	KHSRP
hsa-miR-561-3p	PTCH1
hsa-miR-561-3p	SAMD4B
hsa-miR-561-3p	TMTC2
hsa-miR-569	CLIP1
hsa-miR-569	HNF4A
hsa-miR-573	CHMP3
hsa-miR-573	ERCC4
hsa-miR-573	HNF4A
hsa-miR-573	KHSRP
hsa-miR-573	PCDH19
hsa-miR-573	PLCG1
hsa-miR-573	SNHG16
hsa-miR-573	TNPO2
hsa-miR-578	ACO1
hsa-miR-578	HNF4A
hsa-miR-578	KHSRP
hsa-miR-578	MIR17HG
hsa-miR-578	SRPRA
hsa-miR-578	TPPP
hsa-miR-580-3p	ATF3
hsa-miR-580-3p	BEND4
hsa-miR-580-3p	ERCC4
hsa-miR-580-3p	HNF4A
hsa-miR-580-3p	SAMD4B
hsa-miR-580-3p	TNPO2
hsa-miR-580-3p	hsa-miR-200b-3p
hsa-miR-580-3p	hsa-miR-200c-3p
hsa-miR-584-5p	HNF4A
hsa-miR-584-5p	NDUFV3
hsa-miR-589-5p	MALAT1
hsa-miR-590-3p	ACO1
hsa-miR-590-3p	AR
hsa-miR-590-3p	ARHGEF10
hsa-miR-590-3p	ARL6
hsa-miR-590-3p	ATF3
hsa-miR-590-3p	CHMP1B
hsa-miR-590-3p	CSDE1
hsa-miR-590-3p	DEPDC1B

hsa-miR-590-3p	DMXL2
hsa-miR-590-3p	ERCC4
hsa-miR-590-3p	FAM214B
hsa-miR-590-3p	FNBP4
hsa-miR-590-3p	GRB10
hsa-miR-590-3p	HP1BP3
hsa-miR-590-3p	IL10
hsa-miR-590-3p	KHSRP
hsa-miR-590-3p	LTBP1
hsa-miR-590-3p	MAGI1
hsa-miR-590-3p	MBD1
hsa-miR-590-3p	MECP2
hsa-miR-590-3p	NDUFV3
hsa-miR-590-3p	NORAD
hsa-miR-590-3p	PCDH19
hsa-miR-590-3p	PLAUR
hsa-miR-590-3p	PLCG1
hsa-miR-590-3p	PLEKHA1
hsa-miR-590-3p	PPM1K
hsa-miR-590-3p	PPP2R3A
hsa-miR-590-3p	RBMS1
hsa-miR-590-3p	RPL30
hsa-miR-590-3p	SENP5
hsa-miR-590-3p	SH3BP4
hsa-miR-590-3p	SLC12A2
hsa-miR-590-3p	SRPRA
hsa-miR-590-3p	SRRM2
hsa-miR-590-3p	TMTC2
hsa-miR-590-3p	TUG1
hsa-miR-590-3p	USP38
hsa-miR-590-3p	ZIC3
hsa-miR-590-3p	ZMYND11
hsa-miR-590-5p	ACO1
hsa-miR-590-5p	AR
hsa-miR-590-5p	ARL6
hsa-miR-590-5p	ATF3
hsa-miR-590-5p	HNF4A
hsa-miR-590-5p	IL10
hsa-miR-590-5p	INTS3
hsa-miR-590-5p	LTBP1
hsa-miR-590-5p	NME1-NME2
hsa-miR-590-5p	PCDH19
hsa-miR-590-5p	PLCG1

hsa-miR-590-5p	PLEKHA1
hsa-miR-590-5p	RABAC1
hsa-miR-590-5p	RBMS1
hsa-miR-590-5p	STS
hsa-miR-590-5p	TUG1
hsa-miR-590-5p	hsa-miR-21-5p
hsa-miR-591	AR
hsa-miR-591	ATF3
hsa-miR-591	HNF4A
hsa-miR-591	TNPO2
hsa-miR-591	hsa-miR-543
hsa-miR-612	AR
hsa-miR-612	HNF4A
hsa-miR-612	SNHG16
hsa-miR-612	SRPRA
hsa-miR-612	TMTC2
hsa-miR-612	TUG1
hsa-miR-612	hsa-miR-214-3p
hsa-miR-613	ATF3
hsa-miR-613	HNF4A
hsa-miR-613	HP1BP3
hsa-miR-613	KHSRP
hsa-miR-613	MALAT1
hsa-miR-613	NEAT1
hsa-miR-613	PLCG1
hsa-miR-613	PLEKHA1
hsa-miR-613	PPM1K
hsa-miR-613	PPP2R3A
hsa-miR-613	PRMT2
hsa-miR-613	SLC12A2
hsa-miR-613	SOX9
hsa-miR-613	TNPO2
hsa-miR-613	TPPP
hsa-miR-613	ZMYND11
hsa-miR-613	hsa-miR-543
hsa-miR-617	ACO1
hsa-miR-617	AR
hsa-miR-617	HNF4A
hsa-miR-617	HP1BP3
hsa-miR-617	SNHG16
hsa-miR-618	AR
hsa-miR-618	HNF4A
hsa-miR-618	HP1BP3

hsa-miR-618	PPM1K
hsa-miR-618	PPP2R3A
hsa-miR-618	TNPO2
hsa-miR-619-3p	ACO1
hsa-miR-619-3p	AR
hsa-miR-619-3p	HNF4A
hsa-miR-619-3p	hsa-miR-101-3p
hsa-miR-620	CSDE1
hsa-miR-620	FNBP4
hsa-miR-620	HNF4A
hsa-miR-625-5p	AR
hsa-miR-625-5p	HNF4A
hsa-miR-625-5p	SLC35C2
hsa-miR-625-5p	TNPO2
hsa-miR-629-3p	CARM1
hsa-miR-629-3p	LTBP1
hsa-miR-629-3p	SOX9
hsa-miR-632	ACO1
hsa-miR-632	AR
hsa-miR-632	BNC1
hsa-miR-632	HNF4A
hsa-miR-641	AR
hsa-miR-641	DEPDC1B
hsa-miR-641	HNF4A
hsa-miR-641	HP1BP3
hsa-miR-641	PSD4
hsa-miR-641	SNHG16
hsa-miR-641	SOX9
hsa-miR-670-3p	CARM1
hsa-miR-670-3p	PLCG1
hsa-miR-670-3p	TNPO2
hsa-miR-6733-3p	BEND4
hsa-miR-6733-3p	SH3BP4
hsa-miR-6832-5p	NDUFV3
hsa-miR-6832-5p	RBM28
hsa-miR-744-3p	PLCG1
hsa-miR-744-5p	FZR1
hsa-miR-744-5p	HNF4A
hsa-miR-744-5p	INTS3
hsa-miR-744-5p	KHSRP
hsa-miR-744-5p	L3MBTL2
hsa-miR-744-5p	ND1
hsa-miR-744-5p	NDE1

hsa-miR-744-5p	POLR2A
hsa-miR-744-5p	SAMD4B
hsa-miR-744-5p	SRRM2
hsa-miR-744-5p	ZNF318
hsa-miR-744-5p	hsa-miR-499a-5p
hsa-miR-767-3p	ACO1
hsa-miR-767-3p	CSDE1
hsa-miR-767-3p	PRMT2
hsa-miR-767-3p	PTCH1
hsa-miR-874-3p	ACO1
hsa-miR-874-3p	EML2
hsa-miR-874-3p	HP1BP3
hsa-miR-874-3p	NDUFV3
hsa-miR-874-3p	PLCG1
hsa-miR-874-3p	ZNF318
hsa-miR-874-3p	ZNF354B
hsa-miR-874-3p	hsa-let-7b-5p
hsa-miR-874-3p	hsa-miR-132-3p
hsa-miR-874-3p	hsa-miR-211-5p
hsa-miR-877-3p	GRB10
hsa-miR-877-3p	ND1
hsa-miR-877-3p	ND2
hsa-miR-877-3p	NDE1
hsa-miR-877-3p	SSH1
hsa-miR-887-3p	hsa-miR-19a-3p
hsa-miR-887-3p	hsa-miR-19b-3p
hsa-miR-920	CARM1
hsa-miR-920	GRB10
hsa-miR-920	HNF4A
hsa-miR-920	SNHG16
hsa-miR-920	TIMP2
hsa-miR-924	AR
hsa-miR-924	DEPDC1B
hsa-miR-924	PLCG1
hsa-miR-924	TBRG4
hsa-miR-924	TIMP2
hsa-miR-92a-1-5p	FZR1
hsa-miR-92a-1-5p	HP1BP3
hsa-miR-92a-1-5p	LTBP1
hsa-miR-92a-1-5p	PLEKHA1
hsa-miR-92a-1-5p	SRPRA
hsa-miR-92a-3p	ARHGEF10
hsa-miR-92a-3p	CARM1

hsa-miR-92a-3p	CDCP1
hsa-miR-92a-3p	CSDE1
hsa-miR-92a-3p	CXCL8
hsa-miR-92a-3p	DMXL2
hsa-miR-92a-3p	FNBP4
hsa-miR-92a-3p	FZR1
hsa-miR-92a-3p	HP1BP3
hsa-miR-92a-3p	KHSRP
hsa-miR-92a-3p	LTBP1
hsa-miR-92a-3p	MALAT1
hsa-miR-92a-3p	MECP2
hsa-miR-92a-3p	NDUFV3
hsa-miR-92a-3p	PLCG1
hsa-miR-92a-3p	PLEKHA1
hsa-miR-92a-3p	RBM28
hsa-miR-92a-3p	SH3BP4
hsa-miR-92a-3p	SLC12A2
hsa-miR-92a-3p	SRPRA
hsa-miR-92a-3p	STS
hsa-miR-92a-3p	TPPP
hsa-miR-92a-3p	ZNF354B
hsa-miR-92a-3p	hsa-miR-130a-3p
hsa-miR-92a-3p	hsa-miR-130b-3p
hsa-miR-92a-3p	hsa-miR-301a-3p
hsa-miR-92a-3p	hsa-miR-301b-3p
hsa-miR-92a-3p	hsa-miR-454-3p
hsa-miR-93-3p	CSDE1
hsa-miR-93-3p	FNBP4
hsa-miR-93-3p	SAMD4B
hsa-miR-93-3p	TIMP2
hsa-miR-93-3p	ZIC3
hsa-miR-93-5p	AR
hsa-miR-93-5p	ARHGEF10
hsa-miR-93-5p	ATF3
hsa-miR-93-5p	CDCP1
hsa-miR-93-5p	CSDE1
hsa-miR-93-5p	CXCL8
hsa-miR-93-5p	ERCC4
hsa-miR-93-5p	FNBP4
hsa-miR-93-5p	HNF4A
hsa-miR-93-5p	HP1BP3
hsa-miR-93-5p	IL6
hsa-miR-93-5p	L3MBTL2

hsa-miR-93-5p	MECP2
hsa-miR-93-5p	PLEKHA1
hsa-miR-93-5p	POLR2A
hsa-miR-93-5p	PPP2R3A
hsa-miR-93-5p	PSMB5
hsa-miR-93-5p	RBMS1
hsa-miR-93-5p	RPL30
hsa-miR-93-5p	SAMD4B
hsa-miR-93-5p	SENP5
hsa-miR-93-5p	SH3BP4
hsa-miR-93-5p	SNHG16
hsa-miR-93-5p	SOX9
hsa-miR-93-5p	SRRM2
hsa-miR-93-5p	SSH1
hsa-miR-93-5p	TIMP2
hsa-miR-93-5p	TNF
hsa-miR-93-5p	TPPP
hsa-miR-93-5p	TUG1
hsa-miR-93-5p	ZNF318
hsa-miR-93-5p	ZNF354B
hsa-miR-93-5p	hsa-miR-20a-5p
hsa-miR-93-5p	hsa-miR-373-3p
hsa-miR-93-5p	hsa-miR-520a-3p
hsa-miR-93-5p	hsa-miR-524-3p
hsa-miR-96-3p	ACO1
hsa-miR-96-3p	DEPDC1B
hsa-miR-96-3p	HP1BP3
hsa-miR-96-3p	RBMS1
hsa-miR-96-3p	SLC12A2
hsa-miR-96-3p	TUG1
hsa-miR-96-5p	ACO1
hsa-miR-96-5p	ARL6
hsa-miR-96-5p	CHMP1B
hsa-miR-96-5p	DEPDC1B
hsa-miR-96-5p	HNF4A
hsa-miR-96-5p	HP1BP3
hsa-miR-96-5p	KHSRP
hsa-miR-96-5p	MAGI1
hsa-miR-96-5p	MALAT1
hsa-miR-96-5p	MECP2
hsa-miR-96-5p	NARS2
hsa-miR-96-5p	NME1-NME2
hsa-miR-96-5p	PLEKHA1

hsa-miR-96-5p	PPP2R3A
hsa-miR-96-5p	PRPF31
hsa-miR-96-5p	SH3BP4
hsa-miR-96-5p	hsa-miR-1271-5p
hsa-miR-96-5p	hsa-miR-34a-5p
hsa-mir-1226	CSDE1
hsa-mir-1226	NDUFV3
hsa-mir-1226	lnrCXCR4
hsa-mir-1228	CAT6
hsa-mir-149	KHSRP
hsa-mir-149	NORAD
hsa-mir-149	SBF2-AS1
hsa-mir-149	ZNF318
hsa-mir-16-1	HNF4A
hsa-mir-16-1	NORAD
hsa-mir-16-1	POLR2A
hsa-mir-16-1	hsa-miR-124-3p
hsa-mir-16-1	hsa-miR-16-5p
hsa-mir-16-1	hsa-miR-200b-3p
hsa-mir-16-1	hsa-miR-200c-3p
hsa-mir-16-1	hsa-miR-590-3p
hsa-mir-181a/181b/18	CARM1
hsa-mir-181a/181b/18	ND2
hsa-mir-193b	HP1BP3
hsa-mir-193b	PRPF31
hsa-mir-193b	SH3BP4
hsa-mir-222	GRB10
hsa-mir-222	MROH1
hsa-mir-222	NORAD
hsa-mir-222	SRRM2
hsa-mir-222	TINCR
hsa-mir-222	ZMYND11
hsa-mir-4508	SRRM2
hsa-mir-488-3p	ATF3
hsa-mir-744	FZR1
hsa-mir-744	INTS3
hsa-mir-744	L3MBTL2
hsa-mir-744	ND1
hsa-mir-744	NDE1
hsa-mir-744	NORAD
hsa-mir-744	PLCG1

hsa-mir-744	POLR2A
hsa-mir-744	SAMD4B
hsa-mir-744	SRRM2
hsa-mir-744	ZNF318
hsa-mir-93	POLR2A
hsa-mir-93	RPL30
hsa-mir-93	SAMD4B
hsa-mir-93	SH3BP4
SOX9	hsa-miR-215-5p
TIMP2	hsa-miR-19a-3p
TIMP2	hsa-miR-96-5p
hsa-let-7c-5p	IL10
hsa-miR-1-3p	MALAT1
hsa-miR-106a-5p	TIMP2
hsa-miR-122-5p	DRAIC
hsa-miR-124-3p	AR
hsa-miR-124-3p	NEAT1
hsa-miR-125b-5p	SH3BP4
hsa-miR-142-3p	MALAT1
hsa-miR-143-3p	MALAT1
hsa-miR-144-3p	TUG1
hsa-miR-17-5p	MALAT1
hsa-miR-196a-5p	ZMYND11
hsa-miR-19a-3p	MECP2
hsa-miR-19b-3p	MECP2
hsa-miR-200a-3p	MALAT1
hsa-miR-200c-3p	MALAT1
hsa-miR-204-5p	CXCL8
hsa-miR-204-5p	PPM1K
hsa-miR-205-5p	SNHG16
hsa-miR-206	MALAT1
hsa-miR-21-5p	PLEKHA1
hsa-miR-211-5p	NEAT1
hsa-miR-212-3p	MECP2
hsa-miR-212-3p	TUG1
hsa-miR-221-3p	PLAUR
hsa-miR-221-3p	TIMP2
hsa-miR-24-3p	HNF4A
hsa-miR-30b-5p	SOX9
hsa-miR-30d-5p	SOX9
hsa-miR-34a-5p	HNF4A
hsa-miR-448	NEAT1
hsa-miR-495-3p	SOX9

hsa-miR-509-3p	TNF
hsa-let-7a-5p	NEAT1
hsa-let-7b-5p	IL6
hsa-miR-124-3p	MALAT1
hsa-miR-125b-5p	MALAT1
hsa-miR-17-5p	TIMP2
hsa-miR-186-5p	TUG1
hsa-miR-200a-3p	SNHG16
hsa-miR-204-5p	MALAT1
hsa-miR-211-5p	PPM1K
hsa-miR-30a-5p	MALAT1
hsa-miR-590-3p	SOX9
TNF	IL10
hsa-miR-205-5p	NORAD
hsa-miR-155-5p	PTCH1
hsa-miR-130a-3p	SOX9
