# Supplementary material for: Decoding Non-Coding RNA Regulators in DITRA: From Genomic Insights to Potential Biomarkers and Therapeutic Targets
Source: Genes (Basel). 2025 Jun 27;16(7):753. doi: 10.3390/genes16070753 (PMC12295128; doi:10.3390/genes16070753)
Supplement: Supplementary file 1 [file genes-16-00753-s001.zip › Supplementary-TableS2.pdf]

node_name	MCC	DMNC	MNC	Degree	EPC	BottleNeck	EcCentricity	Closeness	Radiality	Betweenness	Stress	ClusteringCoefficient
**IL1A**	941268	0.18533	149	159	64.926	24	0.33333	340.33333	3.31226	31480.57582	307716	0.073
**IL1R1**	939816	0.20802	56	66	39.868	5	0.33333	293.5	3.13027	7971.97854	55078	0.09091
**IL1B**	939770	0.27292	50	52	33.181	6	0.33333	286.5	3.10345	4785.5521	39372	0.15913
**IL36RN**	937633	0.47067	25	30	19.118	4	0.33333	268.5	2.98084	4022.65391	23514	0.25747
**IL1RAP**	894710	0.10763	109	155	62.421	10	0.33333	337.5	3.29502	24429.91639	299550	0.02623
**IL1RL2**	806418	0.62819	16	26	15.635	6	0.33333	263.83333	2.94253	6347.47014	34796	0.21538
**IL36B**	730809	0.5262	17	20	17.346	4	0.33333	260.83333	2.93103	2797.45081	14054	0.34211
**IL36A**	725761	0.79029	12	13	14.947	1	0.33333	255.33333	2.89464	153.93492	1852	0.69231
**IL1RN**	489074	0.20103	48	78	36.286	8	0.33333	292.16667	3.06897	12638.90044	145786	0.04829
**IL36G**	443545	0.80471	13	14	13.384	1	0.33333	251.5	2.84674	120.19966	1134	0.69231
**IL1RL1**	203223	0.39639	24	33	23.115	3	0.33333	275.5	3.04981	4324.31639	24770	0.16667
**IL1R2**	126174	0.61922	16	16	14.32	1	0.33333	257.83333	2.91188	140.42644	2360	0.575
**IL2**	46099	0.39303	20	23	18.23	1	0.33333	262.33333	2.93678	746.32955	9316	0.25296
**hsa-miR-301b-3p**	12340	0.53309	32	32	34.791	1	0.33333	263.66667	2.91762	197.58357	15822	0.38911
**hsa-miR-301a-3p**	12336	0.52757	32	32	31.976	1	0.33333	263.5	2.91571	211.07613	15212	0.38508
**hsa-miR-130b-3p**	12276	0.42897	38	38	36.277	1	0.33333	269.33333	2.95977	386.571	26088	0.29587
**hsa-miR-19a-3p**	9065	0.32624	41	44	40.441	2	0.33333	274.83333	3	1013.29114	35528	0.19027
**SIGIRR**	5786	0.27679	22	24	18.727	1	0.33333	262.5	2.93487	1109.08362	11780	0.19203
**MIR17HG**	4931	0.1934	45	50	31.713	1	0.25	270.75	2.92337	1728.73693	25338	0.10286
**hsa-miR-454-3p**	4448	0.37748	33	33	33.256	4	0.33333	266.83333	2.95019	263.67951	21610	0.27273
**hsa-miR-130a-3p**	4404	0.38394	32	32	34.162	1	0.33333	263.33333	2.91379	272.33446	17110	0.28024
**MECP2**	3087	0.09415	89	105	55.318	2	0.33333	292	2.9636	3154.99207	99212	0.03626
**CSDE1**	2903	0.08307	112	150	65.208	2	0.33333	317.5	3.08429	9392.57822	290374	0.02282
**hsa-miR-4295**	2582	0.37086	28	28	31.91	1	0.33333	263.83333	2.93487	184.87669	16844	0.28307
**IRAK1**	2528	0.10671	98	122	47.138	19	0.33333	315.33333	3.16667	23313.57358	236362	0.03523
**SNHG16**	1505	0.18936	130	133	60.588	23	0.33333	316.83333	3.14176	9625.10031	142706	0.08464
**hsa-miR-363-3p**	1499	0.31857	22	25	25.282	1	0.33333	253	2.82184	147.30034	10382	0.20333
**hsa-miR-92a-3p**	1494	0.27602	23	29	29.211	5	0.25	256.66667	2.83908	288.17511	19178	0.14039
**FNBP4**	1030	0.10932	67	87	49.023	2	0.33333	281.83333	2.91571	3224.41936	104480	0.03716
**hsa-miR-20a-5p**	1000	0.31783	35	35	36.198	1	0.33333	265.83333	2.93103	310.0564	22274	0.22521
**hsa-miR-3666**	990	0.43602	20	20	23.444	1	0.33333	257.33333	2.8908	89.25557	7646	0.37368
**SLC12A2**	900	0.09977	71	87	51.218	3	0.33333	280.16667	2.89655	2534.31524	90088	0.03742
**NEAT1**	882	0.101	119	121	60.614	3	0.33333	299.66667	2.99042	5528.94697	131114	0.04697
**CHMP3**	840	0.14365	40	54	34.838	1	0.33333	256.66667	2.75287	1240.35504	36258	0.05311
**HNF4A**	808	0.06066	130	178	62.148	24	0.33333	329.33333	3.11303	17111.38357	332052	0.01517

**LTBP1**	677	0.121	52	71	45.585	2	0.33333	272.16667	2.8659	2899.31679	72632	0.04024
**GRB10**	671	0.11926	80	89	54.422	1	0.33333	282	2.90996	2153.65459	70012	0.05235
**hsa-miR-373-3p**	644	0.25681	37	41	38.23	2	0.33333	270.33333	2.95977	688.75852	37818	0.14512
**hsa-miR-106a-5p**	605	0.20117	47	52	44.293	2	0.33333	274.66667	2.96743	1272.82611	59124	0.10558
**MALAT1**	594	0.15648	155	156	64.132	22	0.33333	325.16667	3.15709	11656.68781	182544	0.06849
**TNF**	584	0.13824	44	55	40.048	2	0.33333	253	2.7069	626.43549	20668	0.05926
**CLIP1**	581	0.11111	49	74	48.002	1	0.33333	267	2.79502	1963.68737	68338	0.0311
**hsa-miR-181d-5p**	566	0.29844	36	36	36.893	1	0.25	268.58333	2.95785	328.6915	27022	0.20952
**TIMP2**	565	0.12483	54	75	43.851	15	0.33333	275.83333	2.89272	3379.48549	68076	0.03964
**ZNF354B**	540	0.17365	39	43	34.592	4	0.33333	260.16667	2.83525	695.5523	23534	0.09745
**hsa-miR-93-5p**	530	0.25465	37	37	39.264	9	0.33333	268.5	2.95402	300.61378	30668	0.17718
**hsa-miR-520a-3p**	523	0.32326	29	30	31.424	1	0.25	259.08333	2.86015	280.76838	16580	0.22759
**ND4**	506	0.41544	19	19	18.497	1	0.33333	241.83333	2.71648	87.2813	2134	0.36257
**ND2**	456	0.33897	23	23	20.903	1	0.33333	249.5	2.78927	190.01426	3790	0.27668
**hsa-miR-107**	447	0.2597	49	54	43.276	1	0.33333	276.66667	2.98276	1224.62374	55046	0.13557
**ND5**	442	0.34479	21	21	20.276	1	0.33333	248	2.77969	139.78165	3050	0.29048
**RNR2**	396	0.29615	16	16	11.983	1	0.25	233.5	2.61686	373.30043	4320	0.275
**TUG1**	385	0.17295	105	106	54.851	9	0.33333	298.66667	3.04406	4816.18929	85256	0.08482
**hsa-miR-524-3p**	358	0.36854	19	21	23.93	1	0.25	248	2.77778	114.69906	4042	0.2619
**SSH1**	343	0.172	40	45	35.658	3	0.33333	260.16667	2.82759	619.65273	19884	0.09192
**ZMYND11**	332	0.09928	70	78	47.427	10	0.33333	276.83333	2.89272	2155.74351	59122	0.04529
**TNPO2**	308	0.1207	85	97	47.212	27	0.33333	298.16667	3.06513	6861.91992	123800	0.0494
**ERCC4**	276	0.16777	33	41	30.384	1	0.33333	244.66667	2.66475	379.71844	14336	0.07805
**NORAD**	257	0.23954	37	38	25.009	2	0.33333	263.83333	2.90421	1623.83635	20208	0.15789
**hsa-let-7g-5p**	226	0.18125	41	49	40.282	2	0.33333	274.16667	2.97318	863.8638	50716	0.08503
**SRRM2**	219	0.08973	62	89	45.101	6	0.33333	280.16667	2.88889	4585.05814	131008	0.02554
**PLEKHA1**	211	0.08024	79	96	51.484	3	0.33333	284.33333	2.90996	3103.68361	98588	0.02961
**HP1BP3**	206	0.09182	64	84	46.542	3	0.33333	271.33333	2.80651	2661.48383	73056	0.03098
**hsa-miR-34a-5p**	204	0.26544	37	37	36.452	1	0.33333	271	2.98276	525.39062	25456	0.18468
**SH3BP4**	202	0.09922	52	76	45.088	6	0.33333	269.5	2.81609	2115.46474	70830	0.02877
**hsa-let-7c-5p**	189	0.14835	48	49	41.805	2	0.33333	275.66667	2.99042	840.27369	49604	0.09099
**hsa-miR-26b-5p**	185	0.12244	31	36	33.429	1	0.33333	270	2.9751	626.10697	33044	0.06667
**hsa-miR-181a-5p**	170	0.19063	28	28	29.255	1	0.25	260.91667	2.90038	221.98913	15976	0.1455
**USP38**	165	0.13975	56	59	44.696	5	0.33333	270.66667	2.89464	936.74924	37096	0.07656
**CXCL8**	164	0.14875	50	54	40.325	5	0.33333	265	2.84866	1446.55153	35594	0.08036
**DMXL2**	158	0.0924	55	61	43.2	1	0.33333	262.66667	2.79502	851.28065	34912	0.0459

**hsa-miR-181b-5p**	153	0.16029	30	31	30.788	1	0.25	262.75	2.90996	242.27474	22554	0.11183
**CARM1**	152	0.0996	50	60	36.662	1	0.33333	267.83333	2.85824	1809.3447	50538	0.0435
**RNVU1-7**	146	0.28717	16	16	13.704	1	0.33333	236	2.66092	112.6472	1604	0.26667
**PPP2R3A**	145	0.14696	36	39	32.86	1	0.33333	252.33333	2.76054	319.68354	11882	0.08772
**hsa-miR-30e-5p**	144	0.41155	33	33	32.525	2	0.33333	268	2.9636	235.21481	21648	0.29735
**hsa-miR-30b-5p**	144	0.4562	30	30	31.655	1	0.33333	266	2.95211	178.04885	18152	0.34023
**hsa-miR-186-5p**	142	0.16445	34	34	34.318	1	0.33333	269.5	2.97701	482.07356	27876	0.11765
**hsa-let-7e-5p**	142	0.12517	37	47	40.582	1	0.33333	271.33333	2.94828	730.60528	49424	0.05458
**hsa-miR-96-5p**	137	0.21307	23	24	28.413	7	0.33333	259.5	2.90038	151.96752	13552	0.15942
**hsa-miR-181c-5p**	136	0.18084	26	26	29.197	1	0.25	261.08333	2.90996	163.58098	16544	0.14154
**hsa-miR-17-5p**	134	0.16505	36	38	35.246	2	0.33333	267.83333	2.94253	802.30477	30742	0.10384
**TINCR**	134	0.34442	38	39	32.766	2	0.33333	270.5	2.97701	808.94722	20582	0.22537
**hsa-miR-122-5p**	133	0.14018	36	39	35.457	3	0.33333	272.83333	2.99617	1060.83543	39542	0.08367
**ARHGEF10**	130	0.13323	50	56	40.994	1	0.33333	265.33333	2.84483	919.25226	34130	0.06688
**RBMS1**	126	0.0963	51	89	49.517	1	0.33333	276	2.841	2646.54957	92208	0.01966
**hsa-miR-449a**	124	0.17492	31	33	33.891	2	0.33333	268.83333	2.97318	494.83907	26720	0.11364
**SENP5**	120	0.148	47	51	38.006	1	0.33333	260.5	2.80843	689.15702	22376	0.08078
**CDCP1**	119	0.11512	50	55	40.75	2	0.33333	267.5	2.87356	1449.0355	38504	0.05993
**ATF3**	118	0.11235	54	66	40.636	3	0.33333	271.16667	2.87356	1825.50091	49610	0.04615
**hsa-let-7d-5p**	118	0.13455	34	42	39.156	1	0.25	267.91667	2.9272	556.86631	40902	0.06388
**hsa-miR-4262**	117	0.24565	20	21	23.74	1	0.33333	253.16667	2.83908	105.6586	9104	0.19048
**hsa-let-7f-5p**	116	0.16279	36	46	38.646	1	0.33333	270.33333	2.94061	693.57437	44874	0.07053
**hsa-miR-495-3p**	115	0.13339	36	39	39.326	1	0.33333	267.83333	2.9387	451.15115	42016	0.07962
**hsa-miR-132-3p**	113	0.22457	22	23	25.83	1	0.33333	262.16667	2.93487	172.64716	11424	0.16996
**hsa-let-7a-5p**	113	0.13369	33	44	38.205	1	0.33333	268.16667	2.92337	596.53049	42082	0.05497
**hsa-miR-511-5p**	112	0.32048	15	17	18.404	2	0.25	245.58333	2.76628	142.40153	4508	0.23529
**ZNF318**	110	0.145	41	61	41.432	12	0.33333	259.83333	2.76245	1241.64106	40948	0.04372
**hsa-miR-141-3p**	100	0.25412	32	32	34.608	1	0.33333	267.33333	2.95977	344.98686	27310	0.18548
**MTND4P12**	92	0.2782	16	18	18.555	1	0.33333	231.83333	2.60536	82.65465	2562	0.20261
**hsa-miR-494-3p**	91	0.14367	29	32	33.78	2	0.25	265.41667	2.93678	356.56697	27232	0.09073
**ACO1**	91	0.09899	38	71	38.622	1	0.33333	264.83333	2.78161	2214.14758	56960	0.01972
**PTCH1**	84	0.1066	53	69	42.717	3	0.33333	270	2.84866	1798.8392	57704	0.03879
**hsa-miR-1271-5p**	82	0.3481	17	17	22.034	1	0.33333	259	2.92146	102.45241	6332	0.31618
**hsa-miR-200a-3p**	80	0.29485	26	26	29.252	1	0.33333	262.83333	2.93103	187.53686	16572	0.23077
**hsa-miR-330-3p**	80	0.2395	20	24	26.217	1	0.25	258	2.87548	335.09432	16698	0.1413
**hsa-miR-543**	79	0.15969	25	28	30.181	1	0.33333	263.5	2.93103	278.94368	20976	0.10317

**hsa-miR-214-3p**	77	0.19331	25	26	28.585	1	0.33333	262.16667	2.92337	253.9429	14128	0.14154
**hsa-miR-1179**	77	0.15012	23	30	34.226	1	0.33333	261.5	2.90038	288.87535	25556	0.07126
**hsa-miR-374a-5p**	76	0.15969	25	29	30.878	1	0.33333	262	2.90996	308.34092	19118	0.0936
**hsa-miR-19b-3p**	76	0.16464	23	29	30.134	3	0.25	264.25	2.93487	544.02436	21706	0.08374
**PPM1K**	76	0.11418	41	55	32.819	3	0.33333	259.16667	2.77778	2144.92364	71160	0.04242
**IL10**	73	0.1184	39	48	36.351	3	0.33333	248.16667	2.67816	497.2988	19558	0.05319
**hsa-miR-374b-5p**	73	0.18653	21	28	28.992	2	0.33333	260.16667	2.89272	289.25171	17210	0.0873
**hsa-miR-142-3p**	73	0.20822	23	24	28.168	1	0.33333	261.66667	2.92529	149.26574	14342	0.1558
**STS**	73	0.1401	39	46	35.331	1	0.33333	256.5	2.78161	655.26912	17998	0.0686
**SLC35C2**	73	0.13811	37	46	35.986	1	0.33333	255.66667	2.77203	615.76344	21286	0.06184
**hsa-miR-204-5p**	70	0.17433	23	29	27.629	2	0.33333	262	2.90996	469.32825	22944	0.08867
**hsa-miR-144-3p**	70	0.1681	25	27	30.882	1	0.33333	258.66667	2.87931	194.62691	17366	0.11396
**hsa-let-7b-5p**	69	0.16633	51	52	46.4	1	0.33333	277.83333	3.00383	894.36219	58812	0.1003
**MBD1**	69	0.12082	29	44	31.816	1	0.33333	248.5	2.69732	619.4132	25882	0.03911
**CHMP1B**	69	0.14363	32	35	28.618	1	0.33333	250.66667	2.7567	284.16876	11064	0.08739
**hsa-miR-548c-3p**	68	0.25665	23	23	26.569	1	0.33333	262.83333	2.94253	178.65485	14928	0.20949
**hsa-miR-203a-3p**	68	0.1258	26	34	35.783	1	0.33333	267.66667	2.95594	307.3441	34428	0.05882
**SRPRA**	68	0.10575	49	67	37.757	5	0.33333	265.33333	2.80268	2199.8902	47396	0.03573
**DEPDC1B**	68	0.11923	46	56	38.166	1	0.33333	259.83333	2.78161	969.17774	28524	0.05195
**SOX9**	67	0.10505	51	72	42.92	3	0.33333	272.5	2.8659	2071.71863	52356	0.03326
**POLR2A**	66	0.12687	45	51	36.589	1	0.33333	259.16667	2.7931	1016.54213	27118	0.06431
**KHSRP**	64	0.09053	47	71	40.165	2	0.33333	268.16667	2.81992	2368.34747	58696	0.02535
**hsa-miR-105-5p**	57	0.13061	29	34	31.865	1	0.33333	264.83333	2.92337	473.93102	30612	0.0713
**RPL30**	57	0.10775	29	40	26.225	1	0.33333	247.66667	2.70307	700.03897	19882	0.04359
**MAGI1**	57	0.09927	37	52	34.295	1	0.33333	254.83333	2.73946	803.30594	22746	0.03469
**hsa-miR-215-5p**	56	0.21538	16	18	20.993	1	0.25	252.91667	2.84674	201.72612	9504	0.15686
**CISTR**	55	0.10222	26	29	30.2	1	0.25	262.25	2.91188	292.6735	19532	0.06404
**L3MBTL2**	54	0.15967	20	38	24.864	1	0.33333	245.83333	2.68966	608.91429	20076	0.03841
**hsa-miR-23a-3p**	53	0.16392	21	28	31.659	1	0.33333	258.83333	2.87739	358.04148	25168	0.07672
**hsa-miR-30d-5p**	52	0.31777	31	31	32.614	1	0.33333	266.83333	2.95785	232.3069	21394	0.23441
**hsa-miR-106b-5p**	52	0.23091	37	37	38.687	2	0.33333	270.16667	2.97318	492.42588	28410	0.16066
**TMTC2**	52	0.12209	36	46	33.726	6	0.33333	257.83333	2.79693	897.67533	22704	0.05217
**TMEM201**	52	0.17298	26	34	25.874	3	0.33333	249.5	2.74713	454.83065	15960	0.08021
**ANCR**	51	0.40246	7	8	8.484	1	0.33333	218.33333	2.48851	24.29128	572	0.39286
**hsa-miR-149-5p**	49	0.14739	20	21	22.85	1	0.25	255.41667	2.86398	168.3524	9332	0.11429
**hsa-miR-103a-3p**	49	0.25931	5	41	38.626	1	0.25	266.41667	2.91379	761.67987	49560	0.01463

**hsa-let-7i-5p**	49	0.15948	46	47	41.826	2	0.33333	275.5	2.99617	699.99299	51390	0.09898
**PSMB5**	49	0.13811	37	48	32.355	1	0.33333	258.83333	2.80077	1194.64032	33634	0.05762
**IL6**	48	0.0983	50	58	41.817	3	0.33333	262	2.79885	812.22999	27672	0.04658
**hsa-miR-421**	48	0.25181	23	23	28.16	1	0.33333	258.66667	2.89464	125.5751	10342	0.20553
**hsa-miR-30c-5p**	48	0.33921	27	27	27.663	1	0.33333	262.66667	2.92529	148.95135	15178	0.26211
**hsa-miR-30a-5p**	48	0.29279	32	32	34.009	1	0.33333	267.33333	2.95977	234.73827	22756	0.21371
**hsa-miR-152-3p**	47	0.23648	14	17	19.444	1	0.25	251.41667	2.83333	126.33598	5612	0.15441
**SBF2-AS1**	47	0.19025	12	25	18.961	1	0.33333	240	2.67241	486.83685	5958	0.07333
**hsa-miR-34c-5p**	46	0.20836	26	28	26.724	1	0.33333	262.33333	2.91762	332.4248	16134	0.14021
**hsa-miR-212-3p**	46	0.29246	22	22	23.506	1	0.33333	259.83333	2.91188	206.41869	7652	0.24242
**hsa-miR-196a-5p**	46	0.15967	20	22	23.736	1	0.33333	256.66667	2.87548	133.87659	10030	0.11255
**hsa-miR-143-3p**	46	0.17051	16	24	28.966	1	0.33333	257	2.87165	180.49781	17220	0.06884
**hsa-miR-128-3p**	46	0.2099	31	31	32.407	2	0.33333	263	2.91379	333.70496	17574	0.15484
**hsa-miR-542-3p**	45	0.29378	13	16	22.104	1	0.33333	248	2.79885	66.25504	6482	0.19167
**NARS2**	45	0.14639	32	37	30.691	1	0.33333	250.33333	2.74521	304.30464	11646	0.07958
**SAMD4B**	45	0.11711	34	45	30.055	1	0.33333	250.66667	2.71839	935.30982	26084	0.04747
**RAD51-AS1**	44	0.13222	18	26	22.72	1	0.25	254.25	2.83142	538.81468	13904	0.05538
**hsa-miR-365a-3p**	42	0.16895	18	18	23.118	1	0.33333	259.33333	2.92146	111.709	7272	0.15033
**hsa-miR-33a-5p**	42	0.24269	13	17	21.275	1	0.25	246.58333	2.77778	80.56318	6710	0.13971
**hsa-miR-192-5p**	42	0.26715	17	17	21.989	1	0.33333	256.66667	2.89464	101.40739	6458	0.24265
**hsa-miR-134-5p**	41	0.19028	15	18	18.981	1	0.25	250.58333	2.81226	243.18672	8206	0.13072
**PLCG1**	41	0.14041	29	52	32.48	2	0.33333	256.16667	2.75479	1623.97308	41830	0.03318
**C17orf58**	40	0.16161	18	26	21.832	1	0.33333	242.5	2.69732	221.67671	7948	0.06769
**BEND4**	40	0.12607	25	37	30.777	1	0.33333	247.83333	2.71648	584.67445	17982	0.04505
**hsa-miR-1297**	39	0.15381	17	20	22.903	1	0.33333	253	2.841	107.5903	9714	0.1
**hsa-miR-125a-3p**	38	0.1187	21	21	20.523	1	0.25	257.08333	2.88314	229.39376	12612	0.1
**hsa-miR-1252-5p**	38	0.12488	18	26	27.54	1	0.33333	258	2.87548	269.75962	19368	0.05231
**NSRP1**	38	0.2089	22	22	19.596	1	0.33333	252.83333	2.83142	256.5396	5582	0.17316
**RNA5-8SN3**	38	0.21714	13	15	13.313	2	0.33333	246.5	2.78544	620.83113	6864	0.1619
**FENDRR**	38	0.19918	29	30	25.198	1	0.33333	256.66667	2.85249	384.03864	14228	0.14023
**hsa-miR-155-5p**	37	0.21396	14	21	22.745	1	0.25	249.41667	2.79119	113.42899	9588	0.09048
**hsa-miR-150-5p**	37	0.1916	13	18	20.434	1	0.25	245.75	2.75479	191.85527	9334	0.09804
**hsa-miR-519d-3p**	36	0.14021	15	23	28.427	1	0.25	251.91667	2.81609	127.40755	14756	0.05534
**hsa-miR-505-3p**	36	0.21396	14	18	19.685	1	0.25	248.91667	2.80077	120.85892	7602	0.12418
**hsa-miR-21-5p**	36	0.30023	23	23	23.35	1	0.33333	263.16667	2.94636	199.42117	11188	0.24506
**hsa-miR-20b-5p**	35	0.16191	17	24	26.667	1	0.33333	253.5	2.83142	124.56505	14632	0.07246

**PCDH19**	35	0.19652	20	43	31.328	1	0.33333	247.33333	2.68774	551.30551	24710	0.03654
**ND1**	35	0.26343	12	15	12.724	1	0.33333	231.83333	2.61686	107.76276	2550	0.17143
**hsa-miR-490-3p**	34	0.21031	15	17	24.258	1	0.33333	257.66667	2.90613	131.70773	9006	0.15441
**hsa-miR-485-5p**	34	0.17882	13	19	20.67	2	0.25	253.08333	2.84483	196.46057	10928	0.08187
**hsa-miR-27b-3p**	34	0.19093	9	26	26.553	1	0.33333	253.5	2.82375	185.20541	19826	0.03385
**hsa-miR-24-3p**	34	0.14572	17	17	19.218	1	0.33333	255	2.87548	83.72777	6490	0.13235
**hsa-miR-218-5p**	34	0.24269	13	21	22.118	1	0.33333	248	2.77969	148.02063	9352	0.09524
**hsa-miR-101-3p**	34	0.12534	22	42	39.387	2	0.33333	266.16667	2.90805	754.70648	47342	0.02904
**NDUFV3**	34	0.16968	11	33	19.72	3	0.33333	237.83333	2.61686	1079.78673	23676	0.01894
**AFAP1-AS1**	34	0.20437	13	13	15.469	1	0.33333	246.33333	2.79119	73.97419	1960	0.20513
**hsa-miR-613**	33	0.15381	17	20	22.7	1	0.33333	251.5	2.82375	112.75453	9762	0.1
**INTS3**	33	0.1149	22	33	22.664	2	0.33333	243.16667	2.67816	619.33107	15724	0.04356
**GTF2H2**	33	0.19099	18	23	20.095	1	0.33333	239.33333	2.67241	155.98483	4938	0.10277
**hsa-miR-221-3p**	32	0.20836	26	26	31.49	1	0.33333	261.83333	2.91954	137.94709	15798	0.16308
**hsa-miR-1-3p**	32	0.16154	16	28	27.233	1	0.33333	258.83333	2.87739	444.77963	20930	0.04762
**CRNDE**	32	0.23427	30	31	24.982	1	0.33333	256.16667	2.84291	349.1139	12262	0.16344
**hsa-miR-27a-3p**	31	0.2041	8	24	25.975	1	0.33333	251.5	2.80843	151.87384	16390	0.03623
**TPPP**	31	0.14071	19	30	22.762	4	0.33333	242.16667	2.67816	551.63445	10360	0.04828
**TEX101**	31	0.29279	14	15	14.63	2	0.33333	242.16667	2.73563	203.95091	2984	0.24762
**RNU1-106P**	31	0.25938	10	11	11.71	1	0.33333	236	2.68008	73.85034	1972	0.23636
**hsa-miR-382-5p**	30	0.15022	15	17	21.035	1	0.33333	250.83333	2.82759	71.50417	8138	0.11029
**NDE1**	30	0.18084	26	30	23.396	1	0.33333	250.33333	2.77203	552.97476	9306	0.10575
**hsa-miR-508-3p**	29	0.17882	13	14	18.518	2	0.33333	252.83333	2.86207	94.20813	5754	0.15385
**hsa-mir-181a/181b/18**	28	0.40246	7	9	11.322	1	0.33333	233.5	2.659	18.51424	714	0.30556
**hsa-miR-153-3p**	28	0.17562	12	18	21.559	1	0.33333	245.5	2.76245	70.54285	7096	0.07843
**hsa-miR-1185-5p**	28	0.20489	12	12	14.118	1	0.25	248.25	2.81609	68.51816	3188	0.21212
**lincTNS1**	28	0.19025	12	18	15.234	1	0.25	239.16667	2.6705	131.63248	7104	0.08497
**lnrCXCR4**	28	0.23866	9	15	13.606	5	0.25	239.33333	2.70115	290.40149	4298	0.10476
**PRMT2**	28	0.15426	18	22	19.338	1	0.33333	245	2.74138	248.51768	7716	0.09091
**NME1-NME2**	28	0.22058	11	13	11.741	1	0.33333	227.83333	2.57854	57.55113	1588	0.16667
**hsa-miR-422a**	27	0.19093	9	22	23.855	1	0.33333	252	2.82184	133.65592	14512	0.03896
**U1**	27	0.20362	11	12	8.385	3	0.25	231.91667	2.62835	225.51699	2658	0.18182
**hsa-miR-371a-3p**	26	0.18665	11	15	20.105	1	0.33333	252.66667	2.85632	77.94043	8458	0.10476
**PRPF31**	26	0.18423	20	24	24.522	1	0.33333	242.5	2.70498	245.51969	9014	0.1087
**hsa-miR-376c-3p**	25	0.2148	9	24	27.192	2	0.33333	252.5	2.81992	206.68565	18990	0.03261

**FZR1**	25	0.16154	16	25	24.127	1	0.33333	243.33333	2.71073	447.08222	9590	0.06
**hsa-miR-874-3p**	24	0.21948	10	12	11.69	2	0.33333	244.33333	2.77203	91.22457	2700	0.16667
**hsa-miR-205-5p**	24	0.25554	26	26	29.949	1	0.33333	265	2.95594	194.00076	15336	0.2
**hsa-miR-182-5p**	24	0.2041	8	18	21.169	1	0.25	243.91667	2.74138	106.96629	9318	0.04575
**hsa-miR-125a-5p**	24	0.19025	12	14	17.787	1	0.33333	250	2.8295	134.07836	7564	0.14286
**hsa-miR-526b-3p**	23	0.20489	12	17	23.265	1	0.33333	250.5	2.82375	83.58283	8450	0.10294
**hsa-miR-23b-3p**	23	0.21953	7	24	26.179	1	0.33333	252.33333	2.81801	274.10127	20086	0.02536
**ARL6**	23	0.14527	23	38	28.551	1	0.33333	236.83333	2.58621	246.06686	8464	0.04267
**hsa-miR-23c**	22	0.16098	12	16	20.719	2	0.33333	247.33333	2.79119	133.94697	8308	0.09167
**hsa-miR-140-5p**	22	0.20362	11	13	18.1	1	0.33333	254	2.87931	51.04151	5378	0.15385
**hsa-mir-222**	21	0.2864	9	10	16.485	2	0.25	236.33333	2.68008	64.91737	2446	0.26667
**hsa-miR-146a-5p**	21	0.2041	8	15	18.136	1	0.33333	249	2.81418	196.37643	8158	0.06667
**RNVU1-32**	21	0.26242	8	9	11.501	1	0.33333	234.16667	2.66667	32.30705	1524	0.25
**hsa-miR-448**	20	0.2041	8	24	26.961	1	0.25	252.91667	2.82375	174.1017	17900	0.02899
**hsa-miR-339-5p**	20	0.2148	9	13	16.001	1	0.25	246.83333	2.78927	78.45993	5438	0.11538
**hsa-miR-324-3p**	20	0.19093	9	13	12.188	1	0.25	241.25	2.7318	85.8998	5278	0.10256
**hsa-miR-16-1-3p**	20	0.2148	9	21	22.391	1	0.33333	248.16667	2.78161	176.0132	13250	0.04762
**RBM28**	20	0.16024	15	27	23.121	3	0.33333	236.16667	2.62069	453.42622	13926	0.04558
**GTF2F1**	20	0.19028	15	17	19.135	1	0.33333	241.5	2.72031	207.24393	4652	0.13971
**FAM214B**	20	0.14071	19	25	24.031	1	0.33333	234.66667	2.61111	201.04854	5698	0.07
**hsa-miR-432-5p**	19	0.19093	9	12	16.158	1	0.33333	240.5	2.72797	40.29061	4192	0.12121
**hsa-miR-18a-5p**	19	0.19093	9	16	17.173	1	0.33333	242.5	2.73563	168.2415	6238	0.06667
**hsa-miR-1224-5p**	19	0.23326	8	23	24.737	1	0.25	248.75	2.76054	201.45145	13204	0.03557
**hsa-miR-1193**	19	0.19953	10	15	16.362	1	0.25	243.91667	2.75479	151.50051	6100	0.09524
**hsa-miR-491-5p**	18	0.25546	13	13	16.202	1	0.33333	247.33333	2.80268	36.74979	3484	0.25641
**hsa-miR-18b-5p**	18	0.18665	11	19	25.444	2	0.33333	246.66667	2.77203	150.45922	10112	0.06433
**hsa-miR-16-5p**	18	0.20571	29	29	29.888	1	0.33333	261.33333	2.9023	248.6097	17410	0.15517
**hsa-let-7d-3p**	18	0.23866	9	13	17.224	1	0.33333	242.5	2.74713	56.91809	5324	0.12821
**PLAUR**	18	0.20368	22	26	23.674	1	0.33333	243.5	2.70881	105.03768	4066	0.12
**ZIC3**	18	0.20362	11	17	15.747	3	0.33333	230	2.58812	270.8657	5584	0.08824
**RNU1-148P**	18	0.2927	7	7	8.792	1	0.25	223.08333	2.54598	13.93837	308	0.38095
**hsa-miR-328-3p**	17	0.25931	5	14	18.46	1	0.33333	245.33333	2.77586	93.62167	6028	0.05495
**hsa-miR-2355-5p**	17	0.2927	7	8	8.678	1	0.25	229.58333	2.61494	27.7975	770	0.28571
**hsa-miR-149-3p**	17	0.19093	9	20	24.017	1	0.25	244.41667	2.72605	126.08878	12410	0.04211
**hsa-let-7f-2-3p**	17	0.23775	6	13	16.656	1	0.25	236	2.6705	39.97645	4374	0.07692
**hsa-miR-590-3p**	16	0.14944	47	47	44.739	5	0.33333	278.16667	3.02682	767.76155	59990	0.09621

**hsa-miR-580-3p**	16	0.38896	5	9	9.29	1	0.25	230.33333	2.61494	35.56416	1938	0.16667	
**hsa-miR-4282**	16	0.25931	5	13	17.012	1	0.25	240.66667	2.72414	46.96603	5554	0.05128	
**hsa-mir-744**	15	0.25931	5	12	9.742	2	0.25	230.66667	2.60536	87.9254	3594	0.07576	
**hsa-miR-744-5p**	15	0.30898	3	13	15.013	1	0.25	238.75	2.69157	117.15569	5158	0.0641	
**hsa-miR-346**	15	0.28529	6	9	11.453	1	0.25	239.66667	2.71648	44.67327	2314	0.19444	
**hsa-miR-30b-3p**	15	0.2842	4	16	18.577	1	0.25	243.91667	2.74904	70.85672	6504	0.04167	
**RABAC1**	15	0.16098	12	19	15.613	2	0.33333	236	2.64943	371.67209	6184	0.06433	
**CARD14**	15	0.22667	17	18	14.108	1	0.33333	231.5	2.60153	1068.2766	19280	0.18301	
**hsa-miR-335-5p**	14	0.20036	30	30	29.434	1	0.33333	264.66667	2.93678	413.49994	15612	0.14943	
**hsa-miR-323b-5p**	14	0.23775	6	10	13.502	1	0.25	234.75	2.66858	32.65298	2500	0.11111	
**hsa-miR-199a-3p**	14	0.25931	5	13	16.636	1	0.33333	233.33333	2.64176	46.5897	5264	0.05128	
**hsa-miR-106a-3p**	14	0.19093	9	13	18.941	1	0.25	244.75	2.77203	49.04019	4682	0.11538	
**SENCR**	14	0.2041	8	8	10.739	1	0.25	227.58333	2.59387	37.89032	782	0.25	
**RNA5-8SP6**	14	0.25611	7	7	8.72	1	0.33333	215.83333	2.4636	16.60583	400	0.33333	
**28S_RRNA**	14	0.2842	4	12	12.893	1	0.25	236.41667	2.66284	69.06156	4872	0.04545	
**hsa-miR-548t-5p**	13	0.2842	4	11	15.715	1	0.33333	239.66667	2.72222	28.41402	3850	0.05455	
**hsa-miR-519a-3p**	13	0.25931	5	10	14.48	1	0.25	235.16667	2.67241	19.484	2850	0.08889	
**hsa-miR-509-3p**	13	0.2148	9	12	14.57	1	0.33333	234.66667	2.66092	42.15697	3194	0.13636	
**hsa-miR-431-5p**	13	0.25931	5	10	15.339	1	0.25	239.91667	2.72797	53.05316	3910	0.08889	
**hsa-miR-206**	13	0.2842	4	15	18.035	1	0.25	240.25	2.71264	56.31514	5026	0.02857	
**hsa-miR-135a-5p**	13	0.30779	2	13	15.433	1	0.25	236.58333	2.67816	61.79984	5116	0.01282	
**hsa-miR-133a-3p**	13	0.21953	7	12	15.859	2	0.25	238.41667	2.69349	205.63782	3876	0.10606	
**MPPE1**	13	0.23775	6	19	14.804	1	0.33333	229.66667	2.57663	242.9927	5010	0.02924	
**HID1**	13	0.28529	6	15	14.601	1	0.33333	229.83333	2.59387	127.03362	4176	0.05714	
**hsa-mir-16-1**	12	0.19093	9	9	10.209	1	0.25	226.75	2.5728	29.05865	444	0.22222	
**hsa-miR-29a-3p**	12	0	1	12	16.337	1	0.25	236.33333	2.67816	40.90653	5110	0	
**hsa-miR-146b-5p**	12	0.24024	22	22	24.981	1	0.33333	264.33333	2.9636	329.86916	14764	0.19913	
**hsa-miR-125b-5p**	12	0.2842	4	16	18.719	1	0.25	244	2.74521	145.77309	9344	0.025	
**lincMTX2**	12	0.18911	25	25	25.645	2	0.33333	260.33333	2.90613	314.1998	13198	0.15	
**hsa-miR-573**	11	0.2842	4	9	9.166	1	0.25	235.33333	2.67816	33.55289	3246	0.08333	
**hsa-miR-548v**	11	0.23775	6	7	8.251	1	0.25	231.66667	2.63218	22.99857	1026	0.2381	
**hsa-miR-519c-3p**	11	0	1	11	15.145	1	0.25	236.16667	2.68008	31.4399	4742	0	
**hsa-miR-519b-3p**	11	0	1	11	17.123	1	0.25	236.16667	2.68008	31.98465	4438	0	
**hsa-miR-484**	11	0	1	11	12.657	1	0.25	231	2.60728	48.56585	3958	0	
**hsa-miR-328**	11	0.21953	7	10	13.988	1	0.25	233.33333	2.63602	29.26986	2736	0.13333	
**hsa-miR-325**	11	0.2842	4	9	12.192	1	0.25	236.16667	2.68774	22.18927	2168	0.11111	

**hsa-miR-19b-1-5p**	11	0.30898	3	13	17.229	1	0.33333	245	2.77586	59.86427	5194	0.0641
**hsa-miR-182-3p**	11	0.2041	8	11	16.499	1	0.25	240.41667	2.72989	21.61747	3208	0.12727
**hsa-miR-146a-3p**	11	0.23326	8	9	13.704	1	0.25	241.58333	2.73755	20.82313	2434	0.22222
**hsa-miR-1249-3p**	11	0.30898	3	10	14.392	1	0.33333	242.33333	2.7567	40.5228	3142	0.06667
**AC245014.3**	11	0.32413	5	6	6.779	1	0.25	226.41667	2.58812	28.3244	488	0.33333
**hsa-miR-92a-1-5p**	10	0.25931	5	7	9.4	1	0.25	230.91667	2.63602	16.64515	934	0.19048
**hsa-miR-452-3p**	10	0.21953	7	13	15.519	1	0.33333	238	2.6954	45.58427	4378	0.07692
**hsa-miR-4277**	10	0.30898	3	9	14.223	1	0.33333	237.33333	2.70307	28.8294	3480	0.05556
**hsa-miR-33a-3p**	10	0.25931	5	14	19.745	1	0.25	243	2.73946	57.7952	5040	0.10989
**hsa-miR-3118**	10	0.25931	5	11	11.652	2	0.25	235.91667	2.6705	116.41901	3210	0.09091
**hsa-miR-29b-3p**	10	0.30898	3	11	15.688	1	0.33333	232.33333	2.63793	23.43797	3172	0.03636
**hsa-miR-200b-3p**	10	0.14904	28	28	32.834	1	0.33333	262.66667	2.92146	264.05441	19298	0.11376
**hsa-miR-197-3p**	10	0.30779	2	10	13.152	1	0.33333	234.66667	2.66858	54.90376	3272	0.02222
**NRCP**	10	0.23775	6	6	10.435	1	0.25	228.25	2.6092	6.06219	412	0.33333
**TFAP2A-AS2**	10	0.10122	29	33	25.254	1	0.33333	259.16667	2.86207	783.78515	22148	0.05871
**hsa-miR-548u**	9	0.30898	3	8	12.086	1	0.33333	235.66667	2.68774	33.64638	2802	0.07143
**hsa-miR-425-5p**	9	0.16581	20	21	23.863	3	0.33333	256	2.87165	250.22305	12060	0.12857
**hsa-miR-211-5p**	9	0.16392	21	22	24.455	1	0.33333	261	2.92529	219.53235	13612	0.12554
**hsa-miR-1976**	9	0.30779	2	9	8.707	1	0.33333	232	2.64176	48.11193	3722	0.02778
**hsa-miR-103a-2-5p**	9	0.30779	2	9	11.103	1	0.25	231.16667	2.63027	18.88058	2030	0.02778
**AC053481.3**	9	0	1	9	9.348	1	0.25	192.16667	2.1705	6.94116	140	0
**lincZFP161**	9	0.30779	2	9	11.836	1	0.33333	227	2.58429	34.48334	2344	0.02778
**TBRG4**	9	0.2041	8	13	13.134	1	0.33333	228.66667	2.58812	79.75454	2312	0.08974
**BNC1**	9	0.20362	11	14	13.482	1	0.33333	238.83333	2.70115	101.2995	2646	0.13187
**hsa-mir-30a/30b/30c/**	8	0.37893	4	4	6.704	1	0.33333	224.66667	2.57663	0.63071	56	0.66667
**hsa-miR-93-3p**	8	0.30898	3	7	10.324	2	0.33333	232.66667	2.65709	35.72287	2266	0.09524
**hsa-miR-578**	8	0.30898	3	7	8.563	1	0.25	228.41667	2.60728	14.78673	1132	0.14286
**hsa-miR-561-3p**	8	0	1	8	10.813	1	0.25	229.66667	2.61686	21.7575	1912	0
**hsa-miR-519e-3p**	8	0	1	8	10.821	1	0.33333	232.66667	2.65326	23.79805	2110	0
**hsa-miR-513a-5p**	8	0.30898	3	7	9.243	1	0.33333	227.83333	2.60153	13.94315	1414	0.09524
**hsa-miR-3199**	8	0.2842	4	6	8.739	1	0.25	230.83333	2.63793	37.70887	2084	0.2
**hsa-miR-3120-3p**	8	0.25931	5	7	10.349	1	0.25	234	2.66092	15.51797	1370	0.19048
**hsa-miR-31-5p**	8	0.27679	22	22	26.211	1	0.33333	262	2.93678	146.34504	11064	0.22944
**hsa-miR-191-5p**	8	0.2842	4	6	11.43	1	0.25	241.25	2.75862	8.54926	872	0.2
**hsa-miR-1226-3p**	8	0.2842	4	6	10.141	1	0.25	233.41667	2.65326	22.18306	1864	0.2

**TENM1**	8	0.35765	13	17	16.865	1	0.33333	232	2.61111	91.94391	1874	0.20588
**MROH1**	8	0.30779	2	8	7.629	1	0.33333	212.5	2.42146	32.95971	910	0.03571
**BANCR**	8	0.30898	3	7	8.832	1	0.25	216.91667	2.47126	23.10244	526	0.14286
**CHI3L1**	7	0.2842	4	7	8.287	1	0.33333	218.16667	2.49042	15.66526	662	0.14286
**hsa-miR-877-3p**	7	0.21948	10	11	14.09	1	0.33333	230	2.61111	27.88666	1282	0.2
**hsa-miR-515-3p**	7	0.37893	4	7	9.812	1	0.33333	230.33333	2.63027	14.38731	758	0.19048
**hsa-miR-503-3p**	7	0.30779	2	9	12.291	1	0.25	228	2.58046	22.73485	1948	0.02778
**hsa-miR-4297**	7	0.30898	3	6	8.345	1	0.25	231.41667	2.62835	16.9453	1498	0.13333
**hsa-miR-4287**	7	0	1	7	8.763	1	0.25	224.16667	2.54598	25.61565	2210	0
**hsa-miR-29b-2-5p**	7	0	1	7	8.945	1	0.25	224	2.55556	20.388	1706	0
**hsa-miR-20b-3p**	7	0	1	7	9.5	1	0.25	224	2.55556	11.84751	1726	0
**hsa-miR-198**	7	0.30779	2	7	9.471	1	0.25	233.91667	2.66858	16.26164	1756	0.09524
**hsa-miR-1278**	7	0.2148	9	10	12.507	2	0.33333	243.83333	2.77395	47.96392	1972	0.2
**hsa-miR-124-3p**	7	0.13502	44	45	44.433	1	0.33333	276	3.00958	908.44281	56704	0.08485
**hsa-miR-1207-3p**	7	0.30779	2	7	8.782	1	0.25	225.41667	2.56322	15.04646	1090	0.04762
**hsa-miR-105-3p**	7	0	1	7	9.787	1	0.25	225	2.56705	12.87992	1708	0
**PSD4**	7	0.26242	8	13	11.421	1	0.33333	229	2.59195	148.6761	1878	0.11538
**SNORA73B**	7	0	1	7	6.434	1	0.33333	209.5	2.3908	35.19932	628	0
**RNU1-138P**	7	0.2842	4	5	7.066	1	0.33333	224.5	2.57088	127.76765	1004	0.3
**EEF1A1P9**	7	0	1	7	8.517	1	0.33333	212.33333	2.42337	16.77506	752	0
**hsa-miR-920**	6	0.30779	2	6	11.434	1	0.25	230.58333	2.6341	5.9082	706	0.13333
**hsa-miR-767-3p**	6	0.30779	2	6	9.487	1	0.25	230.5	2.6341	21.62236	1416	0.06667
**hsa-miR-612**	6	0.34989	8	8	12.235	1	0.33333	244.83333	2.7931	10.14142	1050	0.42857
**hsa-miR-556-3p**	6	0.30779	2	6	8.663	1	0.25	215.08333	2.43678	12.23889	950	0.06667
**hsa-miR-548k**	6	0	1	6	7.933	1	0.25	224.75	2.55364	15.35944	1340	0
**hsa-miR-516b-3p**	6	0	1	6	9.871	1	0.25	222.16667	2.52299	14.93751	1470	0
**hsa-miR-4635**	6	0	1	6	8.367	1	0.25	225	2.57088	19.4509	1408	0
**hsa-miR-4301**	6	0.30779	2	6	7.333	1	0.33333	225.16667	2.57471	35.26764	1124	0.06667
**hsa-miR-375-3p**	6	0.22992	13	13	17.846	1	0.33333	252	2.85632	53.20229	3962	0.23077
**hsa-miR-361-3p**	6	0	1	6	8.632	1	0.25	215	2.43678	32.04311	1126	0
**hsa-miR-216a-3p**	6	0	1	6	8.47	1	0.25	215.83333	2.4636	13.66943	700	0
**hsa-miR-200c-3p**	6	0.14414	24	24	29.572	2	0.33333	259	2.89464	202.04325	13778	0.11594
**hsa-miR-1915-3p**	6	0.30898	3	6	7.717	1	0.25	225.5	2.5613	21.56671	368	0.26667
**hsa-miR-17-3p**	6	0.23775	6	12	15.611	1	0.25	236.75	2.68391	51.50255	4156	0.09091
**hsa-miR-146b-3p**	6	0	1	6	8.189	1	0.25	221.08333	2.50383	21.08817	1226	0
**hsa-miR-106b-3p**	6	0	1	6	9.995	1	0.25	222.5	2.54215	8.32778	1210	0

**ATP8**	6	0.2842	4	4	6.622	1	0.33333	223	2.55747	2.41966	76	0.5	
**hsa-mir-93**	5	0.30779	2	5	5.8	1	0.25	212.66667	2.40613	9.10945	676	0.1	
**hsa-mir-149**	5	0.30779	2	5	6.067	1	0.25	204.83333	2.33716	7.80481	228	0.1	
**hsa-miR-518c-5p**	5	0.30779	2	5	8.12	1	0.25	214.16667	2.42146	9.66807	718	0.1	
**hsa-miR-432-3p**	5	0	1	5	7.192	1	0.25	220.33333	2.52107	8.09111	704	0	
**hsa-miR-4267**	5	0	1	5	7.206	1	0.33333	217.66667	2.49234	32.56529	940	0	
**hsa-miR-378a-5p**	5	0.30898	3	8	12.419	1	0.33333	232.66667	2.65326	25.1707	1920	0.07143	
**hsa-miR-3183**	5	0.30779	2	5	6.25	1	0.25	216.91667	2.48084	63.98637	460	0.2	
**hsa-miR-31-3p**	5	0	1	5	7.048	1	0.33333	218.33333	2.5	8.13012	888	0	
**hsa-miR-24-1-5p**	5	0.30898	3	4	6.008	1	0.25	216.91667	2.48659	3.94849	198	0.33333	
**hsa-miR-20a-3p**	5	0	1	5	9.167	1	0.25	223.58333	2.55939	6.32033	1014	0	
**hsa-miR-1914-3p**	5	0	1	5	8.194	1	0.25	222	2.52107	13.22398	1290	0	
**hsa-miR-1909-3p**	5	0.30779	2	5	6.459	1	0.25	220.66667	2.50383	7.14706	438	0.1	
**hsa-miR-150-3p**	5	0	1	5	6.846	1	0.25	220.91667	2.50575	10.04166	904	0	
**hsa-miR-1286**	5	0.30779	2	5	4.797	1	0.25	206.41667	2.36015	24.06654	372	0.2	
**hsa-miR-1225-3p**	5	0.30779	2	5	7.139	1	0.25	213.58333	2.42912	7.41307	660	0.1	
**hsa-miR-370**	5	0.30779	2	7	10.057	1	0.25	229.75	2.60728	15.34927	1540	0.09524	
**CCDC89**	5	0.2842	4	7	10.07	1	0.33333	235	2.68391	27.42819	498	0.14286	
**CAT2**	5	0	1	5	3.829	1	0.25	193.08333	2.18582	41.64595	326	0	
**hsa-mir-193b**	4	0	1	4	4.994	1	0.25	211.41667	2.39272	9.86692	484	0	
**hsa-mir-1226**	4	0.30779	2	4	5.308	1	0.25	218	2.47701	19.18529	714	0.16667	
**hsa-miR-96-3p**	4	0.30898	3	7	9.992	1	0.25	222.16667	2.53448	9.10896	1078	0.09524	
**hsa-miR-670-3p**	4	0	1	4	5.16	1	0.25	214.58333	2.43678	7.94307	548	0	
**hsa-miR-629-3p**	4	0	1	4	4.747	1	0.25	209.25	2.39655	5.54228	480	0	
**hsa-miR-620**	4	0	1	4	7.049	1	0.25	223.16667	2.54215	5.43034	740	0	
**hsa-miR-514b-3p**	4	0	1	4	5.669	1	0.25	208.25	2.36398	5.45229	500	0	
**hsa-miR-499a-5p**	4	0.15256	16	16	19.347	2	0.33333	252.16667	2.84674	109.52781	6790	0.14167	
**hsa-miR-492**	4	0.30779	2	4	6.307	1	0.25	219.25	2.50958	7.68872	570	0.16667	
**hsa-miR-4329**	4	0	1	4	8.425	1	0.25	223.41667	2.55939	6.75657	606	0	
**hsa-miR-4310**	4	0	1	4	7.085	1	0.25	213.33333	2.44253	6.3719	428	0	
**hsa-miR-4294**	4	0	1	4	6.595	1	0.25	222	2.54406	7.84556	578	0	
**hsa-miR-382-3p**	4	0	1	4	5.742	1	0.25	212.5	2.40996	18.03143	876	0	
**hsa-miR-34c-3p**	4	0	1	4	4.397	1	0.25	199.91667	2.26628	10.20686	392	0	
**hsa-miR-3116**	4	0.30898	3	7	9.143	1	0.25	227.58333	2.58621	25.69377	1720	0.09524	
**hsa-miR-24-2-5p**	4	0.30898	3	3	3.939	1	0.25	210.91667	2.42146	1.1532	54	0.66667	
**hsa-miR-2054**	4	0	1	4	6.795	1	0.25	226.83333	2.59962	4.0592	660	0	

**hsa-miR-193b-3p**	4	0.2374	21	21	26.95	1	0.33333	263.5	2.95785	190.08032	12466	0.2
**hsa-miR-18b-3p**	4	0	1	4	6.058	1	0.25	219	2.50958	4.68575	592	0
**hsa-miR-1285-3p**	4	0	1	4	7.089	1	0.25	222.41667	2.54789	4.44676	418	0
**hsa-miR-1270**	4	0	1	4	7.44	1	0.25	217	2.46552	7.04858	810	0
**GDF2**	4	0.2842	4	4	6.504	1	0.33333	221.16667	2.5364	8.18129	194	0.5
**hsa-mir-488-3p**	3	0.37893	4	7	10.998	1	0.33333	229	2.61494	5.79193	852	0.19048
**hsa-miR-887-3p**	3	0	1	3	4.992	1	0.25	190.66667	2.18774	2.04137	26	0
**hsa-miR-7153-5p**	3	0.30779	2	5	8.749	1	0.25	229.5	2.61303	7.46722	826	0.2
**hsa-miR-6832-5p**	3	0	1	3	2.758	1	0.25	184.33333	2.07854	6.40502	138	0
**hsa-miR-6733-3p**	3	0	1	3	4.939	1	0.25	206.41667	2.33908	7.93952	342	0
**hsa-miR-584-5p**	3	0.30779	2	3	4.116	1	0.25	211	2.41762	2.82473	192	0.33333
**hsa-miR-569**	3	0	1	3	5.88	1	0.25	219.16667	2.49042	5.47936	588	0
**hsa-miR-548d-5p**	3	0	1	3	3.981	1	0.25	202.08333	2.31801	1.51969	122	0
**hsa-miR-500b-3p**	3	0	1	3	4.264	1	0.25	194.75	2.20307	13.31492	196	0
**hsa-miR-4326**	3	0	1	3	5.867	1	0.25	219.83333	2.52299	2.19036	284	0
**hsa-miR-4284**	3	0	1	3	4.223	1	0.25	200.16667	2.29693	3.99334	122	0
**hsa-miR-4269**	3	0	1	3	7.377	1	0.25	221	2.51724	3.2834	484	0
**hsa-miR-4253**	3	0	1	3	5.38	1	0.25	219.5	2.51724	6.49379	404	0
**hsa-miR-3158-3p**	3	0	1	3	3.904	1	0.25	205.16667	2.35249	3.84868	298	0
**hsa-miR-184**	3	0.19025	12	13	16.79	1	0.33333	249.33333	2.82567	83.72122	3516	0.16667
**hsa-miR-1231**	3	0	1	3	5.834	1	0.25	218.83333	2.48659	4.85481	540	0
**hsa-miR-1229-3p**	3	0.30898	3	6	7.265	1	0.25	224.33333	2.55556	32.45632	664	0.13333
**hsa-miR-1228-3p**	3	0	1	3	5.008	1	0.25	220.83333	2.53257	1.89869	342	0
**hsa-miR-1197**	3	0.14125	20	23	23.92	2	0.33333	263	2.94444	744.864	18250	0.09091
**RNU4-2**	3	0	1	3	4.052	1	0.25	199	2.26054	27.77649	198	0
**PRRG3**	3	0.2842	4	5	8.419	1	0.33333	221.66667	2.53831	5.28412	312	0.3
**EML2**	3	0.31026	9	10	10.525	1	0.33333	233.33333	2.65326	54.02168	728	0.28889
**DRAIC**	3	0.32413	5	8	4.933	2	0.33333	214.66667	2.44636	70.4906	660	0.17857
**CCDC168**	3	0	1	3	3.413	1	0.33333	198	2.27395	5.1834	48	0
**AC091045.1**	3	0.30779	2	3	2.716	1	0.25	177.08333	1.97126	4.83935	28	0.33333
**hsa-mir-4508**	2	0	1	2	3.43	1	0.25	183.08333	2	3.31901	134	0
**hsa-mir-1228**	2	0	1	2	2.815	2	0.25	200.5	2.30077	108.74072	398	0
**hsa-miR-744-3p**	2	0	1	2	3.006	1	0.25	198.5	2.25096	2.09736	126	0
**hsa-miR-591**	2	0.25611	7	7	9.202	1	0.33333	243.66667	2.78352	72.4144	2024	0.33333
**hsa-miR-514a-3p**	2	0	1	2	3.461	1	0.25	193.41667	2.18774	0.99658	80	0
**hsa-miR-510-3p**	2	0	1	2	2.887	1	0.25	202	2.31801	3.56364	140	0

**hsa-miR-4695-5p**	2	0	1	2	2.885	1	0.25	188.08333	2.15709	1.20889	30	0
**hsa-miR-4695-3p**	2	0.30779	2	2	1.877	1	0.25	174	1.85824	0	0	1
**hsa-miR-4309**	2	0	1	2	4.455	1	0.25	209.83333	2.38697	1.58261	192	0
**hsa-miR-3529-3p**	2	0.30779	2	2	4.197	1	0.25	202.66667	2.29693	0	0	1
**hsa-miR-339-3p**	2	0.30779	2	2	4.307	1	0.25	210.25	2.39655	0	0	1
**hsa-miR-3198**	2	0	1	2	4.41	1	0.25	198.33333	2.25096	2.90862	246	0
**hsa-miR-296-3p**	2	0.21442	19	19	22.891	1	0.33333	252.83333	2.84291	141.01368	6888	0.18713
**hsa-miR-222-3p**	2	0.13566	21	23	25.149	1	0.33333	259.83333	2.90805	241.07974	17210	0.09486
**hsa-miR-1289**	2	0	1	2	3.073	1	0.25	197.58333	2.23946	1.13044	66	0
**hsa-miR-6893-3p**	2	0.30779	2	4	7.735	1	0.25	221.08333	2.51724	5.36725	472	0.16667
**OR8A1**	2	0	1	2	2.039	1	0.25	185.75	2.1341	1.78571	14	0
**MAGI1-IT1**	2	0	1	2	3.617	1	0.25	183.16667	2.10153	1.38333	8	0
**IL1beta-eRNA**	2	0.30779	2	2	3.758	1	0.25	190.41667	2.18774	0	0	1
**IL1beta-RBT46**	2	0.30779	2	2	2.69	1	0.25	190.41667	2.18774	0	0	1
**TG**	2	0.30779	2	4	7.259	1	0.33333	222	2.54598	3.21779	200	0.16667
**RNA5SP298**	2	0.30779	2	2	3.611	1	0.25	202.25	2.32567	0	0	1
**RN7SKP119**	2	0	1	2	3.713	1	0.25	211.08333	2.4272	1.43145	66	0
**MTND1P23**	2	0	1	2	3.1	1	0.25	201.75	2.31992	26.6467	356	0
**KRT6A**	2	0.28529	6	6	9.062	1	0.33333	230.66667	2.63793	18.36731	508	0.4
**AL353746.1**	2	0.30779	2	2	3.401	1	0.25	201.58333	2.31801	0	0	1
**GPR61**	2	0.30898	3	3	4.487	1	0.33333	220.66667	2.53448	2.09147	118	0.66667
**CCDC124**	2	0.2842	4	6	7.638	1	0.33333	222.16667	2.54023	29.37573	696	0.2
**CAT6**	2	0	1	2	1.537	1	0.25	176.41667	1.98659	8.2834	42	0
**C5orf60**	2	0	1	2	2.357	1	0.25	201.58333	2.31801	7.42249	128	0
**AC078899.1**	2	0.30779	2	2	4.659	1	0.25	203.75	2.34291	0	0	1
**kshv-miR-K12-9-3p**	1	0	1	1	1.85	1	0.25	191.5	2.16858	0	0	0
**kshv-miR-K12-4-3p**	1	0	1	1	3.189	1	0.25	199.83333	2.29693	0	0	0
**kshv-miR-K12-3-3p**	1	0	1	1	2.769	1	0.25	199.83333	2.29693	0	0	0
**kshv-miR-K12-11-3p**	1	0	1	1	2.594	1	0.25	199.83333	2.29693	0	0	0
**hsa-mir-93-3p**	1	0	1	1	2.132	1	0.25	191.5	2.16858	0	0	0
**hsa-mir-744-3p**	1	0	1	1	1.991	1	0.25	191.5	2.16858	0	0	0
**hsa-mir-328**	1	0	1	1	2.804	1	0.25	191.5	2.16858	0	0	0
**hsa-mir-193b-3p**	1	0	1	1	1.828	1	0.25	191.5	2.16858	0	0	0
**hsa-mir-142-3p**	1	0	1	1	2.851	1	0.25	191.5	2.16858	0	0	0
**hsa-mir-132**	1	0	1	1	2.297	1	0.25	191.5	2.16858	0	0	0
**hsa-mir-1226-3p**	1	0	1	1	2.564	1	0.25	191.5	2.16858	0	0	0

**hsa-miR-6868-5p**	1	0	1	1	1.633	1	0.25	179	2.05172	0	0	0
**hsa-miR-6862-3p**	1	0	1	1	1.441	1	0.25	173.75	1.94444	0	0	0
**hsa-miR-6784-3p**	1	0	1	1	1.652	1	0.25	173.75	1.94444	0	0	0
**hsa-miR-6716-5p**	1	0	1	1	1.126	1	0.25	172.75	1.93295	0	0	0
**hsa-miR-6511b-3p**	1	0	1	1	1.233	1	0.25	173.75	1.94444	0	0	0
**hsa-miR-6511a-3p**	1	0	1	1	1.36	1	0.25	173.75	1.94444	0	0	0
**hsa-miR-628-5p**	1	0	1	1	2.717	1	0.25	185.25	2.13218	0	0	0
**hsa-miR-619-3p**	1	0.2842	4	5	9.13	1	0.33333	236.66667	2.71073	11.08198	596	0.3
**hsa-miR-590-5p**	1	0.21858	17	18	20.645	3	0.33333	253.66667	2.85632	120.76716	8872	0.17647
**hsa-miR-589-5p**	1	0	1	2	4.492	1	0.25	213.16667	2.43295	1.19447	110	0
**hsa-miR-509-3-5p**	1	0	1	1	2.167	1	0.25	183.58333	2.07088	0	0	0
**hsa-miR-4732-5p**	1	0	1	1	1.437	1	0.25	172.75	1.93295	0	0	0
**hsa-miR-3667-3p**	1	0	1	1	1.738	1	0.25	185.25	2.13218	0	0	0
**hsa-miR-330-5p**	1	0.19953	10	11	13.326	2	0.33333	247.83333	2.81609	84.14078	3754	0.18182
**hsa-miR-218-2-3p**	1	0	1	1	2.174	1	0.25	183.58333	2.07088	0	0	0
**hsa-miR-181d-3p**	1	0	1	1	2.307	1	0.25	185.25	2.13218	0	0	0
**hsa-miR-122-3p**	1	0	1	1	1.996	1	0.25	183.58333	2.07088	0	0	0
**LOC107987013**	1	0	1	1	1.488	1	0.25	175.75	1.98276	0	0	0
**hsa-miR-326**	1	0.18423	20	21	23.906	1	0.33333	261	2.92912	259.0775	15254	0.14286
**AL158829.1**	1	0	1	1	1.691	1	0.25	175.75	1.98276	0	0	0
**AC005183.1**	1	0	1	1	2.643	1	0.25	199.83333	2.29693	0	0	0
**LOC101928266**	1	0	1	1	2.6	1	0.25	185.25	2.13218	0	0	0
**TNFRSF14**	1	0.30898	3	4	5.581	1	0.33333	221	2.53448	16.92235	400	0.33333
**SOWAHB**	1	0	1	1	2.512	1	0.25	200.91667	2.31418	0	0	0
**OPCML-IT1**	1	0	1	1	2.292	1	0.25	200.91667	2.31418	0	0	0
**LOC101928012**	1	0	1	1	2.754	1	0.25	200.91667	2.31418	0	0	0
**IL10RB-DT**	1	0	1	1	2.875	1	0.25	200.91667	2.31418	0	0	0
**ETS1-AS1**	1	0	1	1	1.647	1	0.25	173.75	1.94444	0	0	0
**AL049794**	1	0	1	1	2.372	1	0.25	199.83333	2.29693	0	0	0
**AC005477**	1	0	1	1	2.907	1	0.25	200.91667	2.31418	0	0	0
**hsa-miR-924**	0	0.23775	6	6	7.892	1	0.33333	234.33333	2.68008	14.7991	810	0.33333
**hsa-miR-641**	0	0.29157	8	8	12.197	2	0.33333	246.66667	2.81418	21.39161	1674	0.35714
**hsa-miR-632**	0	0.25931	5	5	7.056	1	0.33333	239.16667	2.73946	18.1772	804	0.4
**hsa-miR-625-5p**	0	0.32413	5	5	9.009	1	0.33333	240.83333	2.75862	7.30403	630	0.5
**hsa-miR-618**	0	0.25611	7	7	12.31	1	0.33333	244.5	2.7931	13.68795	1502	0.33333
**hsa-miR-617**	0	0.28529	6	6	11.893	1	0.33333	244.16667	2.7931	8.62457	1108	0.4

**hsa-miR-4292**	0	0.36588	7	7	11.035	1	0.33333	247.33333	2.82567	15.8962	1472	0.47619
**hsa-miR-4288**	0	0.25931	5	5	9.039	1	0.33333	245.16667	2.80843	6.97731	846	0.4
**hsa-miR-4256**	0	0.32413	5	5	6.448	2	0.33333	240.66667	2.7567	28.72298	776	0.5
**hsa-miR-423-3p**	0	0.23866	9	9	12.211	1	0.33333	247.16667	2.81609	31.7485	2444	0.27778
**hsa-miR-381-3p**	0	0.14939	26	26	29.656	1	0.33333	264.33333	2.94828	186.83854	21388	0.11692
**hsa-miR-34b-3p**	0	0.25452	11	11	13.929	1	0.33333	248.33333	2.82184	46.08522	2826	0.27273
**hsa-miR-342-3p**	0	0.16895	18	18	24.887	1	0.33333	256	2.88314	93.77361	9086	0.15033
**hsa-miR-3163**	0	0.16191	17	17	21.332	1	0.33333	255.16667	2.87739	77.91025	9840	0.14706
**hsa-miR-3133**	0	0.23943	10	10	12.942	1	0.33333	250.16667	2.84674	42.00489	3508	0.26667
**hsa-miR-29c-3p**	0	0.17562	12	12	17.53	1	0.33333	250.33333	2.841	33.62714	3890	0.18182
**hsa-miR-190b-5p**	0	0.23452	19	19	24.373	1	0.33333	260.83333	2.93487	70.07287	9176	0.20468
**hsa-miR-190a-5p**	0	0.26803	19	19	24.602	1	0.33333	260.5	2.93103	72.23454	8002	0.23392
**hsa-miR-147a**	0	0.28529	6	6	8.378	1	0.33333	237.66667	2.71839	8.24243	402	0.4
**hsa-miR-135b-5p**	0	0.20641	16	16	18.976	1	0.33333	253.16667	2.85824	66.74096	5776	0.19167
**hsa-miR-1275**	0	0.25931	5	5	7.989	1	0.33333	233.83333	2.67816	9.50987	620	0.4
**hsa-miR-1273f**	0	0.25611	7	7	10.622	1	0.33333	245.5	2.8046	28.2363	1318	0.33333
**hsa-miR-1237-3p**	0	0.21953	7	7	8.867	1	0.33333	243	2.77586	29.18909	1434	0.28571
**hsa-miR-1205**	0	0.23775	6	6	9.25	1	0.33333	236.5	2.70498	15.80857	908	0.33333
**hsa-miR-1180-3p**	0	0.32929	7	7	12.33	1	0.33333	246	2.81034	21.33216	1062	0.42857
**PLPP7**	0	0.2842	4	4	6.503	1	0.33333	221	2.53448	10.26988	170	0.5
**AR**	0	0.24906	196	197	72.953	32	0.5	358.5	3.38314	28628.03246	381496	0.10173
