# Supplementary material for: Decoding Non-Coding RNA Regulators in DITRA: From Genomic Insights to Potential Biomarkers and Therapeutic Targets
Source: Genes (Basel). 2025 Jun 27;16(7):753. doi: 10.3390/genes16070753 (PMC12295128; doi:10.3390/genes16070753)
Supplement: Supplementary file 1 [file genes-16-00753-s001.zip › Supplementary-TableS3.pdf]

Node name	Frequency
TINCR	11
hsa-miR-19a-3p	11
CSDE1	10
FNBP4	10
HNF4A	10
IL1A	10
IL1B	10
IL1R1	10
IL1RAP	10
IL1RN	10
IRAK1	10
MALAT1	10
MECP2	10
NEAT1	10
PLEKHA1	10
SNHG16	10
TNPO2	10
TUG1	10
hsa-let-7c-5p	10
hsa-let-7g-5p	10
hsa-miR-106a-5p	10
hsa-miR-122-5p	10
hsa-miR-17-5p	10
hsa-miR-30e-5p	10
hsa-miR-373-3p	10
hsa-miR-454-3p	10
AR	9
ATF3	9
CDCP1	9
CXCL8	9
GRB10	9
HP1BP3	9
IL1RL2	9
IL36RN	9
LTBP1	9
SH3BP4	9
SLC12A2	9
SRRM2	9
TIMP2	9
USP38	9
ZMYND11	9
hsa-let-7i-5p	9
hsa-miR-107	9
hsa-miR-130b-3p	9
hsa-miR-20a-5p	9
hsa-miR-590-3p	9
hsa-miR-93-5p	9

ARHGEF10	8
CLIP1	8
DMXL2	8
IL1RL1	8
KHSRP	8
PTCH1	8
RBMS1	8
SOX9	8
SRPRA	8
ZNF318	8
ZNF354B	8
hsa-let-7a-5p	8
hsa-let-7b-5p	8
hsa-let-7d-5p	8
hsa-let-7e-5p	8
hsa-let-7f-5p	8
hsa-miR-101-3p	8
hsa-miR-106b-5p	8
hsa-miR-124-3p	8
hsa-miR-181d-5p	8
hsa-miR-26b-5p	8
hsa-miR-34a-5p	8
hsa-miR-449a	8
CARM1	7
IL36B	7
IL6	7
MIR17HG	7
NORAD	7
PPM1K	7
SSH1	7
TMTC2	7
TNF	7
hsa-miR-130a-3p	7
hsa-miR-186-5p	7
hsa-miR-301a-3p	7
hsa-miR-301b-3p	7
hsa-miR-495-3p	7
