# Supplementary material for: Decoding Non-Coding RNA Regulators in DITRA: From Genomic Insights to Potential Biomarkers and Therapeutic Targets
Source: Genes (Basel). 2025 Jun 27;16(7):753. doi: 10.3390/genes16070753 (PMC12295128; doi:10.3390/genes16070753)
Supplement: Supplementary file 1 [file genes-16-00753-s001.zip › Supplementary-TableS4.pdf]

A/A	Name / NBCI ID / Type	Freq.	Targets	Biological Pathways	Related Disease	Ref.
1.	CSDE1<br>7812<br>Gene	10	TRIM33, CSDE1, lnc-SIKE1-2, SIKE1, RSBN1, AP4B1-AS1, OLFML3, NGF, TSPAN2, piR-58576 and NRAS	**Pathway:** Validated targets of C-MYC transcriptional repression<br>**Involved in:** regulation of translational initiation, male gonad development, stress granule assembly, positive regulation of translation and CRD-mediated mRNA stabilization	**Psoriasis**	[1]
2.	FNBP4<br>23360<br>Gene	10	FNBP4, lnc-FAM180B-4, lnc-FNBP4-1, NUP160, C1QTNF4, PSMC3, PTPRJ, ACP2, CELF1, SPI1, piR-56133-040 and AGBL2	**Pathway:** Actin Polymerization and regulation.<br>**Involved in:** Nuclear speck	**Psoriasis**	[2]
3.	HNF4A<br>3172<br>Gene	10	HNF4A, MIR3646, lnc-R3HDML-1, SRSF6, L3MBTL1, IFT52, FITM2, LINC01430, LINC01620, OSER1-DT, HNF4A-AS1, lnc-FITM2-5, lnc-SERINC3-4, R3HDML-AS1, SRSF6, IFT52, L3MBTL1, OSER1, MYBL2 and TTPAL	**Pathway:** Regulation of DNA-templated transcription<br>**Signaling pathway of:** AMPK, TGF-beta, ERK and MAPK.<br>**Activation pathway of:** LDLR, CYP27A1, GK, AKR1C4, C1QTNF5, NPC1L1, PCK1, G6PC1, PCSK9 and AFP.<br>**Involved in:** Regulation of transcription by RNA polymerase II, transcription by RNA polymerase II and lipid metabolic process	**Psoriasis**<br>**DITRA***<br>(MAPK)	[3]
4.	MECP2<br>4204<br>Gene	10	MECP2, lnc-MECP2-2, SNORA56, HCFC1, DKC1, IRAK1, TMEM187, PLXNA3, IKBKG, RENBP, ARHGAP4, ABCD1, piR-53177-056, SLC10A3, HSALNG0140828 and lnc-IRAK1-3	**Pathway:** Gene expression (Transcription) and Transcriptional Regulation<br>**Signaling pathway of:** ALK, MECP2 and GABA<br>**Activation pathway of:** GAMT, SST, CREB1, DEAF1, YBX1, TET1, GPRIN1, PRPF3, OPRK1, NEMF and DNMT1<br>**Involved in:** negative regulation of transcription by RNA polymerase II, behavioral fear response, response to hypoxia, startle response and nervous system process involved in regulation of systemic arterial blood pressure.	**Psoriasis**	[3]
5.	PLEKHA1		PLEKHA1, lnc-C10orf120-1 and ARMS2	**Pathway:** Metabolism	**Psoriasis**	

	59338<br>Gene	10		**Signaling pathway of:** CD28/T Cell and PI3K<br>**Involved in:** Luteinization, spermatogenesis, androgen metabolic process and estrogen metabolic process	**DITRA*<br>(CD28)**	[4]
6.	TNPO2<br>30000<br>Gene	10	TNPO2, lnc-ASNA1-1, TRMT1, ZNF136, ZNF44, ZNF791, ZNF799, ZNF625, ZNF564, ZNF700, ZNF763, ZNF844, WDR83OS, ZNF20, RPS6P25, ZNF442, TRIR, ZNF490, ZNF878, ZNF823, ZNF491, ZNF439, ZNF788P, ZNF441, ZNF440, RPL17P47, piR-41875 and FBXW9	**Pathway:** Protein transport<br>**Signaling pathway of:** AKT<br>**Involved in:** Protein import into nucleus and intracellular protein transport	**N/A**	
7.	TINCR<br>257000<br>Gene / lncRNA	10	SNRPEP4, TINCR, lnc-SAFB2-3, lnc-TINCR-1, piR-42491-110, RPL36, DUS3L,RANBP3, LONP1, lnc-SAFB-1, ZNRF4, piR-57460-331, TINCR and lnc-SAFB-1	**Pathway:** Mediate stabilization of differentiation mRNAs<br>Controls human epidermal differentiation by a posttrans-criptional mechanism, and Postdevelopmental somatic differentiation<br>**Activation Pathway of:** MAPK, Wnt/β-catenin and PTBP1/ATG5	**Psoriasis<br>DITRA***<br>(MAPK)	[5]
8.	MALAT1<br>378938<br>lncRNA	10	ANXA2, PARP1, JAG1, AXL, AKT1, RHOA, FAS, ADAM10, miR-17-5p, miR-22-3p, miR-23a-3p, miR-25-3p, miR-26a-5p, miR-26b-5p, miR-28-5p, miR-30a-5p, miR-32-5p and miR-20a-5p	**Signaling pathway of:** Interleukins (IL1, IL2, IL3, IL4, IL6, IL10, IL12, IL13, IL15, IL17, IL20, IL21 IL35, IL37, ILRSHC), MAPK family (MAPK1, MAPK2, MAPK3), TLR4, ALK, STAT, JAK-STAT, GHR and Gastrin-CREB via PKC<br>**Activation pathway of:** NFkB, MAPK1, MAP2K, MAPK3, STAT5, TLR4, TLR7 and TLR9.<br>**Involved in:** lncRNA-mediated post-transcriptional gene silencing and cellular response to hypoxia positive regulation of miRNA catabolic process.	**Psoriasis<br>DITRA***<br>(NFkB, MAPK, TLR4, JAK-STAT)	[6,7]
9.	NEAT1<br>283131<br>lncRNA	10	Ltb4r1, Snail2, DNA-PKcs, BCL-2, NF-kB, SRp40, COX-2, CTR1, Rsf-1, c-Myc, AURKB, let-7f-5p, let-7b-5p, let-7d-5p, let-7c-5p, let-7a-5p, miR-16-5p, miR-15a-5p, miR-17-5p and let-7e-5p	**Signaling pathway of:** Interleukins (IL1, IL2, IL3, IL4, IL6, IL7, IL10, IL12, IL13, IL15, IL20, IL21, IL37, ILRSHC), MAPK family (MAPK1, MAPK3), TLR4, ALK, STAT, JAK-STAT, GHR and Gastrin-CREB via PKC and MAPK.	**Psoriasis<br>DITRA***<br>(NFkB, MAPK, TLR4, JAK-STAT)	[8]

				**Activation pathway of:** NFkB, MAPK, MAPK1, MAPK3, MAPKK, p38MAPK, STAT STAT5, TLR4, TLR7, TLR8 and TLR9.<br>**Involved in:** lncRNA-mediated post-transcriptional gene silencing, nuclear body organization, miRNA-mediated post-transcriptional gene silencing and positive regulation of inflammatory response.		
10.	SNHG16<br>100507246<br>lncRNA	10	MUC5AC, SCD, MAPK1, mfn2, PRPS1, YY1, SLC2A4, LASP1, HMGB3, ITGA6, let-7c-5p, let-7b-5p, let-7d-5p, let-7f-5p, miR-16-5p, miR-15a-5p, miR-17-5p, miR-20a-5p, let-7a-5p and let-7e-5p	**Signaling pathway of:** Interleukins (IL1, IL2, IL3, IL4, IL6, IL7, IL10, IL12, IL13, IL15, IL20, IL21, IL37, ILRSHC), MAPK family (MAPK1, MAPK3), TLR4, ALK, STAT, JAK-STAT, GHR and Gastrin-CREB via PKC.<br>**Activation pathway of:** NFkB, MAPK, MAPK1, MAPK3, MAPKK, p38MAPK, STAT STAT5, TLR4, TLR7, TLR8 and TLR9.<br>**Involved in:** RNA processing	**Psoriasis**<br>**DITRA***<br>(NFkB, MAPK, TLR4, JAK-STAT)	[9,10]
11.	TUG1<br>55000<br>lncRNA	10	AURKA, ANPEP, TRPC6, Timp1, TNF, Bcl2l11, RORA, ZEB1, ROCK1, SOX4, miR-34a-5p, miR-27b-3p, miR-17-5p, miR-199a-3p, miR-221-3p, miR-29a-3p, miR-29b-3p, miR-187-3p, miR-132-3p and miR-129-5p	**Signaling pathway of:** Interleukins (IL2, IL4, IL6, IL7, IL10, IL12, IL13, IL20, IL21, IL35, ILRSHC), MAPK family (MAPK, MAPK1, MAPK3), TLR4, ALK, STAT, JAK-STAT, GHR and Gastrin-CREB via PKC.<br>**Activation pathway of:**NFkB, MAPK, MAP2K MAPK1, MAPK3, p38MAPK, STAT STAT5, TLR4, TLR7, TLR8 and TLR9.	**Psoriasis**<br>**R. Arthritis**<br>(TNF)<br>**DITRA***<br>(NFkB, MAPK, TLR4, JAK-STAT)	[11,12]
12.	MIR17HG<br>407975<br>lncRNA	7	HK1, miR-214-3p, miR-130a-3p, miR-302b-3p, miR-223-3p, miR-194-5p, miR-153-3p, miR-155-5p, miR-130b-3p, miR-301a-3p and miR-302a-3p	**Signaling pathway of:** Interleukins (IL2, IL3, IL4, IL6, IL7, IL10, IL12, IL13, IL15 IL17, IL20, IL21, IL27, IL35, IL37, ILRSHC), MAPK family (MAPK1, MAPK3, MAPK4, MAPK6), ALK, STAT, JAK-STAT, GHR and Gastrin-CREB via PKC.<br>**Activation pathway of:** NFkB, MAPK1, MAP2K, MAPK3, MAPKK, STAT STAT5, TLR4, TLR7 and TLR9.<br>**Involved in:** B cell homeostasis.	**Psoriasis**<br>**DITRA***<br>(NFkB, MAPK, TLR4, JAK-STAT)	[13]
13.	NORAD<br>647979<br>lncRNA	7	miR-22-3p, miR-26a-5p, miR-17-5p, miR-20a-5p, miR-93-5p, miR-25-3p, miR-32-5p, miR-30a-5p, miR-92a-3p, miR-26b-5p,	**Signaling pathway of:** Interleukins (IL1, IL2, IL3, IL4, IL6, IL7, IL10, IL12, IL15, IL13, IL17, IL20, IL21, IL27, IL35, ILRSHC), MAPK family (MAPK, MAPK4,	**Psoriasis**<br>**DITRA***<br>(NFkB, MAPK,	[14]

			FOXD1, RUNX3, PTEN, KLF5, MYCN, RHOA, NRF1, TGFB1 and ZEB1	MAPK6), TLR4, ALK, STAT, JAK-STAT and Gastrin-CREB via PKC.<br>**Activation pathway of:** NFkB, MAPK, MAP2K MAPK1, MAPK3, p38MAPK, STAT STAT5, TLR4, TLR7, TLR8 and TLR9.<br>**Involved in**: regulation of mRNA stability.	TLR4, JAK-STAT)	
14.	miR-19a-3p 406979 miRNA	11	MAP3K5, PKNOX1, PRMT5, SIVA1, TLR7, FRZB, IMPDH1, DPYSL2, SMAD4, ZBTB4, NR4A2, HOXA5, TF, MSMO1, VPS4B, TP53INP1, ERBB4, ESR1, ATXN1, SUZ12, AKT1, TNF, KAT2B, PHLPP1, CCND1, NEUROD1, RAB14, TUSC2, TGFBR2, SOCS3, BMPR2, ABCA1, ADRB1, PSAP, CUL5, MECP2, SOCS1, MYCN, MEF2D and TLR2	**Signaling pathway of**: Interleukins (IL4, IL13), MAPK, STAT and JAK-STAT.<br>**Activation pathway of:** MAPK, p38MAPK and STAT.<br>**Involved in:** negative regulation of transforming GFBR, miRNA-mediated post-transcriptional gene silencing, miRNA-mediated gene silencing by mRNA destabilization and B cell activation and negative regulation of cellular response to transforming growth factor beta stimulus.	**Psoriasis DITRA*** (MAPK, JAK-STAT)	[15]
15.	let-7c-5p 406885 miRNA	10	NR4A2, IGF1R, STAT3, MTPN, NUMB, DICER1, IL8, IL10, CASP3, AGO1, CDC25A, TNFRSF10B, TRIB2, PBX2, HSPA4, CEBPB, MYC, GPS1, COPS6, HMGA2, TTGFB1, RICTOR, MTOR, ITGB3, MAP4K3, IL6, IL6R, BCL2L1, TRIM71, COPS8, NRAS and MPL	**Signaling pathway of:** Interleukins (IL4, IL6, IL7, IL8, IL10, IL12, IL13), MAPK family (MAPK, MAPK1, MAPK3), STAT and JAK-STAT.<br>**Activation pathway of**: MAPK, MAP2K and STAT.<br>**Involved in:** miRNA-mediated post-transcriptional gene silencing and miRNA-mediated gene silencing by mRNA destabilization.	**Psoriasis DITRA*** (MAPK, JAK-STAT)	[16]
16.	let-7g-5p 406890 miRNA	10	CASP3, AGO1, BCL2L1, IGF2BP1, BMI1, FN1, MYC, IL13, CCL2, CCL5, TTGFB1, HMGA2, AKT2, THBS1, TNFRSF10B, COL1A2, CDKN2A, SMAD2, GAB2 and KRAS	**Signaling pathway of:** Interleukins (IL4, IL13), MAPK family (MAPK4, MAPK6), TGFB, STAT and JAK-STAT.<br>**Activation pathway of:** MAPK, MAP2K and STAT5.<br>Involved in: miRNA-mediated post-transcriptional gene silencing and negative regulation of inflammatory response.	**Psoriasis DITRA*** (MAPK, JAK-STAT)	[17]
17.	miR-106a-5p 406899 miRNA	10	RBL2, HMGA2, E2F1, RUNX3, APC, HIPK3, SLC2A3, RUNX1, ULK1, RB1, CDX2, PTEN, HIF1A, ERCC1, BCL10, CASP7, TTGFB2, FAS, CDKN1A, MYLIP, CXCL8, IL10, CYP19A1, CCND1, MFN2,	**Signaling pathway of:** Interleukins (IL4, IL10, IL13), MAPK, ALK, TGFB, STAT and JAK-STAT.<br>**Activation pathway of:** MAPK.<br>Involved in: miRNA-mediated post-transcriptional gene silencing, miRNA-mediated gene silencing by inhibition	**Psoriasis DITRA*** (MAPK, JAK-STAT)	[18]

			ATG7, VEGFA, APP, BMP2, SIRPA, ARID4B, RND3 and ATM	of translation and miRNA-mediated gene silencing by mRNA destabilization.		
18.	miR-122-5p 406906 miRNA	10	PRKRA, FUT8, CREB1, NOD2, ALDOA, EGLN3, NCAM1, XPO6, BAX, EGFR, SLC7A1, ZNF395, RAB6B, SPRY2, DSTYK, G6PC3, GYS1, TRIB1, BCL2L2, PRKAB1, MECP2, PDK4, FOXP1, NUMBL, CLIC4, RAB11FIP1, FUNDC2, ANXA11, HMOX1, ADAM10, AP3M2, RAC1, DUSP2, NFATC2IP, CDK4, FAM117B and PTPN1	**Signaling pathway of:** Interleukins (IL4, IL13, IL17), MAPK, TLR4 and Gastrin-CREB.<br>**Activation pathway of:** MAPK.<br>**Involved in:** miRNA-mediated post-transcriptional gene silencing.	**Psoriasis**<br>**R. Arthritis**<br>**A. Dermatitis**<br>**DITRA*** (MAPK, TLR4)	[19,20]
19.	miR-17-5p 406952 miRNA	10	PPP2R2A, TRIM8, TNF, TIMP2, CCND1, DNMT1, NTN4, RBL1, SMAD4, VDUP1, VEGFA, STAT3, ZBTB4, NEUROG1, CDKN1A, RBL2, PKD2, MMP2, SIRPA, APP, JAK1, GPR137B, ADAR, NEUROD1, CLOCK, ETV1, EPHA4, BCL2, ABCA1, RUNX1, CRIM1, KAT2B, MFN2, RND3, MAP3K12, HSPB2, WEE1, CCL1, NPTX1, TLR7, PIK3R1 and DNAJC27.	**Signaling pathway of:** Interleukins (IL2, IL4, IL6, IL7, IL10, IL13, IL15, IL21, IL27, IL35), MAPK, ANK, TGFB, and JAK-STAT.<br>**Activation pathway of:** MAPK.<br>**Involved in:** cysteine-type endopeptidase activity involved in apoptotic process, cellular senescence, intrinsic apoptotic signaling pathway, mitochondrial membrane permeability involved in apoptotic process, hydrogen peroxide-mediated programmed cell death, phagocytosis, cytokine production involved in inflammatory response, hydrogen peroxide-mediated programmed cell death, connective tissue replacement involved in inflammatory response wound healing, miRNA-mediated post-transcriptional gene silencing, miRNA-mediated gene silencing by inhibition of translation and cellular response to lipopolysaccharide and cellular response to hypoxia.	**Psoriasis**<br>**DITRA*** (MAPK, JAK-STAT)	[21]
20.	miR-30e-5p 407034 miRNA	10	MBNL2, MTTP, TUSC3, GNAI2, TP53, ADRB2, BMI1, HELZ, P4HA1, BNIP3L, PLCG1, ATG5, MBNL1, NOTCH1, MYBL2, NFKBIA, CASP3, ADRB1, MBNL3, UBE2I and SNAI1	**Involved in:** miRNA-mediated post-transcriptional gene silencing and regulation of adenylate cyclase-inhibiting adrenergic receptor signaling pathway.	**Psoriasis**	[21]

21.	miR-373-3p 442918 miRNA	10	BTG1, RASSF1, DKK1, MBD2, MTOR, SIRT1, RAD23B, TXNIP, RABEP1, PIK3CA, VEGFA, JAK1, XPA, NFIB, CD44, LATS2, RAD52, RECK, TGFBR2, CXCR4, MYC, TNFAIP1, LEFTY2, LEFTY1, IRF9 and MRE11	**Signaling pathway of:** TGFB and JAK-STAT. **Activation pathway of:** MAPK. **Involved in:** miRNA-mediated post-transcriptional gene silencing and miRNA-mediated gene silencing by mRNA destabilization.	**DITRA*** (MAPK, JAK-STAT)	
22.	miR-454-3p 768216 miRNA	10	CXCL12 and SMAD4	**Involved in:** miRNA-mediated post-transcriptional gene silencing and negative regulation of MAPK cascade.	**. Arthritis DITRA*** (MAPK)	[22]
23.	let-7i-5p 406891 miRNA	9	CXCL8, ASCL1, CD86, NEUROG1, TLR4, HMGB1, IL13, IGF1, AURKB, IL2, BMP4, GPS1, COPS8, SOCS1, COPS6 and AGO1	**Signaling pathway of:** Interleukins (IL4, IL10, IL13), MAPK, TLR4 and JAK-STAT. **Activation pathway of:** NFkB, TLR7, TLR8, TLR9 and MAPK. **Involved in:** negative regulation of gene expression, miRNA-mediated post-transcriptional gene silencing and miRNA-mediated gene silencing by inhibition of translation.	**Psoriasis A. Dermatitis DITRA*** (MAPK, TLR4 JAK-STAT)	[19,21]
24.	miR-107 406901 miRNA	9	DICER1, SERBP1, LIN28A, CHGA, CDK8, AXIN2, RAB1B, PTEN, JAK1, LATS2, ARNT, NFIA, VEGFA, HMGA2, CYP2C19, CDK6, SNCG, CAV1, BACE1, DAPK1, PLAG1, GRN, RAD51, SALL4, SH3GL2, SUZ12, AIP, CPEB1, AGO1, CDC42, AGO3, FBXW7, HIF1A, IL6, NOTCH2, CHRM1, MYB, NPPA and LRP1	**Signaling pathway of:** Interleukins (IL4, IL6, IL12, IL13) and MAPK. **Activation pathway of:** MAPK1 and MAPK3. **Involved in:** miRNA-mediated post-transcriptional gene silencing and miRNA-mediated gene silencing by inhibition of translation.	**Psoriasis DITRA*** (MAPK)	[23]
25.	miR-130b-3p 406920 miRNA	9	ABCA1, ERBB2, SAV1, PPARGC1A, NKD2, IRF1, IGF1, LDLR, TP53INP1, RUNX3, DICER1, ZEB1, DLL1, MMP2, UVRAG, MST1, PDGFRA, PPARG, FMR1, CYP2C9, CSF1, CMPK1, STAT3, ZBTB4, CYLD, SNAI3, SMAD4, PPARA, CCNA2, PTEN, ITGB1 and CCDC6	**Signaling pathway of:** Interleukins (IL4, IL13), STAT and JAK-STAT. **Activation pathway of:** MAPK. **Involved in:** inflammatory response, negative regulation of macrophage derived foam cell differentiation, negative regulation of monooxygenase activity, miRNA-mediated post-transcriptional gene	**Psoriasis A. Dermatitis DITRA*** (MAPK, JAK-STAT)	[19]

				silencing and miRNA-mediated gene silencing by mRNA destabilization.		
26.	miR-20a-5p 406982 miRNA	9	TTGFB2, TBC1D2, MEF2D, MAP3K5, NFKBIB, RGS5, REST, NRAS, SIRPA, NBEA, MAP3K12, ATG16L1, PTEN, LDLR, DUSP2, PPP2R2A, PKD1, NEUROG1, GJA1, E2F3, TCEAL1, PRKG1, PPARG, OSM, CCL1, EPHA4, MYC, RB1CC1, RBL1, IRF2, NPTX1, PURA, APP, BCL2, VEGFA, KIT, CCND2, ETV1, ARHGAP12, RB1, WEE1, BNIP2, RUNX1 and EGLN3	**Signaling pathway of:** Interleukins (IL4, IL13), MAPK, ALK, BMP and JAK-STAT. **Activation pathway of:** NFkB, TLR4, TLR7, TLR8, TLR9 and MAPK. **Involved in:** negative regulation of cytokine-mediated signaling pathway, inflammatory response, negative regulation of gene expression, miRNA-mediated post-transcriptional gene silencing, miRNA-mediated gene silencing by inhibition of translation, miRNA-mediated gene silencing by mRNA destabilization, cellular response to hypoxia, negative regulation of protein serine/threonine kinase activity and negative regulation of p38MAPK cascade.	**Psoriasis DITRA*** (NFkB, MAPK, JAK-STAT)	[24]
27.	miR-590-3p 693175 miRNA	9	CREB5, TGFB1, MACC1, RECK, SMAD3, ATF3, TGFBR1, RB1, APC, SMAD7, NFKB1, FOSL2, ZEB1, ZEB2, OLR1, S100A10, BTG2, ILF3, LPL, ZNF143, EEF1E1, CHL1 and TGFBR2	**Activation pathway of:** NFkB, MAPK1, MAPK3 **Involved in:** positive regulation of cell population proliferation, miRNA-mediated post-transcriptional gene silencing, miRNA-mediated gene silencing by inhibition of translation, positive regulation of cell cycle and negative regulation of inflammatory response	**Psoriasis DITRA*** (NFkB, MAPK)	[25]
28.	miR-93-5p 407050 miRNA	9	TBC1D2, SLC2A4, ANG, F3, RAB11FIP1, ZBTB4, PTENP1, VEGFA, PURA, ZNRF3, FOXA1, ABCA1, PTEN, TP53INP1, TGFBR2, RYR2, LDLR, MAPK9, MMP3, LATS2, RPS6KA4, CERS2, TUSC2, SMAD7, FOXO3, STK11, E2F1, CDKN1A, KAT2B, ITGB8, RHOC, ATG16L1, PHLPP2 and CXCL8	**Signaling pathway of:** Interleukins (IL4, IL13), BMP and TGFB. **Activation pathway of:** MAPK **Involved in:** negative regulation of cytokine production, miRNA-mediated post-transcriptional gene silencing, miRNA-mediated gene silencing by mRNA destabilization, negative regulation of cytokine production involved in inflammatory response and negative regulation of voltage-gated calcium channel activity.	**Psoriasis DITRA*** (MAPK)	[26]

29.	let-7a-5p 406881 miRNA	8	E2F1, E2F2, MAP4K4, PRDM1, RAB40C, IGF2BP1, NR1I2, EGFR, APP, CASP8, IL6, PAK1, EZH2, HMGA2, DICER1, HMGA1, CDC34, KRAS, MYC, FOXA1, PKM, TGFBR3, WNT1, CCND2, HRAS, TMED7, RAVER2, CDKN1A, CASP3, AGO4, LIN28B, AURKB, IGF2, LIN28A, MPL, CDK6, UHRF2 and HAS2	**Signaling pathway of:** Interleukins (IL4, IL6, IL13), MAPK family (MAPK4, MAPK6), TLR4, ALK, STAT, TGFB, JAK-STAT, and Gastrin-CREB via PKC. **Activation pathway of:** NFkB, MAP2K and MAPK. **Involved in**: miRNA-mediated post-transcriptional gene silencing, miRNA-mediated gene silencing by inhibition of translation and miRNA-mediated gene silencing by mRNA destabilization.	**Psoriasis** **A. Dermatitis** **DITRA*** (NFkB, TLR4, MAPK)	[17,27]
30.	let-7b-5p 406884 miRNA	8	CDK6, AGO1, CPEB1, CDC34, HRAS, NR2E1, CCND1, IGF1R, TGFBR1, CTHRC1, RPIA, CASP3, ACTG1, AKT2, IRS2, E2F2, LIN28B, CCNA2, HMGA2, CDC25A, IGF2BP2, RDH10, CYP2J2, PRDM1, LGR4, TNFRSF10B, IL6, NRAS, MTPN, IFNB1, LIN28A, TLR4, EZH2, HMGA1, ANAPC1, CPEB3 and CCND2	**Signaling pathway of**: MAPK family (MAPK1, MAPK3), TGFB, JAK-STAT and Gastrin-CREB via PKC. **Activation pathway of:** MAPK. **Involved in:** negative regulation of autophagy, miRNA-mediated post-transcriptional gene silencing, miRNA-mediated gene silencing by mRNA destabilization, negative regulation of cysteine-type endopeptidase activity involved in apoptotic process, positive regulation of ERK1 and ERK2 cascade, antibacterial innate immune response and negative regulation of hydrogen peroxide-mediated programmed cell death	**Psoriasis** **DITRA*** (NFkB, TLR4, MAPK)	[28]
31.	let-7d-5p 406886 miRNA	8	APP, TNFRSF10B, IL13, AGO1, SLC11A2, CRYGEP, MPL, COL3A1, DICER1 and HMGA2	**Involved in:** miRNA-mediated post-transcriptional gene silencing.	**Psoriasis**	[21]
32.	let-7e-5p 406887 miRNA	8	IGF1, IGF1R, LIN28A, AGO1, TNFRSF10B, AURKB, MMP9, PLK1, TNFAIP3, HMGA2, WNT1, GPS1, COPS6, COPS8, MYCN, MPL, CCND1, SMC1A, EIF3J, ARID3A, EZH2 and FASLG	**Signaling pathway of:** Interleukins (IL4, IL13), STAT and MAPK. **Involved in:** miRNA-mediated post-transcriptional gene silencing, miRNA-mediated gene silencing by mRNA destabilization.	**Psoriasis** **DITRA*** (MAPK)	[21]
33.	let-7f-5p 406889 miRNA	8	IL23R, IL13, IL6, CCL7, CCND1, MPL, DYRK2, IRS2, IGF1R, AGO1, PRDM1, CYP19A1, KLK10, MYH9, KLK6, POSTN, GPS1, ELF4, INSR, SOCS3, COPS6 and COPS8	**Signaling pathway of:** Interleukins (IL4, IL6, IL13, IL17), TGFB, TOR, STAT and JAK-STAT. **Involved in:** miRNA-mediated post-transcriptional gene silencing, negative regulation of apoptotic process,	**Psoriasis** **DITRA*** (JAK-STAT)	[19]

				negative regulation of phosphatidylinositol 3-kinase/protein kinase B signal transduction		
34.	miR-101-3p 406893 miRNA	8	CDK8, CTNNB1, SUZ12, SRF, PRDM1, PIK3CB, MTOR, SNHG1, ZEB2, ATG4D, FBN2, PRKDC, ATM, NOTCH1, SOX9, EYA1, KLF6, PTGS2, RANBP9, STMN1, ATXN1, CDH5, MITF, CFTR, GRSF1, MET, VHL, ITGA3, PIM1, MCL1, ARID1A, FOS, MYCN, EZH2, RHOA, ATP5F1B, CUL3, VEGFC, RAB5A, EED, CPEB1, MEIS1, APP, FMR1 and NLK	**Signaling pathway of:** Interleukins (IL1, IL4, IL13), MAPK family (MAPK, MAPK1, MAPK3), TLR4, TGFB, STAT and JAK-STAT.<br>Activation pathway of: MAPK.<br>**Involved in:** miRNA-mediated post-transcriptional gene silencing, miRNA-mediated gene silencing by inhibition of translation, protein stabilization,<br>negative regulation of SMAD protein signal transduction, negative regulation of necroptotic process and negative regulation of chemokine-mediated signaling pathway.	**Psoriasis DITRA*** (MAPK, TLR4, JAK-STAT)	[29]
35.	miR-106b-5p 406900 miRNA	8	TNFSF11, CRIM1, NIBAN1, MAPK9, EPHA4, SETD2, PURA, E2F5, ATG16L1, WEE1, CASP8, FYN, STAT3, TRIM8, RUNX3, RB1, RBL2, RBL1, APP, TCEAL1, CCND1, PKD2, CXCL8, CDKN1A, JAK1, ZBTB4, APC, RHOC, TNFRSF10A, NTN4, HIF1A, MMP2, PRRX1, CCND2, EOMES, E2F3, PTEN, CASP7, ITCH	**Signaling pathway of:** Interleukins (IL4, IL6, IL8, IL13, IL15, IL20, IL21, IL27 IL35), ALK TLR4, STAT and JAK-STAT.<br>**Activation pathway of:** MAPK.<br>**Involved in:** miRNA-mediated post-transcriptional gene silencing, miRNA-mediated gene silencing by inhibition of translation, miRNA-mediated gene silencing by mRNA destabilization and negative regulation of peptidyl-tyrosine phosphorylation.	**Psoriasis DITRA*** (MAPK, TLR4, JAK-STAT)	[21]
36.	miR-124-3p 406909 miRNA	8	SIRT1, IL6R, HNRNPA2B1, RAC1, SLC16A1, AHR, MTDH, ROCK2, CAPNS1, CD274, ITGB3, NR3C2, REST, NFKBIZ, BACE1, AKT2, PEA15, KLF6, CTDSP1, CCL2, GRIA3, ROCK1, DNMT3B, MECP2, CAV1, RGS4, FXN, NFATC1, CCN2, CAMTA1, E2F6, RDH10, EZH2, AR, B4GALT1, XRCC6, HMGA1, CEBPA, LAMC1 and CDK2	**Signaling pathway of:** Interleukins (IL2, IL3, IL4, IL6, IL13, IL15), ALK, MAPK and JAK-STAT<br>**Activation pathway of:** MAPK<br>**Involved in:** miRNA-mediated post-transcriptional gene silencing	**Psoriasis DITRA*** (MAPK, JAK-STAT)	[30]
37.	miR-181d-5p 574457 miRNA	8	HRAS, DUSP10, MGMT, MALT1, MEG3, IL1B, RAP1B, BCL2 and CCR1	**Signaling pathway of:** Interleukins (IL1B, IL4, IL10, IL12, IL13), MAPK family (MAPK1, MAPK3), JAK-STAT and Gastrin-CREB via PKC.	**Psoriasis DITRA*** (MAPK,	[31]

				**Activation pathway of:** MAPK and MAP2K. **Involved in:** positive regulation of cell migration, miRNA-mediated post-transcriptional gene silencing, miRNA-mediated gene silencing by inhibition of translation, miRNA-mediated gene silencing by mRNA destabilization andpositive regulation of p38MAPK cascade.	JAK-STAT)	
38.	miR-26b-5p 407017 miRNA	8	PTEN, ESR1, SMAD1, EZH2, FH, ULK2, S100A7, TRAF5, USP9X, MIEN1, KPNA2, TAB1, CCN2, GATA4, ST8SIA4, PLOD2, MT-CO2, IGF1R, JAG1, IGF1, CHORDC1, NAMPT, HGF, ARL4C, LARP1, NR2C2, RB1, TLR4, CDK6, ABCA1, EPHA2, PTGS2, COL1A2, CCNE1, PDE4A and HAS2	**Signaling pathway of:** MAPK. **Activation pathway of:** MAPK. **Involved in:** miRNA-mediated post-transcriptional gene silencing, miRNA-mediated gene silencing by inhibition of translation, miRNA-mediated gene silencing by mRNA destabilization, positive regulation of cell cycle, negative regulation of SMAD protein signal transduction, positive regulation of protein serine/threonine kinase activity, positive regulation of tau-protein kinase activity, positive regulation of apoptotic signaling pathway.	**Psoriasis DITRA*** (MAPK)	[32]
39.	miR-34a-5p 407040 miRNA	8	BMP7, NANOG, ATG4D, MAP2K1, IMPA1, HMGB1, GRM7, IMPDH2, PCBP2, SPI1, KCNH1, CXCL11, AR, CCL22, SIRT1, ATG4A, KIT, AGTR1, MAP3K9, ANK3, CACNB3, CD44, CXCL16, TREM2, ACSL1, CEBPB, FOSL1, ERBB2, ULBP2, ARHGDIB, ATG4B, PIK3CG, YY1, RAD51, CDC25A, KCNH2, RICTOR, ZAP70, GDF5, AIP, ATG7, E2F1, ACSL4, SYT1, MAGEA3 and CXCL10	**Signaling pathway of:** Interleukins (IL4, IL13), MAPK family (MAPK1, MAPK3, STAT and JAK-STAT. **Activation pathway of:** MAPK1 and MAPK3. **Involved in:** positive regulation of gene expression, miRNA-mediated post-transcriptional gene silencing, miRNA-mediated gene silencing by inhibition of translation, miRNA-mediated gene silencing by mRNA destabilization, positive regulation of cell cycle, negative regulation of peroxisome proliferator activated receptor signaling pathway, response to axon injury, negative regulation of B cell receptor signaling pathway, negative regulation of phosphatidylinositol 3-kinase/protein kinase B signal transduction and positive regulation of protein acetylation,	**R. Arthritis DITRA*** (MAPK, JAK-STAT)	[33,34]
40.	miR-449a 554213	8	E2F3, CAPN6, SIRT1, XIST, LEF1, POU2F1, IL6R, HNF4A, MFAP4, MET, RRAS, FOS,	**Signaling pathway of:** Interleukins (IL4, IL13), MAPK family (MAPK1, MAPK3) STAT and JAK-STAT.	**Psoriasis DITRA***	[35]

	miRNA		CDK6, CDK4, ADAM10, CCNE2, CCND1, HDAC8, MAZ, CREB5, MYC, FLOT2, ITPR1, CRIP2, PKP4, GMNN, HDAC1, CCN5, NOTCH1, BCL2, CDC25A and TSEN15	**Activation pathway of:** MAPK1 and MAPK3 **Involved in:** negative regulation of cell population proliferation, negative regulation of gene expression, negative regulation of cell migration, miRNA-mediated post-transcriptional gene silencing and positive regulation of apoptotic signaling pathway.	(MAPK, JAK-STAT)	
41.	miR-130a-3p 406919 miRNA	7	RAB5A, NEUROD1, NPTX1, HOXA10, PDGFRA, MAFB, ESR1, TAC1, ATXN1, PPARGC1A, MYC, MECP2, RUNX3, TNF, CSF1, NRP1, SMAD4, APP, ATG2B, DICER1, PTEN, TGFB1, GJA1, NBEA, SLAIN1, MEOX2, IL18, HOXA5, PPARG, TGFB2, KLF4, XIAP and MAP3K12	**Signaling pathway of:** Interleukins (IL4, IL10, IL13), MAPK, STAT and JAK-STAT. **Activation pathway of:** MAPK **Involved in:** positive regulation of natural killer cell activation, miRNA-mediated post-transcriptional gene silencing, miRNA-mediated gene silencing by mRNA destabilization, negative regulation of macrophage activation and cellular response to transforming growth factor beta stimulus.	**Psoriasis DITRA*** (MAPK, JAK-STAT)	[21]
42.	miR-186-5p 406962 miRNA	7	SETD2, MAP3K2, PTTG1, FGF2, MAPT, GJA1, ABCB1, CSNK2A1, NCSTN, TWIST1, HIF1A, FOXO1, XIAP, PPM1B, RELA, VEGFA, P2RX7, PAK5, CASP10, AKAP12 and PVT1	**Signaling pathway of:** Interleukins (IL4, IL13) and MAPK. **Activation pathway of:** MAPK. **Involved in:** miRNA-mediated post-transcriptional gene silencing.	**Psoriasis R. Arthritis DITRA*** (MAPK, JAK-STAT)	[36,37]
43.	miR-301a-3p 407027 miRNA	7	SNIP1, MAP3K5, CDC14A, MEOX2, NDRG2, RUNX3, UVRAG, PTEN, NKRF, TIMP2, BTG1, SERPINE1, BCL2L11, CDKN1A and SMAD4	**Involved in:** miRNA-mediated post-transcriptional gene silencing.	**Psoriasis**	[38]
44.	miR-301b-3p 100126318 miRNA	7	ABCA1, PTEN, TP63, NDRG2, LDLR, BCL2L11 and NR3C2	**Involved in:** miRNA-mediated post-transcriptional gene silencing, lipoprotein transport and negative regulation of cholesterol efflux.	**N/A**	
45.	miR-495-3p 574453 miRNA	7	ATP7A, SMR3B, ABCB1, RUNX3, SOX9, AKT1, PBX3, VEZF1, MTA3, FOXC1, PTP4A3, MEIS1, MAT1A, BMI1, HSPA5, CCL2, TBC1D9 and HMGA2	**Involved in:** negative regulation of transporter activity, miRNA-mediated post-transcriptional gene silencing, miRNA-mediated gene silencing by inhibition of translation, miRNA-mediated gene silencing by mRNA	**R. Arthritis**	[39]

				destabilization, negative regulation of endothelial cell differentiation, negative regulation of cell migration involved in sprouting angiogenesis,<br>positive regulation of G1/S transition of mitotic cell cycle, negative regulation of artery morphogenesis, negative regulation of endothelial cell apoptotic process.		

* Proposed targets that may relate with DITRA based on the Signaling and Activation Pathway

1. Zhang, J.; Zhu, J.; Chen, X.; Xia, H.; Yang, L. E3 ubiquitin ligase Trim33 ubiquitylates Annexin A2 to promote NF-kappaB induced skin inflammation in psoriasis. *Journal of dermatological science* **2022**, *107*, 160-168, doi:10.1016/j.jdermsci.2022.09.002.
2. Baran, A.; Swiderska, M.; Bacharewicz-Szczerbicka, J.; Mysliwiec, H.; Flisiak, I. Serum Fatty Acid-Binding Protein 4 is Increased in Patients with Psoriasis. *Lipids* **2017**, *52*, 51-60, doi:10.1007/s11745-016-4211-4.
3. Gupta, R.; Debbaneh, M.G.; Liao, W. Genetic Epidemiology of Psoriasis. *Current dermatology reports* **2014**, *3*, 61-78, doi:10.1007/s13671-013-0066-6.
4. Zima, K.; Purzycka-Bohdan, D.; Szczerkowska-Dobosz, A.; Gabig-Ciminska, M. Keratinocyte-Mediated Antigen Presentation in Psoriasis: Preliminary Insights from In Vitro Studies. *International journal of molecular sciences* **2024**, *25*, doi:10.3390/ijms252413387.
5. Dopytalska, K.; Ciechanowicz, P.; Wiszniewski, K.; Szymanska, E.; Walecka, I. The Role of Epigenetic Factors in Psoriasis. *International journal of molecular sciences* **2021**, *22*, doi:10.3390/ijms22179294.
6. Shi, R.; Ma, R.; Jiang, X.; Tang, X.; Gong, Y.; Yu, Z.; Shi, Y. Implications of LncRNAs and CircRNAs in psoriasis: a review. *RNA biology* **2023**, *20*, 334-347, doi:10.1080/15476286.2023.2223486.
7. Ghahramani Almanghadim, H.; Karimi, B.; Valizadeh, S.; Ghaedi, K. Biological functions and affected signaling pathways by Long Non-Coding RNAs in the immune system. *Non-coding RNA research* **2025**, *10*, 70-90, doi:10.1016/j.ncrna.2024.09.001.
8. Mostafa, A.; Sabry, D.; Aboraia, N.; Fawzy, A.; Abou-Elalla, A.A. Dyslipidemia initiates keratinocytes proliferation through upregulation of lncRNA NEAT in psoriasis patients. *Molecular biology reports* **2023**, *50*, 7597-7604, doi:10.1007/s11033-023-08527-w.
9. Chen, J.; Ao, L.; Yang, J. Long non-coding RNAs in diseases related to inflammation and immunity. *Annals of translational medicine* **2019**, *7*, 494, doi:10.21037/atm.2019.08.37.
10. Shen, H.; Wang, D.; Zhan, M.; Ding, H.; Zhao, H. MicroRNA-146a and microRNA-146b deficiency correlates with exacerbated disease activity, and their longitude increment relates to etanercept response in psoriasis patients. *Journal of clinical laboratory analysis* **2022**, *36*, e24198, doi:10.1002/jcla.24198.
11. Alhelf, M.; Rashed, L.; Doss, R.W.; Mohamed, S.M.; Abd Elazeem, N.A. Long noncoding RNA (taurine upregulated gene 1) and micro RNA-377: emerging players in the development of metabolic syndrome among psoriasis patients. *Beni-Suef University Journal of Basic and Applied Sciences* **2023**, *12*, 68, doi:10.1186/s43088-023-00404-6.
12. Zhang, M.; Lu, N.; Guo, X.Y.; Li, H.J.; Guo, Y.; Lu, L. Influences of the lncRNA TUG1-miRNA-34a-5p network on fibroblast-like synoviocytes (FLSs) dysfunction in rheumatoid arthritis through targeting the lactate dehydrogenase A (LDHA). *Journal of clinical laboratory analysis* **2021**, *35*, e23969, doi:10.1002/jcla.23969.

13. Zhang, W.; Yi, X.; An, Y.; Guo, S.; Li, S.; Song, P.; Chang, Y.; Zhang, S.; Gao, T.; Wang, G.; et al. MicroRNA-17-92 cluster promotes the proliferation and the chemokine production of keratinocytes: implication for the pathogenesis of psoriasis. *Cell death & disease* **2018**, *9*, 567, doi:10.1038/s41419-018-0621-y.
14. Li, S.; Zhu, X.; Zhang, N.; Cao, R.; Zhao, L.; Li, X.; Zhang, J.; Yu, J. LncRNA NORAD engages in psoriasis by binding to miR-26a to regulate keratinocyte proliferation. *Autoimmunity* **2021**, *54*, 129-137, doi:10.1080/08916934.2021.1897976.
15. Anandaram, H.; Anand, D.A. Computational Analysis of Micro RNA based Target Interactions Related to Genome Wide Association Studies of Psoriasis. *J Pure Appl Microbiol.* **2014**, *8*, 823-826.
16. Laha, S.; Das, S.; Banerjee, U.; Ganguly, T.; Senapati, S.; Chatterjee, G.; Chatterjee, R. Genome-wide RNA-seq, DNA methylation and small RNA-seq analysis unraveled complex gene regulatory networks in psoriasis pathogenesis. *Gene* **2025**, *933*, 148903, doi:10.1016/j.gene.2024.148903.
17. Li, J.; Pang, D.; Zhou, L.; Ouyang, H.; Tian, Y.; Yu, H. miR-26a-5p inhibits the proliferation of psoriasis-like keratinocytes in vitro and in vivo by dual interference with the CDC6/CCNE1 axis. *Aging* **2024**, *16*, 4631-4653, doi:10.18632/aging.205618.
18. Chen, L.; Wang, X.; Liu, C.; Chen, X.; Li, P.; Qiu, W.; Guo, K. Integrative analysis of gene and microRNA expression profiles reveals candidate biomarkers and regulatory networks in psoriasis. *Medicine* **2024**, *103*, e39002, doi:10.1097/MD.0000000000039002.
19. Carreras-Badosa, G.; Maslovskaja, J.; Vaher, H.; Pajusaar, L.; Annilo, T.; Lattekivi, F.; Hubenthal, M.; Rodriguez, E.; Weidinger, S.; Kingo, K.; et al. miRNA expression profiles of the perilesional skin of atopic dermatitis and psoriasis patients are highly similar. *Scientific reports* **2022**, *12*, 22645, doi:10.1038/s41598-022-27235-2.
20. Peng, X.; Wang, Q.; Li, W.; Ge, G.; Peng, J.; Xu, Y.; Yang, H.; Bai, J.; Geng, D. Comprehensive overview of microRNA function in rheumatoid arthritis. *Bone research* **2023**, *11*, 8, doi:10.1038/s41413-023-00244-1.
21. Alatas, E.T.; Kara, M.; Dogan, G.; Akin Belli, A. Blood microRNA expressions in patients with mild to moderate psoriasis and the relationship between microRNAs and psoriasis activity. *Anais brasileiros de dermatologia* **2020**, *95*, 702-707, doi:10.1016/j.abd.2020.07.001.
22. Ouboussad, L.; Hunt, L.; Hensor, E.M.A.; Nam, J.L.; Barnes, N.A.; Emery, P.; McDermott, M.F.; Buch, M.H. Profiling microRNAs in individuals at risk of progression to rheumatoid arthritis. *Arthritis research & therapy* **2017**, *19*, 288, doi:10.1186/s13075-017-1492-9.
23. Jiang, X.; Shi, R.; Ma, R.; Tang, X.; Gong, Y.; Yu, Z.; Shi, Y. The role of microRNA in psoriasis: A review. *Experimental dermatology* **2023**, *32*, 1598-1612, doi:10.1111/exd.14871.
24. Tokic, S.; Jirous, M.; Pluzaric, V.; Mihalj, M.; Sola, M.; Tolusic Levak, M.; Glavas, K.; Balogh, P.; Stefanic, M. The miR-20a/miR-92b Profile Is Associated with Circulating gammadelta T-Cell Perturbations in Mild Psoriasis. *International journal of molecular sciences* **2023**, *24*, doi:10.3390/ijms24054323.
25. Yang, S.C.; Alalaiwe, A.; Lin, Z.C.; Lin, Y.C.; Aljuffali, I.A.; Fang, J.Y. Anti-Inflammatory microRNAs for Treating Inflammatory Skin Diseases. *Biomolecules* **2022**, *12*, doi:10.3390/biom12081072.
26. Liu, Q.; Wu, D.H.; Han, L.; Deng, J.W.; Zhou, L.; He, R.; Lu, C.J.; Mi, Q.S. Roles of microRNAs in psoriasis: Immunological functions and potential biomarkers. *Experimental dermatology* **2017**, *26*, 359-367, doi:10.1111/exd.13249.
27. Ueta, M.; Nishigaki, H.; Komai, S.; Mizushima, K.; Tamagawa-Mineoka, R.; Naito, Y.; Katoh, N.; Sotozono, C.; Kinoshita, S. Positive regulation of innate immune response by miRNA-let-7a-5p. *Frontiers in genetics* **2022**, *13*, 1025539, doi:10.3389/fgene.2022.1025539.

28. Wu, Y.; Liu, L.; Bian, C.; Diao, Q.; Nisar, M.F.; Jiang, X.; Bartsch, J.W.; Zhong, M.; Hu, X.; Zhong, J.L. MicroRNA let-7b inhibits keratinocyte differentiation by targeting IL-6 mediated ERK signaling in psoriasis. *Cell communication and signaling : CCS* **2018**, *16*, 58, doi:10.1186/s12964-018-0271-9.
29. Quah, S.; Sundaram, G.M.; Subramanian, G.; Vaz, C.; Tan, J.S.L.; Kabir, R.F.; Ong, J.M.R.; Oon, H.H.; Theng, C.; Sampath, P. IL-17-Mediated Downregulation of miR-101 Facilitates the Expression of EZH2 to Promote Epidermal Hyperplasia in Psoriasis. *The Journal of investigative dermatology* **2024**, *144*, 403-407 e407, doi:10.1016/j.jid.2023.07.013.
30. Liu, S.; Gong, J. miR-124-3p Delivered Using Exosomes Attenuates the Keratinocyte Response to IL-17A Stimulation in Psoriasis. *Oxidative medicine and cellular longevity* **2022**, *2022*, 6264474, doi:10.1155/2022/6264474.
31. Castro, I.; Carvajal, P.; Jara, D.; Aguilera, S.; Heathcote, B.; Barrera, M.J.; Aliaga-Tobar, V.; Maracaja-Coutinho, V.; Urzua, U.; Quest, A.F.G.; et al. Small RNA Expression Profiling Reveals hsa-miR-181d-5p Downregulation Associated With TNF-alpha Overexpression in Sjogren's Syndrome Patients. *Frontiers in immunology* **2022**, *13*, 870094, doi:10.3389/fimmu.2022.870094.
32. Cheung, L.; Fisher, R.M.; Kuzmina, N.; Li, D.; Li, X.; Werngren, O.; Blomqvist, L.; Stahle, M.; Landen, N.X. Psoriasis Skin Inflammation-Induced microRNA-26b Targets NCEH1 in Underlying Subcutaneous Adipose Tissue. *The Journal of investigative dermatology* **2016**, *136*, 640-648, doi:10.1016/j.jid.2015.12.008.
33. Ren, C.; Li, M.; Zheng, Y.; Wu, F.; Du, W.; Quan, R. Identification of diagnostic genes and vital microRNAs involved in rheumatoid arthritis: based on data mining and experimental verification. *PeerJ* **2021**, *9*, e11427, doi:10.7717/peerj.11427.
34. Nystrom, S.; Hultberg, J.; Blixt, E.; Nilsdotter-Augustinsson, A.; Larsson, M. Plasma Levels of mir-34a-5p Correlate with Systemic Inflammation and Low Naive CD4 T Cells in Common Variable Immunodeficiency. *Journal of clinical immunology* **2023**, *44*, 21, doi:10.1007/s10875-023-01618-0.
35. Yue, Q.; Li, Z.; Zhang, Q.; Jin, Q.; Zhang, X.; Jin, G. Identification of Novel Hub Genes Associated with Psoriasis Using Integrated Bioinformatics Analysis. *International journal of molecular sciences* **2022**, *23*, doi:10.3390/ijms232315286.
36. Cheng, T.; Shang, Q.; Mak, W.; Li, M.; Kwok, K.; Yim, I.; Li, E.; Wong, P.; Lao, V.; Pang, H.; et al. SAT0073 MIR-186-5P targeting IL-33 gene as biomarker to predict subclinical atherosclerosis in patients with early rheumatoid arthritis. *Annals of the Rheumatic Diseases* **2017**, *76*, 796-796, doi:10.1136/annrheumdis-2017-eular.2840.
37. Pelosi, A.; Lunardi, C.; Fiore, P.F.; Tinazzi, E.; Patuzzo, G.; Argentino, G.; Moretta, F.; Puccetti, A.; Dolcino, M. MicroRNA Expression Profiling in Psoriatic Arthritis. *BioMed research international* **2018**, *2018*, 7305380, doi:10.1155/2018/7305380.
38. Gerasymchuk, M.; Cherkasova, V.; Kovalchuk, O.; Kovalchuk, I. The Role of microRNAs in Organismal and Skin Aging. *International journal of molecular sciences* **2020**, *21*, doi:10.3390/ijms21155281.
39. Sui, X.; Liu, H.; Zhou, Y. Expression of miR-495 and miR-326 in peripheral blood of rheumatoid arthritis patients and its significance. *Experimental and therapeutic medicine* **2020**, *20*, 3766-3774, doi:10.3892/etm.2020.9124.
