# Supplementary material for: Decoding Non-Coding RNA Regulators in DITRA: From Genomic Insights to Potential Biomarkers and Therapeutic Targets
Source: Genes (Basel). 2025 Jun 27;16(7):753. doi: 10.3390/genes16070753 (PMC12295128; doi:10.3390/genes16070753)
Supplement: Supplementary file 1 [file genes-16-00753-s001.zip › Supplementary-TableS5.pdf]

A/A	NcRNA / NBCI ID / Type	Category	Related Disease	References
1.	CSDE1<br>7812<br>Gene	Pharmacological Target / Biomarker	Cancer and Anticancer Drug	[1]
2.	FNBP4<br>23360<br>Gene	Pharmacological Target / Biomarker	Cancer	[2]
3.	HNF4A<br>3172<br>Gene	Pharmacological Target / Biomarker	Diabetes and Cancer	[3,4]
4.	MECP2<br>4204<br>Gene	Pharmacological Target / Biomark	Neurological disorders and Rett Syndrome	[5,6]
5.	PLEKHA1<br>59338<br>Gene	Biomark	Systemic Lupus Erythematosus and Ulcerative Colitis	[7]
6.	TINCR<br>257000<br>lncRNA	Pharmacological Target / Biomarker	Carcinoma and Cancer, Psoriasis	[8-11]
7.	MALAT1<br>378938<br>lncRNA	Pharmacological Target / Biomarker	Metastasis and Psoriasis	[12-15]
8.	NEAT1<br>283131<br>lncRNA	Pharmacological Target / Biomarker	Cancer	[16,17]
9.	SNHG16<br>100507246<br>lncRNA	Pharmacological Target / Biomarker	Cancer and Psoriasis	[18,19]
10.	TUG1<br>55000<br>lncRNA	Pharmacological Target / Biomarker	Cancer and Chronic heart failure	[20,21]
11.	MIR17HG<br>407975<br>lncRNA	Pharmacological Target / Biomarker	Cancer	[21,22]
12.	NORAD<br>647979<br>lncRNA	Pharmacological Target / Biomarker	Cancer and Psoriasis	[23-26]
13.	miR-19a-3p<br>406979<br>miRNA	Pharmacological Target / Biomarker	Psoriasis and Atherosclerosis	[27,28]
14.	let-7c-5p<br>406885<br>miRNA	Pharmacological Target / Biomarker	Adenocarcinoma, Cancer and Psoriasis	[29-31]
15.	let-7g-5p	Pharmacological		[32]

	406890<br>miRNA	Target	Oncogenic and Cancer	
16.	miR-106a-5p<br>406899<br>miRNA	Pharmacological Target / Biomarker	Neuroinflammation and Cancer	[33,34]
17.	miR-122-5p<br>406906<br>miRNA	Biomarker	Liver Diseases and Carcinoma	[35,36]
18.	miR-17-5p<br>406952<br>miRNA	Pharmacological Target / Biomarker	Cancer and Psoriasis	[37-40]
19.	miR-30e-5p<br>407034<br>miRNA	Pharmacological Target / Biomarker	Myasthenia Gravis and Cacner	[41-44]
20.	miR-373-3p<br>442918<br>miRNA	Pharmacological Target / Biomarker	Tumorigenesis and Cancer	[45-47]
21.	miR-454-3p<br>768216<br>miRNA	Pharmacological Target / Biomarker	Cancer	[48,49]
22.	let-7i-5p<br>406891<br>miRNA	Pharmacological Target / Biomarker	Cancer, Urethral Fibroblasts and Psoriasis	[50-52]
23.	miR-107<br>406901<br>miRNA	Pharmacological Target / Biomarker	Carcinoma, Cancer and Hypertension	[53-56]
24.	miR-130b-3p<br>406920<br>miRNA	Pharmacological Target / Biomarker	Nephroblastoma, Cancer and	[57-59]
25.	miR-20a-5p<br>406982<br>miRNA	Pharmacological Target / Biomarker	Cancer	[60-62]
26.	miR-590-3p<br>693175<br>miRNA	Pharmacological Target	Cancer	[63,64]
27.	miR-93-5p<br>407050<br>miRNA	Pharmacological Target / Biomarker	Prolactinoma and Myeloid Leukemia	[65-67]
28.	let-7a-5p<br>406881<br>miRNA	Pharmacological Target / Biomarker	Immunotherapy, Osteoporosis and Cancer	[68-70]
29.	let-7b-5p<br>406884<br>miRNA	Biomarker	Antibiotic Sensitivity and Heart disease	[71,72]

30.	let-7d-5p 406886 miRNA	Pharmacological Target / Biomarker	Alzheimer, Cancer, Psoriatic Arthritis, Rheumatoid Arthritis, and Ankylosing Spondyliti	[72-74]
31.	let-7e-5p 406887 miRNA	Pharmacological Target / Biomarker	Multiple Sclerosis, Skin Fibrosis and Ischemic Stroke	[75-78]
32.	let-7f-5p 406889 miRNA	Pharmacological Target / Biomarker	Multiple Sclerosis, Skin Fibrosis and Psoriasis	[52,75,76]
33.	miR-101-3p 406893 miRNA	Pharmacological Target / Biomarker	Carcinoma, Cancer and Depression	[79,80]
34.	miR-106b-5p 406900 miRNA	Pharmacological Target / Biomarker	Cancer	[81,82]
35.	miR-124-3p 406909 miRNA	Pharmacological Target / Biomarker	Carcinoma and Cancer	[83,84]
36.	miR-181d-5p 574457 miRNA	Pharmacological Target / Biomarker	Hypercholesterolemia and Cancer	[85,86]
37.	miR-26b-5p 407017 miRNA	Pharmacological Target / Biomarker	Carcinoma, Cancer and Psoriasis	[87-89]
38.	miR-34a-5p 407040 miRNA	Pharmacological Target	Cancer	[90]
39.	miR-449a 554213 miRNA	Pharmacological Target / Biomarker	Cancer	[91,92]
40.	miR-130a-3p 406919 miRNA	Pharmacological Target / Biomarker	Crohn, Adenocarcinoma, Cancer and Psoriasis	[93-96]
41.	miR-186-5p 406962 miRNA	Pharmacological Target / Biomarker	Stress, Cancer, Skin and Psoriasis	[97-100]
42.	miR-301a-3p 407027 miRNA	Pharmacological Target / Biomarker	Carcinoma and Cancer	[101-103]
43.	miR-301b-3p 100126318 miRNA	Pharmacological Target / Biomarker	Cancer	[104-106]
44.	miR-495-3p	Pharmacological		[107,108]

	574453 miRNA	Target / Biomarker	Tumorigenesis, Cancer and Diabetes	

1. Guo, A.X.; Cui, J.J.; Wang, L.Y.; Yin, J.Y. The role of CSDE1 in translational reprogramming and human diseases. *Cell communication and signaling : CCS* **2020**, *18*, 14, doi:10.1186/s12964-019-0496-2.
2. Baran, A.; Swiderska, M.; Bacharewicz-Szczerbicka, J.; Mysliwiec, H.; Flisiak, I. Serum Fatty Acid-Binding Protein 4 is Increased in Patients with Psoriasis. *Lipids* **2017**, *52*, 51-60, doi:10.1007/s11745-016-4211-4.
3. Girard, R.; Tremblay, S.; Noll, C.; St-Jean, S.; Jones, C.; Gelinas, Y.; Maloum-Rami, F.; Perreault, N.; Laplante, M.; Carpentier, A.C.; et al. The transcription factor hepatocyte nuclear factor 4A acts in the intestine to promote white adipose tissue energy storage. *Nature communications* **2022**, *13*, 224, doi:10.1038/s41467-021-27934-w.
4. Zheng, K.W.; Zhang, C.H.; Wu, W.; Zhu, Z.; Gong, J.P.; Li, C.M. FNBP4 is a Potential Biomarker Associated with Cuproptosis and Promotes Tumor Progression in Hepatocellular Carcinoma. *International journal of general medicine* **2023**, *16*, 467-480, doi:10.2147/IJGM.S395881.
5. Przanowski, P.; Wasko, U.; Zheng, Z.; Yu, J.; Sherman, R.; Zhu, L.J.; McConnell, M.J.; Tushir-Singh, J.; Green, M.R.; Bhatnagar, S. Pharmacological reactivation of inactive X-linked Mecp2 in cerebral cortical neurons of living mice. *Proceedings of the National Academy of Sciences of the United States of America* **2018**, *115*, 7991-7996, doi:10.1073/pnas.1803792115.
6. Good, K.V.; Vincent, J.B.; Ausio, J. MeCP2: The Genetic Driver of Rett Syndrome Epigenetics. *Frontiers in genetics* **2021**, *12*, 620859, doi:10.3389/fgene.2021.620859.
7. Zhou, J.; Guo, Y.; Tian, Z.; Lv, Z.; Jiang, S.; Zhang, W. Aberrant formation of the neutrophil extracellular trap and the expression of the PLEKHA1 in systemic lupus erythematosus and ulcerative colitis. *Molecular and cellular biochemistry* **2025**, doi:10.1007/s11010-025-05300-4.
8. Sharma, U.; Barwal, T.S.; Malhotra, A.; Pant, N.; Vivek; Dey, D.; Gautam, A.; Tuli, H.S.; Vasquez, K.M.; Jain, A. Long non-coding RNA TINCR as potential biomarker and therapeutic target for cancer. *Life sciences* **2020**, *257*, 118035, doi:10.1016/j.lfs.2020.118035.
9. Freisenhausen, J.C.; Luo, L.; Kelemen, E.; Elton, J.; Skoog, V.; Pivarcsi, A.; Sonkoly, E. RNA Sequencing Reveals the Long Non-Coding RNA Signature in Psoriasis Keratinocytes and Identifies CYDAER as a Long Non-Coding RNA Regulating Epidermal Differentiation. *Experimental dermatology* **2025**, *34*, e70054, doi:10.1111/exd.70054.
10. Shi, J.; Guo, C.; Li, Y.; Ma, J. The long noncoding RNA TINCR promotes self-renewal of human liver cancer stem cells through autophagy activation. *Cell death & disease* **2022**, *13*, 961, doi:10.1038/s41419-022-05424-1.
11. Wang, L.; Wang, Y.; Xu, L. Overexpression of lncRNA TINCR inhibits cutaneous squamous cell carcinoma cells through promotes methylation of Myc and TERC genes. *Archives of dermatological research..* **2025**, *317*, 559, doi:10.1007/s00403-025-03964-y.
12. Uthman, Y.A.; Ibrahim, K.G.; Abubakar, B.; Bello, M.B.; Malami, I.; Imam, M.U.; Qusty, N.; Cruz-Martins, N.; Batiha, G.E.; Abubakar, M.B. MALAT1: A Promising Therapeutic Target for the Treatment of Metastatic Colorectal Cancer. *Biochemical pharmacology* **2021**, *190*, 114657, doi:10.1016/j.bcp.2021.114657.
13. Amodio, N.; Raimondi, L.; Juli, G.; Stamato, M.A.; Caracciolo, D.; Tagliaferri, P.; Tassone, P. MALAT1: a druggable long non-coding RNA for targeted anti-cancer approaches. *Journal of hematology & oncology* **2018**, *11*, 63, doi:10.1186/s13045-018-0606-4.
14. Guo, J.; Zhang, H.; Lin, W.; Lu, L.; Su, J.; Chen, X. Signaling pathways and targeted therapies for psoriasis. *Signal transduction and targeted therapy* **2023**, *8*, 437, doi:10.1038/s41392-023-01655-6.

15. Elamir, A.M.; Shaker, O.G.; El-Komy, M.H.; Mahmoud Sharabi, M.; Aboraia, N.M. The role of LncRNA MALAT-1 and MiRNA-9 in Psoriasis. *Biochemistry and biophysics reports* **2021**, *26*, 101030, doi:10.1016/j.bbrep.2021.101030.
16. Dong, P.; Xiong, Y.; Yue, J.; Hanley, S.J.B.; Kobayashi, N.; Todo, Y.; Watari, H. Long Non-coding RNA NEAT1: A Novel Target for Diagnosis and Therapy in Human Tumors. *Frontiers in genetics* **2018**, *9*, 471, doi:10.3389/fgene.2018.00471.
17. Nitusca, D.; Marcu, A.; Dema, A.; Balacescu, L.; Balacescu, O.; Bardan, R.; Cumpanas, A.A.; Sirbu, I.O.; Petrut, B.; Seclaman, E.; et al. Long Noncoding RNA NEAT1 as a Potential Candidate Biomarker for Prostate Cancer. *Life* **2021**, *11*, doi:10.3390/life11040320.
18. Xiao, Y.; Xiao, T.; Ou, W.; Wu, Z.; Wu, J.; Tang, J.; Tian, B.; Zhou, Y.; Su, M.; Wang, W. LncRNA SNHG16 as a potential biomarker and therapeutic target in human cancers. *Biomarker research* **2020**, *8*, 41, doi:10.1186/s40364-020-00221-4.
19. Zhao, Y.; Wang, L.; Zhang, X.; Zhang, L.; Wei, F.; Li, S.; Li, Y. Identification of neutrophil extracellular traps genes as potential biomarkers in psoriasis based on bioinformatics analysis. *Scientific reports* **2024**, *14*, 23848, doi:10.1038/s41598-024-75069-x.
20. Ou, C.; Li, G. Long non-coding RNA TUG1: a novel therapeutic target in small cell lung cancer. *Journal of thoracic disease* **2017**, *9*, E644-E645, doi:10.21037/jtd.2017.06.94.
21. Zhu, Q.; Li, S.; Ji, K.; Zhou, H.; Luo, C.; Sui, Y. Differentially expressed TUG1 and miR-145-5p indicate different severity of chronic heart failure and predict 2-year survival prognosis. *Experimental and therapeutic medicine* **2021**, *22*, 1362, doi:10.3892/etm.2021.10796.
22. Yuan, J.; Tan, L.; Yin, Z.; Zhu, W.; Tao, K.; Wang, G.; Shi, W.; Gao, J. MIR17HG-miR-18a/19a axis, regulated by interferon regulatory factor-1, promotes gastric cancer metastasis via Wnt/beta-catenin signalling. *Cell death & disease* **2019**, *10*, 454, doi:10.1038/s41419-019-1685-z.
23. Capela, A.M.; Tavares-Marcos, C.; Estima-Arede, H.F.; Nobrega-Pereira, S.; Bernardes de Jesus, B. NORAD-Regulated Signaling Pathways in Breast Cancer Progression. *Cancers* **2024**, *16*, doi:10.3390/cancers16030636.
24. Alves-Vale, C.; Capela, A.M.; Tavares-Marcos, C.; Domingues-Silva, B.; Pereira, B.; Santos, F.; Gomes, C.P.; Espadas, G.; Vitorino, R.; Sabido, E.; et al. Expression of NORAD correlates with breast cancer aggressiveness and protects breast cancer cells from chemotherapy. *Molecular therapy. Nucleic acids* **2023**, *33*, 910-924, doi:10.1016/j.omtn.2023.08.019.
25. Kielbowski, K.; Jedrasiak, A.; Bakinowska, E.; Pawlik, A. The Role of Long Non-Coding RNA in the Pathogenesis of Psoriasis. *Non-coding RNA* **2025**, *11*, doi:10.3390/ncrna11010007.
26. Cui, J.; Zhang, X.; Deng, J.; Yan, Y.; Yao, D.; Deng, H.; Yu, J.; Ye, S.; Han, L.; Yu, X.; et al. Potential biomarkers for psoriasis topical treatment by in-depth serum proteomics. *The Journal of dermatological treatment* **2023**, *34*, 2248318, doi:10.1080/09546634.2023.2248318.
27. Jiang, X.; Shi, R.; Ma, R.; Tang, X.; Gong, Y.; Yu, Z.; Shi, Y. The role of microRNA in psoriasis: A review. *Experimental dermatology* **2023**, *32*, 1598-1612, doi:10.1111/exd.14871.
28. Luo, J.; Wang, L.; Cui, C.; Chen, H.; Zeng, W.; Li, X. MicroRNA-19a-3p inhibits endothelial dysfunction in atherosclerosis by targeting JCAD. *BMC cardiovascular disorders* **2024**, *24*, 394, doi:10.1186/s12872-024-04063-y.
29. Li, J.; Pang, D.; Zhou, L.; Ouyang, H.; Tian, Y.; Yu, H. miR-26a-5p inhibits the proliferation of psoriasis-like keratinocytes in vitro and in vivo by dual interference with the CDC6/CCNE1 axis. *Aging* **2024**, *16*, 4631-4653, doi:10.18632/aging.205618.
30. Kar, B.R.; Sathishkumar, D.; Tahiliani, S.; Parthasarathi, A.; Neema, S.; Ganguly, S.; Venkatachalam, K.; Parasramani, S.G.; Komeravelli, H.; Thomas, J. Biomarkers in Psoriasis: The Future of Personalised Treatment. *Indian journal of dermatology* **2024**, *69*, 256-263, doi:10.4103/ijd.ijd_167_24.

31. Wang, L.; Xiao, X.; Du, H. The Regulation of let-7c-5p on the Biological Characteristics of Lung Adenocarcinoma Cells by Targeting AURKB. *Molecular biotechnology* **2022**, *64*, 526-534, doi:10.1007/s12033-021-00446-0.
32. Hung, K.C.; Tien, N.; Bau, D.T.; Yao, C.H.; Chen, C.H.; Yang, J.L.; Lin, M.L.; Chen, S.S. Let-7g Upregulation Attenuated the KRAS-PI3K-Rac1-Akt Axis-Mediated Bioenergetic Functions. *Cells* **2023**, *12*, doi:10.3390/cells12182313.
33. Du, W.; Fan, L.; Du, J. Neuroinflammation-associated miR-106a-5p serves as a biomarker for the diagnosis and prognosis of acute cerebral infarction. *BMC neurology* **2023**, *23*, 248, doi:10.1186/s12883-023-03241-3.
34. Pan, Y.J.; Wei, L.L.; Wu, X.J.; Huo, F.C.; Mou, J.; Pei, D.S. MiR-106a-5p inhibits the cell migration and invasion of renal cell carcinoma through targeting PAK5. *Cell death & disease* **2017**, *8*, e3155, doi:10.1038/cddis.2017.561.
35. Colaianni, F.; Zelli, V.; Compagnoni, C.; Miscione, M.S.; Rossi, M.; Vecchiotti, D.; Di Padova, M.; Alesse, E.; Zazzeroni, F.; Tessitore, A. Role of Circulating microRNAs in Liver Disease and HCC: Focus on miR-122. *Genes* **2024**, *15*, doi:10.3390/genes15101313.
36. Wei, X.Y.; Ding, J.; Tian, W.G.; Yu, Y.C. MicroRNA-122 as a diagnostic biomarker for hepatocellular carcinoma related to hepatitis C virus: a meta-analysis and systematic review. *The Journal of international medical research* **2020**, *48*, 300060520941634, doi:10.1177/0300060520941634.
37. Stoen, M.J.; Andersen, S.; Rakaee, M.; Pedersen, M.I.; Ingebriktsen, L.M.; Bremnes, R.M.; Donnem, T.; Lombardi, A.P.G.; Kilvaer, T.K.; Busund, L.T.; et al. High expression of miR-17-5p in tumor epithelium is a predictor for poor prognosis for prostate cancer patients. *Scientific reports* **2021**, *11*, 13864, doi:10.1038/s41598-021-93208-6.
38. Chen, L.; Wang, X.; Liu, C.; Chen, X.; Li, P.; Qiu, W.; Guo, K. Integrative analysis of gene and microRNA expression profiles reveals candidate biomarkers and regulatory networks in psoriasis. *Medicine* **2024**, *103*, e39002, doi:10.1097/MD.0000000000039002.
39. Bobbili, M.R.; Mader, R.M.; Grillari, J.; Dellago, H. OncomiR-17-5p: alarm signal in cancer? *Oncotarget* **2017**, *8*, 71206-71222, doi:10.18632/oncotarget.19331.
40. Kim, T.W.; Lee, Y.S.; Yun, N.H.; Shin, C.H.; Hong, H.K.; Kim, H.H.; Cho, Y.B. MicroRNA-17-5p regulates EMT by targeting vimentin in colorectal cancer. *British journal of cancer* **2020**, *123*, 1123-1130, doi:10.1038/s41416-020-0940-5.
41. Beretta, F.; Huang, Y.F.; Punga, A.R. Towards Personalized Medicine in Myasthenia Gravis: Role of Circulating microRNAs miR-30e-5p, miR-150-5p and miR-21-5p. *Cells* **2022**, *11*, doi:10.3390/cells11040740.
42. Ge, Y.; Hong, M.; Zhang, Y.; Wang, J.; Li, L.; Zhu, H.; Sheng, Y.; Wu, W.S.; Zhang, Z. miR-30e-5p regulates leukemia stem cell self-renewal through the Cyb561/ROS signaling pathway. *Haematologica* **2024**, *109*, 411-421, doi:10.3324/haematol.2023.282837.
43. Liang, Z.; Tang, S.; He, R.; Luo, W.; Qin, S.; Jiang, H. The effect and mechanism of miR-30e-5p targeting SNAI1 to regulate epithelial-mesenchymal transition on pancreatic cancer. *Bioengineered* **2022**, *13*, 8013-8028, doi:10.1080/21655979.2022.2050880.
44. Bhattacharya, S.; Steele, R.; Shrivastava, S.; Chakraborty, S.; Di Bisceglie, A.M.; Ray, R.B. Serum miR-30e and miR-223 as Novel Noninvasive Biomarkers for Hepatocellular Carcinoma. *The American journal of pathology* **2016**, *186*, 242-247, doi:10.1016/j.ajpath.2015.10.003.
45. Chen, Q.; Li, Y.; Lu, T.; Luo, J.; Yang, L.; Zhou, Z.; Tian, Z.; Tan, S.; Liu, Q. miR-373 promotes invasion and metastasis of colorectal cancer cells via activating ERK/MAPK pathway. *Scientific reports* **2024**, *14*, 124, doi:10.1038/s41598-023-49565-5.
46. Shah, J.A.; Khattak, S.; Rauf, M.A.; Cai, Y.; Jin, J. Potential Biomarkers of miR-371-373 Gene Cluster in Tumorigenesis. *Life* **2021**, *11*, doi:10.3390/life11090984.

47. Lu, Y.; Li, X.; Zuo, Y.; Xu, Q.; Liu, L.; Wu, H.; Chen, L.; Zhang, Y.; Liu, Y.; Li, Y. miR-373-3p inhibits epithelial-mesenchymal transition via regulation of TGFbetaR2 in choriocarcinoma. *The journal of obstetrics and gynaecology research* **2021**, *47*, 2417-2432, doi:10.1111/jog.14809.
48. Liao, H.; Liang, Y.; Kang, L.; Xiao, Y.; Yu, T.; Wan, R. miR-454-3p inhibits non-small cell lung cancer cell proliferation and metastasis by targeting TGFB2. *Oncology reports* **2021**, *45*, doi:10.3892/or.2021.8018.
49. Song, Y.; Guo, Q.; Gao, S.; Hua, K. miR-454-3p promotes proliferation and induces apoptosis in human cervical cancer cells by targeting TRIM3. *Biochemical and biophysical research communications* **2019**, *516*, 872-879, doi:10.1016/j.bbrc.2019.06.126.
50. Zhang, K.; Yang, R.; Chen, J.; Qi, E.; Zhou, S.; Wang, Y.; Fu, Q.; Chen, R.; Fang, X. Let-7i-5p Regulation of Cell Morphology and Migration Through Distinct Signaling Pathways in Normal and Pathogenic Urethral Fibroblasts. *Frontiers in bioengineering and biotechnology* **2020**, *8*, 428, doi:10.3389/fbioe.2020.00428.
51. Liu, Y.; Hu, X.; Hu, L.; Xu, C.; Liang, X. Let-7i-5p enhances cell proliferation, migration and invasion of ccRCC by targeting HABP4. *BMC urology* **2021**, *21*, 49, doi:10.1186/s12894-021-00820-9.
52. Carreras-Badosa, G.; Maslovskaja, J.; Vaher, H.; Pajusaar, L.; Annilo, T.; Lattekivi, F.; Hubenthal, M.; Rodriguez, E.; Weidinger, S.; Kingo, K.; et al. miRNA expression profiles of the perilesional skin of atopic dermatitis and psoriasis patients are highly similar. *Scientific reports* **2022**, *12*, 22645, doi:10.1038/s41598-022-27235-2.
53. Na, C.; Li, X.; Zhang, J.; Han, L.; Li, Y.; Zhang, H. miR-107 targets TRIAP1 to regulate oral squamous cell carcinoma proliferation and migration. *International journal of clinical and experimental pathology* **2019**, *12*, 1820-1825.
54. Wang, L.; Li, K.; Wang, C.; Shi, X.; Yang, H. miR-107 regulates growth and metastasis of gastric cancer cells via activation of the PI3K-AKT signaling pathway by down-regulating FAT4. *Cancer medicine* **2019**, *8*, 5264-5273, doi:10.1002/cam4.2396.
55. Puente-Rivera, J.; De la Rosa Perez, D.A.; Olvera, S.I.N.; Figueroa-Angulo, E.E.; Saucedo, J.G.C.; Hernandez-Leon, O.; Alvarez-Sanchez, M.E. The Circulating miR-107 as a Potential Biomarker Up-Regulated in Castration-Resistant Prostate Cancer. *Non-coding RNA* **2024**, *10*, doi:10.3390/ncrna10050047.
56. Shi, J.; Ren, Y.; Liu, Y.; Cheng, Y.; Liu, Y. Circulating miR-3135b and miR-107 are potential biomarkers for severe hypertension. *Journal of human hypertension* **2021**, *35*, 343-350, doi:10.1038/s41371-020-0338-0.
57. Hu, Y.; Yan, J. Aberrant expression and mechanism of miR-130b-3p/phosphatase and tensin homolog in nephroblastoma in children. *Experimental and therapeutic medicine* **2019**, *18*, 1021-1028, doi:10.3892/etm.2019.7643.
58. Song, D.; Zhang, Q.; Zhang, H.; Zhan, L.; Sun, X. MiR-130b-3p promotes colorectal cancer progression by targeting CHD9. *Cell cycle* **2022**, *21*, 585-601, doi:10.1080/15384101.2022.2029240.
59. Huang, S.; Xue, P.; Han, X.; Zhang, C.; Yang, L.; Liu, L.; Wang, X.; Li, H.; Fu, J.; Zhou, Y. Exosomal miR-130b-3p targets SIK1 to inhibit medulloblastoma tumorigenesis. *Cell death & disease* **2020**, *11*, 408, doi:10.1038/s41419-020-2621-y.
60. Tylden, E.S.; Delgado, A.B.; Lukic, M.; Moi, L.; Busund, L.R.; Pedersen, M.I.; Lombardi, A.P.; Olsen, K.S. Roles of miR-20a-5p in breast cancer based on the clinical and multi-omic (CAMO) cohort and in vitro studies. *Scientific reports* **2024**, *14*, 25022, doi:10.1038/s41598-024-75557-0.
61. Huang, W.; Wu, X.; Xiang, S.; Qiao, M.; Cen, X.; Pan, X.; Huang, X.; Zhao, Z. Regulatory mechanism of miR-20a-5p expression in Cancer. *Cell death discovery* **2022**, *8*, 262, doi:10.1038/s41420-022-01005-5.

62. Lee, Y.J.; Hong, J.W.; Kim, Y.; Kim, J.; Kang, C.W.; Lee, M.H.; Moon, J.H.; Kim, E.H.; Ku, C.R.; Lee, E.J. Circulating miR-20a-5p as a biomarker associated with cabergoline responsiveness in patients with hyperprolactinemia and pituitary adenomas. *European journal of endocrinology* **2025**, *192*, 335-345, doi:10.1093/ejendo/lvaf025.
63. Salem, M.; Shan, Y.; Bernaudo, S.; Peng, C. miR-590-3p Targets Cyclin G2 and FOXO3 to Promote Ovarian Cancer Cell Proliferation, Invasion, and Spheroid Formation. *International journal of molecular sciences* **2019**, *20*, doi:10.3390/ijms20081810.
64. Wang, W.T.; Qi, Q.; Zhao, P.; Li, C.Y.; Yin, X.Y.; Yan, R.B. miR-590-3p is a novel microRNA which suppresses osteosarcoma progression by targeting SOX9. *Biomedicine & pharmacotherapy = Biomedecine & pharmacotherapie* **2018**, *107*, 1763-1769, doi:10.1016/j.biopha.2018.06.124.
65. Hu, B.; Mao, Z.; Du, Q.; Jiang, X.; Wang, Z.; Xiao, Z.; Zhu, D.; Wang, X.; Zhu, Y.; Wang, H. miR-93-5p targets Smad7 to regulate the transforming growth factor-beta1/Smad3 pathway and mediate fibrosis in drug-resistant prolactinoma. *Brain research bulletin* **2019**, *149*, 21-31, doi:10.1016/j.brainresbull.2019.03.013.
66. Wang, J.; Wu, Y.; Uddin, M.N.; Hao, J.P.; Chen, R.; Xiong, D.Q.; Ding, N.; Yang, J.H.; Wang, J.H.; Ding, X.S. Identification of MiR-93-5p Targeted Pathogenic Markers in Acute Myeloid Leukemia through Integrative Bioinformatics Analysis and Clinical Validation. *Journal of oncology* **2021**, *2021*, 5531736, doi:10.1155/2021/5531736.
67. Zhou, G.; Zeng, Y.; Luo, Y.; Guo, S.; Bao, L.; Zhang, Q. Urine miR-93-5p is a promising biomarker for early detection of HBV-related hepatocellular carcinoma. *European journal of surgical oncology : the journal of the European Society of Surgical Oncology and the British Association of Surgical Oncology* **2022**, *48*, 95-102, doi:10.1016/j.ejso.2021.06.015.
68. Gilles, M.E.; Slack, F.J. Let-7 microRNA as a potential therapeutic target with implications for immunotherapy. *Expert opinion on therapeutic targets* **2018**, *22*, 929-939, doi:10.1080/14728222.2018.1535594.
69. Alrashed, M.M.; Alshehry, A.S.; Ahmad, M.; He, J.; Wang, Y.; Xu, Y. miRNA Let-7a-5p targets RNA KCNQ1OT1 and Participates in Osteoblast Differentiation to Improve the Development of Osteoporosis. *Biochemical genetics* **2022**, *60*, 370-381, doi:10.1007/s10528-021-10105-3.
70. Fadhil, R.S.; Wei, M.Q.; Nikolarakos, D.; Good, D.; Nair, R.G. Salivary microRNA miR-let-7a-5p and miR-3928 could be used as potential diagnostic bio-markers for head and neck squamous cell carcinoma. *PloS one* **2020**, *15*, e0221779, doi:10.1371/journal.pone.0221779.
71. Koeppen, K.; Nymon, A.; Barnaby, R.; Bashor, L.; Li, Z.; Hampton, T.H.; Liefeld, A.E.; Kolling, F.W.; LaCroix, I.S.; Gerber, S.A.; et al. Let-7b-5p in vesicles secreted by human airway cells reduces biofilm formation and increases antibiotic sensitivity of P. aeruginosa. *Proceedings of the National Academy of Sciences of the United States of America* **2021**, *118*, doi:10.1073/pnas.2105370118.
72. Han, S.; Fang, J.; Yu, L.; Li, B.; Hu, Y.; Chen, R.; Li, C.; Zhao, C.; Li, J.; Wang, Y.; et al. Serum-derived exosomal hsa-let-7b-5p as a biomarker for predicting the severity of coronary stenosis in patients with coronary heart disease and hyperglycemia. *Molecular medicine reports* **2023**, *28*, doi:10.3892/mmr.2023.13090.
73. Gao, X.; Liu, H.; Wang, R.; Huang, M.; Wu, Q.; Wang, Y.; Zhang, W.; Liu, Y. Hsa-let-7d-5p Promotes Gastric Cancer Progression by Targeting PRDM5. *Journal of oncology* **2022**, *2022*, 2700651, doi:10.1155/2022/2700651.
74. Bonek, K.; Kuca Warnawin, E.; Kornatka, A.; Plebanczyk, M.; Burakowski, T.; Maslinski, W.; Wislowska, M.; Gluszko, P.; Ciechomska, M. Circulating miRNA Correlates with Lipid Profile and Disease Activity in Psoriatic Arthritis, Rheumatoid Arthritis, and Ankylosing Spondylitis Patients. *Biomedicines* **2022**, *10*, doi:10.3390/biomedicines10040893.

75. Li, Z.H.; Wang, Y.F.; He, D.D.; Zhang, X.M.; Zhou, Y.L.; Yue, H.; Huang, S.; Fu, Z.; Zhang, L.Y.; Mao, Z.Q.; et al. Let-7f-5p suppresses Th17 differentiation via targeting STAT3 in multiple sclerosis. *Aging* **2019**, *11*, 4463-4477, doi:10.18632/aging.102093.
76. Liu, B.; Li, C.; Bo, Y.; Tian, G.; Yang, L.; Si, J.; Zhang, L.; Yan, Y. Let-7f-5p Regulated by Hsa_circ_0000437 Ameliorates Bleomycin-Induced Skin Fibrosis. *Journal of cellular biochemistry* **2024**, *125*, e30629, doi:10.1002/jcb.30629.
77. Huang, S.; Lv, Z.; Guo, Y.; Li, L.; Zhang, Y.; Zhou, L.; Yang, B.; Wu, S.; Zhang, Y.; Xie, C.; et al. Identification of Blood Let-7e-5p as a Biomarker for Ischemic Stroke. *PloS one* **2016**, *11*, e0163951, doi:10.1371/journal.pone.0163951.
78. Okamura, T.; Okada, H.; Hashimoto, Y.; Majima, S.; Senmaru, T.; Nakanishi, N.; Asano, M.; Yamazaki, M.; Hamaguchi, M.; Fukui, M. Let-7e-5p Regulates IGF2BP2, and Induces Muscle Atrophy. *Frontiers in endocrinology* **2021**, *12*, 791363, doi:10.3389/fendo.2021.791363.
79. Li, C.Y.; Pang, Y.Y.; Yang, H.; Li, J.; Lu, H.X.; Wang, H.L.; Mo, W.J.; Huang, L.S.; Feng, Z.B.; Chen, G. Identification of miR-101-3p targets and functional features based on bioinformatics, meta-analysis and experimental verification in hepatocellular carcinoma. *American journal of translational research* **2017**, *9*, 2088-2105.
80. Krivosova, M.; Adamcakova, J.; Kaadt, E.; Mumm, B.H.; Dvorska, D.; Brany, D.; Dankova, Z.; Dohal, M.; Samec, M.; Ferencova, N.; et al. The VEGF protein levels, miR-101-3p, and miR-122-5p are dysregulated in plasma from adolescents with major depression. *Journal of affective disorders* **2023**, *334*, 60-68, doi:10.1016/j.jad.2023.04.094.
81. Sagar, S.K. miR-106b as an emerging therapeutic target in cancer. *Genes & diseases* **2022**, *9*, 889-899, doi:10.1016/j.gendis.2021.02.002.
82. Farre, P.L.; Duca, R.B.; Massillo, C.; Dalton, G.N.; Grana, K.D.; Gardner, K.; Lacunza, E.; De Siervi, A. MiR-106b-5p: A Master Regulator of Potential Biomarkers for Breast Cancer Aggressiveness and Prognosis. *International journal of molecular sciences* **2021**, *22*, doi:10.3390/ijms222011135.
83. Jia, X.; Wang, X.; Guo, X.; Ji, J.; Lou, G.; Zhao, J.; Zhou, W.; Guo, M.; Zhang, M.; Li, C.; et al. MicroRNA-124: An emerging therapeutic target in cancer. *Cancer medicine* **2019**, *8*, 5638-5650, doi:10.1002/cam4.2489.
84. He, R.Q.; Yang, X.; Liang, L.; Chen, G.; Ma, J. MicroRNA-124-3p expression and its prospective functional pathways in hepatocellular carcinoma: A quantitative polymerase chain reaction, gene expression omnibus and bioinformatics study. *Oncology letters* **2018**, *15*, 5517-5532, doi:10.3892/ol.2018.8045.
85. Chai, F.; Peng, H.; Qin, L.; Liu, C.; Zeng, Y.; Wang, R.; Xu, G.; Wang, R.; Wei, G.; Huang, H.; et al. MicroRNA miR-181d-5p regulates the MAPK signaling pathway by targeting mitogen-activated protein kinase 8 (MAPK8) to improve lupus nephritis. *Gene* **2024**, *893*, 147961, doi:10.1016/j.gene.2023.147961.
86. Carneiro, V.; Cirino, M.; Panepucci, R.; Peria, F.; Tirapelli, D.; Colli, B.; Carlotti, C.G., Jr. The Role of MicroRNA 181d as a Possible Biomarker Associated With Tumor Progression in Meningiomas. *Cureus* **2021**, *13*, e19158, doi:10.7759/cureus.19158.
87. Niu, F.; Kazimierska, M.; Nolte, I.M.; Terpstra, M.M.; de Jong, D.; Koerts, J.; van der Sluis, T.; Rutgers, B.; O'Connell, R.M.; Kok, K.; et al. The miR-26b-5p/KPNA2 Axis Is an Important Regulator of Burkitt Lymphoma Cell Growth. *Cancers* **2020**, *12*, doi:10.3390/cancers12061464.
88. Han, W.; Li, N.; Liu, J.; Sun, Y.; Yang, X.; Wang, Y. MicroRNA-26b-5p enhances T cell responses by targeting PIM-2 in hepatocellular carcinoma. *Cellular signalling* **2019**, *59*, 182-190, doi:10.1016/j.cellsig.2018.11.011.

89. Liu, Q.; Wu, D.H.; Han, L.; Deng, J.W.; Zhou, L.; He, R.; Lu, C.J.; Mi, Q.S. Roles of microRNAs in psoriasis: Immunological functions and potential biomarkers. *Experimental dermatology* **2017**, *26*, 359-367, doi:10.1111/exd.13249.
90. Oda, T.; Tsutsumi, K.; Obata, T.; Ueta, E.; Kikuchi, T.; Ako, S.; Fujii, Y.; Yamazaki, T.; Uchida, D.; Matsumoto, K.; et al. MicroRNA-34a-5p: A pivotal therapeutic target in gallbladder cancer. *Molecular therapy. Oncology* **2024**, *32*, 200765, doi:10.1016/j.omton.2024.200765.
91. Barati, T.; Mirzaei, Z.; Ebrahimi, A.; Shekari Khaniani, M.; Mansoori Derakhshan, S. miR-449a: A Promising Biomarker and Therapeutic Target in Cancer and Other Diseases. *Cell biochemistry and biophysics* **2024**, *82*, 1629-1650, doi:10.1007/s12013-024-01322-9.
92. Yang, X.; Feng, M.; Jiang, X.; Wu, Z.; Li, Z.; Aau, M.; Yu, Q. miR-449a and miR-449b are direct transcriptional targets of E2F1 and negatively regulate pRb-E2F1 activity through a feedback loop by targeting CDK6 and CDC25A. *Genes & development* **2009**, *23*, 2388-2393, doi:10.1101/gad.1819009.
93. Zhao, J.; Wang, H.; Zhou, J.; Qian, J.; Yang, H.; Zhou, Y.; Ding, H.; Gong, Y.; Qi, X.; Jiao, Y.; et al. miR-130a-3p, a Preclinical Therapeutic Target for Crohn's Disease. *Journal of Crohn's & colitis* **2021**, *15*, 647-664, doi:10.1093/ecco-jcc/jjaa204.
94. Fan, Q.; Huang, T.; Sun, X.; Yang, X.; Wang, J.; Liu, Y.; Ni, T.; Gu, S.; Li, Y.; Wang, Y. miR-130a-3p promotes cell proliferation and invasion by targeting estrogen receptor alpha and androgen receptor in cervical cancer. *Experimental and therapeutic medicine* **2021**, *21*, 414, doi:10.3892/etm.2021.9858.
95. Shintani, T.; Shun, Y.T.; Toyozumi, Y.; Ikemura, K.; Shiroyama, T.; Nagatomo, I.; Jingushi, K.; Takeda, Y.; Kumanogoh, A.; Okuda, M. MicroRNA-130a-3p regulates osimertinib resistance by targeting runt-related transcription factor 3 in lung adenocarcinoma. *Scientific reports* **2024**, *14*, 24429, doi:10.1038/s41598-024-76196-1.
96. Ding, X.; Chen, L.; Xu, D.; Yu, Y.; Tao, X.; Fan, Y.; Huang, Y. Pan-Cancer Analysis of BUB1B/hsa-miR-130a-3p Axis and Identification of Circulating hsa-miR-130a-3p as a Potential Biomarker for Cancer Risk Assessment. *Evidence-based complementary and alternative medicine : eCAM* **2022**, *2022*, 3261300, doi:10.1155/2022/3261300.
97. Wang, Y.; Li, Y.; Ni, D.; Wei, Z.; Fu, Z.; Li, C.; Sun, H.; Wu, Y.; Li, Y.; Zhang, Y.; et al. miR-186-5p targets TGFbetaR2 to inhibit RAW264.7 cell migration and proliferation during mouse skin wound healing. *Environmental toxicology* **2023**, *38*, 2826-2835, doi:10.1002/tox.23914.
98. Li, J.; Xia, L.; Zhou, Z.; Zuo, Z.; Xu, C.; Song, H.; Cai, J. MiR-186-5p upregulation inhibits proliferation, metastasis and epithelial-to-mesenchymal transition of colorectal cancer cell by targeting ZEB1. *Archives of biochemistry and biophysics* **2018**, *640*, 53-60, doi:10.1016/j.abb.2018.01.002.
99. Rodrigues, B.; Leitao, R.A.; Santos, M.; Trofimov, A.; Silva, M.; Inacio, A.S.; Abreu, M.; Nobre, R.J.; Costa, J.; Cardoso, A.L.; et al. MiR-186-5p inhibition restores synaptic transmission and neuronal network activity in a model of chronic stress. *Molecular psychiatry* **2025**, *30*, 1034-1046, doi:10.1038/s41380-024-02715-1.
100. Sil, A.; Chakraborty, D. miRNA: The Next Frontier in Dermatology Research and Therapeutics. *Indian journal of dermatology* **2024**, *69*, 486, doi:10.4103/ijd.ijd_568_23.
101. Cheng, Q.; Li, Q.; Xu, L.; Jiang, H. Exosomal microRNA-301a-3p promotes the proliferation and invasion of nasopharyngeal carcinoma cells by targeting BTG1 mRNA. *Molecular medicine reports* **2021**, *23*, doi:10.3892/mmr.2021.11967.
102. Guo, J.; Zhong, X.; Tan, Q.; Yang, S.; Liao, J.; Zhuge, J.; Hong, Z.; Deng, Q.; Zuo, Q. miR-301a-3p induced by endoplasmic reticulum stress mediates the occurrence and transmission of trastuzumab resistance in HER2-positive gastric cancer. *Cell death & disease* **2021**, *12*, 696, doi:10.1038/s41419-021-03991-3.

103. Liu, T.; Ma, M. Exploring miR-301a-3p and osteosarcoma: from expression differences to mechanism of action. *Discover oncology* **2025**, *16*, 751, doi:10.1007/s12672-025-02345-1.
104. Li, P.; Xing, W.; Xu, J.; Yuan, D.; Liang, G.; Liu, B.; Ma, H. microRNA-301b-3p downregulation underlies a novel inhibitory role of long non-coding RNA MBNL1-AS1 in non-small cell lung cancer. *Stem cell research & therapy* **2019**, *10*, 144, doi:10.1186/s13287-019-1235-8.
105. Fan, Y.; Li, Y.; Zhu, Y.; Dai, G.; Wu, D.; Gao, Z.; Zhang, L.; Xu, D. miR-301b-3p Regulates Breast Cancer Cell Proliferation, Migration, and Invasion by Targeting NR3C2. *Journal of oncology* **2021**, *2021*, 8810517, doi:10.1155/2021/8810517.
106. Qi, W.; Niu, N.; Zhao, J.; Liu, H.; Yang, F. MiR-301b-3p can be used as a Potential Marker for the Diagnosis of Lung Adenocarcinoma. *Combinatorial chemistry & high throughput screening* **2024**, *27*, 1131-1139, doi:10.2174/1386207326666230821112230.
107. Eun, J.W.; Kim, H.S.; Shen, Q.; Yang, H.D.; Kim, S.Y.; Yoon, J.H.; Park, W.S.; Lee, J.Y.; Nam, S.W. MicroRNA-495-3p functions as a tumor suppressor by regulating multiple epigenetic modifiers in gastric carcinogenesis. *The Journal of pathology* **2018**, *244*, 107-119, doi:10.1002/path.4994.
108. Chen, S.; Wu, J.; Jiao, K.; Wu, Q.; Ma, J.; Chen, D.; Kang, J.; Zhao, G.; Shi, Y.; Fan, D.; et al. MicroRNA-495-3p inhibits multidrug resistance by modulating autophagy through GRP78/mTOR axis in gastric cancer. *Cell death & disease* **2018**, *9*, 1070, doi:10.1038/s41419-018-0950-x.
