# Supplementary material for: Decoding Non-Coding RNA Regulators in DITRA: From Genomic Insights to Potential Biomarkers and Therapeutic Targets
Source: Genes (Basel). 2025 Jun 27;16(7):753. doi: 10.3390/genes16070753 (PMC12295128; doi:10.3390/genes16070753)
Supplement: Supplementary file 1 [file genes-16-00753-s001.zip › Supplementary-TableS6.pdf]

Description	Database	ID	p.adjust
Diseases of signal transduction by growth factor receptors and second messengers	Reactome Pathway	R-HSA-5663202	2.34E-36
Signaling by Interleukins	Reactome Pathway	R-HSA-449147	2.59E-35
Interleukin-4 and Interleukin-13 signaling	Reactome Pathway	R-HSA-6785807	2.26E-31
Intracellular signaling by second messengers	Reactome Pathway	R-HSA-9006925	7.25E-25
PIP3 activates AKT signaling	Reactome Pathway	R-HSA-1257604	5.23E-24
PI3K/AKT Signaling in Cancer	Reactome Pathway	R-HSA-2219528	1.08E-21
Signaling by NTRKs	Reactome Pathway	R-HSA-166520	7.95E-21
MAPK family signaling cascades	Reactome Pathway	R-HSA-5683057	1.39E-19
Negative regulation of the PI3K/AKT network	Reactome Pathway	R-HSA-199418	2.52E-19
FOXO-mediated transcription	Reactome Pathway	R-HSA-9614085	1.23E-18
Signaling by NTRK1 (TRKA)	Reactome Pathway	R-HSA-187037	5.86E-18
MyD88:MAL(TIRAP) cascade initiated on plasma membrane	Reactome Pathway	R-HSA-166058	1.12E-17
Toll Like Receptor TLR6:TLR2 Cascade	Reactome Pathway	R-HSA-168188	1.12E-17
Signaling by VEGF	Reactome Pathway	R-HSA-194138	2.93E-17
Toll Like Receptor 4 (TLR4) Cascade	Reactome Pathway	R-HSA-166016	6.08E-17
Toll Like Receptor TLR1:TLR2 Cascade	Reactome Pathway	R-HSA-168179	8.83E-17
Toll Like Receptor 2 (TLR2) Cascade	Reactome Pathway	R-HSA-181438	8.83E-17
Toll Like Receptor 3 (TLR3) Cascade	Reactome Pathway	R-HSA-168164	1.41E-16
PI5P, PP2A and IER3 Regulate PI3K/AKT Signaling	Reactome Pathway	R-HSA-6811558	1.53E-16
MyD88-independent TLR4 cascade	Reactome Pathway	R-HSA-166166	1.53E-16
VEGFA-VEGFR2 Pathway	Reactome Pathway	R-HSA-4420097	1.53E-16
TRIF(TICAM1)-mediated TLR4 signaling	Reactome Pathway	R-HSA-937061	1.53E-16
Signaling by PDGF	Reactome Pathway	R-HSA-186797	2.38E-16
ESR-mediated signaling	Reactome Pathway	R-HSA-8939211	9.81E-16
Signaling by Nuclear Receptors	Reactome Pathway	R-HSA-9006931	9.93E-16
TRAF6 mediated induction of NFkB and MAP kinases upon TLR7/8 or 9 activation	Reactome Pathway	R-HSA-975138	2.26E-15
Toll-like Receptor Cascades	Reactome Pathway	R-HSA-168898	2.35E-15
Constitutive Signaling by Aberrant PI3K in Cancer	Reactome Pathway	R-HSA-2219530	3.91E-15
MyD88 dependent cascade initiated on endosome	Reactome Pathway	R-HSA-975155	4.37E-15
Extracellular matrix organization	Reactome Pathway	R-HSA-1474244	4.63E-15
Toll Like Receptor 7/8 (TLR7/8) Cascade	Reactome Pathway	R-HSA-168181	8.65E-15
MAPK1/MAPK3 signaling	Reactome Pathway	R-HSA-5684996	1.23E-14
Toll Like Receptor 10 (TLR10) Cascade	Reactome Pathway	R-HSA-168142	4.85E-14
Toll Like Receptor 5 (TLR5) Cascade	Reactome Pathway	R-HSA-168176	4.85E-14
MyD88 cascade initiated on plasma membrane	Reactome Pathway	R-HSA-975871	4.85E-14

Toll Like Receptor 9 (TLR9) Cascade	Reactome Pathway	R-HSA-168138	6.35E-14
RAF/MAP kinase cascade	Reactome Pathway	R-HSA-5673001	2.26E-13
Extra-nuclear estrogen signaling	Reactome Pathway	R-HSA-9009391	3.63E-13
Transcriptional Regulation by TP53	Reactome Pathway	R-HSA-3700989	1.69E-12
Signaling by TGFB family members	Reactome Pathway	R-HSA-9006936	1.96E-12
Downstream signal transduction	Reactome Pathway	R-HSA-186763	2.35E-12
MAP kinase activation	Reactome Pathway	R-HSA-450294	4.12E-12
Interleukin-17 signaling	Reactome Pathway	R-HSA-448424	9.23E-12
Insulin receptor signalling cascade	Reactome Pathway	R-HSA-74751	1.10E-11
Nuclear Events (kinase and transcription factor activation)	Reactome Pathway	R-HSA-198725	1.41E-11
Integrin cell surface interactions	Reactome Pathway	R-HSA-216083	1.61E-11
IGF1R signaling cascade	Reactome Pathway	R-HSA-2428924	3.14E-11
Oncogenic MAPK signaling	Reactome Pathway	R-HSA-6802957	4.03E-11
Oncogene Induced Senescence	Reactome Pathway	R-HSA-2559585	6.47E-11
Signaling by TGF-beta Receptor Complex	Reactome Pathway	R-HSA-170834	6.81E-11
Signaling by Type 1 Insulin-like Growth Factor 1 Receptor (IGF1R)	Reactome Pathway	R-HSA-2404192	7.13E-11
IRS-related events triggered by IGF1R	Reactome Pathway	R-HSA-2428928	8.58E-11
SUMO E3 ligases SUMOylate target proteins	Reactome Pathway	R-HSA-3108232	9.99E-11
SUMOylation	Reactome Pathway	R-HSA-2990846	1.02E-10
L1CAM interactions	Reactome Pathway	R-HSA-373760	2.52E-10
Cellular Senescence	Reactome Pathway	R-HSA-2559583	2.89E-10
Signaling by moderate kinase activity BRAF mutants	Reactome Pathway	R-HSA-6802946	3.30E-10
Signaling by RAS mutants	Reactome Pathway	R-HSA-6802949	3.30E-10
Paradoxical activation of RAF signaling by kinase inactive BRAF	Reactome Pathway	R-HSA-6802955	3.30E-10
Signaling downstream of RAS mutants	Reactome Pathway	R-HSA-9649948	3.30E-10
Non-integrin membrane-ECM interactions	Reactome Pathway	R-HSA-3000171	5.47E-10
Signaling by MET	Reactome Pathway	R-HSA-6806834	7.26E-10
IRS-mediated signalling	Reactome Pathway	R-HSA-112399	7.80E-10
Intrinsic Pathway for Apoptosis	Reactome Pathway	R-HSA-109606	8.79E-10
Signaling by FLT3 ITD and TKD mutants	Reactome Pathway	R-HSA-9703648	1.86E-09
Cyclin D associated events in G1	Reactome Pathway	R-HSA-69231	2.18E-09
G1 Phase	Reactome Pathway	R-HSA-69236	2.18E-09
Signal transduction by L1	Reactome Pathway	R-HSA-445144	2.86E-09
Programmed Cell Death	Reactome Pathway	R-HSA-5357801	2.89E-09
Signaling by SCF-KIT	Reactome Pathway	R-HSA-1433557	3.15E-09
Signaling by BRAF and RAF1 fusions	Reactome Pathway	R-HSA-6802952	4.28E-09

Platelet activation, signaling and aggregation	Reactome Pathway	R-HSA-76002	4.82E-09
Signaling by NTRK2 (TRKB)	Reactome Pathway	R-HSA-9006115	5.26E-09
CD28 co-stimulation	Reactome Pathway	R-HSA-389356	5.87E-09
Diseases of Immune System	Reactome Pathway	R-HSA-5260271	5.87E-09
Diseases associated with the TLR signaling cascade	Reactome Pathway	R-HSA-5602358	5.87E-09
NCAM signaling for neurite out-growth	Reactome Pathway	R-HSA-375165	7.36E-09
Signaling by KIT in disease	Reactome Pathway	R-HSA-9669938	9.52E-09
Signaling by phosphorylated juxtamembrane, extracellular and kinase domain KIT mutants	Reactome Pathway	R-HSA-9670439	9.52E-09
Regulation of PTEN gene transcription	Reactome Pathway	R-HSA-8943724	9.52E-09
EPH-Ephrin signaling	Reactome Pathway	R-HSA-2682334	9.52E-09
Signaling by FGFR3	Reactome Pathway	R-HSA-5654741	9.90E-09
Apoptosis	Reactome Pathway	R-HSA-109581	1.11E-08
FLT3 Signaling	Reactome Pathway	R-HSA-9607240	1.16E-08
Signaling by high-kinase activity BRAF mutants	Reactome Pathway	R-HSA-6802948	1.34E-08
Signaling by RAF1 mutants	Reactome Pathway	R-HSA-9656223	1.80E-08
FOXO-mediated transcription of cell cycle genes	Reactome Pathway	R-HSA-9617828	1.80E-08
Aberrant regulation of mitotic G1/S transition in cancer due to RB1 defects	Reactome Pathway	R-HSA-9659787	1.80E-08
Defective binding of RB1 mutants to E2F1,(E2F2, E2F3)	Reactome Pathway	R-HSA-9661069	1.80E-08
Signaling by FGFR1	Reactome Pathway	R-HSA-5654736	1.82E-08
Signaling by WNT	Reactome Pathway	R-HSA-195721	2.05E-08
MET promotes cell motility	Reactome Pathway	R-HSA-8875878	2.16E-08
Nucleotide-binding domain, leucine rich repeat containing receptor (NLR) signaling pathways	Reactome Pathway	R-HSA-168643	2.18E-08
Fcgamma receptor (FCGR) dependent phagocytosis	Reactome Pathway	R-HSA-2029480	2.31E-08
NGF-stimulated transcription	Reactome Pathway	R-HSA-9031628	2.58E-08
Death Receptor Signalling	Reactome Pathway	R-HSA-73887	2.66E-08
PI3K Cascade	Reactome Pathway	R-HSA-109704	3.73E-08
Signaling by PTK6	Reactome Pathway	R-HSA-8848021	5.15E-08
Signaling by Non-Receptor Tyrosine Kinases	Reactome Pathway	R-HSA-9006927	5.15E-08
Downstream signaling of activated FGFR3	Reactome Pathway	R-HSA-5654708	5.28E-08
Signaling by Erythropoietin	Reactome Pathway	R-HSA-9006335	5.28E-08
Signaling by ALK	Reactome Pathway	R-HSA-201556	5.28E-08
Syndecan interactions	Reactome Pathway	R-HSA-3000170	5.28E-08
MAP2K and MAPK activation	Reactome Pathway	R-HSA-5674135	5.59E-08
RET signaling	Reactome Pathway	R-HSA-8853659	5.59E-08

FOXO-mediated transcription of cell death genes	Reactome Pathway	R-HSA-9614657	6.11E-08
Nuclear Receptor transcription pathway	Reactome Pathway	R-HSA-383280	6.44E-08
Signaling by Insulin receptor	Reactome Pathway	R-HSA-74752	7.97E-08
Signaling by ERBB2	Reactome Pathway	R-HSA-1227986	8.43E-08
Signaling by EGFR	Reactome Pathway	R-HSA-177929	8.43E-08
Signaling by WNT in cancer	Reactome Pathway	R-HSA-4791275	9.90E-08
Gastrin-CREB signalling pathway via PKC and MAPK	Reactome Pathway	R-HSA-881907	1.01E-07
FCERI mediated MAPK activation	Reactome Pathway	R-HSA-2871796	1.15E-07
RUNX2 regulates bone development	Reactome Pathway	R-HSA-8941326	1.15E-07
Signaling by FGFR4	Reactome Pathway	R-HSA-5654743	1.16E-07
Mitotic G1 phase and G1/S transition	Reactome Pathway	R-HSA-453279	1.24E-07
Transcriptional activity of SMAD2/SMAD3:SMAD4 heterotrimer	Reactome Pathway	R-HSA-2173793	1.42E-07
Signaling by NOTCH1	Reactome Pathway	R-HSA-1980143	1.45E-07
CD28 dependent PI3K/Akt signaling	Reactome Pathway	R-HSA-389357	1.46E-07
Signaling by FGFR3 in disease	Reactome Pathway	R-HSA-5655332	1.46E-07
Signaling by FGFR3 point mutants in cancer	Reactome Pathway	R-HSA-8853338	1.46E-07
Estrogen-dependent nuclear events downstream of ESR-membrane signaling	Reactome Pathway	R-HSA-9634638	1.53E-07
Degradation of the extracellular matrix	Reactome Pathway	R-HSA-1474228	1.65E-07
Potential therapeutics for SARS	Reactome Pathway	R-HSA-9679191	1.68E-07
NOD1/2 Signaling Pathway	Reactome Pathway	R-HSA-168638	1.74E-07
Collagen degradation	Reactome Pathway	R-HSA-1442490	1.81E-07
EPHB-mediated forward signaling	Reactome Pathway	R-HSA-3928662	2.29E-07
Interleukin-10 signaling	Reactome Pathway	R-HSA-6783783	2.63E-07
Regulation of TP53 Activity	Reactome Pathway	R-HSA-5633007	2.77E-07
Semaphorin interactions	Reactome Pathway	R-HSA-373755	3.04E-07
MAPK targets/ Nuclear events mediated by MAP kinases	Reactome Pathway	R-HSA-450282	3.04E-07
Disassembly of the destruction complex and recruitment of AXIN to the membrane	Reactome Pathway	R-HSA-4641262	3.04E-07
Signaling by PDGFRA transmembrane, juxtamembrane and kinase domain mutants	Reactome Pathway	R-HSA-9673767	3.04E-07
Signaling by PDGFRA extracellular domain mutants	Reactome Pathway	R-HSA-9673770	3.04E-07
Regulation of RUNX1 Expression and Activity	Reactome Pathway	R-HSA-8934593	3.16E-07
Signaling by NTRK3 (TRKC)	Reactome Pathway	R-HSA-9034015	3.16E-07
VEGFR2 mediated vascular permeability	Reactome Pathway	R-HSA-5218920	3.49E-07
Signaling by FLT3 fusion proteins	Reactome Pathway	R-HSA-9703465	4.04E-07
Constitutive Signaling by AKT1 E17K in Cancer	Reactome Pathway	R-HSA-5674400	4.33E-07
Negative regulation of MAPK pathway	Reactome Pathway	R-HSA-5675221	4.33E-07
VEGFR2 mediated cell proliferation	Reactome Pathway	R-HSA-5218921	4.43E-07

RAF activation	Reactome Pathway	R-HSA-5673000	4.44E-07
RAF-independent MAPK1/3 activation	Reactome Pathway	R-HSA-112409	4.44E-07
TP53 Regulates Transcription of Cell Cycle Genes	Reactome Pathway	R-HSA-6791312	4.59E-07
Estrogen-dependent gene expression	Reactome Pathway	R-HSA-9018519	5.00E-07
SMAD2/SMAD3:SMAD4 heterotrimer regulates transcription	Reactome Pathway	R-HSA-2173796	6.91E-07
Transcriptional regulation by RUNX2	Reactome Pathway	R-HSA-8878166	7.49E-07
Interleukin-2 family signaling	Reactome Pathway	R-HSA-451927	8.25E-07
SUMOylation of intracellular receptors	Reactome Pathway	R-HSA-4090294	8.28E-07
Downstream signaling of activated FGFR2	Reactome Pathway	R-HSA-5654696	8.28E-07
Transcriptional Regulation by MECP2	Reactome Pathway	R-HSA-8986944	8.86E-07
FLT3 signaling in disease	Reactome Pathway	R-HSA-9682385	9.84E-07
Fc epsilon receptor (FCERI) signaling	Reactome Pathway	R-HSA-2454202	9.90E-07
Signaling by ERBB2 ECD mutants	Reactome Pathway	R-HSA-9665348	1.06E-06
Signaling by ERBB2 in Cancer	Reactome Pathway	R-HSA-1227990	1.13E-06
RUNX2 regulates osteoblast differentiation	Reactome Pathway	R-HSA-8940973	1.27E-06
Cell surface interactions at the vascular wall	Reactome Pathway	R-HSA-202733	1.30E-06
Signaling by PDGFR in disease	Reactome Pathway	R-HSA-9671555	1.38E-06
TAK1 activates NFkB by phosphorylation and activation of IKKs complex	Reactome Pathway	R-HSA-445989	1.48E-06
Signaling by FGFR1 in disease	Reactome Pathway	R-HSA-5655302	1.86E-06
FOXO-mediated transcription of oxidative stress, metabolic and neuronal genes	Reactome Pathway	R-HSA-9615017	2.33E-06
G0 and Early G1	Reactome Pathway	R-HSA-1538133	2.81E-06
Integrin signaling	Reactome Pathway	R-HSA-354192	2.81E-06
Downstream signaling of activated FGFR4	Reactome Pathway	R-HSA-5654716	2.81E-06
Signaling by FGFR	Reactome Pathway	R-HSA-190236	3.05E-06
RHO GTPase cycle	Reactome Pathway	R-HSA-9012999	3.12E-06
Signaling by ERBB2 KD Mutants	Reactome Pathway	R-HSA-9664565	3.29E-06
Defective Intrinsic Pathway for Apoptosis	Reactome Pathway	R-HSA-9734009	3.29E-06
Platelet Aggregation (Plug Formation)	Reactome Pathway	R-HSA-76009	3.55E-06
Negative regulation of TCF-dependent signaling by WNT ligand antagonists	Reactome Pathway	R-HSA-3772470	3.57E-06
Constitutive Signaling by EGFRvIII	Reactome Pathway	R-HSA-5637810	3.57E-06
Signaling by EGFRvIII in Cancer	Reactome Pathway	R-HSA-5637812	3.57E-06
Apoptotic execution phase	Reactome Pathway	R-HSA-75153	4.25E-06
Activation of the AP-1 family of transcription factors	Reactome Pathway	R-HSA-450341	4.25E-06
Signaling by FGFR3 fusions in cancer	Reactome Pathway	R-HSA-8853334	4.25E-06
STAT5 activation downstream of FLT3 ITD mutants	Reactome Pathway	R-HSA-9702518	4.25E-06
Regulation of TP53 Expression and Degradation	Reactome Pathway	R-HSA-6806003	4.59E-06

Deactivation of the beta-catenin transactivating complex	Reactome Pathway	R-HSA-3769402	4.78E-06
MET activates PTK2 signaling	Reactome Pathway	R-HSA-8874081	4.91E-06
Signaling by ALK in cancer	Reactome Pathway	R-HSA-9700206	4.95E-06
Signaling by ALK fusions and activated point mutants	Reactome Pathway	R-HSA-9725370	4.95E-06
PTEN Regulation	Reactome Pathway	R-HSA-6807070	5.19E-06
Signaling by ERBB4	Reactome Pathway	R-HSA-1236394	5.63E-06
Signaling by NOTCH1 PEST Domain Mutants in Cancer	Reactome Pathway	R-HSA-2644602	5.63E-06
Signaling by NOTCH1 in Cancer	Reactome Pathway	R-HSA-2644603	5.63E-06
Constitutive Signaling by NOTCH1 PEST Domain Mutants	Reactome Pathway	R-HSA-2644606	5.63E-06
Signaling by NOTCH1 HD+PEST Domain Mutants in Cancer	Reactome Pathway	R-HSA-2894858	5.63E-06
Constitutive Signaling by NOTCH1 HD+PEST Domain Mutants	Reactome Pathway	R-HSA-2894862	5.63E-06
Elastic fibre formation	Reactome Pathway	R-HSA-1566948	6.01E-06
Assembly of collagen fibrils and other multimeric structures	Reactome Pathway	R-HSA-2022090	6.40E-06
Regulation of actin dynamics for phagocytic cup formation	Reactome Pathway	R-HSA-2029482	6.40E-06
Transcriptional regulation of white adipocyte differentiation	Reactome Pathway	R-HSA-381340	7.80E-06
Diseases of mitotic cell cycle	Reactome Pathway	R-HSA-9675126	8.30E-06
Circadian Clock	Reactome Pathway	R-HSA-400253	8.65E-06
EPH-ephrin mediated repulsion of cells	Reactome Pathway	R-HSA-3928665	9.14E-06
Downstream signaling of activated FGFR1	Reactome Pathway	R-HSA-5654687	9.85E-06
Regulated Necrosis	Reactome Pathway	R-HSA-5218859	9.96E-06
SHC1 events in ERBB2 signaling	Reactome Pathway	R-HSA-1250196	1.03E-05
Regulation of TP53 Degradation	Reactome Pathway	R-HSA-6804757	1.09E-05
Aberrant regulation of mitotic cell cycle due to RB1 defects	Reactome Pathway	R-HSA-9687139	1.09E-05
FRS-mediated FGFR3 signaling	Reactome Pathway	R-HSA-5654706	1.17E-05
Negative regulation of FGFR3 signaling	Reactome Pathway	R-HSA-5654732	1.23E-05
RORA activates gene expression	Reactome Pathway	R-HSA-1368082	1.23E-05
Tie2 Signaling	Reactome Pathway	R-HSA-210993	1.23E-05
SHC-mediated cascade:FGFR3	Reactome Pathway	R-HSA-5654704	1.23E-05
PI-3K cascade:FGFR3	Reactome Pathway	R-HSA-5654710	1.23E-05
TNF receptor superfamily (TNFSF) members mediating non-canonical NF-kB pathway	Reactome Pathway	R-HSA-5676594	1.23E-05
Signaling by NOTCH	Reactome Pathway	R-HSA-157118	1.38E-05
Signalling to ERKs	Reactome Pathway	R-HSA-187687	1.42E-05
Transcriptional Regulation by VENTX	Reactome Pathway	R-HSA-8853884	1.45E-05
NR1H2 and NR1H3-mediated signaling	Reactome Pathway	R-HSA-9024446	1.67E-05
Heme signaling	Reactome Pathway	R-HSA-9707616	1.67E-05
Costimulation by the CD28 family	Reactome Pathway	R-HSA-388841	1.71E-05

Signaling by EGFR in Cancer	Reactome Pathway	R-HSA-1643713	1.95E-05
TRAF6 mediated NF-kB activation	Reactome Pathway	R-HSA-933542	1.95E-05
Collagen formation	Reactome Pathway	R-HSA-1474290	2.14E-05
Activation of BH3-only proteins	Reactome Pathway	R-HSA-114452	2.46E-05
DAP12 signaling	Reactome Pathway	R-HSA-2424491	2.46E-05
Laminin interactions	Reactome Pathway	R-HSA-3000157	2.46E-05
GPVI-mediated activation cascade	Reactome Pathway	R-HSA-114604	2.62E-05
Signaling by FGFR2	Reactome Pathway	R-HSA-5654738	2.62E-05
NOTCH1 Intracellular Domain Regulates Transcription	Reactome Pathway	R-HSA-2122947	2.69E-05
Interleukin-3, Interleukin-5 and GM-CSF signaling	Reactome Pathway	R-HSA-512988	2.69E-05
ECM proteoglycans	Reactome Pathway	R-HSA-3000178	2.69E-05
TCF dependent signaling in response to WNT	Reactome Pathway	R-HSA-201681	2.72E-05
DDX58/IFIH1-mediated induction of interferon-alpha/beta	Reactome Pathway	R-HSA-168928	2.78E-05
CTLA4 inhibitory signaling	Reactome Pathway	R-HSA-389513	2.78E-05
Interleukin-6 signaling	Reactome Pathway	R-HSA-1059683	2.78E-05
Signaling by Leptin	Reactome Pathway	R-HSA-2586552	2.78E-05
Regulation by c-FLIP	Reactome Pathway	R-HSA-3371378	2.78E-05
Signaling by FGFR4 in disease	Reactome Pathway	R-HSA-5655291	2.78E-05
Dimerization of procaspase-8	Reactome Pathway	R-HSA-69416	2.78E-05
Constitutive Signaling by Overexpressed ERBB2	Reactome Pathway	R-HSA-9634285	2.78E-05
Parasite infection	Reactome Pathway	R-HSA-9664407	2.90E-05
Leishmania phagocytosis	Reactome Pathway	R-HSA-9664417	2.90E-05
FCGR3A-mediated phagocytosis	Reactome Pathway	R-HSA-9664422	2.90E-05
Notch-HLH transcription pathway	Reactome Pathway	R-HSA-350054	3.03E-05
Regulation of lipid metabolism by PPARalpha	Reactome Pathway	R-HSA-400206	3.10E-05
IRAK4 deficiency (TLR2/4)	Reactome Pathway	R-HSA-5603041	3.18E-05
Apoptotic cleavage of cellular proteins	Reactome Pathway	R-HSA-111465	3.24E-05
Regulation of MECP2 expression and activity	Reactome Pathway	R-HSA-9022692	3.30E-05
SARS-CoV Infections	Reactome Pathway	R-HSA-9679506	3.30E-05
RIP-mediated NFkB activation via ZBP1	Reactome Pathway	R-HSA-1810476	3.52E-05
Cell-Cell communication	Reactome Pathway	R-HSA-1500931	3.52E-05
PPARA activates gene expression	Reactome Pathway	R-HSA-1989781	4.16E-05
Regulation of necroptotic cell death	Reactome Pathway	R-HSA-5675482	4.42E-05
Interleukin-6 family signaling	Reactome Pathway	R-HSA-6783589	4.98E-05
Growth hormone receptor signaling	Reactome Pathway	R-HSA-982772	4.98E-05
Regulation of cholesterol biosynthesis by SREBP (SREBF)	Reactome Pathway	R-HSA-1655829	5.07E-05

Activation of NMDA receptors and postsynaptic events	Reactome Pathway	R-HSA-442755	5.07E-05
Caspase activation via extrinsic apoptotic signalling pathway	Reactome Pathway	R-HSA-5357769	5.89E-05
Negative regulation of FGFR2 signaling	Reactome Pathway	R-HSA-5654727	5.98E-05
Deregulated CDK5 triggers multiple neurodegenerative pathways in Alzheimer's disease models	Reactome Pathway	R-HSA-8862803	6.20E-05
Neurodegenerative Diseases	Reactome Pathway	R-HSA-8863678	6.20E-05
Signaling by ERBB2 TMD/JMD mutants	Reactome Pathway	R-HSA-9665686	6.20E-05
Interleukin-1 family signaling	Reactome Pathway	R-HSA-446652	6.25E-05
Oxidative Stress Induced Senescence	Reactome Pathway	R-HSA-2559580	6.31E-05
NCAM1 interactions	Reactome Pathway	R-HSA-419037	6.60E-05
RHOB GTPase cycle	Reactome Pathway	R-HSA-9013026	6.62E-05
Apoptotic factor-mediated response	Reactome Pathway	R-HSA-111471	7.63E-05
Nuclear signaling by ERBB4	Reactome Pathway	R-HSA-1251985	8.00E-05
MyD88 deficiency (TLR2/4)	Reactome Pathway	R-HSA-5602498	9.18E-05
TP53 Regulates Transcription of Genes Involved in G2 Cell Cycle Arrest	Reactome Pathway	R-HSA-6804114	9.18E-05
Clathrin-mediated endocytosis	Reactome Pathway	R-HSA-8856828	9.28E-05
RHOC GTPase cycle	Reactome Pathway	R-HSA-9013106	9.37E-05
MAPK3 (ERK1) activation	Reactome Pathway	R-HSA-110056	9.37E-05
ATF6 (ATF6-alpha) activates chaperone genes	Reactome Pathway	R-HSA-381183	9.37E-05
Signal attenuation	Reactome Pathway	R-HSA-74749	9.37E-05
Interleukin-21 signaling	Reactome Pathway	R-HSA-9020958	9.37E-05
Regulation of FOXO transcriptional activity by acetylation	Reactome Pathway	R-HSA-9617629	9.37E-05
Sema4D in semaphorin signaling	Reactome Pathway	R-HSA-400685	9.86E-05
FRS-mediated FGFR2 signaling	Reactome Pathway	R-HSA-5654700	9.86E-05
Transcriptional Regulation by E2F6	Reactome Pathway	R-HSA-8953750	1.00E-04
Myogenesis	Reactome Pathway	R-HSA-525793	0.000105
TNFR1-induced NFkappaB signaling pathway	Reactome Pathway	R-HSA-5357956	0.000105
Erythropoietin activates Phosphoinositide-3-kinase (PI3K)	Reactome Pathway	R-HSA-9027276	0.00011
SHC1 events in EGFR signaling	Reactome Pathway	R-HSA-180336	0.00011
AKT phosphorylates targets in the cytosol	Reactome Pathway	R-HSA-198323	0.00011
TP53 Regulates Transcription of Genes Involved in G1 Cell Cycle Arrest	Reactome Pathway	R-HSA-6804116	0.00011
PTK6 Regulates RHO GTPases, RAS GTPase and MAP kinases	Reactome Pathway	R-HSA-8849471	0.00011
Erythropoietin activates RAS	Reactome Pathway	R-HSA-9027284	0.00011
TNF signaling	Reactome Pathway	R-HSA-75893	0.000116
Molecules associated with elastic fibres	Reactome Pathway	R-HSA-2129379	0.00012
RUNX1 interacts with co-factors whose precise effect on RUNX1 targets is not known	Reactome Pathway	R-HSA-8939243	0.00012

JNK (c-Jun kinases) phosphorylation and activation mediated by activated human TAK1	Reactome Pathway	R-HSA-450321	0.000124
FRS-mediated FGFR1 signaling	Reactome Pathway	R-HSA-5654693	0.000124
PI-3K cascade:FGFR2	Reactome Pathway	R-HSA-5654695	0.000124
SHC-mediated cascade:FGFR2	Reactome Pathway	R-HSA-5654699	0.000124
Negative regulation of FGFR1 signaling	Reactome Pathway	R-HSA-5654726	0.000133
Regulation of beta-cell development	Reactome Pathway	R-HSA-186712	0.00014
TP53 Regulates Transcription of Cell Death Genes	Reactome Pathway	R-HSA-5633008	0.000158
ZBP1(DAI) mediated induction of type I IFNs	Reactome Pathway	R-HSA-1606322	0.000158
CD209 (DC-SIGN) signaling	Reactome Pathway	R-HSA-5621575	0.000158
SHC-mediated cascade:FGFR1	Reactome Pathway	R-HSA-5654688	0.000158
PI-3K cascade:FGFR1	Reactome Pathway	R-HSA-5654689	0.000158
Macroautophagy	Reactome Pathway	R-HSA-1632852	0.000172
Recycling pathway of L1	Reactome Pathway	R-HSA-437239	0.000175
Negative regulation of FGFR4 signaling	Reactome Pathway	R-HSA-5654733	0.000181
G1/S Transition	Reactome Pathway	R-HSA-69206	0.000193
Constitutive Signaling by Ligand-Responsive EGFR Cancer Variants	Reactome Pathway	R-HSA-1236382	0.000199
Caspase activation via Death Receptors in the presence of ligand	Reactome Pathway	R-HSA-140534	0.000199
Signaling by Ligand-Responsive EGFR Variants in Cancer	Reactome Pathway	R-HSA-5637815	0.000199
Transcriptional regulation by RUNX1	Reactome Pathway	R-HSA-8878171	0.000213
Activation of gene expression by SREBF (SREBP)	Reactome Pathway	R-HSA-2426168	0.000218
RIPK1-mediated regulated necrosis	Reactome Pathway	R-HSA-5213460	0.000223
Response to elevated platelet cytosolic Ca2+	Reactome Pathway	R-HSA-76005	0.000224
Ca2+ pathway	Reactome Pathway	R-HSA-4086398	0.000225
Post NMDA receptor activation events	Reactome Pathway	R-HSA-438064	0.000225
SUMOylation of transcription cofactors	Reactome Pathway	R-HSA-3899300	0.00024
GAB1 signalosome	Reactome Pathway	R-HSA-180292	0.000242
Beta-catenin phosphorylation cascade	Reactome Pathway	R-HSA-196299	0.000242
Autophagy	Reactome Pathway	R-HSA-9612973	0.000245
NR1H3 & NR1H2 regulate gene expression linked to cholesterol transport and efflux	Reactome Pathway	R-HSA-9029569	0.000261
Anchoring fibril formation	Reactome Pathway	R-HSA-2214320	0.000289
GRB2:SOS provides linkage to MAPK signaling for Integrins	Reactome Pathway	R-HSA-354194	0.000289
p130Cas linkage to MAPK signaling for integrins	Reactome Pathway	R-HSA-372708	0.000289
PERK regulates gene expression	Reactome Pathway	R-HSA-381042	0.000303
Platelet degranulation	Reactome Pathway	R-HSA-114608	0.000311
Signaling by FGFR in disease	Reactome Pathway	R-HSA-1226099	0.000313
ERK/MAPK targets	Reactome Pathway	R-HSA-198753	0.000313

FRS-mediated FGFR4 signaling	Reactome Pathway	R-HSA-5654712	0.000313
GRB2 events in EGFR signaling	Reactome Pathway	R-HSA-179812	0.000321
CASP8 activity is inhibited	Reactome Pathway	R-HSA-5218900	0.000321
TNFR1-induced proapoptotic signaling	Reactome Pathway	R-HSA-5357786	0.000321
BMAL1:CLOCK,NPAS2 activates circadian gene expression	Reactome Pathway	R-HSA-1368108	0.000321
Endogenous sterols	Reactome Pathway	R-HSA-211976	0.000321
Interleukin receptor SHC signaling	Reactome Pathway	R-HSA-912526	0.000321
MET activates RAS signaling	Reactome Pathway	R-HSA-8851805	0.000332
MET activates RAP1 and RAC1	Reactome Pathway	R-HSA-8875555	0.000332
Activated NTRK2 signals through FRS2 and FRS3	Reactome Pathway	R-HSA-9028731	0.000332
Regulation of localization of FOXO transcription factors	Reactome Pathway	R-HSA-9614399	0.000332
Signaling by NOTCH3	Reactome Pathway	R-HSA-9012852	0.000374
Signalling to RAS	Reactome Pathway	R-HSA-167044	0.000399
Signaling by Hippo	Reactome Pathway	R-HSA-2028269	0.000399
SUMOylation of transcription factors	Reactome Pathway	R-HSA-3232118	0.000399
SHC-mediated cascade:FGFR4	Reactome Pathway	R-HSA-5654719	0.000399
PI-3K cascade:FGFR4	Reactome Pathway	R-HSA-5654720	0.000399
Transcriptional regulation by the AP-2 (TFAP2) family of transcription factors	Reactome Pathway	R-HSA-8864260	0.000399
Cyclin A/B1/B2 associated events during G2/M transition	Reactome Pathway	R-HSA-69273	0.00043
Pre-NOTCH Processing in Golgi	Reactome Pathway	R-HSA-1912420	0.000523
G1/S-Specific Transcription	Reactome Pathway	R-HSA-69205	0.000562
Transcriptional activation of mitochondrial biogenesis	Reactome Pathway	R-HSA-2151201	0.000577
Cargo recognition for clathrin-mediated endocytosis	Reactome Pathway	R-HSA-8856825	0.000608
TP53 Regulates Metabolic Genes	Reactome Pathway	R-HSA-5628897	0.000641
Role of LAT2/NTAL/LAB on calcium mobilization	Reactome Pathway	R-HSA-2730905	0.000667
FGFR1 mutant receptor activation	Reactome Pathway	R-HSA-1839124	0.00068
RHO GTPase Effectors	Reactome Pathway	R-HSA-195258	0.000745
Unfolded Protein Response (UPR)	Reactome Pathway	R-HSA-381119	0.000752
Sema4D induced cell migration and growth-cone collapse	Reactome Pathway	R-HSA-416572	0.000796
Regulation of IFNG signaling	Reactome Pathway	R-HSA-877312	0.000827
Neutrophil degranulation	Reactome Pathway	R-HSA-6798695	0.000831
RHOU GTPase cycle	Reactome Pathway	R-HSA-9013420	0.00096
ATF6 (ATF6-alpha) activates chaperones	Reactome Pathway	R-HSA-381033	0.000983
CD28 dependent Vav1 pathway	Reactome Pathway	R-HSA-389359	0.000983
Transcription of E2F targets under negative control by DREAM complex	Reactome Pathway	R-HSA-1362277	0.00106
Ephrin signaling	Reactome Pathway	R-HSA-3928664	0.00106

activated TAK1 mediates p38 MAPK activation	Reactome Pathway	R-HSA-450302	0.00106
Transcriptional regulation of pluripotent stem cells	Reactome Pathway	R-HSA-452723	0.00106
STAT3 nuclear events downstream of ALK signaling	Reactome Pathway	R-HSA-9701898	0.00108
Transcriptional regulation by RUNX3	Reactome Pathway	R-HSA-8878159	0.00121
ATF4 activates genes in response to endoplasmic reticulum stress	Reactome Pathway	R-HSA-380994	0.00132
MECP2 regulates neuronal receptors and channels	Reactome Pathway	R-HSA-9022699	0.00142
Signaling by NODAL	Reactome Pathway	R-HSA-1181150	0.00147
Regulation of signaling by CBL	Reactome Pathway	R-HSA-912631	0.00147
p75 NTR receptor-mediated signalling	Reactome Pathway	R-HSA-193704	0.00153
Leishmania infection	Reactome Pathway	R-HSA-9658195	0.00163
Deubiquitination	Reactome Pathway	R-HSA-5688426	0.00182
Signaling by Activin	Reactome Pathway	R-HSA-1502540	0.00182
YAP1- and WWTR1 (TAZ)-stimulated gene expression	Reactome Pathway	R-HSA-2032785	0.00182
Signaling by CTNNB1 phospho-site mutants	Reactome Pathway	R-HSA-4839743	0.00182
Signaling by GSK3beta mutants	Reactome Pathway	R-HSA-5339716	0.00182
S33 mutants of beta-catenin aren't phosphorylated	Reactome Pathway	R-HSA-5358747	0.00182
S37 mutants of beta-catenin aren't phosphorylated	Reactome Pathway	R-HSA-5358749	0.00182
S45 mutants of beta-catenin aren't phosphorylated	Reactome Pathway	R-HSA-5358751	0.00182
T41 mutants of beta-catenin aren't phosphorylated	Reactome Pathway	R-HSA-5358752	0.00182
TFAP2 (AP-2) family regulates transcription of growth factors and their receptors	Reactome Pathway	R-HSA-8866910	0.00182
Interleukin-7 signaling	Reactome Pathway	R-HSA-1266695	0.00184
Signaling by BMP	Reactome Pathway	R-HSA-201451	0.00213
Regulation of TP53 Activity through Phosphorylation	Reactome Pathway	R-HSA-6804756	0.00213
Cytochrome c-mediated apoptotic response	Reactome Pathway	R-HSA-111461	0.00234
p38MAPK events	Reactome Pathway	R-HSA-171007	0.00234
Phospholipase C-mediated cascade; FGFR3	Reactome Pathway	R-HSA-5654227	0.00234
Chk1/Chk2(Cds1) mediated inactivation of Cyclin B:Cdk1 complex	Reactome Pathway	R-HSA-75035	0.00234
EGFR downregulation	Reactome Pathway	R-HSA-182971	0.00237
C-type lectin receptors (CLRs)	Reactome Pathway	R-HSA-5621481	0.00237
Interleukin-1 signaling	Reactome Pathway	R-HSA-9020702	0.00269
Crosslinking of collagen fibrils	Reactome Pathway	R-HSA-2243919	0.00269
Regulation of TNFR1 signaling	Reactome Pathway	R-HSA-5357905	0.00272
Signaling by FGFR2 in disease	Reactome Pathway	R-HSA-5655253	0.00287
Regulation of gene expression by Hypoxia-inducible Factor	Reactome Pathway	R-HSA-1234158	0.00287
Regulated proteolysis of p75NTR	Reactome Pathway	R-HSA-193692	0.00287
Receptor Mediated Mitophagy	Reactome Pathway	R-HSA-8934903	0.00287

NOTCH4 Activation and Transmission of Signal to the Nucleus	Reactome Pathway	R-HSA-9013700	0.00287
Mitophagy	Reactome Pathway	R-HSA-5205647	0.00332
Regulation of gene expression in beta cells	Reactome Pathway	R-HSA-210745	0.00348
Cell junction organization	Reactome Pathway	R-HSA-446728	0.0035
TGF-beta receptor signaling activates SMADs	Reactome Pathway	R-HSA-2173789	0.00356
Spry regulation of FGF signaling	Reactome Pathway	R-HSA-1295596	0.00356
Transcription of E2F targets under negative control by p107 (RBL1) and p130 (RBL2) in complex with HDAC1	Reactome Pathway	R-HSA-1362300	0.00356
Regulation of KIT signaling	Reactome Pathway	R-HSA-1433559	0.00356
Polo-like kinase mediated events	Reactome Pathway	R-HSA-156711	0.00356
p75NTR signals via NF-kB	Reactome Pathway	R-HSA-193639	0.00356
GRB2 events in ERBB2 signaling	Reactome Pathway	R-HSA-1963640	0.00356
PI3K events in ERBB2 signaling	Reactome Pathway	R-HSA-1963642	0.00356
TGF-beta receptor signaling in EMT (epithelial to mesenchymal transition)	Reactome Pathway	R-HSA-2173791	0.00356
Uptake and function of anthrax toxins	Reactome Pathway	R-HSA-5210891	0.00356
RHOQ GTPase cycle	Reactome Pathway	R-HSA-9013406	0.00357
RAC2 GTPase cycle	Reactome Pathway	R-HSA-9013404	0.00363
Regulation of Insulin-like Growth Factor (IGF) transport and uptake by Insulin-like Growth Factor Binding Proteins (IGFBPs)	Reactome Pathway	R-HSA-381426	0.00364
Beta-catenin independent WNT signaling	Reactome Pathway	R-HSA-3858494	0.00382
Uptake and actions of bacterial toxins	Reactome Pathway	R-HSA-5339562	0.00383
Netrin-1 signaling	Reactome Pathway	R-HSA-373752	0.00384
MTOR signalling	Reactome Pathway	R-HSA-165159	0.00389
Collagen chain trimerization	Reactome Pathway	R-HSA-8948216	0.0039
RHO GTPases activate PAKs	Reactome Pathway	R-HSA-5627123	0.00398
RHO GTPases Activate NADPH Oxidases	Reactome Pathway	R-HSA-5668599	0.00398
Pre-NOTCH Expression and Processing	Reactome Pathway	R-HSA-1912422	0.00437
S Phase	Reactome Pathway	R-HSA-69242	0.00443
Downregulation of SMAD2/3:SMAD4 transcriptional activity	Reactome Pathway	R-HSA-2173795	0.0045
Nuclear events stimulated by ALK signaling in cancer	Reactome Pathway	R-HSA-9725371	0.00462
SHC1 events in ERBB4 signaling	Reactome Pathway	R-HSA-1250347	0.00462
Signaling by AXIN mutants	Reactome Pathway	R-HSA-4839735	0.00462
Signaling by APC mutants	Reactome Pathway	R-HSA-4839744	0.00462
Signaling by AMER1 mutants	Reactome Pathway	R-HSA-4839748	0.00462
APC truncation mutants have impaired AXIN binding	Reactome Pathway	R-HSA-5467337	0.00462
AXIN missense mutants destabilize the destruction complex	Reactome Pathway	R-HSA-5467340	0.00462

Truncations of AMER1 destabilize the destruction complex	Reactome Pathway	R-HSA-5467348	0.00462
Regulation of TP53 Activity through Association with Co-factors	Reactome Pathway	R-HSA-6804759	0.00462
RUNX3 regulates NOTCH signaling	Reactome Pathway	R-HSA-8941856	0.00462
Interleukin-15 signaling	Reactome Pathway	R-HSA-8983432	0.00462
Transmission across Chemical Synapses	Reactome Pathway	R-HSA-112315	0.0047
Regulation of TP53 Activity through Acetylation	Reactome Pathway	R-HSA-6804758	0.00475
Signaling by CSF3 (G-CSF)	Reactome Pathway	R-HSA-9674555	0.00475
Activation of Matrix Metalloproteinases	Reactome Pathway	R-HSA-1592389	0.00502
RHO GTPases Activate WASPs and WAVEs	Reactome Pathway	R-HSA-5663213	0.00518
DAP12 interactions	Reactome Pathway	R-HSA-2172127	0.00521
Collagen biosynthesis and modifying enzymes	Reactome Pathway	R-HSA-1650814	0.00528
Listeria monocytogenes entry into host cells	Reactome Pathway	R-HSA-8876384	0.00544
RHOG GTPase cycle	Reactome Pathway	R-HSA-9013408	0.00596
Signaling by activated point mutants of FGFR3	Reactome Pathway	R-HSA-1839130	0.00596
Gap junction degradation	Reactome Pathway	R-HSA-190873	0.00596
FGFR3 mutant receptor activation	Reactome Pathway	R-HSA-2033514	0.00596
PECAM1 interactions	Reactome Pathway	R-HSA-210990	0.00596
Caspase-mediated cleavage of cytoskeletal proteins	Reactome Pathway	R-HSA-264870	0.00596
Repression of WNT target genes	Reactome Pathway	R-HSA-4641265	0.00596
TP53 Regulates Transcription of Caspase Activators and Caspases	Reactome Pathway	R-HSA-6803207	0.00596
TP53 Regulates Transcription of Death Receptors and Ligands	Reactome Pathway	R-HSA-6803211	0.00596
Interleukin-2 signaling	Reactome Pathway	R-HSA-9020558	0.00596
Killing mechanisms	Reactome Pathway	R-HSA-9664420	0.00596
WNT5:FZD7-mediated leishmania damping	Reactome Pathway	R-HSA-9673324	0.00596
ALK mutants bind TKIs	Reactome Pathway	R-HSA-9700645	0.00596
NOTCH3 Intracellular Domain Regulates Transcription	Reactome Pathway	R-HSA-9013508	0.00601
RHOJ GTPase cycle	Reactome Pathway	R-HSA-9013409	0.00625
Activated NOTCH1 Transmits Signal to the Nucleus	Reactome Pathway	R-HSA-2122948	0.00689
RAB geranylgeranylation	Reactome Pathway	R-HSA-8873719	0.00698
RHO GTPases Activate ROCKs	Reactome Pathway	R-HSA-5627117	0.00748
TP53 Regulates Transcription of Genes Involved in Cytochrome C Release	Reactome Pathway	R-HSA-6803204	0.00748
NOTCH4 Intracellular Domain Regulates Transcription	Reactome Pathway	R-HSA-9013695	0.00748
MET receptor recycling	Reactome Pathway	R-HSA-8875656	0.00774
Chemokine receptors bind chemokines	Reactome Pathway	R-HSA-380108	0.00774
Translocation of SLC2A4 (GLUT4) to the plasma membrane	Reactome Pathway	R-HSA-1445148	0.00777
Golgi Associated Vesicle Biogenesis	Reactome Pathway	R-HSA-432722	0.00812

Depolymerisation of the Nuclear Lamina	Reactome Pathway	R-HSA-4419969	0.00848
Response of EIF2AK1 (HRI) to heme deficiency	Reactome Pathway	R-HSA-9648895	0.00848
Cytochrome P450 - arranged by substrate type	Reactome Pathway	R-HSA-211897	0.0088
Diseases of programmed cell death	Reactome Pathway	R-HSA-9645723	0.00902
Downregulation of TGF-beta receptor signaling	Reactome Pathway	R-HSA-2173788	0.00913
Purinergic signaling in leishmaniasis infection	Reactome Pathway	R-HSA-9660826	0.00913
Cell recruitment (pro-inflammatory response)	Reactome Pathway	R-HSA-9664424	0.00913
CREB1 phosphorylation through NMDA receptor-mediated activation of RAS signaling	Reactome Pathway	R-HSA-442742	0.00959
RHO GTPases activate IQGAPs	Reactome Pathway	R-HSA-5626467	0.00979
RAC1 GTPase cycle	Reactome Pathway	R-HSA-9013149	0.00979
RHOH GTPase cycle	Reactome Pathway	R-HSA-9013407	0.00979
RHOV GTPase cycle	Reactome Pathway	R-HSA-9013424	0.00979
Interleukin-12 family signaling	Reactome Pathway	R-HSA-447115	0.0104
Phospholipase C-mediated cascade; FGFR2	Reactome Pathway	R-HSA-5654221	0.0104
Chromatin modifying enzymes	Reactome Pathway	R-HSA-3247509	0.0104
Chromatin organization	Reactome Pathway	R-HSA-4839726	0.0104
FGFR3 ligand binding and activation	Reactome Pathway	R-HSA-190239	0.0115
FGFR3c ligand binding and activation	Reactome Pathway	R-HSA-190372	0.0115
FGFR2c ligand binding and activation	Reactome Pathway	R-HSA-190375	0.0115
NF-kB is activated and signals survival	Reactome Pathway	R-HSA-209560	0.0115
NF-kB activation through FADD/RIP-1 pathway mediated by caspase-8 and -10	Reactome Pathway	R-HSA-933543	0.0115
Cytosolic sensors of pathogen-associated DNA	Reactome Pathway	R-HSA-1834949	0.0115
Global Genome Nucleotide Excision Repair (GG-NER)	Reactome Pathway	R-HSA-5696399	0.0115
Post-translational protein phosphorylation	Reactome Pathway	R-HSA-8957275	0.0115
Regulation of TLR by endogenous ligand	Reactome Pathway	R-HSA-5686938	0.0117
RHOA GTPase cycle	Reactome Pathway	R-HSA-8980692	0.012
Selective autophagy	Reactome Pathway	R-HSA-9663891	0.0124
RND3 GTPase cycle	Reactome Pathway	R-HSA-9696264	0.0127
RND1 GTPase cycle	Reactome Pathway	R-HSA-9696273	0.0127
Ca-dependent events	Reactome Pathway	R-HSA-111996	0.0131
EPHA-mediated growth cone collapse	Reactome Pathway	R-HSA-3928663	0.0135
FCERI mediated Ca+2 mobilization	Reactome Pathway	R-HSA-2871809	0.0135
Opioid Signalling	Reactome Pathway	R-HSA-111885	0.0142
Signal regulatory protein family interactions	Reactome Pathway	R-HSA-391160	0.0142
Sema3A PAK dependent Axon repulsion	Reactome Pathway	R-HSA-399954	0.0142
Phospholipase C-mediated cascade: FGFR1	Reactome Pathway	R-HSA-5654219	0.0142

MAP3K8 (TPL2)-dependent MAPK1/3 activation	Reactome Pathway	R-HSA-5684264	0.0142
The NLRP3 inflammasome	Reactome Pathway	R-HSA-844456	0.0142
IRAK2 mediated activation of TAK1 complex upon TLR7/8 or 9 stimulation	Reactome Pathway	R-HSA-975163	0.0142
Cellular response to heat stress	Reactome Pathway	R-HSA-3371556	0.0153
Formation of apoptosome	Reactome Pathway	R-HSA-111458	0.0153
Signaling by activated point mutants of FGFR1	Reactome Pathway	R-HSA-1839122	0.0153
Formation of annular gap junctions	Reactome Pathway	R-HSA-196025	0.0153
Apoptotic cleavage of cell adhesion proteins	Reactome Pathway	R-HSA-351906	0.0153
DSCAM interactions	Reactome Pathway	R-HSA-376172	0.0153
Type I hemidesmosome assembly	Reactome Pathway	R-HSA-446107	0.0153
SUMOylation of immune response proteins	Reactome Pathway	R-HSA-4755510	0.0153
TICAM1,TRAF6-dependent induction of TAK1 complex	Reactome Pathway	R-HSA-9014325	0.0153
Regulation of the apoptosome activity	Reactome Pathway	R-HSA-9627069	0.0153
Metabolism of steroids	Reactome Pathway	R-HSA-8957322	0.0162
DAG and IP3 signaling	Reactome Pathway	R-HSA-1489509	0.0165
Role of phospholipids in phagocytosis	Reactome Pathway	R-HSA-2029485	0.0182
Basigin interactions	Reactome Pathway	R-HSA-210991	0.0182
NOTCH3 Activation and Transmission of Signal to the Nucleus	Reactome Pathway	R-HSA-9013507	0.0182
Pyroptosis	Reactome Pathway	R-HSA-5620971	0.0185
Interferon alpha/beta signaling	Reactome Pathway	R-HSA-909733	0.0197
RAC3 GTPase cycle	Reactome Pathway	R-HSA-9013423	0.0197
Prolonged ERK activation events	Reactome Pathway	R-HSA-169893	0.0197
Attenuation phase	Reactome Pathway	R-HSA-3371568	0.0197
Defective EXT2 causes exostoses 2	Reactome Pathway	R-HSA-3656237	0.0197
Defective EXT1 causes exostoses 1, TRPS2 and CHDS	Reactome Pathway	R-HSA-3656253	0.0197
TP53 regulates transcription of several additional cell death genes whose specific roles in p53-dependent apoptosis remain uncertain	Reactome Pathway	R-HSA-6803205	0.0197
CLEC7A (Dectin-1) signaling	Reactome Pathway	R-HSA-5607764	0.0197
Interleukin-12 signaling	Reactome Pathway	R-HSA-9020591	0.0201
Senescence-Associated Secretory Phenotype (SASP)	Reactome Pathway	R-HSA-2559582	0.0211
RHOD GTPase cycle	Reactome Pathway	R-HSA-9013405	0.0226
Activated point mutants of FGFR2	Reactome Pathway	R-HSA-2033519	0.0226
SUMOylation of DNA methylation proteins	Reactome Pathway	R-HSA-4655427	0.0226
IL-6-type cytokine receptor ligand interactions	Reactome Pathway	R-HSA-6788467	0.0226
TRAF6-mediated induction of TAK1 complex within TLR4 complex	Reactome Pathway	R-HSA-937072	0.0226
Mitochondrial biogenesis	Reactome Pathway	R-HSA-1592230	0.0231

DNA Damage Recognition in GG-NER	Reactome Pathway	R-HSA-5696394	0.0232
Gene and protein expression by JAK-STAT signaling after Interleukin-12 stimulation	Reactome Pathway	R-HSA-8950505	0.0232
TICAM1, RIP1-mediated IKK complex recruitment	Reactome Pathway	R-HSA-168927	0.0243
Defective B4GALT7 causes EDS, progeroid type	Reactome Pathway	R-HSA-3560783	0.0243
Defective B3GAT3 causes JDSSDHD	Reactome Pathway	R-HSA-3560801	0.0243
Defective B3GALT6 causes EDSP2 and SEMDJL1	Reactome Pathway	R-HSA-4420332	0.0243
RHO GTPases activate CIT	Reactome Pathway	R-HSA-5625900	0.0243
Activation of the pre-replicative complex	Reactome Pathway	R-HSA-68962	0.0247
Energy dependent regulation of mTOR by LKB1-AMPK	Reactome Pathway	R-HSA-380972	0.0252
Downregulation of ERBB2 signaling	Reactome Pathway	R-HSA-8863795	0.0252
DARPP-32 events	Reactome Pathway	R-HSA-180024	0.0254
CaMK IV-mediated phosphorylation of CREB	Reactome Pathway	R-HSA-111932	0.0273
Tetrahydrobiopterin (BH4) synthesis, recycling, salvage and regulation	Reactome Pathway	R-HSA-1474151	0.0273
Frs2-mediated activation	Reactome Pathway	R-HSA-170968	0.0273
FGFR1c ligand binding and activation	Reactome Pathway	R-HSA-190373	0.0273
GP1b-IX-V activation signalling	Reactome Pathway	R-HSA-430116	0.0273
trans-Golgi Network Vesicle Budding	Reactome Pathway	R-HSA-199992	0.0283
Binding and Uptake of Ligands by Scavenger Receptors	Reactome Pathway	R-HSA-2173782	0.0283
RHOF GTPase cycle	Reactome Pathway	R-HSA-9035034	0.0283
Ovarian tumor domain proteases	Reactome Pathway	R-HSA-5689896	0.03
Activation of BAD and translocation to mitochondria	Reactome Pathway	R-HSA-111447	0.0315
Synthesis of bile acids and bile salts via 27-hydroxycholesterol	Reactome Pathway	R-HSA-193807	0.0315
WNT5A-dependent internalization of FZD4	Reactome Pathway	R-HSA-5099900	0.0315
Mismatch Repair	Reactome Pathway	R-HSA-5358508	0.0315
Phospholipase C-mediated cascade; FGFR4	Reactome Pathway	R-HSA-5654228	0.0315
TRAF3-dependent IRF activation pathway	Reactome Pathway	R-HSA-918233	0.0315
Plasma lipoprotein clearance	Reactome Pathway	R-HSA-8964043	0.0327
Interferon Signaling	Reactome Pathway	R-HSA-913531	0.0334
G-protein mediated events	Reactome Pathway	R-HSA-112040	0.0339
Signaling by cytosolic FGFR1 fusion mutants	Reactome Pathway	R-HSA-1839117	0.0339
Cell-extracellular matrix interactions	Reactome Pathway	R-HSA-446353	0.0339
Regulation of FZD by ubiquitination	Reactome Pathway	R-HSA-4641263	0.0349
Negative regulation of MET activity	Reactome Pathway	R-HSA-6807004	0.0349
Interleukin-37 signaling	Reactome Pathway	R-HSA-9008059	0.0349
Glutamate Neurotransmitter Release Cycle	Reactome Pathway	R-HSA-210500	0.0349
HSF1-dependent transactivation	Reactome Pathway	R-HSA-3371571	0.0349

Other interleukin signaling	Reactome Pathway	R-HSA-449836	0.0349
Inflammasomes	Reactome Pathway	R-HSA-622312	0.0349
Synthesis of PIPs at the plasma membrane	Reactome Pathway	R-HSA-1660499	0.0361
Interferon gamma signaling	Reactome Pathway	R-HSA-877300	0.0361
Epigenetic regulation of gene expression	Reactome Pathway	R-HSA-212165	0.0368
Ub-specific processing proteases	Reactome Pathway	R-HSA-5689880	0.037
Neurotransmitter receptors and postsynaptic signal transmission	Reactome Pathway	R-HSA-112314	0.0372
Activation of PPARGC1A (PGC-1alpha) by phosphorylation	Reactome Pathway	R-HSA-2151209	0.0373
MASTL Facilitates Mitotic Progression	Reactome Pathway	R-HSA-2465910	0.0373
TRIF-mediated programmed cell death	Reactome Pathway	R-HSA-2562578	0.0373
CREB1 phosphorylation through the activation of CaMKII/CaMKK/CaMKIV cascasde	Reactome Pathway	R-HSA-442729	0.0373
RUNX1 and FOXP3 control the development of regulatory T lymphocytes (Tregs)	Reactome Pathway	R-HSA-8877330	0.0373
IRAK2 mediated activation of TAK1 complex	Reactome Pathway	R-HSA-937042	0.0373
Smooth Muscle Contraction	Reactome Pathway	R-HSA-445355	0.0373
Calmodulin induced events	Reactome Pathway	R-HSA-111933	0.0396
CaM pathway	Reactome Pathway	R-HSA-111997	0.0396
Inhibition of replication initiation of damaged DNA by RB1/E2F1	Reactome Pathway	R-HSA-113501	0.0436
ERKs are inactivated	Reactome Pathway	R-HSA-202670	0.0436
p75NTR recruits signalling complexes	Reactome Pathway	R-HSA-209543	0.0436
POU5F1 (OCT4), SOX2, NANOG activate genes related to proliferation	Reactome Pathway	R-HSA-2892247	0.0436
CLEC7A (Dectin-1) induces NFAT activation	Reactome Pathway	R-HSA-5607763	0.0436
FGFRL1 modulation of FGFR1 signaling	Reactome Pathway	R-HSA-5658623	0.0436
Mitotic Telophase/Cytokinesis	Reactome Pathway	R-HSA-68884	0.0436
Dissolution of Fibrin Clot	Reactome Pathway	R-HSA-75205	0.0436
Advanced glycosylation endproduct receptor signaling	Reactome Pathway	R-HSA-879415	0.0436
Pyruvate metabolism	Reactome Pathway	R-HSA-70268	0.0436
EGR2 and SOX10-mediated initiation of Schwann cell myelination	Reactome Pathway	R-HSA-9619665	0.0456
Diseases associated with glycosaminoglycan metabolism	Reactome Pathway	R-HSA-3560782	0.0469
FGFR1 ligand binding and activation	Reactome Pathway	R-HSA-190242	0.0469
Regulation of gene expression in late stage (branching morphogenesis) pancreatic bud precursor cells	Reactome Pathway	R-HSA-210744	0.0469
CRMPs in Sema3A signaling	Reactome Pathway	R-HSA-399956	0.0469
IKK complex recruitment mediated by RIP1	Reactome Pathway	R-HSA-937041	0.0469
Pre-NOTCH Transcription and Translation	Reactome Pathway	R-HSA-1912408	0.0475
PINK1-PRKN Mediated Mitophagy	Reactome Pathway	R-HSA-5205685	0.0477
Other semaphorin interactions	Reactome Pathway	R-HSA-416700	0.0477

Regulation of TP53 Activity through Methylation	Reactome Pathway	R-HSA-6804760	0.0477
LDL clearance	Reactome Pathway	R-HSA-8964038	0.0477
Biosynthesis of specialized proresolving mediators (SPMs)	Reactome Pathway	R-HSA-9018678	0.0477
DNA Damage Bypass	Reactome Pathway	R-HSA-73893	0.0499
Proteoglycans in cancer	KEGG Pathway	hsa05205	9.85E-46
Hepatitis B	KEGG Pathway	hsa05161	4.42E-41
MAPK signaling pathway	KEGG Pathway	hsa04010	9.08E-39
PI3K-Akt signaling pathway	KEGG Pathway	hsa04151	3.27E-38
TNF signaling pathway	KEGG Pathway	hsa04668	3.27E-38
Lipid and atherosclerosis	KEGG Pathway	hsa05417	2.61E-37
Breast cancer	KEGG Pathway	hsa05224	4.38E-36
AGE-RAGE signaling pathway in diabetic complications	KEGG Pathway	hsa04933	2.63E-34
Human papillomavirus infection	KEGG Pathway	hsa05165	4.61E-34
Gastric cancer	KEGG Pathway	hsa05226	1.99E-33
Prostate cancer	KEGG Pathway	hsa05215	1.22E-31
Signaling pathways regulating pluripotency of stem cells	KEGG Pathway	hsa04550	1.55E-31
Hepatocellular carcinoma	KEGG Pathway	hsa05225	4.25E-31
FoxO signaling pathway	KEGG Pathway	hsa04068	1.97E-30
Chronic myeloid leukemia	KEGG Pathway	hsa05220	3.15E-30
Yersinia infection	KEGG Pathway	hsa05135	6.19E-28
Endocrine resistance	KEGG Pathway	hsa01522	6.20E-28
Focal adhesion	KEGG Pathway	hsa04510	6.20E-28
Cellular senescence	KEGG Pathway	hsa04218	1.72E-27
Small cell lung cancer	KEGG Pathway	hsa05222	4.97E-27
Pancreatic cancer	KEGG Pathway	hsa05212	1.53E-26
Colorectal cancer	KEGG Pathway	hsa05210	3.97E-26
EGFR tyrosine kinase inhibitor resistance	KEGG Pathway	hsa01521	7.84E-26
Human cytomegalovirus infection	KEGG Pathway	hsa05163	1.73E-25
Neurotrophin signaling pathway	KEGG Pathway	hsa04722	4.32E-24
IL-17 signaling pathway	KEGG Pathway	hsa04657	4.77E-24
Hippo signaling pathway	KEGG Pathway	hsa04390	1.79E-23
Kaposi sarcoma-associated herpesvirus infection	KEGG Pathway	hsa05167	2.98E-23
Human T-cell leukemia virus 1 infection	KEGG Pathway	hsa05166	1.45E-22
Osteoclast differentiation	KEGG Pathway	hsa04380	1.86E-22
Non-small cell lung cancer	KEGG Pathway	hsa05223	2.84E-22
Fluid shear stress and atherosclerosis	KEGG Pathway	hsa05418	7.38E-22

PD-L1 expression and PD-1 checkpoint pathway in cancer	KEGG Pathway	hsa05235	1.08E-21
Toll-like receptor signaling pathway	KEGG Pathway	hsa04620	1.37E-21
Renal cell carcinoma	KEGG Pathway	hsa05211	9.09E-21
T cell receptor signaling pathway	KEGG Pathway	hsa04660	9.28E-21
ErbB signaling pathway	KEGG Pathway	hsa04012	9.55E-21
Ras signaling pathway	KEGG Pathway	hsa04014	1.96E-20
Prolactin signaling pathway	KEGG Pathway	hsa04917	2.90E-20
Melanoma	KEGG Pathway	hsa05218	2.98E-20
Measles	KEGG Pathway	hsa05162	8.79E-20
HIF-1 signaling pathway	KEGG Pathway	hsa04066	8.79E-20
Shigellosis	KEGG Pathway	hsa05131	1.35E-19
Toxoplasmosis	KEGG Pathway	hsa05145	1.55E-19
Hepatitis C	KEGG Pathway	hsa05160	1.64E-19
Bladder cancer	KEGG Pathway	hsa05219	2.54E-19
Autophagy - animal	KEGG Pathway	hsa04140	2.98E-19
Chagas disease	KEGG Pathway	hsa05142	3.79E-19
Apoptosis	KEGG Pathway	hsa04210	1.24E-18
Central carbon metabolism in cancer	KEGG Pathway	hsa05230	2.42E-18
MicroRNAs in cancer	KEGG Pathway	hsa05206	2.56E-18
Wnt signaling pathway	KEGG Pathway	hsa04310	2.77E-18
Cell cycle	KEGG Pathway	hsa04110	4.14E-18
Human immunodeficiency virus 1 infection	KEGG Pathway	hsa05170	4.14E-18
Chemical carcinogenesis - receptor activation	KEGG Pathway	hsa05207	4.14E-18
Rap1 signaling pathway	KEGG Pathway	hsa04015	5.57E-18
Glioma	KEGG Pathway	hsa05214	5.65E-18
Pathogenic Escherichia coli infection	KEGG Pathway	hsa05130	5.96E-18
Acute myeloid leukemia	KEGG Pathway	hsa05221	6.51E-18
Salmonella infection	KEGG Pathway	hsa05132	8.57E-18
C-type lectin receptor signaling pathway	KEGG Pathway	hsa04625	9.40E-18
Transcriptional misregulation in cancer	KEGG Pathway	hsa05202	8.47E-17
Relaxin signaling pathway	KEGG Pathway	hsa04926	1.25E-16
Endometrial cancer	KEGG Pathway	hsa05213	1.83E-16
Epstein-Barr virus infection	KEGG Pathway	hsa05169	2.07E-16
NF-kappa B signaling pathway	KEGG Pathway	hsa04064	2.78E-16
Growth hormone synthesis, secretion and action	KEGG Pathway	hsa04935	4.62E-16
Regulation of actin cytoskeleton	KEGG Pathway	hsa04810	5.83E-16

Longevity regulating pathway	KEGG Pathway	hsa04211	5.94E-16
mTOR signaling pathway	KEGG Pathway	hsa04150	8.31E-16
TGF-beta signaling pathway	KEGG Pathway	hsa04350	4.64E-15
Parathyroid hormone synthesis, secretion and action	KEGG Pathway	hsa04928	5.34E-15
Chemokine signaling pathway	KEGG Pathway	hsa04062	1.01E-14
Insulin signaling pathway	KEGG Pathway	hsa04910	1.18E-14
Viral carcinogenesis	KEGG Pathway	hsa05203	1.55E-14
Platinum drug resistance	KEGG Pathway	hsa01524	1.56E-14
AMPK signaling pathway	KEGG Pathway	hsa04152	1.58E-14
Thyroid hormone signaling pathway	KEGG Pathway	hsa04919	2.82E-14
Axon guidance	KEGG Pathway	hsa04360	4.51E-14
Fc epsilon RI signaling pathway	KEGG Pathway	hsa04664	5.85E-14
Insulin resistance	KEGG Pathway	hsa04931	8.17E-14
Sphingolipid signaling pathway	KEGG Pathway	hsa04071	1.52E-13
Th17 cell differentiation	KEGG Pathway	hsa04659	3.56E-13
Cushing syndrome	KEGG Pathway	hsa04934	4.69E-13
Alcoholic liver disease	KEGG Pathway	hsa04936	5.23E-13
p53 signaling pathway	KEGG Pathway	hsa04115	5.52E-13
Adherens junction	KEGG Pathway	hsa04520	6.72E-13
JAK-STAT signaling pathway	KEGG Pathway	hsa04630	3.05E-12
Longevity regulating pathway - multiple species	KEGG Pathway	hsa04213	2.47E-11
Leukocyte transendothelial migration	KEGG Pathway	hsa04670	3.61E-11
Influenza A	KEGG Pathway	hsa05164	3.77E-11
Basal cell carcinoma	KEGG Pathway	hsa05217	5.21E-11
Progesterone-mediated oocyte maturation	KEGG Pathway	hsa04914	5.54E-11
VEGF signaling pathway	KEGG Pathway	hsa04370	7.83E-11
Apelin signaling pathway	KEGG Pathway	hsa04371	1.84E-10
Fc gamma R-mediated phagocytosis	KEGG Pathway	hsa04666	2.25E-10
Tuberculosis	KEGG Pathway	hsa05152	3.48E-10
cAMP signaling pathway	KEGG Pathway	hsa04024	3.57E-10
GnRH signaling pathway	KEGG Pathway	hsa04912	3.95E-10
Tight junction	KEGG Pathway	hsa04530	3.95E-10
Melanogenesis	KEGG Pathway	hsa04916	4.49E-10
NOD-like receptor signaling pathway	KEGG Pathway	hsa04621	4.89E-10
Adipocytokine signaling pathway	KEGG Pathway	hsa04920	6.19E-10
Bacterial invasion of epithelial cells	KEGG Pathway	hsa05100	9.36E-10

B cell receptor signaling pathway	KEGG Pathway	hsa04662	9.36E-10
Apoptosis - multiple species	KEGG Pathway	hsa04215	1.04E-09
Epithelial cell signaling in Helicobacter pylori infection	KEGG Pathway	hsa05120	1.13E-09
Amoebiasis	KEGG Pathway	hsa05146	2.49E-09
Platelet activation	KEGG Pathway	hsa04611	2.96E-09
Th1 and Th2 cell differentiation	KEGG Pathway	hsa04658	3.13E-09
Choline metabolism in cancer	KEGG Pathway	hsa05231	4.49E-09
Pertussis	KEGG Pathway	hsa05133	8.72E-09
Estrogen signaling pathway	KEGG Pathway	hsa04915	8.81E-09
Leishmaniasis	KEGG Pathway	hsa05140	1.48E-08
Thyroid cancer	KEGG Pathway	hsa05216	2.03E-08
Type II diabetes mellitus	KEGG Pathway	hsa04930	2.17E-08
Inflammatory bowel disease	KEGG Pathway	hsa05321	8.52E-08
Phospholipase D signaling pathway	KEGG Pathway	hsa04072	1.02E-07
Mitophagy - animal	KEGG Pathway	hsa04137	2.25E-07
ECM-receptor interaction	KEGG Pathway	hsa04512	2.28E-07
Notch signaling pathway	KEGG Pathway	hsa04330	8.10E-07
Non-alcoholic fatty liver disease	KEGG Pathway	hsa04932	8.62E-07
RIG-I-like receptor signaling pathway	KEGG Pathway	hsa04622	1.07E-06
Alzheimer disease	KEGG Pathway	hsa05010	1.09E-06
Cytokine-cytokine receptor interaction	KEGG Pathway	hsa04060	1.17E-06
Oxytocin signaling pathway	KEGG Pathway	hsa04921	1.49E-06
Hypertrophic cardiomyopathy	KEGG Pathway	hsa05410	1.55E-06
Amphetamine addiction	KEGG Pathway	hsa05031	2.23E-06
Dopaminergic synapse	KEGG Pathway	hsa04728	2.62E-06
Legionellosis	KEGG Pathway	hsa05134	3.93E-06
Rheumatoid arthritis	KEGG Pathway	hsa05323	4.70E-06
Necroptosis	KEGG Pathway	hsa04217	5.58E-06
Gap junction	KEGG Pathway	hsa04540	5.95E-06
Oocyte meiosis	KEGG Pathway	hsa04114	1.09E-05
Adrenergic signaling in cardiomyocytes	KEGG Pathway	hsa04261	1.30E-05
Dilated cardiomyopathy	KEGG Pathway	hsa05414	1.30E-05
Pathways of neurodegeneration - multiple diseases	KEGG Pathway	hsa05022	1.30E-05
Malaria	KEGG Pathway	hsa05144	2.09E-05
Chemical carcinogenesis - reactive oxygen species	KEGG Pathway	hsa05208	2.53E-05
Cholinergic synapse	KEGG Pathway	hsa04725	2.56E-05

Long-term potentiation	KEGG Pathway	hsa04720	2.90E-05
Inflammatory mediator regulation of TRP channels	KEGG Pathway	hsa04750	6.07E-05
Aldosterone-regulated sodium reabsorption	KEGG Pathway	hsa04960	7.45E-05
Autophagy - other	KEGG Pathway	hsa04136	7.45E-05
Natural killer cell mediated cytotoxicity	KEGG Pathway	hsa04650	0.000115
Arrhythmogenic right ventricular cardiomyopathy	KEGG Pathway	hsa05412	0.000135
cGMP-PKG signaling pathway	KEGG Pathway	hsa04022	0.000145
Cocaine addiction	KEGG Pathway	hsa05030	0.000148
GnRH secretion	KEGG Pathway	hsa04929	0.000219
Regulation of lipolysis in adipocytes	KEGG Pathway	hsa04923	0.000265
Long-term depression	KEGG Pathway	hsa04730	0.000402
Diabetic cardiomyopathy	KEGG Pathway	hsa05415	0.000476
Antifolate resistance	KEGG Pathway	hsa01523	0.000696
Viral protein interaction with cytokine and cytokine receptor	KEGG Pathway	hsa04061	0.00115
Ubiquitin mediated proteolysis	KEGG Pathway	hsa04120	0.0012
Calcium signaling pathway	KEGG Pathway	hsa04020	0.00129
African trypanosomiasis	KEGG Pathway	hsa05143	0.00298
Ferroptosis	KEGG Pathway	hsa04216	0.00452
Coronavirus disease - COVID-19	KEGG Pathway	hsa05171	0.00718
Hippo signaling pathway - multiple species	KEGG Pathway	hsa04392	0.0107
Serotonergic synapse	KEGG Pathway	hsa04726	0.012
Glucagon signaling pathway	KEGG Pathway	hsa04922	0.017
Neutrophil extracellular trap formation	KEGG Pathway	hsa04613	0.0173
Cell adhesion molecules	KEGG Pathway	hsa04514	0.0194
Spinocerebellar ataxia	KEGG Pathway	hsa05017	0.022
Maturity onset diabetes of the young	KEGG Pathway	hsa04950	0.0287
Glutamatergic synapse	KEGG Pathway	hsa04724	0.0293
Hematopoietic cell lineage	KEGG Pathway	hsa04640	0.0404
Endocytosis	KEGG Pathway	hsa04144	0.0404
Alcoholism	KEGG Pathway	hsa05034	0.0405
Renin secretion	KEGG Pathway	hsa04924	0.0432
Hedgehog signaling pathway	KEGG Pathway	hsa04340	0.045
Hepatocellular carcinoma	KEGG Disease	H00048	4.18E-10
Gastric cancer	KEGG Disease	H00018	1.46E-07
Breast cancer	KEGG Disease	H00031	1.46E-07
Glioma	KEGG Disease	H00042	3.06E-07

Non-small cell lung cancer	KEGG Disease	H00014	2.30E-06
Ovarian cancer	KEGG Disease	H00027	2.30E-06
Penile cancer	KEGG Disease	H00025	9.38E-06
Medulloblastoma	KEGG Disease	H01667	1.04E-05
Acute myeloid leukemia	KEGG Disease	H00003	1.58E-05
Graft-versus-host disease	KEGG Disease	H00084	5.96E-05
Colorectal cancer	KEGG Disease	H00020	0.000226
Myelodysplastic/myeloproliferative neoplasms	KEGG Disease	H02410	0.000649
Melanoma	KEGG Disease	H00038	0.000649
Thyroid cancer	KEGG Disease	H00032	0.000649
Noonan syndrome and related disorders	KEGG Disease	H00523	0.000649
Esophageal cancer	KEGG Disease	H00017	0.000649
Diffuse large B-cell lymphoma, not otherwise specified	KEGG Disease	H02434	0.000794
Atypical chronic myeloid leukemia	KEGG Disease	H02412	0.00148
Allograft rejection	KEGG Disease	H00083	0.00148
Meningioma	KEGG Disease	H01556	0.00148
Chronic myelomonocytic leukemia	KEGG Disease	H02411	0.00341
Prostate cancer	KEGG Disease	H00024	0.00358
Myelodysplastic syndrome	KEGG Disease	H01481	0.00358
Inflammatory bowel disease (IBD)	KEGG Disease	H01227	0.00377
Osteoporosis	KEGG Disease	H01593	0.00897
Pancreatic cancer	KEGG Disease	H00019	0.00993
Long QT syndrome	KEGG Disease	H00720	0.0142
Noonan syndrome	KEGG Disease	H01738	0.0212
Pituitary adenomas	KEGG Disease	H01102	0.0247
Lung cancer	TTD Disease	ICD-11[2C25]	7.52E-09
Brain cancer	TTD Disease	ICD-11[2A00]	2.53E-06
Malignant haematopoietic neoplasm	TTD Disease	ICD-11[2B33]	4.57E-06
Breast cancer	TTD Disease	ICD-11[2C60-2C6Y]	1.35E-05
Colorectal cancer	TTD Disease	ICD-11[2B91]	7.36E-05
Rheumatoid arthritis	TTD Disease	ICD-11[FA20]	7.39E-05
Ovarian cancer	TTD Disease	ICD-11[2C73]	0.000101
Multiple myeloma	TTD Disease	ICD-11[2A83]	0.000216
Nasopharyngeal cancer	TTD Disease	ICD-11[2B6B]	0.000328
Melanoma	TTD Disease	ICD-11[2C30]	0.00107
Cutaneous lupus erythematosus	TTD Disease	ICD-11[EB50-EB5Z]	0.00124

Myeloproliferative neoplasm	TTD Disease	ICD-11[2A20]	0.0016
Low bone mass disorder	TTD Disease	ICD-11[FB83]	0.00172
Liver cancer	TTD Disease	ICD-11[2C12]	0.00182
Prostate cancer	TTD Disease	ICD-11[2C82]	0.00443
Pancreatic cancer	TTD Disease	ICD-11[2C10]	0.00443
Transplant rejection	TTD Disease	ICD-11[NE84]	0.00443
Renal cell carcinoma	TTD Disease	ICD-11[2C90]	0.00525
Metastatic tumour	TTD Disease	ICD-11[2D50-2E2Z]	0.00773
Metastatic lymph node neoplasm	TTD Disease	ICD-11[2D60]	0.00773
Thrombocytopenia	TTD Disease	ICD-11[3B64]	0.00798
Myelodysplastic syndrome	TTD Disease	ICD-11[2A37]	0.01
Adrenal cancer	TTD Disease	ICD-11[2D11]	0.0133
Retinopathy	TTD Disease	ICD-11[9B71]	0.0188
Idiopathic interstitial pneumonitis	TTD Disease	ICD-11[CB03]	0.0205
Systemic sclerosis	TTD Disease	ICD-11[4A42]	0.0232
Lymphoma	TTD Disease	ICD-11[2A80-2A86]	0.0238
Mature B-cell leukaemia	TTD Disease	ICD-11[2A82]	0.0243
Colon cancer	TTD Disease	ICD-11[2B90]	0.0283
Lupus erythematosus	TTD Disease	ICD-11[4A40]	0.0307
Diffuse large B-cell lymphoma	TTD Disease	ICD-11[2A81]	0.0404
B-cell lymphoma	TTD Disease	ICD-11[2A86]	0.0417
Immune system disease	TTD Disease	ICD-11[4A01-4B41]	0.0437
Diabetic foot ulcer	TTD Disease	ICD-11[BD54]	0.0437
Urinary system disease	TTD Disease	ICD-11[GC2Z]	0.0471
gland development	Biological Process	GO:0048732	7.83E-66
regulation of apoptotic signaling pathway	Biological Process	GO:2001233	1.85E-53
epithelial cell proliferation	Biological Process	GO:0050673	5.83E-53
neuron death	Biological Process	GO:0070997	2.62E-52
regulation of DNA-binding transcription factor activity	Biological Process	GO:0051090	7.88E-51
epithelial tube morphogenesis	Biological Process	GO:0060562	9.60E-51
urogenital system development	Biological Process	GO:0001655	9.80E-49
ossification	Biological Process	GO:0001503	9.80E-49
positive regulation of protein serine/threonine kinase activity	Biological Process	GO:0071902	1.39E-48
peptidyl-serine phosphorylation	Biological Process	GO:0018105	1.82E-48
peptidyl-serine modification	Biological Process	GO:0018209	6.64E-48
reproductive system development	Biological Process	GO:0061458	2.15E-47

reproductive structure development	Biological Process	GO:0048608	2.24E-47
positive regulation of cell adhesion	Biological Process	GO:0045785	3.43E-47
response to oxygen levels	Biological Process	GO:0070482	4.25E-47
regulation of neuron death	Biological Process	GO:1901214	1.53E-46
response to steroid hormone	Biological Process	GO:0048545	4.39E-46
regulation of MAP kinase activity	Biological Process	GO:0043405	6.51E-46
cellular response to chemical stress	Biological Process	GO:0062197	8.76E-46
regulation of binding	Biological Process	GO:0051098	1.45E-45
regulation of epithelial cell proliferation	Biological Process	GO:0050678	3.46E-45
response to oxidative stress	Biological Process	GO:0006979	6.09E-45
regulation of cell-cell adhesion	Biological Process	GO:0022407	3.11E-44
gliogenesis	Biological Process	GO:0042063	3.42E-44
response to antibiotic	Biological Process	GO:0046677	4.78E-44
response to decreased oxygen levels	Biological Process	GO:0036293	1.45E-43
myeloid cell differentiation	Biological Process	GO:0030099	1.85E-43
morphogenesis of a branching structure	Biological Process	GO:0001763	8.57E-43
aging	Biological Process	GO:0007568	3.48E-41
extrinsic apoptotic signaling pathway	Biological Process	GO:0097191	6.79E-41
negative regulation of phosphorylation	Biological Process	GO:0042326	9.75E-41
neuron apoptotic process	Biological Process	GO:0051402	1.05E-40
activation of protein kinase activity	Biological Process	GO:0032147	1.16E-40
cellular response to oxidative stress	Biological Process	GO:0034599	1.23E-40
ameboidal-type cell migration	Biological Process	GO:0001667	1.23E-40
muscle tissue development	Biological Process	GO:0060537	1.24E-40
renal system development	Biological Process	GO:0072001	1.28E-40
response to molecule of bacterial origin	Biological Process	GO:0002237	1.28E-40
negative regulation of protein phosphorylation	Biological Process	GO:0001933	1.68E-40
response to transforming growth factor beta	Biological Process	GO:0071559	2.10E-40
ERK1 and ERK2 cascade	Biological Process	GO:0070371	4.03E-40
negative regulation of apoptotic signaling pathway	Biological Process	GO:2001234	4.03E-40
response to lipopolysaccharide	Biological Process	GO:0032496	4.79E-40
response to hypoxia	Biological Process	GO:0001666	5.12E-40
osteoblast differentiation	Biological Process	GO:0001649	6.00E-40
T cell activation	Biological Process	GO:0042110	8.29E-40
cellular response to transforming growth factor beta stimulus	Biological Process	GO:0071560	1.00E-39
regulation of hemopoiesis	Biological Process	GO:1903706	1.01E-39

morphogenesis of a branching epithelium	Biological Process	GO:0061138	1.25E-39
stress-activated protein kinase signaling cascade	Biological Process	GO:0031098	3.04E-39
striated muscle tissue development	Biological Process	GO:0014706	3.90E-39
stress-activated MAPK cascade	Biological Process	GO:0051403	8.84E-39
positive regulation of neurogenesis	Biological Process	GO:0050769	9.27E-39
response to nutrient levels	Biological Process	GO:0031667	1.07E-38
embryonic organ development	Biological Process	GO:0048568	2.25E-38
cellular response to external stimulus	Biological Process	GO:0071496	3.05E-38
regulation of vasculature development	Biological Process	GO:1901342	3.05E-38
cellular response to peptide	Biological Process	GO:1901653	3.07E-38
kidney development	Biological Process	GO:0001822	8.07E-38
cell growth	Biological Process	GO:0016049	9.59E-38
positive regulation of MAP kinase activity	Biological Process	GO:0043406	1.61E-37
cell fate commitment	Biological Process	GO:0045165	2.56E-37
cellular response to drug	Biological Process	GO:0035690	5.86E-37
response to acid chemical	Biological Process	GO:0001101	8.06E-37
regulation of ERK1 and ERK2 cascade	Biological Process	GO:0070372	2.10E-36
positive regulation of cell-cell adhesion	Biological Process	GO:0022409	4.66E-36
positive regulation of catabolic process	Biological Process	GO:0009896	5.48E-36
muscle cell proliferation	Biological Process	GO:0033002	1.49E-35
positive regulation of DNA-binding transcription factor activity	Biological Process	GO:0051091	2.27E-35
tissue migration	Biological Process	GO:0090130	2.74E-35
epithelium migration	Biological Process	GO:0090132	3.62E-35
positive regulation of cell cycle	Biological Process	GO:0045787	4.01E-35
positive regulation of cellular catabolic process	Biological Process	GO:0031331	4.01E-35
epithelial cell migration	Biological Process	GO:0010631	4.01E-35
transmembrane receptor protein serine/threonine kinase signaling pathway	Biological Process	GO:0007178	4.55E-35
regulation of neuron apoptotic process	Biological Process	GO:0043523	1.08E-34
response to reactive oxygen species	Biological Process	GO:0000302	2.38E-34
peptidyl-tyrosine phosphorylation	Biological Process	GO:0018108	2.43E-34
response to peptide hormone	Biological Process	GO:0043434	3.16E-34
cellular response to steroid hormone stimulus	Biological Process	GO:0071383	3.73E-34
glial cell differentiation	Biological Process	GO:0010001	3.79E-34
peptidyl-tyrosine modification	Biological Process	GO:0018212	8.16E-34
lymphocyte differentiation	Biological Process	GO:0030098	9.90E-34
branching morphogenesis of an epithelial tube	Biological Process	GO:0048754	1.60E-33

regulation of ossification	Biological Process	GO:0030278	1.76E-33
muscle organ development	Biological Process	GO:0007517	2.34E-33
positive regulation of cytokine production	Biological Process	GO:0001819	3.25E-33
regulation of leukocyte differentiation	Biological Process	GO:1902105	5.56E-33
intrinsic apoptotic signaling pathway	Biological Process	GO:0097193	9.18E-33
regulation of angiogenesis	Biological Process	GO:0045765	1.15E-32
regulation of developmental growth	Biological Process	GO:0048638	2.12E-32
leukocyte cell-cell adhesion	Biological Process	GO:0007159	2.18E-32
axonogenesis	Biological Process	GO:0007409	4.26E-32
regulation of extrinsic apoptotic signaling pathway	Biological Process	GO:2001236	6.72E-32
regulation of cell growth	Biological Process	GO:0001558	7.49E-32
cell-substrate adhesion	Biological Process	GO:0031589	8.93E-32
regulation of animal organ morphogenesis	Biological Process	GO:2000027	1.17E-31
kidney epithelium development	Biological Process	GO:0072073	1.48E-31
histone modification	Biological Process	GO:0016570	1.62E-31
connective tissue development	Biological Process	GO:0061448	1.84E-31
forebrain development	Biological Process	GO:0030900	2.68E-31
regulation of cysteine-type endopeptidase activity involved in apoptotic process	Biological Process	GO:0043281	3.26E-31
canonical Wnt signaling pathway	Biological Process	GO:0060070	5.92E-31
G1/S transition of mitotic cell cycle	Biological Process	GO:0000082	6.67E-31
regulation of Wnt signaling pathway	Biological Process	GO:0030111	7.61E-31
covalent chromatin modification	Biological Process	GO:0016569	9.56E-31
regulation of leukocyte cell-cell adhesion	Biological Process	GO:1903037	1.66E-30
positive regulation of epithelial cell proliferation	Biological Process	GO:0050679	1.75E-30
cell cycle G1/S phase transition	Biological Process	GO:0044843	2.14E-30
rhythmic process	Biological Process	GO:0048511	2.42E-30
protein kinase B signaling	Biological Process	GO:0043491	2.48E-30
regulation of myeloid cell differentiation	Biological Process	GO:0045637	4.37E-30
extracellular matrix organization	Biological Process	GO:0030198	4.92E-30
transforming growth factor beta receptor signaling pathway	Biological Process	GO:0007179	5.20E-30
cellular response to biotic stimulus	Biological Process	GO:0071216	6.20E-30
extracellular structure organization	Biological Process	GO:0043062	7.01E-30
regulation of epithelial cell migration	Biological Process	GO:0010632	7.25E-30
heart morphogenesis	Biological Process	GO:0003007	9.18E-30
mesenchyme development	Biological Process	GO:0060485	1.19E-29
positive regulation of proteolysis	Biological Process	GO:0045862	2.07E-29

regulation of neuron projection development	Biological Process	GO:0010975	2.20E-29
regulation of cysteine-type endopeptidase activity	Biological Process	GO:2000116	2.70E-29
positive regulation of binding	Biological Process	GO:0051099	4.22E-29
regulation of cellular response to growth factor stimulus	Biological Process	GO:0090287	4.58E-29
positive regulation of epithelial cell migration	Biological Process	GO:0010634	6.47E-29
negative regulation of extrinsic apoptotic signaling pathway	Biological Process	GO:2001237	7.23E-29
response to mechanical stimulus	Biological Process	GO:0009612	7.70E-29
respiratory system development	Biological Process	GO:0060541	8.28E-29
regulation of protein binding	Biological Process	GO:0043393	1.20E-28
regulation of stress-activated protein kinase signaling cascade	Biological Process	GO:0070302	1.23E-28
regulation of muscle tissue development	Biological Process	GO:1901861	1.27E-28
regulation of stress-activated MAPK cascade	Biological Process	GO:0032872	2.17E-28
positive regulation of hemopoiesis	Biological Process	GO:1903708	2.48E-28
regulation of peptide secretion	Biological Process	GO:0002791	2.68E-28
cellular response to reactive oxygen species	Biological Process	GO:0034614	3.50E-28
regulation of protein secretion	Biological Process	GO:0050708	4.79E-28
intracellular receptor signaling pathway	Biological Process	GO:0030522	5.50E-28
JNK cascade	Biological Process	GO:0007254	6.06E-28
lung development	Biological Process	GO:0030324	6.73E-28
regulation of striated muscle tissue development	Biological Process	GO:0016202	7.05E-28
positive regulation of vasculature development	Biological Process	GO:1904018	7.05E-28
homeostasis of number of cells	Biological Process	GO:0048872	7.17E-28
positive regulation of leukocyte cell-cell adhesion	Biological Process	GO:1903039	9.29E-28
positive regulation of ERK1 and ERK2 cascade	Biological Process	GO:0070374	9.81E-28
respiratory tube development	Biological Process	GO:0030323	1.23E-27
regulation of T cell activation	Biological Process	GO:0050863	1.37E-27
regulation of muscle organ development	Biological Process	GO:0048634	1.47E-27
regulation of cell morphogenesis	Biological Process	GO:0022604	1.76E-27
muscle cell differentiation	Biological Process	GO:0042692	1.87E-27
myeloid leukocyte differentiation	Biological Process	GO:0002573	1.95E-27
leukocyte proliferation	Biological Process	GO:0070661	1.98E-27
positive regulation of cell cycle process	Biological Process	GO:0090068	1.98E-27
cardiac muscle tissue development	Biological Process	GO:0048738	2.85E-27
regulation of smooth muscle cell proliferation	Biological Process	GO:0048660	3.50E-27
response to metal ion	Biological Process	GO:0010038	3.60E-27
T cell differentiation	Biological Process	GO:0030217	3.72E-27

cell cycle arrest	Biological Process	GO:0007050	4.09E-27
cellular response to molecule of bacterial origin	Biological Process	GO:0071219	4.79E-27
pri-miRNA transcription by RNA polymerase II	Biological Process	GO:0061614	4.81E-27
cellular response to peptide hormone stimulus	Biological Process	GO:0071375	5.09E-27
positive regulation of protein transport	Biological Process	GO:0051222	6.51E-27
smooth muscle cell proliferation	Biological Process	GO:0048659	1.12E-26
cellular response to abiotic stimulus	Biological Process	GO:0071214	1.60E-26
cellular response to environmental stimulus	Biological Process	GO:0104004	1.60E-26
regulation of protein kinase B signaling	Biological Process	GO:0051896	2.28E-26
positive regulation of establishment of protein localization	Biological Process	GO:1904951	2.83E-26
positive regulation of protein kinase B signaling	Biological Process	GO:0051897	3.56E-26
negative regulation of cell development	Biological Process	GO:0010721	3.87E-26
positive regulation of angiogenesis	Biological Process	GO:0045766	4.55E-26
mesenchymal cell differentiation	Biological Process	GO:0048762	5.11E-26
autophagy	Biological Process	GO:0006914	5.23E-26
process utilizing autophagic mechanism	Biological Process	GO:0061919	5.23E-26
gland morphogenesis	Biological Process	GO:0022612	6.27E-26
negative regulation of neuron death	Biological Process	GO:1901215	6.90E-26
mammary gland development	Biological Process	GO:0030879	1.19E-25
regionalization	Biological Process	GO:0003002	1.28E-25
negative regulation of transferase activity	Biological Process	GO:0051348	1.39E-25
regulation of lymphocyte activation	Biological Process	GO:0051249	1.72E-25
regulation of lipid metabolic process	Biological Process	GO:0019216	1.72E-25
gastrulation	Biological Process	GO:0007369	1.72E-25
negative regulation of cell adhesion	Biological Process	GO:0007162	1.73E-25
mesonephric epithelium development	Biological Process	GO:0072163	1.94E-25
mesonephric tubule development	Biological Process	GO:0072164	1.94E-25
multicellular organismal homeostasis	Biological Process	GO:0048871	2.22E-25
regeneration	Biological Process	GO:0031099	2.36E-25
cellular response to lipopolysaccharide	Biological Process	GO:0071222	3.41E-25
pattern specification process	Biological Process	GO:0007389	3.58E-25
nephron development	Biological Process	GO:0072006	3.73E-25
eye development	Biological Process	GO:0001654	4.51E-25
regulation of protein catabolic process	Biological Process	GO:0042176	6.00E-25
regulation of osteoblast differentiation	Biological Process	GO:0045667	6.47E-25
mesonephros development	Biological Process	GO:0001823	6.54E-25

ureteric bud development	Biological Process	GO:0001657	7.16E-25
positive regulation of neuron differentiation	Biological Process	GO:0045666	7.93E-25
sensory system development	Biological Process	GO:0048880	7.93E-25
steroid hormone mediated signaling pathway	Biological Process	GO:0043401	8.65E-25
organ growth	Biological Process	GO:0035265	9.47E-25
regulation of DNA binding	Biological Process	GO:0051101	1.07E-24
endocrine system development	Biological Process	GO:0035270	1.26E-24
reactive oxygen species metabolic process	Biological Process	GO:0072593	1.28E-24
mononuclear cell proliferation	Biological Process	GO:0032943	1.41E-24
visual system development	Biological Process	GO:0150063	1.63E-24
positive regulation of T cell activation	Biological Process	GO:0050870	1.64E-24
positive regulation of secretion by cell	Biological Process	GO:1903532	1.79E-24
cellular response to extracellular stimulus	Biological Process	GO:0031668	1.91E-24
cellular response to acid chemical	Biological Process	GO:0071229	2.21E-24
lymphocyte proliferation	Biological Process	GO:0046651	2.41E-24
negative regulation of growth	Biological Process	GO:0045926	3.10E-24
regulation of peptidyl-tyrosine phosphorylation	Biological Process	GO:0050730	3.50E-24
positive regulation of neuron death	Biological Process	GO:1901216	4.10E-24
positive regulation of NF-kappaB transcription factor activity	Biological Process	GO:0051092	4.50E-24
regulation of reactive oxygen species metabolic process	Biological Process	GO:2000377	5.40E-24
embryonic organ morphogenesis	Biological Process	GO:0048562	5.72E-24
liver development	Biological Process	GO:0001889	5.89E-24
hepaticobiliary system development	Biological Process	GO:0061008	6.43E-24
cellular response to antibiotic	Biological Process	GO:0071236	7.76E-24
cell-matrix adhesion	Biological Process	GO:0007160	8.78E-24
regulation of autophagy	Biological Process	GO:0010506	9.03E-24
cartilage development	Biological Process	GO:0051216	9.68E-24
regulation of canonical Wnt signaling pathway	Biological Process	GO:0060828	9.73E-24
sex differentiation	Biological Process	GO:0007548	1.57E-23
endothelial cell migration	Biological Process	GO:0043542	1.95E-23
response to hydrogen peroxide	Biological Process	GO:0042542	2.44E-23
positive regulation of secretion	Biological Process	GO:0051047	3.40E-23
cardiac chamber development	Biological Process	GO:0003205	4.12E-23
positive regulation of leukocyte differentiation	Biological Process	GO:1902107	4.25E-23
Ras protein signal transduction	Biological Process	GO:0007265	4.43E-23
morphogenesis of embryonic epithelium	Biological Process	GO:0016331	4.46E-23

skeletal system morphogenesis	Biological Process	GO:0048705	4.93E-23
leukocyte migration	Biological Process	GO:0050900	5.36E-23
positive regulation of growth	Biological Process	GO:0045927	5.79E-23
negative regulation of nervous system development	Biological Process	GO:0051961	6.63E-23
negative regulation of neurogenesis	Biological Process	GO:0050768	7.26E-23
regulation of pri-miRNA transcription by RNA polymerase II	Biological Process	GO:1902893	7.34E-23
negative regulation of kinase activity	Biological Process	GO:0033673	7.56E-23
tissue remodeling	Biological Process	GO:0048771	1.01E-22
regulation of transmembrane receptor protein serine/threonine kinase signaling pathway	Biological Process	GO:0090092	1.10E-22
regulation of cell-substrate adhesion	Biological Process	GO:0010810	1.42E-22
cell chemotaxis	Biological Process	GO:0060326	1.42E-22
in utero embryonic development	Biological Process	GO:0001701	1.42E-22
negative regulation of protein kinase activity	Biological Process	GO:0006469	1.47E-22
positive regulation of animal organ morphogenesis	Biological Process	GO:0110110	2.20E-22
myeloid cell homeostasis	Biological Process	GO:0002262	2.36E-22
response to alcohol	Biological Process	GO:0097305	2.56E-22
fat cell differentiation	Biological Process	GO:0045444	2.56E-22
cell junction assembly	Biological Process	GO:0034329	2.78E-22
positive regulation of cell activation	Biological Process	GO:0050867	2.84E-22
bone development	Biological Process	GO:0060348	3.32E-22
cellular response to oxygen levels	Biological Process	GO:0071453	3.83E-22
regulation of JNK cascade	Biological Process	GO:0046328	3.97E-22
tube formation	Biological Process	GO:0035148	4.08E-22
response to insulin	Biological Process	GO:0032868	4.32E-22
regulation of actin filament-based process	Biological Process	GO:0032970	4.36E-22
positive regulation of apoptotic signaling pathway	Biological Process	GO:2001235	4.60E-22
I-kappaB kinase/NF-kappaB signaling	Biological Process	GO:0007249	5.12E-22
protein autophosphorylation	Biological Process	GO:0046777	5.65E-22
response to nutrient	Biological Process	GO:0007584	7.68E-22
response to ketone	Biological Process	GO:1901654	8.31E-22
signal transduction by p53 class mediator	Biological Process	GO:0072331	8.84E-22
response to radiation	Biological Process	GO:0009314	9.58E-22
regulation of peptidase activity	Biological Process	GO:0052547	9.88E-22
positive regulation of smooth muscle cell proliferation	Biological Process	GO:0048661	1.02E-21
cardiac septum development	Biological Process	GO:0003279	1.06E-21
inositol lipid-mediated signaling	Biological Process	GO:0048017	1.09E-21

positive regulation of leukocyte activation	Biological Process	GO:0002696	1.11E-21
regulation of chemotaxis	Biological Process	GO:0050920	1.32E-21
axon guidance	Biological Process	GO:0007411	1.81E-21
camera-type eye development	Biological Process	GO:0043010	1.89E-21
regulation of peptidyl-serine phosphorylation	Biological Process	GO:0033135	2.09E-21
regulation of protein stability	Biological Process	GO:0031647	2.52E-21
neuron projection guidance	Biological Process	GO:0097485	2.57E-21
positive regulation of cell projection organization	Biological Process	GO:0031346	2.65E-21
activation of MAPK activity	Biological Process	GO:0000187	3.53E-21
stem cell differentiation	Biological Process	GO:0048863	3.67E-21
regulation of myeloid leukocyte differentiation	Biological Process	GO:0002761	4.49E-21
phosphatidylinositol-mediated signaling	Biological Process	GO:0048015	5.36E-21
positive regulation of ossification	Biological Process	GO:0045778	5.78E-21
regulation of cell morphogenesis involved in differentiation	Biological Process	GO:0010769	6.32E-21
regulation of endopeptidase activity	Biological Process	GO:0052548	7.75E-21
positive regulation of mitotic cell cycle	Biological Process	GO:0045931	8.61E-21
telencephalon development	Biological Process	GO:0021537	8.97E-21
formation of primary germ layer	Biological Process	GO:0001704	9.16E-21
positive regulation of peptidyl-tyrosine phosphorylation	Biological Process	GO:0050731	9.65E-21
regulation of mononuclear cell proliferation	Biological Process	GO:0032944	1.21E-20
cellular response to insulin stimulus	Biological Process	GO:0032869	1.38E-20
astrocyte differentiation	Biological Process	GO:0048708	1.74E-20
cellular response to nutrient levels	Biological Process	GO:0031669	1.76E-20
positive regulation of lymphocyte activation	Biological Process	GO:0051251	2.11E-20
positive regulation of cellular protein localization	Biological Process	GO:1903829	2.95E-20
regulation of lymphocyte proliferation	Biological Process	GO:0050670	3.22E-20
epithelial to mesenchymal transition	Biological Process	GO:0001837	3.38E-20
epithelial tube formation	Biological Process	GO:0072175	3.40E-20
negative regulation of immune system process	Biological Process	GO:0002683	3.83E-20
nephron epithelium development	Biological Process	GO:0072009	3.85E-20
regulation of leukocyte proliferation	Biological Process	GO:0070663	3.93E-20
anterior/posterior pattern specification	Biological Process	GO:0009952	4.59E-20
striated muscle cell differentiation	Biological Process	GO:0051146	5.23E-20
hormone-mediated signaling pathway	Biological Process	GO:0009755	5.23E-20
phosphatidylinositol 3-kinase signaling	Biological Process	GO:0014065	5.35E-20
signal transduction in response to DNA damage	Biological Process	GO:0042770	5.91E-20

regulation of I-kappaB kinase/NF-kappaB signaling	Biological Process	GO:0043122	6.42E-20
cellular response to decreased oxygen levels	Biological Process	GO:0036294	7.94E-20
regulation of mitotic cell cycle phase transition	Biological Process	GO:1901990	1.39E-19
epithelial cell apoptotic process	Biological Process	GO:1904019	1.40E-19
negative regulation of neuron apoptotic process	Biological Process	GO:0043524	1.58E-19
regulation of cardiac muscle tissue development	Biological Process	GO:0055024	1.67E-19
positive regulation of chemotaxis	Biological Process	GO:0050921	1.80E-19
animal organ formation	Biological Process	GO:0048645	2.28E-19
regulation of chromosome organization	Biological Process	GO:0033044	2.28E-19
cellular response to mechanical stimulus	Biological Process	GO:0071260	2.35E-19
renal tubule development	Biological Process	GO:0061326	2.38E-19
glandular epithelial cell differentiation	Biological Process	GO:0002067	2.40E-19
regulation of morphogenesis of an epithelium	Biological Process	GO:1905330	2.74E-19
embryonic epithelial tube formation	Biological Process	GO:0001838	3.09E-19
regulation of gliogenesis	Biological Process	GO:0014013	3.09E-19
cardiac ventricle development	Biological Process	GO:0003231	3.12E-19
regulation of protein localization to membrane	Biological Process	GO:1905475	3.28E-19
developmental growth involved in morphogenesis	Biological Process	GO:0060560	3.98E-19
placenta development	Biological Process	GO:0001890	4.01E-19
positive regulation of pri-miRNA transcription by RNA polymerase II	Biological Process	GO:1902895	4.72E-19
cell aging	Biological Process	GO:0007569	5.00E-19
positive regulation of endothelial cell migration	Biological Process	GO:0010595	5.43E-19
regulation of endothelial cell migration	Biological Process	GO:0010594	5.62E-19
hormone secretion	Biological Process	GO:0046879	5.87E-19
positive regulation of protein secretion	Biological Process	GO:0050714	5.95E-19
response to tumor necrosis factor	Biological Process	GO:0034612	6.41E-19
endothelium development	Biological Process	GO:0003158	9.26E-19
stem cell proliferation	Biological Process	GO:0072089	9.26E-19
positive regulation of peptide secretion	Biological Process	GO:0002793	1.11E-18
cardiocyte differentiation	Biological Process	GO:0035051	1.16E-18
epithelial cell development	Biological Process	GO:0002064	1.23E-18
positive regulation of protein catabolic process	Biological Process	GO:0045732	1.30E-18
heart valve morphogenesis	Biological Process	GO:0003179	1.83E-18
positive regulation of phosphatidylinositol 3-kinase signaling	Biological Process	GO:0014068	1.86E-18
outflow tract morphogenesis	Biological Process	GO:0003151	1.86E-18
response to purine-containing compound	Biological Process	GO:0014074	1.99E-18

cellular response to toxic substance	Biological Process	GO:0097237	2.31E-18
muscle system process	Biological Process	GO:0003012	2.46E-18
maintenance of cell number	Biological Process	GO:0098727	2.46E-18
nephron tubule development	Biological Process	GO:0072080	2.67E-18
regulation of organ growth	Biological Process	GO:0046620	2.69E-18
regulation of inflammatory response	Biological Process	GO:0050727	2.82E-18
hormone transport	Biological Process	GO:0009914	2.82E-18
development of primary sexual characteristics	Biological Process	GO:0045137	2.82E-18
regulation of response to wounding	Biological Process	GO:1903034	2.97E-18
signal transduction in absence of ligand	Biological Process	GO:0038034	3.45E-18
extrinsic apoptotic signaling pathway in absence of ligand	Biological Process	GO:0097192	3.45E-18
regulation of cyclin-dependent protein serine/threonine kinase activity	Biological Process	GO:0000079	3.81E-18
neural tube development	Biological Process	GO:0021915	3.84E-18
gonad development	Biological Process	GO:0008406	3.96E-18
positive regulation of peptidyl-serine phosphorylation	Biological Process	GO:0033138	4.10E-18
response to interleukin-1	Biological Process	GO:0070555	4.49E-18
positive regulation of cysteine-type endopeptidase activity involved in apoptotic process	Biological Process	GO:0043280	4.52E-18
cardiac chamber morphogenesis	Biological Process	GO:0003206	4.67E-18
positive regulation of peptidase activity	Biological Process	GO:0010952	4.88E-18
regulation of cell cycle phase transition	Biological Process	GO:1901987	5.93E-18
regulation of actin cytoskeleton organization	Biological Process	GO:0032956	7.19E-18
regulation of cell division	Biological Process	GO:0051302	7.46E-18
blood coagulation	Biological Process	GO:0007596	8.52E-18
response to corticosteroid	Biological Process	GO:0031960	9.43E-18
digestive system development	Biological Process	GO:0055123	9.64E-18
response to carbohydrate	Biological Process	GO:0009743	9.79E-18
positive regulation of stress-activated MAPK cascade	Biological Process	GO:0032874	1.01E-17
cardiac septum morphogenesis	Biological Process	GO:0060411	1.06E-17
positive regulation of neuron projection development	Biological Process	GO:0010976	1.17E-17
hemostasis	Biological Process	GO:0007599	1.17E-17
alpha-beta T cell differentiation	Biological Process	GO:0046632	1.20E-17
negative regulation of cytokine production	Biological Process	GO:0001818	1.24E-17
digestive tract development	Biological Process	GO:0048565	1.25E-17
leukocyte apoptotic process	Biological Process	GO:0071887	1.28E-17
heart valve development	Biological Process	GO:0003170	1.29E-17
positive regulation of defense response	Biological Process	GO:0031349	1.32E-17

DNA damage response, signal transduction by p53 class mediator	Biological Process	GO:0030330	1.35E-17
sensory organ morphogenesis	Biological Process	GO:0090596	1.35E-17
temperature homeostasis	Biological Process	GO:0001659	1.51E-17
positive regulation of stress-activated protein kinase signaling cascade	Biological Process	GO:0070304	1.51E-17
coagulation	Biological Process	GO:0050817	1.51E-17
response to monosaccharide	Biological Process	GO:0034284	1.60E-17
response to UV	Biological Process	GO:0009411	1.77E-17
stem cell population maintenance	Biological Process	GO:0019827	1.78E-17
positive regulation of DNA binding	Biological Process	GO:0043388	1.92E-17
negative regulation of neuron differentiation	Biological Process	GO:0045665	1.95E-17
positive regulation of osteoblast differentiation	Biological Process	GO:0045669	2.05E-17
T cell proliferation	Biological Process	GO:0042098	2.24E-17
circadian rhythm	Biological Process	GO:0007623	2.30E-17
multi-multicellular organism process	Biological Process	GO:0044706	2.33E-17
mammary gland epithelium development	Biological Process	GO:0061180	2.73E-17
positive regulation of myeloid cell differentiation	Biological Process	GO:0045639	2.80E-17
osteoclast differentiation	Biological Process	GO:0030316	3.47E-17
response to cAMP	Biological Process	GO:0051591	3.47E-17
artery development	Biological Process	GO:0060840	3.74E-17
positive regulation of cysteine-type endopeptidase activity	Biological Process	GO:2001056	3.78E-17
maintenance of location	Biological Process	GO:0051235	3.84E-17
negative regulation of cell cycle process	Biological Process	GO:0010948	3.84E-17
regulation of phosphatidylinositol 3-kinase signaling	Biological Process	GO:0014066	4.03E-17
regulation of cyclin-dependent protein kinase activity	Biological Process	GO:1904029	4.10E-17
columnar/cuboidal epithelial cell differentiation	Biological Process	GO:0002065	4.27E-17
regulation of histone modification	Biological Process	GO:0031056	4.50E-17
regulation of protein modification by small protein conjugation or removal	Biological Process	GO:1903320	4.50E-17
renal tubule morphogenesis	Biological Process	GO:0061333	4.60E-17
nephron morphogenesis	Biological Process	GO:0072028	4.60E-17
regulation of epithelial cell differentiation	Biological Process	GO:0030856	4.72E-17
regulation of protein-containing complex assembly	Biological Process	GO:0043254	4.77E-17
cellular response to hypoxia	Biological Process	GO:0071456	5.59E-17
peptidyl-threonine modification	Biological Process	GO:0018210	5.61E-17
response to organophosphorus	Biological Process	GO:0046683	5.61E-17
positive regulation of I-kappaB kinase/NF-kappaB signaling	Biological Process	GO:0043123	5.61E-17
regulation of cell-matrix adhesion	Biological Process	GO:0001952	7.16E-17

regulation of extrinsic apoptotic signaling pathway via death domain receptors	Biological Process	GO:1902041	7.27E-17
cellular response to tumor necrosis factor	Biological Process	GO:0071356	7.33E-17
anatomical structure homeostasis	Biological Process	GO:0060249	7.46E-17
regulation of cellular protein catabolic process	Biological Process	GO:1903362	7.67E-17
regulation of G1/S transition of mitotic cell cycle	Biological Process	GO:2000045	8.30E-17
regulation of fat cell differentiation	Biological Process	GO:0045598	9.79E-17
regulation of embryonic development	Biological Process	GO:0045995	9.79E-17
vascular endothelial growth factor receptor signaling pathway	Biological Process	GO:0048010	1.08E-16
peptidyl-threonine phosphorylation	Biological Process	GO:0018107	1.10E-16
regulation of cytokine biosynthetic process	Biological Process	GO:0042035	1.27E-16
female pregnancy	Biological Process	GO:0007565	1.30E-16
appendage development	Biological Process	GO:0048736	1.68E-16
limb development	Biological Process	GO:0060173	1.68E-16
regulation of lymphocyte differentiation	Biological Process	GO:0045619	1.75E-16
positive regulation of lipid metabolic process	Biological Process	GO:0045834	1.77E-16
positive regulation of JNK cascade	Biological Process	GO:0046330	2.17E-16
negative regulation of response to external stimulus	Biological Process	GO:0032102	2.43E-16
blood vessel endothelial cell migration	Biological Process	GO:0043534	2.49E-16
response to estradiol	Biological Process	GO:0032355	2.58E-16
extrinsic apoptotic signaling pathway via death domain receptors	Biological Process	GO:0008625	2.61E-16
regulation of supramolecular fiber organization	Biological Process	GO:1902903	2.61E-16
ventricular septum development	Biological Process	GO:0003281	2.62E-16
cellular response to inorganic substance	Biological Process	GO:0071241	2.72E-16
positive regulation of neuron apoptotic process	Biological Process	GO:0043525	2.77E-16
response to xenobiotic stimulus	Biological Process	GO:0009410	2.86E-16
peptidyl-lysine modification	Biological Process	GO:0018205	3.02E-16
endothelial cell proliferation	Biological Process	GO:0001935	3.23E-16
positive regulation of transmembrane receptor protein serine/threonine kinase signaling pathway	Biological Process	GO:0090100	3.81E-16
response to hexose	Biological Process	GO:0009746	3.97E-16
regulation of cell cycle G1/S phase transition	Biological Process	GO:1902806	3.97E-16
ureteric bud morphogenesis	Biological Process	GO:0060675	4.01E-16
positive regulation of endopeptidase activity	Biological Process	GO:0010950	4.23E-16
receptor metabolic process	Biological Process	GO:0043112	4.67E-16
negative regulation of protein serine/threonine kinase activity	Biological Process	GO:0071901	4.77E-16
prostate gland development	Biological Process	GO:0030850	4.87E-16

nephron epithelium morphogenesis	Biological Process	GO:0072088	5.35E-16
metanephros development	Biological Process	GO:0001656	5.42E-16
chondrocyte differentiation	Biological Process	GO:0002062	5.47E-16
regulation of T cell differentiation	Biological Process	GO:0045580	5.87E-16
Notch signaling pathway	Biological Process	GO:0007219	6.71E-16
intracellular steroid hormone receptor signaling pathway	Biological Process	GO:0030518	7.75E-16
response to glucose	Biological Process	GO:0009749	8.09E-16
regulation of hormone secretion	Biological Process	GO:0046883	8.23E-16
mesonephric tubule morphogenesis	Biological Process	GO:0072171	8.92E-16
cytokine metabolic process	Biological Process	GO:0042107	8.97E-16
dendrite development	Biological Process	GO:0016358	9.13E-16
positive regulation of cellular protein catabolic process	Biological Process	GO:1903364	9.20E-16
regulation of cellular component size	Biological Process	GO:0032535	9.28E-16
negative regulation of DNA-binding transcription factor activity	Biological Process	GO:0043433	9.87E-16
neural precursor cell proliferation	Biological Process	GO:0061351	1.00E-15
response to fibroblast growth factor	Biological Process	GO:0071774	1.00E-15
regulation of muscle system process	Biological Process	GO:0090257	1.00E-15
regulation of morphogenesis of a branching structure	Biological Process	GO:0060688	1.04E-15
regulation of kidney development	Biological Process	GO:0090183	1.04E-15
regulation of DNA metabolic process	Biological Process	GO:0051052	1.09E-15
cell-substrate junction assembly	Biological Process	GO:0007044	1.09E-15
cell-substrate junction organization	Biological Process	GO:0150115	1.09E-15
positive regulation of chromosome organization	Biological Process	GO:2001252	1.23E-15
lymphocyte activation involved in immune response	Biological Process	GO:0002285	1.30E-15
transcription initiation from RNA polymerase II promoter	Biological Process	GO:0006367	1.34E-15
regulation of fibroblast proliferation	Biological Process	GO:0048145	1.41E-15
regulation of response to biotic stimulus	Biological Process	GO:0002831	1.49E-15
negative regulation of cellular component movement	Biological Process	GO:0051271	1.49E-15
alpha-beta T cell activation	Biological Process	GO:0046631	1.57E-15
branching involved in ureteric bud morphogenesis	Biological Process	GO:0001658	1.58E-15
actomyosin structure organization	Biological Process	GO:0031032	1.60E-15
regulation of cell junction assembly	Biological Process	GO:1901888	1.60E-15
glial cell development	Biological Process	GO:0021782	1.72E-15
negative regulation of locomotion	Biological Process	GO:0040013	1.87E-15
regulation of intrinsic apoptotic signaling pathway	Biological Process	GO:2001242	1.94E-15
developmental maturation	Biological Process	GO:0021700	2.04E-15

negative regulation of cell growth	Biological Process	GO:0030308	2.28E-15
cytokine biosynthetic process	Biological Process	GO:0042089	2.48E-15
fibroblast proliferation	Biological Process	GO:0048144	2.66E-15
regulation of NIK/NF-kappaB signaling	Biological Process	GO:1901222	2.84E-15
response to glucocorticoid	Biological Process	GO:0051384	2.98E-15
bone morphogenesis	Biological Process	GO:0060349	2.99E-15
positive regulation of protein binding	Biological Process	GO:0032092	3.24E-15
neural tube formation	Biological Process	GO:0001841	3.33E-15
cellular response to fibroblast growth factor stimulus	Biological Process	GO:0044344	3.34E-15
cold-induced thermogenesis	Biological Process	GO:0106106	3.34E-15
regulation of cold-induced thermogenesis	Biological Process	GO:0120161	3.34E-15
positive regulation of reactive oxygen species metabolic process	Biological Process	GO:2000379	3.34E-15
cellular response to hydrogen peroxide	Biological Process	GO:0070301	3.34E-15
response to BMP	Biological Process	GO:0071772	3.58E-15
cellular response to BMP stimulus	Biological Process	GO:0071773	3.58E-15
positive chemotaxis	Biological Process	GO:0050918	3.96E-15
response to ionizing radiation	Biological Process	GO:0010212	4.50E-15
regulation of muscle cell differentiation	Biological Process	GO:0051147	4.60E-15
regulation of mitotic nuclear division	Biological Process	GO:0007088	4.92E-15
regulation of alpha-beta T cell differentiation	Biological Process	GO:0046637	5.00E-15
positive regulation of mononuclear cell proliferation	Biological Process	GO:0032946	5.21E-15
negative regulation of mitotic cell cycle	Biological Process	GO:0045930	5.48E-15
cell-cell junction organization	Biological Process	GO:0045216	5.53E-15
kidney morphogenesis	Biological Process	GO:0060993	5.69E-15
nephron tubule morphogenesis	Biological Process	GO:0072078	5.87E-15
negative regulation of Wnt signaling pathway	Biological Process	GO:0030178	5.90E-15
regulation of wound healing	Biological Process	GO:0061041	6.80E-15
erythrocyte homeostasis	Biological Process	GO:0034101	6.85E-15
protein localization to nucleus	Biological Process	GO:0034504	6.97E-15
negative regulation of catabolic process	Biological Process	GO:0009895	7.40E-15
regulation of cell size	Biological Process	GO:0008361	7.80E-15
cellular response to interleukin-1	Biological Process	GO:0071347	7.80E-15
positive regulation of protein-containing complex assembly	Biological Process	GO:0031334	8.45E-15
protein acetylation	Biological Process	GO:0006473	8.66E-15
regulation of protein ubiquitination	Biological Process	GO:0031396	8.66E-15
ERBB signaling pathway	Biological Process	GO:0038127	8.77E-15

chemokine production	Biological Process	GO:0032602	9.46E-15
intrinsic apoptotic signaling pathway in response to DNA damage	Biological Process	GO:0008630	9.64E-15
contractile actin filament bundle assembly	Biological Process	GO:0030038	9.95E-15
stress fiber assembly	Biological Process	GO:0043149	9.95E-15
positive regulation of immune effector process	Biological Process	GO:0002699	1.04E-14
ear development	Biological Process	GO:0043583	1.04E-14
regulation of osteoclast differentiation	Biological Process	GO:0045670	1.06E-14
negative regulation of cell-cell adhesion	Biological Process	GO:0022408	1.11E-14
developmental cell growth	Biological Process	GO:0048588	1.16E-14
regulation of tumor necrosis factor superfamily cytokine production	Biological Process	GO:1903555	1.22E-14
pancreas development	Biological Process	GO:0031016	1.29E-14
erythrocyte differentiation	Biological Process	GO:0030218	1.37E-14
positive regulation of lymphocyte proliferation	Biological Process	GO:0050671	1.40E-14
response to amyloid-beta	Biological Process	GO:1904645	1.41E-14
regulation of intracellular transport	Biological Process	GO:0032386	1.45E-14
leukocyte chemotaxis	Biological Process	GO:0030595	1.58E-14
BMP signaling pathway	Biological Process	GO:0030509	1.67E-14
regulation of epithelial to mesenchymal transition	Biological Process	GO:0010717	1.67E-14
positive regulation of histone modification	Biological Process	GO:0031058	1.67E-14
response to vitamin	Biological Process	GO:0033273	1.70E-14
apoptotic mitochondrial changes	Biological Process	GO:0008637	1.70E-14
establishment or maintenance of cell polarity	Biological Process	GO:0007163	1.87E-14
animal organ regeneration	Biological Process	GO:0031100	1.91E-14
anatomical structure maturation	Biological Process	GO:0071695	1.91E-14
endoderm development	Biological Process	GO:0007492	2.18E-14
tumor necrosis factor superfamily cytokine production	Biological Process	GO:0071706	2.19E-14
positive regulation of small molecule metabolic process	Biological Process	GO:0062013	2.28E-14
positive regulation of endothelial cell proliferation	Biological Process	GO:0001938	2.36E-14
heart growth	Biological Process	GO:0060419	2.36E-14
positive regulation of blood vessel endothelial cell migration	Biological Process	GO:0043536	2.41E-14
negative regulation of epithelial cell proliferation	Biological Process	GO:0050680	2.58E-14
regulation of mitochondrion organization	Biological Process	GO:0010821	2.67E-14
positive regulation of chromatin organization	Biological Process	GO:1905269	2.77E-14
regulation of neural precursor cell proliferation	Biological Process	GO:2000177	2.87E-14
regulation of cellular response to transforming growth factor beta stimulus	Biological Process	GO:1903844	2.94E-14
positive regulation of protein localization to membrane	Biological Process	GO:1905477	2.94E-14

peptidyl-lysine acetylation	Biological Process	GO:0018394	3.13E-14
pallium development	Biological Process	GO:0021543	3.13E-14
positive regulation of cell growth	Biological Process	GO:0030307	3.13E-14
positive regulation of epithelial to mesenchymal transition	Biological Process	GO:0010718	3.35E-14
glial cell proliferation	Biological Process	GO:0014009	3.35E-14
negative regulation of cellular response to growth factor stimulus	Biological Process	GO:0090288	3.71E-14
negative regulation of developmental growth	Biological Process	GO:0048640	3.79E-14
regulation of endocytosis	Biological Process	GO:0030100	3.79E-14
regulation of ubiquitin-dependent protein catabolic process	Biological Process	GO:2000058	3.82E-14
regulation of production of molecular mediator of immune response	Biological Process	GO:0002700	3.88E-14
protein localization to cell periphery	Biological Process	GO:1990778	4.25E-14
positive regulation of DNA metabolic process	Biological Process	GO:0051054	4.27E-14
negative regulation of MAPK cascade	Biological Process	GO:0043409	4.42E-14
actin filament bundle assembly	Biological Process	GO:0051017	4.85E-14
ephrin receptor signaling pathway	Biological Process	GO:0048013	4.90E-14
response to fatty acid	Biological Process	GO:0070542	4.90E-14
positive regulation of endocytosis	Biological Process	GO:0045807	4.91E-14
actin filament organization	Biological Process	GO:0007015	4.95E-14
positive regulation of autophagy	Biological Process	GO:0010508	5.03E-14
regulation of transforming growth factor beta receptor signaling pathway	Biological Process	GO:0017015	5.03E-14
regulation of tumor necrosis factor production	Biological Process	GO:0032680	5.09E-14
male sex differentiation	Biological Process	GO:0046661	5.09E-14
regulation of nuclear division	Biological Process	GO:0051783	5.09E-14
viral life cycle	Biological Process	GO:0019058	5.48E-14
myeloid leukocyte migration	Biological Process	GO:0097529	5.78E-14
inner ear development	Biological Process	GO:0048839	5.89E-14
actin filament bundle organization	Biological Process	GO:0061572	5.98E-14
endothelial cell differentiation	Biological Process	GO:0045446	5.99E-14
regulation of chromatin organization	Biological Process	GO:1902275	6.13E-14
negative regulation of canonical Wnt signaling pathway	Biological Process	GO:0090090	6.28E-14
positive regulation of cell morphogenesis involved in differentiation	Biological Process	GO:0010770	7.02E-14
regulation of lipid kinase activity	Biological Process	GO:0043550	7.41E-14
cardiac muscle tissue growth	Biological Process	GO:0055017	7.42E-14
production of miRNAs involved in gene silencing by miRNA	Biological Process	GO:0035196	7.60E-14
cytokine production involved in immune response	Biological Process	GO:0002367	7.82E-14
negative regulation of cysteine-type endopeptidase activity involved in apoptotic process	Biological Process	GO:0043154	8.34E-14

myoblast differentiation	Biological Process	GO:0045445	8.34E-14
embryonic placenta development	Biological Process	GO:0001892	8.52E-14
negative regulation of cell cycle G1/S phase transition	Biological Process	GO:1902807	8.52E-14
protein deacetylation	Biological Process	GO:0006476	8.52E-14
regulation of alpha-beta T cell activation	Biological Process	GO:0046634	8.52E-14
cellular response to ketone	Biological Process	GO:1901655	8.52E-14
positive regulation of supramolecular fiber organization	Biological Process	GO:1902905	8.52E-14
bone remodeling	Biological Process	GO:0046849	8.54E-14
negative regulation of cell migration	Biological Process	GO:0030336	8.69E-14
positive regulation of cytokine biosynthetic process	Biological Process	GO:0042108	8.79E-14
regulation of peptidyl-lysine acetylation	Biological Process	GO:2000756	9.53E-14
DNA-templated transcription, initiation	Biological Process	GO:0006352	9.84E-14
negative regulation of hemopoiesis	Biological Process	GO:1903707	1.01E-13
regulation of endothelial cell proliferation	Biological Process	GO:0001936	1.05E-13
response to ethanol	Biological Process	GO:0045471	1.08E-13
negative regulation of G1/S transition of mitotic cell cycle	Biological Process	GO:2000134	1.08E-13
response to fluid shear stress	Biological Process	GO:0034405	1.10E-13
mesenchymal cell proliferation	Biological Process	GO:0010463	1.11E-13
positive regulation of protein modification by small protein conjugation or removal	Biological Process	GO:1903322	1.12E-13
regulation of signal transduction by p53 class mediator	Biological Process	GO:1901796	1.23E-13
internal protein amino acid acetylation	Biological Process	GO:0006475	1.26E-13
odontogenesis	Biological Process	GO:0042476	1.28E-13
cardiac muscle cell differentiation	Biological Process	GO:0055007	1.28E-13
peptide hormone secretion	Biological Process	GO:0030072	1.28E-13
regulation of intracellular protein transport	Biological Process	GO:0033157	1.28E-13
DNA replication	Biological Process	GO:0006260	1.31E-13
primary neural tube formation	Biological Process	GO:0014020	1.39E-13
positive regulation of developmental growth	Biological Process	GO:0048639	1.42E-13
positive regulation of lymphocyte differentiation	Biological Process	GO:0045621	1.43E-13
positive regulation of mitotic cell cycle phase transition	Biological Process	GO:1901992	1.45E-13
positive regulation of leukocyte proliferation	Biological Process	GO:0070665	1.45E-13
regulation of JUN kinase activity	Biological Process	GO:0043506	1.45E-13
tumor necrosis factor production	Biological Process	GO:0032640	1.48E-13
response to amino acid	Biological Process	GO:0043200	1.61E-13
cellular response to metal ion	Biological Process	GO:0071248	1.62E-13
negative regulation of cell motility	Biological Process	GO:2000146	1.69E-13

regulation of proteolysis involved in cellular protein catabolic process	Biological Process	GO:1903050	1.92E-13
modulation of chemical synaptic transmission	Biological Process	GO:0050804	2.01E-13
muscle cell migration	Biological Process	GO:0014812	2.05E-13
B cell activation	Biological Process	GO:0042113	2.08E-13
regulation of leukocyte migration	Biological Process	GO:0002685	2.09E-13
CD4-positive, alpha-beta T cell differentiation	Biological Process	GO:0043367	2.13E-13
regulation of actomyosin structure organization	Biological Process	GO:0110020	2.40E-13
protein localization to plasma membrane	Biological Process	GO:0072659	2.43E-13
regulation of trans-synaptic signaling	Biological Process	GO:0099177	2.44E-13
negative regulation of cysteine-type endopeptidase activity	Biological Process	GO:2000117	2.47E-13
adaptive thermogenesis	Biological Process	GO:1990845	2.47E-13
regulation of leukocyte apoptotic process	Biological Process	GO:2000106	2.48E-13
roof of mouth development	Biological Process	GO:0060021	2.50E-13
positive regulation of transmembrane transport	Biological Process	GO:0034764	2.60E-13
regulation of neurotransmitter levels	Biological Process	GO:0001505	2.60E-13
endocrine pancreas development	Biological Process	GO:0031018	2.83E-13
pattern recognition receptor signaling pathway	Biological Process	GO:0002221	2.85E-13
regulation of response to cytokine stimulus	Biological Process	GO:0060759	3.09E-13
aorta development	Biological Process	GO:0035904	3.26E-13
central nervous system neuron differentiation	Biological Process	GO:0021953	3.29E-13
regulation of axonogenesis	Biological Process	GO:0050770	3.29E-13
regulation of cartilage development	Biological Process	GO:0061035	3.63E-13
modulation of process of other organism involved in symbiotic interaction	Biological Process	GO:0051817	3.77E-13
female sex differentiation	Biological Process	GO:0046660	3.96E-13
regulation of protein localization to cell periphery	Biological Process	GO:1904375	3.96E-13
response to antineoplastic agent	Biological Process	GO:0097327	3.96E-13
alpha-beta T cell activation involved in immune response	Biological Process	GO:0002287	4.03E-13
alpha-beta T cell differentiation involved in immune response	Biological Process	GO:0002293	4.03E-13
cell death in response to oxidative stress	Biological Process	GO:0036473	4.12E-13
protein dephosphorylation	Biological Process	GO:0006470	4.22E-13
neural tube closure	Biological Process	GO:0001843	4.24E-13
striated muscle cell proliferation	Biological Process	GO:0014855	4.24E-13
establishment of cell polarity	Biological Process	GO:0030010	4.25E-13
dephosphorylation	Biological Process	GO:0016311	4.26E-13
interaction with host	Biological Process	GO:0051701	4.28E-13
interleukin-12 production	Biological Process	GO:0032615	5.33E-13

epidermal growth factor receptor signaling pathway	Biological Process	GO:0007173	5.46E-13
protein deacylation	Biological Process	GO:0035601	5.68E-13
negative regulation of leukocyte differentiation	Biological Process	GO:1902106	5.68E-13
toll-like receptor signaling pathway	Biological Process	GO:0002224	5.81E-13
specification of animal organ identity	Biological Process	GO:0010092	5.92E-13
regulation of protein localization to nucleus	Biological Process	GO:1900180	6.11E-13
dsRNA processing	Biological Process	GO:0031050	6.42E-13
production of small RNA involved in gene silencing by RNA	Biological Process	GO:0070918	6.42E-13
regulation of actin filament bundle assembly	Biological Process	GO:0032231	6.48E-13
regulation of protein acetylation	Biological Process	GO:1901983	6.65E-13
protein stabilization	Biological Process	GO:0050821	6.65E-13
glucose homeostasis	Biological Process	GO:0042593	6.83E-13
positive regulation of cytoskeleton organization	Biological Process	GO:0051495	7.09E-13
tube closure	Biological Process	GO:0060606	7.09E-13
positive regulation of muscle tissue development	Biological Process	GO:1901863	7.30E-13
regulation of chemokine production	Biological Process	GO:0032642	7.40E-13
internal peptidyl-lysine acetylation	Biological Process	GO:0018393	8.61E-13
regulation of interleukin-12 production	Biological Process	GO:0032655	8.67E-13
carbohydrate homeostasis	Biological Process	GO:0033500	8.96E-13
macromolecule deacylation	Biological Process	GO:0098732	9.05E-13
negative regulation of extrinsic apoptotic signaling pathway via death domain receptors	Biological Process	GO:1902042	9.11E-13
regulation of heart morphogenesis	Biological Process	GO:2000826	9.11E-13
endothelial cell apoptotic process	Biological Process	GO:0072577	9.29E-13
regulation of innate immune response	Biological Process	GO:0045088	1.04E-12
T cell differentiation involved in immune response	Biological Process	GO:0002292	1.04E-12
sprouting angiogenesis	Biological Process	GO:0002040	1.04E-12
negative regulation of intrinsic apoptotic signaling pathway	Biological Process	GO:2001243	1.05E-12
negative regulation of binding	Biological Process	GO:0051100	1.17E-12
skeletal muscle organ development	Biological Process	GO:0060538	1.17E-12
B cell differentiation	Biological Process	GO:0030183	1.25E-12
positive regulation of NIK/NF-kappaB signaling	Biological Process	GO:1901224	1.26E-12
positive regulation of cell-substrate adhesion	Biological Process	GO:0010811	1.26E-12
positive regulation of T cell differentiation	Biological Process	GO:0045582	1.28E-12
regulation of cell cycle arrest	Biological Process	GO:0071156	1.30E-12
cellular response to radiation	Biological Process	GO:0071478	1.31E-12
negative regulation of hydrolase activity	Biological Process	GO:0051346	1.32E-12

CD4-positive, alpha-beta T cell differentiation involved in immune response	Biological Process	GO:0002294	1.34E-12
male gonad development	Biological Process	GO:0008584	1.35E-12
mesenchyme morphogenesis	Biological Process	GO:0072132	1.40E-12
regulation of Notch signaling pathway	Biological Process	GO:0008593	1.42E-12
positive regulation of leukocyte migration	Biological Process	GO:0002687	1.44E-12
receptor signaling pathway via JAK-STAT	Biological Process	GO:0007259	1.44E-12
neutrophil activation	Biological Process	GO:0042119	1.50E-12
regulation of cytokine-mediated signaling pathway	Biological Process	GO:0001959	1.53E-12
protein acylation	Biological Process	GO:0043543	1.57E-12
negative regulation of cell activation	Biological Process	GO:0050866	1.58E-12
eye morphogenesis	Biological Process	GO:0048592	1.72E-12
regulation of dendrite development	Biological Process	GO:0050773	1.72E-12
histone acetylation	Biological Process	GO:0016573	1.72E-12
regulation of T cell proliferation	Biological Process	GO:0042129	1.72E-12
regulation of blood vessel endothelial cell migration	Biological Process	GO:0043535	1.72E-12
negative regulation of organelle organization	Biological Process	GO:0010639	1.74E-12
cranial skeletal system development	Biological Process	GO:1904888	1.75E-12
neutrophil mediated immunity	Biological Process	GO:0002446	1.77E-12
positive regulation of interleukin-12 production	Biological Process	GO:0032735	1.79E-12
response to alkaloid	Biological Process	GO:0043279	1.79E-12
lymphocyte apoptotic process	Biological Process	GO:0070227	1.91E-12
development of primary male sexual characteristics	Biological Process	GO:0046546	1.95E-12
skeletal muscle tissue development	Biological Process	GO:0007519	2.01E-12
multicellular organism growth	Biological Process	GO:0035264	2.02E-12
platelet activation	Biological Process	GO:0030168	2.04E-12
positive regulation of inflammatory response	Biological Process	GO:0050729	2.04E-12
positive regulation of striated muscle tissue development	Biological Process	GO:0045844	2.08E-12
positive regulation of muscle organ development	Biological Process	GO:0048636	2.08E-12
positive regulation of cell division	Biological Process	GO:0051781	2.08E-12
neuroinflammatory response	Biological Process	GO:0150076	2.11E-12
regulation of stress fiber assembly	Biological Process	GO:0051492	2.13E-12
regulation of heart growth	Biological Process	GO:0060420	2.13E-12
lipopolysaccharide-mediated signaling pathway	Biological Process	GO:0031663	2.19E-12
positive regulation of cell cycle phase transition	Biological Process	GO:1901989	2.19E-12
positive regulation of kidney development	Biological Process	GO:0090184	2.36E-12
vascular process in circulatory system	Biological Process	GO:0003018	2.52E-12

positive regulation of actin filament bundle assembly	Biological Process	GO:0032233	2.59E-12
substrate adhesion-dependent cell spreading	Biological Process	GO:0034446	2.65E-12
modulation of process of other organism	Biological Process	GO:0035821	2.73E-12
regulation of small molecule metabolic process	Biological Process	GO:0062012	2.76E-12
synapse organization	Biological Process	GO:0050808	2.78E-12
cell cycle checkpoint	Biological Process	GO:0000075	2.84E-12
maternal process involved in female pregnancy	Biological Process	GO:0060135	2.94E-12
muscle adaptation	Biological Process	GO:0043500	3.09E-12
response to light stimulus	Biological Process	GO:0009416	3.15E-12
regulation of cellular response to oxidative stress	Biological Process	GO:1900407	3.46E-12
regulation of epithelial cell apoptotic process	Biological Process	GO:1904035	3.46E-12
second-messenger-mediated signaling	Biological Process	GO:0019932	3.66E-12
positive regulation of protein ubiquitination	Biological Process	GO:0031398	3.66E-12
positive regulation of cell cycle arrest	Biological Process	GO:0071158	3.69E-12
artery morphogenesis	Biological Process	GO:0048844	3.71E-12
response to virus	Biological Process	GO:0009615	3.86E-12
cellular response to amyloid-beta	Biological Process	GO:1904646	3.86E-12
cytokine secretion	Biological Process	GO:0050663	3.87E-12
response to starvation	Biological Process	GO:0042594	3.88E-12
insulin receptor signaling pathway	Biological Process	GO:0008286	4.03E-12
appendage morphogenesis	Biological Process	GO:0035107	4.08E-12
limb morphogenesis	Biological Process	GO:0035108	4.08E-12
fibroblast growth factor receptor signaling pathway	Biological Process	GO:0008543	4.14E-12
mitotic nuclear division	Biological Process	GO:0140014	4.62E-12
positive regulation of fibroblast proliferation	Biological Process	GO:0048146	4.79E-12
positive regulation of stress fiber assembly	Biological Process	GO:0051496	4.79E-12
hindbrain development	Biological Process	GO:0030902	4.84E-12
glomerulus development	Biological Process	GO:0032835	4.96E-12
CD4-positive, alpha-beta T cell activation	Biological Process	GO:0035710	5.33E-12
insulin secretion	Biological Process	GO:0030073	5.38E-12
mesoderm development	Biological Process	GO:0007498	5.62E-12
mammary gland morphogenesis	Biological Process	GO:0060443	5.71E-12
regulation of actin filament organization	Biological Process	GO:0110053	5.77E-12
cell adhesion mediated by integrin	Biological Process	GO:0033627	5.92E-12
positive regulation of myeloid leukocyte differentiation	Biological Process	GO:0002763	6.03E-12
regulation of interleukin-2 production	Biological Process	GO:0032663	6.03E-12

cell differentiation involved in kidney development	Biological Process	GO:0061005	6.03E-12
cellular response to ionizing radiation	Biological Process	GO:0071479	6.19E-12
neutrophil degranulation	Biological Process	GO:0043312	6.34E-12
histone deacetylation	Biological Process	GO:0016575	6.40E-12
regulation of proteasomal ubiquitin-dependent protein catabolic process	Biological Process	GO:0032434	7.11E-12
regulation of peptide hormone secretion	Biological Process	GO:0090276	7.11E-12
cell fate specification	Biological Process	GO:0001708	7.21E-12
non-canonical Wnt signaling pathway	Biological Process	GO:0035567	8.20E-12
steroid metabolic process	Biological Process	GO:0008202	8.94E-12
endocardial cushion development	Biological Process	GO:0003197	9.10E-12
negative regulation of response to wounding	Biological Process	GO:1903035	9.27E-12
positive regulation of ion transport	Biological Process	GO:0043270	9.76E-12
macroautophagy	Biological Process	GO:0016236	9.87E-12
regulation of transcription regulatory region DNA binding	Biological Process	GO:2000677	1.01E-11
integrin-mediated signaling pathway	Biological Process	GO:0007229	1.02E-11
regulation of cytokine production involved in immune response	Biological Process	GO:0002718	1.04E-11
regulation of smooth muscle cell migration	Biological Process	GO:0014910	1.04E-11
neutrophil activation involved in immune response	Biological Process	GO:0002283	1.05E-11
focal adhesion assembly	Biological Process	GO:0048041	1.08E-11
somatic stem cell population maintenance	Biological Process	GO:0035019	1.10E-11
regulation of cardiac muscle tissue growth	Biological Process	GO:0055021	1.10E-11
regulation of intracellular steroid hormone receptor signaling pathway	Biological Process	GO:0033143	1.11E-11
regulation of phosphatidylinositol 3-kinase activity	Biological Process	GO:0043551	1.22E-11
positive regulation of proteolysis involved in cellular protein catabolic process	Biological Process	GO:1903052	1.22E-11
heart process	Biological Process	GO:0003015	1.23E-11
regulation of cytokine secretion	Biological Process	GO:0050707	1.24E-11
regulation of CD4-positive, alpha-beta T cell differentiation	Biological Process	GO:0043370	1.26E-11
positive regulation of alpha-beta T cell differentiation	Biological Process	GO:0046638	1.26E-11
regulation of extrinsic apoptotic signaling pathway in absence of ligand	Biological Process	GO:2001239	1.26E-11
regulation of response to DNA damage stimulus	Biological Process	GO:2001020	1.31E-11
regulation of animal organ formation	Biological Process	GO:0003156	1.35E-11
semi-lunar valve development	Biological Process	GO:1905314	1.35E-11
T-helper cell differentiation	Biological Process	GO:0042093	1.41E-11
glial cell activation	Biological Process	GO:0061900	1.41E-11
smooth muscle cell migration	Biological Process	GO:0014909	1.48E-11
regulation of BMP signaling pathway	Biological Process	GO:0030510	1.48E-11

regulation of B cell proliferation	Biological Process	GO:0030888	1.59E-11
regulation of phospholipid metabolic process	Biological Process	GO:1903725	1.59E-11
endoderm formation	Biological Process	GO:0001706	1.65E-11
mammary gland epithelial cell proliferation	Biological Process	GO:0033598	1.67E-11
biomineral tissue development	Biological Process	GO:0031214	1.73E-11
biomineralization	Biological Process	GO:0110148	1.73E-11
development of primary female sexual characteristics	Biological Process	GO:0046545	1.75E-11
vasculogenesis	Biological Process	GO:0001570	1.85E-11
muscle hypertrophy	Biological Process	GO:0014896	1.85E-11
cellular response to starvation	Biological Process	GO:0009267	1.93E-11
nucleotide-binding oligomerization domain containing signaling pathway	Biological Process	GO:0070423	1.98E-11
regulation of histone acetylation	Biological Process	GO:0035065	2.03E-11
endodermal cell differentiation	Biological Process	GO:0035987	2.03E-11
regulation of GTPase activity	Biological Process	GO:0043087	2.04E-11
regulation of protein localization to plasma membrane	Biological Process	GO:1903076	2.15E-11
positive regulation of ubiquitin-dependent protein catabolic process	Biological Process	GO:2000060	2.15E-11
response to temperature stimulus	Biological Process	GO:0009266	2.19E-11
response to gamma radiation	Biological Process	GO:0010332	2.39E-11
regulation of leukocyte mediated immunity	Biological Process	GO:0002703	2.50E-11
positive regulation of Wnt signaling pathway	Biological Process	GO:0030177	2.65E-11
regulation of mesenchymal cell proliferation	Biological Process	GO:0010464	2.87E-11
interleukin-2 production	Biological Process	GO:0032623	2.97E-11
learning or memory	Biological Process	GO:0007611	2.97E-11
striated muscle hypertrophy	Biological Process	GO:0014897	3.20E-11
limbic system development	Biological Process	GO:0021761	3.20E-11
mesoderm morphogenesis	Biological Process	GO:0048332	3.22E-11
positive regulation of intracellular transport	Biological Process	GO:0032388	3.26E-11
mitotic cell cycle checkpoint	Biological Process	GO:0007093	3.26E-11
positive regulation of gliogenesis	Biological Process	GO:0014015	3.28E-11
positive regulation of JUN kinase activity	Biological Process	GO:0043507	3.28E-11
female gonad development	Biological Process	GO:0008585	3.38E-11
transforming growth factor beta production	Biological Process	GO:0071604	3.41E-11
receptor signaling pathway via STAT	Biological Process	GO:0097696	3.62E-11
cardiac muscle hypertrophy	Biological Process	GO:0003300	3.64E-11
calcium-mediated signaling	Biological Process	GO:0019722	3.77E-11
axis elongation	Biological Process	GO:0003401	4.02E-11

positive regulation of oxidoreductase activity	Biological Process	GO:0051353	4.03E-11
neural crest cell differentiation	Biological Process	GO:0014033	4.10E-11
organelle fission	Biological Process	GO:0048285	4.45E-11
muscle cell apoptotic process	Biological Process	GO:0010657	4.69E-11
segmentation	Biological Process	GO:0035282	4.69E-11
regulation of metal ion transport	Biological Process	GO:0010959	4.99E-11
regulation of focal adhesion assembly	Biological Process	GO:0051893	5.06E-11
regulation of cell-substrate junction assembly	Biological Process	GO:0090109	5.06E-11
regulation of cell-substrate junction organization	Biological Process	GO:0150116	5.06E-11
nucleotide-binding domain, leucine rich repeat containing receptor signaling pathway	Biological Process	GO:0035872	5.18E-11
regulation of transforming growth factor beta production	Biological Process	GO:0071634	5.18E-11
negative regulation of signal transduction in absence of ligand	Biological Process	GO:1901099	5.18E-11
negative regulation of extrinsic apoptotic signaling pathway in absence of ligand	Biological Process	GO:2001240	5.18E-11
oligodendrocyte differentiation	Biological Process	GO:0048709	5.23E-11
regulation of response to oxidative stress	Biological Process	GO:1902882	5.23E-11
cellular senescence	Biological Process	GO:0090398	5.34E-11
bone mineralization	Biological Process	GO:0030282	6.14E-11
positive regulation of proteasomal protein catabolic process	Biological Process	GO:1901800	6.29E-11
regulation of adaptive immune response	Biological Process	GO:0002819	6.64E-11
type I interferon production	Biological Process	GO:0032606	6.79E-11
regulation of interleukin-10 production	Biological Process	GO:0032653	6.79E-11
cognition	Biological Process	GO:0050890	7.14E-11
epithelial cell differentiation involved in kidney development	Biological Process	GO:0035850	7.24E-11
ventricular septum morphogenesis	Biological Process	GO:0060412	7.24E-11
reactive nitrogen species metabolic process	Biological Process	GO:2001057	7.74E-11
developmental induction	Biological Process	GO:0031128	7.75E-11
anoikis	Biological Process	GO:0043276	7.75E-11
embryonic eye morphogenesis	Biological Process	GO:0048048	7.75E-11
morphogenesis of an epithelial sheet	Biological Process	GO:0002011	7.77E-11
interleukin-10 production	Biological Process	GO:0032613	7.77E-11
embryonic limb morphogenesis	Biological Process	GO:0030326	8.00E-11
embryonic appendage morphogenesis	Biological Process	GO:0035113	8.00E-11
positive regulation of bone mineralization	Biological Process	GO:0030501	8.18E-11
response to retinoic acid	Biological Process	GO:0032526	8.18E-11
regulation of lipid biosynthetic process	Biological Process	GO:0046890	8.19E-11
negative regulation of ossification	Biological Process	GO:0030279	8.23E-11

cellular response to UV	Biological Process	GO:0034644	8.23E-11
nitric oxide metabolic process	Biological Process	GO:0046209	8.23E-11
NIK/NF-kappaB signaling	Biological Process	GO:0038061	8.37E-11
regulation of proteasomal protein catabolic process	Biological Process	GO:0061136	8.37E-11
tissue homeostasis	Biological Process	GO:0001894	8.52E-11
cellular response to glucocorticoid stimulus	Biological Process	GO:0071385	8.53E-11
positive regulation of transcription regulatory region DNA binding	Biological Process	GO:2000679	8.72E-11
collagen metabolic process	Biological Process	GO:0032963	8.91E-11
positive regulation of production of molecular mediator of immune response	Biological Process	GO:0002702	8.94E-11
macrophage activation	Biological Process	GO:0042116	8.94E-11
cellular response to corticosteroid stimulus	Biological Process	GO:0071384	9.12E-11
regulation of CD4-positive, alpha-beta T cell activation	Biological Process	GO:2000514	9.12E-11
negative regulation of wound healing	Biological Process	GO:0061045	9.14E-11
response to epidermal growth factor	Biological Process	GO:0070849	9.18E-11
reactive oxygen species biosynthetic process	Biological Process	GO:1903409	9.34E-11
positive regulation of leukocyte mediated immunity	Biological Process	GO:0002705	9.40E-11
cell-cell junction assembly	Biological Process	GO:0007043	9.49E-11
regulation of insulin secretion	Biological Process	GO:0050796	9.49E-11
cardiac muscle cell proliferation	Biological Process	GO:0060038	9.64E-11
aortic valve development	Biological Process	GO:0003176	1.12E-10
regulation of type I interferon production	Biological Process	GO:0032479	1.13E-10
negative regulation of transmembrane receptor protein serine/threonine kinase signaling pathway	Biological Process	GO:0090101	1.13E-10
heart contraction	Biological Process	GO:0060047	1.13E-10
regulation of calcium ion transport	Biological Process	GO:0051924	1.16E-10
leukocyte homeostasis	Biological Process	GO:0001776	1.21E-10
activation of cysteine-type endopeptidase activity involved in apoptotic process	Biological Process	GO:0006919	1.21E-10
regulation of membrane permeability	Biological Process	GO:0090559	1.21E-10
negative regulation of neuron projection development	Biological Process	GO:0010977	1.33E-10
regulation of muscle cell apoptotic process	Biological Process	GO:0010660	1.37E-10
T cell activation involved in immune response	Biological Process	GO:0002286	1.39E-10
nitric oxide biosynthetic process	Biological Process	GO:0006809	1.51E-10
embryonic skeletal system morphogenesis	Biological Process	GO:0048704	1.55E-10
negative regulation of striated muscle tissue development	Biological Process	GO:0045843	1.57E-10
signal transduction involved in cell cycle checkpoint	Biological Process	GO:0072395	1.59E-10
negative regulation of muscle tissue development	Biological Process	GO:1901862	1.64E-10

pathway-restricted SMAD protein phosphorylation	Biological Process	GO:0060389	1.67E-10
cellular response to vascular endothelial growth factor stimulus	Biological Process	GO:0035924	1.67E-10
regulation of adaptive immune response based on somatic recombination of immune receptors built from immunoglobulin superfamily domains	Biological Process	GO:0002822	1.75E-10
positive regulation of leukocyte chemotaxis	Biological Process	GO:0002690	1.92E-10
regulation of blood circulation	Biological Process	GO:1903522	2.05E-10
positive regulation of cold-induced thermogenesis	Biological Process	GO:0120162	2.11E-10
aortic valve morphogenesis	Biological Process	GO:0003180	2.24E-10
prostate gland morphogenesis	Biological Process	GO:0060512	2.24E-10
positive regulation of proteasomal ubiquitin-dependent protein catabolic process	Biological Process	GO:0032436	2.30E-10
regulation of biomineral tissue development	Biological Process	GO:0070167	2.39E-10
regulation of biomineralization	Biological Process	GO:0110149	2.39E-10
positive regulation of cytokine secretion	Biological Process	GO:0050715	2.59E-10
mesoderm formation	Biological Process	GO:0001707	2.64E-10
import into cell	Biological Process	GO:0098657	2.69E-10
signal transduction involved in mitotic G1 DNA damage checkpoint	Biological Process	GO:0072431	2.70E-10
intracellular signal transduction involved in G1 DNA damage checkpoint	Biological Process	GO:1902400	2.70E-10
positive regulation of intracellular protein transport	Biological Process	GO:0090316	2.79E-10
negative regulation of muscle organ development	Biological Process	GO:0048635	2.84E-10
ovulation cycle	Biological Process	GO:0042698	2.87E-10
mitotic G1 DNA damage checkpoint	Biological Process	GO:0031571	2.91E-10
mitotic G1/S transition checkpoint	Biological Process	GO:0044819	2.91E-10
dendrite morphogenesis	Biological Process	GO:0048813	2.94E-10
mammary gland duct morphogenesis	Biological Process	GO:0060603	2.96E-10
histone phosphorylation	Biological Process	GO:0016572	2.97E-10
receptor biosynthetic process	Biological Process	GO:0032800	2.98E-10
prostate gland epithelium morphogenesis	Biological Process	GO:0060740	2.98E-10
regulation of tissue remodeling	Biological Process	GO:0034103	2.98E-10
regulation of lipase activity	Biological Process	GO:0060191	3.17E-10
stem cell development	Biological Process	GO:0048864	3.31E-10
regulation of vascular smooth muscle cell proliferation	Biological Process	GO:1904705	3.31E-10
vascular smooth muscle cell proliferation	Biological Process	GO:1990874	3.31E-10
negative regulation of cell projection organization	Biological Process	GO:0031345	3.31E-10
negative regulation of cell cycle phase transition	Biological Process	GO:1901988	3.54E-10
positive regulation of adaptive immune response	Biological Process	GO:0002821	3.54E-10
cardiac muscle contraction	Biological Process	GO:0060048	3.55E-10

positive regulation of T cell proliferation	Biological Process	GO:0042102	3.61E-10
regulation of immune effector process	Biological Process	GO:0002697	3.64E-10
cell maturation	Biological Process	GO:0048469	3.66E-10
muscle contraction	Biological Process	GO:0006936	3.88E-10
regulation of blood coagulation	Biological Process	GO:0030193	3.96E-10
regulation of tube size	Biological Process	GO:0035150	4.02E-10
regulation of chondrocyte differentiation	Biological Process	GO:0032330	4.21E-10
autophagy of mitochondrion	Biological Process	GO:0000422	4.26E-10
regulation of mitochondrial membrane permeability	Biological Process	GO:0046902	4.26E-10
mitochondrion disassembly	Biological Process	GO:0061726	4.26E-10
cardiac ventricle morphogenesis	Biological Process	GO:0003208	4.53E-10
endochondral bone morphogenesis	Biological Process	GO:0060350	4.53E-10
signal transduction involved in DNA integrity checkpoint	Biological Process	GO:0072401	4.53E-10
signal transduction involved in DNA damage checkpoint	Biological Process	GO:0072422	4.53E-10
negative regulation of mitotic cell cycle phase transition	Biological Process	GO:1901991	4.53E-10
T cell differentiation in thymus	Biological Process	GO:0033077	4.75E-10
regulation of stem cell proliferation	Biological Process	GO:0072091	4.75E-10
positive regulation of chemokine production	Biological Process	GO:0032722	4.83E-10
import into nucleus	Biological Process	GO:0051170	4.83E-10
cellular glucose homeostasis	Biological Process	GO:0001678	4.97E-10
apical junction assembly	Biological Process	GO:0043297	4.98E-10
G1 DNA damage checkpoint	Biological Process	GO:0044783	4.98E-10
neurotransmitter biosynthetic process	Biological Process	GO:0042136	5.17E-10
astrocyte development	Biological Process	GO:0014002	5.17E-10
ensheathment of neurons	Biological Process	GO:0007272	5.18E-10
axon ensheathment	Biological Process	GO:0008366	5.18E-10
muscle cell development	Biological Process	GO:0055001	5.33E-10
regulation of lipid localization	Biological Process	GO:1905952	5.44E-10
DNA damage checkpoint	Biological Process	GO:0000077	5.44E-10
negative regulation of cellular catabolic process	Biological Process	GO:0031330	5.61E-10
positive regulation of smooth muscle cell migration	Biological Process	GO:0014911	6.21E-10
positive regulation of biomineral tissue development	Biological Process	GO:0070169	6.21E-10
positive regulation of biomineralization	Biological Process	GO:0110151	6.21E-10
regulation of hemostasis	Biological Process	GO:1900046	6.25E-10
heart formation	Biological Process	GO:0060914	6.36E-10
organic hydroxy compound biosynthetic process	Biological Process	GO:1901617	6.77E-10

neuron projection extension	Biological Process	GO:1990138	6.91E-10
regulation of interspecies interactions between organisms	Biological Process	GO:0043903	6.92E-10
protein modification by small protein removal	Biological Process	GO:0070646	6.98E-10
positive regulation of adaptive immune response based on somatic recombination of immune receptors built from immunoglobulin superfamily domains	Biological Process	GO:0002824	6.98E-10
lung morphogenesis	Biological Process	GO:0060425	7.09E-10
negative regulation of lymphocyte activation	Biological Process	GO:0051250	7.42E-10
protein deubiquitination	Biological Process	GO:0016579	7.73E-10
response to interferon-gamma	Biological Process	GO:0034341	7.78E-10
regulation of leukocyte chemotaxis	Biological Process	GO:0002688	7.79E-10
regulation of cellular amide metabolic process	Biological Process	GO:0034248	8.04E-10
cellular response to amino acid stimulus	Biological Process	GO:0071230	8.19E-10
regulation of cell shape	Biological Process	GO:0008360	8.19E-10
cellular response to interferon-gamma	Biological Process	GO:0071346	8.23E-10
DNA damage response, signal transduction by p53 class mediator resulting in cell cycle arrest	Biological Process	GO:0006977	8.25E-10
androgen receptor signaling pathway	Biological Process	GO:0030521	8.48E-10
signal transduction involved in mitotic cell cycle checkpoint	Biological Process	GO:0072413	8.48E-10
signal transduction involved in mitotic DNA damage checkpoint	Biological Process	GO:1902402	8.48E-10
signal transduction involved in mitotic DNA integrity checkpoint	Biological Process	GO:1902403	8.48E-10
regulation of endothelial cell apoptotic process	Biological Process	GO:2000351	8.48E-10
regulation of pathway-restricted SMAD protein phosphorylation	Biological Process	GO:0060393	8.53E-10
regulation of cation transmembrane transport	Biological Process	GO:1904062	8.55E-10
myelination	Biological Process	GO:0042552	8.62E-10
regulation of coagulation	Biological Process	GO:0050818	8.78E-10
regulation of small GTPase mediated signal transduction	Biological Process	GO:0051056	8.90E-10
outflow tract septum morphogenesis	Biological Process	GO:0003148	8.92E-10
positive regulation of G2/M transition of mitotic cell cycle	Biological Process	GO:0010971	8.92E-10
enteroendocrine cell differentiation	Biological Process	GO:0035883	8.92E-10
protein import into nucleus	Biological Process	GO:0006606	8.95E-10
regulation of tube diameter	Biological Process	GO:0035296	8.95E-10
regulation of blood vessel diameter	Biological Process	GO:0097746	8.95E-10
regulation of signaling receptor activity	Biological Process	GO:0010469	8.99E-10
camera-type eye morphogenesis	Biological Process	GO:0048593	9.18E-10
nuclear division	Biological Process	GO:0000280	9.53E-10
movement in host environment	Biological Process	GO:0052126	9.63E-10

thymus development	Biological Process	GO:0048538	1.03E-09
regulation of viral process	Biological Process	GO:0050792	1.03E-09
embryonic camera-type eye development	Biological Process	GO:0031076	1.05E-09
regulation of glucose transmembrane transport	Biological Process	GO:0010827	1.07E-09
cardiac epithelial to mesenchymal transition	Biological Process	GO:0060317	1.10E-09
response to endoplasmic reticulum stress	Biological Process	GO:0034976	1.14E-09
regulation of oxidative stress-induced cell death	Biological Process	GO:1903201	1.17E-09
microglial cell activation	Biological Process	GO:0001774	1.19E-09
leukocyte activation involved in inflammatory response	Biological Process	GO:0002269	1.19E-09
positive regulation of tumor necrosis factor superfamily cytokine production	Biological Process	GO:1903557	1.19E-09
metanephric nephron morphogenesis	Biological Process	GO:0072273	1.22E-09
embryonic skeletal system development	Biological Process	GO:0048706	1.25E-09
positive regulation of cell-matrix adhesion	Biological Process	GO:0001954	1.31E-09
negative regulation of fat cell differentiation	Biological Process	GO:0045599	1.31E-09
regulation of skeletal muscle tissue development	Biological Process	GO:0048641	1.31E-09
response to bronchodilator	Biological Process	GO:0097366	1.31E-09
response to calcium ion	Biological Process	GO:0051592	1.34E-09
mesenchymal cell development	Biological Process	GO:0014031	1.34E-09
metanephric nephron development	Biological Process	GO:0072210	1.39E-09
platelet-derived growth factor receptor signaling pathway	Biological Process	GO:0048008	1.45E-09
cellular response to fatty acid	Biological Process	GO:0071398	1.45E-09
regulation of cell aging	Biological Process	GO:0090342	1.46E-09
tight junction organization	Biological Process	GO:0120193	1.46E-09
negative regulation of proteolysis	Biological Process	GO:0045861	1.51E-09
positive regulation of mitochondrion organization	Biological Process	GO:0010822	1.55E-09
positive regulation of gene silencing by miRNA	Biological Process	GO:2000637	1.58E-09
entry into host	Biological Process	GO:0044409	1.62E-09
negative regulation of leukocyte activation	Biological Process	GO:0002695	1.64E-09
endocardial cushion morphogenesis	Biological Process	GO:0003203	1.67E-09
cellular response to epidermal growth factor stimulus	Biological Process	GO:0071364	1.72E-09
regulation of lymphocyte mediated immunity	Biological Process	GO:0002706	1.80E-09
somite development	Biological Process	GO:0061053	1.80E-09
cellular response to alcohol	Biological Process	GO:0097306	1.80E-09
regulation of bone mineralization	Biological Process	GO:0030500	1.85E-09
cerebral cortex development	Biological Process	GO:0021987	1.85E-09
positive regulation of synaptic transmission	Biological Process	GO:0050806	1.93E-09

beta-catenin destruction complex disassembly	Biological Process	GO:1904886	1.93E-09
regulation of bicellular tight junction assembly	Biological Process	GO:2000810	1.93E-09
B cell activation involved in immune response	Biological Process	GO:0002312	2.01E-09
regulation of glial cell differentiation	Biological Process	GO:0045685	2.01E-09
positive regulation of tumor necrosis factor production	Biological Process	GO:0032760	2.05E-09
DNA integrity checkpoint	Biological Process	GO:0031570	2.10E-09
maintenance of location in cell	Biological Process	GO:0051651	2.15E-09
negative regulation of protein binding	Biological Process	GO:0032091	2.15E-09
regulation of phospholipase activity	Biological Process	GO:0010517	2.16E-09
SMAD protein signal transduction	Biological Process	GO:0060395	2.16E-09
response to osmotic stress	Biological Process	GO:0006970	2.32E-09
positive regulation of mitochondrial membrane permeability	Biological Process	GO:0035794	2.42E-09
bicellular tight junction assembly	Biological Process	GO:0070830	2.50E-09
neuroblast proliferation	Biological Process	GO:0007405	2.50E-09
regulation of oxidoreductase activity	Biological Process	GO:0051341	2.63E-09
response to iron ion	Biological Process	GO:0010039	2.64E-09
chromatin remodeling	Biological Process	GO:0006338	2.71E-09
cellular calcium ion homeostasis	Biological Process	GO:0006874	2.88E-09
positive regulation of type I interferon production	Biological Process	GO:0032481	2.90E-09
establishment of organelle localization	Biological Process	GO:0051656	3.00E-09
receptor internalization	Biological Process	GO:0031623	3.15E-09
Fc receptor signaling pathway	Biological Process	GO:0038093	3.31E-09
negative regulation of leukocyte cell-cell adhesion	Biological Process	GO:1903038	3.31E-09
embryonic camera-type eye morphogenesis	Biological Process	GO:0048596	3.61E-09
cellular response to carbohydrate stimulus	Biological Process	GO:0071322	3.68E-09
regulation of interferon-gamma production	Biological Process	GO:0032649	3.70E-09
regulation of receptor-mediated endocytosis	Biological Process	GO:0048259	3.70E-09
negative regulation of cell-substrate adhesion	Biological Process	GO:0010812	3.79E-09
regulation of erythrocyte differentiation	Biological Process	GO:0045646	3.84E-09
neural crest cell development	Biological Process	GO:0014032	4.04E-09
negative regulation of muscle cell differentiation	Biological Process	GO:0051148	4.04E-09
regulation of receptor signaling pathway via JAK-STAT	Biological Process	GO:0046425	4.12E-09
positive regulation of monooxygenase activity	Biological Process	GO:0032770	4.16E-09
somatic diversification of immune receptors via germline recombination within a single locus	Biological Process	GO:0002562	4.21E-09
somatic cell DNA recombination	Biological Process	GO:0016444	4.21E-09

bone resorption	Biological Process	GO:0045453	4.21E-09
positive regulation of alpha-beta T cell activation	Biological Process	GO:0046635	4.21E-09
positive regulation of cardiac muscle tissue development	Biological Process	GO:0055025	4.21E-09
regulation of lymphocyte apoptotic process	Biological Process	GO:0070228	4.32E-09
endothelial cell development	Biological Process	GO:0001885	4.34E-09
tight junction assembly	Biological Process	GO:0120192	4.38E-09
hexose transmembrane transport	Biological Process	GO:0008645	4.44E-09
positive regulation of lymphocyte mediated immunity	Biological Process	GO:0002708	4.47E-09
tissue regeneration	Biological Process	GO:0042246	4.50E-09
post-embryonic development	Biological Process	GO:0009791	4.65E-09
skin epidermis development	Biological Process	GO:0098773	4.65E-09
positive regulation of stem cell proliferation	Biological Process	GO:2000648	4.78E-09
cellular divalent inorganic cation homeostasis	Biological Process	GO:0072503	4.92E-09
B cell apoptotic process	Biological Process	GO:0001783	5.01E-09
atrioventricular valve morphogenesis	Biological Process	GO:0003181	5.01E-09
positive regulation of posttranscriptional gene silencing	Biological Process	GO:0060148	5.01E-09
peripheral nervous system development	Biological Process	GO:0007422	5.01E-09
B cell proliferation	Biological Process	GO:0042100	5.07E-09
carbohydrate transmembrane transport	Biological Process	GO:0034219	5.15E-09
calcium ion homeostasis	Biological Process	GO:0055074	5.22E-09
neuron migration	Biological Process	GO:0001764	6.01E-09
amyloid-beta formation	Biological Process	GO:0034205	6.02E-09
cell fate commitment involved in formation of primary germ layer	Biological Process	GO:0060795	6.02E-09
cellular response to lipoprotein particle stimulus	Biological Process	GO:0071402	6.02E-09
positive regulation of cytosolic calcium ion concentration	Biological Process	GO:0007204	6.10E-09
DNA biosynthetic process	Biological Process	GO:0071897	6.52E-09
regulation of cardiocyte differentiation	Biological Process	GO:1905207	6.54E-09
positive regulation of membrane permeability	Biological Process	GO:1905710	6.54E-09
glucose metabolic process	Biological Process	GO:0006006	6.64E-09
response to cadmium ion	Biological Process	GO:0046686	6.97E-09
regulation of release of cytochrome c from mitochondria	Biological Process	GO:0090199	7.09E-09
striated muscle cell development	Biological Process	GO:0055002	7.22E-09
columnar/cuboidal epithelial cell development	Biological Process	GO:0002066	7.52E-09
regulation of cardiac muscle cell proliferation	Biological Process	GO:0060043	7.52E-09
mitochondrial outer membrane permeabilization	Biological Process	GO:0097345	7.57E-09
hair follicle development	Biological Process	GO:0001942	8.02E-09

actin filament-based movement	Biological Process	GO:0030048	8.05E-09
regulation of ion transmembrane transport	Biological Process	GO:0034765	8.05E-09
axon extension	Biological Process	GO:0048675	8.18E-09
negative regulation of establishment of protein localization	Biological Process	GO:1904950	8.25E-09
vesicle organization	Biological Process	GO:0016050	8.31E-09
vesicle-mediated transport in synapse	Biological Process	GO:0099003	8.38E-09
branch elongation of an epithelium	Biological Process	GO:0060602	8.49E-09
monosaccharide transmembrane transport	Biological Process	GO:0015749	8.68E-09
regulation of circadian rhythm	Biological Process	GO:0042752	8.68E-09
regulation of transporter activity	Biological Process	GO:0032409	8.80E-09
negative regulation of cellular response to transforming growth factor beta stimulus	Biological Process	GO:1903845	9.20E-09
cell fate determination	Biological Process	GO:0001709	9.65E-09
response to dexamethasone	Biological Process	GO:0071548	9.65E-09
regulation of Ras protein signal transduction	Biological Process	GO:0046578	9.80E-09
midbrain development	Biological Process	GO:0030901	1.02E-08
odontogenesis of dentin-containing tooth	Biological Process	GO:0042475	1.02E-08
metencephalon development	Biological Process	GO:0022037	1.05E-08
lymphocyte migration	Biological Process	GO:0072676	1.05E-08
mitotic DNA damage checkpoint	Biological Process	GO:0044773	1.07E-08
intrinsic apoptotic signaling pathway in response to oxidative stress	Biological Process	GO:0008631	1.11E-08
cellular response to glucose stimulus	Biological Process	GO:0071333	1.12E-08
positive regulation of fat cell differentiation	Biological Process	GO:0045600	1.14E-08
regulation of cytosolic calcium ion concentration	Biological Process	GO:0051480	1.14E-08
regulation of muscle contraction	Biological Process	GO:0006937	1.16E-08
interleukin-1 production	Biological Process	GO:0032612	1.21E-08
positive regulation of nucleotide metabolic process	Biological Process	GO:0045981	1.21E-08
positive regulation of purine nucleotide metabolic process	Biological Process	GO:1900544	1.21E-08
regulation of transmembrane transporter activity	Biological Process	GO:0022898	1.22E-08
regulation of DNA replication	Biological Process	GO:0006275	1.26E-08
glucose transmembrane transport	Biological Process	GO:1904659	1.26E-08
negative regulation of epithelial cell differentiation	Biological Process	GO:0030857	1.28E-08
positive regulation of vascular smooth muscle cell proliferation	Biological Process	GO:1904707	1.28E-08
regulation of reactive oxygen species biosynthetic process	Biological Process	GO:1903426	1.28E-08
ventricular cardiac muscle tissue development	Biological Process	GO:0003229	1.30E-08
maternal placenta development	Biological Process	GO:0001893	1.30E-08
regulation of glial cell proliferation	Biological Process	GO:0060251	1.30E-08

positive regulation of morphogenesis of an epithelium	Biological Process	GO:1905332	1.30E-08
positive regulation of neural precursor cell proliferation	Biological Process	GO:2000179	1.30E-08
positive regulation of mesenchymal cell proliferation	Biological Process	GO:0002053	1.42E-08
glandular epithelial cell development	Biological Process	GO:0002068	1.42E-08
type B pancreatic cell differentiation	Biological Process	GO:0003309	1.42E-08
branching involved in mammary gland duct morphogenesis	Biological Process	GO:0060444	1.42E-08
molting cycle	Biological Process	GO:0042303	1.45E-08
hair cycle	Biological Process	GO:0042633	1.45E-08
negative regulation of T cell activation	Biological Process	GO:0050868	1.45E-08
negative regulation of myeloid cell differentiation	Biological Process	GO:0045638	1.48E-08
response to heat	Biological Process	GO:0009408	1.52E-08
positive regulation of cell cycle G2/M phase transition	Biological Process	GO:1902751	1.52E-08
negative regulation of transforming growth factor beta receptor signaling pathway	Biological Process	GO:0030512	1.57E-08
homotypic cell-cell adhesion	Biological Process	GO:0034109	1.57E-08
positive regulation of glucose import	Biological Process	GO:0046326	1.61E-08
glucose import	Biological Process	GO:0046323	1.65E-08
secondary alcohol metabolic process	Biological Process	GO:1902652	1.73E-08
molting cycle process	Biological Process	GO:0022404	1.73E-08
hair cycle process	Biological Process	GO:0022405	1.73E-08
neurotransmitter transport	Biological Process	GO:0006836	1.78E-08
regulation of muscle hypertrophy	Biological Process	GO:0014743	1.80E-08
central nervous system neuron development	Biological Process	GO:0021954	1.80E-08
negative regulation of MAP kinase activity	Biological Process	GO:0043407	1.80E-08
intrinsic apoptotic signaling pathway by p53 class mediator	Biological Process	GO:0072332	1.80E-08
response to anesthetic	Biological Process	GO:0072347	1.80E-08
cardiac cell development	Biological Process	GO:0055006	1.80E-08
regulation of monooxygenase activity	Biological Process	GO:0032768	1.80E-08
regulation of calcium ion transport into cytosol	Biological Process	GO:0010522	1.80E-08
monosaccharide metabolic process	Biological Process	GO:0005996	1.83E-08
activated T cell proliferation	Biological Process	GO:0050798	1.86E-08
intrinsic apoptotic signaling pathway in response to endoplasmic reticulum stress	Biological Process	GO:0070059	1.95E-08
mitochondrial outer membrane permeabilization involved in programmed cell death	Biological Process	GO:1902686	1.95E-08
cellular response to hexose stimulus	Biological Process	GO:0071331	1.99E-08
interferon-gamma production	Biological Process	GO:0032609	1.99E-08
axis specification	Biological Process	GO:0009798	2.00E-08
positive regulation of axonogenesis	Biological Process	GO:0050772	2.00E-08

regulation of metanephros development	Biological Process	GO:0072215	2.02E-08
negative regulation of protein transport	Biological Process	GO:0051224	2.03E-08
diencephalon development	Biological Process	GO:0021536	2.03E-08
positive regulation of glucose transmembrane transport	Biological Process	GO:0010828	2.04E-08
amyloid precursor protein catabolic process	Biological Process	GO:0042987	2.04E-08
release of cytochrome c from mitochondria	Biological Process	GO:0001836	2.06E-08
mitotic DNA integrity checkpoint	Biological Process	GO:0044774	2.09E-08
negative regulation of cyclin-dependent protein serine/threonine kinase activity	Biological Process	GO:0045736	2.09E-08
positive regulation of cartilage development	Biological Process	GO:0061036	2.09E-08
positive regulation of peptidyl-lysine acetylation	Biological Process	GO:2000758	2.09E-08
positive regulation of GTPase activity	Biological Process	GO:0043547	2.13E-08
T cell costimulation	Biological Process	GO:0031295	2.15E-08
positive regulation of protein localization to plasma membrane	Biological Process	GO:1903078	2.15E-08
G0 to G1 transition	Biological Process	GO:0045023	2.16E-08
genitalia development	Biological Process	GO:0048806	2.16E-08
activation of MAPKK activity	Biological Process	GO:0000186	2.20E-08
regulation of myoblast differentiation	Biological Process	GO:0045661	2.20E-08
cellular response to cAMP	Biological Process	GO:0071320	2.20E-08
response to ischemia	Biological Process	GO:0002931	2.20E-08
coronary vasculature development	Biological Process	GO:0060976	2.20E-08
regulation of cardiac muscle cell differentiation	Biological Process	GO:2000725	2.20E-08
negative regulation of secretion	Biological Process	GO:0051048	2.22E-08
regulation of striated muscle cell differentiation	Biological Process	GO:0051153	2.22E-08
negative regulation of cell aging	Biological Process	GO:0090344	2.34E-08
positive regulation of receptor signaling pathway via JAK-STAT	Biological Process	GO:0046427	2.48E-08
regulation of microtubule cytoskeleton organization	Biological Process	GO:0070507	2.50E-08
regulation of heart contraction	Biological Process	GO:0008016	2.50E-08
regulation of interleukin-1 production	Biological Process	GO:0032652	2.50E-08
regulation of chromosome segregation	Biological Process	GO:0051983	2.50E-08
cellular response to monosaccharide stimulus	Biological Process	GO:0071326	2.61E-08
lung epithelium development	Biological Process	GO:0060428	2.64E-08
positive regulation of lipid kinase activity	Biological Process	GO:0090218	2.64E-08
response to hyperoxia	Biological Process	GO:0055093	2.75E-08
regulation of production of small RNA involved in gene silencing by RNA	Biological Process	GO:0070920	2.75E-08
regulation of multicellular organism growth	Biological Process	GO:0040014	2.81E-08
regulation of nitric oxide biosynthetic process	Biological Process	GO:0045428	2.81E-08

regulation of mitochondrial membrane permeability involved in apoptotic process	Biological Process	GO:1902108	2.81E-08
regulation of muscle adaptation	Biological Process	GO:0043502	2.89E-08
tyrosine phosphorylation of STAT protein	Biological Process	GO:0007260	2.90E-08
amyloid precursor protein metabolic process	Biological Process	GO:0042982	3.08E-08
positive regulation of protein localization to cell periphery	Biological Process	GO:1904377	3.08E-08
regulation of synaptic plasticity	Biological Process	GO:0048167	3.14E-08
negative regulation of vasculature development	Biological Process	GO:1901343	3.14E-08
modulation by host of symbiont process	Biological Process	GO:0051851	3.33E-08
positive regulation of mitochondrial membrane permeability involved in apoptotic process	Biological Process	GO:1902110	3.33E-08
positive regulation of cyclin-dependent protein serine/threonine kinase activity	Biological Process	GO:0045737	3.39E-08
regulation of tyrosine phosphorylation of STAT protein	Biological Process	GO:0042509	3.39E-08
pituitary gland development	Biological Process	GO:0021983	3.47E-08
positive regulation of blood circulation	Biological Process	GO:1903524	3.47E-08
regulation of nucleocytoplasmic transport	Biological Process	GO:0046822	3.49E-08
lymphocyte costimulation	Biological Process	GO:0031294	3.54E-08
morphogenesis of an endothelium	Biological Process	GO:0003159	3.58E-08
endothelial tube morphogenesis	Biological Process	GO:0061154	3.58E-08
regulation of production of miRNAs involved in gene silencing by miRNA	Biological Process	GO:1903798	3.58E-08
atrioventricular valve development	Biological Process	GO:0003171	3.59E-08
regulation of oxidative stress-induced neuron death	Biological Process	GO:1903203	3.59E-08
protein localization to mitochondrion	Biological Process	GO:0070585	3.65E-08
granulocyte migration	Biological Process	GO:0097530	3.65E-08
embryonic cranial skeleton morphogenesis	Biological Process	GO:0048701	3.68E-08
negative regulation of DNA binding	Biological Process	GO:0043392	3.69E-08
positive regulation of receptor-mediated endocytosis	Biological Process	GO:0048260	3.77E-08
negative regulation of myeloid leukocyte differentiation	Biological Process	GO:0002762	3.77E-08
regulation of bone remodeling	Biological Process	GO:0046850	3.77E-08
face development	Biological Process	GO:0060324	3.77E-08
positive regulation of phospholipid metabolic process	Biological Process	GO:1903727	3.77E-08
negative regulation of secretion by cell	Biological Process	GO:1903531	3.80E-08
response to unfolded protein	Biological Process	GO:0006986	3.82E-08
somitogenesis	Biological Process	GO:0001756	3.88E-08
protein import	Biological Process	GO:0017038	3.93E-08
interleukin-1 beta production	Biological Process	GO:0032611	4.18E-08
neurotransmitter metabolic process	Biological Process	GO:0042133	4.21E-08

negative regulation of anoikis	Biological Process	GO:2000811	4.29E-08
negative regulation of cyclin-dependent protein kinase activity	Biological Process	GO:1904030	4.35E-08
granulocyte chemotaxis	Biological Process	GO:0071621	4.48E-08
interaction with symbiont	Biological Process	GO:0051702	4.53E-08
positive regulation of hormone metabolic process	Biological Process	GO:0032352	4.56E-08
glial cell apoptotic process	Biological Process	GO:0034349	4.56E-08
positive regulation of nitric-oxide synthase biosynthetic process	Biological Process	GO:0051770	4.56E-08
positive regulation of hormone secretion	Biological Process	GO:0046887	4.87E-08
synaptic vesicle cycle	Biological Process	GO:0099504	4.93E-08
regulation of receptor signaling pathway via STAT	Biological Process	GO:1904892	5.02E-08
smooth muscle cell differentiation	Biological Process	GO:0051145	5.21E-08
regulation of cellular response to insulin stimulus	Biological Process	GO:1900076	5.21E-08
positive regulation of protein localization to nucleus	Biological Process	GO:1900182	5.21E-08
response to vitamin D	Biological Process	GO:0033280	5.21E-08
dopaminergic neuron differentiation	Biological Process	GO:0071542	5.21E-08
cholesterol metabolic process	Biological Process	GO:0008203	5.23E-08
regulation of synapse organization	Biological Process	GO:0050807	5.69E-08
intracellular estrogen receptor signaling pathway	Biological Process	GO:0030520	5.73E-08
regulation of protein import	Biological Process	GO:1904589	5.73E-08
G2/M transition of mitotic cell cycle	Biological Process	GO:0000086	5.73E-08
cerebellum development	Biological Process	GO:0021549	5.82E-08
peptidyl-tyrosine dephosphorylation	Biological Process	GO:0035335	5.82E-08
regulation of bone resorption	Biological Process	GO:0045124	5.87E-08
sterol metabolic process	Biological Process	GO:0016125	5.88E-08
cell cycle DNA replication	Biological Process	GO:0044786	5.92E-08
regulation of steroid biosynthetic process	Biological Process	GO:0050810	6.02E-08
regulation of protein import into nucleus	Biological Process	GO:0042306	6.09E-08
negative regulation of cardiac muscle tissue development	Biological Process	GO:0055026	6.30E-08
somatic recombination of immunoglobulin gene segments	Biological Process	GO:0016447	6.37E-08
regulation of cellular senescence	Biological Process	GO:2000772	6.37E-08
neurotransmitter uptake	Biological Process	GO:0001504	6.50E-08
regulation of lipid storage	Biological Process	GO:0010883	6.50E-08
ovulation cycle process	Biological Process	GO:0022602	6.50E-08
digestive tract morphogenesis	Biological Process	GO:0048546	6.50E-08
positive regulation of extrinsic apoptotic signaling pathway	Biological Process	GO:2001238	6.50E-08
regulation of synapse structure or activity	Biological Process	GO:0050803	6.62E-08

lipid storage	Biological Process	GO:0019915	6.69E-08
hexose metabolic process	Biological Process	GO:0019318	7.16E-08
branching involved in blood vessel morphogenesis	Biological Process	GO:0001569	7.16E-08
ERBB2 signaling pathway	Biological Process	GO:0038128	7.16E-08
positive regulation of phosphatidylinositol 3-kinase activity	Biological Process	GO:0043552	7.16E-08
response to lipoprotein particle	Biological Process	GO:0055094	7.16E-08
cell surface receptor signaling pathway involved in heart development	Biological Process	GO:0061311	7.16E-08
positive regulation of receptor signaling pathway via STAT	Biological Process	GO:1904894	7.18E-08
proteasomal protein catabolic process	Biological Process	GO:0010498	7.34E-08
regulation of extent of cell growth	Biological Process	GO:0061387	7.42E-08
regulation of macroautophagy	Biological Process	GO:0016241	7.54E-08
cellular response to unfolded protein	Biological Process	GO:0034620	7.71E-08
somatic diversification of immune receptors	Biological Process	GO:0002200	7.72E-08
regulation of cardiac muscle hypertrophy	Biological Process	GO:0010611	7.72E-08
organ induction	Biological Process	GO:0001759	7.87E-08
regulation of receptor biosynthetic process	Biological Process	GO:0010869	7.87E-08
positive regulation of nitric-oxide synthase activity	Biological Process	GO:0051000	7.87E-08
positive regulation of transcription from RNA polymerase II promoter involved in cellular response to chemical stimulus	Biological Process	GO:1901522	7.87E-08
proteasome-mediated ubiquitin-dependent protein catabolic process	Biological Process	GO:0043161	8.27E-08
cytoplasmic pattern recognition receptor signaling pathway	Biological Process	GO:0002753	8.29E-08
embryonic pattern specification	Biological Process	GO:0009880	8.29E-08
circadian regulation of gene expression	Biological Process	GO:0032922	8.29E-08
positive regulation of response to biotic stimulus	Biological Process	GO:0002833	8.29E-08
regulation of cardiac muscle contraction	Biological Process	GO:0055117	8.32E-08
carbohydrate transport	Biological Process	GO:0008643	8.34E-08
neuron death in response to oxidative stress	Biological Process	GO:0036475	8.50E-08
aorta morphogenesis	Biological Process	GO:0035909	8.67E-08
regulation of T-helper cell differentiation	Biological Process	GO:0045622	8.67E-08
positive regulation of cyclin-dependent protein kinase activity	Biological Process	GO:1904031	8.67E-08
positive regulation of lipase activity	Biological Process	GO:0060193	8.83E-08
regulation of establishment of protein localization to mitochondrion	Biological Process	GO:1903747	8.83E-08
organic hydroxy compound transport	Biological Process	GO:0015850	8.83E-08
positive regulation of viral process	Biological Process	GO:0048524	8.98E-08
platelet aggregation	Biological Process	GO:0070527	9.06E-08
establishment of protein localization to mitochondrion	Biological Process	GO:0072655	9.51E-08

viral genome replication	Biological Process	GO:0019079	9.75E-08
receptor-mediated endocytosis	Biological Process	GO:0006898	9.75E-08
negative regulation of endopeptidase activity	Biological Process	GO:0010951	9.97E-08
negative regulation of protein secretion	Biological Process	GO:0050709	9.97E-08
cellular response to retinoic acid	Biological Process	GO:0071300	1.01E-07
long-term synaptic potentiation	Biological Process	GO:0060291	1.01E-07
negative regulation of blood coagulation	Biological Process	GO:0030195	1.05E-07
lung alveolus development	Biological Process	GO:0048286	1.07E-07
establishment of endothelial barrier	Biological Process	GO:0061028	1.07E-07
regulation of neurotransmitter uptake	Biological Process	GO:0051580	1.11E-07
nitric-oxide synthase biosynthetic process	Biological Process	GO:0051767	1.11E-07
regulation of nitric-oxide synthase biosynthetic process	Biological Process	GO:0051769	1.11E-07
definitive hemopoiesis	Biological Process	GO:0060216	1.11E-07
histone H3 deacetylation	Biological Process	GO:0070932	1.11E-07
vascular endothelial growth factor signaling pathway	Biological Process	GO:0038084	1.11E-07
regulation of protein deacetylation	Biological Process	GO:0090311	1.11E-07
T cell selection	Biological Process	GO:0045058	1.11E-07
amyloid-beta metabolic process	Biological Process	GO:0050435	1.11E-07
positive regulation of production of miRNAs involved in gene silencing by miRNA	Biological Process	GO:1903800	1.12E-07
interleukin-6 production	Biological Process	GO:0032635	1.15E-07
dendritic spine development	Biological Process	GO:0060996	1.16E-07
positive regulation of interleukin-2 production	Biological Process	GO:0032743	1.16E-07
cellular response to gamma radiation	Biological Process	GO:0071480	1.16E-07
cellular response to dexamethasone stimulus	Biological Process	GO:0071549	1.16E-07
negative regulation of cell junction assembly	Biological Process	GO:1901889	1.16E-07
mitochondrial membrane organization	Biological Process	GO:0007006	1.17E-07
striated muscle contraction	Biological Process	GO:0006941	1.19E-07
regulation of interleukin-1 beta production	Biological Process	GO:0032651	1.20E-07
mononuclear cell migration	Biological Process	GO:0071674	1.20E-07
nuclear transport	Biological Process	GO:0051169	1.20E-07
positive regulation of organ growth	Biological Process	GO:0046622	1.28E-07
superoxide metabolic process	Biological Process	GO:0006801	1.31E-07
regulation of ion transmembrane transporter activity	Biological Process	GO:0032412	1.32E-07
regulation of heart rate	Biological Process	GO:0002027	1.32E-07
organelle disassembly	Biological Process	GO:1903008	1.32E-07
negative regulation of myoblast differentiation	Biological Process	GO:0045662	1.32E-07

positive regulation of focal adhesion assembly	Biological Process	GO:0051894	1.32E-07
apoptotic process involved in morphogenesis	Biological Process	GO:0060561	1.32E-07
regulation of mesonephros development	Biological Process	GO:0061217	1.32E-07
regulation of glucose import	Biological Process	GO:0046324	1.42E-07
calcium ion transport into cytosol	Biological Process	GO:0060402	1.43E-07
negative regulation of endothelial cell apoptotic process	Biological Process	GO:2000352	1.44E-07
activation of NF-kappaB-inducing kinase activity	Biological Process	GO:0007250	1.48E-07
I-kappaB phosphorylation	Biological Process	GO:0007252	1.48E-07
response to muscle stretch	Biological Process	GO:0035994	1.48E-07
regulation of amyloid-beta clearance	Biological Process	GO:1900221	1.48E-07
myeloid cell development	Biological Process	GO:0061515	1.51E-07
regulation of neurotransmitter transport	Biological Process	GO:0051588	1.60E-07
amyloid-beta clearance	Biological Process	GO:0097242	1.67E-07
positive regulation of Notch signaling pathway	Biological Process	GO:0045747	1.69E-07
negative regulation of hemostasis	Biological Process	GO:1900047	1.69E-07
negative regulation of smooth muscle cell proliferation	Biological Process	GO:0048662	1.73E-07
cell cycle G2/M phase transition	Biological Process	GO:0044839	1.80E-07
regulation of nitric-oxide synthase activity	Biological Process	GO:0050999	1.80E-07
platelet degranulation	Biological Process	GO:0002576	1.81E-07
peptidyl-threonine dephosphorylation	Biological Process	GO:0035970	1.86E-07
hepatocyte growth factor receptor signaling pathway	Biological Process	GO:0048012	1.86E-07
cytokinesis	Biological Process	GO:0000910	1.87E-07
alcohol biosynthetic process	Biological Process	GO:0046165	1.87E-07
negative regulation of peptidase activity	Biological Process	GO:0010466	1.88E-07
positive regulation of pathway-restricted SMAD protein phosphorylation	Biological Process	GO:0010862	1.88E-07
exocrine system development	Biological Process	GO:0035272	1.88E-07
regulation of amyloid-beta formation	Biological Process	GO:1902003	1.89E-07
positive regulation of leukocyte apoptotic process	Biological Process	GO:2000108	1.89E-07
negative regulation of protein catabolic process	Biological Process	GO:0042177	1.89E-07
negative regulation of striated muscle cell differentiation	Biological Process	GO:0051154	1.90E-07
autonomic nervous system development	Biological Process	GO:0048483	1.91E-07
regulation of G0 to G1 transition	Biological Process	GO:0070316	1.91E-07
negative regulation of NF-kappaB transcription factor activity	Biological Process	GO:0032088	2.02E-07
hippocampus development	Biological Process	GO:0021766	2.02E-07
lung cell differentiation	Biological Process	GO:0060479	2.03E-07
endoplasmic reticulum unfolded protein response	Biological Process	GO:0030968	2.10E-07

actin-mediated cell contraction	Biological Process	GO:0070252	2.10E-07
negative regulation of peptide secretion	Biological Process	GO:0002792	2.19E-07
regulation of interleukin-6 production	Biological Process	GO:0032675	2.23E-07
positive regulation of cellular amide metabolic process	Biological Process	GO:0034250	2.23E-07
positive regulation of tyrosine phosphorylation of STAT protein	Biological Process	GO:0042531	2.24E-07
cellular response to xenobiotic stimulus	Biological Process	GO:0071466	2.31E-07
cytosolic calcium ion transport	Biological Process	GO:0060401	2.32E-07
regulation of mRNA metabolic process	Biological Process	GO:1903311	2.37E-07
regulation of sprouting angiogenesis	Biological Process	GO:1903670	2.58E-07
regulation of axon extension	Biological Process	GO:0030516	2.69E-07
response to leukemia inhibitory factor	Biological Process	GO:1990823	2.69E-07
cellular response to leukemia inhibitory factor	Biological Process	GO:1990830	2.69E-07
retina development in camera-type eye	Biological Process	GO:0060041	2.81E-07
salivary gland morphogenesis	Biological Process	GO:0007435	2.84E-07
negative regulation of ion transport	Biological Process	GO:0043271	2.89E-07
positive regulation of lipid localization	Biological Process	GO:1905954	2.90E-07
nucleocytoplasmic transport	Biological Process	GO:0006913	2.92E-07
neuron fate commitment	Biological Process	GO:0048663	2.99E-07
T cell lineage commitment	Biological Process	GO:0002360	3.07E-07
negative regulation of embryonic development	Biological Process	GO:0045992	3.07E-07
regulation of positive chemotaxis	Biological Process	GO:0050926	3.07E-07
positive regulation of cell-substrate junction organization	Biological Process	GO:0150117	3.07E-07
sympathetic nervous system development	Biological Process	GO:0048485	3.07E-07
cellular response to fluid shear stress	Biological Process	GO:0071498	3.07E-07
cell proliferation involved in kidney development	Biological Process	GO:0072111	3.07E-07
positive regulation of stem cell differentiation	Biological Process	GO:2000738	3.07E-07
cellular response to nerve growth factor stimulus	Biological Process	GO:1990090	3.12E-07
salivary gland development	Biological Process	GO:0007431	3.12E-07
Schwann cell differentiation	Biological Process	GO:0014037	3.12E-07
regulation of activated T cell proliferation	Biological Process	GO:0046006	3.12E-07
body fluid secretion	Biological Process	GO:0007589	3.25E-07
protein destabilization	Biological Process	GO:0031648	3.25E-07
regulation of cell adhesion mediated by integrin	Biological Process	GO:0033628	3.25E-07
negative regulation of epithelial cell apoptotic process	Biological Process	GO:1904036	3.25E-07
negative regulation of organ growth	Biological Process	GO:0046621	3.27E-07
locomotory behavior	Biological Process	GO:0007626	3.30E-07

cortical actin cytoskeleton organization	Biological Process	GO:0030866	3.31E-07
negative regulation of Notch signaling pathway	Biological Process	GO:0045746	3.31E-07
calcium ion transport	Biological Process	GO:0006816	3.32E-07
neutrophil chemotaxis	Biological Process	GO:0030593	3.33E-07
positive regulation of calcium ion transport	Biological Process	GO:0051928	3.63E-07
regulation of DNA biosynthetic process	Biological Process	GO:2000278	3.66E-07
response to chemokine	Biological Process	GO:1990868	3.67E-07
cellular response to chemokine	Biological Process	GO:1990869	3.67E-07
positive regulation of phospholipase activity	Biological Process	GO:0010518	3.81E-07
forebrain generation of neurons	Biological Process	GO:0021872	3.81E-07
regulation of DNA-templated transcription in response to stress	Biological Process	GO:0043620	3.81E-07
regulation of transcription involved in G1/S transition of mitotic cell cycle	Biological Process	GO:0000083	4.01E-07
regulation of carbohydrate metabolic process	Biological Process	GO:0006109	4.05E-07
response to topologically incorrect protein	Biological Process	GO:0035966	4.05E-07
endomembrane system organization	Biological Process	GO:0010256	4.23E-07
regulation of stem cell differentiation	Biological Process	GO:2000736	4.24E-07
positive regulation of canonical Wnt signaling pathway	Biological Process	GO:0090263	4.45E-07
regulation of insulin receptor signaling pathway	Biological Process	GO:0046626	4.46E-07
negative regulation of lipid storage	Biological Process	GO:0010888	4.46E-07
toll-like receptor 3 signaling pathway	Biological Process	GO:0034138	4.46E-07
negative regulation of posttranscriptional gene silencing	Biological Process	GO:0060149	4.46E-07
negative regulation of gene silencing by RNA	Biological Process	GO:0060967	4.46E-07
cellular response to reactive nitrogen species	Biological Process	GO:1902170	4.46E-07
postsynapse organization	Biological Process	GO:0099173	4.57E-07
negative regulation of mitochondrion organization	Biological Process	GO:0010823	4.68E-07
p38MAPK cascade	Biological Process	GO:0038066	4.68E-07
regulation of steroid metabolic process	Biological Process	GO:0019218	4.74E-07
metanephros morphogenesis	Biological Process	GO:0003338	4.78E-07
response to mineralocorticoid	Biological Process	GO:0051385	4.78E-07
response to lead ion	Biological Process	GO:0010288	4.87E-07
secondary palate development	Biological Process	GO:0062009	4.87E-07
regulation of anoikis	Biological Process	GO:2000209	4.87E-07
lens development in camera-type eye	Biological Process	GO:0002088	4.88E-07
cell migration involved in sprouting angiogenesis	Biological Process	GO:0002042	5.00E-07
steroid biosynthetic process	Biological Process	GO:0006694	5.06E-07
anterior/posterior axis specification	Biological Process	GO:0009948	5.06E-07

regulation of immunoglobulin production	Biological Process	GO:0002637	5.10E-07
type 2 immune response	Biological Process	GO:0042092	5.32E-07
mesodermal cell differentiation	Biological Process	GO:0048333	5.32E-07
bone cell development	Biological Process	GO:0098751	5.32E-07
negative regulation of animal organ morphogenesis	Biological Process	GO:0110111	5.32E-07
regulation of amyloid precursor protein catabolic process	Biological Process	GO:1902991	5.32E-07
somatic recombination of immunoglobulin genes involved in immune response	Biological Process	GO:0002204	5.37E-07
somatic diversification of immunoglobulins involved in immune response	Biological Process	GO:0002208	5.37E-07
isotype switching	Biological Process	GO:0045190	5.37E-07
trabecula morphogenesis	Biological Process	GO:0061383	5.37E-07
T cell apoptotic process	Biological Process	GO:0070231	5.37E-07
positive regulation of ATP metabolic process	Biological Process	GO:1903580	5.37E-07
calcium ion transmembrane import into cytosol	Biological Process	GO:0097553	5.40E-07
memory	Biological Process	GO:0007613	5.54E-07
macrophage differentiation	Biological Process	GO:0030225	5.60E-07
regulation of mitochondrial outer membrane permeabilization involved in apoptotic signaling pathway	Biological Process	GO:1901028	5.60E-07
Wnt signaling pathway, calcium modulating pathway	Biological Process	GO:0007223	5.60E-07
positive regulation of ion transmembrane transport	Biological Process	GO:0034767	5.67E-07
positive regulation of B cell proliferation	Biological Process	GO:0030890	5.68E-07
negative regulation of G0 to G1 transition	Biological Process	GO:0070317	5.68E-07
positive regulation of lipid biosynthetic process	Biological Process	GO:0046889	5.68E-07
regulation of response to endoplasmic reticulum stress	Biological Process	GO:1905897	5.68E-07
response to axon injury	Biological Process	GO:0048678	5.77E-07
production of molecular mediator of immune response	Biological Process	GO:0002440	5.77E-07
viral entry into host cell	Biological Process	GO:0046718	5.82E-07
regulation of anion transport	Biological Process	GO:0044070	6.06E-07
regulation of lipid transport	Biological Process	GO:0032368	6.10E-07
regulation of cell proliferation involved in heart morphogenesis	Biological Process	GO:2000136	6.15E-07
regulation of protein polymerization	Biological Process	GO:0032271	6.31E-07
regulation of microtubule-based process	Biological Process	GO:0032886	6.31E-07
regulation of establishment of planar polarity	Biological Process	GO:0090175	6.42E-07
negative regulation of coagulation	Biological Process	GO:0050819	6.52E-07
positive regulation of response to cytokine stimulus	Biological Process	GO:0060760	6.52E-07
muscle organ morphogenesis	Biological Process	GO:0048644	6.53E-07
regulation of protein tyrosine kinase activity	Biological Process	GO:0061097	6.53E-07

positive regulation of innate immune response	Biological Process	GO:0045089	6.53E-07
regulation of interleukin-4 production	Biological Process	GO:0032673	6.53E-07
negative regulation of osteoclast differentiation	Biological Process	GO:0045671	6.53E-07
astrocyte activation	Biological Process	GO:0048143	6.53E-07
renal system vasculature development	Biological Process	GO:0061437	6.53E-07
kidney vasculature development	Biological Process	GO:0061440	6.53E-07
regulation of alcohol biosynthetic process	Biological Process	GO:1902930	6.81E-07
negative regulation of ERK1 and ERK2 cascade	Biological Process	GO:0070373	6.83E-07
protein polymerization	Biological Process	GO:0051258	7.16E-07
ear morphogenesis	Biological Process	GO:0042471	7.21E-07
neutrophil migration	Biological Process	GO:1990266	7.21E-07
positive regulation of cytokine production involved in immune response	Biological Process	GO:0002720	7.25E-07
collagen fibril organization	Biological Process	GO:0030199	7.25E-07
negative regulation of reactive oxygen species metabolic process	Biological Process	GO:2000378	7.54E-07
thyroid gland development	Biological Process	GO:0030878	7.54E-07
regulation of protein homodimerization activity	Biological Process	GO:0043496	7.54E-07
uterus development	Biological Process	GO:0060065	7.54E-07
lung epithelial cell differentiation	Biological Process	GO:0060487	7.54E-07
regulation of branching involved in ureteric bud morphogenesis	Biological Process	GO:0090189	7.54E-07
extracellular vesicle biogenesis	Biological Process	GO:0140112	7.54E-07
negative regulation of protein acetylation	Biological Process	GO:1901984	7.54E-07
negative regulation of cellular senescence	Biological Process	GO:2000773	7.54E-07
protein insertion into mitochondrial membrane involved in apoptotic signaling pathway	Biological Process	GO:0001844	7.93E-07
positive regulation of histone acetylation	Biological Process	GO:0035066	7.93E-07
positive regulation of CD4-positive, alpha-beta T cell differentiation	Biological Process	GO:0043372	7.93E-07
myeloid leukocyte cytokine production	Biological Process	GO:0061082	7.93E-07
liver regeneration	Biological Process	GO:0097421	7.93E-07
beta-catenin-TCF complex assembly	Biological Process	GO:1904837	7.93E-07
heart field specification	Biological Process	GO:0003128	7.94E-07
response to laminar fluid shear stress	Biological Process	GO:0034616	7.94E-07
embryo implantation	Biological Process	GO:0007566	7.94E-07
negative regulation of osteoblast differentiation	Biological Process	GO:0045668	7.94E-07
negative regulation of leukocyte proliferation	Biological Process	GO:0070664	8.11E-07
megakaryocyte differentiation	Biological Process	GO:0030219	8.13E-07
lactation	Biological Process	GO:0007595	8.64E-07
Rho protein signal transduction	Biological Process	GO:0007266	8.70E-07

negative regulation of chromosome organization	Biological Process	GO:2001251	8.72E-07
regulation of cytokinesis	Biological Process	GO:0032465	8.92E-07
cardiac muscle cell development	Biological Process	GO:0055013	8.92E-07
response to isoquinoline alkaloid	Biological Process	GO:0014072	8.92E-07
positive regulation of BMP signaling pathway	Biological Process	GO:0030513	8.92E-07
interleukin-4 production	Biological Process	GO:0032633	8.92E-07
response to morphine	Biological Process	GO:0043278	8.92E-07
regulation of neuroblast proliferation	Biological Process	GO:1902692	8.92E-07
positive regulation of hormone biosynthetic process	Biological Process	GO:0046886	9.05E-07
negative regulation of pri-miRNA transcription by RNA polymerase II	Biological Process	GO:1902894	9.31E-07
regulation of release of sequestered calcium ion into cytosol	Biological Process	GO:0051279	9.45E-07
muscle tissue morphogenesis	Biological Process	GO:0060415	9.45E-07
macrophage derived foam cell differentiation	Biological Process	GO:0010742	9.50E-07
positive regulation of interleukin-10 production	Biological Process	GO:0032733	9.50E-07
foam cell differentiation	Biological Process	GO:0090077	9.50E-07
cholesterol biosynthetic process	Biological Process	GO:0006695	9.66E-07
positive regulation of muscle cell apoptotic process	Biological Process	GO:0010661	9.68E-07
regulation of intracellular estrogen receptor signaling pathway	Biological Process	GO:0033146	9.68E-07
alcohol metabolic process	Biological Process	GO:0006066	1.03E-06
Wnt signaling pathway, planar cell polarity pathway	Biological Process	GO:0060071	1.04E-06
regulation of membrane potential	Biological Process	GO:0042391	1.04E-06
mRNA transcription	Biological Process	GO:0009299	1.06E-06
positive regulation of osteoclast differentiation	Biological Process	GO:0045672	1.06E-06
positive regulation of positive chemotaxis	Biological Process	GO:0050927	1.06E-06
regulation of striated muscle contraction	Biological Process	GO:0006942	1.10E-06
negative regulation of cellular protein localization	Biological Process	GO:1903828	1.10E-06
response to activity	Biological Process	GO:0014823	1.10E-06
positive regulation of interferon-gamma production	Biological Process	GO:0032729	1.10E-06
T cell migration	Biological Process	GO:0072678	1.10E-06
regulation of receptor internalization	Biological Process	GO:0002090	1.11E-06
type B pancreatic cell development	Biological Process	GO:0003323	1.15E-06
positive regulation of transforming growth factor beta production	Biological Process	GO:0071636	1.15E-06
extracellular exosome biogenesis	Biological Process	GO:0097734	1.15E-06
negative regulation of oxidative stress-induced neuron death	Biological Process	GO:1903204	1.15E-06
divalent inorganic cation transport	Biological Process	GO:0072511	1.16E-06
actin cytoskeleton reorganization	Biological Process	GO:0031532	1.19E-06

renal system process	Biological Process	GO:0003014	1.21E-06
regulation of ubiquitin-protein transferase activity	Biological Process	GO:0051438	1.24E-06
positive regulation of heart growth	Biological Process	GO:0060421	1.24E-06
regulation of substrate adhesion-dependent cell spreading	Biological Process	GO:1900024	1.24E-06
response to nerve growth factor	Biological Process	GO:1990089	1.24E-06
organelle fusion	Biological Process	GO:0048284	1.25E-06
gastrulation with mouth forming second	Biological Process	GO:0001702	1.33E-06
endochondral ossification	Biological Process	GO:0001958	1.33E-06
replacement ossification	Biological Process	GO:0036075	1.33E-06
regulation of reproductive process	Biological Process	GO:2000241	1.36E-06
cardiac muscle tissue morphogenesis	Biological Process	GO:0055008	1.36E-06
secondary alcohol biosynthetic process	Biological Process	GO:1902653	1.37E-06
purine nucleotide metabolic process	Biological Process	GO:0006163	1.43E-06
inner ear morphogenesis	Biological Process	GO:0042472	1.47E-06
regulation of calcium-mediated signaling	Biological Process	GO:0050848	1.47E-06
neuron projection regeneration	Biological Process	GO:0031102	1.48E-06
membrane protein proteolysis	Biological Process	GO:0033619	1.48E-06
positive regulation of erythrocyte differentiation	Biological Process	GO:0045648	1.52E-06
negative regulation of intrinsic apoptotic signaling pathway in response to DNA damage	Biological Process	GO:1902230	1.52E-06
regulation of sister chromatid segregation	Biological Process	GO:0033045	1.59E-06
positive regulation of nitric oxide biosynthetic process	Biological Process	GO:0045429	1.59E-06
positive regulation of protein acetylation	Biological Process	GO:1901985	1.59E-06
regulation of DNA damage response, signal transduction by p53 class mediator	Biological Process	GO:0043516	1.64E-06
response to estrogen	Biological Process	GO:0043627	1.64E-06
regulation of dendrite morphogenesis	Biological Process	GO:0048814	1.65E-06
negative regulation of DNA replication	Biological Process	GO:0008156	1.66E-06
regulation of translation	Biological Process	GO:0006417	1.66E-06
peptidyl-tyrosine autophosphorylation	Biological Process	GO:0038083	1.68E-06
cellular response to topologically incorrect protein	Biological Process	GO:0035967	1.68E-06
T cell mediated immunity	Biological Process	GO:0002456	1.69E-06
immunoglobulin production involved in immunoglobulin mediated immune response	Biological Process	GO:0002381	1.69E-06
negative regulation of oxidative stress-induced cell death	Biological Process	GO:1903202	1.69E-06
regulation of generation of precursor metabolites and energy	Biological Process	GO:0043467	1.70E-06
fatty acid metabolic process	Biological Process	GO:0006631	1.72E-06
metanephric tubule development	Biological Process	GO:0072170	1.72E-06
regulation of B cell apoptotic process	Biological Process	GO:0002902	1.73E-06

cell proliferation involved in heart morphogenesis	Biological Process	GO:0061323	1.73E-06
renal vesicle morphogenesis	Biological Process	GO:0072077	1.73E-06
regulation of blood pressure	Biological Process	GO:0008217	1.86E-06
protein maturation	Biological Process	GO:0051604	1.91E-06
phospholipid metabolic process	Biological Process	GO:0006644	1.97E-06
neuron projection organization	Biological Process	GO:0106027	2.02E-06
positive regulation of protein polymerization	Biological Process	GO:0032273	2.14E-06
positive regulation of cytokine-mediated signaling pathway	Biological Process	GO:0001961	2.16E-06
negative regulation of stress-activated MAPK cascade	Biological Process	GO:0032873	2.16E-06
negative regulation of stress-activated protein kinase signaling cascade	Biological Process	GO:0070303	2.16E-06
response to interleukin-6	Biological Process	GO:0070741	2.16E-06
regulation of protein targeting	Biological Process	GO:1903533	2.20E-06
nuclear DNA replication	Biological Process	GO:0033260	2.20E-06
histone H3 acetylation	Biological Process	GO:0043966	2.20E-06
cellular response to nutrient	Biological Process	GO:0031670	2.32E-06
positive regulation of DNA biosynthetic process	Biological Process	GO:2000573	2.33E-06
purine-containing compound metabolic process	Biological Process	GO:0072521	2.34E-06
regulation of protein autophosphorylation	Biological Process	GO:0031952	2.39E-06
negative regulation of leukocyte apoptotic process	Biological Process	GO:2000107	2.39E-06
regulation of epidermis development	Biological Process	GO:0045682	2.48E-06
neutrophil homeostasis	Biological Process	GO:0001780	2.50E-06
chemokine biosynthetic process	Biological Process	GO:0042033	2.50E-06
chemokine metabolic process	Biological Process	GO:0050755	2.50E-06
ventricular cardiac muscle cell differentiation	Biological Process	GO:0055012	2.50E-06
negative regulation of gene silencing by miRNA	Biological Process	GO:0060965	2.50E-06
positive regulation of amyloid-beta formation	Biological Process	GO:1902004	2.50E-06
negative regulation of cellular response to oxidative stress	Biological Process	GO:1900408	2.55E-06
positive regulation of reactive oxygen species biosynthetic process	Biological Process	GO:1903428	2.55E-06
positive regulation of nitric oxide metabolic process	Biological Process	GO:1904407	2.60E-06
positive regulation of vascular endothelial growth factor production	Biological Process	GO:0010575	2.60E-06
negative regulation of lymphocyte apoptotic process	Biological Process	GO:0070229	2.60E-06
divalent metal ion transport	Biological Process	GO:0070838	2.60E-06
negative regulation of defense response	Biological Process	GO:0031348	2.64E-06
sequestering of calcium ion	Biological Process	GO:0051208	2.66E-06
somatic diversification of immunoglobulins	Biological Process	GO:0016445	2.75E-06
regulation of viral transcription	Biological Process	GO:0046782	2.75E-06

positive regulation of sprouting angiogenesis	Biological Process	GO:1903672	2.75E-06
skeletal muscle tissue regeneration	Biological Process	GO:0043403	2.77E-06
positive regulation of chondrocyte differentiation	Biological Process	GO:0032332	2.77E-06
long-term memory	Biological Process	GO:0007616	2.82E-06
vasodilation	Biological Process	GO:0042311	2.82E-06
regulation of hormone metabolic process	Biological Process	GO:0032350	2.87E-06
interleukin-17 production	Biological Process	GO:0032620	2.87E-06
negative regulation of alpha-beta T cell activation	Biological Process	GO:0046636	2.87E-06
cardiac atrium development	Biological Process	GO:0003230	2.90E-06
positive regulation of mitochondrial outer membrane permeabilization involved in apoptotic signaling pathway	Biological Process	GO:1901030	2.90E-06
regulation of ERBB signaling pathway	Biological Process	GO:1901184	2.97E-06
lipid localization	Biological Process	GO:0010876	2.99E-06
lamellipodium organization	Biological Process	GO:0097581	3.01E-06
positive regulation of lipid transport	Biological Process	GO:0032370	3.23E-06
heterotypic cell-cell adhesion	Biological Process	GO:0034113	3.23E-06
establishment of planar polarity	Biological Process	GO:0001736	3.28E-06
establishment of tissue polarity	Biological Process	GO:0007164	3.28E-06
release of sequestered calcium ion into cytosol	Biological Process	GO:0051209	3.28E-06
regulation of calcium ion transmembrane transport	Biological Process	GO:1903169	3.31E-06
positive regulation of macroautophagy	Biological Process	GO:0016239	3.31E-06
skeletal muscle cell differentiation	Biological Process	GO:0035914	3.31E-06
regulation of sequestering of calcium ion	Biological Process	GO:0051282	3.32E-06
positive regulation of cation transmembrane transport	Biological Process	GO:1904064	3.36E-06
female gamete generation	Biological Process	GO:0007292	3.37E-06
positive regulation of macrophage differentiation	Biological Process	GO:0045651	3.39E-06
positive regulation of extrinsic apoptotic signaling pathway via death domain receptors	Biological Process	GO:1902043	3.39E-06
mitochondrial transport	Biological Process	GO:0006839	3.40E-06
regulation of cell projection assembly	Biological Process	GO:0060491	3.54E-06
positive regulation of carbohydrate metabolic process	Biological Process	GO:0045913	3.67E-06
endocardial cushion formation	Biological Process	GO:0003272	3.70E-06
negative regulation of alpha-beta T cell differentiation	Biological Process	GO:0046639	3.70E-06
cortical cytoskeleton organization	Biological Process	GO:0030865	3.72E-06
ventricular cardiac muscle tissue morphogenesis	Biological Process	GO:0055010	3.72E-06
positive regulation of cardiac muscle tissue growth	Biological Process	GO:0055023	3.72E-06
histone H3-K9 modification	Biological Process	GO:0061647	3.72E-06

monocyte chemotaxis	Biological Process	GO:0002548	3.95E-06
regulation of transcription from RNA polymerase II promoter in response to stress	Biological Process	GO:0043618	4.10E-06
regulation of ATP metabolic process	Biological Process	GO:1903578	4.10E-06
negative regulation of gliogenesis	Biological Process	GO:0014014	4.13E-06
response to progesterone	Biological Process	GO:0032570	4.13E-06
intrinsic apoptotic signaling pathway in response to DNA damage by p53 class mediator	Biological Process	GO:0042771	4.13E-06
positive regulation of chemokine biosynthetic process	Biological Process	GO:0045080	4.19E-06
positive regulation of SMAD protein signal transduction	Biological Process	GO:0060391	4.19E-06
leukocyte aggregation	Biological Process	GO:0070486	4.19E-06
commissural neuron axon guidance	Biological Process	GO:0071679	4.19E-06
cellular response to heat	Biological Process	GO:0034605	4.23E-06
mitral valve morphogenesis	Biological Process	GO:0003183	4.36E-06
ovarian follicle development	Biological Process	GO:0001541	4.36E-06
response to cocaine	Biological Process	GO:0042220	4.36E-06
regulation of macrophage activation	Biological Process	GO:0043030	4.36E-06
positive regulation of protein tyrosine kinase activity	Biological Process	GO:0061098	4.36E-06
negative regulation of platelet activation	Biological Process	GO:0010544	4.36E-06
mesenchymal to epithelial transition	Biological Process	GO:0060231	4.36E-06
trachea development	Biological Process	GO:0060438	4.36E-06
renal vesicle development	Biological Process	GO:0072087	4.36E-06
exosomal secretion	Biological Process	GO:1990182	4.36E-06
regulation of hormone biosynthetic process	Biological Process	GO:0046885	4.38E-06
metanephric epithelium development	Biological Process	GO:0072207	4.38E-06
negative regulation of cardiocyte differentiation	Biological Process	GO:1905208	4.38E-06
membrane protein ectodomain proteolysis	Biological Process	GO:0006509	4.47E-06
stem cell division	Biological Process	GO:0017145	4.47E-06
regulation of autophagy of mitochondrion	Biological Process	GO:1903146	4.47E-06
defense response to virus	Biological Process	GO:0051607	4.51E-06
lymphocyte homeostasis	Biological Process	GO:0002260	4.65E-06
spinal cord development	Biological Process	GO:0021510	4.65E-06
activation of JUN kinase activity	Biological Process	GO:0007257	4.77E-06
regulation of oligodendrocyte differentiation	Biological Process	GO:0048713	4.77E-06
regulation of type 2 immune response	Biological Process	GO:0002828	4.80E-06
miRNA metabolic process	Biological Process	GO:0010586	4.80E-06
positive regulation of release of cytochrome c from mitochondria	Biological Process	GO:0090200	4.80E-06
smooth muscle contraction	Biological Process	GO:0006939	4.82E-06

monocyte differentiation	Biological Process	GO:0030224	4.94E-06
regulation of vascular endothelial growth factor receptor signaling pathway	Biological Process	GO:0030947	4.94E-06
smooth muscle cell apoptotic process	Biological Process	GO:0034390	4.94E-06
regulation of smooth muscle cell apoptotic process	Biological Process	GO:0034391	4.94E-06
regulation of superoxide metabolic process	Biological Process	GO:0090322	4.94E-06
mononuclear cell differentiation	Biological Process	GO:1903131	4.94E-06
regulation of NMDA receptor activity	Biological Process	GO:2000310	4.94E-06
positive regulation of CD4-positive, alpha-beta T cell activation	Biological Process	GO:2000516	4.94E-06
myeloid cell apoptotic process	Biological Process	GO:0033028	4.96E-06
protein processing	Biological Process	GO:0016485	4.96E-06
regulation of exocytosis	Biological Process	GO:0017157	4.96E-06
positive regulation of interleukin-1 beta production	Biological Process	GO:0032731	4.97E-06
carboxylic acid biosynthetic process	Biological Process	GO:0046394	5.09E-06
negative regulation of sequestering of calcium ion	Biological Process	GO:0051283	5.20E-06
phagocytosis	Biological Process	GO:0006909	5.27E-06
negative regulation of cellular amide metabolic process	Biological Process	GO:0034249	5.29E-06
regulation of plasma membrane bounded cell projection assembly	Biological Process	GO:0120032	5.31E-06
negative regulation of blood vessel morphogenesis	Biological Process	GO:2000181	5.31E-06
negative regulation of mononuclear cell proliferation	Biological Process	GO:0032945	5.34E-06
negative regulation of lymphocyte proliferation	Biological Process	GO:0050672	5.34E-06
positive regulation of interleukin-1 production	Biological Process	GO:0032732	5.44E-06
positive regulation of blood vessel diameter	Biological Process	GO:0097755	5.44E-06
negative regulation of response to oxidative stress	Biological Process	GO:1902883	5.44E-06
protein homooligomerization	Biological Process	GO:0051260	5.46E-06
negative regulation of autophagy	Biological Process	GO:0010507	5.46E-06
glial cell migration	Biological Process	GO:0008347	5.66E-06
organic acid biosynthetic process	Biological Process	GO:0016053	5.78E-06
chemokine-mediated signaling pathway	Biological Process	GO:0070098	5.97E-06
embryonic heart tube development	Biological Process	GO:0035050	6.05E-06
positive regulation of neuroblast proliferation	Biological Process	GO:0002052	6.05E-06
regulation of SMAD protein signal transduction	Biological Process	GO:0060390	6.05E-06
DNA-dependent DNA replication	Biological Process	GO:0006261	6.23E-06
lipid homeostasis	Biological Process	GO:0055088	6.23E-06
ER-nucleus signaling pathway	Biological Process	GO:0006984	6.38E-06
labyrinthine layer development	Biological Process	GO:0060711	6.38E-06
cellular response to interleukin-6	Biological Process	GO:0071354	6.38E-06

regulation of gene silencing	Biological Process	GO:0060968	6.49E-06
microtubule cytoskeleton organization involved in mitosis	Biological Process	GO:1902850	6.56E-06
positive regulation of interleukin-17 production	Biological Process	GO:0032740	6.67E-06
thymocyte apoptotic process	Biological Process	GO:0070242	6.67E-06
midbrain dopaminergic neuron differentiation	Biological Process	GO:1904948	6.67E-06
negative regulation of morphogenesis of an epithelium	Biological Process	GO:1905331	6.67E-06
sterol biosynthetic process	Biological Process	GO:0016126	6.71E-06
extracellular matrix disassembly	Biological Process	GO:0022617	6.71E-06
negative regulation of response to DNA damage stimulus	Biological Process	GO:2001021	6.71E-06
positive regulation of interleukin-6 production	Biological Process	GO:0032755	6.79E-06
negative regulation of cell-matrix adhesion	Biological Process	GO:0001953	7.08E-06
cardiac muscle cell contraction	Biological Process	GO:0086003	7.37E-06
positive regulation of dendrite development	Biological Process	GO:1900006	7.37E-06
positive regulation of response to wounding	Biological Process	GO:1903036	7.37E-06
regulation of homotypic cell-cell adhesion	Biological Process	GO:0034110	7.37E-06
modulation by symbiont of host cellular process	Biological Process	GO:0044068	7.37E-06
glomerulus vasculature development	Biological Process	GO:0072012	7.37E-06
cell differentiation involved in metanephros development	Biological Process	GO:0072202	7.37E-06
positive regulation of mitotic nuclear division	Biological Process	GO:0045840	7.41E-06
negative regulation of ERBB signaling pathway	Biological Process	GO:1901185	7.41E-06
T cell cytokine production	Biological Process	GO:0002369	7.70E-06
neural tube patterning	Biological Process	GO:0021532	7.70E-06
modulation by symbiont of host process	Biological Process	GO:0044003	7.70E-06
response to interleukin-7	Biological Process	GO:0098760	7.70E-06
cellular response to interleukin-7	Biological Process	GO:0098761	7.70E-06
embryonic heart tube morphogenesis	Biological Process	GO:0003143	7.77E-06
positive regulation of epithelial cell differentiation	Biological Process	GO:0030858	7.77E-06
myotube differentiation	Biological Process	GO:0014902	7.86E-06
negative regulation of angiogenesis	Biological Process	GO:0016525	8.01E-06
negative regulation of transmembrane transport	Biological Process	GO:0034763	8.19E-06
response to increased oxygen levels	Biological Process	GO:0036296	8.19E-06
hyaluronan metabolic process	Biological Process	GO:0030212	8.22E-06
regulation of vascular endothelial growth factor production	Biological Process	GO:0010574	8.55E-06
regulation of interleukin-17 production	Biological Process	GO:0032660	8.55E-06
regulation of T cell cytokine production	Biological Process	GO:0002724	8.55E-06
response to X-ray	Biological Process	GO:0010165	8.55E-06

regulation of platelet activation	Biological Process	GO:0010543	8.55E-06
wound healing, spreading of cells	Biological Process	GO:0044319	8.55E-06
epiboly involved in wound healing	Biological Process	GO:0090505	8.55E-06
negative regulation of cell morphogenesis involved in differentiation	Biological Process	GO:0010771	8.86E-06
positive regulation of response to DNA damage stimulus	Biological Process	GO:2001022	8.86E-06
neural crest cell migration	Biological Process	GO:0001755	9.29E-06
regulation of glutamate receptor signaling pathway	Biological Process	GO:1900449	9.36E-06
learning	Biological Process	GO:0007612	9.76E-06
regulation of epidermal growth factor receptor signaling pathway	Biological Process	GO:0042058	9.76E-06
regulation of peptidyl-threonine phosphorylation	Biological Process	GO:0010799	9.76E-06
collagen catabolic process	Biological Process	GO:0030574	9.76E-06
developmental pigmentation	Biological Process	GO:0048066	9.76E-06
negative regulation of cold-induced thermogenesis	Biological Process	GO:0120163	9.76E-06
regulation of p38MAPK cascade	Biological Process	GO:1900744	9.76E-06
cellular response to monoamine stimulus	Biological Process	GO:0071868	9.81E-06
cellular response to catecholamine stimulus	Biological Process	GO:0071870	9.81E-06
cardiac right ventricle morphogenesis	Biological Process	GO:0003215	9.84E-06
positive regulation of smooth muscle cell apoptotic process	Biological Process	GO:0034393	9.84E-06
negative regulation of CD4-positive, alpha-beta T cell differentiation	Biological Process	GO:0043371	9.84E-06
tongue development	Biological Process	GO:0043586	9.84E-06
pericardium development	Biological Process	GO:0060039	9.84E-06
positive regulation of amyloid precursor protein catabolic process	Biological Process	GO:1902993	9.84E-06
entry of bacterium into host cell	Biological Process	GO:0035635	9.86E-06
regulation of chemokine biosynthetic process	Biological Process	GO:0045073	9.86E-06
hepatocyte differentiation	Biological Process	GO:0070365	9.86E-06
epithelial cell fate commitment	Biological Process	GO:0072148	9.86E-06
nephron tubule epithelial cell differentiation	Biological Process	GO:0072160	9.86E-06
response to monoamine	Biological Process	GO:0071867	9.95E-06
response to catecholamine	Biological Process	GO:0071869	9.95E-06
regulation of posttranscriptional gene silencing	Biological Process	GO:0060147	1.01E-05
regulation of gene silencing by RNA	Biological Process	GO:0060966	1.01E-05
cellular response to light stimulus	Biological Process	GO:0071482	1.02E-05
hematopoietic progenitor cell differentiation	Biological Process	GO:0002244	1.03E-05
negative regulation of immune response	Biological Process	GO:0050777	1.17E-05
lipid export from cell	Biological Process	GO:0140353	1.23E-05
regulation of response to drug	Biological Process	GO:2001023	1.23E-05

negative regulation of tumor necrosis factor superfamily cytokine production	Biological Process	GO:1903556	1.23E-05
positive regulation of nucleotide biosynthetic process	Biological Process	GO:0030810	1.24E-05
negative regulation of interleukin-2 production	Biological Process	GO:0032703	1.24E-05
positive regulation of interleukin-4 production	Biological Process	GO:0032753	1.24E-05
neuronal stem cell population maintenance	Biological Process	GO:0097150	1.24E-05
positive regulation of purine nucleotide biosynthetic process	Biological Process	GO:1900373	1.24E-05
regulation of non-canonical Wnt signaling pathway	Biological Process	GO:2000050	1.24E-05
negative regulation of cardiac muscle cell differentiation	Biological Process	GO:2000726	1.24E-05
tumor necrosis factor-mediated signaling pathway	Biological Process	GO:0033209	1.25E-05
axon regeneration	Biological Process	GO:0031103	1.27E-05
regulation of B cell activation	Biological Process	GO:0050864	1.27E-05
positive regulation of establishment of protein localization to mitochondrion	Biological Process	GO:1903749	1.33E-05
regulation of cation channel activity	Biological Process	GO:2001257	1.33E-05
positive regulation of cardiac muscle cell proliferation	Biological Process	GO:0060045	1.34E-05
negative regulation of cellular response to insulin stimulus	Biological Process	GO:1900077	1.34E-05
positive regulation of epithelial cell apoptotic process	Biological Process	GO:1904037	1.34E-05
positive regulation of response to endoplasmic reticulum stress	Biological Process	GO:1905898	1.34E-05
membrane depolarization	Biological Process	GO:0051899	1.37E-05
skin development	Biological Process	GO:0043588	1.37E-05
myelination in peripheral nervous system	Biological Process	GO:0022011	1.40E-05
peripheral nervous system axon ensheathment	Biological Process	GO:0032292	1.40E-05
negative regulation of viral transcription	Biological Process	GO:0032897	1.40E-05
positive regulation of collagen biosynthetic process	Biological Process	GO:0032967	1.40E-05
regulation of heterotypic cell-cell adhesion	Biological Process	GO:0034114	1.40E-05
pharyngeal system development	Biological Process	GO:0060037	1.40E-05
cell differentiation involved in embryonic placenta development	Biological Process	GO:0060706	1.40E-05
regulation of protein insertion into mitochondrial membrane involved in apoptotic signaling pathway	Biological Process	GO:1900739	1.40E-05
positive regulation of protein insertion into mitochondrial membrane involved in apoptotic signaling pathway	Biological Process	GO:1900740	1.40E-05
positive regulation of receptor biosynthetic process	Biological Process	GO:0010870	1.40E-05
replicative senescence	Biological Process	GO:0090399	1.40E-05
positive regulation of ATP biosynthetic process	Biological Process	GO:2001171	1.40E-05
negative regulation of production of molecular mediator of immune response	Biological Process	GO:0002701	1.42E-05
negative regulation of intracellular steroid hormone receptor signaling pathway	Biological Process	GO:0033144	1.42E-05
negative regulation of epidermal growth factor receptor signaling pathway	Biological Process	GO:0042059	1.46E-05

positive regulation of transforming growth factor beta receptor signaling pathway	Biological Process	GO:0030511	1.46E-05
positive regulation of microtubule polymerization	Biological Process	GO:0031116	1.46E-05
regulation of myeloid cell apoptotic process	Biological Process	GO:0033032	1.46E-05
epidermis morphogenesis	Biological Process	GO:0048730	1.46E-05
positive regulation of cellular response to transforming growth factor beta stimulus	Biological Process	GO:1903846	1.46E-05
regulation of stem cell population maintenance	Biological Process	GO:2000036	1.46E-05
negative regulation of CD4-positive, alpha-beta T cell activation	Biological Process	GO:2000515	1.46E-05
response to amphetamine	Biological Process	GO:0001975	1.46E-05
regulation of regulatory T cell differentiation	Biological Process	GO:0045589	1.46E-05
placenta blood vessel development	Biological Process	GO:0060674	1.46E-05
regulation of neuron projection regeneration	Biological Process	GO:0070570	1.46E-05
epiboly	Biological Process	GO:0090504	1.46E-05
dendritic spine organization	Biological Process	GO:0097061	1.47E-05
negative regulation of small molecule metabolic process	Biological Process	GO:0062014	1.47E-05
histone methylation	Biological Process	GO:0016571	1.51E-05
negative regulation of ion transmembrane transport	Biological Process	GO:0034766	1.51E-05
negative regulation of lymphocyte differentiation	Biological Process	GO:0045620	1.57E-05
negative regulation of interleukin-10 production	Biological Process	GO:0032693	1.57E-05
mammary gland alveolus development	Biological Process	GO:0060749	1.57E-05
mammary gland lobule development	Biological Process	GO:0061377	1.57E-05
craniofacial suture morphogenesis	Biological Process	GO:0097094	1.57E-05
regulation of cell-cell adhesion mediated by cadherin	Biological Process	GO:2000047	1.57E-05
negative regulation of peptidyl-lysine acetylation	Biological Process	GO:2000757	1.57E-05
regulation of organelle assembly	Biological Process	GO:1902115	1.62E-05
glycerolipid biosynthetic process	Biological Process	GO:0045017	1.66E-05
negative regulation of T cell differentiation	Biological Process	GO:0045581	1.67E-05
positive regulation of exocytosis	Biological Process	GO:0045921	1.68E-05
negative regulation of protein modification by small protein conjugation or removal	Biological Process	GO:1903321	1.68E-05
epidermis development	Biological Process	GO:0008544	1.78E-05
positive regulation of nuclear division	Biological Process	GO:0051785	1.80E-05
adult locomotory behavior	Biological Process	GO:0008344	1.81E-05
mitral valve development	Biological Process	GO:0003174	1.81E-05
histone-serine phosphorylation	Biological Process	GO:0035404	1.81E-05
prostate glandular acinus development	Biological Process	GO:0060525	1.81E-05
histone H4 deacetylation	Biological Process	GO:0070933	1.81E-05
metanephric tubule morphogenesis	Biological Process	GO:0072173	1.81E-05

positive regulation of cysteine-type endopeptidase activity involved in apoptotic signaling pathway	Biological Process	GO:2001269	1.81E-05
regulation of mRNA catabolic process	Biological Process	GO:0061013	1.82E-05
cholesterol homeostasis	Biological Process	GO:0042632	1.83E-05
autophagosome organization	Biological Process	GO:1905037	1.83E-05
negative regulation of Ras protein signal transduction	Biological Process	GO:0046580	1.84E-05
positive regulation of cardiac muscle hypertrophy	Biological Process	GO:0010613	1.88E-05
glutamate receptor signaling pathway	Biological Process	GO:0007215	1.89E-05
acute inflammatory response	Biological Process	GO:0002526	1.96E-05
purine ribonucleotide metabolic process	Biological Process	GO:0009150	2.00E-05
regulation of T cell mediated immunity	Biological Process	GO:0002709	2.02E-05
pulmonary valve development	Biological Process	GO:0003177	2.06E-05
atrial septum development	Biological Process	GO:0003283	2.06E-05
cellular response to platelet-derived growth factor stimulus	Biological Process	GO:0036120	2.06E-05
positive regulation of mesonephros development	Biological Process	GO:0061213	2.06E-05
glomerular epithelium development	Biological Process	GO:0072010	2.06E-05
regulation of skeletal muscle cell differentiation	Biological Process	GO:2001014	2.06E-05
purine nucleoside triphosphate metabolic process	Biological Process	GO:0009144	2.08E-05
response to testosterone	Biological Process	GO:0033574	2.10E-05
neurotrophin signaling pathway	Biological Process	GO:0038179	2.10E-05
positive regulation of DNA recombination	Biological Process	GO:0045911	2.10E-05
regulation of viral life cycle	Biological Process	GO:1903900	2.14E-05
forebrain neuron differentiation	Biological Process	GO:0021879	2.15E-05
negative regulation of GTPase activity	Biological Process	GO:0034260	2.15E-05
programmed necrotic cell death	Biological Process	GO:0097300	2.15E-05
regulation of interleukin-8 production	Biological Process	GO:0032677	2.22E-05
regulation of dendritic spine development	Biological Process	GO:0060998	2.22E-05
cardiac muscle cell action potential	Biological Process	GO:0086001	2.22E-05
regulation of dephosphorylation	Biological Process	GO:0035303	2.25E-05
morphogenesis of a polarized epithelium	Biological Process	GO:0001738	2.26E-05
autophagosome assembly	Biological Process	GO:0000045	2.28E-05
regulation of pattern recognition receptor signaling pathway	Biological Process	GO:0062207	2.28E-05
MyD88-dependent toll-like receptor signaling pathway	Biological Process	GO:0002755	2.29E-05
regulation of cell fate commitment	Biological Process	GO:0010453	2.29E-05
vascular endothelial growth factor production	Biological Process	GO:0010573	2.29E-05
positive regulation of DNA replication	Biological Process	GO:0045740	2.29E-05

negative regulation of insulin receptor signaling pathway	Biological Process	GO:0046627	2.29E-05
regulation of cellular carbohydrate metabolic process	Biological Process	GO:0010675	2.31E-05
telomere maintenance	Biological Process	GO:0000723	2.32E-05
ribose phosphate metabolic process	Biological Process	GO:0019693	2.34E-05
sterol homeostasis	Biological Process	GO:0055092	2.35E-05
positive regulation of receptor internalization	Biological Process	GO:0002092	2.36E-05
positive regulation of activated T cell proliferation	Biological Process	GO:0042104	2.36E-05
decidualization	Biological Process	GO:0046697	2.36E-05
positive regulation of mononuclear cell migration	Biological Process	GO:0071677	2.36E-05
purine-containing compound biosynthetic process	Biological Process	GO:0072522	2.37E-05
telomere organization	Biological Process	GO:0032200	2.44E-05
MyD88-independent toll-like receptor signaling pathway	Biological Process	GO:0002756	2.44E-05
positive regulation of microtubule polymerization or depolymerization	Biological Process	GO:0031112	2.44E-05
positive regulation of transcription from RNA polymerase II promoter in response to stress	Biological Process	GO:0036003	2.44E-05
regulation of isotype switching	Biological Process	GO:0045191	2.44E-05
positive regulation of ubiquitin-protein transferase activity	Biological Process	GO:0051443	2.44E-05
negative regulation of signal transduction by p53 class mediator	Biological Process	GO:1901797	2.44E-05
carboxylic acid transport	Biological Process	GO:0046942	2.44E-05
positive regulation of muscle cell differentiation	Biological Process	GO:0051149	2.46E-05
regulation of gene silencing by miRNA	Biological Process	GO:0060964	2.47E-05
cell-cell adhesion mediated by integrin	Biological Process	GO:0033631	2.47E-05
positive regulation of heterotypic cell-cell adhesion	Biological Process	GO:0034116	2.47E-05
myelin maintenance	Biological Process	GO:0043217	2.47E-05
morphogenesis of an epithelial bud	Biological Process	GO:0060572	2.47E-05
ganglion development	Biological Process	GO:0061548	2.47E-05
cellular response to nitric oxide	Biological Process	GO:0071732	2.47E-05
positive regulation of collagen metabolic process	Biological Process	GO:0010714	2.50E-05
response to immobilization stress	Biological Process	GO:0035902	2.50E-05
collateral sprouting	Biological Process	GO:0048668	2.50E-05
regulation of oxidative stress-induced intrinsic apoptotic signaling pathway	Biological Process	GO:1902175	2.50E-05
positive regulation of DNA-templated transcription, initiation	Biological Process	GO:2000144	2.50E-05
cardiac atrium morphogenesis	Biological Process	GO:0003209	2.50E-05
regulation of macrophage derived foam cell differentiation	Biological Process	GO:0010743	2.50E-05
positive regulation of steroid metabolic process	Biological Process	GO:0045940	2.50E-05
homeostasis of number of cells within a tissue	Biological Process	GO:0048873	2.50E-05

negative regulation of cartilage development	Biological Process	GO:0061037	2.50E-05
interleukin-8 production	Biological Process	GO:0032637	2.54E-05
pyruvate metabolic process	Biological Process	GO:0006090	2.56E-05
positive regulation of cell cycle G1/S phase transition	Biological Process	GO:1902808	2.59E-05
immune response-activating cell surface receptor signaling pathway	Biological Process	GO:0002429	2.68E-05
immune response-activating signal transduction	Biological Process	GO:0002757	2.68E-05
maintenance of protein location	Biological Process	GO:0045185	2.81E-05
positive regulation of muscle hypertrophy	Biological Process	GO:0014742	2.84E-05
T-helper 1 type immune response	Biological Process	GO:0042088	2.84E-05
positive regulation of glial cell differentiation	Biological Process	GO:0045687	2.84E-05
response to electrical stimulus	Biological Process	GO:0051602	2.84E-05
regulation of cellular response to drug	Biological Process	GO:2001038	2.84E-05
Rab protein signal transduction	Biological Process	GO:0032482	2.95E-05
vesicle budding from membrane	Biological Process	GO:0006900	3.04E-05
organic acid transport	Biological Process	GO:0015849	3.10E-05
positive regulation of G1/S transition of mitotic cell cycle	Biological Process	GO:1900087	3.12E-05
nerve development	Biological Process	GO:0021675	3.16E-05
regulation of cellular response to heat	Biological Process	GO:1900034	3.16E-05
negative regulation of mRNA metabolic process	Biological Process	GO:1903312	3.16E-05
positive regulation of heart contraction	Biological Process	GO:0045823	3.23E-05
regulation of protein maturation	Biological Process	GO:1903317	3.33E-05
protein localization to chromosome	Biological Process	GO:0034502	3.36E-05
mesodermal cell fate commitment	Biological Process	GO:0001710	3.39E-05
osteoblast development	Biological Process	GO:0002076	3.39E-05
alpha-beta T cell lineage commitment	Biological Process	GO:0002363	3.39E-05
chronic inflammatory response	Biological Process	GO:0002544	3.39E-05
positive regulation of T cell cytokine production	Biological Process	GO:0002726	3.39E-05
positive regulation of vascular endothelial growth factor receptor signaling pathway	Biological Process	GO:0030949	3.39E-05
response to hepatocyte growth factor	Biological Process	GO:0035728	3.39E-05
mRNA transcription by RNA polymerase II	Biological Process	GO:0042789	3.39E-05
positive regulation of glial cell proliferation	Biological Process	GO:0060252	3.39E-05
response to nitric oxide	Biological Process	GO:0071731	3.39E-05
kidney mesenchyme development	Biological Process	GO:0072074	3.39E-05
regulation of epithelial cell differentiation involved in kidney development	Biological Process	GO:2000696	3.39E-05
regulation of mRNA stability	Biological Process	GO:0043488	3.41E-05
positive regulation of protein import into nucleus	Biological Process	GO:0042307	3.60E-05

histone H3-K9 methylation	Biological Process	GO:0051567	3.60E-05
cellular response to cadmium ion	Biological Process	GO:0071276	3.60E-05
positive regulation of substrate adhesion-dependent cell spreading	Biological Process	GO:1900026	3.60E-05
regulation of DNA-templated transcription, initiation	Biological Process	GO:2000142	3.60E-05
regulation of actin cytoskeleton reorganization	Biological Process	GO:2000249	3.60E-05
regulation of epidermal cell differentiation	Biological Process	GO:0045604	3.60E-05
positive regulation of wound healing	Biological Process	GO:0090303	3.60E-05
positive regulation of viral life cycle	Biological Process	GO:1903902	3.60E-05
ribonucleotide metabolic process	Biological Process	GO:0009259	3.60E-05
glycerolipid metabolic process	Biological Process	GO:0046486	3.60E-05
negative regulation of synaptic transmission	Biological Process	GO:0050805	3.62E-05
regulation of mitochondrial membrane potential	Biological Process	GO:0051881	3.62E-05
cartilage development involved in endochondral bone morphogenesis	Biological Process	GO:0060351	3.64E-05
establishment of protein localization to mitochondrial membrane	Biological Process	GO:0090151	3.64E-05
regulation of lipid catabolic process	Biological Process	GO:0050994	3.74E-05
response to type I interferon	Biological Process	GO:0034340	3.78E-05
glial cell fate commitment	Biological Process	GO:0021781	3.78E-05
platelet-derived growth factor receptor-beta signaling pathway	Biological Process	GO:0035791	3.78E-05
phosphatidylinositol-3-phosphate biosynthetic process	Biological Process	GO:0036092	3.78E-05
T-helper 2 cell differentiation	Biological Process	GO:0045064	3.78E-05
positive regulation of regulatory T cell differentiation	Biological Process	GO:0045591	3.78E-05
regulation of keratinocyte migration	Biological Process	GO:0051547	3.78E-05
positive regulation of metanephros development	Biological Process	GO:0072216	3.78E-05
regulation of cell proliferation involved in kidney development	Biological Process	GO:1901722	3.78E-05
regulation of purine nucleotide metabolic process	Biological Process	GO:1900542	3.80E-05
vasoconstriction	Biological Process	GO:0042310	3.89E-05
activation of protein kinase B activity	Biological Process	GO:0032148	3.92E-05
tumor necrosis factor biosynthetic process	Biological Process	GO:0042533	3.92E-05
regulation of tumor necrosis factor biosynthetic process	Biological Process	GO:0042534	3.92E-05
regulatory T cell differentiation	Biological Process	GO:0045066	3.92E-05
positive regulation of pattern recognition receptor signaling pathway	Biological Process	GO:0062208	3.92E-05
inflammatory cell apoptotic process	Biological Process	GO:0006925	3.97E-05
response to platelet-derived growth factor	Biological Process	GO:0036119	3.97E-05
positive regulation of T-helper cell differentiation	Biological Process	GO:0045624	3.97E-05
immunoglobulin secretion	Biological Process	GO:0048305	3.97E-05
cellular response to low-density lipoprotein particle stimulus	Biological Process	GO:0071404	3.97E-05

positive regulation of protein deacetylation	Biological Process	GO:0090312	3.97E-05
adult behavior	Biological Process	GO:0030534	3.97E-05
response to prostaglandin	Biological Process	GO:0034694	4.18E-05
regulation of cellular respiration	Biological Process	GO:0043457	4.18E-05
regulation of astrocyte differentiation	Biological Process	GO:0048710	4.18E-05
lens fiber cell differentiation	Biological Process	GO:0070306	4.18E-05
positive regulation of tissue remodeling	Biological Process	GO:0034105	4.22E-05
regulation of endothelial cell differentiation	Biological Process	GO:0045601	4.22E-05
lymph vessel development	Biological Process	GO:0001945	4.26E-05
negative regulation of cytokine production involved in immune response	Biological Process	GO:0002719	4.26E-05
removal of superoxide radicals	Biological Process	GO:0019430	4.26E-05
hair follicle morphogenesis	Biological Process	GO:0031069	4.26E-05
interleukin-13 production	Biological Process	GO:0032616	4.26E-05
positive regulation of isotype switching	Biological Process	GO:0045830	4.26E-05
morphogenesis of an epithelial fold	Biological Process	GO:0060571	4.26E-05
negative regulation of DNA-dependent DNA replication	Biological Process	GO:2000104	4.26E-05
negative regulation of blood vessel diameter	Biological Process	GO:0097756	4.30E-05
T cell chemotaxis	Biological Process	GO:0010818	4.31E-05
regulation of interferon-alpha production	Biological Process	GO:0032647	4.31E-05
negative regulation of peptidyl-serine phosphorylation	Biological Process	GO:0033137	4.31E-05
positive regulation of dephosphorylation	Biological Process	GO:0035306	4.31E-05
negative regulation of small GTPase mediated signal transduction	Biological Process	GO:0051058	4.31E-05
negative regulation of translation	Biological Process	GO:0017148	4.34E-05
synaptic vesicle recycling	Biological Process	GO:0036465	4.41E-05
cochlea development	Biological Process	GO:0090102	4.42E-05
regulation of G2/M transition of mitotic cell cycle	Biological Process	GO:0010389	4.44E-05
execution phase of apoptosis	Biological Process	GO:0097194	4.45E-05
negative regulation of immune effector process	Biological Process	GO:0002698	4.62E-05
regulation of fatty acid metabolic process	Biological Process	GO:0019217	4.67E-05
positive regulation of peptide hormone secretion	Biological Process	GO:0090277	4.67E-05
purine nucleotide biosynthetic process	Biological Process	GO:0006164	4.71E-05
protein methylation	Biological Process	GO:0006479	4.71E-05
protein alkylation	Biological Process	GO:0008213	4.71E-05
interleukin-1-mediated signaling pathway	Biological Process	GO:0070498	4.72E-05
organelle membrane fusion	Biological Process	GO:0090174	4.72E-05
regulation of neuroinflammatory response	Biological Process	GO:0150077	4.84E-05

necrotic cell death	Biological Process	GO:0070265	4.89E-05
regulation of chromosome separation	Biological Process	GO:1905818	4.89E-05
regulation of action potential	Biological Process	GO:0098900	5.23E-05
positive regulation of T cell mediated immunity	Biological Process	GO:0002711	5.25E-05
regulation of interferon-beta production	Biological Process	GO:0032648	5.25E-05
regulation of smooth muscle cell differentiation	Biological Process	GO:0051150	5.25E-05
regulation of telomerase activity	Biological Process	GO:0051972	5.25E-05
positive regulation of mRNA catabolic process	Biological Process	GO:0061014	5.25E-05
lipid transport	Biological Process	GO:0006869	5.37E-05
acidic amino acid transport	Biological Process	GO:0015800	5.43E-05
regulation of protein processing	Biological Process	GO:0070613	5.43E-05
nucleotide biosynthetic process	Biological Process	GO:0009165	5.50E-05
hippo signaling	Biological Process	GO:0035329	5.50E-05
negative regulation of insulin secretion	Biological Process	GO:0046676	5.50E-05
apoptotic process involved in development	Biological Process	GO:1902742	5.50E-05
pulmonary valve morphogenesis	Biological Process	GO:0003184	5.50E-05
epithelial to mesenchymal transition involved in endocardial cushion formation	Biological Process	GO:0003198	5.50E-05
heat generation	Biological Process	GO:0031649	5.50E-05
regulation of mammary gland epithelial cell proliferation	Biological Process	GO:0033599	5.50E-05
chondrocyte proliferation	Biological Process	GO:0035988	5.50E-05
cytoplasmic sequestering of transcription factor	Biological Process	GO:0042994	5.50E-05
mammary gland epithelial cell differentiation	Biological Process	GO:0060644	5.50E-05
retina vasculature development in camera-type eye	Biological Process	GO:0061298	5.50E-05
renal filtration cell differentiation	Biological Process	GO:0061318	5.50E-05
glomerular visceral epithelial cell differentiation	Biological Process	GO:0072112	5.50E-05
regulation of early endosome to late endosome transport	Biological Process	GO:2000641	5.50E-05
regulation of cysteine-type endopeptidase activity involved in apoptotic signaling pathway	Biological Process	GO:2001267	5.50E-05
mesenchymal to epithelial transition involved in metanephros morphogenesis	Biological Process	GO:0003337	5.52E-05
mitotic nuclear envelope disassembly	Biological Process	GO:0007077	5.52E-05
regulation of cell fate specification	Biological Process	GO:0042659	5.52E-05
positive regulation of keratinocyte migration	Biological Process	GO:0051549	5.52E-05
epithelial cell differentiation involved in prostate gland development	Biological Process	GO:0060742	5.52E-05
cellular response to X-ray	Biological Process	GO:0071481	5.52E-05
hepatocyte apoptotic process	Biological Process	GO:0097284	5.52E-05
regulation of nucleotide metabolic process	Biological Process	GO:0006140	5.76E-05

stimulatory C-type lectin receptor signaling pathway	Biological Process	GO:0002223	5.84E-05
negative regulation of tumor necrosis factor production	Biological Process	GO:0032720	5.86E-05
regulation of synaptic transmission, glutamatergic	Biological Process	GO:0051966	5.86E-05
nucleoside triphosphate metabolic process	Biological Process	GO:0009141	5.90E-05
positive regulation of cell junction assembly	Biological Process	GO:1901890	5.92E-05
plasma membrane organization	Biological Process	GO:0007009	5.92E-05
negative regulation of muscle cell apoptotic process	Biological Process	GO:0010656	5.92E-05
activation of innate immune response	Biological Process	GO:0002218	5.93E-05
receptor catabolic process	Biological Process	GO:0032801	6.13E-05
cellular response to alkaloid	Biological Process	GO:0071312	6.13E-05
negative regulation of response to drug	Biological Process	GO:2001024	6.13E-05
protein insertion into mitochondrial membrane	Biological Process	GO:0051204	6.13E-05
regulation of long-term synaptic potentiation	Biological Process	GO:1900271	6.13E-05
regulation of phospholipase C activity	Biological Process	GO:1900274	6.13E-05
negative regulation of BMP signaling pathway	Biological Process	GO:0030514	6.23E-05
negative regulation of hormone secretion	Biological Process	GO:0046888	6.62E-05
regulation of lymphocyte migration	Biological Process	GO:2000401	6.62E-05
lymphoid progenitor cell differentiation	Biological Process	GO:0002320	6.69E-05
response to muramyl dipeptide	Biological Process	GO:0032495	6.69E-05
response to hydroperoxide	Biological Process	GO:0033194	6.69E-05
CD4-positive or CD8-positive, alpha-beta T cell lineage commitment	Biological Process	GO:0043369	6.69E-05
smooth muscle tissue development	Biological Process	GO:0048745	6.69E-05
metanephric nephron tubule development	Biological Process	GO:0072234	6.69E-05
negative regulation of cardiac muscle tissue growth	Biological Process	GO:0055022	6.72E-05
face morphogenesis	Biological Process	GO:0060325	6.72E-05
negative regulation of heart growth	Biological Process	GO:0061117	6.72E-05
cellular response to interleukin-4	Biological Process	GO:0071353	6.72E-05
regulation of endoplasmic reticulum stress-induced intrinsic apoptotic signaling pathway	Biological Process	GO:1902235	6.72E-05
regulation of cell migration involved in sprouting angiogenesis	Biological Process	GO:0090049	6.87E-05
regulation of DNA repair	Biological Process	GO:0006282	6.92E-05
natural killer cell activation	Biological Process	GO:0030101	7.10E-05
positive regulation of immunoglobulin production	Biological Process	GO:0002639	7.16E-05
regulation of gastrulation	Biological Process	GO:0010470	7.16E-05
regulation of myelination	Biological Process	GO:0031641	7.16E-05
dendritic cell differentiation	Biological Process	GO:0097028	7.16E-05
regulation of response to reactive oxygen species	Biological Process	GO:1901031	7.16E-05

Schwann cell development	Biological Process	GO:0014044	7.18E-05
regulation of histone deacetylation	Biological Process	GO:0031063	7.18E-05
TRIF-dependent toll-like receptor signaling pathway	Biological Process	GO:0035666	7.18E-05
epithelial tube branching involved in lung morphogenesis	Biological Process	GO:0060441	7.18E-05
forebrain cell migration	Biological Process	GO:0021885	7.18E-05
negative regulation of chondrocyte differentiation	Biological Process	GO:0032331	7.28E-05
low-density lipoprotein receptor particle metabolic process	Biological Process	GO:0032799	7.28E-05
angiogenesis involved in wound healing	Biological Process	GO:0060055	7.28E-05
branching involved in salivary gland morphogenesis	Biological Process	GO:0060445	7.28E-05
vesicle fusion	Biological Process	GO:0006906	7.33E-05
lysosomal transport	Biological Process	GO:0007041	7.35E-05
pigmentation	Biological Process	GO:0043473	7.37E-05
positive regulation of protein autophosphorylation	Biological Process	GO:0031954	7.38E-05
embryonic placenta morphogenesis	Biological Process	GO:0060669	7.38E-05
regulation of androgen receptor signaling pathway	Biological Process	GO:0060765	7.38E-05
T-helper 17 cell differentiation	Biological Process	GO:0072539	7.38E-05
regulation of anion transmembrane transport	Biological Process	GO:1903959	7.38E-05
regulation of collagen metabolic process	Biological Process	GO:0010712	7.40E-05
positive regulation of interleukin-8 production	Biological Process	GO:0032757	7.40E-05
negative regulation of I-kappaB kinase/NF-kappaB signaling	Biological Process	GO:0043124	7.40E-05
cellular response to interleukin-12	Biological Process	GO:0071349	7.40E-05
negative regulation of transcription by competitive promoter binding	Biological Process	GO:0010944	7.54E-05
regulation of glial cell apoptotic process	Biological Process	GO:0034350	7.54E-05
cellular response to iron ion	Biological Process	GO:0071281	7.54E-05
cell proliferation involved in metanephros development	Biological Process	GO:0072203	7.54E-05
positive regulation of small GTPase mediated signal transduction	Biological Process	GO:0051057	7.69E-05
regulation of cell cycle G2/M phase transition	Biological Process	GO:1902749	7.75E-05
regulation of intrinsic apoptotic signaling pathway in response to DNA damage	Biological Process	GO:1902229	8.25E-05
positive regulation of protein import	Biological Process	GO:1904591	8.25E-05
organic anion transport	Biological Process	GO:0015711	8.51E-05
telencephalon cell migration	Biological Process	GO:0022029	8.70E-05
collagen biosynthetic process	Biological Process	GO:0032964	8.70E-05
neuroepithelial cell differentiation	Biological Process	GO:0060563	8.70E-05
cellular response to calcium ion	Biological Process	GO:0071277	8.83E-05
response to amine	Biological Process	GO:0014075	8.85E-05
regulation of mononuclear cell migration	Biological Process	GO:0071675	8.85E-05

calcineurin-mediated signaling	Biological Process	GO:0097720	8.85E-05
lamellipodium assembly	Biological Process	GO:0030032	8.85E-05
IRE1-mediated unfolded protein response	Biological Process	GO:0036498	8.85E-05
lymphocyte chemotaxis	Biological Process	GO:0048247	8.85E-05
regulation of RNA stability	Biological Process	GO:0043487	8.85E-05
endodermal cell fate commitment	Biological Process	GO:0001711	8.85E-05
pre-miRNA processing	Biological Process	GO:0031054	8.85E-05
negative regulation of histone acetylation	Biological Process	GO:0035067	8.85E-05
negative regulation of DNA damage response, signal transduction by p53 class mediator	Biological Process	GO:0043518	8.85E-05
aldehyde biosynthetic process	Biological Process	GO:0046184	8.85E-05
post-embryonic animal organ development	Biological Process	GO:0048569	8.85E-05
metanephric renal vesicle morphogenesis	Biological Process	GO:0072283	8.85E-05
nucleoside phosphate biosynthetic process	Biological Process	GO:1901293	8.92E-05
type I interferon signaling pathway	Biological Process	GO:0060337	9.20E-05
cellular response to type I interferon	Biological Process	GO:0071357	9.20E-05
hindlimb morphogenesis	Biological Process	GO:0035137	9.39E-05
regulation of fibroblast growth factor receptor signaling pathway	Biological Process	GO:0040036	9.39E-05
pigment cell differentiation	Biological Process	GO:0050931	9.39E-05
cellular response to estradiol stimulus	Biological Process	GO:0071392	9.39E-05
regulation of mitotic sister chromatid segregation	Biological Process	GO:0033047	9.53E-05
negative regulation of protein-containing complex assembly	Biological Process	GO:0031333	9.55E-05
DNA recombination	Biological Process	GO:0006310	9.81E-05
regulation of mitotic sister chromatid separation	Biological Process	GO:0010965	9.83E-05
protein heterooligomerization	Biological Process	GO:0051291	9.89E-05
DNA alkylation	Biological Process	GO:0006305	0.000101
DNA methylation	Biological Process	GO:0006306	0.000101
positive regulation of phospholipase C activity	Biological Process	GO:0010863	0.000104
regulation of cyclase activity	Biological Process	GO:0031279	0.000104
calcineurin-NFAT signaling cascade	Biological Process	GO:0033173	0.000104
positive regulation of axon extension	Biological Process	GO:0045773	0.000104
adipose tissue development	Biological Process	GO:0060612	0.000104
regulation of cardiac muscle cell contraction	Biological Process	GO:0086004	0.000104
interferon-beta production	Biological Process	GO:0032608	0.000104
regulation of cholesterol biosynthetic process	Biological Process	GO:0045540	0.000104
response to interleukin-12	Biological Process	GO:0070671	0.000104
regulation of sterol biosynthetic process	Biological Process	GO:0106118	0.000104

negative regulation of protein processing	Biological Process	GO:0010955	0.000105
granulocyte differentiation	Biological Process	GO:0030851	0.000105
neurotrophin TRK receptor signaling pathway	Biological Process	GO:0048011	0.000105
negative regulation of protein maturation	Biological Process	GO:1903318	0.000105
alternative mRNA splicing, via spliceosome	Biological Process	GO:0000380	0.000106
innate immune response activating cell surface receptor signaling pathway	Biological Process	GO:0002220	0.000108
heart looping	Biological Process	GO:0001947	0.000108
regulation of tumor necrosis factor-mediated signaling pathway	Biological Process	GO:0010803	0.000108
positive regulation of intrinsic apoptotic signaling pathway	Biological Process	GO:2001244	0.000108
regulation of glucose metabolic process	Biological Process	GO:0010906	0.00011
regulation of synaptic vesicle cycle	Biological Process	GO:0098693	0.00011
lipid modification	Biological Process	GO:0030258	0.00011
chromosome segregation	Biological Process	GO:0007059	0.000111
endocrine process	Biological Process	GO:0050886	0.000112
regulation of gene expression by genetic imprinting	Biological Process	GO:0006349	0.000112
cholesterol storage	Biological Process	GO:0010878	0.000112
positive regulation of histone deacetylation	Biological Process	GO:0031065	0.000112
DNA damage response, signal transduction resulting in transcription	Biological Process	GO:0042772	0.000112
coronary vasculature morphogenesis	Biological Process	GO:0060977	0.000112
glomerular epithelial cell differentiation	Biological Process	GO:0072311	0.000112
positive regulation of branching involved in ureteric bud morphogenesis	Biological Process	GO:0090190	0.000112
regulation of epithelial tube formation	Biological Process	GO:1905276	0.000112
sister chromatid segregation	Biological Process	GO:0000819	0.000113
digestive system process	Biological Process	GO:0022600	0.000114
action potential	Biological Process	GO:0001508	0.000114
B cell homeostasis	Biological Process	GO:0001782	0.000115
interferon-alpha production	Biological Process	GO:0032607	0.000115
positive regulation of interferon-beta production	Biological Process	GO:0032728	0.000115
negative regulation of B cell activation	Biological Process	GO:0050869	0.000115
mRNA destabilization	Biological Process	GO:0061157	0.000115
vacuole organization	Biological Process	GO:0007033	0.000116
metaphase/anaphase transition of mitotic cell cycle	Biological Process	GO:0007091	0.000119
regulation of DNA-dependent DNA replication	Biological Process	GO:0090329	0.000119
cellular response to osmotic stress	Biological Process	GO:0071470	0.000121
semaphorin-plexin signaling pathway	Biological Process	GO:0071526	0.000121
regulation of purine nucleotide biosynthetic process	Biological Process	GO:1900371	0.000121

positive regulation of skeletal muscle tissue development	Biological Process	GO:0048643	0.000124
cellular response to oxygen radical	Biological Process	GO:0071450	0.000124
cellular response to superoxide	Biological Process	GO:0071451	0.000124
positive regulation of steroid biosynthetic process	Biological Process	GO:0010893	0.000124
maintenance of gastrointestinal epithelium	Biological Process	GO:0030277	0.000124
retinal ganglion cell axon guidance	Biological Process	GO:0031290	0.000124
embryonic hemopoiesis	Biological Process	GO:0035162	0.000124
muscle cell cellular homeostasis	Biological Process	GO:0046716	0.000124
eosinophil chemotaxis	Biological Process	GO:0048245	0.000124
positive regulation of response to oxidative stress	Biological Process	GO:1902884	0.000124
negative regulation of axonogenesis	Biological Process	GO:0050771	0.000124
endocrine hormone secretion	Biological Process	GO:0060986	0.000124
regulation of actin filament-based movement	Biological Process	GO:1903115	0.000124
positive regulation of lipid catabolic process	Biological Process	GO:0050996	0.000127
cochlea morphogenesis	Biological Process	GO:0090103	0.000127
modification of synaptic structure	Biological Process	GO:0099563	0.000127
regulation of cytoplasmic transport	Biological Process	GO:1903649	0.000127
negative regulation of neural precursor cell proliferation	Biological Process	GO:2000178	0.000127
innate immune response-activating signal transduction	Biological Process	GO:0002758	0.00013
regulation of vasoconstriction	Biological Process	GO:0019229	0.000132
purine ribonucleoside triphosphate metabolic process	Biological Process	GO:0009205	0.000136
negative regulation of protein ubiquitination	Biological Process	GO:0031397	0.000136
protein-containing complex disassembly	Biological Process	GO:0032984	0.000138
regulation of telomere maintenance	Biological Process	GO:0032204	0.000139
endocardium development	Biological Process	GO:0003157	0.00014
SMAD protein complex assembly	Biological Process	GO:0007183	0.00014
regulation of skeletal muscle satellite cell proliferation	Biological Process	GO:0014842	0.00014
positive regulation of granulocyte macrophage colony-stimulating factor production	Biological Process	GO:0032725	0.00014
regulation of histone phosphorylation	Biological Process	GO:0033127	0.00014
isotype switching to IgG isotypes	Biological Process	GO:0048291	0.00014
regulation of timing of cell differentiation	Biological Process	GO:0048505	0.00014
eyelid development in camera-type eye	Biological Process	GO:0061029	0.00014
nucleotide-binding oligomerization domain containing 2 signaling pathway	Biological Process	GO:0070431	0.00014
regulation of nephron tubule epithelial cell differentiation	Biological Process	GO:0072182	0.00014
regulation of polysaccharide biosynthetic process	Biological Process	GO:0032885	0.00014
head morphogenesis	Biological Process	GO:0060323	0.00014

positive regulation of lymphocyte migration	Biological Process	GO:2000403	0.00014
adaptive immune response based on somatic recombination of immune receptors built from immunoglobulin superfamily domains	Biological Process	GO:0002460	0.000141
regulation of sodium ion transport	Biological Process	GO:0002028	0.000141
positive regulation of nucleocytoplasmic transport	Biological Process	GO:0046824	0.000144
positive regulation of telomere maintenance	Biological Process	GO:0032206	0.000144
oligodendrocyte development	Biological Process	GO:0014003	0.000148
regulation of collagen biosynthetic process	Biological Process	GO:0032965	0.000148
regulation of filopodium assembly	Biological Process	GO:0051489	0.000148
necroptotic process	Biological Process	GO:0070266	0.000148
regulation of granulocyte chemotaxis	Biological Process	GO:0071622	0.000148
Fc-epsilon receptor signaling pathway	Biological Process	GO:0038095	0.000151
regulation of protein dephosphorylation	Biological Process	GO:0035304	0.000157
positive T cell selection	Biological Process	GO:0043368	0.000161
embryonic digestive tract development	Biological Process	GO:0048566	0.000161
negative regulation of gene silencing	Biological Process	GO:0060969	0.000161
response to interleukin-4	Biological Process	GO:0070670	0.000161
positive regulation of signaling receptor activity	Biological Process	GO:2000273	0.000161
inositol phosphate-mediated signaling	Biological Process	GO:0048016	0.000162
regulation of toll-like receptor signaling pathway	Biological Process	GO:0034121	0.000162
histone lysine methylation	Biological Process	GO:0034968	0.000163
regulation of neurotransmitter receptor activity	Biological Process	GO:0099601	0.000169
mitotic spindle organization	Biological Process	GO:0007052	0.000172
monocarboxylic acid biosynthetic process	Biological Process	GO:0072330	0.000174
hindbrain morphogenesis	Biological Process	GO:0021575	0.000177
regulation of nucleotide biosynthetic process	Biological Process	GO:0030808	0.000177
brown fat cell differentiation	Biological Process	GO:0050873	0.000177
regulation of cell killing	Biological Process	GO:0031341	0.000177
regulation of postsynapse organization	Biological Process	GO:0099175	0.000177
regulation of organic acid transport	Biological Process	GO:0032890	0.000178
negative regulation of T cell proliferation	Biological Process	GO:0042130	0.000178
negative regulation of reproductive process	Biological Process	GO:2000242	0.000178
positive regulation of translation	Biological Process	GO:0045727	0.000178
DNA methylation or demethylation	Biological Process	GO:0044728	0.000179
modulation by virus of host process	Biological Process	GO:0019048	0.000182
osteoblast proliferation	Biological Process	GO:0033687	0.000182

endothelial cell chemotaxis	Biological Process	GO:0035767	0.000182
positive regulation of type 2 immune response	Biological Process	GO:0002830	0.000187
regulation of cholesterol storage	Biological Process	GO:0010885	0.000187
dentate gyrus development	Biological Process	GO:0021542	0.000187
regulation of synaptic transmission, dopaminergic	Biological Process	GO:0032225	0.000187
atrial septum morphogenesis	Biological Process	GO:0060413	0.000187
3'-UTR-mediated mRNA destabilization	Biological Process	GO:0061158	0.000187
glomerular mesangium development	Biological Process	GO:0072109	0.000187
nephric duct development	Biological Process	GO:0072176	0.000187
regulation of exosomal secretion	Biological Process	GO:1903541	0.000187
amyloid fibril formation	Biological Process	GO:1990000	0.000187
regulation of cholesterol metabolic process	Biological Process	GO:0090181	0.000191
negative regulation of mitotic nuclear division	Biological Process	GO:0045839	0.000198
negative regulation of mRNA catabolic process	Biological Process	GO:1902373	0.000198
regulation of osteoblast proliferation	Biological Process	GO:0033688	2.00E-04
negative regulation of glial cell differentiation	Biological Process	GO:0045686	2.00E-04
regulation of axon regeneration	Biological Process	GO:0048679	2.00E-04
positive regulation of lamellipodium organization	Biological Process	GO:1902745	2.00E-04
response to angiotensin	Biological Process	GO:1990776	2.00E-04
T cell receptor signaling pathway	Biological Process	GO:0050852	0.000203
negative regulation of JNK cascade	Biological Process	GO:0046329	0.000208
positive regulation of neurotransmitter transport	Biological Process	GO:0051590	0.000208
regulation of heart rate by cardiac conduction	Biological Process	GO:0086091	0.000208
leukocyte mediated cytotoxicity	Biological Process	GO:0001909	0.00021
blood vessel remodeling	Biological Process	GO:0001974	0.00021
negative regulation of peptide hormone secretion	Biological Process	GO:0090278	0.00021
regulation of lamellipodium organization	Biological Process	GO:1902743	0.00021
positive regulation of calcium ion transmembrane transport	Biological Process	GO:1904427	0.00021
positive regulation of superoxide anion generation	Biological Process	GO:0032930	0.000213
stress granule assembly	Biological Process	GO:0034063	0.000213
lymph vessel morphogenesis	Biological Process	GO:0036303	0.000213
keratinocyte migration	Biological Process	GO:0051546	0.000213
liver morphogenesis	Biological Process	GO:0072576	0.000213
negative regulation of release of cytochrome c from mitochondria	Biological Process	GO:0090201	0.000213
positive regulation of cellular response to oxidative stress	Biological Process	GO:1900409	0.000213
regulation of ATP biosynthetic process	Biological Process	GO:2001169	0.000213

central nervous system projection neuron axonogenesis	Biological Process	GO:0021952	0.000214
eosinophil migration	Biological Process	GO:0072677	0.000214
cardioblast proliferation	Biological Process	GO:0003263	0.000214
regulation of cardioblast proliferation	Biological Process	GO:0003264	0.000214
natural killer cell chemotaxis	Biological Process	GO:0035747	0.000214
regulation of T-helper 2 cell differentiation	Biological Process	GO:0045628	0.000214
trachea morphogenesis	Biological Process	GO:0060439	0.000214
prostate gland growth	Biological Process	GO:0060736	0.000214
epithelial cell proliferation involved in prostate gland development	Biological Process	GO:0060767	0.000214
cardiac neural crest cell differentiation involved in heart development	Biological Process	GO:0061307	0.000214
cardiac neural crest cell development involved in heart development	Biological Process	GO:0061308	0.000214
Notch signaling involved in heart development	Biological Process	GO:0061314	0.000214
glomerular mesangial cell proliferation	Biological Process	GO:0072110	0.000214
positive regulation of transcription from RNA polymerase II promoter in response to endoplasmic reticulum stress	Biological Process	GO:1990440	0.000214
metaphase/anaphase transition of cell cycle	Biological Process	GO:0044784	0.000217
regulation of sister chromatid cohesion	Biological Process	GO:0007063	0.000218
regulation of interleukin-13 production	Biological Process	GO:0032656	0.000218
positive regulation of interferon-alpha production	Biological Process	GO:0032727	0.000218
negative regulation of fibroblast growth factor receptor signaling pathway	Biological Process	GO:0040037	0.000218
vitamin D metabolic process	Biological Process	GO:0042359	0.000218
regulation of macrophage differentiation	Biological Process	GO:0045649	0.000218
negative regulation of response to biotic stimulus	Biological Process	GO:0002832	0.000219
regulation of viral genome replication	Biological Process	GO:0045069	0.000219
membrane fusion	Biological Process	GO:0061025	0.00022
interferon-gamma-mediated signaling pathway	Biological Process	GO:0060333	0.000221
synaptic vesicle exocytosis	Biological Process	GO:0016079	0.000229
import across plasma membrane	Biological Process	GO:0098739	0.000229
mitotic sister chromatid separation	Biological Process	GO:0051306	0.000235
negative regulation of nuclear division	Biological Process	GO:0051784	0.000235
purine ribonucleotide biosynthetic process	Biological Process	GO:0009152	0.000238
mast cell mediated immunity	Biological Process	GO:0002448	0.00024
response to nicotine	Biological Process	GO:0035094	0.00024
central nervous system neuron axonogenesis	Biological Process	GO:0021955	0.00024
positive regulation of transporter activity	Biological Process	GO:0032411	0.000246
positive regulation of Ras protein signal transduction	Biological Process	GO:0046579	0.000249

negative regulation of histone modification	Biological Process	GO:0031057	0.000251
response to copper ion	Biological Process	GO:0046688	0.000251
regulation of G protein-coupled receptor signaling pathway	Biological Process	GO:0008277	0.00026
positive regulation of plasma membrane bounded cell projection assembly	Biological Process	GO:0120034	0.000268
interleukin-1 beta secretion	Biological Process	GO:0050702	0.000268
negative regulation of peptidyl-tyrosine phosphorylation	Biological Process	GO:0050732	0.000268
regulation of natural killer cell activation	Biological Process	GO:0032814	0.000278
response to arsenic-containing substance	Biological Process	GO:0046685	0.000278
RNA destabilization	Biological Process	GO:0050779	0.000278
heart trabecula morphogenesis	Biological Process	GO:0061384	0.000278
cellular response to vitamin	Biological Process	GO:0071295	0.000278
negative regulation of lipid biosynthetic process	Biological Process	GO:0051055	0.000292
inflammatory response to antigenic stimulus	Biological Process	GO:0002437	0.000293
regulation of interleukin-1 beta secretion	Biological Process	GO:0050706	0.000293
negative regulation of inflammatory response	Biological Process	GO:0050728	0.000297
positive regulation of histone methylation	Biological Process	GO:0031062	0.000301
regulation of vascular permeability	Biological Process	GO:0043114	0.000301
membrane assembly	Biological Process	GO:0071709	0.000301
cellular extravasation	Biological Process	GO:0045123	0.000312
mesodermal cell fate specification	Biological Process	GO:0007501	0.000313
skeletal muscle satellite cell proliferation	Biological Process	GO:0014841	0.000313
regulation of skeletal muscle cell proliferation	Biological Process	GO:0014857	0.000313
hyaluronan biosynthetic process	Biological Process	GO:0030213	0.000313
regulation of development, heterochronic	Biological Process	GO:0040034	0.000313
regulation of MHC class II biosynthetic process	Biological Process	GO:0045346	0.000313
induction of positive chemotaxis	Biological Process	GO:0050930	0.000313
response to superoxide	Biological Process	GO:0000303	0.000314
positive regulation of peptidyl-threonine phosphorylation	Biological Process	GO:0010800	0.000314
cranial nerve morphogenesis	Biological Process	GO:0021602	0.000314
interleukin-6-mediated signaling pathway	Biological Process	GO:0070102	0.000314
T-helper 17 type immune response	Biological Process	GO:0072538	0.000314
negative regulation of protein localization to nucleus	Biological Process	GO:1900181	0.000314
immune response-regulating cell surface receptor signaling pathway involved in phagocytosis	Biological Process	GO:0002433	0.000325
Fc-gamma receptor signaling pathway involved in phagocytosis	Biological Process	GO:0038096	0.000325
regulation of smooth muscle contraction	Biological Process	GO:0006940	0.000326

response to cold	Biological Process	GO:0009409	0.000329
body morphogenesis	Biological Process	GO:0010171	0.000329
regulation of microtubule polymerization	Biological Process	GO:0031113	0.000329
phosphatidylinositol phosphorylation	Biological Process	GO:0046854	0.000329
synaptic transmission, glutamatergic	Biological Process	GO:0035249	0.000343
spindle organization	Biological Process	GO:0007051	0.000344
multicellular organismal response to stress	Biological Process	GO:0033555	0.000346
ventricular system development	Biological Process	GO:0021591	0.000348
regulation of receptor binding	Biological Process	GO:1900120	0.000348
regulation of polysaccharide metabolic process	Biological Process	GO:0032881	0.000356
embryonic axis specification	Biological Process	GO:0000578	0.000356
positive regulation of telomerase activity	Biological Process	GO:0051973	0.000356
positive regulation of calcium ion transport into cytosol	Biological Process	GO:0010524	0.000361
DNA damage response, signal transduction by p53 class mediator resulting in transcription of p21 class mediator	Biological Process	GO:0006978	0.000366
female genitalia development	Biological Process	GO:0030540	0.000366
regulation of nuclear cell cycle DNA replication	Biological Process	GO:0033262	0.000366
wound healing, spreading of epidermal cells	Biological Process	GO:0035313	0.000366
cellular response to hepatocyte growth factor stimulus	Biological Process	GO:0035729	0.000366
megakaryocyte development	Biological Process	GO:0035855	0.000366
establishment of planar polarity of embryonic epithelium	Biological Process	GO:0042249	0.000366
CD4-positive, alpha-beta T cell lineage commitment	Biological Process	GO:0043373	0.000366
mitotic cell cycle arrest	Biological Process	GO:0071850	0.000366
negative regulation of kidney development	Biological Process	GO:0090185	0.000366
positive regulation of oxidative stress-induced cell death	Biological Process	GO:1903209	0.000366
positive regulation of toll-like receptor signaling pathway	Biological Process	GO:0034123	0.000372
response to prostaglandin E	Biological Process	GO:0034695	0.000372
positive regulation of glycolytic process	Biological Process	GO:0045821	0.000372
regulation of membrane protein ectodomain proteolysis	Biological Process	GO:0051043	0.000372
negative regulation of neurotransmitter transport	Biological Process	GO:0051589	0.000372
inclusion body assembly	Biological Process	GO:0070841	0.000372
hematopoietic stem cell proliferation	Biological Process	GO:0071425	0.000372
metanephric nephron epithelium development	Biological Process	GO:0072243	0.000372
negative regulation of intrinsic apoptotic signaling pathway by p53 class mediator	Biological Process	GO:1902254	0.000372
vesicle localization	Biological Process	GO:0051648	0.000373
establishment of vesicle localization	Biological Process	GO:0051650	0.000374

cytokine secretion involved in immune response	Biological Process	GO:0002374	0.00038
cardioblast differentiation	Biological Process	GO:0010002	0.00038
response to manganese ion	Biological Process	GO:0010042	0.00038
positive regulation of lamellipodium assembly	Biological Process	GO:0010592	0.00038
signal transduction involved in regulation of gene expression	Biological Process	GO:0023019	0.00038
platelet formation	Biological Process	GO:0030220	0.00038
notochord development	Biological Process	GO:0030903	0.00038
positive regulation of natural killer cell activation	Biological Process	GO:0032816	0.00038
positive regulation of tumor necrosis factor biosynthetic process	Biological Process	GO:0042535	0.00038
negative regulation of endoplasmic reticulum stress-induced intrinsic apoptotic signaling pathway	Biological Process	GO:1902236	0.00038
dendritic spine morphogenesis	Biological Process	GO:0060997	0.000384
interleukin-12-mediated signaling pathway	Biological Process	GO:0035722	0.000402
mast cell degranulation	Biological Process	GO:0043303	0.000402
ribonucleoside diphosphate metabolic process	Biological Process	GO:0009185	0.000402
striated muscle cell apoptotic process	Biological Process	GO:0010658	0.000405
response to sterol	Biological Process	GO:0036314	0.000415
brain morphogenesis	Biological Process	GO:0048854	0.000415
regulation of T cell apoptotic process	Biological Process	GO:0070232	0.000415
Fc receptor mediated stimulatory signaling pathway	Biological Process	GO:0002431	0.000424
dorsal/ventral pattern formation	Biological Process	GO:0009953	0.000428
regulation of carbohydrate biosynthetic process	Biological Process	GO:0043255	0.000428
positive regulation of mRNA metabolic process	Biological Process	GO:1903313	0.00044
cellular carbohydrate metabolic process	Biological Process	GO:0044262	0.000442
regulation of B cell mediated immunity	Biological Process	GO:0002712	0.000444
regulation of immunoglobulin mediated immune response	Biological Process	GO:0002889	0.000444
carbohydrate biosynthetic process	Biological Process	GO:0016051	0.000452
response to oxygen radical	Biological Process	GO:0000305	0.000482
interleukin-7-mediated signaling pathway	Biological Process	GO:0038111	0.000482
modulation by host of viral process	Biological Process	GO:0044788	0.000482
regulation of fatty acid oxidation	Biological Process	GO:0046320	0.000482
negative regulation of fibroblast proliferation	Biological Process	GO:0048147	0.000482
positive regulation of filopodium assembly	Biological Process	GO:0051491	0.000482
positive regulation of protein targeting to membrane	Biological Process	GO:0090314	0.000482
phenol-containing compound metabolic process	Biological Process	GO:0018958	0.000485
regulation of axon guidance	Biological Process	GO:1902667	0.000493

fatty acid derivative biosynthetic process	Biological Process	GO:1901570	0.000502
regulation of striated muscle cell apoptotic process	Biological Process	GO:0010662	0.000507
filopodium assembly	Biological Process	GO:0046847	0.000507
positive regulation of B cell mediated immunity	Biological Process	GO:0002714	0.00051
positive regulation of immunoglobulin mediated immune response	Biological Process	GO:0002891	0.00051
negative regulation of smooth muscle cell migration	Biological Process	GO:0014912	0.00051
cerebellum morphogenesis	Biological Process	GO:0021587	0.00051
T cell homeostasis	Biological Process	GO:0043029	0.00051
positive regulation of fatty acid metabolic process	Biological Process	GO:0045923	0.00051
axon extension involved in axon guidance	Biological Process	GO:0048846	0.00051
positive regulation of cell migration involved in sprouting angiogenesis	Biological Process	GO:0090050	0.00051
neuron projection extension involved in neuron projection guidance	Biological Process	GO:1902284	0.00051
mast cell chemotaxis	Biological Process	GO:0002551	0.00051
negative regulation of B cell apoptotic process	Biological Process	GO:0002903	0.00051
programmed cell death involved in cell development	Biological Process	GO:0010623	0.00051
rostrocaudal neural tube patterning	Biological Process	GO:0021903	0.00051
hemidesmosome assembly	Biological Process	GO:0031581	0.00051
response to vitamin E	Biological Process	GO:0033197	0.00051
interleukin-2-mediated signaling pathway	Biological Process	GO:0038110	0.00051
negative regulation of protein import into nucleus	Biological Process	GO:0042308	0.00051
vitamin D biosynthetic process	Biological Process	GO:0042368	0.00051
regulation of isotype switching to IgG isotypes	Biological Process	GO:0048302	0.00051
peripheral nervous system neuron differentiation	Biological Process	GO:0048934	0.00051
peripheral nervous system neuron development	Biological Process	GO:0048935	0.00051
regulation of attachment of spindle microtubules to kinetochore	Biological Process	GO:0051988	0.00051
positive regulation of nuclear-transcribed mRNA poly(A) tail shortening	Biological Process	GO:0060213	0.00051
branching involved in prostate gland morphogenesis	Biological Process	GO:0060442	0.00051
embryonic camera-type eye formation	Biological Process	GO:0060900	0.00051
nephric duct morphogenesis	Biological Process	GO:0072178	0.00051
mast cell migration	Biological Process	GO:0097531	0.00051
regulation of apoptotic process involved in morphogenesis	Biological Process	GO:1902337	0.00051
negative regulation of protein import	Biological Process	GO:1904590	0.00051
Wnt signaling pathway involved in midbrain dopaminergic neuron differentiation	Biological Process	GO:1904953	0.00051
regulation of removal of superoxide radicals	Biological Process	GO:2000121	0.00051
protein localization to cell surface	Biological Process	GO:0034394	0.000523
Fc-gamma receptor signaling pathway	Biological Process	GO:0038094	0.000523

negative regulation of cation transmembrane transport	Biological Process	GO:1904063	0.000523
determination of heart left/right asymmetry	Biological Process	GO:0061371	0.000537
mitotic sister chromatid segregation	Biological Process	GO:0000070	0.000541
ribonucleoside triphosphate metabolic process	Biological Process	GO:0009199	0.000541
inactivation of MAPK activity	Biological Process	GO:0000188	0.000541
regulation of the force of heart contraction	Biological Process	GO:0002026	0.000541
substrate-dependent cell migration	Biological Process	GO:0006929	0.000541
regulation of epidermal growth factor-activated receptor activity	Biological Process	GO:0007176	0.000541
telomere maintenance via semi-conservative replication	Biological Process	GO:0032201	0.000541
positive regulation of macrophage activation	Biological Process	GO:0043032	0.000541
genetic imprinting	Biological Process	GO:0071514	0.000541
neurotransmitter reuptake	Biological Process	GO:0098810	0.000541
regulation of lymphocyte chemotaxis	Biological Process	GO:1901623	0.000541
regulation of fatty acid transport	Biological Process	GO:2000191	0.000541
chondrocyte development	Biological Process	GO:0002063	0.000541
mast cell activation involved in immune response	Biological Process	GO:0002279	0.000541
negative regulation of carbohydrate metabolic process	Biological Process	GO:0045912	0.000541
positive regulation of muscle contraction	Biological Process	GO:0045933	0.000541
regulation of microtubule polymerization or depolymerization	Biological Process	GO:0031110	0.000544
regulation of megakaryocyte differentiation	Biological Process	GO:0045652	0.000544
positive regulation of ion transmembrane transporter activity	Biological Process	GO:0032414	0.00058
regulation of neurotransmitter secretion	Biological Process	GO:0046928	0.00058
purine nucleoside diphosphate metabolic process	Biological Process	GO:0009135	0.000586
purine ribonucleoside diphosphate metabolic process	Biological Process	GO:0009179	0.000586
positive regulation of embryonic development	Biological Process	GO:0040019	0.000593
extracellular matrix assembly	Biological Process	GO:0085029	0.000593
vesicle-mediated transport between endosomal compartments	Biological Process	GO:0098927	0.000593
positive regulation of cardiocyte differentiation	Biological Process	GO:1905209	0.000593
ribose phosphate biosynthetic process	Biological Process	GO:0046390	0.000597
carbohydrate derivative catabolic process	Biological Process	GO:1901136	0.000597
regulation of platelet-derived growth factor receptor signaling pathway	Biological Process	GO:0010640	6.00E-04
dorsal/ventral neural tube patterning	Biological Process	GO:0021904	6.00E-04
microvillus organization	Biological Process	GO:0032528	6.00E-04
regulation of establishment or maintenance of cell polarity	Biological Process	GO:0032878	6.00E-04
cytoplasmic sequestering of protein	Biological Process	GO:0051220	6.00E-04
growth hormone receptor signaling pathway	Biological Process	GO:0060396	6.00E-04

positive regulation of cellular response to insulin stimulus	Biological Process	GO:1900078	6.00E-04
negative regulation of protein localization to cell periphery	Biological Process	GO:1904376	6.00E-04
positive regulation of vascular endothelial cell proliferation	Biological Process	GO:1905564	6.00E-04
regulation of endothelial cell chemotaxis	Biological Process	GO:2001026	6.00E-04
regulation of calcineurin-NFAT signaling cascade	Biological Process	GO:0070884	0.000601
regulation of calcineurin-mediated signaling	Biological Process	GO:0106056	0.000601
dicarboxylic acid transport	Biological Process	GO:0006835	0.000617
skeletal muscle cell proliferation	Biological Process	GO:0014856	0.000617
sequestering of triglyceride	Biological Process	GO:0030730	0.000617
regulation of granulocyte macrophage colony-stimulating factor production	Biological Process	GO:0032645	0.000617
positive regulation of interleukin-13 production	Biological Process	GO:0032736	0.000617
MHC class II biosynthetic process	Biological Process	GO:0045342	0.000617
positive regulation of interleukin-6 biosynthetic process	Biological Process	GO:0045410	0.000617
positive regulation of fatty acid oxidation	Biological Process	GO:0046321	0.000617
neuroblast division	Biological Process	GO:0055057	0.000617
convergent extension	Biological Process	GO:0060026	0.000617
negative regulation of androgen receptor signaling pathway	Biological Process	GO:0060766	0.000617
venous blood vessel development	Biological Process	GO:0060841	0.000617
otic vesicle development	Biological Process	GO:0071599	0.000617
metanephric mesenchyme development	Biological Process	GO:0072075	0.000617
establishment of planar polarity involved in neural tube closure	Biological Process	GO:0090177	0.000617
positive regulation of exosomal secretion	Biological Process	GO:1903543	0.000617
regulation of Wnt signaling pathway, planar cell polarity pathway	Biological Process	GO:2000095	0.000617
positive regulation of endothelial cell chemotaxis	Biological Process	GO:2001028	0.000617
synaptic vesicle endocytosis	Biological Process	GO:0048488	0.000618
cell communication involved in cardiac conduction	Biological Process	GO:0086065	0.000618
presynaptic endocytosis	Biological Process	GO:0140238	0.000618
glycolytic process	Biological Process	GO:0006096	0.000634
nuclear chromosome segregation	Biological Process	GO:0098813	0.000638
neurotransmitter secretion	Biological Process	GO:0007269	0.000638
signal release from synapse	Biological Process	GO:0099643	0.000638
negative regulation of chemokine production	Biological Process	GO:0032682	0.000638
CD4-positive, alpha-beta T cell cytokine production	Biological Process	GO:0035743	0.000638
platelet morphogenesis	Biological Process	GO:0036344	0.000638
PERK-mediated unfolded protein response	Biological Process	GO:0036499	0.000638
thymic T cell selection	Biological Process	GO:0045061	0.000638

response to corticosterone	Biological Process	GO:0051412	0.000638
labyrinthine layer morphogenesis	Biological Process	GO:0060713	0.000638
neuron projection arborization	Biological Process	GO:0140058	0.000638
positive regulation of lymphocyte chemotaxis	Biological Process	GO:0140131	0.000638
regulation of establishment of cell polarity	Biological Process	GO:2000114	0.000638
double-strand break repair	Biological Process	GO:0006302	0.00064
glycerophospholipid metabolic process	Biological Process	GO:0006650	0.00064
mast cell activation	Biological Process	GO:0045576	0.000643
positive regulation of transcription of Notch receptor target	Biological Process	GO:0007221	0.000649
modulation by virus of host cellular process	Biological Process	GO:0019054	0.000649
membrane protein intracellular domain proteolysis	Biological Process	GO:0031293	0.000649
osteoclast development	Biological Process	GO:0036035	0.000649
positive regulation of bone resorption	Biological Process	GO:0045780	0.000649
positive regulation of bone remodeling	Biological Process	GO:0046852	0.000649
regulation of immunoglobulin secretion	Biological Process	GO:0051023	0.000649
positive regulation of lymphocyte apoptotic process	Biological Process	GO:0070230	0.000649
negative regulation of T cell apoptotic process	Biological Process	GO:0070233	0.000649
hepatocyte proliferation	Biological Process	GO:0072574	0.000649
epithelial cell proliferation involved in liver morphogenesis	Biological Process	GO:0072575	0.000649
positive regulation of monocyte chemotaxis	Biological Process	GO:0090026	0.000649
regulation of platelet aggregation	Biological Process	GO:0090330	0.000649
regulation of T-helper 17 cell differentiation	Biological Process	GO:2000319	0.000649
mRNA stabilization	Biological Process	GO:0048255	0.000656
microtubule polymerization	Biological Process	GO:0046785	0.000674
mitotic cytokinesis	Biological Process	GO:0000281	0.000675
positive regulation of insulin secretion	Biological Process	GO:0032024	0.000675
ADP metabolic process	Biological Process	GO:0046031	0.000678
regulation of phosphatase activity	Biological Process	GO:0010921	0.00068
triglyceride metabolic process	Biological Process	GO:0006641	0.000686
Golgi vesicle transport	Biological Process	GO:0048193	0.000687
synaptic transmission, dopaminergic	Biological Process	GO:0001963	0.000698
positive regulation of acute inflammatory response	Biological Process	GO:0002675	0.000698
exit from mitosis	Biological Process	GO:0010458	0.000698
negative regulation of epithelial to mesenchymal transition	Biological Process	GO:0010719	0.000698
regulation of fibroblast migration	Biological Process	GO:0010762	0.000698
olfactory lobe development	Biological Process	GO:0021988	0.000698

negative regulation of calcium-mediated signaling	Biological Process	GO:0050849	0.000698
relaxation of muscle	Biological Process	GO:0090075	0.000698
blastocyst formation	Biological Process	GO:0001825	0.000705
mismatch repair	Biological Process	GO:0006298	0.000705
positive regulation of glucose metabolic process	Biological Process	GO:0010907	0.000705
carbohydrate catabolic process	Biological Process	GO:0016052	0.000706
regulation of DNA recombination	Biological Process	GO:0000018	0.000712
ATP generation from ADP	Biological Process	GO:0006757	0.00075
negative regulation of epithelial cell migration	Biological Process	GO:0010633	0.00075
glycerophospholipid biosynthetic process	Biological Process	GO:0046474	0.000764
ribonucleotide biosynthetic process	Biological Process	GO:0009260	0.000774
cytosolic transport	Biological Process	GO:0016482	0.000777
striated muscle adaptation	Biological Process	GO:0014888	0.000803
negative regulation of mitotic sister chromatid segregation	Biological Process	GO:0033048	0.000803
fever generation	Biological Process	GO:0001660	0.000803
cardiac ventricle formation	Biological Process	GO:0003211	0.000803
foregut morphogenesis	Biological Process	GO:0007440	0.000803
positive regulation of prostaglandin secretion	Biological Process	GO:0032308	0.000803
aldosterone metabolic process	Biological Process	GO:0032341	0.000803
aldosterone biosynthetic process	Biological Process	GO:0032342	0.000803
positive regulation of osteoblast proliferation	Biological Process	GO:0033690	0.000803
mitochondrial fragmentation involved in apoptotic process	Biological Process	GO:0043653	0.000803
positive regulation of MHC class II biosynthetic process	Biological Process	GO:0045348	0.000803
negative regulation of cell size	Biological Process	GO:0045792	0.000803
autophagic cell death	Biological Process	GO:0048102	0.000803
somatic stem cell division	Biological Process	GO:0048103	0.000803
positive regulation of isotype switching to IgG isotypes	Biological Process	GO:0048304	0.000803
paraxial mesoderm morphogenesis	Biological Process	GO:0048340	0.000803
branching morphogenesis of a nerve	Biological Process	GO:0048755	0.000803
vagina development	Biological Process	GO:0060068	0.000803
prostatic bud formation	Biological Process	GO:0060513	0.000803
regulation of epithelial cell proliferation involved in prostate gland development	Biological Process	GO:0060768	0.000803
lung secretory cell differentiation	Biological Process	GO:0061140	0.000803
response to lipoteichoic acid	Biological Process	GO:0070391	0.000803
cellular response to lipoteichoic acid	Biological Process	GO:0071223	0.000803
regulation of glomerular mesangial cell proliferation	Biological Process	GO:0072124	0.000803

primitive streak formation	Biological Process	GO:0090009	0.000803
positive regulation of cholesterol metabolic process	Biological Process	GO:0090205	0.000803
establishment of endothelial intestinal barrier	Biological Process	GO:0090557	0.000803
cellular response to oxidised low-density lipoprotein particle stimulus	Biological Process	GO:0140052	0.000803
negative regulation of amyloid-beta clearance	Biological Process	GO:1900222	0.000803
positive regulation of tumor necrosis factor-mediated signaling pathway	Biological Process	GO:1903265	0.000803
negative regulation of production of miRNAs involved in gene silencing by miRNA	Biological Process	GO:1903799	0.000803
regulation of dopaminergic neuron differentiation	Biological Process	GO:1904338	0.000803
positive regulation of cell-cell adhesion mediated by cadherin	Biological Process	GO:2000049	0.000803
regulation of receptor catabolic process	Biological Process	GO:2000644	0.000803
negative regulation of steroid biosynthetic process	Biological Process	GO:0010894	0.000805
response to gonadotropin	Biological Process	GO:0034698	0.000805
embryonic hindlimb morphogenesis	Biological Process	GO:0035116	0.000805
regulation of hair cycle	Biological Process	GO:0042634	0.000805
regulation of B cell differentiation	Biological Process	GO:0045577	0.000805
positive regulation of alcohol biosynthetic process	Biological Process	GO:1902932	0.000805
embryonic digit morphogenesis	Biological Process	GO:0042733	0.000817
negative regulation of lipid localization	Biological Process	GO:1905953	0.000817
nucleoside diphosphate metabolic process	Biological Process	GO:0009132	0.000821
nucleoside diphosphate phosphorylation	Biological Process	GO:0006165	0.000832
regulation of alternative mRNA splicing, via spliceosome	Biological Process	GO:0000381	0.000832
regulation of histone methylation	Biological Process	GO:0031060	0.000832
DNA geometric change	Biological Process	GO:0032392	0.000836
phospholipid biosynthetic process	Biological Process	GO:0008654	0.000842
multicellular organism aging	Biological Process	GO:0010259	0.000842
myotube cell development	Biological Process	GO:0014904	0.000842
apoptotic nuclear changes	Biological Process	GO:0030262	0.000842
negative regulation of interferon-gamma production	Biological Process	GO:0032689	0.000842
tumor necrosis factor secretion	Biological Process	GO:1990774	0.000842
post-translational protein modification	Biological Process	GO:0043687	0.00087
regulation of acute inflammatory response	Biological Process	GO:0002673	0.000877
establishment or maintenance of apical/basal cell polarity	Biological Process	GO:0035088	0.000877
oocyte development	Biological Process	GO:0048599	0.000877
establishment or maintenance of bipolar cell polarity	Biological Process	GO:0061245	0.000877
synapse assembly	Biological Process	GO:0007416	0.000882
regulation of phagocytosis	Biological Process	GO:0050764	0.000885

cell killing	Biological Process	GO:0001906	0.000886
tolerance induction	Biological Process	GO:0002507	0.00092
positive regulation of protein oligomerization	Biological Process	GO:0032461	0.00092
regulation of response to interferon-gamma	Biological Process	GO:0060330	0.00092
regulation of interferon-gamma-mediated signaling pathway	Biological Process	GO:0060334	0.00092
cellular response to growth hormone stimulus	Biological Process	GO:0071378	0.00092
centrosome cycle	Biological Process	GO:0007098	0.000934
positive regulation of anion transport	Biological Process	GO:1903793	0.000942
fatty acid biosynthetic process	Biological Process	GO:0006633	0.000952
fibroblast migration	Biological Process	GO:0010761	0.000976
negative regulation of muscle hypertrophy	Biological Process	GO:0014741	0.000976
vascular smooth muscle cell differentiation	Biological Process	GO:0035886	0.000976
regulation of membrane repolarization	Biological Process	GO:0060306	0.000976
regulation of macrophage migration	Biological Process	GO:1905521	0.000976
hormone biosynthetic process	Biological Process	GO:0042446	0.000981
telomere maintenance via telomere lengthening	Biological Process	GO:0010833	0.00101
regulation of synaptic transmission, GABAergic	Biological Process	GO:0032228	0.00101
positive regulation of vasoconstriction	Biological Process	GO:0045907	0.00101
regulation of axon extension involved in axon guidance	Biological Process	GO:0048841	0.00101
regulation of tumor necrosis factor secretion	Biological Process	GO:1904467	0.00101
response to lithium ion	Biological Process	GO:0010226	0.00103
positive regulation of cyclase activity	Biological Process	GO:0031281	0.00103
regulation of nuclease activity	Biological Process	GO:0032069	0.00103
interleukin-5 production	Biological Process	GO:0032634	0.00103
regulation of superoxide anion generation	Biological Process	GO:0032928	0.00103
regulation of protein sumoylation	Biological Process	GO:0033233	0.00103
negative regulation of Rho protein signal transduction	Biological Process	GO:0035024	0.00103
intestinal epithelial cell differentiation	Biological Process	GO:0060575	0.00103
cellular response to prostaglandin stimulus	Biological Process	GO:0071379	0.00103
regulation of ubiquitin protein ligase activity	Biological Process	GO:1904666	0.00103
associative learning	Biological Process	GO:0008306	0.00103
regulation of synaptic vesicle exocytosis	Biological Process	GO:2000300	0.00103
macrophage activation involved in immune response	Biological Process	GO:0002281	0.00103
Wnt signaling pathway involved in heart development	Biological Process	GO:0003306	0.00103
negative regulation of cell fate commitment	Biological Process	GO:0010454	0.00103
negative regulation of macrophage derived foam cell differentiation	Biological Process	GO:0010745	0.00103

positive regulation of fibroblast migration	Biological Process	GO:0010763	0.00103
negative regulation of muscle adaptation	Biological Process	GO:0014745	0.00103
regulation of vitamin metabolic process	Biological Process	GO:0030656	0.00103
regulation of heat generation	Biological Process	GO:0031650	0.00103
regulation of microvillus organization	Biological Process	GO:0032530	0.00103
positive regulation of intracellular steroid hormone receptor signaling pathway	Biological Process	GO:0033145	0.00103
protein hexamerization	Biological Process	GO:0034214	0.00103
collagen-activated signaling pathway	Biological Process	GO:0038065	0.00103
negative regulation of oligodendrocyte differentiation	Biological Process	GO:0048715	0.00103
white fat cell differentiation	Biological Process	GO:0050872	0.00103
lateral sprouting from an epithelium	Biological Process	GO:0060601	0.00103
cellular response to manganese ion	Biological Process	GO:0071287	0.00103
cellular response to interleukin-2	Biological Process	GO:0071352	0.00103
ectodermal placode development	Biological Process	GO:0071696	0.00103
cellular response to epinephrine stimulus	Biological Process	GO:0071872	0.00103
planar cell polarity pathway involved in neural tube closure	Biological Process	GO:0090179	0.00103
regulation of histone H4 acetylation	Biological Process	GO:0090239	0.00103
positive regulation of endoplasmic reticulum unfolded protein response	Biological Process	GO:1900103	0.00103
negative regulation of long-term synaptic potentiation	Biological Process	GO:1900272	0.00103
regulation of endothelial cell development	Biological Process	GO:1901550	0.00103
regulation of establishment of endothelial barrier	Biological Process	GO:1903140	0.00103
negative regulation of anion transmembrane transport	Biological Process	GO:1903960	0.00103
positive regulation of non-canonical Wnt signaling pathway	Biological Process	GO:2000052	0.00103
glycogen metabolic process	Biological Process	GO:0005977	0.00103
lysosome localization	Biological Process	GO:0032418	0.00103
negative regulation of chromatin organization	Biological Process	GO:1905268	0.00103
telomere maintenance via telomerase	Biological Process	GO:0007004	0.00104
demethylation	Biological Process	GO:0070988	0.00104
low-density lipoprotein particle clearance	Biological Process	GO:0034383	0.00107
keratinocyte proliferation	Biological Process	GO:0043616	0.00107
neutral lipid biosynthetic process	Biological Process	GO:0046460	0.00107
acylglycerol biosynthetic process	Biological Process	GO:0046463	0.00107
positive regulation of calcium-mediated signaling	Biological Process	GO:0050850	0.00107
sensory perception of mechanical stimulus	Biological Process	GO:0050954	0.00108
TOR signaling	Biological Process	GO:0031929	0.00108
microtubule polymerization or depolymerization	Biological Process	GO:0031109	0.00109

regulation of exit from mitosis	Biological Process	GO:0007096	0.00109
blastoderm segmentation	Biological Process	GO:0007350	0.00109
myelin assembly	Biological Process	GO:0032288	0.00109
response to vitamin A	Biological Process	GO:0033189	0.00109
positive regulation of myeloid leukocyte cytokine production involved in immune response	Biological Process	GO:0061081	0.00109
cellular response to arsenic-containing substance	Biological Process	GO:0071243	0.00109
lymphangiogenesis	Biological Process	GO:0001946	0.0011
T-helper cell lineage commitment	Biological Process	GO:0002295	0.0011
positive regulation of cholesterol efflux	Biological Process	GO:0010875	0.0011
macrophage cytokine production	Biological Process	GO:0010934	0.0011
microvillus assembly	Biological Process	GO:0030033	0.0011
hyaluronan catabolic process	Biological Process	GO:0030214	0.0011
membrane disassembly	Biological Process	GO:0030397	0.0011
negative regulation of B cell proliferation	Biological Process	GO:0030889	0.0011
corticosteroid receptor signaling pathway	Biological Process	GO:0031958	0.0011
granulocyte macrophage colony-stimulating factor production	Biological Process	GO:0032604	0.0011
estrous cycle	Biological Process	GO:0044849	0.0011
negative regulation of T-helper cell differentiation	Biological Process	GO:0045623	0.0011
nuclear envelope disassembly	Biological Process	GO:0051081	0.0011
relaxation of cardiac muscle	Biological Process	GO:0055119	0.0011
cellular response to prostaglandin E stimulus	Biological Process	GO:0071380	0.0011
response to epinephrine	Biological Process	GO:0071871	0.0011
catecholamine uptake	Biological Process	GO:0090493	0.0011
nucleotide phosphorylation	Biological Process	GO:0046939	0.00111
regulation of actin polymerization or depolymerization	Biological Process	GO:0008064	0.00113
bone growth	Biological Process	GO:0098868	0.00115
growth plate cartilage development	Biological Process	GO:0003417	0.00117
superoxide anion generation	Biological Process	GO:0042554	0.00117
regulation of protein targeting to membrane	Biological Process	GO:0090313	0.00117
energy reserve metabolic process	Biological Process	GO:0006112	0.00117
epithelial structure maintenance	Biological Process	GO:0010669	0.00117
regulation of cardiac muscle contraction by calcium ion signaling	Biological Process	GO:0010882	0.00117
regulation of DNA methylation	Biological Process	GO:0044030	0.00117
cartilage morphogenesis	Biological Process	GO:0060536	0.00117
response to cholesterol	Biological Process	GO:0070723	0.00117

positive regulation of neutrophil migration	Biological Process	GO:1902624	0.00117
negative regulation of dendrite development	Biological Process	GO:2000171	0.00117
positive regulation of cardiac muscle cell differentiation	Biological Process	GO:2000727	0.00117
sensory perception of pain	Biological Process	GO:0019233	0.00119
oocyte differentiation	Biological Process	GO:0009994	0.00121
NAD metabolic process	Biological Process	GO:0019674	0.00121
cranial nerve development	Biological Process	GO:0021545	0.00121
regulation of mitotic metaphase/anaphase transition	Biological Process	GO:0030071	0.00121
negative regulation of G protein-coupled receptor signaling pathway	Biological Process	GO:0045744	0.00121
negative regulation of cytoskeleton organization	Biological Process	GO:0051494	0.00123
cellular glucan metabolic process	Biological Process	GO:0006073	0.00127
glucan metabolic process	Biological Process	GO:0044042	0.00127
regulation of actin filament length	Biological Process	GO:0030832	0.00128
negative regulation of RNA catabolic process	Biological Process	GO:1902369	0.00129
histone H4 acetylation	Biological Process	GO:0043967	0.0013
sister chromatid cohesion	Biological Process	GO:0007062	0.0013
mating	Biological Process	GO:0007618	0.00131
cerebral cortex cell migration	Biological Process	GO:0021795	0.00131
positive regulation of cytokinesis	Biological Process	GO:0032467	0.00131
negative regulation of cytokine biosynthetic process	Biological Process	GO:0042036	0.00131
positive regulation of release of sequestered calcium ion into cytosol	Biological Process	GO:0051281	0.00131
cellular process involved in reproduction in multicellular organism	Biological Process	GO:0022412	0.00131
regulation of actin filament polymerization	Biological Process	GO:0030833	0.00132
negative regulation of lipid metabolic process	Biological Process	GO:0045833	0.00137
apoptotic DNA fragmentation	Biological Process	GO:0006309	0.00138
positive regulation of heart rate	Biological Process	GO:0010460	0.00138
positive regulation of granulocyte chemotaxis	Biological Process	GO:0071624	0.00138
negative regulation of ATP metabolic process	Biological Process	GO:1903579	0.00138
positive regulation of vascular associated smooth muscle cell migration	Biological Process	GO:1904754	0.00138
fatty acid derivative metabolic process	Biological Process	GO:1901568	0.0014
regulation of Rho protein signal transduction	Biological Process	GO:0035023	0.00141
regulation of keratinocyte proliferation	Biological Process	GO:0010837	0.00142
positive regulation of interleukin-1 beta secretion	Biological Process	GO:0050718	0.00142
endochondral bone growth	Biological Process	GO:0003416	0.00143
negative regulation of sister chromatid segregation	Biological Process	GO:0033046	0.00143
positive regulation of protein dephosphorylation	Biological Process	GO:0035307	0.00143

membrane biogenesis	Biological Process	GO:0044091	0.00143
cell-cell adhesion mediated by cadherin	Biological Process	GO:0044331	0.00143
leukocyte adhesion to vascular endothelial cell	Biological Process	GO:0061756	0.00143
regulation of protein targeting to mitochondrion	Biological Process	GO:1903214	0.00143
negative regulation of response to endoplasmic reticulum stress	Biological Process	GO:1903573	0.00143
negative regulation of viral process	Biological Process	GO:0048525	0.00149
actin polymerization or depolymerization	Biological Process	GO:0008154	0.00149
monovalent inorganic cation homeostasis	Biological Process	GO:0055067	0.00152
calcium ion transmembrane transport	Biological Process	GO:0070588	0.00156
fatty acid transport	Biological Process	GO:0015908	0.00157
response to fungus	Biological Process	GO:0009620	0.00158
ruffle organization	Biological Process	GO:0031529	0.00158
positive regulation of sterol transport	Biological Process	GO:0032373	0.00158
positive regulation of cholesterol transport	Biological Process	GO:0032376	0.00158
CD8-positive, alpha-beta T cell activation	Biological Process	GO:0036037	0.00158
regulation of histone H3-K9 methylation	Biological Process	GO:0051570	0.00158
cellular response to vitamin D	Biological Process	GO:0071305	0.00158
membrane depolarization during cardiac muscle cell action potential	Biological Process	GO:0086012	0.00158
renal filtration	Biological Process	GO:0097205	0.00158
DNA replication initiation	Biological Process	GO:0006270	0.00162
cellular component disassembly involved in execution phase of apoptosis	Biological Process	GO:0006921	0.00162
positive regulation of telomere maintenance via telomere lengthening	Biological Process	GO:1904358	0.00162
amino acid transport	Biological Process	GO:0006865	0.00162
cardiac muscle cell apoptotic process	Biological Process	GO:0010659	0.00163
natural killer cell mediated cytotoxicity	Biological Process	GO:0042267	0.00163
insulin secretion involved in cellular response to glucose stimulus	Biological Process	GO:0035773	0.00163
negative regulation of chemotaxis	Biological Process	GO:0050922	0.00163
regulation of systemic arterial blood pressure	Biological Process	GO:0003073	0.00164
cellular response to dopamine	Biological Process	GO:1903351	0.00164
actin filament polymerization	Biological Process	GO:0030041	0.00165
olfactory bulb development	Biological Process	GO:0021772	0.0017
negative regulation of steroid metabolic process	Biological Process	GO:0045939	0.0017
microtubule organizing center localization	Biological Process	GO:0061842	0.0017
regulation of mRNA processing	Biological Process	GO:0050684	0.00173
pro-B cell differentiation	Biological Process	GO:0002328	0.00173
lateral ventricle development	Biological Process	GO:0021670	0.00173

positive regulation of heat generation	Biological Process	GO:0031652	0.00173
regulation of prostaglandin secretion	Biological Process	GO:0032306	0.00173
regulation of transforming growth factor beta1 production	Biological Process	GO:0032908	0.00173
positive regulation of intracellular estrogen receptor signaling pathway	Biological Process	GO:0033148	0.00173
negative regulation of cell adhesion mediated by integrin	Biological Process	GO:0033629	0.00173
TRAIL-activated apoptotic signaling pathway	Biological Process	GO:0036462	0.00173
negative regulation of multicellular organism growth	Biological Process	GO:0040015	0.00173
regulation of transcription from RNA polymerase II promoter in response to oxidative stress	Biological Process	GO:0043619	0.00173
type I interferon biosynthetic process	Biological Process	GO:0045351	0.00173
positive regulation of cell size	Biological Process	GO:0045793	0.00173
negative regulation of activated T cell proliferation	Biological Process	GO:0046007	0.00173
ventricular cardiac muscle cell development	Biological Process	GO:0055015	0.00173
regulation of dopamine receptor signaling pathway	Biological Process	GO:0060159	0.00173
bronchus development	Biological Process	GO:0060433	0.00173
blood vessel endothelial cell differentiation	Biological Process	GO:0060837	0.00173
response to interleukin-18	Biological Process	GO:0070673	0.00173
positive regulation of protein homodimerization activity	Biological Process	GO:0090073	0.00173
regulation of osteoclast development	Biological Process	GO:2001204	0.00173
cardiac conduction	Biological Process	GO:0061337	0.00174
cellular component assembly involved in morphogenesis	Biological Process	GO:0010927	0.00176
regulation of monocyte differentiation	Biological Process	GO:0045655	0.00176
peptidyl-serine dephosphorylation	Biological Process	GO:0070262	0.00176
regulation of microglial cell activation	Biological Process	GO:1903978	0.00176
regulation of keratinocyte differentiation	Biological Process	GO:0045616	0.00176
membrane docking	Biological Process	GO:0022406	0.00176
negative regulation of gene expression, epigenetic	Biological Process	GO:0045814	0.00176
acid secretion	Biological Process	GO:0046717	0.00182
regulation of regulated secretory pathway	Biological Process	GO:1903305	0.00184
smoothened signaling pathway	Biological Process	GO:0007224	0.00185
regulation of mRNA splicing, via spliceosome	Biological Process	GO:0048024	0.00186
negative regulation of supramolecular fiber organization	Biological Process	GO:1902904	0.00186
negative regulation of chromosome segregation	Biological Process	GO:0051985	0.00189
prostaglandin transport	Biological Process	GO:0015732	0.0019
response to insecticide	Biological Process	GO:0017085	0.0019
positive regulation by host of viral transcription	Biological Process	GO:0043923	0.0019

calcium ion transmembrane transport via high voltage-gated calcium channel	Biological Process	GO:0061577	0.0019
regulation of glial cell migration	Biological Process	GO:1903975	0.0019
positive regulation of leukocyte adhesion to vascular endothelial cell	Biological Process	GO:1904996	0.0019
heart valve formation	Biological Process	GO:0003188	0.00191
cardiac left ventricle morphogenesis	Biological Process	GO:0003214	0.00191
negative regulation of epidermal growth factor-activated receptor activity	Biological Process	GO:0007175	0.00191
regulation of macrophage cytokine production	Biological Process	GO:0010935	0.00191
response to inactivity	Biological Process	GO:0014854	0.00191
prostaglandin secretion	Biological Process	GO:0032310	0.00191
low-density lipoprotein particle receptor catabolic process	Biological Process	GO:0032802	0.00191
CD8-positive, alpha-beta T cell differentiation	Biological Process	GO:0043374	0.00191
negative regulation by host of viral transcription	Biological Process	GO:0043922	0.00191
response to redox state	Biological Process	GO:0051775	0.00191
Sertoli cell development	Biological Process	GO:0060009	0.00191
regulation of nuclear-transcribed mRNA poly(A) tail shortening	Biological Process	GO:0060211	0.00191
cardiac cell fate commitment	Biological Process	GO:0060911	0.00191
response to interleukin-2	Biological Process	GO:0070669	0.00191
response to interleukin-15	Biological Process	GO:0070672	0.00191
negative regulation of calcineurin-NFAT signaling cascade	Biological Process	GO:0070885	0.00191
cellular response to ethanol	Biological Process	GO:0071361	0.00191
chemokine (C-X-C motif) ligand 2 production	Biological Process	GO:0072567	0.00191
regulation of establishment of planar polarity involved in neural tube closure	Biological Process	GO:0090178	0.00191
negative regulation of calcineurin-mediated signaling	Biological Process	GO:0106057	0.00191
regulation of apoptotic process involved in development	Biological Process	GO:1904748	0.00191
response to dopamine	Biological Process	GO:1903350	0.00194
positive regulation of telomere maintenance via telomerase	Biological Process	GO:0032212	0.00196
cell death in response to hydrogen peroxide	Biological Process	GO:0036474	0.00196
neuron fate specification	Biological Process	GO:0048665	0.00196
peptidyl-lysine methylation	Biological Process	GO:0018022	0.00196
adenylate cyclase-modulating G protein-coupled receptor signaling pathway	Biological Process	GO:0007188	0.00198
visual behavior	Biological Process	GO:0007632	0.00201
regulation of interleukin-1 secretion	Biological Process	GO:0050704	0.00201
regulation of metaphase/anaphase transition of cell cycle	Biological Process	GO:1902099	0.00201
macrophage migration	Biological Process	GO:1905517	0.00201
regulation of T-helper 1 type immune response	Biological Process	GO:0002825	0.00201
positive regulation of coagulation	Biological Process	GO:0050820	0.00201

myoblast proliferation	Biological Process	GO:0051450	0.00201
negative regulation of cellular response to drug	Biological Process	GO:2001039	0.00201
adherens junction organization	Biological Process	GO:0034332	0.00201
positive regulation of DNA repair	Biological Process	GO:0045739	0.00201
regulation of antigen receptor-mediated signaling pathway	Biological Process	GO:0050854	0.00201
regulation of cardiac muscle cell apoptotic process	Biological Process	GO:0010665	0.00203
monoamine transport	Biological Process	GO:0015844	0.00203
interleukin-1 secretion	Biological Process	GO:0050701	0.00203
vacuolar transport	Biological Process	GO:0007034	0.00212
behavioral defense response	Biological Process	GO:0002209	0.00217
physiological muscle hypertrophy	Biological Process	GO:0003298	0.00217
physiological cardiac muscle hypertrophy	Biological Process	GO:0003301	0.00217
early endosome to late endosome transport	Biological Process	GO:0045022	0.00217
insulin-like growth factor receptor signaling pathway	Biological Process	GO:0048009	0.00217
macrophage chemotaxis	Biological Process	GO:0048246	0.00217
cell growth involved in cardiac muscle cell development	Biological Process	GO:0061049	0.00217
negative regulation of vascular smooth muscle cell proliferation	Biological Process	GO:1904706	0.00217
sterol transport	Biological Process	GO:0015918	0.00217
regulation of cellular ketone metabolic process	Biological Process	GO:0010565	0.00224
regulation of leukocyte mediated cytotoxicity	Biological Process	GO:0001910	0.0023
positive regulation of reproductive process	Biological Process	GO:2000243	0.0023
macromolecule methylation	Biological Process	GO:0043414	0.00233
cellular response to glucose starvation	Biological Process	GO:0042149	0.00234
forebrain neuron development	Biological Process	GO:0021884	0.00237
regulation of T cell differentiation in thymus	Biological Process	GO:0033081	0.00237
toll-like receptor 9 signaling pathway	Biological Process	GO:0034162	0.00237
fibroblast apoptotic process	Biological Process	GO:0044346	0.00237
negative regulation of receptor signaling pathway via JAK-STAT	Biological Process	GO:0046426	0.00237
cell communication by electrical coupling involved in cardiac conduction	Biological Process	GO:0086064	0.00237
positive regulation of neutrophil chemotaxis	Biological Process	GO:0090023	0.00237
regulation of bone development	Biological Process	GO:1903010	0.00237
negative regulation of cytokine secretion	Biological Process	GO:0050710	0.00238
muscle hypertrophy in response to stress	Biological Process	GO:0003299	0.00238
cardiac muscle adaptation	Biological Process	GO:0014887	0.00238
cardiac muscle hypertrophy in response to stress	Biological Process	GO:0014898	0.00238
positive regulation of multicellular organism growth	Biological Process	GO:0040018	0.00238

long-term synaptic depression	Biological Process	GO:0060292	0.00238
regulation of intrinsic apoptotic signaling pathway by p53 class mediator	Biological Process	GO:1902253	0.00238
apoptotic cell clearance	Biological Process	GO:0043277	0.00245
negative chemotaxis	Biological Process	GO:0050919	0.00245
vascular associated smooth muscle cell migration	Biological Process	GO:1904738	0.00245
regulation of vascular associated smooth muscle cell migration	Biological Process	GO:1904752	0.00245
RNA stabilization	Biological Process	GO:0043489	0.00252
membrane repolarization	Biological Process	GO:0086009	0.00252
respiratory gaseous exchange by respiratory system	Biological Process	GO:0007585	0.00254
neural nucleus development	Biological Process	GO:0048857	0.00254
protein insertion into membrane	Biological Process	GO:0051205	0.00254
regulation of sodium ion transmembrane transport	Biological Process	GO:1902305	0.00254
regulation of carbohydrate catabolic process	Biological Process	GO:0043470	0.00255
oogenesis	Biological Process	GO:0048477	0.00255
catecholamine metabolic process	Biological Process	GO:0006584	0.00255
catechol-containing compound metabolic process	Biological Process	GO:0009712	0.00255
thioester biosynthetic process	Biological Process	GO:0035384	0.00255
acyl-CoA biosynthetic process	Biological Process	GO:0071616	0.00255
anion homeostasis	Biological Process	GO:0055081	0.00256
hormone metabolic process	Biological Process	GO:0042445	0.00258
cytoskeleton-dependent cytokinesis	Biological Process	GO:0061640	0.00258
hematopoietic stem cell differentiation	Biological Process	GO:0060218	0.00267
toll-like receptor 4 signaling pathway	Biological Process	GO:0034142	0.00268
positive regulation of viral genome replication	Biological Process	GO:0045070	0.00268
regulation of type I interferon-mediated signaling pathway	Biological Process	GO:0060338	0.00268
protein depolymerization	Biological Process	GO:0051261	0.0027
natural killer cell differentiation	Biological Process	GO:0001779	0.00274
catechol-containing compound biosynthetic process	Biological Process	GO:0009713	0.00274
negative regulation of peptidyl-threonine phosphorylation	Biological Process	GO:0010801	0.00274
ovulation	Biological Process	GO:0030728	0.00274
regulation of interleukin-5 production	Biological Process	GO:0032674	0.00274
protein-DNA complex disassembly	Biological Process	GO:0032986	0.00274
catecholamine biosynthetic process	Biological Process	GO:0042423	0.00274
positive regulation of tumor necrosis factor secretion	Biological Process	GO:1904469	0.00274
regulation of T-helper 17 type immune response	Biological Process	GO:2000316	0.00274
negative regulation of stem cell differentiation	Biological Process	GO:2000737	0.00274

positive regulation of organelle assembly	Biological Process	GO:1902117	0.00289
myeloid dendritic cell activation	Biological Process	GO:0001773	0.00289
hyperosmotic response	Biological Process	GO:0006972	0.00289
cell proliferation in forebrain	Biological Process	GO:0021846	0.00289
fibrinolysis	Biological Process	GO:0042730	0.00289
regulation of transcription initiation from RNA polymerase II promoter	Biological Process	GO:0060260	0.00289
histone lysine demethylation	Biological Process	GO:0070076	0.00289
negative regulation of axon guidance	Biological Process	GO:1902668	0.00289
positive regulation of epidermis development	Biological Process	GO:0045684	0.00291
negative regulation of mitotic sister chromatid separation	Biological Process	GO:2000816	0.00291
regulation of protein oligomerization	Biological Process	GO:0032459	0.00307
activin receptor signaling pathway	Biological Process	GO:0032924	0.00307
establishment or maintenance of epithelial cell apical/basal polarity	Biological Process	GO:0045197	0.00307
negative regulation of sodium ion transport	Biological Process	GO:0010766	0.00309
regulation of fatty acid beta-oxidation	Biological Process	GO:0031998	0.00309
interferon-gamma biosynthetic process	Biological Process	GO:0042095	0.00309
myeloid dendritic cell differentiation	Biological Process	GO:0043011	0.00309
paraxial mesoderm development	Biological Process	GO:0048339	0.00309
negative regulation of focal adhesion assembly	Biological Process	GO:0051895	0.00309
cellular response to gonadotropin stimulus	Biological Process	GO:0071371	0.00309
monocyte chemotactic protein-1 production	Biological Process	GO:0071605	0.00309
regulation of monocyte chemotactic protein-1 production	Biological Process	GO:0071637	0.00309
nephron tubule formation	Biological Process	GO:0072079	0.00309
positive regulation of neuroinflammatory response	Biological Process	GO:0150078	0.00309
negative regulation of cell-substrate junction organization	Biological Process	GO:0150118	0.00309
positive regulation of nuclear-transcribed mRNA catabolic process, deadenylation-dependent decay	Biological Process	GO:1900153	0.00309
purine ribonucleoside triphosphate biosynthetic process	Biological Process	GO:0009206	0.00314
glycosaminoglycan biosynthetic process	Biological Process	GO:0006024	0.00315
unsaturated fatty acid metabolic process	Biological Process	GO:0033559	0.00315
acute-phase response	Biological Process	GO:0006953	0.00316
cerebellar cortex development	Biological Process	GO:0021695	0.00316
lymphocyte mediated immunity	Biological Process	GO:0002449	0.00318
negative regulation of innate immune response	Biological Process	GO:0045824	0.00319
regulation of nervous system process	Biological Process	GO:0031644	0.00321
glycosaminoglycan metabolic process	Biological Process	GO:0030203	0.00327

phosphatidylinositol metabolic process	Biological Process	GO:0046488	0.0033
positive regulation of smooth muscle contraction	Biological Process	GO:0045987	0.0033
fatty-acyl-CoA biosynthetic process	Biological Process	GO:0046949	0.0033
skeletal muscle fiber development	Biological Process	GO:0048741	0.0033
trophectodermal cell differentiation	Biological Process	GO:0001829	0.0033
germinal center formation	Biological Process	GO:0002467	0.0033
regulation of granulocyte differentiation	Biological Process	GO:0030852	0.0033
negative regulation of homotypic cell-cell adhesion	Biological Process	GO:0034111	0.0033
fat-soluble vitamin biosynthetic process	Biological Process	GO:0042362	0.0033
glucocorticoid receptor signaling pathway	Biological Process	GO:0042921	0.0033
activation of Janus kinase activity	Biological Process	GO:0042976	0.0033
negative regulation of JUN kinase activity	Biological Process	GO:0043508	0.0033
long-chain fatty acid import into cell	Biological Process	GO:0044539	0.0033
negative regulation of bone remodeling	Biological Process	GO:0046851	0.0033
fatty acid homeostasis	Biological Process	GO:0055089	0.0033
negative regulation of glial cell proliferation	Biological Process	GO:0060253	0.0033
negative regulation of neuron projection regeneration	Biological Process	GO:0070571	0.0033
cellular hyperosmotic response	Biological Process	GO:0071474	0.0033
regulation of glomerulus development	Biological Process	GO:0090192	0.0033
dopamine uptake	Biological Process	GO:0090494	0.0033
lipid import into cell	Biological Process	GO:0140354	0.0033
cell junction disassembly	Biological Process	GO:0150146	0.0033
mitotic DNA replication	Biological Process	GO:1902969	0.0033
regulation of CD8-positive, alpha-beta T cell activation	Biological Process	GO:2001185	0.0033
nucleoside triphosphate biosynthetic process	Biological Process	GO:0009142	0.0033
Golgi vesicle budding	Biological Process	GO:0048194	0.0033
dendritic cell cytokine production	Biological Process	GO:0002371	0.0033
cardiac chamber formation	Biological Process	GO:0003207	0.0033
regulation of cell communication by electrical coupling	Biological Process	GO:0010649	0.0033
regulation of sequestering of triglyceride	Biological Process	GO:0010889	0.0033
muscle atrophy	Biological Process	GO:0014889	0.0033
regulation of phospholipase A2 activity	Biological Process	GO:0032429	0.0033
transforming growth factor beta1 production	Biological Process	GO:0032905	0.0033
primary alcohol biosynthetic process	Biological Process	GO:0034309	0.0033
endothelial cell activation	Biological Process	GO:0042118	0.0033
dopamine biosynthetic process	Biological Process	GO:0042416	0.0033

positive regulation of vascular permeability	Biological Process	GO:0043117	0.0033
response to ether	Biological Process	GO:0045472	0.0033
negative regulation of skeletal muscle tissue development	Biological Process	GO:0048642	0.0033
regulation of helicase activity	Biological Process	GO:0051095	0.0033
negative regulation of cytosolic calcium ion concentration	Biological Process	GO:0051481	0.0033
branching involved in labyrinthine layer morphogenesis	Biological Process	GO:0060670	0.0033
trophoblast giant cell differentiation	Biological Process	GO:0060707	0.0033
ectodermal placode formation	Biological Process	GO:0060788	0.0033
regulation of thymocyte apoptotic process	Biological Process	GO:0070243	0.0033
ectodermal placode morphogenesis	Biological Process	GO:0071697	0.0033
protein localization to cell cortex	Biological Process	GO:0072697	0.0033
negative regulation of platelet aggregation	Biological Process	GO:0090331	0.0033
negative regulation of transcription from RNA polymerase II promoter in response to stress	Biological Process	GO:0097201	0.0033
regulation of deacetylase activity	Biological Process	GO:0150065	0.0033
positive regulation of protein polyubiquitination	Biological Process	GO:1902916	0.0033
regulation of IRE1-mediated unfolded protein response	Biological Process	GO:1903894	0.0033
positive regulation of ubiquitin protein ligase activity	Biological Process	GO:1904668	0.0033
regulation of modification of synaptic structure	Biological Process	GO:1905244	0.0033
positive regulation of vascular associated smooth muscle cell apoptotic process	Biological Process	GO:1905461	0.0033
regulation of chemokine (C-X-C motif) ligand 2 production	Biological Process	GO:2000341	0.0033
positive regulation of B cell activation	Biological Process	GO:0050871	0.00336
cyclic-nucleotide-mediated signaling	Biological Process	GO:0019935	0.0034
microtubule organizing center organization	Biological Process	GO:0031023	0.0034
chromatin organization involved in regulation of transcription	Biological Process	GO:0034401	0.0034
mature B cell differentiation	Biological Process	GO:0002335	0.00341
adrenal gland development	Biological Process	GO:0030325	0.00341
regulation of lipopolysaccharide-mediated signaling pathway	Biological Process	GO:0031664	0.00341
GTP metabolic process	Biological Process	GO:0046039	0.00341
glucose 6-phosphate metabolic process	Biological Process	GO:0051156	0.00341
regulation of cell growth involved in cardiac muscle cell development	Biological Process	GO:0061050	0.00341
regulation of monocyte chemotaxis	Biological Process	GO:0090025	0.00341
cellular response to angiotensin	Biological Process	GO:1904385	0.00341
negative regulation of synapse organization	Biological Process	GO:1905809	0.00341
positive regulation of endothelial cell apoptotic process	Biological Process	GO:2000353	0.00341
drug transmembrane transport	Biological Process	GO:0006855	0.00342

ATP metabolic process	Biological Process	GO:0046034	0.00347
negative regulation of mitotic metaphase/anaphase transition	Biological Process	GO:0045841	0.00356
regulation of neutrophil migration	Biological Process	GO:1902622	0.00356
production of molecular mediator involved in inflammatory response	Biological Process	GO:0002532	0.00357
neural retina development	Biological Process	GO:0003407	0.0037
nucleoside bisphosphate biosynthetic process	Biological Process	GO:0033866	0.0037
ribonucleoside bisphosphate biosynthetic process	Biological Process	GO:0034030	0.0037
purine nucleoside bisphosphate biosynthetic process	Biological Process	GO:0034033	0.0037
multicellular organismal water homeostasis	Biological Process	GO:0050891	0.0037
NADH metabolic process	Biological Process	GO:0006734	0.00378
fear response	Biological Process	GO:0042596	0.00378
guanosine-containing compound metabolic process	Biological Process	GO:1901068	0.00378
negative regulation of chromosome separation	Biological Process	GO:1905819	0.00378
natural killer cell mediated immunity	Biological Process	GO:0002228	0.00381
purine nucleoside triphosphate biosynthetic process	Biological Process	GO:0009145	0.00381
lipid phosphorylation	Biological Process	GO:0046834	0.00381
vesicle docking	Biological Process	GO:0048278	0.00381
DNA modification	Biological Process	GO:0006304	0.00391
phosphatidylinositol biosynthetic process	Biological Process	GO:0006661	0.00391
glutamate secretion	Biological Process	GO:0014047	0.00391
phenol-containing compound biosynthetic process	Biological Process	GO:0046189	0.00391
positive regulation of dendritic spine development	Biological Process	GO:0060999	0.00391
cardiac muscle cell action potential involved in contraction	Biological Process	GO:0086002	0.00397
neuron recognition	Biological Process	GO:0008038	0.00398
visual learning	Biological Process	GO:0008542	0.00398
regulation of glycogen biosynthetic process	Biological Process	GO:0005979	0.00399
cell communication by electrical coupling	Biological Process	GO:0010644	0.00399
regulation of glucan biosynthetic process	Biological Process	GO:0010962	0.00399
histone demethylation	Biological Process	GO:0016577	0.00399
interleukin-6 biosynthetic process	Biological Process	GO:0042226	0.00399
centrosome localization	Biological Process	GO:0051642	0.00399
protein localization to chromosome, telomeric region	Biological Process	GO:0070198	0.00399
nuclear envelope organization	Biological Process	GO:0006998	0.00399
regulation of neuronal synaptic plasticity	Biological Process	GO:0048168	0.00399
regulation of sodium ion transmembrane transporter activity	Biological Process	GO:2000649	0.00399
lens morphogenesis in camera-type eye	Biological Process	GO:0002089	0.00404

glomerular filtration	Biological Process	GO:0003094	0.00404
mitotic recombination	Biological Process	GO:0006312	0.00404
mitochondrial fusion	Biological Process	GO:0008053	0.00404
dorsal spinal cord development	Biological Process	GO:0021516	0.00404
negative regulation of cell killing	Biological Process	GO:0031342	0.00404
endoplasmic reticulum calcium ion homeostasis	Biological Process	GO:0032469	0.00404
integrin activation	Biological Process	GO:0033622	0.00404
interleukin-2 biosynthetic process	Biological Process	GO:0042094	0.00404
response to leptin	Biological Process	GO:0044321	0.00404
positive regulation of insulin receptor signaling pathway	Biological Process	GO:0046628	0.00404
negative regulation of protein localization to plasma membrane	Biological Process	GO:1903077	0.00404
RNA-dependent DNA biosynthetic process	Biological Process	GO:0006278	0.00411
neutral lipid metabolic process	Biological Process	GO:0006638	0.00416
mucopolysaccharide metabolic process	Biological Process	GO:1903510	0.00416
chromosome separation	Biological Process	GO:0051304	0.00418
regulation of defense response to virus	Biological Process	GO:0050688	0.0043
fatty acid oxidation	Biological Process	GO:0019395	0.00436
cholesterol transport	Biological Process	GO:0030301	0.00436
regulation of lamellipodium assembly	Biological Process	GO:0010591	0.00442
erythrocyte development	Biological Process	GO:0048821	0.00442
negative regulation of NIK/NF-kappaB signaling	Biological Process	GO:1901223	0.00442
positive regulation of response to drug	Biological Process	GO:2001025	0.00442
regulation of phosphoprotein phosphatase activity	Biological Process	GO:0043666	0.0045
monocarboxylic acid transport	Biological Process	GO:0015718	0.00461
behavioral fear response	Biological Process	GO:0001662	0.00473
negative regulation of cardiac muscle hypertrophy	Biological Process	GO:0010614	0.00473
negative regulation of metaphase/anaphase transition of cell cycle	Biological Process	GO:1902100	0.00473
protein-DNA complex subunit organization	Biological Process	GO:0071824	0.00473
mature B cell differentiation involved in immune response	Biological Process	GO:0002313	0.00473
nucleosome disassembly	Biological Process	GO:0006337	0.00473
post-anal tail morphogenesis	Biological Process	GO:0036342	0.00473
muscle cell fate commitment	Biological Process	GO:0042693	0.00473
xenobiotic transport	Biological Process	GO:0042908	0.00473
regulation of interleukin-2 biosynthetic process	Biological Process	GO:0045076	0.00473
tetrahydrofolate metabolic process	Biological Process	GO:0046653	0.00473
labyrinthine layer blood vessel development	Biological Process	GO:0060716	0.00473

response to salt	Biological Process	GO:1902074	0.00473
negative regulation of oxidative stress-induced intrinsic apoptotic signaling pathway	Biological Process	GO:1902176	0.00473
tertiary alcohol metabolic process	Biological Process	GO:1902644	0.00473
vascular associated smooth muscle cell apoptotic process	Biological Process	GO:1905288	0.00473
regulation of vascular associated smooth muscle cell apoptotic process	Biological Process	GO:1905459	0.00473
glycosaminoglycan catabolic process	Biological Process	GO:0006027	0.00479
regulation of protein-containing complex disassembly	Biological Process	GO:0043244	0.00482
oocyte maturation	Biological Process	GO:0001556	0.00486
regulation of macrophage chemotaxis	Biological Process	GO:0010758	0.00486
positive regulation of blood coagulation	Biological Process	GO:0030194	0.00486
melanocyte differentiation	Biological Process	GO:0030318	0.00486
intracellular sterol transport	Biological Process	GO:0032366	0.00486
intracellular cholesterol transport	Biological Process	GO:0032367	0.00486
regulation of long-term neuronal synaptic plasticity	Biological Process	GO:0048169	0.00486
negative regulation of axon extension involved in axon guidance	Biological Process	GO:0048843	0.00486
positive regulation of hemostasis	Biological Process	GO:1900048	0.00486
regulation of protein localization to synapse	Biological Process	GO:1902473	0.00486
regulation of telomere capping	Biological Process	GO:1904353	0.00486
negative regulation of striated muscle cell apoptotic process	Biological Process	GO:0010664	0.00489
triglyceride biosynthetic process	Biological Process	GO:0019432	0.00489
fatty-acyl-CoA metabolic process	Biological Process	GO:0035337	0.00489
positive regulation of viral transcription	Biological Process	GO:0050434	0.00489
regulation of calcium ion import	Biological Process	GO:0090279	0.00489
negative regulation of intracellular protein transport	Biological Process	GO:0090317	0.00489
hexose catabolic process	Biological Process	GO:0019320	0.00489
regulation of insulin secretion involved in cellular response to glucose stimulus	Biological Process	GO:0061178	0.00489
negative regulation of signaling receptor activity	Biological Process	GO:2000272	0.00489
sensory perception of sound	Biological Process	GO:0007605	0.00489
zymogen activation	Biological Process	GO:0031638	0.00497
synaptic transmission, GABAergic	Biological Process	GO:0051932	0.00499
regulation of membrane depolarization	Biological Process	GO:0003254	0.00501
selective autophagy	Biological Process	GO:0061912	0.00501
amine transport	Biological Process	GO:0015837	0.00507
excitatory postsynaptic potential	Biological Process	GO:0060079	0.00507
water homeostasis	Biological Process	GO:0030104	0.00515
epithelial cell maturation	Biological Process	GO:0002070	0.00534

regulation of tolerance induction	Biological Process	GO:0002643	0.00534
regulation of cytokine secretion involved in immune response	Biological Process	GO:0002739	0.00534
ventricular trabecula myocardium morphogenesis	Biological Process	GO:0003222	0.00534
serotonin transport	Biological Process	GO:0006837	0.00534
establishment or maintenance of cytoskeleton polarity	Biological Process	GO:0030952	0.00534
hair follicle maturation	Biological Process	GO:0048820	0.00534
regulation of transcription involved in cell fate commitment	Biological Process	GO:0060850	0.00534
chaperone-mediated autophagy	Biological Process	GO:0061684	0.00534
metanephric glomerulus development	Biological Process	GO:0072224	0.00534
cellular response to thyroid hormone stimulus	Biological Process	GO:0097067	0.00534
negative regulation of intrinsic apoptotic signaling pathway in response to DNA damage by p53 class mediator	Biological Process	GO:1902166	0.00534
negative regulation of amyloid precursor protein catabolic process	Biological Process	GO:1902992	0.00534
positive regulation of sodium ion transmembrane transporter activity	Biological Process	GO:2000651	0.00534
positive regulation of neuron migration	Biological Process	GO:2001224	0.00534
DNA conformation change	Biological Process	GO:0071103	0.00535
cerebellar cortex morphogenesis	Biological Process	GO:0021696	0.00545
negative regulation of biomineral tissue development	Biological Process	GO:0070168	0.00545
regulation of neutrophil chemotaxis	Biological Process	GO:0090022	0.00545
negative regulation of biomineralization	Biological Process	GO:0110150	0.00545
regulation of hydrogen peroxide-induced cell death	Biological Process	GO:1903205	0.00545
negative regulation of protein localization to membrane	Biological Process	GO:1905476	0.00545
negative regulation of DNA metabolic process	Biological Process	GO:0051053	0.00548
cellular ketone metabolic process	Biological Process	GO:0042180	0.00559
G protein-coupled receptor signaling pathway, coupled to cyclic nucleotide second messenger	Biological Process	GO:0007187	0.00571
membranous septum morphogenesis	Biological Process	GO:0003149	0.00571
forebrain neuron fate commitment	Biological Process	GO:0021877	0.00571
positive regulation of fatty acid beta-oxidation	Biological Process	GO:0032000	0.00571
response to cobalt ion	Biological Process	GO:0032025	0.00571
regulation of deoxyribonuclease activity	Biological Process	GO:0032070	0.00571
glomerular basement membrane development	Biological Process	GO:0032836	0.00571
regulation of DNA endoreduplication	Biological Process	GO:0032875	0.00571
cortisol metabolic process	Biological Process	GO:0034650	0.00571
endosome to melanosome transport	Biological Process	GO:0035646	0.00571
nail development	Biological Process	GO:0035878	0.00571

tetrahydrofolate interconversion	Biological Process	GO:0035999	0.00571
collagen-activated tyrosine kinase receptor signaling pathway	Biological Process	GO:0038063	0.00571
endosome to pigment granule transport	Biological Process	GO:0043485	0.00571
relaxation of smooth muscle	Biological Process	GO:0044557	0.00571
low-density lipoprotein particle receptor biosynthetic process	Biological Process	GO:0045713	0.00571
regulation of photoreceptor cell differentiation	Biological Process	GO:0046532	0.00571
pigment granule maturation	Biological Process	GO:0048757	0.00571
anatomical structure regression	Biological Process	GO:0060033	0.00571
cardiac muscle cell fate commitment	Biological Process	GO:0060923	0.00571
retina vasculature morphogenesis in camera-type eye	Biological Process	GO:0061299	0.00571
response to interleukin-9	Biological Process	GO:0071104	0.00571
glomerular visceral epithelial cell development	Biological Process	GO:0072015	0.00571
regulation of steroid hormone biosynthetic process	Biological Process	GO:0090030	0.00571
regulation of neuron projection arborization	Biological Process	GO:0150011	0.00571
regulation of histone deacetylase activity	Biological Process	GO:1901725	0.00571
positive regulation of protein localization to synapse	Biological Process	GO:1902474	0.00571
regulation of interleukin-13 secretion	Biological Process	GO:2000665	0.00571
negative regulation of mesenchymal cell apoptotic process	Biological Process	GO:2001054	0.00571
suckling behavior	Biological Process	GO:0001967	0.00571
negative regulation of type 2 immune response	Biological Process	GO:0002829	0.00571
mineralocorticoid biosynthetic process	Biological Process	GO:0006705	0.00571
mineralocorticoid metabolic process	Biological Process	GO:0008212	0.00571
negative regulation of platelet-derived growth factor receptor signaling pathway	Biological Process	GO:0010642	0.00571
spinal cord motor neuron cell fate specification	Biological Process	GO:0021520	0.00571
adenohypophysis development	Biological Process	GO:0021984	0.00571
positive regulation of interleukin-5 production	Biological Process	GO:0032754	0.00571
interleukin-15-mediated signaling pathway	Biological Process	GO:0035723	0.00571
T-helper 2 cell cytokine production	Biological Process	GO:0035745	0.00571
norepinephrine metabolic process	Biological Process	GO:0042415	0.00571
positive regulation of interferon-gamma biosynthetic process	Biological Process	GO:0045078	0.00571
positive regulation of interleukin-2 biosynthetic process	Biological Process	GO:0045086	0.00571
negative regulation of bone resorption	Biological Process	GO:0045779	0.00571
negative regulation of axon regeneration	Biological Process	GO:0048681	0.00571
positive regulation of astrocyte differentiation	Biological Process	GO:0048711	0.00571
myoblast migration	Biological Process	GO:0051451	0.00571
intestinal epithelial cell development	Biological Process	GO:0060576	0.00571

vascular wound healing	Biological Process	GO:0061042	0.00571
negative regulation of cell growth involved in cardiac muscle cell development	Biological Process	GO:0061052	0.00571
bone trabecula morphogenesis	Biological Process	GO:0061430	0.00571
regulation of type B pancreatic cell proliferation	Biological Process	GO:0061469	0.00571
cellular response to interleukin-15	Biological Process	GO:0071350	0.00571
inflammatory response to wounding	Biological Process	GO:0090594	0.00571
mesenchymal cell apoptotic process	Biological Process	GO:0097152	0.00571
L-glutamate import across plasma membrane	Biological Process	GO:0098712	0.00571
regulation of tau-protein kinase activity	Biological Process	GO:1902947	0.00571
regulation of RNA polymerase II regulatory region sequence-specific DNA binding	Biological Process	GO:1903025	0.00571
positive regulation of cytoplasmic transport	Biological Process	GO:1903651	0.00571
regulation of telomere maintenance via telomere lengthening	Biological Process	GO:1904356	0.00574
nucleotide-excision repair, DNA damage recognition	Biological Process	GO:0000715	0.00577
L-glutamate transmembrane transport	Biological Process	GO:0015813	0.00577
male genitalia development	Biological Process	GO:0030539	0.00577
positive regulation of oligodendrocyte differentiation	Biological Process	GO:0048714	0.00577
detection of stimulus involved in sensory perception of pain	Biological Process	GO:0062149	0.00577
amino acid import across plasma membrane	Biological Process	GO:0089718	0.00577
positive regulation of cell aging	Biological Process	GO:0090343	0.00577
mitotic spindle assembly checkpoint	Biological Process	GO:0007094	0.00577
positive regulation of phosphatase activity	Biological Process	GO:0010922	0.00577
Rac protein signal transduction	Biological Process	GO:0016601	0.00577
spindle checkpoint	Biological Process	GO:0031577	0.00577
spindle assembly checkpoint	Biological Process	GO:0071173	0.00577
mitotic spindle checkpoint	Biological Process	GO:0071174	0.00577
regulation of cardiac muscle cell action potential	Biological Process	GO:0098901	0.00577
lipid oxidation	Biological Process	GO:0034440	0.00578
cellular transition metal ion homeostasis	Biological Process	GO:0046916	0.00582
syncytium formation	Biological Process	GO:0006949	0.00591
positive regulation of cellular carbohydrate metabolic process	Biological Process	GO:0010676	0.00591
energy derivation by oxidation of organic compounds	Biological Process	GO:0015980	0.00591
transition metal ion homeostasis	Biological Process	GO:0055076	0.00603
negative regulation of lymphocyte mediated immunity	Biological Process	GO:0002707	0.00603
regulation of protein export from nucleus	Biological Process	GO:0046825	0.00603
positive regulation of interleukin-1 secretion	Biological Process	GO:0050716	0.00603
response to growth hormone	Biological Process	GO:0060416	0.00603

ventricular cardiac muscle cell action potential	Biological Process	GO:0086005	0.00603
negative regulation of DNA biosynthetic process	Biological Process	GO:2000279	0.00603
cell differentiation in spinal cord	Biological Process	GO:0021515	0.00605
regulation of telomere maintenance via telomerase	Biological Process	GO:0032210	0.00605
negative regulation of cellular carbohydrate metabolic process	Biological Process	GO:0010677	0.00617
activation of GTPase activity	Biological Process	GO:0090630	0.00618
glycogen biosynthetic process	Biological Process	GO:0005978	0.0062
glucan biosynthetic process	Biological Process	GO:0009250	0.0062
negative regulation of type I interferon production	Biological Process	GO:0032480	0.0062
regulation of lyase activity	Biological Process	GO:0051339	0.0062
aminoglycan biosynthetic process	Biological Process	GO:0006023	0.00622
heterochromatin organization	Biological Process	GO:0070828	0.00639
acylglycerol metabolic process	Biological Process	GO:0006639	0.00651
regulation of amine transport	Biological Process	GO:0051952	0.00662
startle response	Biological Process	GO:0001964	0.00664
one-carbon metabolic process	Biological Process	GO:0006730	0.00664
response to salt stress	Biological Process	GO:0009651	0.00664
muscle fiber development	Biological Process	GO:0048747	0.00666
positive regulation of T-helper 1 type immune response	Biological Process	GO:0002827	0.00692
asymmetric cell division	Biological Process	GO:0008356	0.00692
positive regulation of CREB transcription factor activity	Biological Process	GO:0032793	0.00692
negative regulation of tissue remodeling	Biological Process	GO:0034104	0.00692
middle ear morphogenesis	Biological Process	GO:0042474	0.00692
negative regulation of nucleocytoplasmic transport	Biological Process	GO:0046823	0.00692
regulation of collateral sprouting	Biological Process	GO:0048670	0.00692
Sertoli cell differentiation	Biological Process	GO:0060008	0.00692
positive regulation of transcription initiation from RNA polymerase II promoter	Biological Process	GO:0060261	0.00692
atrial cardiac muscle cell action potential	Biological Process	GO:0086014	0.00692
atrial cardiac muscle cell to AV node cell signaling	Biological Process	GO:0086026	0.00692
atrial cardiac muscle cell to AV node cell communication	Biological Process	GO:0086066	0.00692
dendritic spine maintenance	Biological Process	GO:0097062	0.00692
response to mitochondrial depolarisation	Biological Process	GO:0098780	0.00692
regulation of nuclear-transcribed mRNA catabolic process, deadenylation-dependent decay	Biological Process	GO:1900151	0.00692
positive regulation of autophagy of mitochondrion	Biological Process	GO:1903599	0.00692
positive regulation of macrophage migration	Biological Process	GO:1905523	0.00692

positive regulation of nervous system process	Biological Process	GO:0031646	0.00694
drug transport	Biological Process	GO:0015893	0.00697
adenylate cyclase-inhibiting G protein-coupled receptor signaling pathway	Biological Process	GO:0007193	0.00704
regulation of calcium ion transmembrane transporter activity	Biological Process	GO:1901019	0.00704
regulation of smoothened signaling pathway	Biological Process	GO:0008589	0.00718
protein demethylation	Biological Process	GO:0006482	0.00722
female meiotic nuclear division	Biological Process	GO:0007143	0.00722
protein dealkylation	Biological Process	GO:0008214	0.00722
G2 DNA damage checkpoint	Biological Process	GO:0031572	0.00722
positive regulation of T cell migration	Biological Process	GO:2000406	0.00722
regulation of hematopoietic progenitor cell differentiation	Biological Process	GO:1901532	0.00752
positive regulation of striated muscle cell differentiation	Biological Process	GO:0051155	0.00759
COPII-coated vesicle budding	Biological Process	GO:0090114	0.00759
regulation of glycogen metabolic process	Biological Process	GO:0070873	0.00762
chromatin silencing	Biological Process	GO:0006342	0.00768
cellular detoxification	Biological Process	GO:1990748	0.0077
organic acid transmembrane transport	Biological Process	GO:1903825	0.00774
carboxylic acid transmembrane transport	Biological Process	GO:1905039	0.00774
dopamine metabolic process	Biological Process	GO:0042417	0.00781
positive regulation of dendrite morphogenesis	Biological Process	GO:0050775	0.00781
fat-soluble vitamin metabolic process	Biological Process	GO:0006775	0.00787
establishment of spindle localization	Biological Process	GO:0051293	0.00787
presynapse assembly	Biological Process	GO:0099054	0.00787
positive regulation of cell killing	Biological Process	GO:0031343	0.008
determination of adult lifespan	Biological Process	GO:0008340	0.00815
response to UV-B	Biological Process	GO:0010224	0.00815
positive regulation of macrophage derived foam cell differentiation	Biological Process	GO:0010744	0.00815
response to caffeine	Biological Process	GO:0031000	0.00815
response to diuretic	Biological Process	GO:0036270	0.00815
regulation of interferon-gamma biosynthetic process	Biological Process	GO:0045072	0.00815
positive regulation of glycogen biosynthetic process	Biological Process	GO:0045725	0.00815
anatomical structure arrangement	Biological Process	GO:0048532	0.00815
postsynaptic neurotransmitter receptor internalization	Biological Process	GO:0098884	0.00815
modification of postsynaptic structure	Biological Process	GO:0099010	0.00815
postsynaptic endocytosis	Biological Process	GO:0140239	0.00815
positive regulation of telomere capping	Biological Process	GO:1904355	0.00815

dendritic cell chemotaxis	Biological Process	GO:0002407	0.00815
regulation of cardiac muscle contraction by regulation of the release of sequestered calcium ion	Biological Process	GO:0010881	0.00815
subpallium development	Biological Process	GO:0021544	0.00815
forebrain regionalization	Biological Process	GO:0021871	0.00815
cerebral cortex neuron differentiation	Biological Process	GO:0021895	0.00815
membrane raft organization	Biological Process	GO:0031579	0.00815
regulation of activin receptor signaling pathway	Biological Process	GO:0032925	0.00815
cellular response to sterol	Biological Process	GO:0036315	0.00815
trabecula formation	Biological Process	GO:0060343	0.00815
regulation of cellular response to vascular endothelial growth factor stimulus	Biological Process	GO:1902547	0.00815
regulation of protein polyubiquitination	Biological Process	GO:1902914	0.00815
regulation of glycolytic process	Biological Process	GO:0006110	0.00848
regulation of transcription from RNA polymerase II promoter in response to hypoxia	Biological Process	GO:0061418	0.00848
antibiotic metabolic process	Biological Process	GO:0016999	0.00859
chromatin organization involved in negative regulation of transcription	Biological Process	GO:0097549	0.00859
maintenance of protein location in cell	Biological Process	GO:0032507	0.00877
myofibril assembly	Biological Process	GO:0030239	0.00902
regulation of interleukin-6 biosynthetic process	Biological Process	GO:0045408	0.00904
positive regulation of G protein-coupled receptor signaling pathway	Biological Process	GO:0045745	0.00904
regulation of adenylate cyclase activity	Biological Process	GO:0045761	0.00904
RNA polymerase II preinitiation complex assembly	Biological Process	GO:0051123	0.00904
protein localization to chromatin	Biological Process	GO:0071168	0.00904
regulation of leukocyte adhesion to vascular endothelial cell	Biological Process	GO:1904994	0.00904
positive regulation of excitatory postsynaptic potential	Biological Process	GO:2000463	0.00904
syncytium formation by plasma membrane fusion	Biological Process	GO:0000768	0.00913
mitotic spindle assembly	Biological Process	GO:0090307	0.00913
cell-cell fusion	Biological Process	GO:0140253	0.00913
hydrogen peroxide-mediated programmed cell death	Biological Process	GO:0010421	0.00934
negative regulation of mitochondrial membrane potential	Biological Process	GO:0010917	0.00934
regulation of Rac protein signal transduction	Biological Process	GO:0035020	0.00934
positive regulation of urine volume	Biological Process	GO:0035810	0.00934
negative regulation of epidermal cell differentiation	Biological Process	GO:0045605	0.00934
negative regulation of natural killer cell mediated cytotoxicity	Biological Process	GO:0045953	0.00934
organelle inheritance	Biological Process	GO:0048308	0.00934
Golgi inheritance	Biological Process	GO:0048313	0.00934

Golgi localization	Biological Process	GO:0051645	0.00934
L-glutamate import	Biological Process	GO:0051938	0.00934
growth hormone receptor signaling pathway via JAK-STAT	Biological Process	GO:0060397	0.00934
programmed cell death in response to reactive oxygen species	Biological Process	GO:0097468	0.00934
regulation of hepatocyte proliferation	Biological Process	GO:2000345	0.00934
ATP biosynthetic process	Biological Process	GO:0006754	0.00943
cellular defense response	Biological Process	GO:0006968	0.00943
long-chain fatty acid metabolic process	Biological Process	GO:0001676	0.00945
ribonucleoside triphosphate biosynthetic process	Biological Process	GO:0009201	0.0095
positive regulation of phagocytosis	Biological Process	GO:0050766	0.0095
monosaccharide biosynthetic process	Biological Process	GO:0046364	0.00953
positive regulation of synaptic transmission, glutamatergic	Biological Process	GO:0051968	0.00955
regulation of cardiac muscle cell membrane repolarization	Biological Process	GO:0099623	0.00955
positive regulation of p38MAPK cascade	Biological Process	GO:1900745	0.00955
neuromuscular junction development	Biological Process	GO:0007528	0.00967
spindle localization	Biological Process	GO:0051653	0.00967
vascular endothelial cell proliferation	Biological Process	GO:0101023	0.00967
regulation of vascular endothelial cell proliferation	Biological Process	GO:1905562	0.00967
negative regulation of ubiquitin-dependent protein catabolic process	Biological Process	GO:2000059	0.00967
aminoglycan metabolic process	Biological Process	GO:0006022	0.00973
detoxification	Biological Process	GO:0098754	0.00979
glucose catabolic process	Biological Process	GO:0006007	0.00983
dopamine receptor signaling pathway	Biological Process	GO:0007212	0.00984
regulation of natural killer cell mediated cytotoxicity	Biological Process	GO:0042269	0.00984
regulation of endocrine process	Biological Process	GO:0044060	0.00984
regulation of dendritic spine morphogenesis	Biological Process	GO:0061001	0.00984
regulation of T cell migration	Biological Process	GO:2000404	0.00984
regulation of triglyceride metabolic process	Biological Process	GO:0090207	0.0099
regulation of protein localization to cell surface	Biological Process	GO:2000008	0.0099
nucleotide-excision repair, preincision complex stabilization	Biological Process	GO:0006293	0.00994
nucleotide-excision repair, DNA incision, 3'-to lesion	Biological Process	GO:0006295	0.00994
regulation of hydrogen peroxide metabolic process	Biological Process	GO:0010310	0.00994
chromatin disassembly	Biological Process	GO:0031498	0.00994
negative regulation of cardiac muscle cell proliferation	Biological Process	GO:0060044	0.00994
positive regulation of triglyceride metabolic process	Biological Process	GO:0090208	0.00994
positive regulation of calcium ion import	Biological Process	GO:0090280	0.00994

positive regulation of long-term synaptic potentiation	Biological Process	GO:1900273	0.00994
positive regulation of sodium ion transmembrane transport	Biological Process	GO:1902307	0.00994
positive regulation of extracellular matrix organization	Biological Process	GO:1903055	0.00994
regulation of mesoderm development	Biological Process	GO:2000380	0.00994
negative regulation of transcription regulatory region DNA binding	Biological Process	GO:2000678	0.00994
polysaccharide metabolic process	Biological Process	GO:0005976	0.00994
aminoglycan catabolic process	Biological Process	GO:0006026	0.00997
multicellular organismal signaling	Biological Process	GO:0035637	0.01
Golgi organization	Biological Process	GO:0007030	0.01
activation of MAPKKK activity	Biological Process	GO:0000185	0.01
T cell tolerance induction	Biological Process	GO:0002517	0.01
negative regulation of immunoglobulin production	Biological Process	GO:0002638	0.01
regulation of dendritic cell cytokine production	Biological Process	GO:0002730	0.01
activation of JNKK activity	Biological Process	GO:0007256	0.01
response to gravity	Biological Process	GO:0009629	0.01
dorsal/ventral axis specification	Biological Process	GO:0009950	0.01
bleb assembly	Biological Process	GO:0032060	0.01
response to peptidoglycan	Biological Process	GO:0032494	0.01
negative regulation of protein sumoylation	Biological Process	GO:0033234	0.01
regulation of cell-cell adhesion mediated by integrin	Biological Process	GO:0033632	0.01
DNA endoreduplication	Biological Process	GO:0042023	0.01
positive regulation of odontogenesis	Biological Process	GO:0042482	0.01
negative regulation of histone H3-K9 methylation	Biological Process	GO:0051573	0.01
positive regulation of histone H3-K9 methylation	Biological Process	GO:0051574	0.01
dopamine uptake involved in synaptic transmission	Biological Process	GO:0051583	0.01
catecholamine uptake involved in synaptic transmission	Biological Process	GO:0051934	0.01
negative regulation of telomerase activity	Biological Process	GO:0051974	0.01
cardiac muscle cell myoblast differentiation	Biological Process	GO:0060379	0.01
response to fungicide	Biological Process	GO:0060992	0.01
interleukin-35-mediated signaling pathway	Biological Process	GO:0070757	0.01
positive regulation of podosome assembly	Biological Process	GO:0071803	0.01
glomerular epithelial cell development	Biological Process	GO:0072310	0.01
caveolin-mediated endocytosis	Biological Process	GO:0072584	0.01
interleukin-13 secretion	Biological Process	GO:0072611	0.01
T cell extravasation	Biological Process	GO:0072683	0.01
tubulin deacetylation	Biological Process	GO:0090042	0.01

regulation of postsynaptic neurotransmitter receptor internalization	Biological Process	GO:0099149	0.01
response to L-glutamate	Biological Process	GO:1902065	0.01
protein localization to early endosome	Biological Process	GO:1902946	0.01
positive regulation of glial cell migration	Biological Process	GO:1903977	0.01
regulation of aspartic-type peptidase activity	Biological Process	GO:1905245	0.01
regulation of mesoderm formation	Biological Process	GO:1905902	0.01
regulation of lamellipodium morphogenesis	Biological Process	GO:2000392	0.01
regulation of T-helper 2 cell cytokine production	Biological Process	GO:2000551	0.01
regulation of potassium ion transport	Biological Process	GO:0043266	0.01
cellular aldehyde metabolic process	Biological Process	GO:0006081	0.0104
mechanoreceptor differentiation	Biological Process	GO:0042490	0.0104
DNA duplex unwinding	Biological Process	GO:0032508	0.0107
feeding behavior	Biological Process	GO:0007631	0.0108
regulation of plasma lipoprotein particle levels	Biological Process	GO:0097006	0.0108
hydrogen peroxide metabolic process	Biological Process	GO:0042743	0.0109
plasminogen activation	Biological Process	GO:0031639	0.0111
ionotropic glutamate receptor signaling pathway	Biological Process	GO:0035235	0.0111
vesicle cargo loading	Biological Process	GO:0035459	0.0111
regulation of endoplasmic reticulum unfolded protein response	Biological Process	GO:1900101	0.0111
specification of symmetry	Biological Process	GO:0009799	0.0114
negative regulation of interleukin-6 production	Biological Process	GO:0032715	0.0114
cellular response to virus	Biological Process	GO:0098586	0.0114
L-alpha-amino acid transmembrane transport	Biological Process	GO:1902475	0.0114
regulation of synapse assembly	Biological Process	GO:0051963	0.0115
inorganic cation import across plasma membrane	Biological Process	GO:0098659	0.0117
inorganic ion import across plasma membrane	Biological Process	GO:0099587	0.0117
negative regulation of leukocyte mediated immunity	Biological Process	GO:0002704	0.0118
regulation of systemic arterial blood pressure mediated by a chemical signal	Biological Process	GO:0003044	0.0118
vesicle targeting, rough ER to cis-Golgi	Biological Process	GO:0048207	0.0118
COPII vesicle coating	Biological Process	GO:0048208	0.0118
regulation of postsynaptic membrane neurotransmitter receptor levels	Biological Process	GO:0099072	0.0118
nitric oxide mediated signal transduction	Biological Process	GO:0007263	0.0119
establishment of epithelial cell polarity	Biological Process	GO:0090162	0.0119
negative regulation of leukocyte mediated cytotoxicity	Biological Process	GO:0001911	0.012
negative regulation of myotube differentiation	Biological Process	GO:0010832	0.012
establishment of apical/basal cell polarity	Biological Process	GO:0035089	0.012

cellular response to leptin stimulus	Biological Process	GO:0044320	0.012
negative regulation of epidermis development	Biological Process	GO:0045683	0.012
embryonic digestive tract morphogenesis	Biological Process	GO:0048557	0.012
negative regulation of lipase activity	Biological Process	GO:0060192	0.012
positive regulation of glycogen metabolic process	Biological Process	GO:0070875	0.012
regulation of intrinsic apoptotic signaling pathway in response to DNA damage by p53 class mediator	Biological Process	GO:1902165	0.012
positive regulation of fatty acid transport	Biological Process	GO:2000193	0.012
digestion	Biological Process	GO:0007586	0.0122
methylation	Biological Process	GO:0032259	0.0122
protein kinase A signaling	Biological Process	GO:0010737	0.0123
deoxyribose phosphate metabolic process	Biological Process	GO:0019692	0.0123
establishment of mitotic spindle localization	Biological Process	GO:0040001	0.0123
alpha-beta T cell proliferation	Biological Process	GO:0046633	0.0123
spleen development	Biological Process	GO:0048536	0.0123
positive regulation of ERBB signaling pathway	Biological Process	GO:1901186	0.0123
DNA catabolic process	Biological Process	GO:0006308	0.0123
forelimb morphogenesis	Biological Process	GO:0035136	0.0123
ruffle assembly	Biological Process	GO:0097178	0.0123
modulation of excitatory postsynaptic potential	Biological Process	GO:0098815	0.0123
regulation of extracellular matrix organization	Biological Process	GO:1903053	0.0123
cellular oxidant detoxification	Biological Process	GO:0098869	0.0124
nuclear-transcribed mRNA poly(A) tail shortening	Biological Process	GO:0000289	0.0124
secretion by tissue	Biological Process	GO:0032941	0.0124
regulation of cofactor metabolic process	Biological Process	GO:0051193	0.0124
regulation of mitotic spindle organization	Biological Process	GO:0060236	0.0124
toxin transport	Biological Process	GO:1901998	0.0124
release of sequestered calcium ion into cytosol by endoplasmic reticulum	Biological Process	GO:1903514	0.0124
regulation of myotube differentiation	Biological Process	GO:0010830	0.0124
negative regulation of intracellular transport	Biological Process	GO:0032387	0.0124
negative regulation of protein kinase B signaling	Biological Process	GO:0051898	0.0124
vesicle targeting	Biological Process	GO:0006903	0.0125
sulfur compound biosynthetic process	Biological Process	GO:0044272	0.0126
nucleus organization	Biological Process	GO:0006997	0.0127
antigen receptor-mediated signaling pathway	Biological Process	GO:0050851	0.0127
regulation of centrosome cycle	Biological Process	GO:0046605	0.0131

regulation of RNA splicing	Biological Process	GO:0043484	0.0132
protein targeting to mitochondrion	Biological Process	GO:0006626	0.0134
calcium ion import	Biological Process	GO:0070509	0.0134
exocytic process	Biological Process	GO:0140029	0.0134
negative regulation of transporter activity	Biological Process	GO:0032410	0.0135
regulation of postsynaptic membrane potential	Biological Process	GO:0060078	0.0136
unsaturated fatty acid biosynthetic process	Biological Process	GO:0006636	0.0138
protein trimerization	Biological Process	GO:0070206	0.0138
mitophagy	Biological Process	GO:0000423	0.0138
mating behavior	Biological Process	GO:0007617	0.0138
cerebral cortex radial glia guided migration	Biological Process	GO:0021801	0.0138
hypothalamus development	Biological Process	GO:0021854	0.0138
central nervous system myelination	Biological Process	GO:0022010	0.0138
telencephalon glial cell migration	Biological Process	GO:0022030	0.0138
axon ensheathment in central nervous system	Biological Process	GO:0032291	0.0138
type B pancreatic cell proliferation	Biological Process	GO:0044342	0.0138
positive regulation of myoblast differentiation	Biological Process	GO:0045663	0.0138
cAMP metabolic process	Biological Process	GO:0046058	0.0138
negative regulation of phagocytosis	Biological Process	GO:0050765	0.0138
positive regulation of signal transduction by p53 class mediator	Biological Process	GO:1901798	0.0138
germ cell development	Biological Process	GO:0007281	0.0138
centrosome duplication	Biological Process	GO:0051298	0.0139
organelle localization by membrane tethering	Biological Process	GO:0140056	0.0141
negative regulation of natural killer cell mediated immunity	Biological Process	GO:0002716	0.0142
tripartite regional subdivision	Biological Process	GO:0007351	0.0142
anterior/posterior axis specification, embryo	Biological Process	GO:0008595	0.0142
protein nitrosylation	Biological Process	GO:0017014	0.0142
peptidyl-cysteine S-nitrosylation	Biological Process	GO:0018119	0.0142
Rap protein signal transduction	Biological Process	GO:0032486	0.0142
positive regulation of mast cell activation involved in immune response	Biological Process	GO:0033008	0.0142
V(D)J recombination	Biological Process	GO:0033151	0.0142
erythrocyte maturation	Biological Process	GO:0043249	0.0142
positive regulation of mast cell degranulation	Biological Process	GO:0043306	0.0142
positive regulation of DNA damage response, signal transduction by p53 class mediator	Biological Process	GO:0043517	0.0142
nose development	Biological Process	GO:0043584	0.0142
locomotor rhythm	Biological Process	GO:0045475	0.0142

negative regulation of membrane potential	Biological Process	GO:0045837	0.0142
regulation of RNA polymerase II transcription preinitiation complex assembly	Biological Process	GO:0045898	0.0142
regulation of developmental pigmentation	Biological Process	GO:0048070	0.0142
negative regulation of astrocyte differentiation	Biological Process	GO:0048712	0.0142
prepulse inhibition	Biological Process	GO:0060134	0.0142
regulation of podosome assembly	Biological Process	GO:0071801	0.0142
regulation of calcium ion transmembrane transport via high voltage-gated calcium channel	Biological Process	GO:1902514	0.0142
regulation of plasma membrane organization	Biological Process	GO:1903729	0.0142
cellular pigmentation	Biological Process	GO:0033059	0.0143
cellular iron ion homeostasis	Biological Process	GO:0006879	0.0148
cAMP-mediated signaling	Biological Process	GO:0019933	0.0148
chemical synaptic transmission, postsynaptic	Biological Process	GO:0099565	0.0148
regulation of natural killer cell mediated immunity	Biological Process	GO:0002715	0.0149
mitochondrion localization	Biological Process	GO:0051646	0.0149
presynapse organization	Biological Process	GO:0099172	0.0149
response to amino acid starvation	Biological Process	GO:1990928	0.0149
regulation of odontogenesis	Biological Process	GO:0042481	0.0149
amino acid import	Biological Process	GO:0043090	0.0149
cardiac myofibril assembly	Biological Process	GO:0055003	0.0149
regulation of ryanodine-sensitive calcium-release channel activity	Biological Process	GO:0060314	0.0149
response to thyroid hormone	Biological Process	GO:0097066	0.0149
positive regulation of actin filament polymerization	Biological Process	GO:0030838	0.0152
negative regulation of axon extension	Biological Process	GO:0030517	0.0153
regulation of mast cell activation	Biological Process	GO:0033003	0.0153
synaptic vesicle transport	Biological Process	GO:0048489	0.0153
cardiac muscle cell membrane repolarization	Biological Process	GO:0099622	0.0153
regulation of neuron migration	Biological Process	GO:2001222	0.0153
positive regulation of cation channel activity	Biological Process	GO:2001259	0.0153
chondrocyte differentiation involved in endochondral bone morphogenesis	Biological Process	GO:0003413	0.0155
reproductive behavior	Biological Process	GO:0019098	0.0155
long-chain fatty acid biosynthetic process	Biological Process	GO:0042759	0.0155
regulation of alpha-beta T cell proliferation	Biological Process	GO:0046640	0.0155
negative regulation of receptor signaling pathway via STAT	Biological Process	GO:1904893	0.0155
regulation of presynapse assembly	Biological Process	GO:1905606	0.0155
activation of adenylate cyclase activity	Biological Process	GO:0007190	0.0156

chaperone-mediated protein folding	Biological Process	GO:0061077	0.0156
DNA catabolic process, endonucleolytic	Biological Process	GO:0000737	0.0156
positive regulation of sodium ion transport	Biological Process	GO:0010765	0.0156
positive regulation of organic acid transport	Biological Process	GO:0032892	0.0156
negative regulation of exocytosis	Biological Process	GO:0045920	0.0156
adrenergic receptor signaling pathway	Biological Process	GO:0071875	0.0156
energy homeostasis	Biological Process	GO:0097009	0.0156
ER overload response	Biological Process	GO:0006983	0.0164
layer formation in cerebral cortex	Biological Process	GO:0021819	0.0164
establishment or maintenance of actin cytoskeleton polarity	Biological Process	GO:0030950	0.0164
pigment accumulation	Biological Process	GO:0043476	0.0164
cellular pigment accumulation	Biological Process	GO:0043482	0.0164
positive regulation of B cell differentiation	Biological Process	GO:0045579	0.0164
radial glial cell differentiation	Biological Process	GO:0060019	0.0164
negative regulation of pathway-restricted SMAD protein phosphorylation	Biological Process	GO:0060394	0.0164
trophoblast cell migration	Biological Process	GO:0061450	0.0164
centromeric sister chromatid cohesion	Biological Process	GO:0070601	0.0164
response to parathyroid hormone	Biological Process	GO:0071107	0.0164
cellular response to electrical stimulus	Biological Process	GO:0071257	0.0164
distal tubule development	Biological Process	GO:0072017	0.0164
T-helper 17 cell lineage commitment	Biological Process	GO:0072540	0.0164
inhibition of cysteine-type endopeptidase activity	Biological Process	GO:0097340	0.0164
zymogen inhibition	Biological Process	GO:0097341	0.0164
modification of postsynaptic actin cytoskeleton	Biological Process	GO:0098885	0.0164
regulation of trophoblast cell migration	Biological Process	GO:1901163	0.0164
regulation of hydrogen peroxide-mediated programmed cell death	Biological Process	GO:1901298	0.0164
positive regulation of membrane depolarization	Biological Process	GO:1904181	0.0164
regulation of protein localization to endosome	Biological Process	GO:1905666	0.0164
positive regulation of DNA-dependent DNA replication	Biological Process	GO:2000105	0.0164
negative regulation of sodium ion transmembrane transporter activity	Biological Process	GO:2000650	0.0164
regulation of mesenchymal cell apoptotic process	Biological Process	GO:2001053	0.0164
regulation of dendritic cell differentiation	Biological Process	GO:2001198	0.0164
icosanoid biosynthetic process	Biological Process	GO:0046456	0.0164
retina morphogenesis in camera-type eye	Biological Process	GO:0060042	0.0164
synaptic vesicle localization	Biological Process	GO:0097479	0.0164
negative regulation of cellular protein catabolic process	Biological Process	GO:1903363	0.0165

catecholamine transport	Biological Process	GO:0051937	0.0165
protein polyubiquitination	Biological Process	GO:0000209	0.0168
negative regulation of calcium ion transport into cytosol	Biological Process	GO:0010523	0.0169
positive regulation of striated muscle cell apoptotic process	Biological Process	GO:0010663	0.0169
positive regulation of cardiac muscle cell apoptotic process	Biological Process	GO:0010666	0.0169
regulation of icosanoid secretion	Biological Process	GO:0032303	0.0169
negative regulation of interleukin-17 production	Biological Process	GO:0032700	0.0169
SNARE complex assembly	Biological Process	GO:0035493	0.0169
T-helper 1 cell differentiation	Biological Process	GO:0045063	0.0169
parasympathetic nervous system development	Biological Process	GO:0048486	0.0169
cell migration involved in heart development	Biological Process	GO:0060973	0.0169
regulation of phospholipid biosynthetic process	Biological Process	GO:0071071	0.0169
podosome assembly	Biological Process	GO:0071800	0.0169
positive regulation of actin cytoskeleton reorganization	Biological Process	GO:2000251	0.0169
determination of bilateral symmetry	Biological Process	GO:0009855	0.0171
arachidonic acid metabolic process	Biological Process	GO:0019369	0.0172
icosanoid metabolic process	Biological Process	GO:0006690	0.0173
amino acid transmembrane transport	Biological Process	GO:0003333	0.0178
gluconeogenesis	Biological Process	GO:0006094	0.0178
protein sumoylation	Biological Process	GO:0016925	0.0178
regulation of TOR signaling	Biological Process	GO:0032006	0.018
bile acid metabolic process	Biological Process	GO:0008206	0.018
neuron maturation	Biological Process	GO:0042551	0.018
purine ribonucleoside metabolic process	Biological Process	GO:0046128	0.0184
axonal fasciculation	Biological Process	GO:0007413	0.0186
negative regulation of smooth muscle cell differentiation	Biological Process	GO:0051151	0.0186
positive regulation of lyase activity	Biological Process	GO:0051349	0.0186
mitochondrial depolarization	Biological Process	GO:0051882	0.0186
negative regulation of cell cycle arrest	Biological Process	GO:0071157	0.0186
regulation of mitochondrial fission	Biological Process	GO:0090140	0.0186
cell aggregation	Biological Process	GO:0098743	0.0186
neuron projection fasciculation	Biological Process	GO:0106030	0.0186
regulation of vascular endothelial growth factor signaling pathway	Biological Process	GO:1900746	0.0186
negative regulation of response to reactive oxygen species	Biological Process	GO:1901032	0.0186
negative regulation of hydrogen peroxide-induced cell death	Biological Process	GO:1903206	0.0186
regulation of myoblast proliferation	Biological Process	GO:2000291	0.0186

substantia nigra development	Biological Process	GO:0021762	0.0187
sarcoplasmic reticulum calcium ion transport	Biological Process	GO:0070296	0.0187
axo-dendritic transport	Biological Process	GO:0008088	0.019
polysaccharide biosynthetic process	Biological Process	GO:0000271	0.0191
negative regulation of ion transmembrane transporter activity	Biological Process	GO:0032413	0.0191
cytokinetic process	Biological Process	GO:0032506	0.0193
negative regulation of interleukin-1 production	Biological Process	GO:0032692	0.0193
regulation of T cell receptor signaling pathway	Biological Process	GO:0050856	0.0193
acylglycerol homeostasis	Biological Process	GO:0055090	0.0193
triglyceride homeostasis	Biological Process	GO:0070328	0.0193
NADH regeneration	Biological Process	GO:0006735	0.0195
dendritic cell migration	Biological Process	GO:0036336	0.0195
negative regulation of muscle contraction	Biological Process	GO:0045932	0.0195
animal organ maturation	Biological Process	GO:0048799	0.0195
regulation of ventricular cardiac muscle cell membrane repolarization	Biological Process	GO:0060307	0.0195
innervation	Biological Process	GO:0060384	0.0195
canonical glycolysis	Biological Process	GO:0061621	0.0195
glucose catabolic process to pyruvate	Biological Process	GO:0061718	0.0195
cellular response to copper ion	Biological Process	GO:0071280	0.0195
positive regulation of leukocyte mediated cytotoxicity	Biological Process	GO:0001912	0.0195
inner ear receptor cell differentiation	Biological Process	GO:0060113	0.0195
double-strand break repair via homologous recombination	Biological Process	GO:0000724	0.0196
mitochondrial fission	Biological Process	GO:0000266	0.0197
deoxyribonucleotide metabolic process	Biological Process	GO:0009262	0.0197
positive regulation of amine transport	Biological Process	GO:0051954	0.0197
regulation of oxidative phosphorylation	Biological Process	GO:0002082	0.0197
proximal/distal pattern formation	Biological Process	GO:0009954	0.0197
regulation of mast cell degranulation	Biological Process	GO:0043304	0.0197
regulation of amino acid transport	Biological Process	GO:0051955	0.0197
walking behavior	Biological Process	GO:0090659	0.0197
regulation of presynapse organization	Biological Process	GO:0099174	0.0197
ventricular cardiac muscle cell membrane repolarization	Biological Process	GO:0099625	0.0197
endoplasmic reticulum to Golgi vesicle-mediated transport	Biological Process	GO:0006888	0.0198
vesicle targeting, to, from or within Golgi	Biological Process	GO:0048199	0.0205
dopamine transport	Biological Process	GO:0015872	0.0206
response to UV-C	Biological Process	GO:0010225	0.0207

regulation of ketone biosynthetic process	Biological Process	GO:0010566	0.0207
positive regulation of icosanoid secretion	Biological Process	GO:0032305	0.0207
negative regulation of myeloid cell apoptotic process	Biological Process	GO:0033033	0.0207
positive regulation by host of viral process	Biological Process	GO:0044794	0.0207
modulation by host of viral genome replication	Biological Process	GO:0044827	0.0207
negative regulation of smooth muscle contraction	Biological Process	GO:0045986	0.0207
sequestering of metal ion	Biological Process	GO:0051238	0.0207
negative regulation of ubiquitin-protein transferase activity	Biological Process	GO:0051444	0.0207
positive regulation of histone H3-K4 methylation	Biological Process	GO:0051571	0.0207
renal absorption	Biological Process	GO:0070293	0.0207
ureter development	Biological Process	GO:0072189	0.0207
G protein-coupled receptor signaling pathway involved in heart process	Biological Process	GO:0086103	0.0207
regulation of synaptic vesicle endocytosis	Biological Process	GO:1900242	0.0207
positive regulation of glutamate receptor signaling pathway	Biological Process	GO:1900451	0.0207
negative regulation of endothelial cell migration	Biological Process	GO:0010596	0.021
ventral spinal cord development	Biological Process	GO:0021517	0.0217
recombinational repair	Biological Process	GO:0000725	0.0218
vesicle coating	Biological Process	GO:0006901	0.0221
localization within membrane	Biological Process	GO:0051668	0.0228
cartilage condensation	Biological Process	GO:0001502	0.0234
positive regulation of mast cell activation	Biological Process	GO:0033005	0.0234
positive regulation of cell adhesion mediated by integrin	Biological Process	GO:0033630	0.0234
regulation of toll-like receptor 4 signaling pathway	Biological Process	GO:0034143	0.0234
regulation of hair follicle development	Biological Process	GO:0051797	0.0234
establishment of monopolar cell polarity	Biological Process	GO:0061162	0.0234
cellular response to cholesterol	Biological Process	GO:0071397	0.0234
connective tissue replacement	Biological Process	GO:0097709	0.0234
regulation of mitotic spindle assembly	Biological Process	GO:1901673	0.0234
positive regulation of cellular senescence	Biological Process	GO:2000774	0.0234
negative regulation of calcium ion transport	Biological Process	GO:0051926	0.0238
negative regulation of adaptive immune response based on somatic recombination of immune receptors built from immunoglobulin superfamily domains	Biological Process	GO:0002823	0.0238
protein targeting to vacuole	Biological Process	GO:0006623	0.0238
protein refolding	Biological Process	GO:0042026	0.0238
regulation of digestive system process	Biological Process	GO:0044058	0.0238
clathrin-dependent endocytosis	Biological Process	GO:0072583	0.0238

blastocyst development	Biological Process	GO:0001824	0.0238
renal water homeostasis	Biological Process	GO:0003091	0.0246
release of sequestered calcium ion into cytosol by sarcoplasmic reticulum	Biological Process	GO:0014808	0.0246
positive regulation of regulated secretory pathway	Biological Process	GO:1903307	0.0247
cytoskeleton-dependent intracellular transport	Biological Process	GO:0030705	0.0247
sex determination	Biological Process	GO:0007530	0.0247
vitamin biosynthetic process	Biological Process	GO:0009110	0.0247
protein localization to endosome	Biological Process	GO:0036010	0.0247
exogenous drug catabolic process	Biological Process	GO:0042738	0.0247
positive regulation of leukocyte degranulation	Biological Process	GO:0043302	0.0247
positive regulation of endothelial cell differentiation	Biological Process	GO:0045603	0.0247
positive regulation of epidermal cell differentiation	Biological Process	GO:0045606	0.0247
positive regulation of antigen receptor-mediated signaling pathway	Biological Process	GO:0050857	0.0247
neurotransmitter receptor internalization	Biological Process	GO:0099590	0.0247
positive regulation of glycoprotein metabolic process	Biological Process	GO:1903020	0.0247
cellular polysaccharide metabolic process	Biological Process	GO:0044264	0.0248
regulation of transcription by RNA polymerase I	Biological Process	GO:0006356	0.0249
attachment of spindle microtubules to kinetochore	Biological Process	GO:0008608	0.0249
regulation of release of sequestered calcium ion into cytosol by sarcoplasmic reticulum	Biological Process	GO:0010880	0.0249
regulation of mast cell activation involved in immune response	Biological Process	GO:0033006	0.0249
viral RNA genome replication	Biological Process	GO:0039694	0.0249
cell-cell signaling involved in cardiac conduction	Biological Process	GO:0086019	0.0249
membrane raft assembly	Biological Process	GO:0001765	0.0249
endothelial cell morphogenesis	Biological Process	GO:0001886	0.0249
detection of calcium ion	Biological Process	GO:0005513	0.0249
cellular response to nitrogen starvation	Biological Process	GO:0006995	0.0249
response to herbicide	Biological Process	GO:0009635	0.0249
UV protection	Biological Process	GO:0009650	0.0249
negative regulation of keratinocyte proliferation	Biological Process	GO:0010839	0.0249
Cdc42 protein signal transduction	Biological Process	GO:0032488	0.0249
negative regulation of tyrosine phosphorylation of STAT protein	Biological Process	GO:0042532	0.0249
positive regulation of hair cycle	Biological Process	GO:0042635	0.0249
cellular response to nitrogen levels	Biological Process	GO:0043562	0.0249
negative regulation of endothelial cell differentiation	Biological Process	GO:0045602	0.0249
negative regulation of glycolytic process	Biological Process	GO:0045820	0.0249
regulation of interleukin-1 beta biosynthetic process	Biological Process	GO:0050722	0.0249

detection of mechanical stimulus involved in sensory perception of pain	Biological Process	GO:0050966	0.0249
positive regulation of type I interferon-mediated signaling pathway	Biological Process	GO:0060340	0.0249
response to platelet aggregation inhibitor	Biological Process	GO:0061478	0.0249
protein heterotrimerization	Biological Process	GO:0070208	0.0249
UV-damage excision repair	Biological Process	GO:0070914	0.0249
semaphorin-plexin signaling pathway involved in neuron projection guidance	Biological Process	GO:1902285	0.0249
negative regulation of sodium ion transmembrane transport	Biological Process	GO:1902306	0.0249
negative regulation of amyloid-beta formation	Biological Process	GO:1902430	0.0249
regulation of interferon-gamma secretion	Biological Process	GO:1902713	0.0249
positive regulation of autophagy of mitochondrion in response to mitochondrial depolarization	Biological Process	GO:1904925	0.0249
positive regulation of cellular response to drug	Biological Process	GO:2001040	0.0249
androgen metabolic process	Biological Process	GO:0008209	0.025
negative regulation of mRNA processing	Biological Process	GO:0050686	0.025
positive regulation of chromosome segregation	Biological Process	GO:0051984	0.025
glycolytic process through fructose-6-phosphate	Biological Process	GO:0061615	0.025
glycolytic process through glucose-6-phosphate	Biological Process	GO:0061620	0.025
negative regulation of anion transport	Biological Process	GO:1903792	0.025
regulation of vascular smooth muscle cell differentiation	Biological Process	GO:1905063	0.025
potassium ion transport	Biological Process	GO:0006813	0.025
regulation of ATPase activity	Biological Process	GO:0043462	0.0251
regulation of multi-organism process	Biological Process	GO:0043900	0.0254
regulation of behavior	Biological Process	GO:0050795	0.0254
cellular monovalent inorganic cation homeostasis	Biological Process	GO:0030004	0.0254
protein homotetramerization	Biological Process	GO:0051289	0.0256
transport along microtubule	Biological Process	GO:0010970	0.0258
T cell mediated cytotoxicity	Biological Process	GO:0001913	0.0259
regulation of meiotic cell cycle	Biological Process	GO:0051445	0.0259
icosanoid transport	Biological Process	GO:0071715	0.0259
fatty acid derivative transport	Biological Process	GO:1901571	0.0259
regulation of glycoprotein metabolic process	Biological Process	GO:1903018	0.0259
hexose biosynthetic process	Biological Process	GO:0019319	0.0265
negative regulation of endothelial cell proliferation	Biological Process	GO:0001937	0.0271
L-amino acid transport	Biological Process	GO:0015807	0.0274
monosaccharide catabolic process	Biological Process	GO:0046365	0.0274
negative regulation of adaptive immune response	Biological Process	GO:0002820	0.0274

intracellular lipid transport	Biological Process	GO:0032365	0.0274
mRNA catabolic process	Biological Process	GO:0006402	0.0278
iron ion homeostasis	Biological Process	GO:0055072	0.0288
protein tetramerization	Biological Process	GO:0051262	0.0289
myoblast fusion	Biological Process	GO:0007520	0.0289
regulation of spindle organization	Biological Process	GO:0090224	0.0289
regulation of execution phase of apoptosis	Biological Process	GO:1900117	0.0289
G protein-coupled receptor internalization	Biological Process	GO:0002031	0.0291
N-terminal protein amino acid acetylation	Biological Process	GO:0006474	0.0291
thyroid hormone generation	Biological Process	GO:0006590	0.0291
NADPH regeneration	Biological Process	GO:0006740	0.0291
regulation of T cell chemotaxis	Biological Process	GO:0010819	0.0291
ventral spinal cord interneuron differentiation	Biological Process	GO:0021514	0.0291
preganglionic parasympathetic fiber development	Biological Process	GO:0021783	0.0291
positive regulation of myelination	Biological Process	GO:0031643	0.0291
linoleic acid metabolic process	Biological Process	GO:0043651	0.0291
lamellipodium morphogenesis	Biological Process	GO:0072673	0.0291
negative regulation of proteolysis involved in cellular protein catabolic process	Biological Process	GO:1903051	0.0291
natural killer cell proliferation	Biological Process	GO:0001787	0.0291
neural plate development	Biological Process	GO:0001840	0.0291
substrate-dependent cell migration, cell extension	Biological Process	GO:0006930	0.0291
Notch receptor processing	Biological Process	GO:0007220	0.0291
folic acid-containing compound biosynthetic process	Biological Process	GO:0009396	0.0291
miRNA catabolic process	Biological Process	GO:0010587	0.0291
regulation of neuron maturation	Biological Process	GO:0014041	0.0291
peristalsis	Biological Process	GO:0030432	0.0291
negative regulation of hormone biosynthetic process	Biological Process	GO:0032353	0.0291
oligopeptide transmembrane transport	Biological Process	GO:0035672	0.0291
cellular response to potassium ion	Biological Process	GO:0035865	0.0291
ATF6-mediated unfolded protein response	Biological Process	GO:0036500	0.0291
drinking behavior	Biological Process	GO:0042756	0.0291
sequestering of actin monomers	Biological Process	GO:0042989	0.0291
memory T cell differentiation	Biological Process	GO:0043379	0.0291
positive regulation of monocyte differentiation	Biological Process	GO:0045657	0.0291
behavioral response to ethanol	Biological Process	GO:0048149	0.0291
notochord morphogenesis	Biological Process	GO:0048570	0.0291

embryonic viscerocranium morphogenesis	Biological Process	GO:0048703	0.0291
response to folic acid	Biological Process	GO:0051593	0.0291
positive regulation of mitochondrial depolarization	Biological Process	GO:0051901	0.0291
bone trabecula formation	Biological Process	GO:0060346	0.0291
dichotomous subdivision of an epithelial terminal unit	Biological Process	GO:0060600	0.0291
regulation of chemokine-mediated signaling pathway	Biological Process	GO:0070099	0.0291
otic vesicle morphogenesis	Biological Process	GO:0071600	0.0291
chemokine (C-C motif) ligand 5 production	Biological Process	GO:0071609	0.0291
regulation of cell proliferation in bone marrow	Biological Process	GO:0071863	0.0291
interleukin-12 secretion	Biological Process	GO:0072610	0.0291
SA node cell to atrial cardiac muscle cell communication	Biological Process	GO:0086070	0.0291
immunological memory formation process	Biological Process	GO:0090715	0.0291
dendritic cell apoptotic process	Biological Process	GO:0097048	0.0291
complement-dependent cytotoxicity	Biological Process	GO:0097278	0.0291
positive regulation of receptor binding	Biological Process	GO:1900122	0.0291
positive regulation of mitophagy	Biological Process	GO:1901526	0.0291
positive regulation of endoplasmic reticulum stress-induced intrinsic apoptotic signaling pathway	Biological Process	GO:1902237	0.0291
regulation of microtubule binding	Biological Process	GO:1904526	0.0291
neuron projection maintenance	Biological Process	GO:1990535	0.0291
regulation of macrophage apoptotic process	Biological Process	GO:2000109	0.0291
negative regulation of T-helper 17 type immune response	Biological Process	GO:2000317	0.0291
regulation of miRNA metabolic process	Biological Process	GO:2000628	0.0291
regulation of dendritic cell apoptotic process	Biological Process	GO:2000668	0.0291
purine nucleoside metabolic process	Biological Process	GO:0042278	0.0292
determination of left/right symmetry	Biological Process	GO:0007368	0.0298
nucleotide-excision repair, DNA incision, 5'-to lesion	Biological Process	GO:0006296	0.0298
positive regulation of blood pressure	Biological Process	GO:0045777	0.0298
membrane depolarization during action potential	Biological Process	GO:0086010	0.0298
postsynaptic signal transduction	Biological Process	GO:0098926	0.0298
negative regulation of reactive oxygen species biosynthetic process	Biological Process	GO:1903427	0.0298
regulation of double-strand break repair	Biological Process	GO:2000779	0.0308
protein-DNA complex assembly	Biological Process	GO:0065004	0.0309
positive regulation of myeloid leukocyte mediated immunity	Biological Process	GO:0002888	0.0309
negative regulation of macroautophagy	Biological Process	GO:0016242	0.0309
cGMP-mediated signaling	Biological Process	GO:0019934	0.0309

negative regulation of interleukin-1 beta production	Biological Process	GO:0032691	0.0309
embryonic forelimb morphogenesis	Biological Process	GO:0035115	0.0309
positive regulation of neurotransmitter secretion	Biological Process	GO:0001956	0.0309
desensitization of G protein-coupled receptor signaling pathway	Biological Process	GO:0002029	0.0309
acute inflammatory response to antigenic stimulus	Biological Process	GO:0002438	0.0309
cerebellar cortex formation	Biological Process	GO:0021697	0.0309
negative adaptation of signaling pathway	Biological Process	GO:0022401	0.0309
negative regulation of organic acid transport	Biological Process	GO:0032891	0.0309
thyroid hormone metabolic process	Biological Process	GO:0042403	0.0309
deoxyribose phosphate catabolic process	Biological Process	GO:0046386	0.0309
regulation of mitochondrial depolarization	Biological Process	GO:0051900	0.0309
establishment or maintenance of monopolar cell polarity	Biological Process	GO:0061339	0.0309
histone H3-K27 methylation	Biological Process	GO:0070734	0.0309
protein localization to chromosome, centromeric region	Biological Process	GO:0071459	0.0309
regulation of fibroblast apoptotic process	Biological Process	GO:2000269	0.0309
establishment of mitotic spindle orientation	Biological Process	GO:0000132	0.0315
nucleotide-excision repair, preincision complex assembly	Biological Process	GO:0006294	0.0315
adult walking behavior	Biological Process	GO:0007628	0.0315
nucleobase-containing small molecule interconversion	Biological Process	GO:0015949	0.0315
cerebral cortex radially oriented cell migration	Biological Process	GO:0021799	0.0315
regulation of B cell receptor signaling pathway	Biological Process	GO:0050855	0.0315
negative regulation of protein tyrosine kinase activity	Biological Process	GO:0061099	0.0315
regulation of ruffle assembly	Biological Process	GO:1900027	0.0315
renal system process involved in regulation of systemic arterial blood pressure	Biological Process	GO:0003071	0.0316
long-chain fatty-acyl-CoA metabolic process	Biological Process	GO:0035336	0.0316
leukocyte tethering or rolling	Biological Process	GO:0050901	0.0316
adenylate cyclase-activating G protein-coupled receptor signaling pathway	Biological Process	GO:0007189	0.0319
eye photoreceptor cell differentiation	Biological Process	GO:0001754	0.0324
heterophilic cell-cell adhesion via plasma membrane cell adhesion molecules	Biological Process	GO:0007157	0.0324
regulation of leukocyte degranulation	Biological Process	GO:0043300	0.0324
negative regulation of blood pressure	Biological Process	GO:0045776	0.0324
regulation of cardiac conduction	Biological Process	GO:1903779	0.0333
telomere capping	Biological Process	GO:0016233	0.0336
regulation of fatty acid biosynthetic process	Biological Process	GO:0042304	0.0336
receptor clustering	Biological Process	GO:0043113	0.0336
cellular component maintenance	Biological Process	GO:0043954	0.0336

base-excision repair	Biological Process	GO:0006284	0.0345
immunological synapse formation	Biological Process	GO:0001771	0.0359
leukocyte migration involved in inflammatory response	Biological Process	GO:0002523	0.0359
Golgi to vacuole transport	Biological Process	GO:0006896	0.0359
histone H3-K9 demethylation	Biological Process	GO:0033169	0.0359
genitalia morphogenesis	Biological Process	GO:0035112	0.0359
cellular response to increased oxygen levels	Biological Process	GO:0036295	0.0359
angiotensin-activated signaling pathway	Biological Process	GO:0038166	0.0359
regulation of odontogenesis of dentin-containing tooth	Biological Process	GO:0042487	0.0359
negative regulation of centrosome cycle	Biological Process	GO:0046606	0.0359
interleukin-1 beta biosynthetic process	Biological Process	GO:0050720	0.0359
positive regulation of T cell receptor signaling pathway	Biological Process	GO:0050862	0.0359
chloride ion homeostasis	Biological Process	GO:0055064	0.0359
positive regulation of cardiac muscle contraction	Biological Process	GO:0060452	0.0359
positive regulation of T cell apoptotic process	Biological Process	GO:0070234	0.0359
cellular response to estrogen stimulus	Biological Process	GO:0071391	0.0359
signal transduction involved in G2 DNA damage checkpoint	Biological Process	GO:0072425	0.0359
COPII-coated vesicle cargo loading	Biological Process	GO:0090110	0.0359
activation of cysteine-type endopeptidase activity	Biological Process	GO:0097202	0.0359
presynaptic modulation of chemical synaptic transmission	Biological Process	GO:0099171	0.0359
regulation of adenylate cyclase-activating G protein-coupled receptor signaling pathway	Biological Process	GO:0106070	0.0359
negative regulation of receptor binding	Biological Process	GO:1900121	0.0359
regulation of DNA catabolic process	Biological Process	GO:1903624	0.0359
regulation of autophagy of mitochondrion in response to mitochondrial depolarization	Biological Process	GO:1904923	0.0359
negative regulation of vascular smooth muscle cell differentiation	Biological Process	GO:1905064	0.0359
regulation of vasculogenesis	Biological Process	GO:2001212	0.0359
plasma lipoprotein particle clearance	Biological Process	GO:0034381	0.036
DNA synthesis involved in DNA repair	Biological Process	GO:0000731	0.036
negative regulation of cardiac muscle cell apoptotic process	Biological Process	GO:0010667	0.0362
regulation of necrotic cell death	Biological Process	GO:0010939	0.0362
autophagosome maturation	Biological Process	GO:0097352	0.0362
positive regulation of calcium ion transmembrane transporter activity	Biological Process	GO:1901021	0.0362
negative regulation of blood circulation	Biological Process	GO:1903523	0.0362
regulation of protein depolymerization	Biological Process	GO:1901879	0.0366
neuron cellular homeostasis	Biological Process	GO:0070050	0.038
neuromuscular process	Biological Process	GO:0050905	0.0382

protein localization to vacuole	Biological Process	GO:0072665	0.039
endosomal transport	Biological Process	GO:0016197	0.0391
protein K48-linked ubiquitination	Biological Process	GO:0070936	0.0393
negative regulation of adenylate cyclase activity	Biological Process	GO:0007194	0.0393
deoxyribonucleoside triphosphate metabolic process	Biological Process	GO:0009200	0.0393
striatum development	Biological Process	GO:0021756	0.0393
transcytosis	Biological Process	GO:0045056	0.0393
negative regulation of cell division	Biological Process	GO:0051782	0.0393
establishment of protein localization to telomere	Biological Process	GO:0070200	0.0393
negative regulation of purine nucleotide metabolic process	Biological Process	GO:1900543	0.0393
detection of biotic stimulus	Biological Process	GO:0009595	0.0394
mitotic G2/M transition checkpoint	Biological Process	GO:0044818	0.0394
positive regulation of smoothened signaling pathway	Biological Process	GO:0045880	0.0394
retinoic acid receptor signaling pathway	Biological Process	GO:0048384	0.0394
regulation of defense response to virus by virus	Biological Process	GO:0050690	0.0394
postsynapse assembly	Biological Process	GO:0099068	0.0394
endosome organization	Biological Process	GO:0007032	0.0398
C21-steroid hormone biosynthetic process	Biological Process	GO:0006700	0.0403
exploration behavior	Biological Process	GO:0035640	0.0403
regulation of insulin-like growth factor receptor signaling pathway	Biological Process	GO:0043567	0.0403
pigment granule organization	Biological Process	GO:0048753	0.0403
global genome nucleotide-excision repair	Biological Process	GO:0070911	0.0403
basement membrane organization	Biological Process	GO:0071711	0.0403
nucleotide-excision repair, DNA duplex unwinding	Biological Process	GO:0000717	0.0404
ectoderm development	Biological Process	GO:0007398	0.0404
positive regulation of lipid storage	Biological Process	GO:0010884	0.0404
adaptation of signaling pathway	Biological Process	GO:0023058	0.0404
synaptic vesicle fusion to presynaptic active zone membrane	Biological Process	GO:0031629	0.0404
regulation of urine volume	Biological Process	GO:0035809	0.0404
steroid hormone secretion	Biological Process	GO:0035929	0.0404
ubiquitin-dependent protein catabolic process via the multivesicular body sorting pathway	Biological Process	GO:0043162	0.0404
positive regulation of transcription by RNA polymerase I	Biological Process	GO:0045943	0.0404
interferon-gamma secretion	Biological Process	GO:0072643	0.0404
regulation of pH	Biological Process	GO:0006885	0.0404
RNA catabolic process	Biological Process	GO:0006401	0.0404

cellular response to amino acid starvation	Biological Process	GO:0034198	0.041
organophosphate catabolic process	Biological Process	GO:0046434	0.0413
protein folding	Biological Process	GO:0006457	0.0418
small molecule catabolic process	Biological Process	GO:0044282	0.042
cellular lipid catabolic process	Biological Process	GO:0044242	0.0422
postreplication repair	Biological Process	GO:0006301	0.0422
spindle assembly	Biological Process	GO:0051225	0.0423
muscle filament sliding	Biological Process	GO:0030049	0.0437
actin-myosin filament sliding	Biological Process	GO:0033275	0.0437
nucleotide-excision repair, DNA incision	Biological Process	GO:0033683	0.0437
DNA damage response, detection of DNA damage	Biological Process	GO:0042769	0.0437
regulation of dendritic cell antigen processing and presentation	Biological Process	GO:0002604	0.0441
oligopeptide transport	Biological Process	GO:0006857	0.0441
response to muscle inactivity	Biological Process	GO:0014870	0.0441
facial nerve development	Biological Process	GO:0021561	0.0441
facial nerve morphogenesis	Biological Process	GO:0021610	0.0441
negative regulation of hormone metabolic process	Biological Process	GO:0032351	0.0441
detection of molecule of bacterial origin	Biological Process	GO:0032490	0.0441
positive regulation of protein sumoylation	Biological Process	GO:0033235	0.0441
Leydig cell differentiation	Biological Process	GO:0033327	0.0441
negative regulation of heterotypic cell-cell adhesion	Biological Process	GO:0034115	0.0441
negative thymic T cell selection	Biological Process	GO:0045060	0.0441
positive regulation of RNA polymerase II transcription preinitiation complex assembly	Biological Process	GO:0045899	0.0441
regulation of skeletal muscle fiber development	Biological Process	GO:0048742	0.0441
forebrain morphogenesis	Biological Process	GO:0048853	0.0441
limb bud formation	Biological Process	GO:0060174	0.0441
embryonic skeletal joint morphogenesis	Biological Process	GO:0060272	0.0441
establishment of blood-brain barrier	Biological Process	GO:0060856	0.0441
cardiac vascular smooth muscle cell differentiation	Biological Process	GO:0060947	0.0441
interleukin-27-mediated signaling pathway	Biological Process	GO:0070106	0.0441
regulation of cell-cell adhesion involved in gastrulation	Biological Process	GO:0070587	0.0441
cell proliferation in bone marrow	Biological Process	GO:0071838	0.0441
fibroblast activation	Biological Process	GO:0072537	0.0441
positive regulation of glomerulus development	Biological Process	GO:0090193	0.0441
synapse pruning	Biological Process	GO:0098883	0.0441
atrial cardiac muscle cell membrane repolarization	Biological Process	GO:0099624	0.0441

extracellular regulation of signal transduction	Biological Process	GO:1900115	0.0441
extracellular negative regulation of signal transduction	Biological Process	GO:1900116	0.0441
regulation of relaxation of muscle	Biological Process	GO:1901077	0.0441
response to forskolin	Biological Process	GO:1904321	0.0441
cellular response to forskolin	Biological Process	GO:1904322	0.0441
protein localization to presynapse	Biological Process	GO:1905383	0.0441
positive regulation of protein localization to endosome	Biological Process	GO:1905668	0.0441
positive regulation of gonad development	Biological Process	GO:1905941	0.0441
positive regulation of T-helper 17 type immune response	Biological Process	GO:2000318	0.0441
protein monoubiquitination	Biological Process	GO:0006513	0.0444
secondary metabolic process	Biological Process	GO:0019748	0.0444
dicarboxylic acid metabolic process	Biological Process	GO:0043648	0.0449
positive regulation of potassium ion transport	Biological Process	GO:0043268	0.0451
regulation of cAMP-mediated signaling	Biological Process	GO:0043949	0.0451
regulation of myeloid leukocyte mediated immunity	Biological Process	GO:0002886	0.0453
post-Golgi vesicle-mediated transport	Biological Process	GO:0006892	0.0459
establishment of spindle orientation	Biological Process	GO:0051294	0.046
regulation of renal system process	Biological Process	GO:0098801	0.046
regulation of voltage-gated calcium channel activity	Biological Process	GO:1901385	0.046
immunoglobulin production	Biological Process	GO:0002377	0.0461
cellular modified amino acid metabolic process	Biological Process	GO:0006575	0.0462
meiotic cell cycle process	Biological Process	GO:1903046	0.0462
negative regulation of viral life cycle	Biological Process	GO:1903901	0.0462
meiotic cell cycle	Biological Process	GO:0051321	0.0463
2'-deoxyribonucleotide metabolic process	Biological Process	GO:0009394	0.0483
regulation of vesicle fusion	Biological Process	GO:0031338	0.0483
response to ATP	Biological Process	GO:0033198	0.0483
multivesicular body organization	Biological Process	GO:0036257	0.0483
positive regulation of epidermal growth factor receptor signaling pathway	Biological Process	GO:0045742	0.0483
adenylate cyclase-activating adrenergic receptor signaling pathway	Biological Process	GO:0071880	0.0483
membrane repolarization during action potential	Biological Process	GO:0086011	0.0483
regulation of viral release from host cell	Biological Process	GO:1902186	0.0483
positive regulation of double-strand break repair	Biological Process	GO:2000781	0.0483
peptidyl-lysine trimethylation	Biological Process	GO:0018023	0.0485
transcription preinitiation complex assembly	Biological Process	GO:0070897	0.0485
heterochromatin assembly	Biological Process	GO:0031507	0.0486

nuclear export	Biological Process	GO:0051168	0.0495
positive regulation of receptor recycling	Biological Process	GO:0001921	0.0495
pentose-phosphate shunt	Biological Process	GO:0006098	0.0495
positive regulation of cell fate commitment	Biological Process	GO:0010455	0.0495
positive regulation of macrophage chemotaxis	Biological Process	GO:0010759	0.0495
cell proliferation in external granule layer	Biological Process	GO:0021924	0.0495
cerebellar granule cell precursor proliferation	Biological Process	GO:0021930	0.0495
gonadotropin secretion	Biological Process	GO:0032274	0.0495
cell migration involved in gastrulation	Biological Process	GO:0042074	0.0495
regulation of sulfur metabolic process	Biological Process	GO:0042762	0.0495
dicarboxylic acid biosynthetic process	Biological Process	GO:0043650	0.0495
regulation of interleukin-1 biosynthetic process	Biological Process	GO:0045360	0.0495
positive regulation of membrane potential	Biological Process	GO:0045838	0.0495
detection of temperature stimulus involved in sensory perception	Biological Process	GO:0050961	0.0495
detection of temperature stimulus involved in sensory perception of pain	Biological Process	GO:0050965	0.0495
positive regulation of membrane protein ectodomain proteolysis	Biological Process	GO:0051044	0.0495
ventral spinal cord interneuron fate commitment	Biological Process	GO:0060579	0.0495
cell fate commitment involved in pattern specification	Biological Process	GO:0060581	0.0495
positive regulation of mitochondrial fission	Biological Process	GO:0090141	0.0495
positive regulation of chemokine secretion	Biological Process	GO:0090197	0.0495
positive regulation of execution phase of apoptosis	Biological Process	GO:1900119	0.0495
regulation of high voltage-gated calcium channel activity	Biological Process	GO:1901841	0.0495
regulation of Golgi organization	Biological Process	GO:1903358	0.0495
protein export from nucleus	Biological Process	GO:0006611	0.0499
focal adhesion	Cellular Component	GO:0005925	1.95E-38
cell-substrate junction	Cellular Component	GO:0030055	1.95E-38
transcription regulator complex	Cellular Component	GO:0005667	1.85E-37
membrane raft	Cellular Component	GO:0045121	5.64E-30
membrane microdomain	Cellular Component	GO:0098857	6.84E-30
membrane region	Cellular Component	GO:0098589	4.40E-29
nuclear chromatin	Cellular Component	GO:0000790	9.10E-28
cell leading edge	Cellular Component	GO:0031252	5.35E-21
transferase complex, transferring phosphorus-containing groups	Cellular Component	GO:0061695	6.08E-19
RNA polymerase II transcription regulator complex	Cellular Component	GO:0090575	4.09E-18
early endosome	Cellular Component	GO:0005769	3.72E-16
neuronal cell body	Cellular Component	GO:0043025	1.32E-15

cell-cell junction	Cellular Component	GO:0005911	6.81E-15
endocytic vesicle	Cellular Component	GO:0030139	8.74E-15
glutamatergic synapse	Cellular Component	GO:0098978	1.28E-14
protein kinase complex	Cellular Component	GO:1902911	1.48E-14
collagen-containing extracellular matrix	Cellular Component	GO:0062023	5.94E-14
serine/threonine protein kinase complex	Cellular Component	GO:1902554	1.09E-13
apical part of cell	Cellular Component	GO:0045177	6.83E-13
ruffle	Cellular Component	GO:0001726	1.55E-12
lamellipodium	Cellular Component	GO:0030027	1.06E-11
basolateral plasma membrane	Cellular Component	GO:0016323	1.31E-11
cyclin-dependent protein kinase holoenzyme complex	Cellular Component	GO:0000307	1.94E-11
cell projection membrane	Cellular Component	GO:0031253	2.66E-11
dendritic spine	Cellular Component	GO:0043197	9.38E-11
growth cone	Cellular Component	GO:0030426	1.14E-10
neuron spine	Cellular Component	GO:0044309	1.66E-10
vesicle lumen	Cellular Component	GO:0031983	1.70E-10
endosome membrane	Cellular Component	GO:0010008	2.44E-10
cytoplasmic vesicle lumen	Cellular Component	GO:0060205	2.54E-10
lateral plasma membrane	Cellular Component	GO:0016328	2.92E-10
apical plasma membrane	Cellular Component	GO:0016324	3.28E-10
site of polarized growth	Cellular Component	GO:0030427	4.06E-10
cell cortex	Cellular Component	GO:0005938	5.51E-10
plasma membrane raft	Cellular Component	GO:0044853	1.20E-09
secretory granule lumen	Cellular Component	GO:0034774	1.27E-09
phosphatidylinositol 3-kinase complex	Cellular Component	GO:0005942	1.27E-09
melanosome	Cellular Component	GO:0042470	1.31E-09
pigment granule	Cellular Component	GO:0048770	1.31E-09
caveola	Cellular Component	GO:0005901	2.24E-09
filopodium	Cellular Component	GO:0030175	2.26E-09
endoplasmic reticulum lumen	Cellular Component	GO:0005788	7.17E-09
cytoplasmic ribonucleoprotein granule	Cellular Component	GO:0036464	7.65E-09
actin-based cell projection	Cellular Component	GO:0098858	7.65E-09
distal axon	Cellular Component	GO:0150034	9.88E-09
transcription repressor complex	Cellular Component	GO:0017053	1.04E-08
leading edge membrane	Cellular Component	GO:0031256	1.28E-08
ribonucleoprotein granule	Cellular Component	GO:0035770	1.30E-08

outer membrane	Cellular Component	GO:0019867	1.48E-08
sarcolemma	Cellular Component	GO:0042383	1.61E-08
organelle outer membrane	Cellular Component	GO:0031968	2.34E-08
external side of plasma membrane	Cellular Component	GO:0009897	5.02E-08
mitochondrial outer membrane	Cellular Component	GO:0005741	8.79E-08
secretory granule membrane	Cellular Component	GO:0030667	9.56E-08
cytoplasmic stress granule	Cellular Component	GO:0010494	1.15E-07
asymmetric synapse	Cellular Component	GO:0032279	1.60E-07
basement membrane	Cellular Component	GO:0005604	1.66E-07
postsynaptic density	Cellular Component	GO:0014069	1.68E-07
presynapse	Cellular Component	GO:0098793	1.70E-07
PML body	Cellular Component	GO:0016605	1.77E-07
coated vesicle	Cellular Component	GO:0030135	1.82E-07
cytoplasmic side of plasma membrane	Cellular Component	GO:0009898	2.23E-07
neuron to neuron synapse	Cellular Component	GO:0098984	2.65E-07
chromosomal region	Cellular Component	GO:0098687	3.33E-07
ruffle membrane	Cellular Component	GO:0032587	3.66E-07
postsynaptic specialization	Cellular Component	GO:0099572	3.78E-07
cytoplasmic side of membrane	Cellular Component	GO:0098562	4.83E-07
bicellular tight junction	Cellular Component	GO:0005923	7.23E-07
nuclear periphery	Cellular Component	GO:0034399	7.25E-07
apical junction complex	Cellular Component	GO:0043296	7.25E-07
tight junction	Cellular Component	GO:0070160	9.26E-07
midbody	Cellular Component	GO:0030496	9.53E-07
actomyosin	Cellular Component	GO:0042641	1.02E-06
nuclear envelope	Cellular Component	GO:0005635	1.06E-06
SWI/SNF superfamily-type complex	Cellular Component	GO:0070603	1.23E-06
clathrin-coated pit	Cellular Component	GO:0005905	1.27E-06
platelet alpha granule	Cellular Component	GO:0031091	1.35E-06
ATPase complex	Cellular Component	GO:1904949	1.36E-06
clathrin-coated vesicle	Cellular Component	GO:0030136	1.44E-06
histone deacetylase complex	Cellular Component	GO:0000118	1.77E-06
filopodium membrane	Cellular Component	GO:0031527	2.39E-06
cell-cell contact zone	Cellular Component	GO:0044291	2.52E-06
nuclear matrix	Cellular Component	GO:0016363	2.84E-06
transport vesicle	Cellular Component	GO:0030133	2.97E-06

extrinsic component of membrane	Cellular Component	GO:0019898	3.34E-06
basal part of cell	Cellular Component	GO:0045178	3.35E-06
nuclear membrane	Cellular Component	GO:0031965	3.77E-06
spindle	Cellular Component	GO:0005819	3.77E-06
contractile fiber	Cellular Component	GO:0043292	4.64E-06
Wnt signalosome	Cellular Component	GO:1990909	6.08E-06
platelet alpha granule lumen	Cellular Component	GO:0031093	7.37E-06
endocytic vesicle membrane	Cellular Component	GO:0030666	7.78E-06
basal plasma membrane	Cellular Component	GO:0009925	1.01E-05
phagocytic vesicle	Cellular Component	GO:0045335	1.44E-05
early endosome membrane	Cellular Component	GO:0031901	1.48E-05
ficolin-1-rich granule	Cellular Component	GO:0101002	1.48E-05
ficolin-1-rich granule lumen	Cellular Component	GO:1904813	1.48E-05
chromosome, telomeric region	Cellular Component	GO:0000781	1.60E-05
nuclear speck	Cellular Component	GO:0016607	1.60E-05
myofibril	Cellular Component	GO:0030016	1.60E-05
stress fiber	Cellular Component	GO:0001725	2.38E-05
contractile actin filament bundle	Cellular Component	GO:0097517	2.38E-05
coated vesicle membrane	Cellular Component	GO:0030662	2.40E-05
immunological synapse	Cellular Component	GO:0001772	2.65E-05
actin filament bundle	Cellular Component	GO:0032432	2.73E-05
nuclear chromosome, telomeric region	Cellular Component	GO:0000784	2.82E-05
actin filament	Cellular Component	GO:0005884	3.04E-05
late endosome	Cellular Component	GO:0005770	3.08E-05
intercalated disc	Cellular Component	GO:0014704	3.24E-05
PcG protein complex	Cellular Component	GO:0031519	3.90E-05
complex of collagen trimers	Cellular Component	GO:0098644	4.49E-05
protein complex involved in cell adhesion	Cellular Component	GO:0098636	4.56E-05
sarcomere	Cellular Component	GO:0030017	4.56E-05
cleavage furrow	Cellular Component	GO:0032154	5.38E-05
clathrin-coated vesicle membrane	Cellular Component	GO:0030665	7.24E-05
beta-catenin destruction complex	Cellular Component	GO:0030877	8.12E-05
cell division site	Cellular Component	GO:0032153	9.66E-05
specific granule	Cellular Component	GO:0042581	0.000106
NuRD complex	Cellular Component	GO:0016581	0.000128
CHD-type complex	Cellular Component	GO:0090545	0.000128

exocytic vesicle	Cellular Component	GO:0070382	0.000143
intrinsic component of mitochondrial outer membrane	Cellular Component	GO:0031306	0.000172
inclusion body	Cellular Component	GO:0016234	0.000173
synaptic vesicle	Cellular Component	GO:0008021	2.00E-04
microvillus	Cellular Component	GO:0005902	0.000221
extrinsic component of plasma membrane	Cellular Component	GO:0019897	0.000239
myelin sheath	Cellular Component	GO:0043209	0.000282
recycling endosome	Cellular Component	GO:0055037	0.000324
synaptic membrane	Cellular Component	GO:0097060	0.000327
CD40 receptor complex	Cellular Component	GO:0035631	0.000331
fibrillar center	Cellular Component	GO:0001650	0.000361
aggresome	Cellular Component	GO:0016235	0.000432
Schaffer collateral - CA1 synapse	Cellular Component	GO:0098685	0.000447
vacuolar membrane	Cellular Component	GO:0005774	0.000517
specific granule membrane	Cellular Component	GO:0035579	0.000522
integral component of mitochondrial outer membrane	Cellular Component	GO:0031307	0.000539
lysosomal membrane	Cellular Component	GO:0005765	0.000616
vacuolar lumen	Cellular Component	GO:0005775	0.000673
lytic vacuole membrane	Cellular Component	GO:0098852	0.000673
heterochromatin	Cellular Component	GO:0000792	0.000727
npBAF complex	Cellular Component	GO:0071564	0.000781
spindle pole	Cellular Component	GO:0000922	0.000787
primary lysosome	Cellular Component	GO:0005766	0.000789
azurophil granule	Cellular Component	GO:0042582	0.000789
protein serine/threonine phosphatase complex	Cellular Component	GO:0008287	0.000789
phosphatase complex	Cellular Component	GO:1903293	0.000789
glial cell projection	Cellular Component	GO:0097386	0.000803
postsynaptic membrane	Cellular Component	GO:0045211	0.000841
euchromatin	Cellular Component	GO:0000791	0.000872
DNA polymerase complex	Cellular Component	GO:0042575	0.000872
chromosome, centromeric region	Cellular Component	GO:0000775	0.000877
integrin complex	Cellular Component	GO:0008305	0.000887
cortical cytoskeleton	Cellular Component	GO:0030863	0.000945
organelle subcompartment	Cellular Component	GO:0031984	0.000994
phagophore assembly site	Cellular Component	GO:0000407	0.00131
mast cell granule	Cellular Component	GO:0042629	0.00143

sarcoplasm	Cellular Component	GO:0016528	0.00162
phagophore assembly site membrane	Cellular Component	GO:0034045	0.00162
ESC/E(Z) complex	Cellular Component	GO:0035098	0.00162
invadopodium	Cellular Component	GO:0071437	0.00162
autophagosome	Cellular Component	GO:0005776	0.00166
Golgi lumen	Cellular Component	GO:0005796	0.00175
cortical actin cytoskeleton	Cellular Component	GO:0030864	0.00215
Golgi apparatus subcompartment	Cellular Component	GO:0098791	0.00224
podosome	Cellular Component	GO:0002102	0.00226
nuclear euchromatin	Cellular Component	GO:0005719	0.00226
endoplasmic reticulum-Golgi intermediate compartment	Cellular Component	GO:0005793	0.00227
Golgi-associated vesicle	Cellular Component	GO:0005798	0.00255
trans-Golgi network	Cellular Component	GO:0005802	0.00258
tertiary granule	Cellular Component	GO:0070820	0.00258
fibrillar collagen trimer	Cellular Component	GO:0005583	0.00261
banded collagen fibril	Cellular Component	GO:0098643	0.00261
apical dendrite	Cellular Component	GO:0097440	0.00271
DNA repair complex	Cellular Component	GO:1990391	0.00289
presynaptic membrane	Cellular Component	GO:0042734	0.00318
sarcoplasmic reticulum	Cellular Component	GO:0016529	0.00331
lipid droplet	Cellular Component	GO:0005811	0.00441
COP9 signalosome	Cellular Component	GO:0008180	0.00477
histone methyltransferase complex	Cellular Component	GO:0035097	0.00491
integral component of presynaptic membrane	Cellular Component	GO:0099056	0.00491
T-tubule	Cellular Component	GO:0030315	0.00491
pronucleus	Cellular Component	GO:0045120	0.00491
mitotic spindle	Cellular Component	GO:0072686	0.00531
extrinsic component of cytoplasmic side of plasma membrane	Cellular Component	GO:0031234	0.00531
perinuclear endoplasmic reticulum	Cellular Component	GO:0097038	0.00578
nuclear heterochromatin	Cellular Component	GO:0005720	0.00592
integral component of synaptic membrane	Cellular Component	GO:0099699	0.00603
protein phosphatase type 2A complex	Cellular Component	GO:0000159	0.0069
neuron projection cytoplasm	Cellular Component	GO:0120111	0.0073
smooth endoplasmic reticulum	Cellular Component	GO:0005790	0.00797
transport vesicle membrane	Cellular Component	GO:0030658	0.00805
microvillus membrane	Cellular Component	GO:0031528	0.00841

clathrin-coated endocytic vesicle	Cellular Component	GO:0045334	0.00848
mitochondria-associated endoplasmic reticulum membrane	Cellular Component	GO:0044233	0.00882
brush border	Cellular Component	GO:0005903	0.00909
nuclear ubiquitin ligase complex	Cellular Component	GO:0000152	0.0104
intrinsic component of presynaptic membrane	Cellular Component	GO:0098889	0.0109
collagen trimer	Cellular Component	GO:0005581	0.0117
protein-DNA complex	Cellular Component	GO:0032993	0.0117
region of cytosol	Cellular Component	GO:0099522	0.0118
perikaryon	Cellular Component	GO:0043204	0.0122
axonal growth cone	Cellular Component	GO:0044295	0.0128
intrinsic component of synaptic membrane	Cellular Component	GO:0099240	0.0136
neuron projection membrane	Cellular Component	GO:0032589	0.0139
axolemma	Cellular Component	GO:0030673	0.0143
COPII-coated ER to Golgi transport vesicle	Cellular Component	GO:0030134	0.0143
Schmidt-Lanterman incisure	Cellular Component	GO:0043220	0.0159
beta-catenin-TCF complex	Cellular Component	GO:1990907	0.0159
azurophil granule membrane	Cellular Component	GO:0035577	0.0167
methyltransferase complex	Cellular Component	GO:0034708	0.017
phagocytic vesicle membrane	Cellular Component	GO:0030670	0.0172
I band	Cellular Component	GO:0031674	0.0194
lamellipodium membrane	Cellular Component	GO:0031258	0.0204
Ino80 complex	Cellular Component	GO:0031011	0.0218
DNA helicase complex	Cellular Component	GO:0033202	0.0218
tertiary granule membrane	Cellular Component	GO:0070821	0.0218
site of DNA damage	Cellular Component	GO:0090734	0.0218
sex chromosome	Cellular Component	GO:0000803	0.0219
ubiquitin ligase complex	Cellular Component	GO:0000151	0.0237
cluster of actin-based cell projections	Cellular Component	GO:0098862	0.0238
SNARE complex	Cellular Component	GO:0031201	0.0239
SWI/SNF complex	Cellular Component	GO:0016514	0.0253
nuclear cyclin-dependent protein kinase holoenzyme complex	Cellular Component	GO:0019908	0.0253
STAGA complex	Cellular Component	GO:0030914	0.0253
astrocyte projection	Cellular Component	GO:0097449	0.0253
replication fork	Cellular Component	GO:0005657	0.0272
P-body	Cellular Component	GO:0000932	0.0277
histone acetyltransferase complex	Cellular Component	GO:0000123	0.0321

adherens junction	Cellular Component	GO:0005912	0.0335
neuromuscular junction	Cellular Component	GO:0031594	0.0335
protein acetyltransferase complex	Cellular Component	GO:0031248	0.034
acetyltransferase complex	Cellular Component	GO:1902493	0.034
site of double-strand break	Cellular Component	GO:0035861	0.0349
dendrite cytoplasm	Cellular Component	GO:0032839	0.035
microtubule	Cellular Component	GO:0005874	0.0358
azurophil granule lumen	Cellular Component	GO:0035578	0.0371
telomere cap complex	Cellular Component	GO:0000782	0.0376
nuclear telomere cap complex	Cellular Component	GO:0000783	0.0376
uropod	Cellular Component	GO:0001931	0.0376
cell trailing edge	Cellular Component	GO:0031254	0.0376
extrinsic component of synaptic membrane	Cellular Component	GO:0099243	0.0376
chromosome, telomeric repeat region	Cellular Component	GO:0140445	0.0376
spindle microtubule	Cellular Component	GO:0005876	0.0399
myosin complex	Cellular Component	GO:0016459	0.0437
integral component of postsynaptic membrane	Cellular Component	GO:0099055	0.0439
Z disc	Cellular Component	GO:0030018	0.0439
carboxy-terminal domain protein kinase complex	Cellular Component	GO:0032806	0.0439
postsynaptic specialization, intracellular component	Cellular Component	GO:0099091	0.0439
organelle envelope lumen	Cellular Component	GO:0031970	0.0439
fascia adherens	Cellular Component	GO:0005916	0.0439
laminin complex	Cellular Component	GO:0043256	0.0439
contractile ring	Cellular Component	GO:0070938	0.0439
tetraspanin-enriched microdomain	Cellular Component	GO:0097197	0.0439
ribbon synapse	Cellular Component	GO:0097470	0.0439
multivesicular body	Cellular Component	GO:0005771	0.0458
DNA-binding transcription activator activity, RNA polymerase II-specific	Molecular Function	GO:0001228	4.71E-50
DNA-binding transcription activator activity	Molecular Function	GO:0001216	4.71E-50
DNA-binding transcription factor binding	Molecular Function	GO:0140297	3.65E-39
protein serine/threonine kinase activity	Molecular Function	GO:0004674	1.18E-32
cell adhesion molecule binding	Molecular Function	GO:0050839	1.62E-27
RNA polymerase II-specific DNA-binding transcription factor binding	Molecular Function	GO:0061629	2.03E-27
ubiquitin-like protein ligase binding	Molecular Function	GO:0044389	8.46E-25
cytokine receptor binding	Molecular Function	GO:0005126	4.80E-24
ubiquitin protein ligase binding	Molecular Function	GO:0031625	5.60E-24

growth factor binding	Molecular Function	GO:0019838	2.84E-22
SMAD binding	Molecular Function	GO:0046332	3.13E-19
protein tyrosine kinase activity	Molecular Function	GO:0004713	1.35E-18
kinase regulator activity	Molecular Function	GO:0019207	9.55E-18
cadherin binding	Molecular Function	GO:0045296	5.48E-17
transcription coactivator activity	Molecular Function	GO:0003713	3.28E-16
transmembrane receptor protein kinase activity	Molecular Function	GO:0019199	4.24E-16
nuclear receptor binding	Molecular Function	GO:0016922	3.08E-15
histone deacetylase binding	Molecular Function	GO:0042826	5.11E-15
phosphatase binding	Molecular Function	GO:0019902	3.05E-14
growth factor receptor binding	Molecular Function	GO:0070851	4.99E-14
nuclear hormone receptor binding	Molecular Function	GO:0035257	1.13E-13
transcription corepressor activity	Molecular Function	GO:0003714	1.13E-13
activating transcription factor binding	Molecular Function	GO:0033613	1.27E-13
hormone receptor binding	Molecular Function	GO:0051427	2.93E-13
integrin binding	Molecular Function	GO:0005178	2.93E-13
protein kinase regulator activity	Molecular Function	GO:0019887	5.41E-13
protein phosphatase binding	Molecular Function	GO:0019903	5.41E-13
growth factor activity	Molecular Function	GO:0008083	1.80E-12
signaling receptor activator activity	Molecular Function	GO:0030546	1.88E-12
protease binding	Molecular Function	GO:0002020	2.96E-12
receptor ligand activity	Molecular Function	GO:0048018	3.26E-12
steroid hormone receptor binding	Molecular Function	GO:0035258	5.44E-12
DNA-binding transcription repressor activity	Molecular Function	GO:0001217	6.35E-12
DNA-binding transcription repressor activity, RNA polymerase II-specific	Molecular Function	GO:0001227	6.35E-12
transmembrane receptor protein tyrosine kinase activity	Molecular Function	GO:0004714	1.24E-11
beta-catenin binding	Molecular Function	GO:0008013	1.36E-11
protein C-terminus binding	Molecular Function	GO:0008022	1.42E-11
cytokine activity	Molecular Function	GO:0005125	1.66E-11
steroid hormone receptor activity	Molecular Function	GO:0003707	5.00E-11
nuclear receptor activity	Molecular Function	GO:0004879	1.53E-10
ligand-activated transcription factor activity	Molecular Function	GO:0098531	1.53E-10
E-box binding	Molecular Function	GO:0070888	2.03E-10
RNA polymerase II transcription factor binding	Molecular Function	GO:0001085	2.15E-10
kinase activator activity	Molecular Function	GO:0019209	4.62E-10
repressing transcription factor binding	Molecular Function	GO:0070491	4.74E-10

tumor necrosis factor receptor superfamily binding	Molecular Function	GO:0032813	4.85E-10
tumor necrosis factor receptor binding	Molecular Function	GO:0005164	4.14E-09
cytokine binding	Molecular Function	GO:0019955	4.88E-09
Wnt-protein binding	Molecular Function	GO:0017147	1.06E-08
NAD-dependent histone deacetylase activity	Molecular Function	GO:0017136	1.17E-08
G protein-coupled receptor binding	Molecular Function	GO:0001664	1.79E-08
protein serine/threonine/tyrosine kinase activity	Molecular Function	GO:0004712	1.79E-08
bHLH transcription factor binding	Molecular Function	GO:0043425	2.11E-08
chaperone binding	Molecular Function	GO:0051087	2.44E-08
phosphoprotein phosphatase activity	Molecular Function	GO:0004721	3.53E-08
protein kinase activator activity	Molecular Function	GO:0030295	3.54E-08
histone acetyltransferase binding	Molecular Function	GO:0035035	5.20E-08
MAP kinase activity	Molecular Function	GO:0004707	5.20E-08
tau protein binding	Molecular Function	GO:0048156	6.25E-08
NAD-dependent protein deacetylase activity	Molecular Function	GO:0034979	6.47E-08
cyclin-dependent protein serine/threonine kinase regulator activity	Molecular Function	GO:0016538	1.25E-07
platelet-derived growth factor binding	Molecular Function	GO:0048407	1.28E-07
transcription coactivator binding	Molecular Function	GO:0001223	1.44E-07
RNA polymerase II activating transcription factor binding	Molecular Function	GO:0001102	2.19E-07
phosphatase activity	Molecular Function	GO:0016791	2.50E-07
retinoic acid receptor binding	Molecular Function	GO:0042974	2.64E-07
retinoid X receptor binding	Molecular Function	GO:0046965	2.73E-07
promoter-specific chromatin binding	Molecular Function	GO:1990841	3.45E-07
transforming growth factor beta receptor binding	Molecular Function	GO:0005160	3.49E-07
protein tyrosine kinase binding	Molecular Function	GO:1990782	3.74E-07
protein serine/threonine kinase activator activity	Molecular Function	GO:0043539	4.11E-07
transcription cofactor binding	Molecular Function	GO:0001221	5.23E-07
phosphoric ester hydrolase activity	Molecular Function	GO:0042578	5.40E-07
protein self-association	Molecular Function	GO:0043621	9.09E-07
glycosaminoglycan binding	Molecular Function	GO:0005539	9.96E-07
disordered domain specific binding	Molecular Function	GO:0097718	1.06E-06
protein tyrosine phosphatase activity	Molecular Function	GO:0004725	1.17E-06
phosphoprotein binding	Molecular Function	GO:0051219	1.27E-06
histone deacetylase activity	Molecular Function	GO:0004407	1.27E-06
R-SMAD binding	Molecular Function	GO:0070412	1.27E-06
frizzled binding	Molecular Function	GO:0005109	1.35E-06

protein phosphorylated amino acid binding	Molecular Function	GO:0045309	1.47E-06
miRNA binding	Molecular Function	GO:0035198	1.61E-06
protein N-terminus binding	Molecular Function	GO:0047485	1.95E-06
molecular adaptor activity	Molecular Function	GO:0060090	2.13E-06
ion channel binding	Molecular Function	GO:0044325	2.13E-06
amyloid-beta binding	Molecular Function	GO:0001540	2.26E-06
phosphotyrosine residue binding	Molecular Function	GO:0001784	2.28E-06
regulatory RNA binding	Molecular Function	GO:0061980	2.35E-06
protein deacetylase activity	Molecular Function	GO:0033558	2.45E-06
protein-macromolecule adaptor activity	Molecular Function	GO:0030674	2.92E-06
GTPase activity	Molecular Function	GO:0003924	3.13E-06
protein serine/threonine phosphatase activity	Molecular Function	GO:0004722	3.58E-06
chemoattractant activity	Molecular Function	GO:0042056	3.66E-06
transforming growth factor beta-activated receptor activity	Molecular Function	GO:0005024	3.97E-06
type I transforming growth factor beta receptor binding	Molecular Function	GO:0034713	4.10E-06
insulin receptor substrate binding	Molecular Function	GO:0043560	4.10E-06
I-SMAD binding	Molecular Function	GO:0070411	4.10E-06
mitogen-activated protein kinase kinase binding	Molecular Function	GO:0031434	4.34E-06
cyclin binding	Molecular Function	GO:0030332	4.58E-06
extracellular matrix structural constituent	Molecular Function	GO:0005201	5.88E-06
insulin-like growth factor binding	Molecular Function	GO:0005520	7.48E-06
scaffold protein binding	Molecular Function	GO:0097110	1.08E-05
MAP kinase kinase activity	Molecular Function	GO:0004708	1.18E-05
insulin-like growth factor receptor binding	Molecular Function	GO:0005159	1.18E-05
glucocorticoid receptor binding	Molecular Function	GO:0035259	1.18E-05
MAP kinase kinase kinase activity	Molecular Function	GO:0004709	1.18E-05
p53 binding	Molecular Function	GO:0002039	1.18E-05
protein kinase inhibitor activity	Molecular Function	GO:0004860	1.35E-05
1-phosphatidylinositol-3-kinase regulator activity	Molecular Function	GO:0046935	1.52E-05
coreceptor activity	Molecular Function	GO:0015026	1.59E-05
collagen binding	Molecular Function	GO:0005518	1.65E-05
kinase inhibitor activity	Molecular Function	GO:0019210	1.65E-05
virus receptor activity	Molecular Function	GO:0001618	1.69E-05
exogenous protein binding	Molecular Function	GO:0140272	1.69E-05
insulin-like growth factor I binding	Molecular Function	GO:0031994	1.71E-05
histone deacetylase activity (H3-K14 specific)	Molecular Function	GO:0031078	1.71E-05

NAD-dependent histone deacetylase activity (H3-K14 specific)	Molecular Function	GO:0032041	1.71E-05
heparin binding	Molecular Function	GO:0008201	1.85E-05
actin binding	Molecular Function	GO:0003779	1.88E-05
estrogen receptor binding	Molecular Function	GO:0030331	2.51E-05
transmembrane receptor protein serine/threonine kinase activity	Molecular Function	GO:0004675	2.87E-05
phosphatidylinositol 3-kinase regulator activity	Molecular Function	GO:0035014	2.87E-05
Wnt-activated receptor activity	Molecular Function	GO:0042813	2.87E-05
calmodulin binding	Molecular Function	GO:0005516	2.88E-05
platelet-derived growth factor receptor binding	Molecular Function	GO:0005161	4.16E-05
RNA polymerase II core promoter sequence-specific DNA binding	Molecular Function	GO:0000979	4.36E-05
purine nucleoside binding	Molecular Function	GO:0001883	4.92E-05
GDP binding	Molecular Function	GO:0019003	4.92E-05
heat shock protein binding	Molecular Function	GO:0031072	5.38E-05
extracellular matrix binding	Molecular Function	GO:0050840	5.50E-05
co-receptor binding	Molecular Function	GO:0039706	5.53E-05
myosin binding	Molecular Function	GO:0017022	5.67E-05
purine ribonucleoside binding	Molecular Function	GO:0032550	5.67E-05
androgen receptor binding	Molecular Function	GO:0050681	5.79E-05
GTP binding	Molecular Function	GO:0005525	5.94E-05
magnesium ion binding	Molecular Function	GO:0000287	6.03E-05
extracellular matrix structural constituent conferring tensile strength	Molecular Function	GO:0030020	6.16E-05
mitogen-activated protein kinase binding	Molecular Function	GO:0051019	6.16E-05
nucleoside binding	Molecular Function	GO:0001882	6.16E-05
protein serine/threonine kinase inhibitor activity	Molecular Function	GO:0030291	6.75E-05
protein phosphatase 2A binding	Molecular Function	GO:0051721	6.75E-05
ribonucleoside binding	Molecular Function	GO:0032549	7.69E-05
receptor tyrosine kinase binding	Molecular Function	GO:0030971	8.77E-05
protein kinase C activity	Molecular Function	GO:0004697	9.59E-05
HMG box domain binding	Molecular Function	GO:0071837	9.59E-05
SH3 domain binding	Molecular Function	GO:0017124	9.90E-05
proteoglycan binding	Molecular Function	GO:0043394	0.000106
ephrin receptor binding	Molecular Function	GO:0046875	0.000108
phosphatidylinositol 3-kinase binding	Molecular Function	GO:0043548	0.000111
oxidoreductase activity, acting on paired donors, with incorporation or reduction of molecular oxygen	Molecular Function	GO:0016705	0.000114
protein kinase C binding	Molecular Function	GO:0005080	0.000137

activin binding	Molecular Function	GO:0048185	0.00014
cysteine-type endopeptidase inhibitor activity involved in apoptotic process	Molecular Function	GO:0043027	0.000179
peptide binding	Molecular Function	GO:0042277	0.000189
guanyl nucleotide binding	Molecular Function	GO:0019001	0.000189
guanyl ribonucleotide binding	Molecular Function	GO:0032561	0.000189
cyclin-dependent protein serine/threonine kinase inhibitor activity	Molecular Function	GO:0004861	0.000196
co-SMAD binding	Molecular Function	GO:0070410	0.000196
deacetylase activity	Molecular Function	GO:0019213	0.000199
histone kinase activity	Molecular Function	GO:0035173	0.000206
damaged DNA binding	Molecular Function	GO:0003684	0.000215
Rac GTPase binding	Molecular Function	GO:0048365	0.00024
1-phosphatidylinositol-3-kinase activity	Molecular Function	GO:0016303	0.000258
RNA polymerase II repressing transcription factor binding	Molecular Function	GO:0001103	0.000268
chemokine binding	Molecular Function	GO:0019956	0.000283
fibroblast growth factor binding	Molecular Function	GO:0017134	0.000283
core promoter sequence-specific DNA binding	Molecular Function	GO:0001046	0.000283
laminin binding	Molecular Function	GO:0043236	0.000291
NF-kappaB binding	Molecular Function	GO:0051059	0.000291
lipoprotein particle receptor binding	Molecular Function	GO:0070325	0.000292
translation repressor activity, mRNA regulatory element binding	Molecular Function	GO:0000900	0.000312
nuclear receptor transcription coactivator activity	Molecular Function	GO:0030374	0.000372
PDZ domain binding	Molecular Function	GO:0030165	4.00E-04
non-membrane spanning protein tyrosine kinase activity	Molecular Function	GO:0004715	4.00E-04
epidermal growth factor receptor binding	Molecular Function	GO:0005154	0.000435
low-density lipoprotein particle receptor binding	Molecular Function	GO:0050750	0.000463
cyclin-dependent protein kinase activity	Molecular Function	GO:0097472	0.000464
fibronectin binding	Molecular Function	GO:0001968	0.000482
sulfur compound binding	Molecular Function	GO:1901681	0.000484
calcium-dependent protein binding	Molecular Function	GO:0048306	0.000525
Rho GTPase binding	Molecular Function	GO:0017048	0.000531
chromatin DNA binding	Molecular Function	GO:0031490	0.000539
enzyme inhibitor activity	Molecular Function	GO:0004857	0.000679
thioesterase binding	Molecular Function	GO:0031996	0.000701
phosphatidylinositol 3-kinase activity	Molecular Function	GO:0035004	0.000701
Hsp90 protein binding	Molecular Function	GO:0051879	0.000744
insulin receptor binding	Molecular Function	GO:0005158	0.000795

transforming growth factor beta binding	Molecular Function	GO:0050431	0.000795
single-stranded DNA binding	Molecular Function	GO:0003697	0.000795
cAMP response element binding	Molecular Function	GO:0035497	0.00105
cysteine-type endopeptidase regulator activity involved in apoptotic process	Molecular Function	GO:0043028	0.00105
iron ion binding	Molecular Function	GO:0005506	0.00109
cyclin-dependent protein serine/threonine kinase activity	Molecular Function	GO:0004693	0.00119
steroid binding	Molecular Function	GO:0005496	0.0012
death receptor binding	Molecular Function	GO:0005123	0.00124
apolipoprotein binding	Molecular Function	GO:0034185	0.00124
translation repressor activity	Molecular Function	GO:0030371	0.00127
DNA binding, bending	Molecular Function	GO:0008301	0.00132
carboxylic acid binding	Molecular Function	GO:0031406	0.0015
amide binding	Molecular Function	GO:0033218	0.00154
Toll-like receptor binding	Molecular Function	GO:0035325	0.00162
peroxisome proliferator activated receptor binding	Molecular Function	GO:0042975	0.00162
chemokine receptor binding	Molecular Function	GO:0042379	0.00192
telomeric DNA binding	Molecular Function	GO:0042162	0.00198
S100 protein binding	Molecular Function	GO:0044548	0.00202
cysteine-type endopeptidase activity involved in apoptotic process	Molecular Function	GO:0097153	0.00202
RNA polymerase II CTD heptapeptide repeat kinase activity	Molecular Function	GO:0008353	0.0022
mitogen-activated protein kinase kinase kinase binding	Molecular Function	GO:0031435	0.0022
histone binding	Molecular Function	GO:0042393	0.00249
BH domain binding	Molecular Function	GO:0051400	0.0025
double-stranded RNA binding	Molecular Function	GO:0003725	0.00254
mRNA 3'-UTR binding	Molecular Function	GO:0003730	0.00259
channel inhibitor activity	Molecular Function	GO:0016248	0.00273
actin filament binding	Molecular Function	GO:0051015	0.00279
AU-rich element binding	Molecular Function	GO:0017091	0.00291
chemokine activity	Molecular Function	GO:0008009	0.00291
ubiquitin-like protein binding	Molecular Function	GO:0032182	0.00307
fibroblast growth factor receptor binding	Molecular Function	GO:0005104	0.00323
protein kinase A catalytic subunit binding	Molecular Function	GO:0034236	0.00326
four-way junction DNA binding	Molecular Function	GO:0000400	0.0036
phosphatase activator activity	Molecular Function	GO:0019211	0.0036
GTPase activating protein binding	Molecular Function	GO:0032794	0.0036
cadherin binding involved in cell-cell adhesion	Molecular Function	GO:0098641	0.00362

organic acid binding	Molecular Function	GO:0043177	0.00371
14-3-3 protein binding	Molecular Function	GO:0071889	0.00422
SH2 domain binding	Molecular Function	GO:0042169	0.00437
glutamate receptor binding	Molecular Function	GO:0035254	0.00457
mRNA 3'-UTR AU-rich region binding	Molecular Function	GO:0035925	0.00479
metalloendopeptidase activity	Molecular Function	GO:0004222	0.00503
CXCR chemokine receptor binding	Molecular Function	GO:0045236	0.00526
RAGE receptor binding	Molecular Function	GO:0050786	0.00526
structural molecule activity conferring elasticity	Molecular Function	GO:0097493	0.00526
ion channel inhibitor activity	Molecular Function	GO:0008200	0.00594
nitric-oxide synthase binding	Molecular Function	GO:0050998	0.006
ubiquitin binding	Molecular Function	GO:0043130	0.0069
histone demethylase activity	Molecular Function	GO:0032452	0.00694
protein demethylase activity	Molecular Function	GO:0140457	0.00694
heme binding	Molecular Function	GO:0020037	0.00696
2-oxoglutarate-dependent dioxygenase activity	Molecular Function	GO:0016706	0.00713
steroid hydroxylase activity	Molecular Function	GO:0008395	0.00795
WW domain binding	Molecular Function	GO:0050699	0.00836
protein kinase A binding	Molecular Function	GO:0051018	0.00978
ubiquitin-like protein transferase activity	Molecular Function	GO:0019787	0.00987
actin monomer binding	Molecular Function	GO:0003785	0.00987
signaling adaptor activity	Molecular Function	GO:0035591	0.00999
low-density lipoprotein particle receptor activity	Molecular Function	GO:0005041	0.01
myosin heavy chain binding	Molecular Function	GO:0032036	0.01
lipoprotein particle receptor activity	Molecular Function	GO:0030228	0.0102
phosphatidylinositol phosphate binding	Molecular Function	GO:1901981	0.0115
monosaccharide binding	Molecular Function	GO:0048029	0.0121
cytokine receptor activity	Molecular Function	GO:0004896	0.0124
signaling receptor complex adaptor activity	Molecular Function	GO:0030159	0.0126
microfilament motor activity	Molecular Function	GO:0000146	0.0131
GTP-dependent protein binding	Molecular Function	GO:0030742	0.0131
phospholipid binding	Molecular Function	GO:0005543	0.0144
SNARE binding	Molecular Function	GO:0000149	0.0146
adrenergic receptor binding	Molecular Function	GO:0031690	0.0149
lipoprotein particle binding	Molecular Function	GO:0071813	0.0151
protein-lipid complex binding	Molecular Function	GO:0071814	0.0151

NAD binding	Molecular Function	GO:0051287	0.0151
structural constituent of cytoskeleton	Molecular Function	GO:0005200	0.0158
methyl-CpG binding	Molecular Function	GO:0008327	0.0158
phospholipase binding	Molecular Function	GO:0043274	0.0161
phosphatidylinositol kinase activity	Molecular Function	GO:0052742	0.0161
demethylase activity	Molecular Function	GO:0032451	0.0161
neurotrophin receptor binding	Molecular Function	GO:0005165	0.0161
chloride channel inhibitor activity	Molecular Function	GO:0019869	0.0161
Tat protein binding	Molecular Function	GO:0030957	0.0161
histone methyltransferase activity (H3-K9 specific)	Molecular Function	GO:0046974	0.0161
G-rich strand telomeric DNA binding	Molecular Function	GO:0098505	0.0161
vascular endothelial growth factor receptor binding	Molecular Function	GO:0005172	0.0166
protein phosphatase activator activity	Molecular Function	GO:0072542	0.0166
hydrolase activity, acting on carbon-nitrogen (but not peptide) bonds, in linear amides	Molecular Function	GO:0016811	0.0168
phosphatidylinositol binding	Molecular Function	GO:0035091	0.02
ADP binding	Molecular Function	GO:0043531	0.0206
manganese ion binding	Molecular Function	GO:0030145	0.0217
tetrapyrrole binding	Molecular Function	GO:0046906	0.0236
phosphatidylinositol-4,5-bisphosphate binding	Molecular Function	GO:0005546	0.0238
chloride channel regulator activity	Molecular Function	GO:0017081	0.0242
myosin V binding	Molecular Function	GO:0031489	0.0242
DNA secondary structure binding	Molecular Function	GO:0000217	0.0254
mismatched DNA binding	Molecular Function	GO:0030983	0.0269
NAD+ binding	Molecular Function	GO:0070403	0.0269
sequence-specific single stranded DNA binding	Molecular Function	GO:0098847	0.0269
glucose binding	Molecular Function	GO:0005536	0.028
mismatch repair complex binding	Molecular Function	GO:0032404	0.028
siRNA binding	Molecular Function	GO:0035197	0.028
protein kinase B binding	Molecular Function	GO:0043422	0.028
BMP receptor binding	Molecular Function	GO:0070700	0.028
cargo adaptor activity	Molecular Function	GO:0140312	0.028
tubulin binding	Molecular Function	GO:0015631	0.0288
Ras GTPase binding	Molecular Function	GO:0017016	0.029
microtubule binding	Molecular Function	GO:0008017	0.0317
NADP binding	Molecular Function	GO:0050661	0.0323
histone-lysine N-methyltransferase activity	Molecular Function	GO:0018024	0.0326

nuclear localization sequence binding	Molecular Function	GO:0008139	0.0335
receptor serine/threonine kinase binding	Molecular Function	GO:0033612	0.0335
mRNA 5'-UTR binding	Molecular Function	GO:0048027	0.0335
nucleoside-triphosphatase regulator activity	Molecular Function	GO:0060589	0.0354
peptidase regulator activity	Molecular Function	GO:0061134	0.0354
dioxygenase activity	Molecular Function	GO:0051213	0.0367
small GTPase binding	Molecular Function	GO:0031267	0.0368
clathrin binding	Molecular Function	GO:0030276	0.0376
C2H2 zinc finger domain binding	Molecular Function	GO:0070742	0.0398
tau-protein kinase activity	Molecular Function	GO:0050321	0.0402
Notch binding	Molecular Function	GO:0005112	0.0437
syntaxin-1 binding	Molecular Function	GO:0017075	0.0437
syntaxin binding	Molecular Function	GO:0019905	0.0437
ubiquitin-protein transferase activity	Molecular Function	GO:0004842	0.0437
ribonucleoprotein complex binding	Molecular Function	GO:0043021	0.0437
semaphorin receptor activity	Molecular Function	GO:0017154	0.0437
phosphate ion binding	Molecular Function	GO:0042301	0.0437
single-stranded telomeric DNA binding	Molecular Function	GO:0043047	0.0437
gamma-catenin binding	Molecular Function	GO:0045295	0.0437
sequence-specific mRNA binding	Molecular Function	GO:1990825	0.0437
ephrin receptor activity	Molecular Function	GO:0005003	0.0473
transmembrane receptor protein serine/threonine kinase binding	Molecular Function	GO:0070696	0.0473
DNA polymerase activity	Molecular Function	GO:0034061	0.0478
mRNA target	TTD Target	mRNA target	7.64E-18
Kinase	TTD Target	Kinase	8.93E-12
Nuclear hormone receptor	TTD Target	Nuclear hormone receptor	0.000146
Basic leucine zipper bZIP	TTD Target	Basic leucine zipper bZIP	0.00121
