# Supplementary material for: Decoding Non-Coding RNA Regulators in DITRA: From Genomic Insights to Potential Biomarkers and Therapeutic Targets
Source: Genes (Basel). 2025 Jun 27;16(7):753. doi: 10.3390/genes16070753 (PMC12295128; doi:10.3390/genes16070753)
Supplement: Supplementary file 1 [file genes-16-00753-s001.zip › Supplementary-TableS7.pdf]

Description	Database	ID	p.adjust
Interleukin-4 and Interleukin-13 signaling	Reactome Pathway	R-HSA-6785807	1.55E-36
Signaling by Interleukins	Reactome Pathway	R-HSA-449147	4.20E-26
Diseases of signal transduction by growth factor receptors and second messengers	Reactome Pathway	R-HSA-5663202	7.53E-20
Intracellular signaling by second messengers	Reactome Pathway	R-HSA-9006925	4.86E-18
PIP3 activates AKT signaling	Reactome Pathway	R-HSA-1257604	4.86E-18
Signaling by VEGF	Reactome Pathway	R-HSA-194138	2.12E-15
VEGFA-VEGFR2 Pathway	Reactome Pathway	R-HSA-4420097	6.06E-15
Cellular Senescence	Reactome Pathway	R-HSA-2559583	2.14E-14
PI3K/AKT Signaling in Cancer	Reactome Pathway	R-HSA-2219528	2.53E-14
Cyclin D associated events in G1	Reactome Pathway	R-HSA-69231	2.53E-12
G1 Phase	Reactome Pathway	R-HSA-69236	2.53E-12
MAPK family signaling cascades	Reactome Pathway	R-HSA-5683057	2.70E-12
Aberrant regulation of mitotic G1/S transition in cancer due to RB1 defects	Reactome Pathway	R-HSA-9659787	1.23E-11
Defective binding of RB1 mutants to E2F1,(E2F2, E2F3)	Reactome Pathway	R-HSA-9661069	1.23E-11
Signaling by NTRKs	Reactome Pathway	R-HSA-166520	5.08E-11
Mitotic G1 phase and G1/S transition	Reactome Pathway	R-HSA-453279	1.80E-10
Extra-nuclear estrogen signaling	Reactome Pathway	R-HSA-9009391	2.83E-10
FOXO-mediated transcription	Reactome Pathway	R-HSA-9614085	3.30E-10
Negative regulation of the PI3K/AKT network	Reactome Pathway	R-HSA-199418	3.41E-10
Signaling by SCF-KIT	Reactome Pathway	R-HSA-1433557	3.73E-10
Signaling by PTK6	Reactome Pathway	R-HSA-8848021	3.73E-10
Signaling by Non-Receptor Tyrosine Kinases	Reactome Pathway	R-HSA-9006927	3.73E-10
ESR-mediated signaling	Reactome Pathway	R-HSA-8939211	3.73E-10
Signaling by MET	Reactome Pathway	R-HSA-6806834	3.73E-10
Signaling by ERBB2	Reactome Pathway	R-HSA-1227986	6.52E-10
Signaling by TGFB family members	Reactome Pathway	R-HSA-9006936	1.02E-09
Signaling by Nuclear Receptors	Reactome Pathway	R-HSA-9006931	1.11E-09
Interleukin-10 signaling	Reactome Pathway	R-HSA-6783783	1.74E-09
Signaling by NTRK1 (TRKA)	Reactome Pathway	R-HSA-187037	2.04E-09
FLT3 Signaling	Reactome Pathway	R-HSA-9607240	3.63E-09
CD28 co-stimulation	Reactome Pathway	R-HSA-389356	3.63E-09
Signaling by KIT in disease	Reactome Pathway	R-HSA-9669938	3.92E-09
Signaling by phosphorylated juxtamembrane, extracellular and kinase domain KIT mutants	Reactome Pathway	R-HSA-9670439	3.92E-09
Signaling by TGF-beta Receptor Complex	Reactome Pathway	R-HSA-170834	4.28E-09

Aberrant regulation of mitotic cell cycle due to RB1 defects	Reactome Pathway	R-HSA-9687139	1.46E-08
Programmed Cell Death	Reactome Pathway	R-HSA-5357801	2.21E-08
Regulation of TP53 Expression and Degradation	Reactome Pathway	R-HSA-6806003	2.21E-08
G0 and Early G1	Reactome Pathway	R-HSA-1538133	2.22E-08
Regulated Necrosis	Reactome Pathway	R-HSA-5218859	3.05E-08
Diseases of mitotic cell cycle	Reactome Pathway	R-HSA-9675126	3.19E-08
Transcriptional Regulation by TP53	Reactome Pathway	R-HSA-3700989	3.41E-08
Oncogene Induced Senescence	Reactome Pathway	R-HSA-2559585	3.54E-08
Signaling by PDGFRA transmembrane, juxtamembrane and kinase domain mutants	Reactome Pathway	R-HSA-9673767	3.63E-08
Signaling by PDGFRA extracellular domain mutants	Reactome Pathway	R-HSA-9673770	3.63E-08
Transcriptional Regulation by VENTX	Reactome Pathway	R-HSA-8853884	4.37E-08
Constitutive Signaling by Aberrant PI3K in Cancer	Reactome Pathway	R-HSA-2219530	4.76E-08
Transcriptional regulation of pluripotent stem cells	Reactome Pathway	R-HSA-452723	4.76E-08
Downstream signal transduction	Reactome Pathway	R-HSA-186763	5.34E-08
Signaling by FLT3 ITD and TKD mutants	Reactome Pathway	R-HSA-9703648	5.77E-08
Signaling by Erythropoietin	Reactome Pathway	R-HSA-9006335	8.16E-08
Transcriptional regulation by RUNX3	Reactome Pathway	R-HSA-8878159	8.83E-08
Regulation of TP53 Degradation	Reactome Pathway	R-HSA-6804757	1.13E-07
Regulation of RUNX1 Expression and Activity	Reactome Pathway	R-HSA-8934593	1.21E-07
Signaling by NTRK3 (TRKC)	Reactome Pathway	R-HSA-9034015	1.21E-07
PTEN Regulation	Reactome Pathway	R-HSA-6807070	1.33E-07
Intrinsic Pathway for Apoptosis	Reactome Pathway	R-HSA-109606	1.40E-07
MAPK1/MAPK3 signaling	Reactome Pathway	R-HSA-5684996	1.40E-07
Oxidative Stress Induced Senescence	Reactome Pathway	R-HSA-2559580	1.87E-07
Signaling by ALK	Reactome Pathway	R-HSA-201556	2.08E-07
TP53 Regulates Transcription of Genes Involved in G1 Cell Cycle Arrest	Reactome Pathway	R-HSA-6804116	2.18E-07
SUMO E3 ligases SUMOylate target proteins	Reactome Pathway	R-HSA-3108232	2.75E-07
Platelet activation, signaling and aggregation	Reactome Pathway	R-HSA-76002	3.17E-07
IRS-related events triggered by IGF1R	Reactome Pathway	R-HSA-2428928	3.78E-07
Constitutive Signaling by EGFRvIII	Reactome Pathway	R-HSA-5637810	4.78E-07
Signaling by EGFRvIII in Cancer	Reactome Pathway	R-HSA-5637812	4.78E-07
IGF1R signaling cascade	Reactome Pathway	R-HSA-2428924	4.89E-07
PI5P, PP2A and IER3 Regulate PI3K/AKT Signaling	Reactome Pathway	R-HSA-6811558	5.02E-07
Nucleotide-binding domain, leucine rich repeat containing receptor (NLR) signaling pathways	Reactome Pathway	R-HSA-168643	5.02E-07
SUMOylation	Reactome Pathway	R-HSA-2990846	5.44E-07

RAF/MAP kinase cascade	Reactome Pathway	R-HSA-5673001	5.59E-07
Regulation of TP53 Activity	Reactome Pathway	R-HSA-5633007	5.66E-07
Signaling by Type 1 Insulin-like Growth Factor 1 Receptor (IGF1R)	Reactome Pathway	R-HSA-2404192	6.03E-07
Regulation of PTEN gene transcription	Reactome Pathway	R-HSA-8943724	6.14E-07
EPH-Ephrin signaling	Reactome Pathway	R-HSA-2682334	7.27E-07
Sema4D in semaphorin signaling	Reactome Pathway	R-HSA-400685	7.49E-07
Constitutive Signaling by AKT1 E17K in Cancer	Reactome Pathway	R-HSA-5674400	7.49E-07
Signaling by NTRK2 (TRKB)	Reactome Pathway	R-HSA-9006115	7.49E-07
Transcriptional Regulation by MECP2	Reactome Pathway	R-HSA-8986944	7.49E-07
Apoptosis	Reactome Pathway	R-HSA-109581	7.69E-07
Signaling by ERBB2 ECD mutants	Reactome Pathway	R-HSA-9665348	8.42E-07
Cell surface interactions at the vascular wall	Reactome Pathway	R-HSA-202733	1.14E-06
Signal transduction by L1	Reactome Pathway	R-HSA-445144	1.17E-06
Signaling by EGFR	Reactome Pathway	R-HSA-177929	1.17E-06
TGF-beta receptor signaling activates SMADs	Reactome Pathway	R-HSA-2173789	1.45E-06
Semaphorin interactions	Reactome Pathway	R-HSA-373755	1.48E-06
Signaling by PDGF	Reactome Pathway	R-HSA-186797	1.54E-06
FOXO-mediated transcription of cell cycle genes	Reactome Pathway	R-HSA-9617828	1.56E-06
CD28 dependent PI3K/Akt signaling	Reactome Pathway	R-HSA-389357	1.89E-06
FLT3 signaling in disease	Reactome Pathway	R-HSA-9682385	2.70E-06
MET promotes cell motility	Reactome Pathway	R-HSA-8875878	3.88E-06
VEGFR2 mediated vascular permeability	Reactome Pathway	R-HSA-5218920	3.98E-06
Caspase activation via extrinsic apoptotic signalling pathway	Reactome Pathway	R-HSA-5357769	3.98E-06
Estrogen-dependent nuclear events downstream of ESR-membrane signaling	Reactome Pathway	R-HSA-9634638	4.84E-06
Constitutive Signaling by Ligand-Responsive EGFR Cancer Variants	Reactome Pathway	R-HSA-1236382	4.84E-06
Signaling by Ligand-Responsive EGFR Variants in Cancer	Reactome Pathway	R-HSA-5637815	4.84E-06
Signaling by FLT3 fusion proteins	Reactome Pathway	R-HSA-9703465	4.84E-06
EPHB-mediated forward signaling	Reactome Pathway	R-HSA-3928662	4.93E-06
DAP12 signaling	Reactome Pathway	R-HSA-2424491	5.60E-06
Potential therapeutics for SARS	Reactome Pathway	R-HSA-9679191	6.03E-06
Interleukin-6 signaling	Reactome Pathway	R-HSA-1059683	6.84E-06
Signaling by FGFR4 in disease	Reactome Pathway	R-HSA-5655291	6.84E-06
MET activates RAS signaling	Reactome Pathway	R-HSA-8851805	6.84E-06
Signaling by ERBB2 KD Mutants	Reactome Pathway	R-HSA-9664565	6.96E-06
Signaling by PDGFR in disease	Reactome Pathway	R-HSA-9671555	7.70E-06
Signaling by ERBB4	Reactome Pathway	R-HSA-1236394	7.91E-06

TP53 Regulates Transcription of Cell Death Genes	Reactome Pathway	R-HSA-5633008	8.25E-06
Signaling by NOTCH1	Reactome Pathway	R-HSA-1980143	8.82E-06
Signaling by ERBB2 in Cancer	Reactome Pathway	R-HSA-1227990	1.01E-05
Downregulation of TGF-beta receptor signaling	Reactome Pathway	R-HSA-2173788	1.01E-05
Cyclin E associated events during G1/S transition	Reactome Pathway	R-HSA-69202	1.01E-05
Nuclear Receptor transcription pathway	Reactome Pathway	R-HSA-383280	1.04E-05
Regulation of KIT signaling	Reactome Pathway	R-HSA-1433559	1.13E-05
Transcriptional regulation of white adipocyte differentiation	Reactome Pathway	R-HSA-381340	1.19E-05
Cyclin A:Cdk2-associated events at S phase entry	Reactome Pathway	R-HSA-69656	1.41E-05
G1/S Transition	Reactome Pathway	R-HSA-69206	1.44E-05
Transcriptional regulation by RUNX2	Reactome Pathway	R-HSA-8878166	1.49E-05
Costimulation by the CD28 family	Reactome Pathway	R-HSA-388841	1.49E-05
Insulin receptor signalling cascade	Reactome Pathway	R-HSA-74751	1.61E-05
Pyroptosis	Reactome Pathway	R-HSA-5620971	2.06E-05
Signaling by NOTCH	Reactome Pathway	R-HSA-157118	2.11E-05
IRS-mediated signalling	Reactome Pathway	R-HSA-112399	2.15E-05
Signaling by FGFR4	Reactome Pathway	R-HSA-5654743	2.15E-05
Fc epsilon receptor (FCERI) signaling	Reactome Pathway	R-HSA-2454202	2.32E-05
GPVI-mediated activation cascade	Reactome Pathway	R-HSA-114604	2.59E-05
RAF-independent MAPK1/3 activation	Reactome Pathway	R-HSA-112409	2.66E-05
MyD88-independent TLR4 cascade	Reactome Pathway	R-HSA-166166	3.11E-05
TRIF(TICAM1)-mediated TLR4 signaling	Reactome Pathway	R-HSA-937061	3.11E-05
DDX58/IFIH1-mediated induction of interferon-alpha/beta	Reactome Pathway	R-HSA-168928	3.37E-05
Signaling by high-kinase activity BRAF mutants	Reactome Pathway	R-HSA-6802948	3.40E-05
TRAF6 mediated induction of NFkB and MAP kinases upon TLR7/8 or 9 activation	Reactome Pathway	R-HSA-975138	3.50E-05
Signaling by RAF1 mutants	Reactome Pathway	R-HSA-9656223	3.50E-05
L1CAM interactions	Reactome Pathway	R-HSA-373760	3.72E-05
Interleukin-6 family signaling	Reactome Pathway	R-HSA-6783589	3.72E-05
EPHA-mediated growth cone collapse	Reactome Pathway	R-HSA-3928663	3.72E-05
SUMOylation of intracellular receptors	Reactome Pathway	R-HSA-4090294	3.72E-05
MET activates PTK2 signaling	Reactome Pathway	R-HSA-8874081	3.72E-05
MyD88 dependent cascade initiated on endosome	Reactome Pathway	R-HSA-975155	3.91E-05
Extracellular matrix organization	Reactome Pathway	R-HSA-1474244	3.93E-05
EPH-ephrin mediated repulsion of cells	Reactome Pathway	R-HSA-3928665	3.96E-05
Chemokine receptors bind chemokines	Reactome Pathway	R-HSA-380108	4.24E-05
Oncogenic MAPK signaling	Reactome Pathway	R-HSA-6802957	4.33E-05

Erythropoietin activates RAS	Reactome Pathway	R-HSA-9027284	4.34E-05
MyD88:MAL(TIRAP) cascade initiated on plasma membrane	Reactome Pathway	R-HSA-166058	4.34E-05
Toll Like Receptor TLR6:TLR2 Cascade	Reactome Pathway	R-HSA-168188	4.34E-05
Toll Like Receptor 7/8 (TLR7/8) Cascade	Reactome Pathway	R-HSA-168181	4.34E-05
Toll Like Receptor 3 (TLR3) Cascade	Reactome Pathway	R-HSA-168164	5.05E-05
Signaling by EGFR in Cancer	Reactome Pathway	R-HSA-1643713	5.10E-05
Signaling by FGFR3 fusions in cancer	Reactome Pathway	R-HSA-8853334	5.25E-05
Molecules associated with elastic fibres	Reactome Pathway	R-HSA-2129379	5.30E-05
Fcgamma receptor (FCGR) dependent phagocytosis	Reactome Pathway	R-HSA-2029480	5.69E-05
Death Receptor Signalling	Reactome Pathway	R-HSA-73887	5.88E-05
SARS-CoV Infections	Reactome Pathway	R-HSA-9679506	6.03E-05
Toll Like Receptor TLR1:TLR2 Cascade	Reactome Pathway	R-HSA-168179	6.30E-05
Toll Like Receptor 2 (TLR2) Cascade	Reactome Pathway	R-HSA-181438	6.30E-05
SMAD2/SMAD3:SMAD4 heterotrimer regulates transcription	Reactome Pathway	R-HSA-2173796	6.30E-05
Transcriptional activity of SMAD2/SMAD3:SMAD4 heterotrimer	Reactome Pathway	R-HSA-2173793	6.35E-05
RHO GTPases Activate ROCKs	Reactome Pathway	R-HSA-5627117	6.36E-05
Toll Like Receptor 9 (TLR9) Cascade	Reactome Pathway	R-HSA-168138	6.36E-05
NGF-stimulated transcription	Reactome Pathway	R-HSA-9031628	6.56E-05
Purinergic signaling in leishmaniasis infection	Reactome Pathway	R-HSA-9660826	6.76E-05
Cell recruitment (pro-inflammatory response)	Reactome Pathway	R-HSA-9664424	6.76E-05
Signaling by moderate kinase activity BRAF mutants	Reactome Pathway	R-HSA-6802946	7.64E-05
Signaling by RAS mutants	Reactome Pathway	R-HSA-6802949	7.64E-05
Paradoxical activation of RAF signaling by kinase inactive BRAF	Reactome Pathway	R-HSA-6802955	7.64E-05
Signaling downstream of RAS mutants	Reactome Pathway	R-HSA-9649948	7.64E-05
Signaling by FGFR3	Reactome Pathway	R-HSA-5654741	8.17E-05
MAP2K and MAPK activation	Reactome Pathway	R-HSA-5674135	8.17E-05
Regulation of gene expression in beta cells	Reactome Pathway	R-HSA-210745	9.06E-05
Sema4D induced cell migration and growth-cone collapse	Reactome Pathway	R-HSA-416572	9.06E-05
VEGFR2 mediated cell proliferation	Reactome Pathway	R-HSA-5218921	9.06E-05
Downstream signaling of activated FGFR4	Reactome Pathway	R-HSA-5654716	9.06E-05
Interleukin-3, Interleukin-5 and GM-CSF signaling	Reactome Pathway	R-HSA-512988	9.22E-05
TP53 Regulates Transcription of Cell Cycle Genes	Reactome Pathway	R-HSA-6791312	9.22E-05
Regulation by c-FLIP	Reactome Pathway	R-HSA-3371378	9.29E-05
Dimerization of procaspase-8	Reactome Pathway	R-HSA-69416	9.29E-05
Transcription of E2F targets under negative control by p107 (RBL1) and p130 (RBL2) in complex with HDAC1	Reactome Pathway	R-HSA-1362300	0.000106

GRB2 events in ERBB2 signaling	Reactome Pathway	R-HSA-1963640	0.000106
G1/S-Specific Transcription	Reactome Pathway	R-HSA-69205	0.000122
Deactivation of the beta-catenin transactivating complex	Reactome Pathway	R-HSA-3769402	0.000126
SHC1 events in ERBB2 signaling	Reactome Pathway	R-HSA-1250196	0.000126
Signaling by FGFR3 in disease	Reactome Pathway	R-HSA-5655332	0.000126
Signaling by FGFR3 point mutants in cancer	Reactome Pathway	R-HSA-8853338	0.000126
Signaling by ERBB2 TMD/JMD mutants	Reactome Pathway	R-HSA-9665686	0.000126
Signaling by NOTCH1 PEST Domain Mutants in Cancer	Reactome Pathway	R-HSA-2644602	0.000133
Signaling by NOTCH1 in Cancer	Reactome Pathway	R-HSA-2644603	0.000133
Constitutive Signaling by NOTCH1 PEST Domain Mutants	Reactome Pathway	R-HSA-2644606	0.000133
Signaling by NOTCH1 HD+PEST Domain Mutants in Cancer	Reactome Pathway	R-HSA-2894858	0.000133
Constitutive Signaling by NOTCH1 HD+PEST Domain Mutants	Reactome Pathway	R-HSA-2894862	0.000133
Signaling by BRAF and RAF1 fusions	Reactome Pathway	R-HSA-6802952	0.00015
MECP2 regulates neuronal receptors and channels	Reactome Pathway	R-HSA-9022699	0.000159
TP53 Regulates Transcription of Death Receptors and Ligands	Reactome Pathway	R-HSA-6803211	0.000161
Erythropoietin activates Phosphoinositide-3-kinase (PI3K)	Reactome Pathway	R-HSA-9027276	0.000161
Toll Like Receptor 10 (TLR10) Cascade	Reactome Pathway	R-HSA-168142	0.00018
Toll Like Receptor 5 (TLR5) Cascade	Reactome Pathway	R-HSA-168176	0.00018
MyD88 cascade initiated on plasma membrane	Reactome Pathway	R-HSA-975871	0.00018
Toll Like Receptor 4 (TLR4) Cascade	Reactome Pathway	R-HSA-166016	0.000212
Nuclear Events (kinase and transcription factor activation)	Reactome Pathway	R-HSA-198725	0.000226
Elastic fibre formation	Reactome Pathway	R-HSA-1566948	0.000231
DAP12 interactions	Reactome Pathway	R-HSA-2172127	0.000231
Tie2 Signaling	Reactome Pathway	R-HSA-210993	0.000234
Gastrin-CREB signalling pathway via PKC and MAPK	Reactome Pathway	R-HSA-881907	0.000234
Inflammasomes	Reactome Pathway	R-HSA-622312	0.000236
Signaling by FGFR1 in disease	Reactome Pathway	R-HSA-5655302	0.000256
Transcriptional regulation by the AP-2 (TFAP2) family of transcription factors	Reactome Pathway	R-HSA-8864260	0.000256
Ca2+ pathway	Reactome Pathway	R-HSA-4086398	0.00026
Activated NOTCH1 Transmits Signal to the Nucleus	Reactome Pathway	R-HSA-2122948	0.00026
Regulation of necroptotic cell death	Reactome Pathway	R-HSA-5675482	0.00026
p38MAPK events	Reactome Pathway	R-HSA-171007	0.00026
POU5F1 (OCT4), SOX2, NANOG activate genes related to proliferation	Reactome Pathway	R-HSA-2892247	0.00026
CASP8 activity is inhibited	Reactome Pathway	R-HSA-5218900	0.00026
Deubiquitination	Reactome Pathway	R-HSA-5688426	0.000288
Estrogen-dependent gene expression	Reactome Pathway	R-HSA-9018519	0.000288

Downstream signaling of activated FGFR3	Reactome Pathway	R-HSA-5654708	0.000311
Defective Intrinsic Pathway for Apoptosis	Reactome Pathway	R-HSA-9734009	0.000311
Transcription of E2F targets under negative control by DREAM complex	Reactome Pathway	R-HSA-1362277	0.000328
Caspase activation via Death Receptors in the presence of ligand	Reactome Pathway	R-HSA-140534	0.000328
FCERI mediated MAPK activation	Reactome Pathway	R-HSA-2871796	0.000329
Regulation of TP53 Activity through Phosphorylation	Reactome Pathway	R-HSA-6804756	0.00038
RET signaling	Reactome Pathway	R-HSA-8853659	0.000385
Senescence-Associated Secretory Phenotype (SASP)	Reactome Pathway	R-HSA-2559582	0.000397
AKT phosphorylates targets in the cytosol	Reactome Pathway	R-HSA-198323	0.000409
Regulation of TP53 Activity through Association with Co-factors	Reactome Pathway	R-HSA-6804759	0.000409
TAK1 activates NFkB by phosphorylation and activation of IKKs complex	Reactome Pathway	R-HSA-445989	0.000418
Signalling to RAS	Reactome Pathway	R-HSA-167044	0.000462
Regulation of beta-cell development	Reactome Pathway	R-HSA-186712	0.00047
Signalling to ERKs	Reactome Pathway	R-HSA-187687	0.00053
RIPK1-mediated regulated necrosis	Reactome Pathway	R-HSA-5213460	0.00053
Syndecan interactions	Reactome Pathway	R-HSA-3000170	0.000538
Interleukin receptor SHC signaling	Reactome Pathway	R-HSA-912526	0.000538
Macroautophagy	Reactome Pathway	R-HSA-1632852	0.000556
Netrin-1 signaling	Reactome Pathway	R-HSA-373752	0.000559
Signaling by FGFR1	Reactome Pathway	R-HSA-5654736	0.000559
Non-integrin membrane-ECM interactions	Reactome Pathway	R-HSA-3000171	0.000611
Signaling by NOTCH1 HD Domain Mutants in Cancer	Reactome Pathway	R-HSA-2691230	0.000611
Constitutive Signaling by NOTCH1 HD Domain Mutants	Reactome Pathway	R-HSA-2691232	0.000611
TFAP2 (AP-2) family regulates transcription of growth factors and their receptors	Reactome Pathway	R-HSA-8866910	0.000611
CTLA4 inhibitory signaling	Reactome Pathway	R-HSA-389513	0.00062
CD209 (DC-SIGN) signaling	Reactome Pathway	R-HSA-5621575	0.00062
Toll-like Receptor Cascades	Reactome Pathway	R-HSA-168898	0.00062
Transcriptional Regulation by E2F6	Reactome Pathway	R-HSA-8953750	0.000639
MAPK3 (ERK1) activation	Reactome Pathway	R-HSA-110056	0.000639
Regulation of FOXO transcriptional activity by acetylation	Reactome Pathway	R-HSA-9617629	0.000639
STAT3 nuclear events downstream of ALK signaling	Reactome Pathway	R-HSA-9701898	0.000639
Signaling by Insulin receptor	Reactome Pathway	R-HSA-74752	0.000675
S Phase	Reactome Pathway	R-HSA-69242	0.000728
Platelet degranulation	Reactome Pathway	R-HSA-114608	0.000753
Autophagy	Reactome Pathway	R-HSA-9612973	0.000791
Regulation of signaling by CBL	Reactome Pathway	R-HSA-912631	0.000829

Role of LAT2/NTAL/LAB on calcium mobilization	Reactome Pathway	R-HSA-2730905	0.000874
The NLRP3 inflammasome	Reactome Pathway	R-HSA-844456	0.000874
FOXO-mediated transcription of cell death genes	Reactome Pathway	R-HSA-9614657	0.000874
NOD1/2 Signaling Pathway	Reactome Pathway	R-HSA-168638	0.000969
MET activates RAP1 and RAC1	Reactome Pathway	R-HSA-8875555	0.00106
Activated NTRK2 signals through FRS2 and FRS3	Reactome Pathway	R-HSA-9028731	0.00106
Regulation of localization of FOXO transcription factors	Reactome Pathway	R-HSA-9614399	0.00106
Constitutive Signaling by Overexpressed ERBB2	Reactome Pathway	R-HSA-9634285	0.00106
NCAM signaling for neurite out-growth	Reactome Pathway	R-HSA-375165	0.00107
Activation of BH3-only proteins	Reactome Pathway	R-HSA-114452	0.00107
Myogenesis	Reactome Pathway	R-HSA-525793	0.00107
Downstream signaling of activated FGFR2	Reactome Pathway	R-HSA-5654696	0.00107
Signaling by CSF3 (G-CSF)	Reactome Pathway	R-HSA-9674555	0.00107
Response to elevated platelet cytosolic Ca2+	Reactome Pathway	R-HSA-76005	0.00117
RIP-mediated NFkB activation via ZBP1	Reactome Pathway	R-HSA-1810476	0.00122
SUMOylation of DNA methylation proteins	Reactome Pathway	R-HSA-4655427	0.00122
Diseases of programmed cell death	Reactome Pathway	R-HSA-9645723	0.00123
Downstream signaling of activated FGFR1	Reactome Pathway	R-HSA-5654687	0.00134
Platelet Aggregation (Plug Formation)	Reactome Pathway	R-HSA-76009	0.0014
Growth hormone receptor signaling	Reactome Pathway	R-HSA-982772	0.00141
PECAM1 interactions	Reactome Pathway	R-HSA-210990	0.00163
Caspase-mediated cleavage of cytoskeletal proteins	Reactome Pathway	R-HSA-264870	0.00163
CD28 dependent Vav1 pathway	Reactome Pathway	R-HSA-389359	0.00163
Cyclin A/B1/B2 associated events during G2/M transition	Reactome Pathway	R-HSA-69273	0.00182
TRAF6 mediated NF-kB activation	Reactome Pathway	R-HSA-933542	0.00182
Regulation of MECP2 expression and activity	Reactome Pathway	R-HSA-9022692	0.00207
G1/S DNA Damage Checkpoints	Reactome Pathway	R-HSA-69615	0.00209
MAPK6/MAPK4 signaling	Reactome Pathway	R-HSA-5687128	0.00247
GRB2 events in EGFR signaling	Reactome Pathway	R-HSA-179812	0.00247
TNFR1-induced proapoptotic signaling	Reactome Pathway	R-HSA-5357786	0.00247
Transcriptional regulation of granulopoiesis	Reactome Pathway	R-HSA-9616222	0.00274
Downregulation of SMAD2/3:SMAD4 transcriptional activity	Reactome Pathway	R-HSA-2173795	0.00296
Integrin signaling	Reactome Pathway	R-HSA-354192	0.00296
Apoptotic factor-mediated response	Reactome Pathway	R-HSA-111471	0.00306
SHC-mediated cascade:FGFR4	Reactome Pathway	R-HSA-5654719	0.00306
TP53 Regulates Transcription of Genes Involved in Cytochrome C Release	Reactome Pathway	R-HSA-6803204	0.00306

PI3K Cascade	Reactome Pathway	R-HSA-109704	0.00338
Interleukin-2 family signaling	Reactome Pathway	R-HSA-451927	0.00338
SHC1 events in ERBB4 signaling	Reactome Pathway	R-HSA-1250347	0.0035
SHC1 events in EGFR signaling	Reactome Pathway	R-HSA-180336	0.0035
Regulation of IFNG signaling	Reactome Pathway	R-HSA-877312	0.0035
PTK6 Regulates RHO GTPases, RAS GTPase and MAP kinases	Reactome Pathway	R-HSA-8849471	0.0035
Activation of NMDA receptors and postsynaptic events	Reactome Pathway	R-HSA-442755	0.00368
Signaling by FGFR2	Reactome Pathway	R-HSA-5654738	0.00382
Leishmania infection	Reactome Pathway	R-HSA-9658195	0.00388
Ub-specific processing proteases	Reactome Pathway	R-HSA-5689880	0.00392
ZBP1(DAI) mediated induction of type I IFNs	Reactome Pathway	R-HSA-1606322	0.00393
Downregulation of ERBB2 signaling	Reactome Pathway	R-HSA-8863795	0.00443
FOXO-mediated transcription of oxidative stress, metabolic and neuronal genes	Reactome Pathway	R-HSA-9615017	0.00443
Signaling by ALK in cancer	Reactome Pathway	R-HSA-9700206	0.00443
Signaling by ALK fusions and activated point mutants	Reactome Pathway	R-HSA-9725370	0.00443
Degradation of the extracellular matrix	Reactome Pathway	R-HSA-1474228	0.00481
GRB2:SOS provides linkage to MAPK signaling for Integrins	Reactome Pathway	R-HSA-354194	0.00482
Signaling by NODAL	Reactome Pathway	R-HSA-1181150	0.00496
NOTCH2 Activation and Transmission of Signal to the Nucleus	Reactome Pathway	R-HSA-2979096	0.00496
FRS-mediated FGFR4 signaling	Reactome Pathway	R-HSA-5654712	0.00496
Deregulated CDK5 triggers multiple neurodegenerative pathways in Alzheimer's disease models	Reactome Pathway	R-HSA-8862803	0.00496
Neurodegenerative Diseases	Reactome Pathway	R-HSA-8863678	0.00496
Apoptotic cleavage of cellular proteins	Reactome Pathway	R-HSA-111465	0.00503
p53-Dependent G1 DNA Damage Response	Reactome Pathway	R-HSA-69563	0.00518
p53-Dependent G1/S DNA damage checkpoint	Reactome Pathway	R-HSA-69580	0.00518
ECM proteoglycans	Reactome Pathway	R-HSA-3000178	0.00518
Signaling by FGFR	Reactome Pathway	R-HSA-190236	0.00556
TP53 Regulates Metabolic Genes	Reactome Pathway	R-HSA-5628897	0.00556
Interleukin-12 family signaling	Reactome Pathway	R-HSA-447115	0.00563
Pre-NOTCH Expression and Processing	Reactome Pathway	R-HSA-1912422	0.00581
Ovarian tumor domain proteases	Reactome Pathway	R-HSA-5689896	0.00586
NOTCH1 Intracellular Domain Regulates Transcription	Reactome Pathway	R-HSA-2122947	0.00589
PI3K events in ERBB2 signaling	Reactome Pathway	R-HSA-1963642	0.00631
TGF-beta receptor signaling in EMT (epithelial to mesenchymal transition)	Reactome Pathway	R-HSA-2173791	0.00631
Sema3A PAK dependent Axon repulsion	Reactome Pathway	R-HSA-399954	0.00631

Signaling by WNT	Reactome Pathway	R-HSA-195721	0.00633
Activation of PPARGC1A (PGC-1alpha) by phosphorylation	Reactome Pathway	R-HSA-2151209	0.00669
Caspase activation via Dependence Receptors in the absence of ligand	Reactome Pathway	R-HSA-418889	0.00669
Activation of the AP-1 family of transcription factors	Reactome Pathway	R-HSA-450341	0.00669
STAT5 activation downstream of FLT3 ITD mutants	Reactome Pathway	R-HSA-9702518	0.00669
Signaling by the B Cell Receptor (BCR)	Reactome Pathway	R-HSA-983705	0.0074
Nuclear signaling by ERBB4	Reactome Pathway	R-HSA-1251985	0.00748
G-protein beta:gamma signalling	Reactome Pathway	R-HSA-397795	0.00748
MicroRNA (miRNA) biogenesis	Reactome Pathway	R-HSA-203927	0.00751
Other interleukin signaling	Reactome Pathway	R-HSA-449836	0.00751
RHO GTPases activate PAKs	Reactome Pathway	R-HSA-5627123	0.00751
DNA Damage/Telomere Stress Induced Senescence	Reactome Pathway	R-HSA-2559586	0.00761
Post NMDA receptor activation events	Reactome Pathway	R-HSA-438064	0.00761
Circadian Clock	Reactome Pathway	R-HSA-400253	0.00786
Interferon alpha/beta signaling	Reactome Pathway	R-HSA-909733	0.00786
Signaling by NOTCH2	Reactome Pathway	R-HSA-1980145	0.00882
RND3 GTPase cycle	Reactome Pathway	R-HSA-9696264	0.00904
Role of phospholipids in phagocytosis	Reactome Pathway	R-HSA-2029485	0.0091
Basigin interactions	Reactome Pathway	R-HSA-210991	0.0091
Xenobiotics	Reactome Pathway	R-HSA-211981	0.0091
NOTCH3 Activation and Transmission of Signal to the Nucleus	Reactome Pathway	R-HSA-9013507	0.0091
Inactivation of CSF3 (G-CSF) signaling	Reactome Pathway	R-HSA-9705462	0.0091
Pre-NOTCH Transcription and Translation	Reactome Pathway	R-HSA-1912408	0.00932
Regulation of gene expression by Hypoxia-inducible Factor	Reactome Pathway	R-HSA-1234158	0.00932
Regulated proteolysis of p75NTR	Reactome Pathway	R-HSA-193692	0.00932
Signaling by Leptin	Reactome Pathway	R-HSA-2586552	0.00932
DSCAM interactions	Reactome Pathway	R-HSA-376172	0.00932
RHO GTPases activate KTN1	Reactome Pathway	R-HSA-5625970	0.00932
Receptor Mediated Mitophagy	Reactome Pathway	R-HSA-8934903	0.00932
NOTCH4 Activation and Transmission of Signal to the Nucleus	Reactome Pathway	R-HSA-9013700	0.00932
Apoptotic execution phase	Reactome Pathway	R-HSA-75153	0.00949
Signaling by FGFR2 in disease	Reactome Pathway	R-HSA-5655253	0.0101
SHC-mediated cascade:FGFR3	Reactome Pathway	R-HSA-5654704	0.0102
Cargo recognition for clathrin-mediated endocytosis	Reactome Pathway	R-HSA-8856825	0.0108
Signaling by FGFR in disease	Reactome Pathway	R-HSA-1226099	0.0108
Cytosolic sensors of pathogen-associated DNA	Reactome Pathway	R-HSA-1834949	0.0121

MAP kinase activation	Reactome Pathway	R-HSA-450294	0.0121
C-type lectin receptors (CLRs)	Reactome Pathway	R-HSA-5621481	0.0127
Ephrin signaling	Reactome Pathway	R-HSA-3928664	0.0129
Regulation of TP53 Activity through Methylation	Reactome Pathway	R-HSA-6804760	0.0129
Repression of WNT target genes	Reactome Pathway	R-HSA-4641265	0.0129
TP53 Regulates Transcription of Caspase Activators and Caspases	Reactome Pathway	R-HSA-6803207	0.0129
RAF activation	Reactome Pathway	R-HSA-5673000	0.0137
TNF signaling	Reactome Pathway	R-HSA-75893	0.0151
Beta-catenin independent WNT signaling	Reactome Pathway	R-HSA-3858494	0.0154
Signaling by BMP	Reactome Pathway	R-HSA-201451	0.0156
NR1H3 & NR1H2 regulate gene expression linked to cholesterol transport and efflux	Reactome Pathway	R-HSA-9029569	0.0159
SUMOylation of transcription factors	Reactome Pathway	R-HSA-3232118	0.016
RHO GTPases activate CIT	Reactome Pathway	R-HSA-5625900	0.016
FRS-mediated FGFR3 signaling	Reactome Pathway	R-HSA-5654706	0.016
Interleukin-12 signaling	Reactome Pathway	R-HSA-9020591	0.0169
NR1H2 and NR1H3-mediated signaling	Reactome Pathway	R-HSA-9024446	0.0169
Heme signaling	Reactome Pathway	R-HSA-9707616	0.0169
Cytochrome c-mediated apoptotic response	Reactome Pathway	R-HSA-111461	0.0172
Downregulation of ERBB2:ERBB3 signaling	Reactome Pathway	R-HSA-1358803	0.0172
RHOH GTPase cycle	Reactome Pathway	R-HSA-9013407	0.0181
RHOV GTPase cycle	Reactome Pathway	R-HSA-9013424	0.0181
Transcriptional regulation by RUNX1	Reactome Pathway	R-HSA-8878171	0.0194
SHC-mediated cascade:FGFR1	Reactome Pathway	R-HSA-5654688	0.0194
Negative regulation of MET activity	Reactome Pathway	R-HSA-6807004	0.0194
Interferon gamma signaling	Reactome Pathway	R-HSA-877300	0.0196
Signaling by NOTCH3	Reactome Pathway	R-HSA-9012852	0.0212
TNFR1-induced NFkappaB signaling pathway	Reactome Pathway	R-HSA-5357956	0.0212
Regulation of TP53 Activity through Acetylation	Reactome Pathway	R-HSA-6804758	0.0212
SEMA3A-Plexin repulsion signaling by inhibiting Integrin adhesion	Reactome Pathway	R-HSA-399955	0.0221
DCC mediated attractive signaling	Reactome Pathway	R-HSA-418885	0.0221
Activation of RAC1	Reactome Pathway	R-HSA-428540	0.0221
TP53 regulates transcription of several additional cell death genes whose specific roles in p53-dependent apoptosis remain uncertain	Reactome Pathway	R-HSA-6803205	0.0221
RUNX3 regulates NOTCH signaling	Reactome Pathway	R-HSA-8941856	0.0221
Interleukin-15 signaling	Reactome Pathway	R-HSA-8983432	0.0221
RHOU GTPase cycle	Reactome Pathway	R-HSA-9013420	0.0233

Listeria monocytogenes entry into host cells	Reactome Pathway	R-HSA-8876384	0.0233
Interleukin-1 family signaling	Reactome Pathway	R-HSA-446652	0.0233
Cell-Cell communication	Reactome Pathway	R-HSA-1500931	0.0236
DNA Double-Strand Break Repair	Reactome Pathway	R-HSA-5693532	0.0249
Translocation of SLC2A4 (GLUT4) to the plasma membrane	Reactome Pathway	R-HSA-1445148	0.0254
Interleukin-17 signaling	Reactome Pathway	R-HSA-448424	0.0254
Nephrin family interactions	Reactome Pathway	R-HSA-373753	0.0276
FRS-mediated FGFR1 signaling	Reactome Pathway	R-HSA-5654693	0.0276
SHC-mediated cascade:FGFR2	Reactome Pathway	R-HSA-5654699	0.0276
RHOBTB1 GTPase cycle	Reactome Pathway	R-HSA-9013422	0.0276
Activation of BAD and translocation to mitochondria	Reactome Pathway	R-HSA-111447	0.0276
Signaling by Activin	Reactome Pathway	R-HSA-1502540	0.0276
YAP1- and WWTR1 (TAZ)-stimulated gene expression	Reactome Pathway	R-HSA-2032785	0.0276
p130Cas linkage to MAPK signaling for integrins	Reactome Pathway	R-HSA-372708	0.0276
ERBB2 Regulates Cell Motility	Reactome Pathway	R-HSA-6785631	0.0276
RUNX2 regulates bone development	Reactome Pathway	R-HSA-8941326	0.0277
Integrin cell surface interactions	Reactome Pathway	R-HSA-216083	0.0291
Clathrin-mediated endocytosis	Reactome Pathway	R-HSA-8856828	0.031
Diseases of Immune System	Reactome Pathway	R-HSA-5260271	0.0319
Diseases associated with the TLR signaling cascade	Reactome Pathway	R-HSA-5602358	0.0319
activated TAK1 mediates p38 MAPK activation	Reactome Pathway	R-HSA-450302	0.0325
Negative regulation of MAPK pathway	Reactome Pathway	R-HSA-5675221	0.0327
FCGR3A-mediated IL10 synthesis	Reactome Pathway	R-HSA-9664323	0.0327
Polo-like kinase mediated events	Reactome Pathway	R-HSA-156711	0.0343
Formation of Senescence-Associated Heterochromatin Foci (SAHF)	Reactome Pathway	R-HSA-2559584	0.0343
G beta:gamma signalling through PI3Kgamma	Reactome Pathway	R-HSA-392451	0.0381
FRS-mediated FGFR2 signaling	Reactome Pathway	R-HSA-5654700	0.0381
Cytochrome P450 - arranged by substrate type	Reactome Pathway	R-HSA-211897	0.0391
CLEC7A (Dectin-1) signaling	Reactome Pathway	R-HSA-5607764	0.0398
Negative regulators of DDX58/IFIH1 signaling	Reactome Pathway	R-HSA-936440	0.0414
RHOBTB GTPase Cycle	Reactome Pathway	R-HSA-9706574	0.0414
GAB1 signalosome	Reactome Pathway	R-HSA-180292	0.0419
IL-6-type cytokine receptor ligand interactions	Reactome Pathway	R-HSA-6788467	0.0419
Biosynthesis of DHA-derived SPMs	Reactome Pathway	R-HSA-9018677	0.0419
Regulation of IFNA signaling	Reactome Pathway	R-HSA-912694	0.0442
Interleukin-1 signaling	Reactome Pathway	R-HSA-9020702	0.0452

Interleukin-7 signaling	Reactome Pathway	R-HSA-1266695	0.0466
PI3K events in ERBB4 signaling	Reactome Pathway	R-HSA-1250342	0.0484
TRIF-mediated programmed cell death	Reactome Pathway	R-HSA-2562578	0.0484
CDC6 association with the ORC:origin complex	Reactome Pathway	R-HSA-68689	0.0484
Signal attenuation	Reactome Pathway	R-HSA-74749	0.0484
MET receptor recycling	Reactome Pathway	R-HSA-8875656	0.0484
RHOBTB3 ATPase cycle	Reactome Pathway	R-HSA-9706019	0.0484
Recycling pathway of L1	Reactome Pathway	R-HSA-437239	0.0498
Signaling by cytosolic FGFR1 fusion mutants	Reactome Pathway	R-HSA-1839117	0.05
TNF receptor superfamily (TNFSF) members mediating non-canonical NF-kB pathway	Reactome Pathway	R-HSA-5676594	0.05
TP53 Regulates Transcription of Genes Involved in G2 Cell Cycle Arrest	Reactome Pathway	R-HSA-6804114	0.05
AGE-RAGE signaling pathway in diabetic complications	KEGG Pathway	hsa04933	3.01E-33
Proteoglycans in cancer	KEGG Pathway	hsa05205	2.90E-31
Cellular senescence	KEGG Pathway	hsa04218	1.94E-29
MicroRNAs in cancer	KEGG Pathway	hsa05206	1.61E-28
Hepatitis B	KEGG Pathway	hsa05161	1.28E-27
Prostate cancer	KEGG Pathway	hsa05215	1.24E-26
Lipid and atherosclerosis	KEGG Pathway	hsa05417	5.95E-26
Kaposi sarcoma-associated herpesvirus infection	KEGG Pathway	hsa05167	1.61E-25
TNF signaling pathway	KEGG Pathway	hsa04668	1.62E-25
Human cytomegalovirus infection	KEGG Pathway	hsa05163	8.28E-25
PI3K-Akt signaling pathway	KEGG Pathway	hsa04151	1.72E-24
Pancreatic cancer	KEGG Pathway	hsa05212	2.02E-24
Endocrine resistance	KEGG Pathway	hsa01522	1.83E-23
Chronic myeloid leukemia	KEGG Pathway	hsa05220	3.62E-22
EGFR tyrosine kinase inhibitor resistance	KEGG Pathway	hsa01521	1.92E-21
Colorectal cancer	KEGG Pathway	hsa05210	6.35E-21
Human T-cell leukemia virus 1 infection	KEGG Pathway	hsa05166	1.05E-20
FoxO signaling pathway	KEGG Pathway	hsa04068	2.23E-20
Epstein-Barr virus infection	KEGG Pathway	hsa05169	5.55E-20
Small cell lung cancer	KEGG Pathway	hsa05222	9.49E-20
Bladder cancer	KEGG Pathway	hsa05219	1.17E-19
Human papillomavirus infection	KEGG Pathway	hsa05165	1.78E-19
MAPK signaling pathway	KEGG Pathway	hsa04010	2.21E-19
Breast cancer	KEGG Pathway	hsa05224	5.31E-19
p53 signaling pathway	KEGG Pathway	hsa04115	9.95E-19

Osteoclast differentiation	KEGG Pathway	hsa04380	2.41E-18
Melanoma	KEGG Pathway	hsa05218	6.14E-18
Non-small cell lung cancer	KEGG Pathway	hsa05223	6.14E-18
Hepatocellular carcinoma	KEGG Pathway	hsa05225	3.28E-17
Gastric cancer	KEGG Pathway	hsa05226	3.43E-17
Measles	KEGG Pathway	hsa05162	6.99E-17
Focal adhesion	KEGG Pathway	hsa04510	1.29E-16
Signaling pathways regulating pluripotency of stem cells	KEGG Pathway	hsa04550	2.16E-16
Glioma	KEGG Pathway	hsa05214	2.37E-16
C-type lectin receptor signaling pathway	KEGG Pathway	hsa04625	5.58E-16
Endometrial cancer	KEGG Pathway	hsa05213	6.51E-16
Yersinia infection	KEGG Pathway	hsa05135	1.29E-15
Chemical carcinogenesis - receptor activation	KEGG Pathway	hsa05207	1.29E-15
Hepatitis C	KEGG Pathway	hsa05160	1.46E-15
Transcriptional misregulation in cancer	KEGG Pathway	hsa05202	1.71E-15
Prolactin signaling pathway	KEGG Pathway	hsa04917	1.98E-15
Acute myeloid leukemia	KEGG Pathway	hsa05221	4.85E-15
PD-L1 expression and PD-1 checkpoint pathway in cancer	KEGG Pathway	hsa05235	7.13E-15
Renal cell carcinoma	KEGG Pathway	hsa05211	1.16E-14
Apoptosis	KEGG Pathway	hsa04210	2.99E-14
Longevity regulating pathway	KEGG Pathway	hsa04211	5.26E-14
Cell cycle	KEGG Pathway	hsa04110	6.41E-14
Chagas disease	KEGG Pathway	hsa05142	7.96E-14
HIF-1 signaling pathway	KEGG Pathway	hsa04066	1.05E-13
T cell receptor signaling pathway	KEGG Pathway	hsa04660	1.44E-13
Toxoplasmosis	KEGG Pathway	hsa05145	2.48E-13
Platinum drug resistance	KEGG Pathway	hsa01524	4.78E-13
Chemokine signaling pathway	KEGG Pathway	hsa04062	6.59E-13
Shigellosis	KEGG Pathway	hsa05131	7.56E-13
Human immunodeficiency virus 1 infection	KEGG Pathway	hsa05170	1.60E-12
Fluid shear stress and atherosclerosis	KEGG Pathway	hsa05418	1.60E-12
Adherens junction	KEGG Pathway	hsa04520	1.67E-12
Non-alcoholic fatty liver disease	KEGG Pathway	hsa04932	2.45E-12
Autophagy - animal	KEGG Pathway	hsa04140	2.45E-12
Alcoholic liver disease	KEGG Pathway	hsa04936	3.06E-12
Influenza A	KEGG Pathway	hsa05164	3.31E-12

Viral carcinogenesis	KEGG Pathway	hsa05203	5.64E-12
Sphingolipid signaling pathway	KEGG Pathway	hsa04071	8.15E-12
Thyroid hormone signaling pathway	KEGG Pathway	hsa04919	1.33E-11
Insulin resistance	KEGG Pathway	hsa04931	1.39E-11
Relaxin signaling pathway	KEGG Pathway	hsa04926	1.74E-11
Toll-like receptor signaling pathway	KEGG Pathway	hsa04620	2.65E-11
Ras signaling pathway	KEGG Pathway	hsa04014	4.04E-11
Neurotrophin signaling pathway	KEGG Pathway	hsa04722	4.04E-11
JAK-STAT signaling pathway	KEGG Pathway	hsa04630	4.24E-11
AMPK signaling pathway	KEGG Pathway	hsa04152	5.01E-11
Central carbon metabolism in cancer	KEGG Pathway	hsa05230	5.64E-11
Rap1 signaling pathway	KEGG Pathway	hsa04015	2.10E-10
Adipocytokine signaling pathway	KEGG Pathway	hsa04920	2.83E-10
Hippo signaling pathway	KEGG Pathway	hsa04390	2.83E-10
TGF-beta signaling pathway	KEGG Pathway	hsa04350	3.03E-10
Salmonella infection	KEGG Pathway	hsa05132	4.76E-10
NF-kappa B signaling pathway	KEGG Pathway	hsa04064	7.05E-10
ErbB signaling pathway	KEGG Pathway	hsa04012	8.44E-10
Growth hormone synthesis, secretion and action	KEGG Pathway	hsa04935	8.99E-10
Longevity regulating pathway - multiple species	KEGG Pathway	hsa04213	1.25E-09
Fc epsilon RI signaling pathway	KEGG Pathway	hsa04664	1.29E-09
IL-17 signaling pathway	KEGG Pathway	hsa04657	1.56E-09
Th17 cell differentiation	KEGG Pathway	hsa04659	1.71E-09
VEGF signaling pathway	KEGG Pathway	hsa04370	3.11E-09
Cushing syndrome	KEGG Pathway	hsa04934	1.19E-08
Tuberculosis	KEGG Pathway	hsa05152	1.35E-08
Cytokine-cytokine receptor interaction	KEGG Pathway	hsa04060	1.59E-08
Thyroid cancer	KEGG Pathway	hsa05216	2.28E-08
NOD-like receptor signaling pathway	KEGG Pathway	hsa04621	2.42E-08
Pathogenic Escherichia coli infection	KEGG Pathway	hsa05130	4.67E-08
B cell receptor signaling pathway	KEGG Pathway	hsa04662	5.83E-08
Insulin signaling pathway	KEGG Pathway	hsa04910	1.14E-07
Viral protein interaction with cytokine and cytokine receptor	KEGG Pathway	hsa04061	1.38E-07
Axon guidance	KEGG Pathway	hsa04360	2.00E-07
Amoebiasis	KEGG Pathway	hsa05146	2.03E-07
cAMP signaling pathway	KEGG Pathway	hsa04024	3.27E-07

Malaria	KEGG Pathway	hsa05144	3.34E-07
Leishmaniasis	KEGG Pathway	hsa05140	4.34E-07
Apelin signaling pathway	KEGG Pathway	hsa04371	5.52E-07
Inflammatory bowel disease	KEGG Pathway	hsa05321	6.26E-07
Regulation of actin cytoskeleton	KEGG Pathway	hsa04810	6.45E-07
Mitophagy - animal	KEGG Pathway	hsa04137	6.52E-07
Chemical carcinogenesis - reactive oxygen species	KEGG Pathway	hsa05208	1.14E-06
Pertussis	KEGG Pathway	hsa05133	1.60E-06
Leukocyte transendothelial migration	KEGG Pathway	hsa04670	1.69E-06
Apoptosis - multiple species	KEGG Pathway	hsa04215	1.73E-06
Alzheimer disease	KEGG Pathway	hsa05010	1.74E-06
Epithelial cell signaling in Helicobacter pylori infection	KEGG Pathway	hsa05120	1.95E-06
Th1 and Th2 cell differentiation	KEGG Pathway	hsa04658	2.02E-06
Legionellosis	KEGG Pathway	hsa05134	2.20E-06
Diabetic cardiomyopathy	KEGG Pathway	hsa05415	2.52E-06
Progesterone-mediated oocyte maturation	KEGG Pathway	hsa04914	3.02E-06
Phospholipase D signaling pathway	KEGG Pathway	hsa04072	6.19E-06
Natural killer cell mediated cytotoxicity	KEGG Pathway	hsa04650	6.21E-06
Platelet activation	KEGG Pathway	hsa04611	7.55E-06
Rheumatoid arthritis	KEGG Pathway	hsa05323	9.35E-06
Autophagy - other	KEGG Pathway	hsa04136	1.25E-05
Type II diabetes mellitus	KEGG Pathway	hsa04930	2.04E-05
Wnt signaling pathway	KEGG Pathway	hsa04310	2.10E-05
Choline metabolism in cancer	KEGG Pathway	hsa05231	2.10E-05
Necroptosis	KEGG Pathway	hsa04217	2.35E-05
Bacterial invasion of epithelial cells	KEGG Pathway	hsa05100	3.27E-05
RIG-I-like receptor signaling pathway	KEGG Pathway	hsa04622	3.65E-05
Regulation of lipolysis in adipocytes	KEGG Pathway	hsa04923	4.14E-05
Coronavirus disease - COVID-19	KEGG Pathway	hsa05171	4.59E-05
Pathways of neurodegeneration - multiple diseases	KEGG Pathway	hsa05022	5.02E-05
Estrogen signaling pathway	KEGG Pathway	hsa04915	0.000137
cGMP-PKG signaling pathway	KEGG Pathway	hsa04022	0.000152
Ferroptosis	KEGG Pathway	hsa04216	0.000157
Cholinergic synapse	KEGG Pathway	hsa04725	0.000169
Oxytocin signaling pathway	KEGG Pathway	hsa04921	0.000277
mTOR signaling pathway	KEGG Pathway	hsa04150	0.000304

African trypanosomiasis	KEGG Pathway	hsa05143	0.000304
Tight junction	KEGG Pathway	hsa04530	0.000459
Fc gamma R-mediated phagocytosis	KEGG Pathway	hsa04666	0.000627
GnRH signaling pathway	KEGG Pathway	hsa04912	0.00115
Notch signaling pathway	KEGG Pathway	hsa04330	0.00119
Long-term depression	KEGG Pathway	hsa04730	0.00139
Parathyroid hormone synthesis, secretion and action	KEGG Pathway	hsa04928	0.00176
Gap junction	KEGG Pathway	hsa04540	0.00183
Inflammatory mediator regulation of TRP channels	KEGG Pathway	hsa04750	0.00201
Basal cell carcinoma	KEGG Pathway	hsa05217	0.00213
Neutrophil extracellular trap formation	KEGG Pathway	hsa04613	0.00254
Spinocerebellar ataxia	KEGG Pathway	hsa05017	0.00361
Arrhythmogenic right ventricular cardiomyopathy	KEGG Pathway	hsa05412	0.00417
Viral myocarditis	KEGG Pathway	hsa05416	0.00457
Aldosterone-regulated sodium reabsorption	KEGG Pathway	hsa04960	0.0061
Cytosolic DNA-sensing pathway	KEGG Pathway	hsa04623	0.00671
GnRH secretion	KEGG Pathway	hsa04929	0.00757
Glucagon signaling pathway	KEGG Pathway	hsa04922	0.0121
Graft-versus-host disease	KEGG Pathway	hsa05332	0.0134
Hematopoietic cell lineage	KEGG Pathway	hsa04640	0.0143
Oocyte meiosis	KEGG Pathway	hsa04114	0.0155
Dopaminergic synapse	KEGG Pathway	hsa04728	0.0166
Serotonergic synapse	KEGG Pathway	hsa04726	0.0224
Prion disease	KEGG Pathway	hsa05020	0.0233
Carbohydrate digestion and absorption	KEGG Pathway	hsa04973	0.0254
Adrenergic signaling in cardiomyocytes	KEGG Pathway	hsa04261	0.0265
Amphetamine addiction	KEGG Pathway	hsa05031	0.0342
Hypertrophic cardiomyopathy	KEGG Pathway	hsa05410	0.0358
Melanogenesis	KEGG Pathway	hsa04916	0.037
Ovarian steroidogenesis	KEGG Pathway	hsa04913	0.0392
Endocytosis	KEGG Pathway	hsa04144	0.0432
Type I diabetes mellitus	KEGG Pathway	hsa04940	0.0457
Hepatocellular carcinoma	KEGG Disease	H00048	6.60E-09
Gastric cancer	KEGG Disease	H00018	1.20E-08
Penile cancer	KEGG Disease	H00025	7.38E-08
Glioma	KEGG Disease	H00042	7.38E-08

Breast cancer	KEGG Disease	H00031	8.40E-07
Esophageal cancer	KEGG Disease	H00017	1.30E-06
Non-small cell lung cancer	KEGG Disease	H00014	2.27E-06
Ovarian cancer	KEGG Disease	H00027	2.27E-06
Myelodysplastic/myeloproliferative neoplasms	KEGG Disease	H02410	3.89E-06
Chronic myelomonocytic leukemia	KEGG Disease	H02411	3.89E-06
Pancreatic cancer	KEGG Disease	H00019	2.45E-05
Atypical chronic myeloid leukemia	KEGG Disease	H02412	2.57E-05
Colorectal cancer	KEGG Disease	H00020	3.31E-05
Diffuse large B-cell lymphoma, not otherwise specified	KEGG Disease	H02434	3.31E-05
Melanoma	KEGG Disease	H00038	3.78E-05
Graft-versus-host disease	KEGG Disease	H00084	3.78E-05
Thyroid cancer	KEGG Disease	H00032	0.000356
Noonan syndrome and related disorders	KEGG Disease	H00523	0.000356
Medulloblastoma	KEGG Disease	H01667	0.000356
Inflammatory bowel disease (IBD)	KEGG Disease	H01227	0.000462
Acute myeloid leukemia	KEGG Disease	H00003	0.000462
Myelodysplastic syndrome	KEGG Disease	H01481	0.00121
Allograft rejection	KEGG Disease	H00083	0.00271
Meningioma	KEGG Disease	H01556	0.00271
Osteoporosis	KEGG Disease	H01593	0.00641
Malignant paraganglioma	KEGG Disease	H01510	0.016
Cushing syndrome	KEGG Disease	H01431	0.0224
Allergic rhinitis	KEGG Disease	H01360	0.0359
Brain cancer	TTD Disease	ICD-11[2A00]	1.72E-07
Lung cancer	TTD Disease	ICD-11[2C25]	5.38E-07
Multiple myeloma	TTD Disease	ICD-11[2A83]	2.00E-06
Malignant haematopoietic neoplasm	TTD Disease	ICD-11[2B33]	2.00E-06
Breast cancer	TTD Disease	ICD-11[2C60-2C6Y]	3.70E-05
Pancreatic cancer	TTD Disease	ICD-11[2C10]	7.53E-05
Rheumatoid arthritis	TTD Disease	ICD-11[FA20]	0.000186
Prostate cancer	TTD Disease	ICD-11[2C82]	0.000572
Diffuse large B-cell lymphoma	TTD Disease	ICD-11[2A81]	0.0012
Renal cell carcinoma	TTD Disease	ICD-11[2C90]	0.002
Myeloproliferative neoplasm	TTD Disease	ICD-11[2A20]	0.00244
Acute myeloid leukaemia	TTD Disease	ICD-11[2A60]	0.0028

Ovarian cancer	TTD Disease	ICD-11[2C73]	0.00297
Liver cancer	TTD Disease	ICD-11[2C12]	0.00299
Colorectal cancer	TTD Disease	ICD-11[2B91]	0.00304
Myelodysplastic syndrome	TTD Disease	ICD-11[2A37]	0.00373
Systemic sclerosis	TTD Disease	ICD-11[4A42]	0.00485
Melanoma	TTD Disease	ICD-11[2C30]	0.0103
Nasopharyngeal cancer	TTD Disease	ICD-11[2B6B]	0.0103
Crohn disease	TTD Disease	ICD-11[DD70]	0.0129
Head and neck cancer	TTD Disease	ICD-11[2D42]	0.0189
Ulcerative colitis	TTD Disease	ICD-11[DD71]	0.021
Diabetes mellitus	TTD Disease	ICD-11[5A10]	0.0218
Idiopathic interstitial pneumonitis	TTD Disease	ICD-11[CB03]	0.0218
Lymphoma	TTD Disease	ICD-11[2A80-2A86]	0.0218
Motor neuron disease	TTD Disease	ICD-11[8B60]	0.0218
Stomach cancer	TTD Disease	ICD-11[2B72]	0.022
Mature B-cell leukaemia	TTD Disease	ICD-11[2A82]	0.022
Colon cancer	TTD Disease	ICD-11[2B90]	0.022
Bladder cancer	TTD Disease	ICD-11[2C94]	0.022
COVID-19	TTD Disease	ICD-11[1D6Y]	0.022
Peritoneal cancer	TTD Disease	ICD-11[2C51]	0.0259
Chronic obstructive pulmonary disease	TTD Disease	ICD-11[CA22]	0.0296
Postoperative inflammation	TTD Disease	ICD-11[1A00-CA43]	0.0301
Immune system disease	TTD Disease	ICD-11[4A01-4B41]	0.0314
Metastatic lymph node neoplasm	TTD Disease	ICD-11[2D60]	0.0331
Psoriasis	TTD Disease	ICD-11[EA90]	0.0339
Osteoarthritis	TTD Disease	ICD-11[FA00-FA05]	0.0344
Retinopathy	TTD Disease	ICD-11[9B71]	0.0358
Sarcoma	TTD Disease	ICD-11[2A60-2C35]	0.0373
Type 2 diabetes mellitus	TTD Disease	ICD-11[5A11]	0.0496
epithelial cell proliferation	Biological Process	GO:0050673	3.65E-40
gland development	Biological Process	GO:0048732	1.39E-39
T cell activation	Biological Process	GO:0042110	3.62E-37
response to oxygen levels	Biological Process	GO:0070482	1.28E-35
regulation of epithelial cell proliferation	Biological Process	GO:0050678	1.53E-34
cellular response to chemical stress	Biological Process	GO:0062197	6.04E-32
response to decreased oxygen levels	Biological Process	GO:0036293	6.04E-32

reproductive structure development	Biological Process	GO:0048608	3.62E-31
reproductive system development	Biological Process	GO:0061458	5.46E-31
neuron death	Biological Process	GO:0070997	1.34E-30
regulation of vasculature development	Biological Process	GO:1901342	2.18E-30
response to hypoxia	Biological Process	GO:0001666	9.84E-30
regulation of cell-cell adhesion	Biological Process	GO:0022407	1.38E-29
regulation of apoptotic signaling pathway	Biological Process	GO:2001233	1.65E-28
response to oxidative stress	Biological Process	GO:0006979	2.14E-28
cellular response to oxidative stress	Biological Process	GO:0034599	5.15E-28
muscle cell proliferation	Biological Process	GO:0033002	6.21E-28
positive regulation of epithelial cell proliferation	Biological Process	GO:0050679	7.65E-28
positive regulation of neurogenesis	Biological Process	GO:0050769	1.10E-27
cellular response to drug	Biological Process	GO:0035690	1.76E-27
myeloid cell differentiation	Biological Process	GO:0030099	3.55E-27
regulation of neuron death	Biological Process	GO:1901214	3.55E-27
response to mechanical stimulus	Biological Process	GO:0009612	1.59E-26
regulation of angiogenesis	Biological Process	GO:0045765	1.59E-26
response to steroid hormone	Biological Process	GO:0048545	1.59E-26
extrinsic apoptotic signaling pathway	Biological Process	GO:0097191	5.48E-26
positive regulation of cell adhesion	Biological Process	GO:0045785	6.89E-26
gliogenesis	Biological Process	GO:0042063	6.89E-26
positive regulation of cell-cell adhesion	Biological Process	GO:0022409	1.23E-25
regulation of DNA-binding transcription factor activity	Biological Process	GO:0051090	1.87E-25
negative regulation of apoptotic signaling pathway	Biological Process	GO:2001234	1.94E-25
positive regulation of catabolic process	Biological Process	GO:0009896	3.34E-25
cell growth	Biological Process	GO:0016049	4.33E-25
leukocyte cell-cell adhesion	Biological Process	GO:0007159	1.39E-24
response to antibiotic	Biological Process	GO:0046677	1.43E-24
ERK1 and ERK2 cascade	Biological Process	GO:0070371	1.45E-24
response to lipopolysaccharide	Biological Process	GO:0032496	2.32E-24
cellular response to peptide	Biological Process	GO:1901653	3.07E-24
response to molecule of bacterial origin	Biological Process	GO:0002237	3.46E-24
regulation of leukocyte cell-cell adhesion	Biological Process	GO:1903037	5.04E-24
regulation of MAP kinase activity	Biological Process	GO:0043405	7.17E-24
leukocyte proliferation	Biological Process	GO:0070661	1.03E-23
regulation of hemopoiesis	Biological Process	GO:1903706	1.25E-23

positive regulation of cellular catabolic process	Biological Process	GO:0031331	1.25E-23
cellular response to abiotic stimulus	Biological Process	GO:0071214	1.39E-23
cellular response to environmental stimulus	Biological Process	GO:0104004	1.39E-23
positive regulation of protein serine/threonine kinase activity	Biological Process	GO:0071902	2.29E-23
regulation of smooth muscle cell proliferation	Biological Process	GO:0048660	6.85E-23
protein kinase B signaling	Biological Process	GO:0043491	7.02E-23
regulation of ERK1 and ERK2 cascade	Biological Process	GO:0070372	8.14E-23
lymphocyte differentiation	Biological Process	GO:0030098	8.47E-23
negative regulation of phosphorylation	Biological Process	GO:0042326	1.02E-22
smooth muscle cell proliferation	Biological Process	GO:0048659	1.06E-22
response to peptide hormone	Biological Process	GO:0043434	1.54E-22
ameboidal-type cell migration	Biological Process	GO:0001667	2.14E-22
response to nutrient levels	Biological Process	GO:0031667	2.34E-22
leukocyte migration	Biological Process	GO:0050900	2.34E-22
urogenital system development	Biological Process	GO:0001655	3.41E-22
peptidyl-tyrosine phosphorylation	Biological Process	GO:0018108	3.61E-22
response to acid chemical	Biological Process	GO:0001101	4.78E-22
myeloid leukocyte differentiation	Biological Process	GO:0002573	5.20E-22
response to reactive oxygen species	Biological Process	GO:0000302	5.32E-22
peptidyl-tyrosine modification	Biological Process	GO:0018212	5.32E-22
cell fate commitment	Biological Process	GO:0045165	5.32E-22
response to radiation	Biological Process	GO:0009314	6.73E-22
regulation of developmental growth	Biological Process	GO:0048638	8.26E-22
regulation of protein kinase B signaling	Biological Process	GO:0051896	8.31E-22
response to transforming growth factor beta	Biological Process	GO:0071559	1.05E-21
homeostasis of number of cells	Biological Process	GO:0048872	1.20E-21
glial cell differentiation	Biological Process	GO:0010001	1.33E-21
positive regulation of vasculature development	Biological Process	GO:1904018	2.27E-21
T cell differentiation	Biological Process	GO:0030217	2.42E-21
epithelial tube morphogenesis	Biological Process	GO:0060562	2.50E-21
mononuclear cell proliferation	Biological Process	GO:0032943	5.23E-21
cellular response to external stimulus	Biological Process	GO:0071496	6.56E-21
regulation of extrinsic apoptotic signaling pathway	Biological Process	GO:2001236	7.41E-21
muscle tissue development	Biological Process	GO:0060537	7.46E-21
positive regulation of leukocyte cell-cell adhesion	Biological Process	GO:1903039	8.86E-21
intrinsic apoptotic signaling pathway	Biological Process	GO:0097193	1.12E-20

neuron apoptotic process	Biological Process	GO:0051402	1.24E-20
regulation of T cell activation	Biological Process	GO:0050863	2.06E-20
lymphocyte proliferation	Biological Process	GO:0046651	2.06E-20
positive regulation of cytokine production	Biological Process	GO:0001819	2.06E-20
positive regulation of angiogenesis	Biological Process	GO:0045766	2.26E-20
negative regulation of protein phosphorylation	Biological Process	GO:0001933	2.36E-20
transmembrane receptor protein serine/threonine kinase signaling pathway	Biological Process	GO:0007178	2.68E-20
morphogenesis of a branching structure	Biological Process	GO:0001763	2.86E-20
regulation of cysteine-type endopeptidase activity involved in apoptotic process	Biological Process	GO:0043281	3.04E-20
epithelial cell migration	Biological Process	GO:0010631	4.06E-20
epithelium migration	Biological Process	GO:0090132	6.25E-20
positive regulation of neuron death	Biological Process	GO:1901216	6.25E-20
morphogenesis of a branching epithelium	Biological Process	GO:0061138	6.91E-20
cellular response to transforming growth factor beta stimulus	Biological Process	GO:0071560	7.23E-20
regulation of cell growth	Biological Process	GO:0001558	9.00E-20
positive regulation of DNA-binding transcription factor activity	Biological Process	GO:0051091	9.92E-20
regulation of leukocyte differentiation	Biological Process	GO:1902105	1.11E-19
tissue migration	Biological Process	GO:0090130	1.40E-19
regulation of inflammatory response	Biological Process	GO:0050727	1.71E-19
ossification	Biological Process	GO:0001503	1.84E-19
stress-activated MAPK cascade	Biological Process	GO:0051403	1.93E-19
regulation of lymphocyte activation	Biological Process	GO:0051249	2.22E-19
tissue remodeling	Biological Process	GO:0048771	2.48E-19
striated muscle tissue development	Biological Process	GO:0014706	2.96E-19
mesenchyme development	Biological Process	GO:0060485	3.39E-19
cellular response to steroid hormone stimulus	Biological Process	GO:0071383	3.43E-19
regulation of cysteine-type endopeptidase activity	Biological Process	GO:2000116	4.17E-19
positive regulation of proteolysis	Biological Process	GO:0045862	4.26E-19
cellular response to antibiotic	Biological Process	GO:0071236	4.72E-19
axonogenesis	Biological Process	GO:0007409	5.38E-19
cell chemotaxis	Biological Process	GO:0060326	5.62E-19
stress-activated protein kinase signaling cascade	Biological Process	GO:0031098	5.62E-19
positive regulation of smooth muscle cell proliferation	Biological Process	GO:0048661	6.22E-19
regulation of gliogenesis	Biological Process	GO:0014013	6.76E-19
positive regulation of protein kinase B signaling	Biological Process	GO:0051897	8.78E-19
regulation of reactive oxygen species metabolic process	Biological Process	GO:2000377	9.74E-19

positive regulation of ERK1 and ERK2 cascade	Biological Process	GO:0070374	1.14E-18
aging	Biological Process	GO:0007568	1.33E-18
peptidyl-serine phosphorylation	Biological Process	GO:0018105	1.35E-18
cellular response to biotic stimulus	Biological Process	GO:0071216	1.38E-18
activation of protein kinase activity	Biological Process	GO:0032147	1.45E-18
embryonic organ development	Biological Process	GO:0048568	1.54E-18
leukocyte apoptotic process	Biological Process	GO:0071887	1.54E-18
negative regulation of extrinsic apoptotic signaling pathway	Biological Process	GO:2001237	1.54E-18
regeneration	Biological Process	GO:0031099	1.69E-18
heart morphogenesis	Biological Process	GO:0003007	2.02E-18
transforming growth factor beta receptor signaling pathway	Biological Process	GO:0007179	2.04E-18
autophagy	Biological Process	GO:0006914	2.72E-18
process utilizing autophagic mechanism	Biological Process	GO:0061919	2.72E-18
regulation of autophagy	Biological Process	GO:0010506	3.40E-18
response to ionizing radiation	Biological Process	GO:0010212	3.44E-18
positive regulation of T cell activation	Biological Process	GO:0050870	3.60E-18
Ras protein signal transduction	Biological Process	GO:0007265	3.60E-18
multicellular organismal homeostasis	Biological Process	GO:0048871	3.60E-18
regulation of peptidase activity	Biological Process	GO:0052547	5.70E-18
branching morphogenesis of an epithelial tube	Biological Process	GO:0048754	6.97E-18
peptidyl-serine modification	Biological Process	GO:0018209	7.04E-18
maintenance of cell number	Biological Process	GO:0098727	7.22E-18
positive regulation of cell activation	Biological Process	GO:0050867	8.67E-18
positive regulation of MAP kinase activity	Biological Process	GO:0043406	9.15E-18
positive regulation of neuron differentiation	Biological Process	GO:0045666	9.71E-18
regulation of binding	Biological Process	GO:0051098	1.25E-17
production of miRNAs involved in gene silencing by miRNA	Biological Process	GO:0035196	1.45E-17
regulation of neuron apoptotic process	Biological Process	GO:0043523	1.58E-17
mesenchymal cell differentiation	Biological Process	GO:0048762	1.58E-17
regulation of fibroblast proliferation	Biological Process	GO:0048145	1.85E-17
positive regulation of cell cycle	Biological Process	GO:0045787	2.03E-17
cardiac muscle tissue development	Biological Process	GO:0048738	2.58E-17
fibroblast proliferation	Biological Process	GO:0048144	2.62E-17
regulation of ossification	Biological Process	GO:0030278	2.65E-17
cell cycle G1/S phase transition	Biological Process	GO:0044843	2.72E-17
cellular response to peptide hormone stimulus	Biological Process	GO:0071375	2.84E-17

cellular response to oxygen levels	Biological Process	GO:0071453	2.97E-17
positive regulation of hemopoiesis	Biological Process	GO:1903708	3.39E-17
G1/S transition of mitotic cell cycle	Biological Process	GO:0000082	4.30E-17
epithelial to mesenchymal transition	Biological Process	GO:0001837	4.71E-17
cell aging	Biological Process	GO:0007569	4.71E-17
cellular response to reactive oxygen species	Biological Process	GO:0034614	4.80E-17
cell cycle arrest	Biological Process	GO:0007050	4.80E-17
regulation of epithelial cell migration	Biological Process	GO:0010632	4.80E-17
sex differentiation	Biological Process	GO:0007548	5.61E-17
negative regulation of mitotic cell cycle	Biological Process	GO:0045930	5.68E-17
regulation of endopeptidase activity	Biological Process	GO:0052548	7.27E-17
mammary gland development	Biological Process	GO:0030879	7.31E-17
response to nutrient	Biological Process	GO:0007584	7.37E-17
regulation of myeloid cell differentiation	Biological Process	GO:0045637	8.24E-17
response to carbohydrate	Biological Process	GO:0009743	8.27E-17
reactive oxygen species metabolic process	Biological Process	GO:0072593	8.54E-17
endothelial cell proliferation	Biological Process	GO:0001935	1.03E-16
positive regulation of epithelial to mesenchymal transition	Biological Process	GO:0010718	1.18E-16
positive regulation of leukocyte activation	Biological Process	GO:0002696	1.20E-16
cellular response to molecule of bacterial origin	Biological Process	GO:0071219	1.25E-16
response to hexose	Biological Process	GO:0009746	1.26E-16
gland morphogenesis	Biological Process	GO:0022612	1.29E-16
positive regulation of endothelial cell proliferation	Biological Process	GO:0001938	1.32E-16
response to alcohol	Biological Process	GO:0097305	1.32E-16
regulation of peptidyl-tyrosine phosphorylation	Biological Process	GO:0050730	1.74E-16
kidney development	Biological Process	GO:0001822	1.74E-16
intracellular receptor signaling pathway	Biological Process	GO:0030522	1.74E-16
regulation of protein secretion	Biological Process	GO:0050708	1.74E-16
organ growth	Biological Process	GO:0035265	1.75E-16
dsRNA processing	Biological Process	GO:0031050	1.86E-16
production of small RNA involved in gene silencing by RNA	Biological Process	GO:0070918	1.86E-16
negative regulation of immune system process	Biological Process	GO:0002683	2.05E-16
cellular response to lipopolysaccharide	Biological Process	GO:0071222	2.06E-16
stem cell population maintenance	Biological Process	GO:0019827	2.08E-16
regulation of endothelial cell proliferation	Biological Process	GO:0001936	2.20E-16
positive regulation of growth	Biological Process	GO:0045927	2.65E-16

renal system development	Biological Process	GO:0072001	2.88E-16
response to monosaccharide	Biological Process	GO:0034284	2.89E-16
heart valve morphogenesis	Biological Process	GO:0003179	2.97E-16
response to insulin	Biological Process	GO:0032868	3.52E-16
cellular response to acid chemical	Biological Process	GO:0071229	4.09E-16
pri-miRNA transcription by RNA polymerase II	Biological Process	GO:0061614	4.09E-16
negative regulation of transferase activity	Biological Process	GO:0051348	4.53E-16
endothelial cell migration	Biological Process	GO:0043542	4.66E-16
negative regulation of cell development	Biological Process	GO:0010721	5.01E-16
positive regulation of epithelial cell migration	Biological Process	GO:0010634	5.27E-16
forebrain development	Biological Process	GO:0030900	5.28E-16
positive regulation of cell cycle process	Biological Process	GO:0090068	5.58E-16
steroid hormone mediated signaling pathway	Biological Process	GO:0043401	6.81E-16
regulation of peptide secretion	Biological Process	GO:0002791	7.34E-16
negative regulation of cell adhesion	Biological Process	GO:0007162	7.78E-16
response to ketone	Biological Process	GO:1901654	8.30E-16
muscle cell differentiation	Biological Process	GO:0042692	8.30E-16
in utero embryonic development	Biological Process	GO:0001701	8.64E-16
heart valve development	Biological Process	GO:0003170	1.09E-15
gastrulation	Biological Process	GO:0007369	1.18E-15
chemokine production	Biological Process	GO:0032602	1.22E-15
response to glucose	Biological Process	GO:0009749	1.70E-15
pattern specification process	Biological Process	GO:0007389	2.49E-15
positive regulation of lymphocyte activation	Biological Process	GO:0051251	2.83E-15
positive regulation of fibroblast proliferation	Biological Process	GO:0048146	2.92E-15
hormone-mediated signaling pathway	Biological Process	GO:0009755	3.67E-15
positive regulation of inflammatory response	Biological Process	GO:0050729	4.18E-15
positive regulation of peptidyl-tyrosine phosphorylation	Biological Process	GO:0050731	4.19E-15
alpha-beta T cell differentiation	Biological Process	GO:0046632	4.29E-15
eye development	Biological Process	GO:0001654	4.30E-15
JNK cascade	Biological Process	GO:0007254	5.24E-15
T cell proliferation	Biological Process	GO:0042098	6.02E-15
regulation of muscle tissue development	Biological Process	GO:1901861	6.21E-15
response to hydrogen peroxide	Biological Process	GO:0042542	6.63E-15
regulation of stress-activated MAPK cascade	Biological Process	GO:0032872	6.65E-15
visual system development	Biological Process	GO:0150063	6.75E-15

epithelial cell apoptotic process	Biological Process	GO:1904019	6.84E-15
regulation of muscle organ development	Biological Process	GO:0048634	7.47E-15
myeloid cell homeostasis	Biological Process	GO:0002262	8.05E-15
gonad development	Biological Process	GO:0008406	8.26E-15
regulation of chemotaxis	Biological Process	GO:0050920	8.26E-15
alpha-beta T cell activation	Biological Process	GO:0046631	8.26E-15
regulation of stress-activated protein kinase signaling cascade	Biological Process	GO:0070302	8.77E-15
positive regulation of peptidase activity	Biological Process	GO:0010952	9.55E-15
formation of primary germ layer	Biological Process	GO:0001704	9.68E-15
negative regulation of neuron death	Biological Process	GO:1901215	1.05E-14
sensory system development	Biological Process	GO:0048880	1.16E-14
positive regulation of defense response	Biological Process	GO:0031349	1.20E-14
regulation of epithelial to mesenchymal transition	Biological Process	GO:0010717	1.57E-14
positive regulation of protein transport	Biological Process	GO:0051222	1.87E-14
cytokine metabolic process	Biological Process	GO:0042107	1.95E-14
phosphatidylinositol-mediated signaling	Biological Process	GO:0048015	2.03E-14
development of primary sexual characteristics	Biological Process	GO:0045137	2.09E-14
positive regulation of secretion	Biological Process	GO:0051047	2.09E-14
regulation of striated muscle tissue development	Biological Process	GO:0016202	2.16E-14
positive regulation of secretion by cell	Biological Process	GO:1903532	2.31E-14
negative regulation of kinase activity	Biological Process	GO:0033673	2.31E-14
positive regulation of establishment of protein localization	Biological Process	GO:1904951	2.39E-14
leukocyte chemotaxis	Biological Process	GO:0030595	2.39E-14
developmental growth involved in morphogenesis	Biological Process	GO:0060560	2.40E-14
Notch signaling pathway	Biological Process	GO:0007219	2.69E-14
regulation of cardiac muscle tissue development	Biological Process	GO:0055024	2.69E-14
cell-substrate adhesion	Biological Process	GO:0031589	2.78E-14
temperature homeostasis	Biological Process	GO:0001659	2.81E-14
positive regulation of NIK/NF-kappaB signaling	Biological Process	GO:1901224	2.84E-14
liver development	Biological Process	GO:0001889	2.91E-14
positive regulation of chemotaxis	Biological Process	GO:0050921	2.91E-14
inositol lipid-mediated signaling	Biological Process	GO:0048017	3.28E-14
connective tissue development	Biological Process	GO:0061448	4.19E-14
cellular response to decreased oxygen levels	Biological Process	GO:0036294	4.19E-14
regulation of leukocyte migration	Biological Process	GO:0002685	4.35E-14
regulation of cyclin-dependent protein serine/threonine kinase activity	Biological Process	GO:0000079	4.45E-14

regulation of epithelial cell differentiation	Biological Process	GO:0030856	4.52E-14
regulation of lipid metabolic process	Biological Process	GO:0019216	4.60E-14
epithelial cell development	Biological Process	GO:0002064	4.62E-14
hepaticobiliary system development	Biological Process	GO:0061008	5.43E-14
response to metal ion	Biological Process	GO:0010038	6.66E-14
regulation of DNA metabolic process	Biological Process	GO:0051052	8.00E-14
positive regulation of cell growth	Biological Process	GO:0030307	8.12E-14
developmental cell growth	Biological Process	GO:0048588	1.02E-13
regulation of cellular response to growth factor stimulus	Biological Process	GO:0090287	1.05E-13
regulation of cytokine biosynthetic process	Biological Process	GO:0042035	1.05E-13
cellular response to radiation	Biological Process	GO:0071478	1.05E-13
cytokine biosynthetic process	Biological Process	GO:0042089	1.08E-13
regulation of muscle cell differentiation	Biological Process	GO:0051147	1.11E-13
negative regulation of protein kinase activity	Biological Process	GO:0006469	1.16E-13
regulation of cyclin-dependent protein kinase activity	Biological Process	GO:1904029	1.20E-13
regulation of chemokine production	Biological Process	GO:0032642	1.28E-13
osteoblast differentiation	Biological Process	GO:0001649	1.36E-13
cell-matrix adhesion	Biological Process	GO:0007160	1.36E-13
camera-type eye development	Biological Process	GO:0043010	1.43E-13
neuroinflammatory response	Biological Process	GO:0150076	1.50E-13
response to estradiol	Biological Process	GO:0032355	1.59E-13
negative regulation of growth	Biological Process	GO:0045926	1.67E-13
regulation of leukocyte apoptotic process	Biological Process	GO:2000106	1.70E-13
negative regulation of cellular component movement	Biological Process	GO:0051271	1.72E-13
positive regulation of leukocyte differentiation	Biological Process	GO:1902107	1.77E-13
cellular response to insulin stimulus	Biological Process	GO:0032869	1.79E-13
regulation of G1/S transition of mitotic cell cycle	Biological Process	GO:2000045	1.79E-13
negative regulation of cell migration	Biological Process	GO:0030336	1.89E-13
regulation of myeloid leukocyte differentiation	Biological Process	GO:0002761	2.00E-13
regulation of NIK/NF-kappaB signaling	Biological Process	GO:1901222	2.00E-13
negative regulation of cell cycle process	Biological Process	GO:0010948	2.28E-13
regulation of animal organ morphogenesis	Biological Process	GO:2000027	2.82E-13
regulation of Wnt signaling pathway	Biological Process	GO:0030111	2.82E-13
regulation of mononuclear cell proliferation	Biological Process	GO:0032944	3.21E-13
regulation of neuron projection development	Biological Process	GO:0010975	3.29E-13
transcription initiation from RNA polymerase II promoter	Biological Process	GO:0006367	3.42E-13

positive regulation of reactive oxygen species metabolic process	Biological Process	GO:2000379	3.47E-13
phosphatidylinositol 3-kinase signaling	Biological Process	GO:0014065	3.76E-13
regulation of leukocyte proliferation	Biological Process	GO:0070663	4.28E-13
positive regulation of binding	Biological Process	GO:0051099	4.33E-13
positive regulation of neuron apoptotic process	Biological Process	GO:0043525	4.34E-13
positive regulation of NF-kappaB transcription factor activity	Biological Process	GO:0051092	4.52E-13
negative regulation of locomotion	Biological Process	GO:0040013	4.71E-13
cellular response to mechanical stimulus	Biological Process	GO:0071260	4.87E-13
fat cell differentiation	Biological Process	GO:0045444	4.87E-13
positive regulation of phosphatidylinositol 3-kinase signaling	Biological Process	GO:0014068	5.04E-13
kidney epithelium development	Biological Process	GO:0072073	5.08E-13
protein autophosphorylation	Biological Process	GO:0046777	5.36E-13
intrinsic apoptotic signaling pathway in response to DNA damage	Biological Process	GO:0008630	5.52E-13
regulation of cell cycle G1/S phase transition	Biological Process	GO:1902806	5.60E-13
positive regulation of cell projection organization	Biological Process	GO:0031346	5.67E-13
muscle organ development	Biological Process	GO:0007517	5.96E-13
regulation of cellular response to oxidative stress	Biological Process	GO:1900407	6.52E-13
positive regulation of cysteine-type endopeptidase activity involved in apoptotic process	Biological Process	GO:0043280	6.75E-13
negative regulation of cytokine production	Biological Process	GO:0001818	6.82E-13
lung development	Biological Process	GO:0030324	7.35E-13
nephron development	Biological Process	GO:0072006	7.35E-13
placenta development	Biological Process	GO:0001890	7.63E-13
osteoclast differentiation	Biological Process	GO:0030316	7.83E-13
astrocyte differentiation	Biological Process	GO:0048708	8.41E-13
regulation of phosphatidylinositol 3-kinase signaling	Biological Process	GO:0014066	8.76E-13
negative regulation of cell motility	Biological Process	GO:2000146	9.31E-13
positive regulation of gliogenesis	Biological Process	GO:0014015	1.04E-12
negative regulation of G1/S transition of mitotic cell cycle	Biological Process	GO:2000134	1.08E-12
regulation of endothelial cell migration	Biological Process	GO:0010594	1.10E-12
cellular response to hypoxia	Biological Process	GO:0071456	1.16E-12
regulation of lymphocyte proliferation	Biological Process	GO:0050670	1.35E-12
regulation of pri-miRNA transcription by RNA polymerase II	Biological Process	GO:1902893	1.40E-12
respiratory tube development	Biological Process	GO:0030323	1.41E-12
regulation of protein localization to membrane	Biological Process	GO:1905475	1.50E-12
digestive system development	Biological Process	GO:0055123	1.53E-12

response to amyloid-beta	Biological Process	GO:1904645	1.66E-12
positive regulation of endopeptidase activity	Biological Process	GO:0010950	1.95E-12
I-kappaB kinase/NF-kappaB signaling	Biological Process	GO:0007249	2.07E-12
positive regulation of apoptotic signaling pathway	Biological Process	GO:2001235	2.28E-12
cell death in response to oxidative stress	Biological Process	GO:0036473	2.33E-12
rhythmic process	Biological Process	GO:0048511	2.51E-12
cellular response to toxic substance	Biological Process	GO:0097237	2.57E-12
positive regulation of cysteine-type endopeptidase activity	Biological Process	GO:2001056	2.64E-12
blood vessel endothelial cell migration	Biological Process	GO:0043534	2.65E-12
canonical Wnt signaling pathway	Biological Process	GO:0060070	3.18E-12
regulation of cell morphogenesis	Biological Process	GO:0022604	3.22E-12
semi-lunar valve development	Biological Process	GO:1905314	3.26E-12
DNA-templated transcription, initiation	Biological Process	GO:0006352	3.30E-12
negative regulation of cell cycle G1/S phase transition	Biological Process	GO:1902807	3.53E-12
regulation of cell-substrate adhesion	Biological Process	GO:0010810	3.59E-12
positive regulation of small molecule metabolic process	Biological Process	GO:0062013	3.70E-12
regulation of mitotic cell cycle phase transition	Biological Process	GO:1901990	3.80E-12
aortic valve development	Biological Process	GO:0003176	4.20E-12
endothelium development	Biological Process	GO:0003158	4.25E-12
regionalization	Biological Process	GO:0003002	4.29E-12
glial cell proliferation	Biological Process	GO:0014009	4.45E-12
vascular endothelial growth factor receptor signaling pathway	Biological Process	GO:0048010	4.74E-12
positive regulation of developmental growth	Biological Process	GO:0048639	4.86E-12
regulation of leukocyte chemotaxis	Biological Process	GO:0002688	4.97E-12
cardiac muscle tissue growth	Biological Process	GO:0055017	4.99E-12
response to light stimulus	Biological Process	GO:0009416	5.03E-12
macrophage differentiation	Biological Process	GO:0030225	5.17E-12
regulation of transmembrane receptor protein serine/threonine kinase signaling pathway	Biological Process	GO:0090092	5.22E-12
mammary gland epithelium development	Biological Process	GO:0061180	5.29E-12
cell-cell junction organization	Biological Process	GO:0045216	5.58E-12
regulation of JNK cascade	Biological Process	GO:0046328	5.58E-12
regulation of response to oxidative stress	Biological Process	GO:1902882	5.90E-12
digestive tract development	Biological Process	GO:0048565	6.06E-12
regulation of chromosome organization	Biological Process	GO:0033044	6.34E-12
animal organ regeneration	Biological Process	GO:0031100	7.04E-12

positive regulation of animal organ morphogenesis	Biological Process	GO:0110110	7.14E-12
negative regulation of protein serine/threonine kinase activity	Biological Process	GO:0071901	7.41E-12
respiratory system development	Biological Process	GO:0060541	7.48E-12
aortic valve morphogenesis	Biological Process	GO:0003180	8.05E-12
anatomical structure homeostasis	Biological Process	GO:0060249	8.05E-12
regulation of heart morphogenesis	Biological Process	GO:2000826	8.18E-12
myeloid leukocyte migration	Biological Process	GO:0097529	8.30E-12
positive regulation of ossification	Biological Process	GO:0045778	8.45E-12
regulation of blood vessel endothelial cell migration	Biological Process	GO:0043535	8.45E-12
stem cell differentiation	Biological Process	GO:0048863	8.50E-12
negative regulation of striated muscle tissue development	Biological Process	GO:0045843	9.24E-12
cardiocyte differentiation	Biological Process	GO:0035051	9.25E-12
negative regulation of neurogenesis	Biological Process	GO:0050768	9.65E-12
positive regulation of myeloid cell differentiation	Biological Process	GO:0045639	1.07E-11
regulation of cell division	Biological Process	GO:0051302	1.08E-11
positive regulation of leukocyte migration	Biological Process	GO:0002687	1.20E-11
positive regulation of endothelial cell migration	Biological Process	GO:0010595	1.20E-11
regulation of oxidative stress-induced cell death	Biological Process	GO:1903201	1.21E-11
negative regulation of muscle organ development	Biological Process	GO:0048635	1.27E-11
interaction with host	Biological Process	GO:0051701	1.29E-11
regulation of cell-matrix adhesion	Biological Process	GO:0001952	1.33E-11
CD4-positive, alpha-beta T cell activation	Biological Process	GO:0035710	1.35E-11
positive regulation of cellular protein localization	Biological Process	GO:1903829	1.39E-11
covalent chromatin modification	Biological Process	GO:0016569	1.39E-11
myoblast differentiation	Biological Process	GO:0045445	1.51E-11
artery morphogenesis	Biological Process	GO:0048844	1.57E-11
positive regulation of autophagy	Biological Process	GO:0010508	1.60E-11
stem cell proliferation	Biological Process	GO:0072089	1.60E-11
cellular response to ketone	Biological Process	GO:1901655	1.70E-11
cardiac chamber development	Biological Process	GO:0003205	1.70E-11
axon guidance	Biological Process	GO:0007411	1.85E-11
lymphocyte apoptotic process	Biological Process	GO:0070227	2.00E-11
mesoderm morphogenesis	Biological Process	GO:0048332	2.05E-11
neuron projection guidance	Biological Process	GO:0097485	2.06E-11
response to UV	Biological Process	GO:0009411	2.07E-11
regulation of protein binding	Biological Process	GO:0043393	2.07E-11

columnar/cuboidal epithelial cell differentiation	Biological Process	GO:0002065	2.09E-11
heart growth	Biological Process	GO:0060419	2.09E-11
extracellular matrix organization	Biological Process	GO:0030198	2.12E-11
response to corticosteroid	Biological Process	GO:0031960	2.17E-11
glucose homeostasis	Biological Process	GO:0042593	2.17E-11
regulation of cell cycle phase transition	Biological Process	GO:1901987	2.22E-11
negative regulation of nervous system development	Biological Process	GO:0051961	2.26E-11
extracellular structure organization	Biological Process	GO:0043062	2.31E-11
negative regulation of muscle tissue development	Biological Process	GO:1901862	2.32E-11
extrinsic apoptotic signaling pathway via death domain receptors	Biological Process	GO:0008625	2.41E-11
carbohydrate homeostasis	Biological Process	GO:0033500	2.44E-11
positive regulation of mitotic cell cycle	Biological Process	GO:0045931	2.51E-11
response to amino acid	Biological Process	GO:0043200	2.52E-11
regulation of organ growth	Biological Process	GO:0046620	2.52E-11
cellular senescence	Biological Process	GO:0090398	2.58E-11
histone modification	Biological Process	GO:0016570	2.70E-11
striated muscle cell differentiation	Biological Process	GO:0051146	2.93E-11
cellular response to extracellular stimulus	Biological Process	GO:0031668	2.93E-11
positive regulation of protein secretion	Biological Process	GO:0050714	2.93E-11
female gonad development	Biological Process	GO:0008585	3.26E-11
response to gamma radiation	Biological Process	GO:0010332	3.48E-11
female sex differentiation	Biological Process	GO:0046660	3.75E-11
positive regulation of osteoblast differentiation	Biological Process	GO:0045669	4.24E-11
cellular response to amyloid-beta	Biological Process	GO:1904646	4.33E-11
signal transduction in absence of ligand	Biological Process	GO:0038034	4.48E-11
extrinsic apoptotic signaling pathway in absence of ligand	Biological Process	GO:0097192	4.48E-11
glial cell development	Biological Process	GO:0021782	4.55E-11
regulation of osteoblast differentiation	Biological Process	GO:0045667	4.83E-11
mesonephric epithelium development	Biological Process	GO:0072163	5.06E-11
mesonephric tubule development	Biological Process	GO:0072164	5.06E-11
appendage development	Biological Process	GO:0048736	5.44E-11
limb development	Biological Process	GO:0060173	5.44E-11
telencephalon development	Biological Process	GO:0021537	5.56E-11
endocrine system development	Biological Process	GO:0035270	5.77E-11
cellular response to hydrogen peroxide	Biological Process	GO:0070301	6.27E-11
modulation of chemical synaptic transmission	Biological Process	GO:0050804	6.34E-11

endocardial cushion development	Biological Process	GO:0003197	6.34E-11
astrocyte development	Biological Process	GO:0014002	6.34E-11
glial cell activation	Biological Process	GO:0061900	6.57E-11
positive regulation of peptide secretion	Biological Process	GO:0002793	6.60E-11
regulation of trans-synaptic signaling	Biological Process	GO:0099177	6.85E-11
cell junction assembly	Biological Process	GO:0034329	6.93E-11
receptor signaling pathway via JAK-STAT	Biological Process	GO:0007259	6.93E-11
receptor metabolic process	Biological Process	GO:0043112	6.93E-11
CD4-positive, alpha-beta T cell differentiation	Biological Process	GO:0043367	7.51E-11
development of primary female sexual characteristics	Biological Process	GO:0046545	9.53E-11
regulation of reactive oxygen species biosynthetic process	Biological Process	GO:1903426	9.53E-11
mesoderm formation	Biological Process	GO:0001707	9.80E-11
response to xenobiotic stimulus	Biological Process	GO:0009410	1.01E-10
regulation of small molecule metabolic process	Biological Process	GO:0062012	1.15E-10
mesonephros development	Biological Process	GO:0001823	1.17E-10
positive regulation of neuron projection development	Biological Process	GO:0010976	1.22E-10
activation of MAPK activity	Biological Process	GO:0000187	1.23E-10
learning or memory	Biological Process	GO:0007611	1.23E-10
regulation of actin filament-based process	Biological Process	GO:0032970	1.26E-10
endoderm development	Biological Process	GO:0007492	1.26E-10
positive regulation of chromosome organization	Biological Process	GO:2001252	1.29E-10
positive regulation of protein localization to membrane	Biological Process	GO:1905477	1.38E-10
regulation of vascular smooth muscle cell proliferation	Biological Process	GO:1904705	1.42E-10
vascular smooth muscle cell proliferation	Biological Process	GO:1990874	1.42E-10
cartilage development	Biological Process	GO:0051216	1.43E-10
cognition	Biological Process	GO:0050890	1.51E-10
cold-induced thermogenesis	Biological Process	GO:0106106	1.55E-10
regulation of cold-induced thermogenesis	Biological Process	GO:0120161	1.55E-10
positive regulation of gene silencing by miRNA	Biological Process	GO:2000637	1.58E-10
alpha-beta T cell activation involved in immune response	Biological Process	GO:0002287	1.61E-10
alpha-beta T cell differentiation involved in immune response	Biological Process	GO:0002293	1.61E-10
regulation of protein stability	Biological Process	GO:0031647	1.64E-10
regulation of muscle system process	Biological Process	GO:0090257	1.70E-10
regulation of intrinsic apoptotic signaling pathway	Biological Process	GO:2001242	1.70E-10
negative regulation of mitotic cell cycle phase transition	Biological Process	GO:1901991	2.00E-10
regulation of cardiac muscle tissue growth	Biological Process	GO:0055021	2.05E-10

embryonic placenta development	Biological Process	GO:0001892	2.23E-10
positive regulation of leukocyte chemotaxis	Biological Process	GO:0002690	2.23E-10
mesoderm development	Biological Process	GO:0007498	2.28E-10
cellular response to nutrient levels	Biological Process	GO:0031669	2.31E-10
regulation of hormone secretion	Biological Process	GO:0046883	2.36E-10
positive regulation of lipid metabolic process	Biological Process	GO:0045834	2.49E-10
regulation of response to wounding	Biological Process	GO:1903034	2.57E-10
positive regulation of protein catabolic process	Biological Process	GO:0045732	2.65E-10
prostate gland development	Biological Process	GO:0030850	2.69E-10
ureteric bud development	Biological Process	GO:0001657	2.75E-10
positive regulation of DNA metabolic process	Biological Process	GO:0051054	2.78E-10
cellular response to ionizing radiation	Biological Process	GO:0071479	2.84E-10
positive regulation of posttranscriptional gene silencing	Biological Process	GO:0060148	2.92E-10
negative regulation of cell-cell adhesion	Biological Process	GO:0022408	2.93E-10
receptor signaling pathway via STAT	Biological Process	GO:0097696	3.02E-10
contractile actin filament bundle assembly	Biological Process	GO:0030038	3.37E-10
stress fiber assembly	Biological Process	GO:0043149	3.37E-10
negative regulation of response to external stimulus	Biological Process	GO:0032102	3.40E-10
cellular response to alcohol	Biological Process	GO:0097306	3.44E-10
somatic stem cell population maintenance	Biological Process	GO:0035019	3.65E-10
male gonad development	Biological Process	GO:0008584	3.68E-10
tissue homeostasis	Biological Process	GO:0001894	3.72E-10
regulation of endocytosis	Biological Process	GO:0030100	3.72E-10
regulation of osteoclast differentiation	Biological Process	GO:0045670	3.72E-10
male sex differentiation	Biological Process	GO:0046661	4.03E-10
bone remodeling	Biological Process	GO:0046849	4.25E-10
mononuclear cell migration	Biological Process	GO:0071674	4.25E-10
regulation of T cell differentiation	Biological Process	GO:0045580	4.29E-10
development of primary male sexual characteristics	Biological Process	GO:0046546	4.29E-10
NIK/NF-kappaB signaling	Biological Process	GO:0038061	4.35E-10
nephron epithelium development	Biological Process	GO:0072009	4.49E-10
cardiac chamber morphogenesis	Biological Process	GO:0003206	4.55E-10
regulation of glial cell differentiation	Biological Process	GO:0045685	4.63E-10
regulation of protein modification by small protein conjugation or removal	Biological Process	GO:1903320	4.65E-10
anterior/posterior pattern specification	Biological Process	GO:0009952	4.74E-10
artery development	Biological Process	GO:0060840	4.96E-10

negative regulation of cardiac muscle tissue development	Biological Process	GO:0055026	5.11E-10
cellular response to UV	Biological Process	GO:0034644	5.12E-10
positive regulation of chemokine production	Biological Process	GO:0032722	6.15E-10
T-helper cell differentiation	Biological Process	GO:0042093	6.15E-10
regulation of mitotic nuclear division	Biological Process	GO:0007088	7.15E-10
regulation of protein catabolic process	Biological Process	GO:0042176	7.27E-10
second-messenger-mediated signaling	Biological Process	GO:0019932	7.66E-10
erythrocyte homeostasis	Biological Process	GO:0034101	7.84E-10
reactive oxygen species biosynthetic process	Biological Process	GO:1903409	7.84E-10
regulation of alpha-beta T cell activation	Biological Process	GO:0046634	7.97E-10
regulation of heart growth	Biological Process	GO:0060420	8.08E-10
negative regulation of epithelial cell proliferation	Biological Process	GO:0050680	8.18E-10
positive regulation of cytokine biosynthetic process	Biological Process	GO:0042108	8.23E-10
adaptive thermogenesis	Biological Process	GO:1990845	8.23E-10
maintenance of location	Biological Process	GO:0051235	9.33E-10
mesenchyme morphogenesis	Biological Process	GO:0072132	9.79E-10
T cell differentiation involved in immune response	Biological Process	GO:0002292	1.07E-09
cellular response to vascular endothelial growth factor stimulus	Biological Process	GO:0035924	1.07E-09
cellular response to amino acid stimulus	Biological Process	GO:0071230	1.07E-09
positive regulation of transmembrane receptor protein serine/threonine kinase signaling pathway	Biological Process	GO:0090100	1.08E-09
CD4-positive, alpha-beta T cell differentiation involved in immune response	Biological Process	GO:0002294	1.08E-09
regulation of cell aging	Biological Process	GO:0090342	1.08E-09
regulation of response to DNA damage stimulus	Biological Process	GO:2001020	1.10E-09
macrophage activation	Biological Process	GO:0042116	1.19E-09
response to fatty acid	Biological Process	GO:0070542	1.24E-09
multicellular organism growth	Biological Process	GO:0035264	1.26E-09
positive regulation of cytosolic calcium ion concentration	Biological Process	GO:0007204	1.34E-09
signal transduction by p53 class mediator	Biological Process	GO:0072331	1.49E-09
negative regulation of cell cycle phase transition	Biological Process	GO:1901988	1.49E-09
regulation of receptor signaling pathway via JAK-STAT	Biological Process	GO:0046425	1.66E-09
negative regulation of catabolic process	Biological Process	GO:0009895	1.70E-09
hormone secretion	Biological Process	GO:0046879	1.70E-09
positive regulation of cold-induced thermogenesis	Biological Process	GO:0120162	1.77E-09
positive regulation of blood vessel endothelial cell migration	Biological Process	GO:0043536	1.91E-09
positive regulation of carbohydrate metabolic process	Biological Process	GO:0045913	1.91E-09

response to virus	Biological Process	GO:0009615	1.94E-09
sprouting angiogenesis	Biological Process	GO:0002040	1.97E-09
negative regulation of transmembrane transport	Biological Process	GO:0034763	2.12E-09
negative regulation of MAPK cascade	Biological Process	GO:0043409	2.24E-09
regulation of peptidyl-serine phosphorylation	Biological Process	GO:0033135	2.25E-09
regulation of alpha-beta T cell differentiation	Biological Process	GO:0046637	2.44E-09
response to tumor necrosis factor	Biological Process	GO:0034612	2.46E-09
anoikis	Biological Process	GO:0043276	2.47E-09
positive regulation of production of miRNAs involved in gene silencing by miRNA	Biological Process	GO:1903800	2.61E-09
phagocytosis	Biological Process	GO:0006909	2.78E-09
microglial cell activation	Biological Process	GO:0001774	2.81E-09
leukocyte activation involved in inflammatory response	Biological Process	GO:0002269	2.81E-09
positive regulation of histone modification	Biological Process	GO:0031058	2.86E-09
muscle system process	Biological Process	GO:0003012	2.89E-09
intracellular steroid hormone receptor signaling pathway	Biological Process	GO:0030518	2.90E-09
regulation of transforming growth factor beta receptor signaling pathway	Biological Process	GO:0017015	3.12E-09
segmentation	Biological Process	GO:0035282	3.13E-09
vascular process in circulatory system	Biological Process	GO:0003018	3.13E-09
regulation of lipid kinase activity	Biological Process	GO:0043550	3.13E-09
regulation of actin cytoskeleton organization	Biological Process	GO:0032956	3.30E-09
positive regulation of mononuclear cell proliferation	Biological Process	GO:0032946	3.38E-09
regulation of I-kappaB kinase/NF-kappaB signaling	Biological Process	GO:0043122	3.58E-09
nitric oxide metabolic process	Biological Process	GO:0046209	3.65E-09
regulation of glial cell proliferation	Biological Process	GO:0060251	3.69E-09
lymphocyte migration	Biological Process	GO:0072676	3.79E-09
negative regulation of epithelial cell differentiation	Biological Process	GO:0030857	3.79E-09
positive regulation of vascular smooth muscle cell proliferation	Biological Process	GO:1904707	3.79E-09
hormone transport	Biological Process	GO:0009914	3.79E-09
mitotic cell cycle checkpoint	Biological Process	GO:0007093	3.79E-09
odontogenesis	Biological Process	GO:0042476	3.91E-09
regulation of fat cell differentiation	Biological Process	GO:0045598	3.91E-09
negative regulation of muscle cell differentiation	Biological Process	GO:0051148	3.98E-09
pathway-restricted SMAD protein phosphorylation	Biological Process	GO:0060389	3.98E-09
regulation of cellular response to transforming growth factor beta stimulus	Biological Process	GO:1903844	4.26E-09
regulation of tyrosine phosphorylation of STAT protein	Biological Process	GO:0042509	4.46E-09
cardiac septum development	Biological Process	GO:0003279	4.46E-09

response to alkaloid	Biological Process	GO:0043279	4.46E-09
response to vitamin	Biological Process	GO:0033273	5.09E-09
nephron tubule development	Biological Process	GO:0072080	5.09E-09
regulation of nitric oxide biosynthetic process	Biological Process	GO:0045428	5.10E-09
response to interleukin-6	Biological Process	GO:0070741	5.10E-09
regulation of cell size	Biological Process	GO:0008361	5.13E-09
positive regulation of chromatin organization	Biological Process	GO:1905269	5.34E-09
lipopolysaccharide-mediated signaling pathway	Biological Process	GO:0031663	5.39E-09
regulation of extrinsic apoptotic signaling pathway via death domain receptors	Biological Process	GO:1902041	5.39E-09
B cell apoptotic process	Biological Process	GO:0001783	5.48E-09
neuron projection extension	Biological Process	GO:1990138	5.58E-09
female pregnancy	Biological Process	GO:0007565	5.84E-09
response to glucocorticoid	Biological Process	GO:0051384	6.13E-09
regulation of receptor signaling pathway via STAT	Biological Process	GO:1904892	6.13E-09
erythrocyte differentiation	Biological Process	GO:0030218	6.21E-09
endothelial cell differentiation	Biological Process	GO:0045446	6.21E-09
regulation of lymphocyte differentiation	Biological Process	GO:0045619	6.33E-09
reactive nitrogen species metabolic process	Biological Process	GO:2001057	6.69E-09
embryonic limb morphogenesis	Biological Process	GO:0030326	6.79E-09
embryonic appendage morphogenesis	Biological Process	GO:0035113	6.79E-09
renal tubule development	Biological Process	GO:0061326	7.38E-09
ear development	Biological Process	GO:0043583	7.65E-09
response to fluid shear stress	Biological Process	GO:0034405	7.78E-09
regulation of endothelial cell differentiation	Biological Process	GO:0045601	7.82E-09
anatomical structure maturation	Biological Process	GO:0071695	7.91E-09
leukocyte homeostasis	Biological Process	GO:0001776	8.13E-09
tyrosine phosphorylation of STAT protein	Biological Process	GO:0007260	8.13E-09
negative regulation of cell-substrate adhesion	Biological Process	GO:0010812	8.13E-09
nitric oxide biosynthetic process	Biological Process	GO:0006809	8.48E-09
pre-miRNA processing	Biological Process	GO:0031054	8.94E-09
T cell activation involved in immune response	Biological Process	GO:0002286	8.95E-09
regulation of cellular senescence	Biological Process	GO:2000772	9.02E-09
regulation of canonical Wnt signaling pathway	Biological Process	GO:0060828	9.02E-09
positive regulation of pri-miRNA transcription by RNA polymerase II	Biological Process	GO:1902895	9.34E-09
regulation of cytosolic calcium ion concentration	Biological Process	GO:0051480	1.02E-08
nephron morphogenesis	Biological Process	GO:0072028	1.05E-08

multi-multicellular organism process	Biological Process	GO:0044706	1.05E-08
embryonic organ morphogenesis	Biological Process	GO:0048562	1.08E-08
positive regulation of leukocyte proliferation	Biological Process	GO:0070665	1.09E-08
regulation of cytokine secretion	Biological Process	GO:0050707	1.11E-08
regulation of CD4-positive, alpha-beta T cell activation	Biological Process	GO:2000514	1.16E-08
regulation of myoblast differentiation	Biological Process	GO:0045661	1.19E-08
regulation of lymphocyte apoptotic process	Biological Process	GO:0070228	1.19E-08
regulation of tissue remodeling	Biological Process	GO:0034103	1.20E-08
cell-cell junction assembly	Biological Process	GO:0007043	1.24E-08
negative regulation of leukocyte cell-cell adhesion	Biological Process	GO:1903038	1.24E-08
T cell differentiation in thymus	Biological Process	GO:0033077	1.28E-08
SMAD protein signal transduction	Biological Process	GO:0060395	1.28E-08
actomyosin structure organization	Biological Process	GO:0031032	1.30E-08
negative regulation of vasculature development	Biological Process	GO:1901343	1.33E-08
hindbrain development	Biological Process	GO:0030902	1.38E-08
cardiac epithelial to mesenchymal transition	Biological Process	GO:0060317	1.40E-08
insulin receptor signaling pathway	Biological Process	GO:0008286	1.43E-08
positive regulation of lymphocyte proliferation	Biological Process	GO:0050671	1.43E-08
glandular epithelial cell differentiation	Biological Process	GO:0002067	1.44E-08
cellular response to interleukin-6	Biological Process	GO:0071354	1.44E-08
positive regulation of receptor signaling pathway via JAK-STAT	Biological Process	GO:0046427	1.44E-08
regulation of oxidative stress-induced neuron death	Biological Process	GO:1903203	1.46E-08
regulation of nuclear division	Biological Process	GO:0051783	1.48E-08
cell fate specification	Biological Process	GO:0001708	1.49E-08
positive regulation of myeloid leukocyte differentiation	Biological Process	GO:0002763	1.54E-08
negative regulation of extrinsic apoptotic signaling pathway via death domain receptors	Biological Process	GO:1902042	1.55E-08
regulation of ion transmembrane transport	Biological Process	GO:0034765	1.65E-08
cytokine secretion	Biological Process	GO:0050663	1.76E-08
positive regulation of cell morphogenesis involved in differentiation	Biological Process	GO:0010770	1.79E-08
blood coagulation	Biological Process	GO:0007596	1.83E-08
regulation of histone modification	Biological Process	GO:0031056	1.88E-08
cellular response to inorganic substance	Biological Process	GO:0071241	1.89E-08
regulation of calcium ion transport	Biological Process	GO:0051924	1.91E-08
striated muscle cell proliferation	Biological Process	GO:0014855	1.91E-08
cellular response to platelet-derived growth factor stimulus	Biological Process	GO:0036120	1.93E-08
ovarian follicle development	Biological Process	GO:0001541	2.01E-08

negative regulation of hemopoiesis	Biological Process	GO:1903707	2.03E-08
calcium ion homeostasis	Biological Process	GO:0055074	2.05E-08
positive regulation of mitotic cell cycle phase transition	Biological Process	GO:1901992	2.08E-08
cell cycle checkpoint	Biological Process	GO:0000075	2.08E-08
interleukin-1 beta production	Biological Process	GO:0032611	2.11E-08
signal transduction in response to DNA damage	Biological Process	GO:0042770	2.19E-08
sensory organ morphogenesis	Biological Process	GO:0090596	2.29E-08
neuron death in response to oxidative stress	Biological Process	GO:0036475	2.29E-08
regulation of T cell proliferation	Biological Process	GO:0042129	2.30E-08
tumor necrosis factor superfamily cytokine production	Biological Process	GO:0071706	2.38E-08
regulation of signal transduction by p53 class mediator	Biological Process	GO:1901796	2.38E-08
regulation of morphogenesis of an epithelium	Biological Process	GO:1905330	2.38E-08
cellular calcium ion homeostasis	Biological Process	GO:0006874	2.38E-08
positive regulation of receptor signaling pathway via STAT	Biological Process	GO:1904894	2.49E-08
cytokine production involved in immune response	Biological Process	GO:0002367	2.49E-08
calcium-mediated signaling	Biological Process	GO:0019722	2.55E-08
T cell costimulation	Biological Process	GO:0031295	2.58E-08
lymphocyte activation involved in immune response	Biological Process	GO:0002285	2.67E-08
pallium development	Biological Process	GO:0021543	2.67E-08
hemostasis	Biological Process	GO:0007599	2.69E-08
coagulation	Biological Process	GO:0050817	2.92E-08
negative regulation of ion transmembrane transport	Biological Process	GO:0034766	2.94E-08
negative regulation of leukocyte differentiation	Biological Process	GO:1902106	2.94E-08
regulation of interleukin-17 production	Biological Process	GO:0032660	2.95E-08
regulation of neuroinflammatory response	Biological Process	GO:0150077	2.96E-08
viral life cycle	Biological Process	GO:0019058	2.97E-08
negative regulation of neuron apoptotic process	Biological Process	GO:0043524	3.17E-08
response to platelet-derived growth factor	Biological Process	GO:0036119	3.27E-08
regulation of cell morphogenesis involved in differentiation	Biological Process	GO:0010769	3.27E-08
lymphocyte costimulation	Biological Process	GO:0031294	3.31E-08
positive regulation of I-kappaB kinase/NF-kappaB signaling	Biological Process	GO:0043123	3.34E-08
circadian rhythm	Biological Process	GO:0007623	3.38E-08
regulation of stress fiber assembly	Biological Process	GO:0051492	3.38E-08
negative regulation of cell aging	Biological Process	GO:0090344	3.51E-08
positive regulation of interleukin-17 production	Biological Process	GO:0032740	3.58E-08
negative regulation of anoikis	Biological Process	GO:2000811	3.58E-08

appendage morphogenesis	Biological Process	GO:0035107	3.59E-08
limb morphogenesis	Biological Process	GO:0035108	3.59E-08
interleukin-1 production	Biological Process	GO:0032612	3.73E-08
regulation of protein localization to cell periphery	Biological Process	GO:1904375	3.73E-08
peptidyl-threonine phosphorylation	Biological Process	GO:0018107	3.83E-08
regulation of type I interferon production	Biological Process	GO:0032479	3.83E-08
regulation of Notch signaling pathway	Biological Process	GO:0008593	4.04E-08
positive regulation of peptidyl-serine phosphorylation	Biological Process	GO:0033138	4.04E-08
cardiac cell development	Biological Process	GO:0055006	4.17E-08
regulation of actomyosin structure organization	Biological Process	GO:0110020	4.17E-08
endocardial cushion morphogenesis	Biological Process	GO:0003203	4.19E-08
response to ischemia	Biological Process	GO:0002931	4.41E-08
cardiac septum morphogenesis	Biological Process	GO:0060411	4.45E-08
cardiac muscle cell proliferation	Biological Process	GO:0060038	4.55E-08
neural precursor cell proliferation	Biological Process	GO:0061351	4.64E-08
negative regulation of cell growth	Biological Process	GO:0030308	4.65E-08
mitotic nuclear division	Biological Process	GO:0140014	4.69E-08
positive regulation of cell cycle phase transition	Biological Process	GO:1901989	4.73E-08
ephrin receptor signaling pathway	Biological Process	GO:0048013	4.87E-08
regulation of carbohydrate metabolic process	Biological Process	GO:0006109	4.90E-08
response to interferon-gamma	Biological Process	GO:0034341	4.90E-08
negative regulation of cell activation	Biological Process	GO:0050866	4.90E-08
regulation of Ras protein signal transduction	Biological Process	GO:0046578	5.03E-08
type I interferon production	Biological Process	GO:0032606	5.06E-08
regulation of metal ion transport	Biological Process	GO:0010959	5.19E-08
membrane protein proteolysis	Biological Process	GO:0033619	5.30E-08
positive regulation of DNA binding	Biological Process	GO:0043388	5.30E-08
neuronal stem cell population maintenance	Biological Process	GO:0097150	5.30E-08
negative regulation of leukocyte activation	Biological Process	GO:0002695	5.32E-08
biomineral tissue development	Biological Process	GO:0031214	5.33E-08
biomineralization	Biological Process	GO:0110148	5.33E-08
regulation of tumor necrosis factor superfamily cytokine production	Biological Process	GO:1903555	5.33E-08
negative regulation of cell-matrix adhesion	Biological Process	GO:0001953	5.36E-08
skeletal system morphogenesis	Biological Process	GO:0048705	5.48E-08
response to chemokine	Biological Process	GO:1990868	5.79E-08
cellular response to chemokine	Biological Process	GO:1990869	5.79E-08

macrophage derived foam cell differentiation	Biological Process	GO:0010742	5.83E-08
foam cell differentiation	Biological Process	GO:0090077	5.83E-08
cellular glucose homeostasis	Biological Process	GO:0001678	5.87E-08
regulation of small GTPase mediated signal transduction	Biological Process	GO:0051056	6.36E-08
pancreas development	Biological Process	GO:0031016	6.52E-08
regulation of B cell apoptotic process	Biological Process	GO:0002902	6.52E-08
actin filament bundle assembly	Biological Process	GO:0051017	6.67E-08
negative regulation of intrinsic apoptotic signaling pathway	Biological Process	GO:2001243	6.84E-08
post-embryonic development	Biological Process	GO:0009791	6.90E-08
skin epidermis development	Biological Process	GO:0098773	6.90E-08
regulation of neural precursor cell proliferation	Biological Process	GO:2000177	6.90E-08
regulation of response to cytokine stimulus	Biological Process	GO:0060759	7.10E-08
ERBB signaling pathway	Biological Process	GO:0038127	7.22E-08
miRNA metabolic process	Biological Process	GO:0010586	7.70E-08
regulation of macrophage derived foam cell differentiation	Biological Process	GO:0010743	7.70E-08
outflow tract morphogenesis	Biological Process	GO:0003151	7.89E-08
developmental maturation	Biological Process	GO:0021700	7.96E-08
regulation of stem cell proliferation	Biological Process	GO:0072091	8.49E-08
protein modification by small protein removal	Biological Process	GO:0070646	8.65E-08
cardiac muscle cell differentiation	Biological Process	GO:0055007	8.73E-08
positive regulation of protein-containing complex assembly	Biological Process	GO:0031334	8.73E-08
cellular response to xenobiotic stimulus	Biological Process	GO:0071466	9.33E-08
regulation of interleukin-1 beta production	Biological Process	GO:0032651	9.87E-08
skin development	Biological Process	GO:0043588	1.01E-07
positive regulation of tyrosine phosphorylation of STAT protein	Biological Process	GO:0042531	1.05E-07
glomerulus development	Biological Process	GO:0032835	1.05E-07
regulation of pathway-restricted SMAD protein phosphorylation	Biological Process	GO:0060393	1.05E-07
BMP signaling pathway	Biological Process	GO:0030509	1.09E-07
negative regulation of ion transport	Biological Process	GO:0043271	1.09E-07
actin filament bundle organization	Biological Process	GO:0061572	1.09E-07
positive regulation of glucose metabolic process	Biological Process	GO:0010907	1.13E-07
interleukin-17 production	Biological Process	GO:0032620	1.13E-07
peptidyl-threonine modification	Biological Process	GO:0018210	1.14E-07
negative regulation of transforming growth factor beta receptor signaling pathway	Biological Process	GO:0030512	1.15E-07
regulation of production of miRNAs involved in gene silencing by miRNA	Biological Process	GO:1903798	1.15E-07
smooth muscle cell migration	Biological Process	GO:0014909	1.16E-07

molting cycle	Biological Process	GO:0042303	1.17E-07
hair cycle	Biological Process	GO:0042633	1.17E-07
negative regulation of lymphocyte activation	Biological Process	GO:0051250	1.20E-07
response to BMP	Biological Process	GO:0071772	1.20E-07
cellular response to BMP stimulus	Biological Process	GO:0071773	1.20E-07
movement in host environment	Biological Process	GO:0052126	1.22E-07
regulation of mononuclear cell migration	Biological Process	GO:0071675	1.26E-07
negative regulation of myoblast differentiation	Biological Process	GO:0045662	1.31E-07
positive regulation of transcription regulatory region DNA binding	Biological Process	GO:2000679	1.31E-07
protein localization to cell periphery	Biological Process	GO:1990778	1.35E-07
negative regulation of developmental growth	Biological Process	GO:0048640	1.36E-07
negative regulation of Wnt signaling pathway	Biological Process	GO:0030178	1.50E-07
regulation of interleukin-1 production	Biological Process	GO:0032652	1.52E-07
morphogenesis of an epithelial sheet	Biological Process	GO:0002011	1.54E-07
regulation of phosphatidylinositol 3-kinase activity	Biological Process	GO:0043551	1.54E-07
regulation of chromatin organization	Biological Process	GO:1902275	1.60E-07
negative regulation of cardiac muscle tissue growth	Biological Process	GO:0055022	1.60E-07
negative regulation of heart growth	Biological Process	GO:0061117	1.60E-07
cell surface receptor signaling pathway involved in heart development	Biological Process	GO:0061311	1.60E-07
regulation of neuron projection regeneration	Biological Process	GO:0070570	1.60E-07
apical junction assembly	Biological Process	GO:0043297	1.61E-07
positive regulation of T cell differentiation	Biological Process	GO:0045582	1.63E-07
negative regulation of cellular response to transforming growth factor beta stimulus	Biological Process	GO:1903845	1.63E-07
regulation of CD4-positive, alpha-beta T cell differentiation	Biological Process	GO:0043370	1.63E-07
negative regulation of leukocyte apoptotic process	Biological Process	GO:2000107	1.63E-07
regulation of extrinsic apoptotic signaling pathway in absence of ligand	Biological Process	GO:2001239	1.63E-07
eye morphogenesis	Biological Process	GO:0048592	1.73E-07
muscle cell migration	Biological Process	GO:0014812	1.76E-07
nephron tubule morphogenesis	Biological Process	GO:0072078	1.87E-07
positive regulation of lymphocyte differentiation	Biological Process	GO:0045621	1.90E-07
kidney morphogenesis	Biological Process	GO:0060993	1.90E-07
regulation of biomineral tissue development	Biological Process	GO:0070167	1.90E-07
regulation of biomineralization	Biological Process	GO:0110149	1.90E-07
regulation of morphogenesis of a branching structure	Biological Process	GO:0060688	1.92E-07
regulation of kidney development	Biological Process	GO:0090183	1.92E-07
negative regulation of oxidative stress-induced cell death	Biological Process	GO:1903202	1.92E-07

macroautophagy	Biological Process	GO:0016236	1.93E-07
negative regulation of autophagy	Biological Process	GO:0010507	1.94E-07
regulation of smooth muscle cell migration	Biological Process	GO:0014910	1.94E-07
monocyte chemotaxis	Biological Process	GO:0002548	1.96E-07
ureteric bud morphogenesis	Biological Process	GO:0060675	1.96E-07
endothelial cell apoptotic process	Biological Process	GO:0072577	1.96E-07
T cell migration	Biological Process	GO:0072678	1.96E-07
regulation of anion transmembrane transport	Biological Process	GO:1903959	1.97E-07
cellular divalent inorganic cation homeostasis	Biological Process	GO:0072503	2.00E-07
B cell activation	Biological Process	GO:0042113	2.01E-07
negative regulation of organ growth	Biological Process	GO:0046621	2.02E-07
exocrine system development	Biological Process	GO:0035272	2.10E-07
regulation of response to biotic stimulus	Biological Process	GO:0002831	2.12E-07
positive regulation of cytokine secretion	Biological Process	GO:0050715	2.12E-07
regulation of gene silencing	Biological Process	GO:0060968	2.12E-07
regulation of protein localization to plasma membrane	Biological Process	GO:1903076	2.21E-07
negative regulation of cyclin-dependent protein serine/threonine kinase activity	Biological Process	GO:0045736	2.22E-07
regulation of insulin secretion	Biological Process	GO:0050796	2.29E-07
mesenchymal cell development	Biological Process	GO:0014031	2.29E-07
positive regulation of axonogenesis	Biological Process	GO:0050772	2.29E-07
epidermis development	Biological Process	GO:0008544	2.36E-07
platelet-derived growth factor receptor signaling pathway	Biological Process	GO:0048008	2.38E-07
regulation of cardiac muscle cell proliferation	Biological Process	GO:0060043	2.38E-07
negative regulation of cellular response to oxidative stress	Biological Process	GO:1900408	2.38E-07
positive regulation of reactive oxygen species biosynthetic process	Biological Process	GO:1903428	2.38E-07
mesonephric tubule morphogenesis	Biological Process	GO:0072171	2.39E-07
regulation of cardiocyte differentiation	Biological Process	GO:1905207	2.39E-07
cellular response to light stimulus	Biological Process	GO:0071482	2.59E-07
response to temperature stimulus	Biological Process	GO:0009266	2.62E-07
Rho protein signal transduction	Biological Process	GO:0007266	2.64E-07
nephron epithelium morphogenesis	Biological Process	GO:0072088	2.66E-07
hair follicle development	Biological Process	GO:0001942	2.70E-07
activation of cysteine-type endopeptidase activity involved in apoptotic process	Biological Process	GO:0006919	2.70E-07
negative regulation of canonical Wnt signaling pathway	Biological Process	GO:0090090	2.82E-07
bone development	Biological Process	GO:0060348	2.86E-07
regulation of protein ubiquitination	Biological Process	GO:0031396	2.90E-07

regulation of hormone biosynthetic process	Biological Process	GO:0046885	2.90E-07
astrocyte activation	Biological Process	GO:0048143	2.90E-07
cardiac ventricle development	Biological Process	GO:0003231	2.93E-07
muscle adaptation	Biological Process	GO:0043500	2.93E-07
prostate gland growth	Biological Process	GO:0060736	2.99E-07
cell-substrate junction assembly	Biological Process	GO:0007044	2.99E-07
regulation of actin filament bundle assembly	Biological Process	GO:0032231	2.99E-07
oligodendrocyte differentiation	Biological Process	GO:0048709	2.99E-07
cell-substrate junction organization	Biological Process	GO:0150115	2.99E-07
negative regulation of cyclin-dependent protein kinase activity	Biological Process	GO:1904030	3.06E-07
response to hyperoxia	Biological Process	GO:0055093	3.06E-07
regulation of production of small RNA involved in gene silencing by RNA	Biological Process	GO:0070920	3.06E-07
epidermal growth factor receptor signaling pathway	Biological Process	GO:0007173	3.07E-07
inner ear development	Biological Process	GO:0048839	3.17E-07
regulation of cell junction assembly	Biological Process	GO:1901888	3.17E-07
nuclear division	Biological Process	GO:0000280	3.28E-07
protein localization to plasma membrane	Biological Process	GO:0072659	3.41E-07
endoderm formation	Biological Process	GO:0001706	3.41E-07
lung morphogenesis	Biological Process	GO:0060425	3.41E-07
cellular response to interferon-gamma	Biological Process	GO:0071346	3.44E-07
membrane protein ectodomain proteolysis	Biological Process	GO:0006509	3.52E-07
negative regulation of striated muscle cell differentiation	Biological Process	GO:0051154	3.52E-07
branching involved in ureteric bud morphogenesis	Biological Process	GO:0001658	3.66E-07
neuron projection regeneration	Biological Process	GO:0031102	3.66E-07
negative regulation of response to oxidative stress	Biological Process	GO:1902883	3.66E-07
molting cycle process	Biological Process	GO:0022404	3.72E-07
hair cycle process	Biological Process	GO:0022405	3.72E-07
chemokine-mediated signaling pathway	Biological Process	GO:0070098	3.72E-07
positive regulation of muscle tissue development	Biological Process	GO:1901863	3.72E-07
regulation of epithelial cell apoptotic process	Biological Process	GO:1904035	3.72E-07
renal tubule morphogenesis	Biological Process	GO:0061333	3.76E-07
regulation of lipid localization	Biological Process	GO:1905952	3.78E-07
response to interleukin-1	Biological Process	GO:0070555	3.81E-07
hepatocyte growth factor receptor signaling pathway	Biological Process	GO:0048012	3.85E-07
prostate gland morphogenesis	Biological Process	GO:0060512	4.18E-07
response to angiotensin	Biological Process	GO:1990776	4.18E-07

cellular response to tumor necrosis factor	Biological Process	GO:0071356	4.18E-07
regulation of peptide hormone secretion	Biological Process	GO:0090276	4.19E-07
cellular response to glucose stimulus	Biological Process	GO:0071333	4.23E-07
neuron migration	Biological Process	GO:0001764	4.23E-07
positive regulation of stress fiber assembly	Biological Process	GO:0051496	4.29E-07
learning	Biological Process	GO:0007612	4.30E-07
somite development	Biological Process	GO:0061053	4.36E-07
protein dephosphorylation	Biological Process	GO:0006470	4.45E-07
positive regulation of phospholipase C activity	Biological Process	GO:0010863	4.57E-07
positive regulation of nitric oxide biosynthetic process	Biological Process	GO:0045429	4.57E-07
positive regulation of axon extension	Biological Process	GO:0045773	4.57E-07
entry into host	Biological Process	GO:0044409	4.78E-07
negative regulation of cellular senescence	Biological Process	GO:2000773	4.79E-07
cytokinesis	Biological Process	GO:0000910	5.01E-07
metanephros development	Biological Process	GO:0001656	5.13E-07
neural crest cell differentiation	Biological Process	GO:0014033	5.13E-07
somitogenesis	Biological Process	GO:0001756	5.16E-07
cellular response to hexose stimulus	Biological Process	GO:0071331	5.41E-07
positive regulation of protein modification by small protein conjugation or removal	Biological Process	GO:1903322	5.41E-07
negative regulation of T cell activation	Biological Process	GO:0050868	5.42E-07
positive regulation of stress-activated MAPK cascade	Biological Process	GO:0032874	5.57E-07
lung epithelium development	Biological Process	GO:0060428	5.63E-07
amyloid-beta clearance	Biological Process	GO:0097242	5.63E-07
negative regulation of signal transduction in absence of ligand	Biological Process	GO:1901099	5.63E-07
negative regulation of extrinsic apoptotic signaling pathway in absence of ligand	Biological Process	GO:2001240	5.63E-07
negative regulation of neuron differentiation	Biological Process	GO:0045665	5.80E-07
regulation of DNA binding	Biological Process	GO:0051101	5.88E-07
regulation of tumor necrosis factor production	Biological Process	GO:0032680	5.88E-07
mesenchymal cell proliferation	Biological Process	GO:0010463	5.93E-07
positive regulation of nitric oxide metabolic process	Biological Process	GO:1904407	5.93E-07
interleukin-6-mediated signaling pathway	Biological Process	GO:0070102	5.95E-07
negative regulation of cellular catabolic process	Biological Process	GO:0031330	5.98E-07
cellular response to monosaccharide stimulus	Biological Process	GO:0071326	6.07E-07
positive regulation of stress-activated protein kinase signaling cascade	Biological Process	GO:0070304	6.14E-07
positive regulation of small GTPase mediated signal transduction	Biological Process	GO:0051057	6.17E-07
organelle fission	Biological Process	GO:0048285	6.17E-07

programmed cell death involved in cell development	Biological Process	GO:0010623	6.41E-07
response to ethanol	Biological Process	GO:0045471	6.67E-07
regulation of cell proliferation involved in heart morphogenesis	Biological Process	GO:2000136	6.69E-07
positive regulation of mitotic nuclear division	Biological Process	GO:0045840	6.77E-07
protein deubiquitination	Biological Process	GO:0016579	6.77E-07
regulation of cation transmembrane transport	Biological Process	GO:1904062	6.79E-07
regulation of cellular carbohydrate metabolic process	Biological Process	GO:0010675	6.83E-07
regulation of sprouting angiogenesis	Biological Process	GO:1903670	6.83E-07
bone mineralization	Biological Process	GO:0030282	7.06E-07
lung cell differentiation	Biological Process	GO:0060479	7.31E-07
positive regulation of lipase activity	Biological Process	GO:0060193	7.41E-07
regulation of cartilage development	Biological Process	GO:0061035	7.41E-07
regulation of polysaccharide biosynthetic process	Biological Process	GO:0032885	7.52E-07
peptidyl-tyrosine autophosphorylation	Biological Process	GO:0038083	7.52E-07
cellular response to metal ion	Biological Process	GO:0071248	7.58E-07
endocrine pancreas development	Biological Process	GO:0031018	7.59E-07
endodermal cell differentiation	Biological Process	GO:0035987	7.59E-07
regulation of phospholipase C activity	Biological Process	GO:1900274	7.59E-07
tumor necrosis factor production	Biological Process	GO:0032640	8.07E-07
regulation of interleukin-12 production	Biological Process	GO:0032655	8.46E-07
regulation of cytokine-mediated signaling pathway	Biological Process	GO:0001959	9.25E-07
cell maturation	Biological Process	GO:0048469	9.25E-07
regulation of cell projection assembly	Biological Process	GO:0060491	9.25E-07
positive regulation of JNK cascade	Biological Process	GO:0046330	9.79E-07
positive regulation of cellular protein catabolic process	Biological Process	GO:1903364	9.79E-07
mammary gland morphogenesis	Biological Process	GO:0060443	9.82E-07
positive regulation of transmembrane transport	Biological Process	GO:0034764	9.92E-07
histone phosphorylation	Biological Process	GO:0016572	1.00E-06
regulation of hormone metabolic process	Biological Process	GO:0032350	1.00E-06
lymphocyte chemotaxis	Biological Process	GO:0048247	1.01E-06
animal organ formation	Biological Process	GO:0048645	1.01E-06
regulation of cytokine production involved in immune response	Biological Process	GO:0002718	1.03E-06
regulation of striated muscle cell differentiation	Biological Process	GO:0051153	1.05E-06
platelet activation	Biological Process	GO:0030168	1.06E-06
atrioventricular valve morphogenesis	Biological Process	GO:0003181	1.10E-06
positive regulation of mononuclear cell migration	Biological Process	GO:0071677	1.10E-06

regulation of anoikis	Biological Process	GO:2000209	1.10E-06
cardiac muscle hypertrophy	Biological Process	GO:0003300	1.10E-06
neurotransmitter biosynthetic process	Biological Process	GO:0042136	1.10E-06
mammary gland alveolus development	Biological Process	GO:0060749	1.12E-06
cell proliferation involved in heart morphogenesis	Biological Process	GO:0061323	1.12E-06
mammary gland lobule development	Biological Process	GO:0061377	1.12E-06
cellular response to interleukin-1	Biological Process	GO:0071347	1.12E-06
stem cell development	Biological Process	GO:0048864	1.21E-06
regulation of protein-containing complex assembly	Biological Process	GO:0043254	1.24E-06
positive regulation of smooth muscle cell migration	Biological Process	GO:0014911	1.24E-06
T cell selection	Biological Process	GO:0045058	1.24E-06
positive regulation of alpha-beta T cell differentiation	Biological Process	GO:0046638	1.24E-06
genitalia development	Biological Process	GO:0048806	1.24E-06
amyloid-beta metabolic process	Biological Process	GO:0050435	1.24E-06
response to epidermal growth factor	Biological Process	GO:0070849	1.24E-06
DNA damage response, signal transduction by p53 class mediator	Biological Process	GO:0030330	1.25E-06
regulation of intracellular steroid hormone receptor signaling pathway	Biological Process	GO:0033143	1.25E-06
response to antineoplastic agent	Biological Process	GO:0097327	1.26E-06
regulation of vitamin metabolic process	Biological Process	GO:0030656	1.27E-06
negative regulation of pri-miRNA transcription by RNA polymerase II	Biological Process	GO:1902894	1.27E-06
insulin secretion	Biological Process	GO:0030073	1.29E-06
interleukin-12 production	Biological Process	GO:0032615	1.29E-06
heart process	Biological Process	GO:0003015	1.31E-06
acute inflammatory response	Biological Process	GO:0002526	1.44E-06
regulation of cell cycle arrest	Biological Process	GO:0071156	1.44E-06
peptide hormone secretion	Biological Process	GO:0030072	1.46E-06
regulation of multicellular organism growth	Biological Process	GO:0040014	1.46E-06
cranial skeletal system development	Biological Process	GO:1904888	1.46E-06
cellular response to carbohydrate stimulus	Biological Process	GO:0071322	1.53E-06
regulation of embryonic development	Biological Process	GO:0045995	1.56E-06
positive regulation of pathway-restricted SMAD protein phosphorylation	Biological Process	GO:0010862	1.56E-06
response to mineralocorticoid	Biological Process	GO:0051385	1.59E-06
striated muscle hypertrophy	Biological Process	GO:0014897	1.64E-06
calcium ion transport	Biological Process	GO:0006816	1.64E-06
positive regulation of striated muscle tissue development	Biological Process	GO:0045844	1.64E-06
positive regulation of muscle organ development	Biological Process	GO:0048636	1.64E-06

positive regulation of cell division	Biological Process	GO:0051781	1.64E-06
regulation of axonogenesis	Biological Process	GO:0050770	1.65E-06
cell migration involved in sprouting angiogenesis	Biological Process	GO:0002042	1.68E-06
regulation of lipase activity	Biological Process	GO:0060191	1.68E-06
positive regulation of kidney development	Biological Process	GO:0090184	1.71E-06
axon extension	Biological Process	GO:0048675	1.73E-06
positive regulation of epithelial cell differentiation	Biological Process	GO:0030858	1.75E-06
negative regulation of smooth muscle cell proliferation	Biological Process	GO:0048662	1.75E-06
morphogenesis of an endothelium	Biological Process	GO:0003159	1.78E-06
positive regulation of glial cell proliferation	Biological Process	GO:0060252	1.78E-06
trachea development	Biological Process	GO:0060438	1.78E-06
endothelial tube morphogenesis	Biological Process	GO:0061154	1.78E-06
positive regulation of cellular response to oxidative stress	Biological Process	GO:1900409	1.78E-06
calcium ion transport into cytosol	Biological Process	GO:0060402	1.78E-06
regulation of lipid biosynthetic process	Biological Process	GO:0046890	1.93E-06
regulation of transmembrane transporter activity	Biological Process	GO:0022898	1.93E-06
negative regulation of small molecule metabolic process	Biological Process	GO:0062014	1.93E-06
positive regulation of cellular carbohydrate metabolic process	Biological Process	GO:0010676	1.93E-06
negative regulation of small GTPase mediated signal transduction	Biological Process	GO:0051058	1.93E-06
divalent inorganic cation transport	Biological Process	GO:0072511	1.94E-06
cellular response to nerve growth factor stimulus	Biological Process	GO:1990090	1.94E-06
positive regulation of extrinsic apoptotic signaling pathway	Biological Process	GO:2001238	1.94E-06
regulation of transporter activity	Biological Process	GO:0032409	1.98E-06
negative regulation of secretion by cell	Biological Process	GO:1903531	1.99E-06
lipid storage	Biological Process	GO:0019915	2.09E-06
muscle hypertrophy	Biological Process	GO:0014896	2.11E-06
response to iron ion	Biological Process	GO:0010039	2.13E-06
regulation of mesenchymal cell proliferation	Biological Process	GO:0010464	2.13E-06
response to isoquinoline alkaloid	Biological Process	GO:0014072	2.13E-06
response to morphine	Biological Process	GO:0043278	2.13E-06
neural tube development	Biological Process	GO:0021915	2.18E-06
activated T cell proliferation	Biological Process	GO:0050798	2.18E-06
brown fat cell differentiation	Biological Process	GO:0050873	2.18E-06
chondrocyte differentiation	Biological Process	GO:0002062	2.18E-06
negative regulation of angiogenesis	Biological Process	GO:0016525	2.20E-06
T cell lineage commitment	Biological Process	GO:0002360	2.26E-06

atrioventricular valve development	Biological Process	GO:0003171	2.26E-06
receptor biosynthetic process	Biological Process	GO:0032800	2.26E-06
prostate gland epithelium morphogenesis	Biological Process	GO:0060740	2.26E-06
cellular response to ethanol	Biological Process	GO:0071361	2.31E-06
regulation of wound healing	Biological Process	GO:0061041	2.33E-06
positive regulation of phospholipase activity	Biological Process	GO:0010518	2.33E-06
androgen receptor signaling pathway	Biological Process	GO:0030521	2.33E-06
regulation of glucose metabolic process	Biological Process	GO:0010906	2.38E-06
interleukin-6 production	Biological Process	GO:0032635	2.41E-06
regulation of cardiac muscle cell differentiation	Biological Process	GO:2000725	2.41E-06
actin cytoskeleton reorganization	Biological Process	GO:0031532	2.52E-06
odontogenesis of dentin-containing tooth	Biological Process	GO:0042475	2.54E-06
regulation of carbohydrate biosynthetic process	Biological Process	GO:0043255	2.54E-06
response to purine-containing compound	Biological Process	GO:0014074	2.59E-06
regulation of plasma membrane bounded cell projection assembly	Biological Process	GO:0120032	2.66E-06
negative regulation of blood vessel morphogenesis	Biological Process	GO:2000181	2.66E-06
regulation of cellular amide metabolic process	Biological Process	GO:0034248	2.72E-06
response to muramyl dipeptide	Biological Process	GO:0032495	2.75E-06
amyloid-beta formation	Biological Process	GO:0034205	2.82E-06
cell fate commitment involved in formation of primary germ layer	Biological Process	GO:0060795	2.82E-06
regulation of focal adhesion assembly	Biological Process	GO:0051893	2.82E-06
regulation of cell-substrate junction assembly	Biological Process	GO:0090109	2.82E-06
tight junction organization	Biological Process	GO:0120193	2.82E-06
regulation of cell-substrate junction organization	Biological Process	GO:0150116	2.82E-06
regulation of calcium ion transport into cytosol	Biological Process	GO:0010522	2.88E-06
cytosolic calcium ion transport	Biological Process	GO:0060401	2.92E-06
regulation of phospholipase activity	Biological Process	GO:0010517	2.93E-06
peptidyl-lysine modification	Biological Process	GO:0018205	3.03E-06
negative regulation of transmembrane receptor protein serine/threonine kinase signaling pathway	Biological Process	GO:0090101	3.11E-06
divalent metal ion transport	Biological Process	GO:0070838	3.18E-06
renal system vasculature development	Biological Process	GO:0061437	3.18E-06
kidney vasculature development	Biological Process	GO:0061440	3.18E-06
negative regulation of cardiocyte differentiation	Biological Process	GO:1905208	3.18E-06
regulation of innate immune response	Biological Process	GO:0045088	3.23E-06
neural crest cell development	Biological Process	GO:0014032	3.28E-06

regulation of production of molecular mediator of immune response	Biological Process	GO:0002700	3.39E-06
response to starvation	Biological Process	GO:0042594	3.41E-06
positive regulation of actin filament bundle assembly	Biological Process	GO:0032233	3.41E-06
collagen metabolic process	Biological Process	GO:0032963	3.47E-06
dendrite development	Biological Process	GO:0016358	3.54E-06
regulation of polysaccharide metabolic process	Biological Process	GO:0032881	3.54E-06
positive regulation of glial cell differentiation	Biological Process	GO:0045687	3.54E-06
cellular response to epidermal growth factor stimulus	Biological Process	GO:0071364	3.54E-06
negative regulation of Ras protein signal transduction	Biological Process	GO:0046580	3.68E-06
response to nerve growth factor	Biological Process	GO:1990089	3.68E-06
regulation of supramolecular fiber organization	Biological Process	GO:1902903	3.68E-06
nucleotide-binding oligomerization domain containing signaling pathway	Biological Process	GO:0070423	3.72E-06
cellular response to alkaloid	Biological Process	GO:0071312	3.72E-06
negative regulation of animal organ morphogenesis	Biological Process	GO:0110111	3.72E-06
positive regulation of cyclin-dependent protein kinase activity	Biological Process	GO:1904031	3.72E-06
body fluid secretion	Biological Process	GO:0007589	3.88E-06
hematopoietic progenitor cell differentiation	Biological Process	GO:0002244	3.93E-06
negative regulation of cellular response to growth factor stimulus	Biological Process	GO:0090288	3.93E-06
positive regulation of hormone metabolic process	Biological Process	GO:0032352	3.97E-06
glial cell apoptotic process	Biological Process	GO:0034349	3.97E-06
positive regulation of nitric-oxide synthase biosynthetic process	Biological Process	GO:0051770	3.97E-06
regulation of intracellular transport	Biological Process	GO:0032386	3.97E-06
bone resorption	Biological Process	GO:0045453	4.07E-06
positive regulation of alpha-beta T cell activation	Biological Process	GO:0046635	4.07E-06
intrinsic apoptotic signaling pathway in response to endoplasmic reticulum stress	Biological Process	GO:0070059	4.07E-06
DNA alkylation	Biological Process	GO:0006305	4.07E-06
DNA methylation	Biological Process	GO:0006306	4.07E-06
cell proliferation involved in kidney development	Biological Process	GO:0072111	4.10E-06
positive regulation of response to oxidative stress	Biological Process	GO:1902884	4.10E-06
regulation of bicellular tight junction assembly	Biological Process	GO:2000810	4.10E-06
regulation of neurotransmitter levels	Biological Process	GO:0001505	4.12E-06
mammary gland epithelial cell proliferation	Biological Process	GO:0033598	4.36E-06
response to increased oxygen levels	Biological Process	GO:0036296	4.36E-06
memory	Biological Process	GO:0007613	4.36E-06
positive regulation of protein ubiquitination	Biological Process	GO:0031398	4.36E-06
regulation of posttranscriptional gene silencing	Biological Process	GO:0060147	4.36E-06

regulation of gene silencing by RNA	Biological Process	GO:0060966	4.36E-06
DNA methylation or demethylation	Biological Process	GO:0044728	4.42E-06
amyloid precursor protein catabolic process	Biological Process	GO:0042987	4.42E-06
p38MAPK cascade	Biological Process	GO:0038066	4.46E-06
positive regulation of synaptic transmission	Biological Process	GO:0050806	4.73E-06
cardiac ventricle morphogenesis	Biological Process	GO:0003208	4.79E-06
nucleotide-binding domain, leucine rich repeat containing receptor signaling pathway	Biological Process	GO:0035872	4.83E-06
mitotic G1 DNA damage checkpoint	Biological Process	GO:0031571	4.85E-06
amyloid precursor protein metabolic process	Biological Process	GO:0042982	4.85E-06
mitotic G1/S transition checkpoint	Biological Process	GO:0044819	4.85E-06
positive regulation of ubiquitin-dependent protein catabolic process	Biological Process	GO:2000060	5.08E-06
negative regulation of cysteine-type endopeptidase activity involved in apoptotic process	Biological Process	GO:0043154	5.10E-06
regulation of tube diameter	Biological Process	GO:0035296	5.13E-06
regulation of blood vessel diameter	Biological Process	GO:0097746	5.13E-06
negative regulation of secretion	Biological Process	GO:0051048	5.15E-06
regulation of signaling receptor activity	Biological Process	GO:0010469	5.19E-06
establishment or maintenance of cell polarity	Biological Process	GO:0007163	5.29E-06
B cell differentiation	Biological Process	GO:0030183	5.41E-06
regulation of muscle adaptation	Biological Process	GO:0043502	5.44E-06
regulation of lipid catabolic process	Biological Process	GO:0050994	5.44E-06
positive regulation of oxidoreductase activity	Biological Process	GO:0051353	5.44E-06
blood vessel remodeling	Biological Process	GO:0001974	5.54E-06
negative regulation of T cell differentiation	Biological Process	GO:0045581	5.54E-06
negative regulation of defense response	Biological Process	GO:0031348	5.55E-06
cellular response to nutrient	Biological Process	GO:0031670	5.58E-06
smooth muscle cell differentiation	Biological Process	GO:0051145	5.58E-06
regulation of cellular response to insulin stimulus	Biological Process	GO:1900076	5.58E-06
regulation of tube size	Biological Process	GO:0035150	5.66E-06
negative regulation of DNA-binding transcription factor activity	Biological Process	GO:0043433	5.68E-06
G1 DNA damage checkpoint	Biological Process	GO:0044783	5.74E-06
positive regulation of Ras protein signal transduction	Biological Process	GO:0046579	5.74E-06
positive regulation of sprouting angiogenesis	Biological Process	GO:1903672	5.74E-06
negative regulation of reactive oxygen species metabolic process	Biological Process	GO:2000378	5.74E-06
regulation of muscle cell apoptotic process	Biological Process	GO:0010660	5.74E-06
regulation of fatty acid metabolic process	Biological Process	GO:0019217	5.74E-06

epithelial tube branching involved in lung morphogenesis	Biological Process	GO:0060441	5.90E-06
negative regulation of lymphocyte apoptotic process	Biological Process	GO:0070229	5.90E-06
negative regulation of Rho protein signal transduction	Biological Process	GO:0035024	5.96E-06
lung epithelial cell differentiation	Biological Process	GO:0060487	5.96E-06
positive regulation of transcription from RNA polymerase II promoter involved in cellular response to chemical stimulus	Biological Process	GO:1901522	5.96E-06
myelination	Biological Process	GO:0042552	5.97E-06
positive regulation of proteolysis involved in cellular protein catabolic process	Biological Process	GO:1903052	6.15E-06
regulation of activated T cell proliferation	Biological Process	GO:0046006	6.18E-06
regulation of actin cytoskeleton reorganization	Biological Process	GO:2000249	6.18E-06
DNA damage checkpoint	Biological Process	GO:0000077	6.23E-06
chemokine biosynthetic process	Biological Process	GO:0042033	6.45E-06
chemokine metabolic process	Biological Process	GO:0050755	6.45E-06
ventricular septum development	Biological Process	GO:0003281	6.49E-06
regulation of cardiac muscle hypertrophy	Biological Process	GO:0010611	6.49E-06
mitotic DNA damage checkpoint	Biological Process	GO:0044773	6.53E-06
bicellular tight junction assembly	Biological Process	GO:0070830	6.53E-06
regulation of lipid storage	Biological Process	GO:0010883	6.84E-06
protein destabilization	Biological Process	GO:0031648	6.84E-06
limbic system development	Biological Process	GO:0021761	6.88E-06
dephosphorylation	Biological Process	GO:0016311	6.91E-06
monosaccharide metabolic process	Biological Process	GO:0005996	7.19E-06
ensheathment of neurons	Biological Process	GO:0007272	7.42E-06
axon ensheathment	Biological Process	GO:0008366	7.42E-06
positive regulation of endocytosis	Biological Process	GO:0045807	7.47E-06
positive regulation of hormone biosynthetic process	Biological Process	GO:0046886	7.53E-06
bronchus development	Biological Process	GO:0060433	7.53E-06
Notch signaling involved in heart development	Biological Process	GO:0061314	7.53E-06
regulation of bone mineralization	Biological Process	GO:0030500	7.57E-06
regulation of adaptive immune response	Biological Process	GO:0002819	7.63E-06
viral genome replication	Biological Process	GO:0019079	7.71E-06
tight junction assembly	Biological Process	GO:0120192	7.89E-06
positive regulation of protein localization to plasma membrane	Biological Process	GO:1903078	7.89E-06
regulation of fatty acid oxidation	Biological Process	GO:0046320	7.89E-06
homeostasis of number of cells within a tissue	Biological Process	GO:0048873	7.89E-06
cellular response to gamma radiation	Biological Process	GO:0071480	7.89E-06

negative regulation of cell junction assembly	Biological Process	GO:1901889	7.89E-06
hexose metabolic process	Biological Process	GO:0019318	7.97E-06
regulation of insulin receptor signaling pathway	Biological Process	GO:0046626	8.03E-06
positive regulation of nuclear division	Biological Process	GO:0051785	8.03E-06
establishment of cell polarity	Biological Process	GO:0030010	8.19E-06
tube formation	Biological Process	GO:0035148	8.41E-06
T cell apoptotic process	Biological Process	GO:0070231	8.43E-06
modulation of process of other organism involved in symbiotic interaction	Biological Process	GO:0051817	8.45E-06
angiogenesis involved in wound healing	Biological Process	GO:0060055	8.51E-06
positive regulation of cell aging	Biological Process	GO:0090343	8.51E-06
negative regulation of cardiac muscle cell differentiation	Biological Process	GO:2000726	8.51E-06
actin filament organization	Biological Process	GO:0007015	8.67E-06
metencephalon development	Biological Process	GO:0022037	8.70E-06
regulation of JUN kinase activity	Biological Process	GO:0043506	8.83E-06
positive regulation of tumor necrosis factor superfamily cytokine production	Biological Process	GO:1903557	8.83E-06
regulation of phospholipid metabolic process	Biological Process	GO:1903725	8.83E-06
cellular response to fatty acid	Biological Process	GO:0071398	9.49E-06
muscle cell apoptotic process	Biological Process	GO:0010657	9.61E-06
release of sequestered calcium ion into cytosol	Biological Process	GO:0051209	9.61E-06
regulation of cellular protein catabolic process	Biological Process	GO:1903362	9.93E-06
glucose metabolic process	Biological Process	GO:0006006	1.00E-05
regulation of oligodendrocyte differentiation	Biological Process	GO:0048713	1.01E-05
vacuole organization	Biological Process	GO:0007033	1.01E-05
cardiac muscle cell development	Biological Process	GO:0055013	1.01E-05
heat generation	Biological Process	GO:0031649	1.01E-05
positive regulation of glycogen biosynthetic process	Biological Process	GO:0045725	1.01E-05
mammary gland epithelial cell differentiation	Biological Process	GO:0060644	1.01E-05
positive regulation of oxidative stress-induced cell death	Biological Process	GO:1903209	1.01E-05
associative learning	Biological Process	GO:0008306	1.01E-05
regulation of glucose transmembrane transport	Biological Process	GO:0010827	1.01E-05
regulation of muscle hypertrophy	Biological Process	GO:0014743	1.01E-05
tissue regeneration	Biological Process	GO:0042246	1.01E-05
negative regulation of MAP kinase activity	Biological Process	GO:0043407	1.01E-05
response to anesthetic	Biological Process	GO:0072347	1.01E-05
morphogenesis of embryonic epithelium	Biological Process	GO:0016331	1.02E-05
lactation	Biological Process	GO:0007595	1.03E-05

visual learning	Biological Process	GO:0008542	1.03E-05
regulation of bone remodeling	Biological Process	GO:0046850	1.03E-05
positive regulation of acute inflammatory response	Biological Process	GO:0002675	1.04E-05
response to X-ray	Biological Process	GO:0010165	1.04E-05
positive regulation of cyclin-dependent protein serine/threonine kinase activity	Biological Process	GO:0045737	1.04E-05
regulation of lipid transport	Biological Process	GO:0032368	1.06E-05
regulation of leukocyte mediated immunity	Biological Process	GO:0002703	1.08E-05
regulation of gene silencing by miRNA	Biological Process	GO:0060964	1.09E-05
positive chemotaxis	Biological Process	GO:0050918	1.11E-05
protein stabilization	Biological Process	GO:0050821	1.15E-05
vasculogenesis	Biological Process	GO:0001570	1.17E-05
negative regulation of sequestering of calcium ion	Biological Process	GO:0051283	1.19E-05
response to lead ion	Biological Process	GO:0010288	1.19E-05
positive regulation of activated T cell proliferation	Biological Process	GO:0042104	1.19E-05
fibroblast apoptotic process	Biological Process	GO:0044346	1.19E-05
regulation of intracellular protein transport	Biological Process	GO:0033157	1.22E-05
regulation of circadian rhythm	Biological Process	GO:0042752	1.23E-05
regulation of interleukin-6 production	Biological Process	GO:0032675	1.23E-05
regulation of mitochondrion organization	Biological Process	GO:0010821	1.25E-05
response to nicotine	Biological Process	GO:0035094	1.25E-05
digestive tract morphogenesis	Biological Process	GO:0048546	1.25E-05
programmed necrotic cell death	Biological Process	GO:0097300	1.25E-05
cell fate determination	Biological Process	GO:0001709	1.25E-05
modulation by symbiont of host process	Biological Process	GO:0044003	1.25E-05
regulation of bone resorption	Biological Process	GO:0045124	1.25E-05
metanephric nephron development	Biological Process	GO:0072210	1.25E-05
ovulation cycle	Biological Process	GO:0042698	1.29E-05
regulation of BMP signaling pathway	Biological Process	GO:0030510	1.31E-05
negative regulation of cation transmembrane transport	Biological Process	GO:1904063	1.31E-05
positive regulation of hormone secretion	Biological Process	GO:0046887	1.31E-05
regulation of sister chromatid segregation	Biological Process	GO:0033045	1.35E-05
neurotransmitter metabolic process	Biological Process	GO:0042133	1.35E-05
calcium ion transmembrane import into cytosol	Biological Process	GO:0097553	1.35E-05
regulation of cellular ketone metabolic process	Biological Process	GO:0010565	1.35E-05
regulation of blood circulation	Biological Process	GO:1903522	1.35E-05
viral RNA genome replication	Biological Process	GO:0039694	1.35E-05

positive regulation of phosphatidylinositol 3-kinase activity	Biological Process	GO:0043552	1.35E-05
negative regulation of B cell apoptotic process	Biological Process	GO:0002903	1.35E-05
vitamin D biosynthetic process	Biological Process	GO:0042368	1.35E-05
positive regulation of SMAD protein signal transduction	Biological Process	GO:0060391	1.35E-05
epithelial cell differentiation involved in prostate gland development	Biological Process	GO:0060742	1.35E-05
regulation of chromosome segregation	Biological Process	GO:0051983	1.37E-05
cellular carbohydrate metabolic process	Biological Process	GO:0044262	1.38E-05
heart contraction	Biological Process	GO:0060047	1.38E-05
platelet degranulation	Biological Process	GO:0002576	1.45E-05
regulation of sequestering of calcium ion	Biological Process	GO:0051282	1.45E-05
negative regulation of cysteine-type endopeptidase activity	Biological Process	GO:2000117	1.49E-05
response to cold	Biological Process	GO:0009409	1.52E-05
coronary vasculature development	Biological Process	GO:0060976	1.52E-05
response to muscle stretch	Biological Process	GO:0035994	1.52E-05
coronary vasculature morphogenesis	Biological Process	GO:0060977	1.52E-05
positive regulation of glycogen metabolic process	Biological Process	GO:0070875	1.52E-05
positive regulation of monocyte chemotaxis	Biological Process	GO:0090026	1.52E-05
regulation of amyloid-beta clearance	Biological Process	GO:1900221	1.52E-05
positive regulation of proteasomal protein catabolic process	Biological Process	GO:1901800	1.54E-05
hippocampus development	Biological Process	GO:0021766	1.54E-05
focal adhesion assembly	Biological Process	GO:0048041	1.54E-05
regulation of blood pressure	Biological Process	GO:0008217	1.60E-05
modulation by symbiont of host cellular process	Biological Process	GO:0044068	1.62E-05
positive regulation of osteoclast differentiation	Biological Process	GO:0045672	1.62E-05
apoptotic process involved in morphogenesis	Biological Process	GO:0060561	1.62E-05
morphogenesis of an epithelial fold	Biological Process	GO:0060571	1.62E-05
metanephric nephron morphogenesis	Biological Process	GO:0072273	1.62E-05
regulation of monocyte chemotaxis	Biological Process	GO:0090025	1.62E-05
autophagosome assembly	Biological Process	GO:0000045	1.68E-05
positive regulation of protein binding	Biological Process	GO:0032092	1.68E-05
positive regulation of BMP signaling pathway	Biological Process	GO:0030513	1.76E-05
myeloid cell apoptotic process	Biological Process	GO:0033028	1.76E-05
positive regulation of cell cycle arrest	Biological Process	GO:0071158	1.77E-05
regulation of cell migration involved in sprouting angiogenesis	Biological Process	GO:0090049	1.77E-05
neuroblast proliferation	Biological Process	GO:0007405	1.87E-05
regulation of B cell proliferation	Biological Process	GO:0030888	1.87E-05

negative regulation of lipid localization	Biological Process	GO:1905953	1.87E-05
positive regulation of intrinsic apoptotic signaling pathway	Biological Process	GO:2001244	1.87E-05
camera-type eye morphogenesis	Biological Process	GO:0048593	1.90E-05
DNA integrity checkpoint	Biological Process	GO:0031570	1.94E-05
regulation of generation of precursor metabolites and energy	Biological Process	GO:0043467	1.94E-05
mitotic DNA integrity checkpoint	Biological Process	GO:0044774	1.95E-05
sequestering of calcium ion	Biological Process	GO:0051208	1.98E-05
regulation of macroautophagy	Biological Process	GO:0016241	2.03E-05
B cell proliferation	Biological Process	GO:0042100	2.18E-05
positive regulation of T cell proliferation	Biological Process	GO:0042102	2.18E-05
regulation of anion transport	Biological Process	GO:0044070	2.18E-05
regulation of oxidoreductase activity	Biological Process	GO:0051341	2.20E-05
lymphocyte homeostasis	Biological Process	GO:0002260	2.21E-05
embryonic pattern specification	Biological Process	GO:0009880	2.21E-05
necrotic cell death	Biological Process	GO:0070265	2.21E-05
regulation of interleukin-10 production	Biological Process	GO:0032653	2.21E-05
regulation of transcription regulatory region DNA binding	Biological Process	GO:2000677	2.21E-05
positive regulation of intracellular transport	Biological Process	GO:0032388	2.21E-05
negative regulation of lipid storage	Biological Process	GO:0010888	2.25E-05
negative regulation of posttranscriptional gene silencing	Biological Process	GO:0060149	2.25E-05
negative regulation of gene silencing by RNA	Biological Process	GO:0060967	2.25E-05
salivary gland morphogenesis	Biological Process	GO:0007435	2.25E-05
positive T cell selection	Biological Process	GO:0043368	2.25E-05
regulation of DNA damage response, signal transduction by p53 class mediator	Biological Process	GO:0043516	2.25E-05
SMAD protein complex assembly	Biological Process	GO:0007183	2.32E-05
positive regulation of fibroblast migration	Biological Process	GO:0010763	2.32E-05
regulation of endothelial cell development	Biological Process	GO:1901550	2.32E-05
regulation of establishment of endothelial barrier	Biological Process	GO:1903140	2.32E-05
negative regulation of anion transmembrane transport	Biological Process	GO:1903960	2.32E-05
negative regulation of immune effector process	Biological Process	GO:0002698	2.35E-05
positive regulation of blood circulation	Biological Process	GO:1903524	2.35E-05
regulation of ion transmembrane transporter activity	Biological Process	GO:0032412	2.39E-05
chromatin organization involved in regulation of transcription	Biological Process	GO:0034401	2.41E-05
response to retinoic acid	Biological Process	GO:0032526	2.44E-05
glucose transmembrane transport	Biological Process	GO:1904659	2.44E-05
regulation of DNA biosynthetic process	Biological Process	GO:2000278	2.44E-05

autophagosome organization	Biological Process	GO:1905037	2.44E-05
purine ribonucleoside triphosphate biosynthetic process	Biological Process	GO:0009206	2.59E-05
positive regulation of protein localization to cell periphery	Biological Process	GO:1904377	2.59E-05
regulation of lymphocyte migration	Biological Process	GO:2000401	2.59E-05
visual behavior	Biological Process	GO:0007632	2.64E-05
purine nucleoside triphosphate metabolic process	Biological Process	GO:0009144	2.64E-05
regulation of epidermis development	Biological Process	GO:0045682	2.64E-05
positive regulation of response to wounding	Biological Process	GO:1903036	2.72E-05
negative regulation of production of molecular mediator of immune response	Biological Process	GO:0002701	2.86E-05
cardiac atrium development	Biological Process	GO:0003230	2.86E-05
positive regulation of interleukin-12 production	Biological Process	GO:0032735	2.86E-05
receptor catabolic process	Biological Process	GO:0032801	2.86E-05
regulation of T-helper cell differentiation	Biological Process	GO:0045622	2.86E-05
mesodermal cell differentiation	Biological Process	GO:0048333	2.86E-05
regulation of amyloid precursor protein catabolic process	Biological Process	GO:1902991	2.86E-05
positive regulation of morphogenesis of an epithelium	Biological Process	GO:1905332	2.86E-05
vascular endothelial growth factor signaling pathway	Biological Process	GO:0038084	2.94E-05
positive regulation of DNA-templated transcription, initiation	Biological Process	GO:2000144	2.96E-05
positive regulation of supramolecular fiber organization	Biological Process	GO:1902905	2.99E-05
positive regulation of tumor necrosis factor production	Biological Process	GO:0032760	3.01E-05
purine nucleoside triphosphate biosynthetic process	Biological Process	GO:0009145	3.03E-05
positive regulation of fat cell differentiation	Biological Process	GO:0045600	3.03E-05
regulation of extent of cell growth	Biological Process	GO:0061387	3.06E-05
synapse organization	Biological Process	GO:0050808	3.07E-05
positive regulation of intracellular protein transport	Biological Process	GO:0090316	3.07E-05
positive regulation of Notch signaling pathway	Biological Process	GO:0045747	3.15E-05
regulation of ubiquitin-dependent protein catabolic process	Biological Process	GO:2000058	3.16E-05
lymphoid progenitor cell differentiation	Biological Process	GO:0002320	3.23E-05
nitric-oxide synthase biosynthetic process	Biological Process	GO:0051767	3.23E-05
regulation of nitric-oxide synthase biosynthetic process	Biological Process	GO:0051769	3.23E-05
pericardium development	Biological Process	GO:0060039	3.23E-05
negative regulation of oxidative stress-induced neuron death	Biological Process	GO:1903204	3.23E-05
positive regulation of cellular senescence	Biological Process	GO:2000774	3.23E-05
negative regulation of organelle organization	Biological Process	GO:0010639	3.33E-05
regulation of cell shape	Biological Process	GO:0008360	3.47E-05
response to activity	Biological Process	GO:0014823	3.55E-05

thymus development	Biological Process	GO:0048538	3.59E-05
autophagy of mitochondrion	Biological Process	GO:0000422	3.60E-05
mitochondrion disassembly	Biological Process	GO:0061726	3.60E-05
regulation of immune effector process	Biological Process	GO:0002697	3.61E-05
monocyte differentiation	Biological Process	GO:0030224	3.61E-05
regulation of transforming growth factor beta production	Biological Process	GO:0071634	3.61E-05
positive regulation of lipid kinase activity	Biological Process	GO:0090218	3.61E-05
mononuclear cell differentiation	Biological Process	GO:1903131	3.61E-05
interleukin-10 production	Biological Process	GO:0032613	3.74E-05
regulation of macrophage activation	Biological Process	GO:0043030	3.74E-05
negative regulation of lymphocyte differentiation	Biological Process	GO:0045620	3.74E-05
cellular response to increased oxygen levels	Biological Process	GO:0036295	3.75E-05
CD8-positive, alpha-beta T cell differentiation	Biological Process	GO:0043374	3.75E-05
positive regulation of macrophage differentiation	Biological Process	GO:0045651	3.75E-05
chemokine (C-X-C motif) ligand 2 production	Biological Process	GO:0072567	3.75E-05
regulation of cell proliferation involved in kidney development	Biological Process	GO:1901722	3.75E-05
hexose transmembrane transport	Biological Process	GO:0008645	3.78E-05
muscle contraction	Biological Process	GO:0006936	3.88E-05
T cell chemotaxis	Biological Process	GO:0010818	3.88E-05
regulation of axon regeneration	Biological Process	GO:0048679	3.88E-05
regulation of amyloid-beta formation	Biological Process	GO:1902003	3.88E-05
positive regulation of leukocyte apoptotic process	Biological Process	GO:2000108	3.88E-05
positive regulation of plasma membrane bounded cell projection assembly	Biological Process	GO:0120034	3.91E-05
steroid metabolic process	Biological Process	GO:0008202	3.97E-05
purine ribonucleoside triphosphate metabolic process	Biological Process	GO:0009205	4.10E-05
histone deacetylation	Biological Process	GO:0016575	4.10E-05
positive regulation of type I interferon production	Biological Process	GO:0032481	4.10E-05
defense response to virus	Biological Process	GO:0051607	4.10E-05
interferon-gamma production	Biological Process	GO:0032609	4.21E-05
modulation of process of other organism	Biological Process	GO:0035821	4.21E-05
regulation of acute inflammatory response	Biological Process	GO:0002673	4.32E-05
positive regulation of nucleotide metabolic process	Biological Process	GO:0045981	4.32E-05
positive regulation of purine nucleotide metabolic process	Biological Process	GO:1900544	4.32E-05
lipid localization	Biological Process	GO:0010876	4.32E-05
regulation of cytokinesis	Biological Process	GO:0032465	4.37E-05
roof of mouth development	Biological Process	GO:0060021	4.37E-05

regulation of interferon-gamma production	Biological Process	GO:0032649	4.37E-05
regulation of receptor-mediated endocytosis	Biological Process	GO:0048259	4.37E-05
regulation of animal organ formation	Biological Process	GO:0003156	4.48E-05
salivary gland development	Biological Process	GO:0007431	4.48E-05
hyaluronan metabolic process	Biological Process	GO:0030212	4.48E-05
regulation of DNA-templated transcription, initiation	Biological Process	GO:2000142	4.48E-05
positive regulation of lymphocyte migration	Biological Process	GO:2000403	4.48E-05
negative regulation of cardiac muscle cell proliferation	Biological Process	GO:0060044	4.52E-05
monosaccharide transmembrane transport	Biological Process	GO:0015749	4.67E-05
cardiac muscle contraction	Biological Process	GO:0060048	4.68E-05
intrinsic apoptotic signaling pathway by p53 class mediator	Biological Process	GO:0072332	4.68E-05
regulation of membrane potential	Biological Process	GO:0042391	4.77E-05
cerebellum development	Biological Process	GO:0021549	4.90E-05
Fc receptor signaling pathway	Biological Process	GO:0038093	5.02E-05
negative regulation of cellular amide metabolic process	Biological Process	GO:0034249	5.04E-05
regulation of glycogen biosynthetic process	Biological Process	GO:0005979	5.05E-05
positive regulation of vascular endothelial growth factor production	Biological Process	GO:0010575	5.05E-05
regulation of glucan biosynthetic process	Biological Process	GO:0010962	5.05E-05
regulation of myeloid cell apoptotic process	Biological Process	GO:0033032	5.05E-05
heart formation	Biological Process	GO:0060914	5.05E-05
negative regulation of CD4-positive, alpha-beta T cell activation	Biological Process	GO:2000515	5.05E-05
signal transduction involved in mitotic G1 DNA damage checkpoint	Biological Process	GO:0072431	5.17E-05
intracellular signal transduction involved in G1 DNA damage checkpoint	Biological Process	GO:1902400	5.17E-05
acute-phase response	Biological Process	GO:0006953	5.17E-05
G0 to G1 transition	Biological Process	GO:0045023	5.17E-05
trabecula morphogenesis	Biological Process	GO:0061383	5.17E-05
regulation of p38MAPK cascade	Biological Process	GO:1900744	5.17E-05
positive regulation of ATP metabolic process	Biological Process	GO:1903580	5.17E-05
negative regulation of binding	Biological Process	GO:0051100	5.39E-05
integrin-mediated signaling pathway	Biological Process	GO:0007229	5.46E-05
glucose import	Biological Process	GO:0046323	5.47E-05
interferon-gamma-mediated signaling pathway	Biological Process	GO:0060333	5.54E-05
regulation of cellular component size	Biological Process	GO:0032535	5.54E-05
chromosome segregation	Biological Process	GO:0007059	5.54E-05
Wnt signaling pathway, calcium modulating pathway	Biological Process	GO:0007223	5.54E-05
negative regulation of alpha-beta T cell activation	Biological Process	GO:0046636	5.54E-05

insulin-like growth factor receptor signaling pathway	Biological Process	GO:0048009	5.54E-05
transforming growth factor beta production	Biological Process	GO:0071604	5.54E-05
positive regulation of epithelial cell apoptotic process	Biological Process	GO:1904037	5.54E-05
DNA modification	Biological Process	GO:0006304	5.70E-05
carbohydrate transmembrane transport	Biological Process	GO:0034219	5.70E-05
regulation of stem cell differentiation	Biological Process	GO:2000736	5.70E-05
endodermal cell fate commitment	Biological Process	GO:0001711	5.74E-05
V(D)J recombination	Biological Process	GO:0033151	5.74E-05
entry of bacterium into host cell	Biological Process	GO:0035635	5.74E-05
fat-soluble vitamin biosynthetic process	Biological Process	GO:0042362	5.74E-05
negative regulation of DNA damage response, signal transduction by p53 class mediator	Biological Process	GO:0043518	5.74E-05
regulation of chemokine biosynthetic process	Biological Process	GO:0045073	5.74E-05
positive regulation of fatty acid oxidation	Biological Process	GO:0046321	5.74E-05
post-embryonic animal organ development	Biological Process	GO:0048569	5.74E-05
lens development in camera-type eye	Biological Process	GO:0002088	6.03E-05
nucleoside triphosphate biosynthetic process	Biological Process	GO:0009142	6.03E-05
aorta development	Biological Process	GO:0035904	6.03E-05
cellular response to glucocorticoid stimulus	Biological Process	GO:0071385	6.03E-05
positive regulation of ion transport	Biological Process	GO:0043270	6.07E-05
regulation of proteolysis involved in cellular protein catabolic process	Biological Process	GO:1903050	6.08E-05
cellular response to fibroblast growth factor stimulus	Biological Process	GO:0044344	6.08E-05
regulation of smooth muscle cell differentiation	Biological Process	GO:0051150	6.16E-05
regulation of release of cytochrome c from mitochondria	Biological Process	GO:0090199	6.16E-05
inflammatory cell apoptotic process	Biological Process	GO:0006925	6.16E-05
regulation of receptor biosynthetic process	Biological Process	GO:0010869	6.16E-05
regulation of SMAD protein signal transduction	Biological Process	GO:0060390	6.16E-05
intestinal epithelial cell differentiation	Biological Process	GO:0060575	6.16E-05
negative regulation of lipid metabolic process	Biological Process	GO:0045833	6.17E-05
ribonucleoside triphosphate biosynthetic process	Biological Process	GO:0009201	6.24E-05
B cell homeostasis	Biological Process	GO:0001782	6.42E-05
negative regulation of cartilage development	Biological Process	GO:0061037	6.42E-05
myeloid leukocyte cytokine production	Biological Process	GO:0061082	6.42E-05
cellular response to dexamethasone stimulus	Biological Process	GO:0071549	6.42E-05
liver regeneration	Biological Process	GO:0097421	6.42E-05
regulation of translation	Biological Process	GO:0006417	6.55E-05
muscle cell development	Biological Process	GO:0055001	6.64E-05

fibroblast migration	Biological Process	GO:0010761	6.78E-05
regulation of intracellular estrogen receptor signaling pathway	Biological Process	GO:0033146	6.78E-05
positive regulation of stem cell proliferation	Biological Process	GO:2000648	6.78E-05
positive regulation of proteasomal ubiquitin-dependent protein catabolic process	Biological Process	GO:0032436	6.81E-05
negative regulation of response to DNA damage stimulus	Biological Process	GO:2001021	6.81E-05
protein deacetylation	Biological Process	GO:0006476	6.92E-05
embryonic skeletal system morphogenesis	Biological Process	GO:0048704	6.92E-05
regulation of ERBB signaling pathway	Biological Process	GO:1901184	6.92E-05
release of cytochrome c from mitochondria	Biological Process	GO:0001836	6.99E-05
endothelial cell development	Biological Process	GO:0001885	6.99E-05
signal transduction involved in mitotic cell cycle checkpoint	Biological Process	GO:0072413	6.99E-05
signal transduction involved in mitotic DNA damage checkpoint	Biological Process	GO:1902402	6.99E-05
signal transduction involved in mitotic DNA integrity checkpoint	Biological Process	GO:1902403	6.99E-05
regulation of endothelial cell apoptotic process	Biological Process	GO:2000351	6.99E-05
protein localization to nucleus	Biological Process	GO:0034504	7.05E-05
myeloid cell development	Biological Process	GO:0061515	7.14E-05
regulation of adaptive immune response based on somatic recombination of immune receptors built from immunoglobulin superfamily domains	Biological Process	GO:0002822	7.21E-05
regulation of collagen metabolic process	Biological Process	GO:0010712	7.32E-05
regulation of release of sequestered calcium ion into cytosol	Biological Process	GO:0051279	7.73E-05
skeletal muscle tissue development	Biological Process	GO:0007519	8.03E-05
regulation of reproductive process	Biological Process	GO:2000241	8.03E-05
negative regulation of hydrolase activity	Biological Process	GO:0051346	8.09E-05
adaptive immune response based on somatic recombination of immune receptors built from immunoglobulin superfamily domains	Biological Process	GO:0002460	8.10E-05
fever generation	Biological Process	GO:0001660	8.15E-05
response to lipoteichoic acid	Biological Process	GO:0070391	8.15E-05
cellular response to lipoteichoic acid	Biological Process	GO:0071223	8.15E-05
regulation of fibroblast migration	Biological Process	GO:0010762	8.17E-05
endothelial cell chemotaxis	Biological Process	GO:0035767	8.17E-05
negative regulation of intrinsic apoptotic signaling pathway in response to DNA damage	Biological Process	GO:1902230	8.17E-05
regulation of glucose import	Biological Process	GO:0046324	8.17E-05
response to dexamethasone	Biological Process	GO:0071548	8.28E-05
negative regulation of chondrocyte differentiation	Biological Process	GO:0032331	8.31E-05
CD8-positive, alpha-beta T cell activation	Biological Process	GO:0036037	8.31E-05
positive regulation of immune effector process	Biological Process	GO:0002699	8.49E-05

maintenance of location in cell	Biological Process	GO:0051651	8.49E-05
neutrophil homeostasis	Biological Process	GO:0001780	8.49E-05
positive regulation of heterotypic cell-cell adhesion	Biological Process	GO:0034116	8.49E-05
morphogenesis of an epithelial bud	Biological Process	GO:0060572	8.49E-05
negative regulation of gene silencing by miRNA	Biological Process	GO:0060965	8.49E-05
glomerular mesangium development	Biological Process	GO:0072109	8.49E-05
positive regulation of amyloid-beta formation	Biological Process	GO:1902004	8.49E-05
amyloid fibril formation	Biological Process	GO:1990000	8.49E-05
negative regulation of stress-activated MAPK cascade	Biological Process	GO:0032873	8.67E-05
negative regulation of stress-activated protein kinase signaling cascade	Biological Process	GO:0070303	8.67E-05
ribonucleoside triphosphate metabolic process	Biological Process	GO:0009199	8.68E-05
negative regulation of endopeptidase activity	Biological Process	GO:0010951	8.75E-05
receptor-mediated endocytosis	Biological Process	GO:0006898	9.06E-05
chromatin remodeling	Biological Process	GO:0006338	9.10E-05
positive regulation of cell-substrate adhesion	Biological Process	GO:0010811	9.23E-05
viral entry into host cell	Biological Process	GO:0046718	9.23E-05
regulation of ATP metabolic process	Biological Process	GO:1903578	9.23E-05
mitotic cytokinesis	Biological Process	GO:0000281	9.36E-05
cellular extravasation	Biological Process	GO:0045123	9.45E-05
regulation of epidermal cell differentiation	Biological Process	GO:0045604	9.45E-05
cellular response to corticosteroid stimulus	Biological Process	GO:0071384	9.45E-05
regulation of axon extension	Biological Process	GO:0030516	9.68E-05
positive regulation of interleukin-6 production	Biological Process	GO:0032755	9.68E-05
striated muscle contraction	Biological Process	GO:0006941	9.96E-05
establishment of endothelial barrier	Biological Process	GO:0061028	1.00E-04
nucleoside triphosphate metabolic process	Biological Process	GO:0009141	0.000101
positive regulation of muscle cell differentiation	Biological Process	GO:0051149	0.000101
regulation of dendrite development	Biological Process	GO:0050773	0.000101
chromatin organization involved in negative regulation of transcription	Biological Process	GO:0097549	0.000102
embryo implantation	Biological Process	GO:0007566	0.000102
axon regeneration	Biological Process	GO:0031103	0.000102
negative regulation of fat cell differentiation	Biological Process	GO:0045599	0.000102
cochlea development	Biological Process	GO:0090102	0.000102
branching involved in blood vessel morphogenesis	Biological Process	GO:0001569	0.000102
metanephros morphogenesis	Biological Process	GO:0003338	0.000102
ERBB2 signaling pathway	Biological Process	GO:0038128	0.000102

placenta blood vessel development	Biological Process	GO:0060674	0.000102
signal transduction involved in DNA integrity checkpoint	Biological Process	GO:0072401	0.000107
signal transduction involved in DNA damage checkpoint	Biological Process	GO:0072422	0.000107
somatic diversification of immune receptors via germline recombination within a single locus	Biological Process	GO:0002562	0.000109
somatic cell DNA recombination	Biological Process	GO:0016444	0.000109
circadian regulation of gene expression	Biological Process	GO:0032922	0.000109
positive regulation of cardiac muscle tissue development	Biological Process	GO:0055025	0.000109
regulation of chromosome separation	Biological Process	GO:1905818	0.000109
endocardial cushion formation	Biological Process	GO:0003272	0.00011
negative regulation of alpha-beta T cell differentiation	Biological Process	GO:0046639	0.00011
positive regulation of vascular endothelial cell proliferation	Biological Process	GO:1905564	0.00011
non-canonical Wnt signaling pathway	Biological Process	GO:0035567	0.00011
response to fibroblast growth factor	Biological Process	GO:0071774	0.00011
carbohydrate biosynthetic process	Biological Process	GO:0016051	0.000111
granulocyte chemotaxis	Biological Process	GO:0071621	0.000111
production of molecular mediator of immune response	Biological Process	GO:0002440	0.000114
positive regulation of Wnt signaling pathway	Biological Process	GO:0030177	0.000115
regulation of calcium-mediated signaling	Biological Process	GO:0050848	0.00012
ATP biosynthetic process	Biological Process	GO:0006754	0.00012
regulation of chondrocyte differentiation	Biological Process	GO:0032330	0.00012
positive regulation of interleukin-1 beta production	Biological Process	GO:0032731	0.00012
positive regulation of JUN kinase activity	Biological Process	GO:0043507	0.000121
signal transduction involved in cell cycle checkpoint	Biological Process	GO:0072395	0.000121
apoptotic mitochondrial changes	Biological Process	GO:0008637	0.000122
epithelial to mesenchymal transition involved in endocardial cushion formation	Biological Process	GO:0003198	0.000122
positive regulation of macrophage derived foam cell differentiation	Biological Process	GO:0010744	0.000122
natural killer cell activation	Biological Process	GO:0030101	0.000124
regulation of epidermal growth factor receptor signaling pathway	Biological Process	GO:0042058	0.000124
mammary gland duct morphogenesis	Biological Process	GO:0060603	0.000128
regulation of neuroblast proliferation	Biological Process	GO:1902692	0.000128
positive regulation of cellular amide metabolic process	Biological Process	GO:0034250	0.000129
regulation of calcium ion transmembrane transport	Biological Process	GO:1903169	0.000129
positive regulation of cytoskeleton organization	Biological Process	GO:0051495	0.000135
DNA biosynthetic process	Biological Process	GO:0071897	0.000136
TRAIL-activated apoptotic signaling pathway	Biological Process	GO:0036462	0.000137

trachea morphogenesis	Biological Process	GO:0060439	0.000137
epithelial cell proliferation involved in prostate gland development	Biological Process	GO:0060767	0.000137
glomerular mesangial cell proliferation	Biological Process	GO:0072110	0.000137
somatic diversification of immune receptors	Biological Process	GO:0002200	0.000137
peripheral nervous system development	Biological Process	GO:0007422	0.000137
activation of MAPKK activity	Biological Process	GO:0000186	0.000141
regulation of histone acetylation	Biological Process	GO:0035065	0.000141
interleukin-1 beta secretion	Biological Process	GO:0050702	0.000141
positive regulation of mesenchymal cell proliferation	Biological Process	GO:0002053	0.000143
negative regulation of cytokine production involved in immune response	Biological Process	GO:0002719	0.000143
mRNA transcription	Biological Process	GO:0009299	0.000143
branching involved in mammary gland duct morphogenesis	Biological Process	GO:0060444	0.000143
glomerulus vasculature development	Biological Process	GO:0072012	0.000143
cellular response to angiotensin	Biological Process	GO:1904385	0.000143
negative regulation of DNA-dependent DNA replication	Biological Process	GO:2000104	0.000143
maternal process involved in female pregnancy	Biological Process	GO:0060135	0.000144
intrinsic apoptotic signaling pathway in response to oxidative stress	Biological Process	GO:0008631	0.000144
T-helper 1 type immune response	Biological Process	GO:0042088	0.000144
regulation of cellular response to drug	Biological Process	GO:2001038	0.000144
pattern recognition receptor signaling pathway	Biological Process	GO:0002221	0.000145
proteasomal protein catabolic process	Biological Process	GO:0010498	0.000145
regulation of protein tyrosine kinase activity	Biological Process	GO:0061097	0.000156
negative regulation of protein modification by small protein conjugation or removal	Biological Process	GO:1903321	0.000156
negative regulation of proteolysis	Biological Process	GO:0045861	0.000158
regulation of vascular endothelial growth factor production	Biological Process	GO:0010574	0.000158
activation of protein kinase B activity	Biological Process	GO:0032148	0.000158
negative regulation of gene silencing	Biological Process	GO:0060969	0.000158
cellular response to lipoprotein particle stimulus	Biological Process	GO:0071402	0.000158
negative regulation of inflammatory response	Biological Process	GO:0050728	0.00016
skeletal muscle organ development	Biological Process	GO:0060538	0.00016
granulocyte migration	Biological Process	GO:0097530	0.000165
adherens junction organization	Biological Process	GO:0034332	0.000166
neuron fate commitment	Biological Process	GO:0048663	0.000166
regulation of actin filament organization	Biological Process	GO:0110053	0.000169
membrane protein intracellular domain proteolysis	Biological Process	GO:0031293	0.000172
regulation of fatty acid beta-oxidation	Biological Process	GO:0031998	0.000172

regulation of filopodium assembly	Biological Process	GO:0051489	0.000173
ventricular septum morphogenesis	Biological Process	GO:0060412	0.000173
leukocyte adhesion to vascular endothelial cell	Biological Process	GO:0061756	0.000173
necroptotic process	Biological Process	GO:0070266	0.000173
positive regulation of ion transmembrane transport	Biological Process	GO:0034767	0.000178
negative regulation of peptidase activity	Biological Process	GO:0010466	0.000179
negative regulation of gene expression, epigenetic	Biological Process	GO:0045814	0.000182
regulation of heterotypic cell-cell adhesion	Biological Process	GO:0034114	0.000186
T-helper 17 cell differentiation	Biological Process	GO:0072539	0.000186
negative regulation of ATP metabolic process	Biological Process	GO:1903579	0.000186
regulation of muscle contraction	Biological Process	GO:0006937	0.000187
ventricular cardiac muscle tissue development	Biological Process	GO:0003229	0.000192
negative regulation of intracellular steroid hormone receptor signaling pathway	Biological Process	GO:0033144	0.000195
aorta morphogenesis	Biological Process	GO:0035909	0.000195
type 2 immune response	Biological Process	GO:0042092	0.000195
regulation of glycogen metabolic process	Biological Process	GO:0070873	0.000195
bone cell development	Biological Process	GO:0098751	0.000195
polysaccharide biosynthetic process	Biological Process	GO:0000271	0.000199
negative regulation of leukocyte proliferation	Biological Process	GO:0070664	0.000199
negative regulation of protein binding	Biological Process	GO:0032091	0.000201
protein deacylation	Biological Process	GO:0035601	0.000201
regulation of synaptic plasticity	Biological Process	GO:0048167	0.000205
negative regulation of gliogenesis	Biological Process	GO:0014014	0.000206
response to progesterone	Biological Process	GO:0032570	0.000206
embryonic cranial skeleton morphogenesis	Biological Process	GO:0048701	0.000206
regulation of G0 to G1 transition	Biological Process	GO:0070316	0.000206
striated muscle cell development	Biological Process	GO:0055002	0.000217
negative regulation of myeloid cell differentiation	Biological Process	GO:0045638	0.000217
mast cell chemotaxis	Biological Process	GO:0002551	0.000217
establishment or maintenance of actin cytoskeleton polarity	Biological Process	GO:0030950	0.000217
endothelial cell activation	Biological Process	GO:0042118	0.000217
positive regulation of chemokine biosynthetic process	Biological Process	GO:0045080	0.000217
leukocyte aggregation	Biological Process	GO:0070486	0.000217
mast cell migration	Biological Process	GO:0097531	0.000217
regulation of apoptotic process involved in morphogenesis	Biological Process	GO:1902337	0.000217
regulation of chemokine (C-X-C motif) ligand 2 production	Biological Process	GO:2000341	0.000217

macromolecule deacylation	Biological Process	GO:0098732	0.000222
DNA damage response, signal transduction by p53 class mediator resulting in cell cycle arrest	Biological Process	GO:0006977	0.000222
alpha-beta T cell lineage commitment	Biological Process	GO:0002363	0.000236
chronic inflammatory response	Biological Process	GO:0002544	0.000236
lymph vessel morphogenesis	Biological Process	GO:0036303	0.000236
cell migration involved in heart development	Biological Process	GO:0060973	0.000236
kidney mesenchyme development	Biological Process	GO:0072074	0.000236
negative regulation of release of cytochrome c from mitochondria	Biological Process	GO:0090201	0.000236
cellular response to reactive nitrogen species	Biological Process	GO:1902170	0.000236
vascular associated smooth muscle cell apoptotic process	Biological Process	GO:1905288	0.000236
regulation of vascular associated smooth muscle cell apoptotic process	Biological Process	GO:1905459	0.000236
positive regulation of G2/M transition of mitotic cell cycle	Biological Process	GO:0010971	0.000236
regulation of lymphocyte chemotaxis	Biological Process	GO:1901623	0.000236
regulation of fatty acid transport	Biological Process	GO:2000191	0.000236
vascular endothelial growth factor production	Biological Process	GO:0010573	0.000236
positive regulation of interleukin-10 production	Biological Process	GO:0032733	0.000236
smooth muscle cell apoptotic process	Biological Process	GO:0034390	0.000236
regulation of smooth muscle cell apoptotic process	Biological Process	GO:0034391	0.000236
cellular response to estradiol stimulus	Biological Process	GO:0071392	0.000236
positive regulation of CD4-positive, alpha-beta T cell activation	Biological Process	GO:2000516	0.000236
glycogen biosynthetic process	Biological Process	GO:0005978	0.000241
glucan biosynthetic process	Biological Process	GO:0009250	0.000241
ovulation cycle process	Biological Process	GO:0022602	0.000241
negative regulation of type I interferon production	Biological Process	GO:0032480	0.000241
apoptotic cell clearance	Biological Process	GO:0043277	0.000241
regulation of interleukin-1 beta secretion	Biological Process	GO:0050706	0.000241
proteasome-mediated ubiquitin-dependent protein catabolic process	Biological Process	GO:0043161	0.000243
plasma membrane organization	Biological Process	GO:0007009	0.000243
positive regulation of macroautophagy	Biological Process	GO:0016239	0.000244
regulation of mitotic sister chromatid segregation	Biological Process	GO:0033047	0.000244
negative regulation of chromosome organization	Biological Process	GO:2001251	0.000246
neural crest cell migration	Biological Process	GO:0001755	0.000255
columnar/cuboidal epithelial cell development	Biological Process	GO:0002066	0.000255
regulation of mitotic sister chromatid separation	Biological Process	GO:0010965	0.000255
negative regulation of lipid biosynthetic process	Biological Process	GO:0051055	0.000255

positive regulation of response to cytokine stimulus	Biological Process	GO:0060760	0.000255
response to heat	Biological Process	GO:0009408	0.000266
T cell mediated immunity	Biological Process	GO:0002456	0.000268
regulation of interspecies interactions between organisms	Biological Process	GO:0043903	0.000274
cardiac muscle tissue morphogenesis	Biological Process	GO:0055008	0.000279
lipid modification	Biological Process	GO:0030258	0.000279
extracellular matrix disassembly	Biological Process	GO:0022617	0.000279
regulation of telomere maintenance	Biological Process	GO:0032204	0.000279
homotypic cell-cell adhesion	Biological Process	GO:0034109	0.000279
telomere maintenance	Biological Process	GO:0000723	0.000279
regulation of erythrocyte differentiation	Biological Process	GO:0045646	0.000284
positive regulation of biomineral tissue development	Biological Process	GO:0070169	0.000284
positive regulation of biomineralization	Biological Process	GO:0110151	0.000284
T cell homeostasis	Biological Process	GO:0043029	0.000285
positive regulation of fatty acid metabolic process	Biological Process	GO:0045923	0.000285
cellular response to cadmium ion	Biological Process	GO:0071276	0.000285
dopaminergic neuron differentiation	Biological Process	GO:0071542	0.000285
response to calcium ion	Biological Process	GO:0051592	0.000288
response to organophosphorus	Biological Process	GO:0046683	0.00029
regulation of vasoconstriction	Biological Process	GO:0019229	0.000293
intracellular estrogen receptor signaling pathway	Biological Process	GO:0030520	0.000293
negative regulation of glial cell differentiation	Biological Process	GO:0045686	0.000296
protein localization to chromatin	Biological Process	GO:0071168	0.000296
negative regulation of anion transport	Biological Process	GO:1903792	0.000296
regulation of lymphocyte mediated immunity	Biological Process	GO:0002706	0.000312
negative regulation of ossification	Biological Process	GO:0030279	0.000312
muscle tissue morphogenesis	Biological Process	GO:0060415	0.000312
lamellipodium organization	Biological Process	GO:0097581	0.000312
positive regulation of lipid localization	Biological Process	GO:1905954	0.000312
cytokine secretion involved in immune response	Biological Process	GO:0002374	0.000314
positive regulation of tumor necrosis factor biosynthetic process	Biological Process	GO:0042535	0.000314
CD4-positive or CD8-positive, alpha-beta T cell lineage commitment	Biological Process	GO:0043369	0.000314
negative regulation of CD4-positive, alpha-beta T cell differentiation	Biological Process	GO:0043371	0.000314
smooth muscle tissue development	Biological Process	GO:0048745	0.000314
branch elongation of an epithelium	Biological Process	GO:0060602	0.000314
chemokine secretion	Biological Process	GO:0090195	0.000314

positive regulation of amyloid precursor protein catabolic process	Biological Process	GO:1902993	0.000314
regulation of microglial cell activation	Biological Process	GO:1903978	0.000314
negative regulation of translation	Biological Process	GO:0017148	0.000318
regulation of GTPase activity	Biological Process	GO:0043087	0.000321
regulation of DNA replication	Biological Process	GO:0006275	0.000322
negative regulation of response to biotic stimulus	Biological Process	GO:0002832	0.000324
negative regulation of macrophage derived foam cell differentiation	Biological Process	GO:0010745	0.000324
regulation of histone phosphorylation	Biological Process	GO:0033127	0.000324
white fat cell differentiation	Biological Process	GO:0050872	0.000324
detection of mechanical stimulus involved in sensory perception of pain	Biological Process	GO:0050966	0.000324
eyelid development in camera-type eye	Biological Process	GO:0061029	0.000324
nucleotide-binding oligomerization domain containing 2 signaling pathway	Biological Process	GO:0070431	0.000324
replicative senescence	Biological Process	GO:0090399	0.000324
inflammatory response to wounding	Biological Process	GO:0090594	0.000324
regulation of tau-protein kinase activity	Biological Process	GO:1902947	0.000324
negative regulation of myeloid leukocyte differentiation	Biological Process	GO:0002762	0.000329
ventricular cardiac muscle tissue morphogenesis	Biological Process	GO:0055010	0.000329
face development	Biological Process	GO:0060324	0.000329
histone H3-K9 modification	Biological Process	GO:0061647	0.000329
vascular endothelial cell proliferation	Biological Process	GO:0101023	0.000329
positive regulation of phospholipid metabolic process	Biological Process	GO:1903727	0.000329
regulation of vascular endothelial cell proliferation	Biological Process	GO:1905562	0.000329
negative regulation of immune response	Biological Process	GO:0050777	0.000332
positive regulation of interleukin-1 production	Biological Process	GO:0032732	0.000332
regulation of organic acid transport	Biological Process	GO:0032890	0.000332
platelet aggregation	Biological Process	GO:0070527	0.000332
regulation of peptidyl-lysine acetylation	Biological Process	GO:2000756	0.000332
positive regulation of leukocyte mediated immunity	Biological Process	GO:0002705	0.000337
female gamete generation	Biological Process	GO:0007292	0.000337
physiological muscle hypertrophy	Biological Process	GO:0003298	0.000338
physiological cardiac muscle hypertrophy	Biological Process	GO:0003301	0.000338
positive regulation of glucose import	Biological Process	GO:0046326	0.000338
cell growth involved in cardiac muscle cell development	Biological Process	GO:0061049	0.000338
apoptotic process involved in development	Biological Process	GO:1902742	0.000338
regulation of cation channel activity	Biological Process	GO:2001257	0.000346
positive regulation of calcium ion transport	Biological Process	GO:0051928	0.000365

regulation of transcription involved in G1/S transition of mitotic cell cycle	Biological Process	GO:0000083	0.000366
regulation of DNA methylation	Biological Process	GO:0044030	0.000366
epidermis morphogenesis	Biological Process	GO:0048730	0.000366
response to cholesterol	Biological Process	GO:0070723	0.000366
T-helper 17 type immune response	Biological Process	GO:0072538	0.000366
mitotic sister chromatid separation	Biological Process	GO:0051306	0.000381
glial cell migration	Biological Process	GO:0008347	0.000383
positive regulation of interleukin-8 production	Biological Process	GO:0032757	0.000383
negative regulation of I-kappaB kinase/NF-kappaB signaling	Biological Process	GO:0043124	0.000383
cellular response to interleukin-12	Biological Process	GO:0071349	0.000383
smooth muscle contraction	Biological Process	GO:0006939	0.000384
primary neural tube formation	Biological Process	GO:0014020	0.000393
regulation of mitochondrial membrane potential	Biological Process	GO:0051881	0.000395
negative regulation of tumor necrosis factor superfamily cytokine production	Biological Process	GO:1903556	0.000395
cellular process involved in reproduction in multicellular organism	Biological Process	GO:0022412	0.000397
embryonic epithelial tube formation	Biological Process	GO:0001838	0.000397
regulation of steroid metabolic process	Biological Process	GO:0019218	0.000397
positive regulation of muscle cell apoptotic process	Biological Process	GO:0010661	0.000405
positive regulation of bone mineralization	Biological Process	GO:0030501	0.000405
positive regulation of histone methylation	Biological Process	GO:0031062	0.000405
response to testosterone	Biological Process	GO:0033574	0.000405
regulation of vascular permeability	Biological Process	GO:0043114	0.000405
regulation of intrinsic apoptotic signaling pathway in response to DNA damage	Biological Process	GO:1902229	0.000405
pulmonary valve development	Biological Process	GO:0003177	0.00041
atrial septum development	Biological Process	GO:0003283	0.00041
negative regulation of chemokine production	Biological Process	GO:0032682	0.00041
negative regulation of organic acid transport	Biological Process	GO:0032891	0.00041
sympathetic nervous system development	Biological Process	GO:0048485	0.00041
glomerular epithelium development	Biological Process	GO:0072010	0.00041
regulation of establishment of cell polarity	Biological Process	GO:2000114	0.00041
regulation of fibroblast apoptotic process	Biological Process	GO:2000269	0.00041
receptor internalization	Biological Process	GO:0031623	0.000419
central nervous system neuron differentiation	Biological Process	GO:0021953	0.000422
lipid transport	Biological Process	GO:0006869	0.000426
regulation of protein dephosphorylation	Biological Process	GO:0035304	0.000427
organic hydroxy compound transport	Biological Process	GO:0015850	0.000428

nuclear chromosome segregation	Biological Process	GO:0098813	0.000428
regulation of phagocytosis	Biological Process	GO:0050764	0.000431
positive regulation of lipid transport	Biological Process	GO:0032370	0.000431
heterotypic cell-cell adhesion	Biological Process	GO:0034113	0.000431
interleukin-1 secretion	Biological Process	GO:0050701	0.000431
cellular ketone metabolic process	Biological Process	GO:0042180	0.000432
nuclear transport	Biological Process	GO:0051169	0.000435
negative regulation of mononuclear cell proliferation	Biological Process	GO:0032945	0.000441
negative regulation of lymphocyte proliferation	Biological Process	GO:0050672	0.000441
regulation of protein acetylation	Biological Process	GO:1901983	0.000441
response to interleukin-12	Biological Process	GO:0070671	0.000442
protein insertion into mitochondrial membrane involved in apoptotic signaling pathway	Biological Process	GO:0001844	0.000448
regulation of type 2 immune response	Biological Process	GO:0002828	0.000448
positive regulation of CD4-positive, alpha-beta T cell differentiation	Biological Process	GO:0043372	0.000448
positive regulation of filopodium assembly	Biological Process	GO:0051491	0.000448
negative regulation of biomineral tissue development	Biological Process	GO:0070168	0.000448
positive regulation of protein targeting to membrane	Biological Process	GO:0090314	0.000448
negative regulation of biomineralization	Biological Process	GO:0110150	0.000448
positive regulation of cell cycle G2/M phase transition	Biological Process	GO:1902751	0.000448
beta-catenin-TCF complex assembly	Biological Process	GO:1904837	0.000448
regulation of Rho protein signal transduction	Biological Process	GO:0035023	0.000459
heart valve formation	Biological Process	GO:0003188	0.000465
genitalia morphogenesis	Biological Process	GO:0035112	0.000465
angiotensin-activated signaling pathway	Biological Process	GO:0038166	0.000465
regulation of MHC class II biosynthetic process	Biological Process	GO:0045346	0.000465
positive regulation of regulatory T cell differentiation	Biological Process	GO:0045591	0.000465
regulation of keratinocyte migration	Biological Process	GO:0051547	0.000465
response to redox state	Biological Process	GO:0051775	0.000465
regulation of apoptotic process involved in development	Biological Process	GO:1904748	0.000465
cellular carbohydrate biosynthetic process	Biological Process	GO:0034637	0.000468
negative regulation of DNA replication	Biological Process	GO:0008156	0.000476
fatty acid metabolic process	Biological Process	GO:0006631	0.000481
cytoplasmic pattern recognition receptor signaling pathway	Biological Process	GO:0002753	0.000489
glycogen metabolic process	Biological Process	GO:0005977	0.000492
regulation of interleukin-8 production	Biological Process	GO:0032677	0.000492
negative regulation of ERK1 and ERK2 cascade	Biological Process	GO:0070373	0.000492

positive regulation of dendrite development	Biological Process	GO:1900006	0.000492
positive regulation of protein localization to nucleus	Biological Process	GO:1900182	0.000492
negative regulation of protein secretion	Biological Process	GO:0050709	0.000494
positive regulation of cell-matrix adhesion	Biological Process	GO:0001954	0.000509
chromatin silencing	Biological Process	GO:0006342	0.000509
regulation of microtubule cytoskeleton organization	Biological Process	GO:0070507	0.00051
cytoskeleton-dependent cytokinesis	Biological Process	GO:0061640	0.000518
energy reserve metabolic process	Biological Process	GO:0006112	0.000519
regulation of protein sumoylation	Biological Process	GO:0033233	0.000528
vitamin D metabolic process	Biological Process	GO:0042359	0.000528
response to leptin	Biological Process	GO:0044321	0.000528
positive regulation of T-helper cell differentiation	Biological Process	GO:0045624	0.000528
regulation of macrophage differentiation	Biological Process	GO:0045649	0.000528
negative regulation of phagocytosis	Biological Process	GO:0050765	0.000528
polysaccharide metabolic process	Biological Process	GO:0005976	0.000541
histone lysine methylation	Biological Process	GO:0034968	0.000541
modulation by virus of host process	Biological Process	GO:0019048	0.000546
response to prostaglandin	Biological Process	GO:0034694	0.000546
wound healing, spreading of cells	Biological Process	GO:0044319	0.000546
positive regulation of epidermal growth factor receptor signaling pathway	Biological Process	GO:0045742	0.000546
regulation of astrocyte differentiation	Biological Process	GO:0048710	0.000546
negative regulation of calcium-mediated signaling	Biological Process	GO:0050849	0.000546
epiboly involved in wound healing	Biological Process	GO:0090505	0.000546
positive regulation of T cell migration	Biological Process	GO:2000406	0.000546
cellular glucan metabolic process	Biological Process	GO:0006073	0.000549
glucan metabolic process	Biological Process	GO:0044042	0.000549
response to cadmium ion	Biological Process	GO:0046686	0.000551
negative regulation of hormone secretion	Biological Process	GO:0046888	0.000551
regulation of keratinocyte differentiation	Biological Process	GO:0045616	0.00056
negative regulation of G0 to G1 transition	Biological Process	GO:0070317	0.00056
extracellular matrix assembly	Biological Process	GO:0085029	0.00056
regulation of heart contraction	Biological Process	GO:0008016	0.000563
organelle disassembly	Biological Process	GO:1903008	0.000565
muscle organ morphogenesis	Biological Process	GO:0048644	0.000571
execution phase of apoptosis	Biological Process	GO:0097194	0.000571
negative regulation of mitotic nuclear division	Biological Process	GO:0045839	0.000584

regulation of ubiquitin-protein transferase activity	Biological Process	GO:0051438	0.000584
positive regulation of neural precursor cell proliferation	Biological Process	GO:2000179	0.000584
regulation of purine nucleotide metabolic process	Biological Process	GO:1900542	0.000587
protein acetylation	Biological Process	GO:0006473	0.000594
sister chromatid segregation	Biological Process	GO:0000819	0.000616
lamellipodium assembly	Biological Process	GO:0030032	0.000625
positive regulation of mitochondrion organization	Biological Process	GO:0010822	0.000642
regulation of protein localization to nucleus	Biological Process	GO:1900180	0.000642
sequestering of triglyceride	Biological Process	GO:0030730	0.000653
long-chain fatty acid import into cell	Biological Process	GO:0044539	0.000653
MHC class II biosynthetic process	Biological Process	GO:0045342	0.000653
metanephric mesenchyme development	Biological Process	GO:0072075	0.000653
positive regulation of chemokine secretion	Biological Process	GO:0090197	0.000653
lipid import into cell	Biological Process	GO:0140354	0.000653
cell junction disassembly	Biological Process	GO:0150146	0.000653
regulation of CD8-positive, alpha-beta T cell activation	Biological Process	GO:2001185	0.000653
dendritic cell differentiation	Biological Process	GO:0097028	0.00066
regulation of response to reactive oxygen species	Biological Process	GO:1901031	0.00066
long-term memory	Biological Process	GO:0007616	0.000662
regulation of regulatory T cell differentiation	Biological Process	GO:0045589	0.000662
positive regulation of vasoconstriction	Biological Process	GO:0045907	0.000662
response to lipoprotein particle	Biological Process	GO:0055094	0.000662
face morphogenesis	Biological Process	GO:0060325	0.000662
heart trabecula morphogenesis	Biological Process	GO:0061384	0.000662
epiboly	Biological Process	GO:0090504	0.000662
neutrophil mediated immunity	Biological Process	GO:0002446	0.000663
telomere organization	Biological Process	GO:0032200	0.000668
negative regulation of mitochondrion organization	Biological Process	GO:0010823	0.000668
regulation of interleukin-1 secretion	Biological Process	GO:0050704	0.000668
neuroepithelial cell differentiation	Biological Process	GO:0060563	0.000668
nucleotide-excision repair, DNA damage recognition	Biological Process	GO:0000715	0.000669
response to prostaglandin E	Biological Process	GO:0034695	0.000669
positive regulation of glycolytic process	Biological Process	GO:0045821	0.000669
positive regulation of oligodendrocyte differentiation	Biological Process	GO:0048714	0.000669
regulation of membrane protein ectodomain proteolysis	Biological Process	GO:0051043	0.000669
branching involved in salivary gland morphogenesis	Biological Process	GO:0060445	0.000669

embryonic heart tube development	Biological Process	GO:0035050	0.000679
response to axon injury	Biological Process	GO:0048678	0.000679
regulation of nucleotide metabolic process	Biological Process	GO:0006140	0.000691
regulation of smooth muscle contraction	Biological Process	GO:0006940	7.00E-04
toll-like receptor signaling pathway	Biological Process	GO:0002224	0.000712
sensory perception of pain	Biological Process	GO:0019233	0.000741
epithelial tube formation	Biological Process	GO:0072175	0.000742
DNA replication	Biological Process	GO:0006260	0.000749
regulation of viral process	Biological Process	GO:0050792	0.000753
central nervous system neuron development	Biological Process	GO:0021954	0.000759
metaphase/anaphase transition of mitotic cell cycle	Biological Process	GO:0007091	0.000766
collagen fibril organization	Biological Process	GO:0030199	0.000766
cell differentiation involved in kidney development	Biological Process	GO:0061005	0.000766
membrane depolarization	Biological Process	GO:0051899	0.000766
calcineurin-NFAT signaling cascade	Biological Process	GO:0033173	0.000768
negative regulation of Notch signaling pathway	Biological Process	GO:0045746	0.000768
neutral lipid biosynthetic process	Biological Process	GO:0046460	0.000768
acylglycerol biosynthetic process	Biological Process	GO:0046463	0.000768
response to sterol	Biological Process	GO:0036314	0.000797
alpha-beta T cell proliferation	Biological Process	GO:0046633	0.000797
positive regulation of ubiquitin-protein transferase activity	Biological Process	GO:0051443	0.000797
positive regulation of cartilage development	Biological Process	GO:0061036	0.000797
positive regulation of ERBB signaling pathway	Biological Process	GO:1901186	0.000797
negative regulation of signal transduction by p53 class mediator	Biological Process	GO:1901797	0.000797
neural tube formation	Biological Process	GO:0001841	0.000807
positive regulation of adaptive immune response	Biological Process	GO:0002821	0.000807
nucleocytoplasmic transport	Biological Process	GO:0006913	0.000813
meiosis I cell cycle process	Biological Process	GO:0061982	0.000815
negative regulation of peptide secretion	Biological Process	GO:0002792	0.000821
carbohydrate transport	Biological Process	GO:0008643	0.000821
negative regulation of blood vessel endothelial cell migration	Biological Process	GO:0043537	0.000841
vitamin biosynthetic process	Biological Process	GO:0009110	0.000845
regulation of establishment or maintenance of cell polarity	Biological Process	GO:0032878	0.000845
positive regulation of endothelial cell differentiation	Biological Process	GO:0045603	0.000845
negative regulation of receptor signaling pathway via JAK-STAT	Biological Process	GO:0046426	0.000845
positive regulation of lipid catabolic process	Biological Process	GO:0050996	0.000845

cochlea morphogenesis	Biological Process	GO:0090103	0.000845
regulation of endothelial cell chemotaxis	Biological Process	GO:2001026	0.000845
inositol phosphate-mediated signaling	Biological Process	GO:0048016	0.000872
positive regulation of protein tyrosine kinase activity	Biological Process	GO:0061098	0.000872
spinal cord development	Biological Process	GO:0021510	0.00088
regulation of response to drug	Biological Process	GO:2001023	0.00088
cellular response to starvation	Biological Process	GO:0009267	0.000881
positive regulation of DNA biosynthetic process	Biological Process	GO:2000573	0.000884
epithelial cell maturation	Biological Process	GO:0002070	0.000885
dentate gyrus development	Biological Process	GO:0021542	0.000885
establishment or maintenance of cytoskeleton polarity	Biological Process	GO:0030952	0.000885
myelin maintenance	Biological Process	GO:0043217	0.000885
positive regulation of histone H3-K4 methylation	Biological Process	GO:0051571	0.000885
regulation of brown fat cell differentiation	Biological Process	GO:0090335	0.000885
positive regulation of glucose transmembrane transport	Biological Process	GO:0010828	0.000888
regulation of collagen biosynthetic process	Biological Process	GO:0032965	0.000888
positive regulation of tissue remodeling	Biological Process	GO:0034105	0.000888
epithelial cell differentiation involved in kidney development	Biological Process	GO:0035850	0.000888
detection of mechanical stimulus	Biological Process	GO:0050982	0.000888
regulation of T cell migration	Biological Process	GO:2000404	0.000888
developmental induction	Biological Process	GO:0031128	0.000949
tumor necrosis factor biosynthetic process	Biological Process	GO:0042533	0.000949
regulation of tumor necrosis factor biosynthetic process	Biological Process	GO:0042534	0.000949
regulatory T cell differentiation	Biological Process	GO:0045066	0.000949
negative regulation of exocytosis	Biological Process	GO:0045920	0.000949
regulation of calcineurin-NFAT signaling cascade	Biological Process	GO:0070884	0.000949
regulation of calcineurin-mediated signaling	Biological Process	GO:0106056	0.000949
positive regulation of signaling receptor activity	Biological Process	GO:2000273	0.000949
immune response-activating cell surface receptor signaling pathway	Biological Process	GO:0002429	0.000971
immune response-activating signal transduction	Biological Process	GO:0002757	0.000971
cell adhesion mediated by integrin	Biological Process	GO:0033627	0.000988
metaphase/anaphase transition of cell cycle	Biological Process	GO:0044784	0.000988
internal protein amino acid acetylation	Biological Process	GO:0006475	0.001
lipid homeostasis	Biological Process	GO:0055088	0.00101
regulation of DNA repair	Biological Process	GO:0006282	0.00103
regulation of proteasomal ubiquitin-dependent protein catabolic process	Biological Process	GO:0032434	0.00103

intrinsic apoptotic signaling pathway in response to DNA damage by p53 class mediator	Biological Process	GO:0042771	0.00103
autonomic nervous system development	Biological Process	GO:0048483	0.00103
protein insertion into mitochondrial membrane	Biological Process	GO:0051204	0.00103
regulation of lamellipodium organization	Biological Process	GO:1902743	0.00103
lymph vessel development	Biological Process	GO:0001945	0.00105
glandular epithelial cell development	Biological Process	GO:0002068	0.00105
tolerance induction	Biological Process	GO:0002507	0.00105
hair follicle morphogenesis	Biological Process	GO:0031069	0.00105
positive regulation of protein oligomerization	Biological Process	GO:0032461	0.00105
positive regulation of focal adhesion assembly	Biological Process	GO:0051894	0.00105
regulation of response to interferon-gamma	Biological Process	GO:0060330	0.00105
regulation of interferon-gamma-mediated signaling pathway	Biological Process	GO:0060334	0.00105
regulation of cell growth involved in cardiac muscle cell development	Biological Process	GO:0061050	0.00105
negative regulation of regulated secretory pathway	Biological Process	GO:1903306	0.00105
positive regulation of endothelial cell apoptotic process	Biological Process	GO:2000353	0.00105
locomotory behavior	Biological Process	GO:0007626	0.00105
regulation of nervous system process	Biological Process	GO:0031644	0.00107
regulation of proteasomal protein catabolic process	Biological Process	GO:0061136	0.0011
cellular response to retinoic acid	Biological Process	GO:0071300	0.0011
regulation of steroid biosynthetic process	Biological Process	GO:0050810	0.0011
regulation of insulin secretion involved in cellular response to glucose stimulus	Biological Process	GO:0061178	0.00112
interleukin-8 production	Biological Process	GO:0032637	0.00113
regulation of cardiac muscle contraction	Biological Process	GO:0055117	0.00113
protein homooligomerization	Biological Process	GO:0051260	0.00114
pulmonary valve morphogenesis	Biological Process	GO:0003184	0.00118
response to UV-B	Biological Process	GO:0010224	0.00118
female genitalia development	Biological Process	GO:0030540	0.00118
regulation of mammary gland epithelial cell proliferation	Biological Process	GO:0033599	0.00118
cellular response to hepatocyte growth factor stimulus	Biological Process	GO:0035729	0.00118
retina vasculature development in camera-type eye	Biological Process	GO:0061298	0.00118
renal filtration cell differentiation	Biological Process	GO:0061318	0.00118
thymocyte apoptotic process	Biological Process	GO:0070242	0.00118
glomerular visceral epithelial cell differentiation	Biological Process	GO:0072112	0.00118
regulation of glial cell migration	Biological Process	GO:1903975	0.00118
negative regulation of morphogenesis of an epithelium	Biological Process	GO:1905331	0.00118

regulation of cysteine-type endopeptidase activity involved in apoptotic signaling pathway	Biological Process	GO:2001267	0.00118
oocyte development	Biological Process	GO:0048599	0.00119
labyrinthine layer development	Biological Process	GO:0060711	0.00119
calcineurin-mediated signaling	Biological Process	GO:0097720	0.00119
cholesterol homeostasis	Biological Process	GO:0042632	0.0012
response to leukemia inhibitory factor	Biological Process	GO:1990823	0.0012
cellular response to leukemia inhibitory factor	Biological Process	GO:1990830	0.0012
neutrophil activation	Biological Process	GO:0042119	0.00121
negative regulation of tumor necrosis factor production	Biological Process	GO:0032720	0.00122
histone methylation	Biological Process	GO:0016571	0.00123
regulation of neurotransmitter transport	Biological Process	GO:0051588	0.00123
foregut morphogenesis	Biological Process	GO:0007440	0.00123
positive regulation of fatty acid beta-oxidation	Biological Process	GO:0032000	0.00123
response to cobalt ion	Biological Process	GO:0032025	0.00123
positive regulation of MHC class II biosynthetic process	Biological Process	GO:0045348	0.00123
negative regulation of cell size	Biological Process	GO:0045792	0.00123
prostatic bud formation	Biological Process	GO:0060513	0.00123
regulation of epithelial cell proliferation involved in prostate gland development	Biological Process	GO:0060768	0.00123
lung secretory cell differentiation	Biological Process	GO:0061140	0.00123
chemokine (C-C motif) ligand 5 production	Biological Process	GO:0071609	0.00123
regulation of glomerular mesangial cell proliferation	Biological Process	GO:0072124	0.00123
cell proliferation involved in metanephros development	Biological Process	GO:0072203	0.00123
interleukin-12 secretion	Biological Process	GO:0072610	0.00123
negative regulation of mesenchymal cell apoptotic process	Biological Process	GO:2001054	0.00123
positive regulation of dephosphorylation	Biological Process	GO:0035306	0.00125
regulation of macrophage chemotaxis	Biological Process	GO:0010758	0.00128
positive regulation of protein autophosphorylation	Biological Process	GO:0031954	0.00128
positive regulation of collagen biosynthetic process	Biological Process	GO:0032967	0.00128
regulation of long-term neuronal synaptic plasticity	Biological Process	GO:0048169	0.00128
embryonic placenta morphogenesis	Biological Process	GO:0060669	0.00128
cell differentiation involved in embryonic placenta development	Biological Process	GO:0060706	0.00128
positive regulation of cell-substrate junction organization	Biological Process	GO:0150117	0.00128
regulation of microtubule-based process	Biological Process	GO:0032886	0.0013
response to cAMP	Biological Process	GO:0051591	0.0013
sterol homeostasis	Biological Process	GO:0055092	0.0013

positive regulation of response to DNA damage stimulus	Biological Process	GO:2001022	0.0013
response to endoplasmic reticulum stress	Biological Process	GO:0034976	0.00131
regulation of vascular endothelial growth factor receptor signaling pathway	Biological Process	GO:0030947	0.00132
hindlimb morphogenesis	Biological Process	GO:0035137	0.00132
regulation of protein targeting to membrane	Biological Process	GO:0090313	0.00132
regulation of NMDA receptor activity	Biological Process	GO:2000310	0.00132
cellular polysaccharide biosynthetic process	Biological Process	GO:0033692	0.00135
interleukin-12-mediated signaling pathway	Biological Process	GO:0035722	0.00135
mast cell degranulation	Biological Process	GO:0043303	0.00135
establishment of protein localization to mitochondrial membrane	Biological Process	GO:0090151	0.00135
positive regulation of lipid biosynthetic process	Biological Process	GO:0046889	0.00136
heterochromatin organization	Biological Process	GO:0070828	0.00136
negative regulation of blood vessel diameter	Biological Process	GO:0097756	0.00136
embryonic skeletal system development	Biological Process	GO:0048706	0.00138
histone acetylation	Biological Process	GO:0016573	0.00139
negative regulation of T cell proliferation	Biological Process	GO:0042130	0.00141
filopodium assembly	Biological Process	GO:0046847	0.00141
ATP metabolic process	Biological Process	GO:0046034	0.00142
negative regulation of cell projection organization	Biological Process	GO:0031345	0.00147
regulation of DNA-templated transcription in response to stress	Biological Process	GO:0043620	0.00149
axis specification	Biological Process	GO:0009798	0.0015
production of molecular mediator involved in inflammatory response	Biological Process	GO:0002532	0.0015
negative regulation of synaptic transmission	Biological Process	GO:0050805	0.0015
regulation of gene expression by genetic imprinting	Biological Process	GO:0006349	0.00152
activation of NF-kappaB-inducing kinase activity	Biological Process	GO:0007250	0.00152
I-kappaB phosphorylation	Biological Process	GO:0007252	0.00152
modulation by virus of host cellular process	Biological Process	GO:0019054	0.00152
positive regulation of histone deacetylation	Biological Process	GO:0031065	0.00152
osteoclast development	Biological Process	GO:0036035	0.00152
cellular response to leptin stimulus	Biological Process	GO:0044320	0.00152
positive regulation of lymphocyte apoptotic process	Biological Process	GO:0070230	0.00152
glomerular epithelial cell differentiation	Biological Process	GO:0072311	0.00152
regulation of chemokine secretion	Biological Process	GO:0090196	0.00152
positive regulation of neuroinflammatory response	Biological Process	GO:0150078	0.00152
negative regulation of purine nucleotide metabolic process	Biological Process	GO:1900543	0.00152
regulation of T-helper 17 cell differentiation	Biological Process	GO:2000319	0.00152

glycolytic process	Biological Process	GO:0006096	0.00154
cerebral cortex development	Biological Process	GO:0021987	0.00154
mast cell activation involved in immune response	Biological Process	GO:0002279	0.00154
regulation of interferon-beta production	Biological Process	GO:0032648	0.00154
negative regulation of carbohydrate metabolic process	Biological Process	GO:0045912	0.00154
positive regulation of muscle contraction	Biological Process	GO:0045933	0.00154
regulation of telomerase activity	Biological Process	GO:0051972	0.00154
positive regulation of cardiac muscle tissue growth	Biological Process	GO:0055023	0.00154
negative regulation of cardiac muscle hypertrophy	Biological Process	GO:0010614	0.00154
response to vitamin D	Biological Process	GO:0033280	0.00154
respiratory burst	Biological Process	GO:0045730	0.00154
regulation of cofactor metabolic process	Biological Process	GO:0051193	0.00154
head morphogenesis	Biological Process	GO:0060323	0.00154
postsynaptic signal transduction	Biological Process	GO:0098926	0.00154
negative regulation of reactive oxygen species biosynthetic process	Biological Process	GO:1903427	0.00154
inactivation of MAPK activity	Biological Process	GO:0000188	0.00154
outflow tract septum morphogenesis	Biological Process	GO:0003148	0.00154
substrate-dependent cell migration	Biological Process	GO:0006929	0.00154
positive regulation of collagen metabolic process	Biological Process	GO:0010714	0.00154
enteroendocrine cell differentiation	Biological Process	GO:0035883	0.00154
response to immobilization stress	Biological Process	GO:0035902	0.00154
positive regulation of macrophage activation	Biological Process	GO:0043032	0.00154
negative regulation of osteoclast differentiation	Biological Process	GO:0045671	0.00154
genetic imprinting	Biological Process	GO:0071514	0.00154
regulation of oxidative stress-induced intrinsic apoptotic signaling pathway	Biological Process	GO:1902175	0.00154
negative regulation of nuclear division	Biological Process	GO:0051784	0.00156
positive regulation of cell cycle G1/S phase transition	Biological Process	GO:1902808	0.00156
dendrite morphogenesis	Biological Process	GO:0048813	0.00159
hormone biosynthetic process	Biological Process	GO:0042446	0.00162
regulation of membrane permeability	Biological Process	GO:0090559	0.00162
response to estrogen	Biological Process	GO:0043627	0.00165
ATP generation from ADP	Biological Process	GO:0006757	0.00165
fibroblast growth factor receptor signaling pathway	Biological Process	GO:0008543	0.00165
negative regulation of epithelial cell migration	Biological Process	GO:0010633	0.00165
positive regulation of adaptive immune response based on somatic recombination of immune receptors built from immunoglobulin superfamily domains	Biological Process	GO:0002824	0.00167

glutamate receptor signaling pathway	Biological Process	GO:0007215	0.00167
substrate adhesion-dependent cell spreading	Biological Process	GO:0034446	0.00167
peptidyl-lysine methylation	Biological Process	GO:0018022	0.0017
positive regulation of cation transmembrane transport	Biological Process	GO:1904064	0.0017
mast cell mediated immunity	Biological Process	GO:0002448	0.00175
macromolecule methylation	Biological Process	GO:0043414	0.00176
embryonic digit morphogenesis	Biological Process	GO:0042733	0.00176
positive regulation of wound healing	Biological Process	GO:0090303	0.00176
regulation of carbohydrate catabolic process	Biological Process	GO:0043470	0.00177
DNA recombination	Biological Process	GO:0006310	0.00177
cardioblast proliferation	Biological Process	GO:0003263	0.00177
regulation of cardioblast proliferation	Biological Process	GO:0003264	0.00177
response to gravity	Biological Process	GO:0009629	0.00177
positive regulation of heat generation	Biological Process	GO:0031652	0.00177
detection of molecule of bacterial origin	Biological Process	GO:0032490	0.00177
response to peptidoglycan	Biological Process	GO:0032494	0.00177
positive regulation of intracellular estrogen receptor signaling pathway	Biological Process	GO:0033148	0.00177
Leydig cell differentiation	Biological Process	GO:0033327	0.00177
natural killer cell chemotaxis	Biological Process	GO:0035747	0.00177
regulation of transcription from RNA polymerase II promoter in response to oxidative stress	Biological Process	GO:0043619	0.00177
positive regulation of cell size	Biological Process	GO:0045793	0.00177
prostate glandular acinus development	Biological Process	GO:0060525	0.00177
blood vessel endothelial cell differentiation	Biological Process	GO:0060837	0.00177
cardiac neural crest cell differentiation involved in heart development	Biological Process	GO:0061307	0.00177
cardiac neural crest cell development involved in heart development	Biological Process	GO:0061308	0.00177
histone H4 deacetylation	Biological Process	GO:0070933	0.00177
positive regulation of podosome assembly	Biological Process	GO:0071803	0.00177
loop of Henle development	Biological Process	GO:0072070	0.00177
positive regulation of glial cell migration	Biological Process	GO:1903977	0.00177
positive regulation of cysteine-type endopeptidase activity involved in apoptotic signaling pathway	Biological Process	GO:2001269	0.00177
negative regulation of JNK cascade	Biological Process	GO:0046329	0.00177
macrophage chemotaxis	Biological Process	GO:0048246	0.00177
negative regulation of blood circulation	Biological Process	GO:1903523	0.00177
negative regulation of vascular smooth muscle cell proliferation	Biological Process	GO:1904706	0.00177

regulation of neurotransmitter receptor activity	Biological Process	GO:0099601	0.0018
response to gonadotropin	Biological Process	GO:0034698	0.00186
embryonic hindlimb morphogenesis	Biological Process	GO:0035116	0.00186
positive regulation of lamellipodium organization	Biological Process	GO:1902745	0.00186
regulation of leukocyte adhesion to vascular endothelial cell	Biological Process	GO:1904994	0.00186
internal peptidyl-lysine acetylation	Biological Process	GO:0018393	0.00188
regulation of dephosphorylation	Biological Process	GO:0035303	0.00189
mesodermal cell fate commitment	Biological Process	GO:0001710	0.00192
positive regulation of vascular endothelial growth factor receptor signaling pathway	Biological Process	GO:0030949	0.00192
toll-like receptor 3 signaling pathway	Biological Process	GO:0034138	0.00192
response to hepatocyte growth factor	Biological Process	GO:0035728	0.00192
mRNA transcription by RNA polymerase II	Biological Process	GO:0042789	0.00192
regulation of interleukin-8 biosynthetic process	Biological Process	GO:0045414	0.00192
negative regulation of nucleotide metabolic process	Biological Process	GO:0045980	0.00192
keratinocyte migration	Biological Process	GO:0051546	0.00192
response to nitric oxide	Biological Process	GO:0071731	0.00192
regulation of ATP biosynthetic process	Biological Process	GO:2001169	0.00192
protein insertion into membrane	Biological Process	GO:0051205	0.00194
positive regulation of cytokine-mediated signaling pathway	Biological Process	GO:0001961	0.00196
interferon-beta production	Biological Process	GO:0032608	0.00196
phosphatidylinositol phosphorylation	Biological Process	GO:0046854	0.00196
positive regulation of G1/S transition of mitotic cell cycle	Biological Process	GO:1900087	0.00196
diencephalon development	Biological Process	GO:0021536	0.00199
activation of JUN kinase activity	Biological Process	GO:0007257	0.00206
negative regulation of muscle hypertrophy	Biological Process	GO:0014741	0.00206
vascular smooth muscle cell differentiation	Biological Process	GO:0035886	0.00206
membrane assembly	Biological Process	GO:0071709	0.00206
regulation of macrophage migration	Biological Process	GO:1905521	0.00206
negative regulation of mitotic sister chromatid separation	Biological Process	GO:2000816	0.00206
nucleoside diphosphate phosphorylation	Biological Process	GO:0006165	0.00208
negative regulation of transporter activity	Biological Process	GO:0032410	0.0021
cellular polysaccharide metabolic process	Biological Process	GO:0044264	0.00211
regulation of immunoglobulin production	Biological Process	GO:0002637	0.00217
sister chromatid cohesion	Biological Process	GO:0007062	0.00217
positive regulation of organ growth	Biological Process	GO:0046622	0.00217
heterochromatin assembly	Biological Process	GO:0031507	0.00219

vasoconstriction	Biological Process	GO:0042310	0.00219
regulation of mitochondrial membrane permeability	Biological Process	GO:0046902	0.00219
cell communication by electrical coupling	Biological Process	GO:0010644	0.00222
positive regulation of transforming growth factor beta receptor signaling pathway	Biological Process	GO:0030511	0.00222
interleukin-6 biosynthetic process	Biological Process	GO:0042226	0.00222
negative regulation of heart contraction	Biological Process	GO:0045822	0.00222
detection of mechanical stimulus involved in sensory perception	Biological Process	GO:0050974	0.00222
positive regulation of cellular response to transforming growth factor beta stimulus	Biological Process	GO:1903846	0.00222
regulation of stem cell population maintenance	Biological Process	GO:2000036	0.00222
oocyte differentiation	Biological Process	GO:0009994	0.00222
regulation of mitotic metaphase/anaphase transition	Biological Process	GO:0030071	0.00222
positive regulation of telomere maintenance	Biological Process	GO:0032206	0.00222
positive regulation of receptor-mediated endocytosis	Biological Process	GO:0048260	0.00222
response to bronchodilator	Biological Process	GO:0097366	0.00222
ribose phosphate metabolic process	Biological Process	GO:0019693	0.00227
neutrophil chemotaxis	Biological Process	GO:0030593	0.00228
regulation of nucleocytoplasmic transport	Biological Process	GO:0046822	0.00228
neural tube closure	Biological Process	GO:0001843	0.00228
chromosome separation	Biological Process	GO:0051304	0.00228
negative regulation of response to wounding	Biological Process	GO:1903035	0.00228
regulation of viral life cycle	Biological Process	GO:1903900	0.00234
positive regulation of cytokinesis	Biological Process	GO:0032467	0.00238
response to interleukin-7	Biological Process	GO:0098760	0.00238
cellular response to interleukin-7	Biological Process	GO:0098761	0.00238
negative regulation of chromosome separation	Biological Process	GO:1905819	0.00238
negative regulation of establishment of protein localization	Biological Process	GO:1904950	0.00238
mitochondrial membrane organization	Biological Process	GO:0007006	0.00238
nucleotide phosphorylation	Biological Process	GO:0046939	0.00238
sensory perception of mechanical stimulus	Biological Process	GO:0050954	0.00239
regulation of glycolytic process	Biological Process	GO:0006110	0.0024
adult locomotory behavior	Biological Process	GO:0008344	0.0024
negative regulation of protein ubiquitination	Biological Process	GO:0031397	0.0024
insulin secretion involved in cellular response to glucose stimulus	Biological Process	GO:0035773	0.0024
lipid phosphorylation	Biological Process	GO:0046834	0.0024
regulation of glutamate receptor signaling pathway	Biological Process	GO:1900449	0.0024
type B pancreatic cell development	Biological Process	GO:0003323	0.0024

cardioblast differentiation	Biological Process	GO:0010002	0.0024
response to manganese ion	Biological Process	GO:0010042	0.0024
positive regulation of lamellipodium assembly	Biological Process	GO:0010592	0.0024
signal transduction involved in regulation of gene expression	Biological Process	GO:0023019	0.0024
platelet formation	Biological Process	GO:0030220	0.0024
positive regulation of smooth muscle cell apoptotic process	Biological Process	GO:0034393	0.0024
interleukin-8 biosynthetic process	Biological Process	GO:0042228	0.0024
tongue development	Biological Process	GO:0043586	0.0024
histone H3 deacetylation	Biological Process	GO:0070932	0.0024
connective tissue replacement	Biological Process	GO:0097709	0.0024
positive regulation of innate immune response	Biological Process	GO:0045089	0.00243
positive regulation of lymphocyte mediated immunity	Biological Process	GO:0002708	0.00245
regulation of phospholipase A2 activity	Biological Process	GO:0032429	0.00245
response to vitamin E	Biological Process	GO:0033197	0.00245
response to macrophage colony-stimulating factor	Biological Process	GO:0036005	0.00245
cellular response to macrophage colony-stimulating factor stimulus	Biological Process	GO:0036006	0.00245
positive regulation of interleukin-8 biosynthetic process	Biological Process	GO:0045416	0.00245
response to ether	Biological Process	GO:0045472	0.00245
positive regulation of gluconeogenesis	Biological Process	GO:0045722	0.00245
NK T cell activation	Biological Process	GO:0051132	0.00245
positive regulation of keratinocyte migration	Biological Process	GO:0051549	0.00245
radial glial cell differentiation	Biological Process	GO:0060019	0.00245
positive regulation of neuron projection regeneration	Biological Process	GO:0070572	0.00245
commissural neuron axon guidance	Biological Process	GO:0071679	0.00245
distal tubule development	Biological Process	GO:0072017	0.00245
hepatocyte apoptotic process	Biological Process	GO:0097284	0.00245
regulation of IRE1-mediated unfolded protein response	Biological Process	GO:1903894	0.00245
positive regulation of vascular associated smooth muscle cell apoptotic process	Biological Process	GO:1905461	0.00245
regulation of mesenchymal cell apoptotic process	Biological Process	GO:2001053	0.00245
pyruvate metabolic process	Biological Process	GO:0006090	0.00245
protein acylation	Biological Process	GO:0043543	0.00245
tube closure	Biological Process	GO:0060606	0.00245
ruffle organization	Biological Process	GO:0031529	0.00247
positive regulation of heart growth	Biological Process	GO:0060421	0.00247
positive regulation of response to biotic stimulus	Biological Process	GO:0002833	0.00258
calcium ion transmembrane transport	Biological Process	GO:0070588	0.0026

cardiac atrium morphogenesis	Biological Process	GO:0003209	0.0026
positive regulation of histone acetylation	Biological Process	GO:0035066	0.0026
regulation of alpha-beta T cell proliferation	Biological Process	GO:0046640	0.0026
negative regulation of fibroblast proliferation	Biological Process	GO:0048147	0.0026
regulation of hydrogen peroxide-induced cell death	Biological Process	GO:1903205	0.0026
negative regulation of receptor signaling pathway via STAT	Biological Process	GO:1904893	0.0026
negative regulation of ion transmembrane transporter activity	Biological Process	GO:0032413	0.0026
neutrophil degranulation	Biological Process	GO:0043312	0.00263
positive regulation of interferon-gamma production	Biological Process	GO:0032729	0.00263
regulation of monooxygenase activity	Biological Process	GO:0032768	0.00263
ribonucleoside diphosphate metabolic process	Biological Process	GO:0009185	0.00268
regulation of transcription from RNA polymerase II promoter in response to stress	Biological Process	GO:0043618	0.00269
triglyceride biosynthetic process	Biological Process	GO:0019432	0.00269
positive regulation of B cell proliferation	Biological Process	GO:0030890	0.00269
lung alveolus development	Biological Process	GO:0048286	0.00269
lipid export from cell	Biological Process	GO:0140353	0.00269
regulation of extracellular matrix organization	Biological Process	GO:1903053	0.00269
hormone metabolic process	Biological Process	GO:0042445	0.00269
collagen biosynthetic process	Biological Process	GO:0032964	0.00278
regulation of metaphase/anaphase transition of cell cycle	Biological Process	GO:1902099	0.00278
macrophage migration	Biological Process	GO:1905517	0.00278
regulation of B cell activation	Biological Process	GO:0050864	0.0028
establishment of protein localization to mitochondrion	Biological Process	GO:0072655	0.00286
ADP metabolic process	Biological Process	GO:0046031	0.00289
neutrophil activation involved in immune response	Biological Process	GO:0002283	0.0029
regulation of systemic arterial blood pressure	Biological Process	GO:0003073	0.0029
synaptic transmission, glutamatergic	Biological Process	GO:0035249	0.0029
negative regulation of calcium ion transport	Biological Process	GO:0051926	0.00292
regulation of protein processing	Biological Process	GO:0070613	0.00292
epidermal cell differentiation	Biological Process	GO:0009913	0.00292
ribonucleotide biosynthetic process	Biological Process	GO:0009260	0.00296
regulation of hydrogen peroxide metabolic process	Biological Process	GO:0010310	0.00296
platelet morphogenesis	Biological Process	GO:0036344	0.00296
thymic T cell selection	Biological Process	GO:0045061	0.00296
response to corticosterone	Biological Process	GO:0051412	0.00296
cellular response to fluid shear stress	Biological Process	GO:0071498	0.00296

positive regulation of lymphocyte chemotaxis	Biological Process	GO:0140131	0.00296
regulation of T-helper 17 type immune response	Biological Process	GO:2000316	0.00296
positive regulation of stem cell differentiation	Biological Process	GO:2000738	0.00296
peptidyl-lysine acetylation	Biological Process	GO:0018394	0.00297
Fc-epsilon receptor signaling pathway	Biological Process	GO:0038095	0.00297
ribonucleotide metabolic process	Biological Process	GO:0009259	0.003
T cell receptor signaling pathway	Biological Process	GO:0050852	0.00304
regulation of T cell cytokine production	Biological Process	GO:0002724	0.00306
axis elongation	Biological Process	GO:0003401	0.00306
positive regulation of monooxygenase activity	Biological Process	GO:0032770	0.00306
lens fiber cell differentiation	Biological Process	GO:0070306	0.00306
positive regulation of cardiac muscle hypertrophy	Biological Process	GO:0010613	0.00307
stem cell division	Biological Process	GO:0017145	0.00307
regulation of myelination	Biological Process	GO:0031641	0.00307
negative regulation of mitotic sister chromatid segregation	Biological Process	GO:0033048	0.00307
response to copper ion	Biological Process	GO:0046688	0.00307
positive regulation of cytokine production involved in immune response	Biological Process	GO:0002720	0.00311
positive regulation of calcium ion transport into cytosol	Biological Process	GO:0010524	0.00311
regulation of DNA-dependent DNA replication	Biological Process	GO:0090329	0.00311
mitochondrial outer membrane permeabilization	Biological Process	GO:0097345	0.00311
nucleoside diphosphate metabolic process	Biological Process	GO:0009132	0.00312
regulation of mRNA metabolic process	Biological Process	GO:1903311	0.00315
embryonic heart tube morphogenesis	Biological Process	GO:0003143	0.00321
immune response-regulating cell surface receptor signaling pathway involved in phagocytosis	Biological Process	GO:0002433	0.00324
Fc-gamma receptor signaling pathway involved in phagocytosis	Biological Process	GO:0038096	0.00324
post-translational protein modification	Biological Process	GO:0043687	0.00325
negative regulation of neuron projection development	Biological Process	GO:0010977	0.0033
positive regulation of receptor biosynthetic process	Biological Process	GO:0010870	0.0033
positive regulation of pseudopodium assembly	Biological Process	GO:0031274	0.0033
regulation of heat generation	Biological Process	GO:0031650	0.0033
positive regulation of intracellular steroid hormone receptor signaling pathway	Biological Process	GO:0033145	0.0033
negative regulation of tyrosine phosphorylation of STAT protein	Biological Process	GO:0042532	0.0033
positive regulation of insulin-like growth factor receptor signaling pathway	Biological Process	GO:0043568	0.0033
negative regulation of endothelial cell differentiation	Biological Process	GO:0045602	0.0033
negative regulation of glycolytic process	Biological Process	GO:0045820	0.0033

regulation of timing of cell differentiation	Biological Process	GO:0048505	0.0033
negative regulation of oligodendrocyte differentiation	Biological Process	GO:0048715	0.0033
intestinal epithelial cell development	Biological Process	GO:0060576	0.0033
lateral sprouting from an epithelium	Biological Process	GO:0060601	0.0033
vascular wound healing	Biological Process	GO:0061042	0.0033
negative regulation of cell growth involved in cardiac muscle cell development	Biological Process	GO:0061052	0.0033
mesenchymal cell apoptotic process	Biological Process	GO:0097152	0.0033
positive regulation of endoplasmic reticulum unfolded protein response	Biological Process	GO:1900103	0.0033
positive regulation of ATP biosynthetic process	Biological Process	GO:2001171	0.0033
positive regulation of production of molecular mediator of immune response	Biological Process	GO:0002702	0.00338
regulation of receptor internalization	Biological Process	GO:0002090	0.00347
fat-soluble vitamin metabolic process	Biological Process	GO:0006775	0.00349
positive regulation of muscle hypertrophy	Biological Process	GO:0014742	0.00349
regulation of protein oligomerization	Biological Process	GO:0032459	0.00349
activin receptor signaling pathway	Biological Process	GO:0032924	0.00349
positive regulation of calcium-mediated signaling	Biological Process	GO:0050850	0.00349
purine ribonucleotide biosynthetic process	Biological Process	GO:0009152	0.0035
regulation of protein maturation	Biological Process	GO:1903317	0.00353
regulation of natural killer cell activation	Biological Process	GO:0032814	0.00358
cellular response to vitamin	Biological Process	GO:0071295	0.00358
organ induction	Biological Process	GO:0001759	0.00361
positive regulation of neuroblast proliferation	Biological Process	GO:0002052	0.00361
mitotic recombination	Biological Process	GO:0006312	0.00361
regulation of sister chromatid cohesion	Biological Process	GO:0007063	0.00361
positive regulation of insulin receptor signaling pathway	Biological Process	GO:0046628	0.00361
uterus development	Biological Process	GO:0060065	0.00361
cellular response to dsRNA	Biological Process	GO:0071359	0.00361
cellular response to low-density lipoprotein particle stimulus	Biological Process	GO:0071404	0.00361
regulation of branching involved in ureteric bud morphogenesis	Biological Process	GO:0090189	0.00361
positive regulation of protein deacetylation	Biological Process	GO:0090312	0.00361
negative regulation of protein acetylation	Biological Process	GO:1901984	0.00361
megakaryocyte differentiation	Biological Process	GO:0030219	0.00365
positive regulation of peptide hormone secretion	Biological Process	GO:0090277	0.00365
protein localization to mitochondrion	Biological Process	GO:0070585	0.00365
protein sumoylation	Biological Process	GO:0016925	0.00367
forebrain cell migration	Biological Process	GO:0021885	0.00387

methylation	Biological Process	GO:0032259	0.00389
activation of innate immune response	Biological Process	GO:0002218	0.0039
Fc-gamma receptor signaling pathway	Biological Process	GO:0038094	0.0039
regulation of striated muscle contraction	Biological Process	GO:0006942	0.00395
negative regulation of endothelial cell migration	Biological Process	GO:0010596	0.00395
fatty acid transport	Biological Process	GO:0015908	0.00395
negative regulation of sister chromatid segregation	Biological Process	GO:0033046	0.00396
positive regulation of protein dephosphorylation	Biological Process	GO:0035307	0.00396
membrane biogenesis	Biological Process	GO:0044091	0.00396
regulation of granulocyte chemotaxis	Biological Process	GO:0071622	0.00396
response to osmotic stress	Biological Process	GO:0006970	0.00401
protein localization to chromosome	Biological Process	GO:0034502	0.00401
positive regulation of translation	Biological Process	GO:0045727	0.00401
import into cell	Biological Process	GO:0098657	0.00403
myotube differentiation	Biological Process	GO:0014902	0.00404
sterol transport	Biological Process	GO:0015918	0.00404
protein import into nucleus	Biological Process	GO:0006606	0.00414
positive regulation of GTPase activity	Biological Process	GO:0043547	0.00415
regulation of lamellipodium assembly	Biological Process	GO:0010591	0.00415
regulation of keratinocyte proliferation	Biological Process	GO:0010837	0.00415
negative regulation of protein processing	Biological Process	GO:0010955	0.00415
negative regulation of macroautophagy	Biological Process	GO:0016242	0.00415
positive regulation of transcription from RNA polymerase II promoter in response to stress	Biological Process	GO:0036003	0.00415
brain morphogenesis	Biological Process	GO:0048854	0.00415
negative regulation of protein maturation	Biological Process	GO:1903318	0.00415
positive regulation of peptidyl-lysine acetylation	Biological Process	GO:2000758	0.00415
ribose phosphate biosynthetic process	Biological Process	GO:0046390	0.00424
regulation of T cell mediated immunity	Biological Process	GO:0002709	0.00425
regulation of toll-like receptor signaling pathway	Biological Process	GO:0034121	0.00425
regulation of synaptic transmission, glutamatergic	Biological Process	GO:0051966	0.00425
homologous chromosome segregation	Biological Process	GO:0045143	0.00429
regulation of response to endoplasmic reticulum stress	Biological Process	GO:1905897	0.00435
cardiac left ventricle morphogenesis	Biological Process	GO:0003214	0.00435
glial cell fate commitment	Biological Process	GO:0021781	0.00435
hyaluronan biosynthetic process	Biological Process	GO:0030213	0.00435

regulation of pseudopodium assembly	Biological Process	GO:0031272	0.00435
platelet-derived growth factor receptor-beta signaling pathway	Biological Process	GO:0035791	0.00435
phosphatidylinositol-3-phosphate biosynthetic process	Biological Process	GO:0036092	0.00435
regulation of development, heterochronic	Biological Process	GO:0040034	0.00435
negative regulation of epidermal cell differentiation	Biological Process	GO:0045605	0.00435
induction of positive chemotaxis	Biological Process	GO:0050930	0.00435
Sertoli cell development	Biological Process	GO:0060009	0.00435
positive regulation of extrinsic apoptotic signaling pathway via death domain receptors	Biological Process	GO:1902043	0.00435
adult behavior	Biological Process	GO:0030534	0.00437
regulation of histone H3-K9 methylation	Biological Process	GO:0051570	0.00437
detection of stimulus involved in sensory perception of pain	Biological Process	GO:0062149	0.00437
regulation of metanephros development	Biological Process	GO:0072215	0.00437
negative regulation of protein transport	Biological Process	GO:0051224	0.00444
negative regulation of chromosome segregation	Biological Process	GO:0051985	0.00446
regulation of mRNA stability	Biological Process	GO:0043488	0.00457
fatty acid oxidation	Biological Process	GO:0019395	0.00457
cholesterol transport	Biological Process	GO:0030301	0.00457
response to type I interferon	Biological Process	GO:0034340	0.00457
meiosis I	Biological Process	GO:0007127	0.00462
bone morphogenesis	Biological Process	GO:0060349	0.00462
Fc receptor mediated stimulatory signaling pathway	Biological Process	GO:0002431	0.00464
positive regulation of calcium ion transmembrane transport	Biological Process	GO:1904427	0.00465
syncytium formation	Biological Process	GO:0006949	0.00475
specification of animal organ identity	Biological Process	GO:0010092	0.0048
cell death in response to hydrogen peroxide	Biological Process	GO:0036474	0.0048
embryonic eye morphogenesis	Biological Process	GO:0048048	0.0048
ER-nucleus signaling pathway	Biological Process	GO:0006984	0.00505
negative regulation of epithelial cell apoptotic process	Biological Process	GO:1904036	0.00505
response to amino acid starvation	Biological Process	GO:1990928	0.00505
G2/M transition of mitotic cell cycle	Biological Process	GO:0000086	0.00507
protein methylation	Biological Process	GO:0006479	0.0051
protein alkylation	Biological Process	GO:0008213	0.0051
positive regulation of insulin secretion	Biological Process	GO:0032024	0.0051
forebrain generation of neurons	Biological Process	GO:0021872	0.00527
positive regulation of blood vessel diameter	Biological Process	GO:0097755	0.00527
negative regulation of reproductive process	Biological Process	GO:2000242	0.00527

dendritic cell chemotaxis	Biological Process	GO:0002407	0.00527
exogenous drug catabolic process	Biological Process	GO:0042738	0.00527
positive regulation of epidermal cell differentiation	Biological Process	GO:0045606	0.00527
secondary palate development	Biological Process	GO:0062009	0.00527
cell communication by electrical coupling involved in cardiac conduction	Biological Process	GO:0086064	0.00527
modification of synaptic structure	Biological Process	GO:0099563	0.00527
positive regulation of cellular response to insulin stimulus	Biological Process	GO:1900078	0.00527
positive regulation of glycoprotein metabolic process	Biological Process	GO:1903020	0.00527
double-strand break repair	Biological Process	GO:0006302	0.00528
regulation of heart rate	Biological Process	GO:0002027	0.0053
lipid oxidation	Biological Process	GO:0034440	0.0053
cell cycle G2/M phase transition	Biological Process	GO:0044839	0.00536
organic hydroxy compound biosynthetic process	Biological Process	GO:1901617	0.00536
maternal placenta development	Biological Process	GO:0001893	0.00554
central nervous system neuron axonogenesis	Biological Process	GO:0021955	0.00554
apoptotic nuclear changes	Biological Process	GO:0030262	0.00554
positive regulation of mitochondrial outer membrane permeabilization involved in apoptotic signaling pathway	Biological Process	GO:1901030	0.00554
oogenesis	Biological Process	GO:0048477	0.00554
B cell activation involved in immune response	Biological Process	GO:0002312	0.00557
multicellular organismal response to stress	Biological Process	GO:0033555	0.00557
trophectodermal cell differentiation	Biological Process	GO:0001829	0.00559
heart field specification	Biological Process	GO:0003128	0.00559
Rap protein signal transduction	Biological Process	GO:0032486	0.00559
response to laminar fluid shear stress	Biological Process	GO:0034616	0.00559
negative regulation of histone acetylation	Biological Process	GO:0035067	0.00559
activation of Janus kinase activity	Biological Process	GO:0042976	0.00559
positive regulation of interleukin-6 biosynthetic process	Biological Process	GO:0045410	0.00559
regulation of RNA polymerase II transcription preinitiation complex assembly	Biological Process	GO:0045898	0.00559
regulation of developmental pigmentation	Biological Process	GO:0048070	0.00559
negative regulation of androgen receptor signaling pathway	Biological Process	GO:0060766	0.00559
negative regulation of neuron projection regeneration	Biological Process	GO:0070571	0.00559
regulation of podosome assembly	Biological Process	GO:0071801	0.00559
epithelial cell fate commitment	Biological Process	GO:0072148	0.00559
regulation of glomerulus development	Biological Process	GO:0090192	0.00559
positive regulation of endothelial cell chemotaxis	Biological Process	GO:2001028	0.00559

regulation of peptidyl-threonine phosphorylation	Biological Process	GO:0010799	0.00562
regulation of protein autophosphorylation	Biological Process	GO:0031952	0.00562
neuron maturation	Biological Process	GO:0042551	0.00562
developmental pigmentation	Biological Process	GO:0048066	0.00562
regulation of phosphoprotein phosphatase activity	Biological Process	GO:0043666	0.00563
mast cell activation	Biological Process	GO:0045576	0.00578
positive regulation of mitochondrial membrane permeability involved in apoptotic process	Biological Process	GO:1902110	0.00578
regulation of mRNA catabolic process	Biological Process	GO:0061013	0.00597
negative regulation of NF-kappaB transcription factor activity	Biological Process	GO:0032088	0.00597
positive regulation of exocytosis	Biological Process	GO:0045921	0.00597
neuron projection organization	Biological Process	GO:0106027	0.00597
ear morphogenesis	Biological Process	GO:0042471	0.00603
neutrophil migration	Biological Process	GO:1990266	0.00603
lysosome localization	Biological Process	GO:0032418	0.00605
purine nucleoside diphosphate metabolic process	Biological Process	GO:0009135	0.00619
purine ribonucleoside diphosphate metabolic process	Biological Process	GO:0009179	0.00619
protein processing	Biological Process	GO:0016485	0.00622
regulation of exocytosis	Biological Process	GO:0017157	0.00622
regulation of RNA stability	Biological Process	GO:0043487	0.00622
type B pancreatic cell differentiation	Biological Process	GO:0003309	0.00623
central nervous system projection neuron axonogenesis	Biological Process	GO:0021952	0.00623
adrenal gland development	Biological Process	GO:0030325	0.00623
leukocyte tethering or rolling	Biological Process	GO:0050901	0.00623
positive regulation of positive chemotaxis	Biological Process	GO:0050927	0.00623
regulation of mesonephros development	Biological Process	GO:0061217	0.00623
eosinophil migration	Biological Process	GO:0072677	0.00623
regulation of endoplasmic reticulum unfolded protein response	Biological Process	GO:1900101	0.00623
purine ribonucleotide metabolic process	Biological Process	GO:0009150	0.00629
negative regulation of mitotic metaphase/anaphase transition	Biological Process	GO:0045841	0.0063
regulation of superoxide metabolic process	Biological Process	GO:0090322	0.0063
heart looping	Biological Process	GO:0001947	0.00636
positive regulation of viral life cycle	Biological Process	GO:1903902	0.00636
tumor necrosis factor-mediated signaling pathway	Biological Process	GO:0033209	0.00641
regulation of synapse organization	Biological Process	GO:0050807	0.0065
positive regulation of ion transmembrane transporter activity	Biological Process	GO:0032414	0.00653

mitotic sister chromatid segregation	Biological Process	GO:0000070	0.00653
DNA-dependent DNA replication	Biological Process	GO:0006261	0.00653
negative regulation of protein kinase B signaling	Biological Process	GO:0051898	0.00704
mitochondrial outer membrane permeabilization involved in programmed cell death	Biological Process	GO:1902686	0.00704
negative regulation of leukocyte migration	Biological Process	GO:0002686	0.00704
regulation of membrane depolarization	Biological Process	GO:0003254	0.00704
selective autophagy	Biological Process	GO:0061912	0.00704
regulation of glycoprotein metabolic process	Biological Process	GO:1903018	0.00704
lymphangiogenesis	Biological Process	GO:0001946	0.00704
T-helper cell lineage commitment	Biological Process	GO:0002295	0.00704
regulation of tolerance induction	Biological Process	GO:0002643	0.00704
regulation of cytokine secretion involved in immune response	Biological Process	GO:0002739	0.00704
positive regulation of type 2 immune response	Biological Process	GO:0002830	0.00704
response to UV-C	Biological Process	GO:0010225	0.00704
regulation of ketone biosynthetic process	Biological Process	GO:0010566	0.00704
positive regulation of cholesterol efflux	Biological Process	GO:0010875	0.00704
regulation of cholesterol storage	Biological Process	GO:0010885	0.00704
pseudopodium assembly	Biological Process	GO:0031269	0.00704
corticosteroid receptor signaling pathway	Biological Process	GO:0031958	0.00704
positive regulation of keratinocyte differentiation	Biological Process	GO:0045618	0.00704
ganglion development	Biological Process	GO:0061548	0.00704
positive regulation of calcineurin-NFAT signaling cascade	Biological Process	GO:0070886	0.00704
cellular response to prostaglandin E stimulus	Biological Process	GO:0071380	0.00704
cellular response to nitric oxide	Biological Process	GO:0071732	0.00704
nephric duct development	Biological Process	GO:0072176	0.00704
metanephric glomerulus development	Biological Process	GO:0072224	0.00704
G protein-coupled receptor signaling pathway involved in heart process	Biological Process	GO:0086103	0.00704
positive regulation of calcineurin-mediated signaling	Biological Process	GO:0106058	0.00704
regulation of extracellular matrix assembly	Biological Process	GO:1901201	0.00704
negative regulation of amyloid precursor protein catabolic process	Biological Process	GO:1902992	0.00704
negative regulation of endothelial cell proliferation	Biological Process	GO:0001937	0.00711
negative regulation of wound healing	Biological Process	GO:0061045	0.00711
cellular component disassembly involved in execution phase of apoptosis	Biological Process	GO:0006921	0.00714
Schwann cell differentiation	Biological Process	GO:0014037	0.00714
negative regulation of smooth muscle cell migration	Biological Process	GO:0014912	0.00714
cerebellum morphogenesis	Biological Process	GO:0021587	0.00714

embryonic camera-type eye development	Biological Process	GO:0031076	0.00714
secretion by tissue	Biological Process	GO:0032941	0.00714
histone H3-K9 methylation	Biological Process	GO:0051567	0.00714
positive regulation of cell migration involved in sprouting angiogenesis	Biological Process	GO:0090050	0.00714
negative regulation of metaphase/anaphase transition of cell cycle	Biological Process	GO:1902100	0.00714
apoptotic DNA fragmentation	Biological Process	GO:0006309	0.00729
DNA methylation-dependent heterochromatin assembly	Biological Process	GO:0006346	0.00729
ventricular system development	Biological Process	GO:0021591	0.00729
regulation of insulin-like growth factor receptor signaling pathway	Biological Process	GO:0043567	0.00729
negative regulation of embryonic development	Biological Process	GO:0045992	0.00729
regulation of positive chemotaxis	Biological Process	GO:0050926	0.00729
pharyngeal system development	Biological Process	GO:0060037	0.00729
regulation of receptor binding	Biological Process	GO:1900120	0.00729
regulation of protein insertion into mitochondrial membrane involved in apoptotic signaling pathway	Biological Process	GO:1900739	0.00729
positive regulation of protein insertion into mitochondrial membrane involved in apoptotic signaling pathway	Biological Process	GO:1900740	0.00729
positive regulation of vascular associated smooth muscle cell migration	Biological Process	GO:1904754	0.00729
regulation of metallopeptidase activity	Biological Process	GO:1905048	0.00729
cellular response to heat	Biological Process	GO:0034605	0.00729
positive regulation of nervous system process	Biological Process	GO:0031646	0.00763
protein localization to cell surface	Biological Process	GO:0034394	0.00763
regulation of transcription from RNA polymerase II promoter in response to hypoxia	Biological Process	GO:0061418	0.0077
anterior/posterior axis specification	Biological Process	GO:0009948	0.00777
body morphogenesis	Biological Process	GO:0010171	0.00777
regulation of microtubule polymerization	Biological Process	GO:0031113	0.00777
membrane repolarization	Biological Process	GO:0086009	0.00777
purine-containing compound biosynthetic process	Biological Process	GO:0072522	0.00783
negative regulation of insulin secretion	Biological Process	GO:0046676	0.00812
positive regulation of cardiac muscle cell proliferation	Biological Process	GO:0060045	0.00812
negative regulation of cellular response to insulin stimulus	Biological Process	GO:1900077	0.00812
positive regulation of response to endoplasmic reticulum stress	Biological Process	GO:1905898	0.00812
negative regulation of DNA biosynthetic process	Biological Process	GO:2000279	0.00812
negative regulation of DNA metabolic process	Biological Process	GO:0051053	0.00819
positive regulation of mitochondrial membrane permeability	Biological Process	GO:0035794	0.00837
negative regulation of chemotaxis	Biological Process	GO:0050922	0.00837

nucleotide biosynthetic process	Biological Process	GO:0009165	0.00857
regulation of the force of heart contraction	Biological Process	GO:0002026	0.00857
regulation of epidermal growth factor-activated receptor activity	Biological Process	GO:0007176	0.00857
dendritic cell migration	Biological Process	GO:0036336	0.00857
negative regulation of muscle contraction	Biological Process	GO:0045932	0.00857
collateral sprouting	Biological Process	GO:0048668	0.00857
regulation of histone H3-K4 methylation	Biological Process	GO:0051569	0.00857
negative regulation of cellular response to drug	Biological Process	GO:2001039	0.00857
regulation of pattern recognition receptor signaling pathway	Biological Process	GO:0062207	0.00859
regulation of skeletal muscle tissue development	Biological Process	GO:0048641	0.00863
regulation of nitric-oxide synthase activity	Biological Process	GO:0050999	0.00863
negative regulation of cation channel activity	Biological Process	GO:2001258	0.00863
regulation of postsynaptic membrane potential	Biological Process	GO:0060078	0.00867
DNA ligation	Biological Process	GO:0006266	0.00867
segment specification	Biological Process	GO:0007379	0.00867
regulation of T cell chemotaxis	Biological Process	GO:0010819	0.00867
RNA interference	Biological Process	GO:0016246	0.00867
response to caffeine	Biological Process	GO:0031000	0.00867
pseudopodium organization	Biological Process	GO:0031268	0.00867
chondrocyte proliferation	Biological Process	GO:0035988	0.00867
response to diuretic	Biological Process	GO:0036270	0.00867
cytoplasmic sequestering of transcription factor	Biological Process	GO:0042994	0.00867
CD4-positive, alpha-beta T cell lineage commitment	Biological Process	GO:0043373	0.00867
anatomical structure arrangement	Biological Process	GO:0048532	0.00867
hydrogen peroxide biosynthetic process	Biological Process	GO:0050665	0.00867
positive regulation of sequestering of calcium ion	Biological Process	GO:0051284	0.00867
regulation of respiratory burst	Biological Process	GO:0060263	0.00867
mitotic cell cycle arrest	Biological Process	GO:0071850	0.00867
negative regulation of kidney development	Biological Process	GO:0090185	0.00867
midbrain dopaminergic neuron differentiation	Biological Process	GO:1904948	0.00867
positive regulation of leukocyte adhesion to vascular endothelial cell	Biological Process	GO:1904996	0.00867
antigen receptor-mediated signaling pathway	Biological Process	GO:0050851	0.00892
negative regulation of mRNA metabolic process	Biological Process	GO:1903312	0.009
phospholipid metabolic process	Biological Process	GO:0006644	0.009
nucleotide-excision repair	Biological Process	GO:0006289	0.00902
phosphatidylinositol metabolic process	Biological Process	GO:0046488	0.00906

regulation of histone methylation	Biological Process	GO:0031060	0.00909
regulation of antigen receptor-mediated signaling pathway	Biological Process	GO:0050854	0.00909
cytokinetic process	Biological Process	GO:0032506	0.00912
positive regulation of epidermis development	Biological Process	GO:0045684	0.00912
regulation of membrane repolarization	Biological Process	GO:0060306	0.00912
TOR signaling	Biological Process	GO:0031929	0.00918
germ cell development	Biological Process	GO:0007281	0.00921
purine nucleotide biosynthetic process	Biological Process	GO:0006164	0.00948
protein import	Biological Process	GO:0017038	0.00948
somatic recombination of immunoglobulin gene segments	Biological Process	GO:0016447	0.0095
regulation of neuronal synaptic plasticity	Biological Process	GO:0048168	0.0095
negative regulation of mRNA catabolic process	Biological Process	GO:1902373	0.0095
regulation of synapse structure or activity	Biological Process	GO:0050803	0.00951
purine nucleotide metabolic process	Biological Process	GO:0006163	0.00954
dendritic spine organization	Biological Process	GO:0097061	0.00972
regulation of viral genome replication	Biological Process	GO:0045069	0.00984
type I interferon signaling pathway	Biological Process	GO:0060337	0.00984
cellular response to type I interferon	Biological Process	GO:0071357	0.00984
nucleoside phosphate biosynthetic process	Biological Process	GO:1901293	0.00988
myeloid dendritic cell activation	Biological Process	GO:0001773	0.00988
motor neuron axon guidance	Biological Process	GO:0008045	0.00988
negative regulation of steroid biosynthetic process	Biological Process	GO:0010894	0.00988
regulation of interferon-alpha production	Biological Process	GO:0032647	0.00988
negative regulation of peptidyl-serine phosphorylation	Biological Process	GO:0033137	0.00988
regulation of interleukin-6 biosynthetic process	Biological Process	GO:0045408	0.00988
regulation of transcription initiation from RNA polymerase II promoter	Biological Process	GO:0060260	0.00988
positive regulation of excitatory postsynaptic potential	Biological Process	GO:2000463	0.00988
acidic amino acid transport	Biological Process	GO:0015800	0.00988
regulation of mitochondrial membrane permeability involved in apoptotic process	Biological Process	GO:1902108	0.00988
positive regulation of membrane permeability	Biological Process	GO:1905710	0.00988
T cell cytokine production	Biological Process	GO:0002369	0.0103
negative regulation of cytokine biosynthetic process	Biological Process	GO:0042036	0.0103
positive regulation of heart contraction	Biological Process	GO:0045823	0.0103
positive regulation of release of sequestered calcium ion into cytosol	Biological Process	GO:0051281	0.0103
regulation of purine nucleotide biosynthetic process	Biological Process	GO:1900371	0.0103
regulation of organelle assembly	Biological Process	GO:1902115	0.0104

regulation of protein targeting	Biological Process	GO:1903533	0.0105
blood vessel endothelial cell proliferation involved in sprouting angiogenesis	Biological Process	GO:0002043	0.0105
telencephalon cell migration	Biological Process	GO:0022029	0.0105
cellular response to cAMP	Biological Process	GO:0071320	0.0105
negative regulation of ERBB signaling pathway	Biological Process	GO:1901185	0.0105
cholesterol storage	Biological Process	GO:0010878	0.0106
negative regulation of interleukin-10 production	Biological Process	GO:0032693	0.0106
negative regulation of epidermis development	Biological Process	GO:0045683	0.0106
regulation of immunoglobulin secretion	Biological Process	GO:0051023	0.0106
negative regulation of cell division	Biological Process	GO:0051782	0.0106
negative regulation of focal adhesion assembly	Biological Process	GO:0051895	0.0106
renal vesicle morphogenesis	Biological Process	GO:0072077	0.0106
positive regulation of branching involved in ureteric bud morphogenesis	Biological Process	GO:0090190	0.0106
craniofacial suture morphogenesis	Biological Process	GO:0097094	0.0106
negative regulation of cell-substrate junction organization	Biological Process	GO:0150118	0.0106
fatty acid transmembrane transport	Biological Process	GO:1902001	0.0106
regulation of cell-cell adhesion mediated by cadherin	Biological Process	GO:2000047	0.0106
negative regulation of stem cell proliferation	Biological Process	GO:2000647	0.0106
negative regulation of peptidyl-lysine acetylation	Biological Process	GO:2000757	0.0106
cyclic-nucleotide-mediated signaling	Biological Process	GO:0019935	0.0106
determination of heart left/right asymmetry	Biological Process	GO:0061371	0.0107
positive regulation of transporter activity	Biological Process	GO:0032411	0.0108
protein maturation	Biological Process	GO:0051604	0.011
vesicle-mediated transport in synapse	Biological Process	GO:0099003	0.0111
meiotic cell cycle	Biological Process	GO:0051321	0.0112
dendritic spine development	Biological Process	GO:0060996	0.0112
steroid biosynthetic process	Biological Process	GO:0006694	0.0113
epithelial structure maintenance	Biological Process	GO:0010669	0.0114
positive regulation of peptidyl-threonine phosphorylation	Biological Process	GO:0010800	0.0114
regulation of histone deacetylation	Biological Process	GO:0031063	0.0114
positive regulation of microtubule polymerization	Biological Process	GO:0031116	0.0114
regulation of cytoplasmic translation	Biological Process	GO:2000765	0.0114
DNA catabolic process	Biological Process	GO:0006308	0.0114
myoblast fusion	Biological Process	GO:0007520	0.0114
hindbrain morphogenesis	Biological Process	GO:0021575	0.0114
regulation of nucleotide biosynthetic process	Biological Process	GO:0030808	0.0114

forelimb morphogenesis	Biological Process	GO:0035136	0.0114
positive regulation of embryonic development	Biological Process	GO:0040019	0.0114
skeletal muscle tissue regeneration	Biological Process	GO:0043403	0.0114
regulation of calcium ion import	Biological Process	GO:0090279	0.0114
modulation of excitatory postsynaptic potential	Biological Process	GO:0098815	0.0114
positive regulation of cardiocyte differentiation	Biological Process	GO:1905209	0.0114
negative regulation of DNA binding	Biological Process	GO:0043392	0.0115
specification of symmetry	Biological Process	GO:0009799	0.0115
stimulatory C-type lectin receptor signaling pathway	Biological Process	GO:0002223	0.0115
blastocyst development	Biological Process	GO:0001824	0.012
inner ear morphogenesis	Biological Process	GO:0042472	0.012
pigmentation	Biological Process	GO:0043473	0.012
import into nucleus	Biological Process	GO:0051170	0.012
postsynapse organization	Biological Process	GO:0099173	0.012
glycerolipid biosynthetic process	Biological Process	GO:0045017	0.0121
hematopoietic stem cell differentiation	Biological Process	GO:0060218	0.0121
carbohydrate catabolic process	Biological Process	GO:0016052	0.0123
response to cocaine	Biological Process	GO:0042220	0.0126
regulation of fatty acid biosynthetic process	Biological Process	GO:0042304	0.0126
icosanoid biosynthetic process	Biological Process	GO:0046456	0.0126
regulation of action potential	Biological Process	GO:0098900	0.0126
positive regulation of T cell cytokine production	Biological Process	GO:0002726	0.0127
negative regulation of systemic arterial blood pressure	Biological Process	GO:0003085	0.0127
regulation of exit from mitosis	Biological Process	GO:0007096	0.0127
negative regulation of calcium ion transport into cytosol	Biological Process	GO:0010523	0.0127
response to magnesium ion	Biological Process	GO:0032026	0.0127
negative regulation of interleukin-17 production	Biological Process	GO:0032700	0.0127
positive regulation of superoxide anion generation	Biological Process	GO:0032930	0.0127
response to vitamin A	Biological Process	GO:0033189	0.0127
podosome assembly	Biological Process	GO:0071800	0.0127
renal vesicle development	Biological Process	GO:0072087	0.0127
liver morphogenesis	Biological Process	GO:0072576	0.0127
detection of external biotic stimulus	Biological Process	GO:0098581	0.0127
fatty acid derivative biosynthetic process	Biological Process	GO:1901570	0.0127
regulation of gastrulation	Biological Process	GO:0010470	0.0127
regulation of glycoprotein biosynthetic process	Biological Process	GO:0010559	0.0127

negative regulation of cellular carbohydrate metabolic process	Biological Process	GO:0010677	0.0127
negative regulation of histone modification	Biological Process	GO:0031057	0.0127
regulation of autophagy of mitochondrion	Biological Process	GO:1903146	0.0127
microtubule cytoskeleton organization involved in mitosis	Biological Process	GO:1902850	0.0128
protein deneddylation	Biological Process	GO:0000338	0.0128
establishment of T cell polarity	Biological Process	GO:0001768	0.0128
natural killer cell proliferation	Biological Process	GO:0001787	0.0128
mitral valve morphogenesis	Biological Process	GO:0003183	0.0128
Notch receptor processing	Biological Process	GO:0007220	0.0128
regulation of nitric oxide mediated signal transduction	Biological Process	GO:0010749	0.0128
regulation of deoxyribonuclease activity	Biological Process	GO:0032070	0.0128
positive regulation of prostaglandin secretion	Biological Process	GO:0032308	0.0128
negative regulation of hormone biosynthetic process	Biological Process	GO:0032353	0.0128
positive regulation of protein homooligomerization	Biological Process	GO:0032464	0.0128
regulation of DNA endoreduplication	Biological Process	GO:0032875	0.0128
regulation of glial cell apoptotic process	Biological Process	GO:0034350	0.0128
positive regulation of transcription by RNA polymerase III	Biological Process	GO:0045945	0.0128
autophagic cell death	Biological Process	GO:0048102	0.0128
paraxial mesoderm morphogenesis	Biological Process	GO:0048340	0.0128
regulation of NK T cell activation	Biological Process	GO:0051133	0.0128
vagina development	Biological Process	GO:0060068	0.0128
cellular response to iron ion	Biological Process	GO:0071281	0.0128
glomerular visceral epithelial cell development	Biological Process	GO:0072015	0.0128
dendritic cell apoptotic process	Biological Process	GO:0097048	0.0128
type B pancreatic cell apoptotic process	Biological Process	GO:0097050	0.0128
positive regulation of receptor binding	Biological Process	GO:1900122	0.0128
positive regulation of response to reactive oxygen species	Biological Process	GO:1901033	0.0128
negative regulation of production of miRNAs involved in gene silencing by miRNA	Biological Process	GO:1903799	0.0128
positive regulation of cell-cell adhesion mediated by cadherin	Biological Process	GO:2000049	0.0128
regulation of macrophage apoptotic process	Biological Process	GO:2000109	0.0128
positive regulation of fibroblast apoptotic process	Biological Process	GO:2000271	0.0128
regulation of apoptotic cell clearance	Biological Process	GO:2000425	0.0128
regulation of miRNA metabolic process	Biological Process	GO:2000628	0.0128
regulation of receptor catabolic process	Biological Process	GO:2000644	0.0128
regulation of dendritic cell apoptotic process	Biological Process	GO:2000668	0.0128
detection of biotic stimulus	Biological Process	GO:0009595	0.0128

olfactory bulb development	Biological Process	GO:0021772	0.0128
interferon-alpha production	Biological Process	GO:0032607	0.0128
positive regulation of interferon-beta production	Biological Process	GO:0032728	0.0128
positive regulation of interleukin-2 production	Biological Process	GO:0032743	0.0128
negative regulation of steroid metabolic process	Biological Process	GO:0045939	0.0128
positive regulation of steroid metabolic process	Biological Process	GO:0045940	0.0128
negative regulation of B cell activation	Biological Process	GO:0050869	0.0128
interleukin-1-mediated signaling pathway	Biological Process	GO:0070498	0.0134
endomembrane system organization	Biological Process	GO:0010256	0.0136
innate immune response activating cell surface receptor signaling pathway	Biological Process	GO:0002220	0.0136
syncytium formation by plasma membrane fusion	Biological Process	GO:0000768	0.0136
mitotic spindle assembly	Biological Process	GO:0090307	0.0136
cell-cell fusion	Biological Process	GO:0140253	0.0136
retina development in camera-type eye	Biological Process	GO:0060041	0.0137
regulation of hematopoietic progenitor cell differentiation	Biological Process	GO:1901532	0.0137
regulation of calcium ion-dependent exocytosis	Biological Process	GO:0017158	0.014
cellular response to amino acid starvation	Biological Process	GO:0034198	0.014
keratinocyte proliferation	Biological Process	GO:0043616	0.014
response to electrical stimulus	Biological Process	GO:0051602	0.014
adipose tissue development	Biological Process	GO:0060612	0.014
positive regulation of protein acetylation	Biological Process	GO:1901985	0.014
negative regulation of calcium ion transmembrane transport	Biological Process	GO:1903170	0.014
regulation of DNA recombination	Biological Process	GO:0000018	0.0143
positive regulation of cell junction assembly	Biological Process	GO:1901890	0.0143
innate immune response-activating signal transduction	Biological Process	GO:0002758	0.0144
activation of phospholipase C activity	Biological Process	GO:0007202	0.0147
proximal/distal pattern formation	Biological Process	GO:0009954	0.0147
exit from mitosis	Biological Process	GO:0010458	0.0147
olfactory lobe development	Biological Process	GO:0021988	0.0147
positive regulation of erythrocyte differentiation	Biological Process	GO:0045648	0.0147
long-term synaptic depression	Biological Process	GO:0060292	0.0147
regulation of intrinsic apoptotic signaling pathway by p53 class mediator	Biological Process	GO:1902253	0.0147
long-term synaptic potentiation	Biological Process	GO:0060291	0.0147
hydrogen peroxide metabolic process	Biological Process	GO:0042743	0.0149
cartilage condensation	Biological Process	GO:0001502	0.0149
regulation of antigen processing and presentation	Biological Process	GO:0002577	0.0149

positive regulation of T-helper 1 type immune response	Biological Process	GO:0002827	0.0149
cardiac right ventricle morphogenesis	Biological Process	GO:0003215	0.0149
positive regulation of glycoprotein biosynthetic process	Biological Process	GO:0010560	0.0149
regulation of protein kinase A signaling	Biological Process	GO:0010738	0.0149
epoxygenase P450 pathway	Biological Process	GO:0019373	0.0149
regulation of protein homooligomerization	Biological Process	GO:0032462	0.0149
positive regulation of CREB transcription factor activity	Biological Process	GO:0032793	0.0149
positive regulation of natural killer cell activation	Biological Process	GO:0032816	0.0149
response to hydroperoxide	Biological Process	GO:0033194	0.0149
negative regulation of nitric oxide biosynthetic process	Biological Process	GO:0045019	0.0149
regulation of monocyte differentiation	Biological Process	GO:0045655	0.0149
regulation of neurotransmitter uptake	Biological Process	GO:0051580	0.0149
Sertoli cell differentiation	Biological Process	GO:0060008	0.0149
positive regulation of transcription initiation from RNA polymerase II promoter	Biological Process	GO:0060261	0.0149
positive regulation of transforming growth factor beta production	Biological Process	GO:0071636	0.0149
metanephric nephron tubule development	Biological Process	GO:0072234	0.0149
dendritic spine maintenance	Biological Process	GO:0097062	0.0149
negative regulation of nitric oxide metabolic process	Biological Process	GO:1904406	0.0149
positive regulation of macrophage migration	Biological Process	GO:1905523	0.0149
peptidyl-tyrosine dephosphorylation	Biological Process	GO:0035335	0.0151
oligodendrocyte development	Biological Process	GO:0014003	0.0155
glutamate secretion	Biological Process	GO:0014047	0.0155
regulation of endocrine process	Biological Process	GO:0044060	0.0155
cell-cell adhesion mediated by cadherin	Biological Process	GO:0044331	0.0155
negative regulation of cell migration involved in sprouting angiogenesis	Biological Process	GO:0090051	0.0155
regulation of protein deacetylation	Biological Process	GO:0090311	0.0155
regulation of mitochondrial outer membrane permeabilization involved in apoptotic signaling pathway	Biological Process	GO:1901028	0.0155
regulation of axon guidance	Biological Process	GO:1902667	0.0155
regulation of establishment of protein localization to mitochondrion	Biological Process	GO:1903747	0.0157
positive regulation of cation channel activity	Biological Process	GO:2001259	0.0157
DNA geometric change	Biological Process	GO:0032392	0.0161
estrogen metabolic process	Biological Process	GO:0008210	0.0164
attachment of spindle microtubules to kinetochore	Biological Process	GO:0008608	0.0164
regulation of synaptic transmission, GABAergic	Biological Process	GO:0032228	0.0164
positive regulation of smooth muscle contraction	Biological Process	GO:0045987	0.0164

regulation of axon extension involved in axon guidance	Biological Process	GO:0048841	0.0164
cellular response to interleukin-4	Biological Process	GO:0071353	0.0164
positive regulation of p38MAPK cascade	Biological Process	GO:1900745	0.0164
regulation of endoplasmic reticulum stress-induced intrinsic apoptotic signaling pathway	Biological Process	GO:1902235	0.0164
positive regulation of interleukin-6 secretion	Biological Process	GO:2000778	0.0164
activation of MAPKKK activity	Biological Process	GO:0000185	0.0164
establishment of lymphocyte polarity	Biological Process	GO:0001767	0.0164
pro-B cell differentiation	Biological Process	GO:0002328	0.0164
regulation of dendritic cell antigen processing and presentation	Biological Process	GO:0002604	0.0164
negative regulation of immunoglobulin production	Biological Process	GO:0002638	0.0164
mitral valve development	Biological Process	GO:0003174	0.0164
activation of JNKK activity	Biological Process	GO:0007256	0.0164
lateral ventricle development	Biological Process	GO:0021670	0.0164
production of siRNA involved in RNA interference	Biological Process	GO:0030422	0.0164
regulation of prostaglandin secretion	Biological Process	GO:0032306	0.0164
negative regulation of hormone metabolic process	Biological Process	GO:0032351	0.0164
negative regulation of protein sumoylation	Biological Process	GO:0033234	0.0164
regulation of NAD(P)H oxidase activity	Biological Process	GO:0033860	0.0164
histone-serine phosphorylation	Biological Process	GO:0035404	0.0164
CD4-positive, alpha-beta T cell proliferation	Biological Process	GO:0035739	0.0164
negative regulation of multicellular organism growth	Biological Process	GO:0040015	0.0164
DNA endoreduplication	Biological Process	GO:0042023	0.0164
susceptibility to natural killer cell mediated cytotoxicity	Biological Process	GO:0042271	0.0164
positive regulation of odontogenesis	Biological Process	GO:0042482	0.0164
negative thymic T cell selection	Biological Process	GO:0045060	0.0164
type I interferon biosynthetic process	Biological Process	GO:0045351	0.0164
regulation of T-helper 2 cell differentiation	Biological Process	GO:0045628	0.0164
positive regulation of RNA polymerase II transcription preinitiation complex assembly	Biological Process	GO:0045899	0.0164
positive regulation of axon regeneration	Biological Process	GO:0048680	0.0164
negative regulation of histone H3-K9 methylation	Biological Process	GO:0051573	0.0164
positive regulation of histone H3-K9 methylation	Biological Process	GO:0051574	0.0164
negative regulation of telomerase activity	Biological Process	GO:0051974	0.0164
embryonic skeletal joint morphogenesis	Biological Process	GO:0060272	0.0164
cardiac muscle cell myoblast differentiation	Biological Process	GO:0060379	0.0164
cardiac vascular smooth muscle cell differentiation	Biological Process	GO:0060947	0.0164

interleukin-27-mediated signaling pathway	Biological Process	GO:0070106	0.0164
response to interleukin-18	Biological Process	GO:0070673	0.0164
interleukin-35-mediated signaling pathway	Biological Process	GO:0070757	0.0164
metanephric tubule morphogenesis	Biological Process	GO:0072173	0.0164
glomerular epithelial cell development	Biological Process	GO:0072310	0.0164
fibroblast activation	Biological Process	GO:0072537	0.0164
T cell extravasation	Biological Process	GO:0072683	0.0164
synapse pruning	Biological Process	GO:0098883	0.0164
regulation of aspartic-type peptidase activity	Biological Process	GO:1905245	0.0164
positive regulation of transcription from RNA polymerase II promoter in response to endoplasmic reticulum stress	Biological Process	GO:1990440	0.0164
positive regulation of T-helper 17 type immune response	Biological Process	GO:2000318	0.0164
regulation of CD4-positive, alpha-beta T cell proliferation	Biological Process	GO:2000561	0.0164
regulation of osteoclast development	Biological Process	GO:2001204	0.0164
meiotic cell cycle process	Biological Process	GO:1903046	0.0166
superoxide metabolic process	Biological Process	GO:0006801	0.0167
endochondral bone morphogenesis	Biological Process	GO:0060350	0.0167
renal system process	Biological Process	GO:0003014	0.0167
glycerolipid metabolic process	Biological Process	GO:0046486	0.0168
mRNA stabilization	Biological Process	GO:0048255	0.0169
negative regulation of peptide hormone secretion	Biological Process	GO:0090278	0.0169
negative regulation of muscle cell apoptotic process	Biological Process	GO:0010656	0.0172
regulation of striated muscle cell apoptotic process	Biological Process	GO:0010662	0.0172
negative regulation of innate immune response	Biological Process	GO:0045824	0.0172
negative regulation of RNA catabolic process	Biological Process	GO:1902369	0.0172
negative regulation of glucose transmembrane transport	Biological Process	GO:0010829	0.0172
positive regulation of steroid biosynthetic process	Biological Process	GO:0010893	0.0172
antibiotic biosynthetic process	Biological Process	GO:0017000	0.0172
cerebellar cortex formation	Biological Process	GO:0021697	0.0172
maintenance of gastrointestinal epithelium	Biological Process	GO:0030277	0.0172
ovulation	Biological Process	GO:0030728	0.0172
embryonic hemopoiesis	Biological Process	GO:0035162	0.0172
CD4-positive, alpha-beta T cell cytokine production	Biological Process	GO:0035743	0.0172
PERK-mediated unfolded protein response	Biological Process	GO:0036499	0.0172
muscle cell cellular homeostasis	Biological Process	GO:0046716	0.0172
eosinophil chemotaxis	Biological Process	GO:0048245	0.0172

regulation of mitochondrial depolarization	Biological Process	GO:0051900	0.0172
labyrinthine layer morphogenesis	Biological Process	GO:0060713	0.0172
positive regulation of mesonephros development	Biological Process	GO:0061213	0.0172
positive regulation of extracellular matrix organization	Biological Process	GO:1903055	0.0172
beta-catenin destruction complex disassembly	Biological Process	GO:1904886	0.0172
negative regulation of stem cell differentiation	Biological Process	GO:2000737	0.0172
triglyceride metabolic process	Biological Process	GO:0006641	0.0179
regulation of dendritic spine development	Biological Process	GO:0060998	0.0179
protein kinase A signaling	Biological Process	GO:0010737	0.0184
granulocyte differentiation	Biological Process	GO:0030851	0.0184
positive regulation of microtubule polymerization or depolymerization	Biological Process	GO:0031112	0.0184
embryonic forelimb morphogenesis	Biological Process	GO:0035115	0.0184
neurotrophin TRK receptor signaling pathway	Biological Process	GO:0048011	0.0184
positive regulation of interleukin-1 beta secretion	Biological Process	GO:0050718	0.0184
regulation of T cell apoptotic process	Biological Process	GO:0070232	0.0184
negative regulation of potassium ion transmembrane transport	Biological Process	GO:1901380	0.0184
inflammatory response to antigenic stimulus	Biological Process	GO:0002437	0.0186
negative regulation of lipid transport	Biological Process	GO:0032369	0.0186
negative regulation of blood pressure	Biological Process	GO:0045776	0.0186
vascular associated smooth muscle cell migration	Biological Process	GO:1904738	0.0186
regulation of vascular associated smooth muscle cell migration	Biological Process	GO:1904752	0.0186
midbrain development	Biological Process	GO:0030901	0.0188
maintenance of protein location	Biological Process	GO:0045185	0.0188
regulation of potassium ion transmembrane transport	Biological Process	GO:1901379	0.0188
lysosomal transport	Biological Process	GO:0007041	0.0189
mitotic spindle organization	Biological Process	GO:0007052	0.0189
chromatin assembly or disassembly	Biological Process	GO:0006333	0.0194
regulation of dendrite morphogenesis	Biological Process	GO:0048814	0.0201
positive regulation of viral process	Biological Process	GO:0048524	0.0201
mitochondrial genome maintenance	Biological Process	GO:0000002	0.0202
mitophagy	Biological Process	GO:0000423	0.0202
lens morphogenesis in camera-type eye	Biological Process	GO:0002089	0.0202
central nervous system myelination	Biological Process	GO:0022010	0.0202
axon ensheathment in central nervous system	Biological Process	GO:0032291	0.0202
endoplasmic reticulum calcium ion homeostasis	Biological Process	GO:0032469	0.0202
positive regulation of interferon-alpha production	Biological Process	GO:0032727	0.0202

regulation of superoxide anion generation	Biological Process	GO:0032928	0.0202
integrin activation	Biological Process	GO:0033622	0.0202
interleukin-2 biosynthetic process	Biological Process	GO:0042094	0.0202
positive regulation of myoblast differentiation	Biological Process	GO:0045663	0.0202
immunoglobulin secretion	Biological Process	GO:0048305	0.0202
positive regulation of nitric-oxide synthase activity	Biological Process	GO:0051000	0.0202
cellular response to prostaglandin stimulus	Biological Process	GO:0071379	0.0202
negative regulation of protein localization to plasma membrane	Biological Process	GO:1903077	0.0202
regulation of tumor necrosis factor-mediated signaling pathway	Biological Process	GO:0010803	0.0203
synaptic vesicle cycle	Biological Process	GO:0099504	0.0204
cerebellar cortex development	Biological Process	GO:0021695	0.0204
endocrine hormone secretion	Biological Process	GO:0060986	0.0204
negative regulation of cold-induced thermogenesis	Biological Process	GO:0120163	0.0204
microtubule polymerization	Biological Process	GO:0046785	0.0204
DNA catabolic process, endonucleolytic	Biological Process	GO:0000737	0.0204
mitotic spindle assembly checkpoint	Biological Process	GO:0007094	0.0204
spindle checkpoint	Biological Process	GO:0031577	0.0204
embryonic digestive tract development	Biological Process	GO:0048566	0.0204
positive regulation of pattern recognition receptor signaling pathway	Biological Process	GO:0062208	0.0204
response to interleukin-4	Biological Process	GO:0070670	0.0204
spindle assembly checkpoint	Biological Process	GO:0071173	0.0204
mitotic spindle checkpoint	Biological Process	GO:0071174	0.0204
energy homeostasis	Biological Process	GO:0097009	0.0204
centrosome cycle	Biological Process	GO:0007098	0.0204
dendritic cell cytokine production	Biological Process	GO:0002371	0.0204
dendritic cell antigen processing and presentation	Biological Process	GO:0002468	0.0204
ER overload response	Biological Process	GO:0006983	0.0204
mitotic nuclear envelope disassembly	Biological Process	GO:0007077	0.0204
regulation of sequestering of triglyceride	Biological Process	GO:0010889	0.0204
muscle atrophy	Biological Process	GO:0014889	0.0204
regulation of prostaglandin biosynthetic process	Biological Process	GO:0031392	0.0204
hemidesmosome assembly	Biological Process	GO:0031581	0.0204
mitochondrial DNA metabolic process	Biological Process	GO:0032042	0.0204
regulation of cell fate specification	Biological Process	GO:0042659	0.0204
positive regulation of vascular permeability	Biological Process	GO:0043117	0.0204
negative T cell selection	Biological Process	GO:0043383	0.0204

saliva secretion	Biological Process	GO:0046541	0.0204
negative regulation of neurotransmitter secretion	Biological Process	GO:0046929	0.0204
negative regulation of skeletal muscle tissue development	Biological Process	GO:0048642	0.0204
peripheral nervous system neuron differentiation	Biological Process	GO:0048934	0.0204
peripheral nervous system neuron development	Biological Process	GO:0048935	0.0204
regulation of attachment of spindle microtubules to kinetochore	Biological Process	GO:0051988	0.0204
negative regulation of pathway-restricted SMAD protein phosphorylation	Biological Process	GO:0060394	0.0204
branching involved in prostate gland morphogenesis	Biological Process	GO:0060442	0.0204
trophoblast giant cell differentiation	Biological Process	GO:0060707	0.0204
trophoblast cell migration	Biological Process	GO:0061450	0.0204
regulation of thymocyte apoptotic process	Biological Process	GO:0070243	0.0204
cellular response to X-ray	Biological Process	GO:0071481	0.0204
macrophage apoptotic process	Biological Process	GO:0071888	0.0204
T-helper 17 cell lineage commitment	Biological Process	GO:0072540	0.0204
regulation of trophoblast cell migration	Biological Process	GO:1901163	0.0204
regulation of hydrogen peroxide-mediated programmed cell death	Biological Process	GO:1901298	0.0204
regulation of receptor clustering	Biological Process	GO:1903909	0.0204
positive regulation of membrane depolarization	Biological Process	GO:1904181	0.0204
regulation of GTP binding	Biological Process	GO:1904424	0.0204
positive regulation of ubiquitin protein ligase activity	Biological Process	GO:1904668	0.0204
regulation of modification of synaptic structure	Biological Process	GO:1905244	0.0204
negative regulation of neuron migration	Biological Process	GO:2001223	0.0204
keratinocyte differentiation	Biological Process	GO:0030216	0.0205
energy derivation by oxidation of organic compounds	Biological Process	GO:0015980	0.0205
DNA conformation change	Biological Process	GO:0071103	0.0212
xenobiotic metabolic process	Biological Process	GO:0006805	0.0215
regulation of G protein-coupled receptor signaling pathway	Biological Process	GO:0008277	0.0216
purine-containing compound metabolic process	Biological Process	GO:0072521	0.0216
regulation of cell cycle G2/M phase transition	Biological Process	GO:1902749	0.0217
respiratory gaseous exchange by respiratory system	Biological Process	GO:0007585	0.0217
striated muscle cell apoptotic process	Biological Process	GO:0010658	0.0217
regulation of myotube differentiation	Biological Process	GO:0010830	0.0217
interleukin-2 production	Biological Process	GO:0032623	0.0217
positive regulation of nucleocytoplasmic transport	Biological Process	GO:0046824	0.0217
negative regulation of chromatin organization	Biological Process	GO:1905268	0.0217
interaction with symbiont	Biological Process	GO:0051702	0.0218

regulation of double-strand break repair	Biological Process	GO:2000779	0.0218
positive regulation of T cell mediated immunity	Biological Process	GO:0002711	0.0221
negative regulation of epidermal growth factor receptor signaling pathway	Biological Process	GO:0042059	0.0221
regulation of regulated secretory pathway	Biological Process	GO:1903305	0.0223
regulation of amine transport	Biological Process	GO:0051952	0.0223
vacuolar transport	Biological Process	GO:0007034	0.0226
regulation of G2/M transition of mitotic cell cycle	Biological Process	GO:0010389	0.0227
negative regulation of interferon-gamma production	Biological Process	GO:0032689	0.0227
dendrite extension	Biological Process	GO:0097484	0.0227
regulation of renal system process	Biological Process	GO:0098801	0.0227
negative regulation of response to drug	Biological Process	GO:2001024	0.0227
response to tumor cell	Biological Process	GO:0002347	0.0229
regulation of transcription by RNA polymerase III	Biological Process	GO:0006359	0.0229
male genitalia development	Biological Process	GO:0030539	0.0229
positive regulation of nucleotide biosynthetic process	Biological Process	GO:0030810	0.0229
positive regulation of sterol transport	Biological Process	GO:0032373	0.0229
positive regulation of cholesterol transport	Biological Process	GO:0032376	0.0229
low-density lipoprotein receptor particle metabolic process	Biological Process	GO:0032799	0.0229
positive regulation of toll-like receptor signaling pathway	Biological Process	GO:0034123	0.0229
regulation of respiratory gaseous exchange	Biological Process	GO:0043576	0.0229
negative regulation of lipid catabolic process	Biological Process	GO:0050995	0.0229
negative regulation of smooth muscle cell differentiation	Biological Process	GO:0051151	0.0229
negative regulation of neurotransmitter transport	Biological Process	GO:0051589	0.0229
mitochondrial depolarization	Biological Process	GO:0051882	0.0229
negative regulation of cell cycle arrest	Biological Process	GO:0071157	0.0229
metanephric tubule development	Biological Process	GO:0072170	0.0229
metanephric nephron epithelium development	Biological Process	GO:0072243	0.0229
amino acid import across plasma membrane	Biological Process	GO:0089718	0.0229
cell aggregation	Biological Process	GO:0098743	0.0229
positive regulation of purine nucleotide biosynthetic process	Biological Process	GO:1900373	0.0229
negative regulation of response to reactive oxygen species	Biological Process	GO:1901032	0.0229
negative regulation of intrinsic apoptotic signaling pathway by p53 class mediator	Biological Process	GO:1902254	0.0229
negative regulation of hydrogen peroxide-induced cell death	Biological Process	GO:1903206	0.0229
positive regulation of reproductive process	Biological Process	GO:2000243	0.0232
cellular iron ion homeostasis	Biological Process	GO:0006879	0.0233
defense response to bacterium	Biological Process	GO:0042742	0.0244

nerve development	Biological Process	GO:0021675	0.0249
regulation of blood coagulation	Biological Process	GO:0030193	0.0249
regulation of microtubule polymerization or depolymerization	Biological Process	GO:0031110	0.0249
determination of bilateral symmetry	Biological Process	GO:0009855	0.0249
protein heterooligomerization	Biological Process	GO:0051291	0.0249
cellular lipid catabolic process	Biological Process	GO:0044242	0.0249
carboxylic acid transport	Biological Process	GO:0046942	0.0249
somatic diversification of immunoglobulins	Biological Process	GO:0016445	0.0249
telomere maintenance via recombination	Biological Process	GO:0000722	0.0249
membrane raft assembly	Biological Process	GO:0001765	0.0249
regulation of respiratory gaseous exchange by nervous system process	Biological Process	GO:0002087	0.0249
macrophage activation involved in immune response	Biological Process	GO:0002281	0.0249
respiratory burst involved in defense response	Biological Process	GO:0002679	0.0249
negative regulation of type 2 immune response	Biological Process	GO:0002829	0.0249
Wnt signaling pathway involved in heart development	Biological Process	GO:0003306	0.0249
cellular response to nitrogen starvation	Biological Process	GO:0006995	0.0249
UV protection	Biological Process	GO:0009650	0.0249
positive regulation of keratinocyte proliferation	Biological Process	GO:0010838	0.0249
negative regulation of muscle adaptation	Biological Process	GO:0014745	0.0249
regulation of skeletal muscle satellite cell proliferation	Biological Process	GO:0014842	0.0249
urea metabolic process	Biological Process	GO:0019627	0.0249
telencephalon regionalization	Biological Process	GO:0021978	0.0249
Cdc42 protein signal transduction	Biological Process	GO:0032488	0.0249
positive regulation of granulocyte macrophage colony-stimulating factor production	Biological Process	GO:0032725	0.0249
ceramide transport	Biological Process	GO:0035627	0.0249
cellular response to nitrogen levels	Biological Process	GO:0043562	0.0249
positive thymic T cell selection	Biological Process	GO:0045059	0.0249
positive regulation of interleukin-2 biosynthetic process	Biological Process	GO:0045086	0.0249
negative regulation of bone resorption	Biological Process	GO:0045779	0.0249
negative regulation of axon regeneration	Biological Process	GO:0048681	0.0249
regulation of interleukin-1 beta biosynthetic process	Biological Process	GO:0050722	0.0249
UV-damage excision repair	Biological Process	GO:0070914	0.0249
cellular response to manganese ion	Biological Process	GO:0071287	0.0249
ectodermal placode development	Biological Process	GO:0071696	0.0249
collecting duct development	Biological Process	GO:0072044	0.0249
regulation of histone H4 acetylation	Biological Process	GO:0090239	0.0249

negative regulation of amyloid-beta formation	Biological Process	GO:1902430	0.0249
regulation of bile acid metabolic process	Biological Process	GO:1904251	0.0249
positive regulation of cellular response to drug	Biological Process	GO:2001040	0.0249
embryonic axis specification	Biological Process	GO:0000578	0.0249
MyD88-dependent toll-like receptor signaling pathway	Biological Process	GO:0002755	0.0249
regulation of cell fate commitment	Biological Process	GO:0010453	0.0249
superoxide anion generation	Biological Process	GO:0042554	0.0249
positive regulation of DNA replication	Biological Process	GO:0045740	0.0249
negative regulation of insulin receptor signaling pathway	Biological Process	GO:0046627	0.0249
positive regulation of telomerase activity	Biological Process	GO:0051973	0.0249
negative regulation of calcium ion transmembrane transporter activity	Biological Process	GO:1901020	0.0249
monocarboxylic acid transport	Biological Process	GO:0015718	0.025
regulation of synaptic vesicle cycle	Biological Process	GO:0098693	0.0258
mucopolysaccharide metabolic process	Biological Process	GO:1903510	0.0258
acylglycerol metabolic process	Biological Process	GO:0006639	0.026
cellular hormone metabolic process	Biological Process	GO:0034754	0.026
RNA stabilization	Biological Process	GO:0043489	0.026
positive regulation of anion transport	Biological Process	GO:1903793	0.026
regulation of platelet-derived growth factor receptor signaling pathway	Biological Process	GO:0010640	0.026
regulation of cardiac muscle contraction by regulation of the release of sequestered calcium ion	Biological Process	GO:0010881	0.026
response to muscle activity	Biological Process	GO:0014850	0.026
forebrain regionalization	Biological Process	GO:0021871	0.026
regulation of T cell differentiation in thymus	Biological Process	GO:0033081	0.026
negative regulation of defense response to virus	Biological Process	GO:0050687	0.026
positive regulation of antigen receptor-mediated signaling pathway	Biological Process	GO:0050857	0.026
cytoplasmic sequestering of protein	Biological Process	GO:0051220	0.026
trabecula formation	Biological Process	GO:0060343	0.026
growth hormone receptor signaling pathway	Biological Process	GO:0060396	0.026
negative regulation of protein localization to cell periphery	Biological Process	GO:1904376	0.026
negative regulation of neural precursor cell proliferation	Biological Process	GO:2000178	0.026
regulation of hemostasis	Biological Process	GO:1900046	0.0261
organic acid transport	Biological Process	GO:0015849	0.0263
lipid catabolic process	Biological Process	GO:0016042	0.0263
regulation of alternative mRNA splicing, via spliceosome	Biological Process	GO:0000381	0.0267
positive regulation of DNA repair	Biological Process	GO:0045739	0.0267

chromosome organization involved in meiotic cell cycle	Biological Process	GO:0070192	0.0267
regulation of potassium ion transmembrane transporter activity	Biological Process	GO:1901016	0.0267
positive regulation of canonical Wnt signaling pathway	Biological Process	GO:0090263	0.0268
neutral lipid metabolic process	Biological Process	GO:0006638	0.0272
DNA replication initiation	Biological Process	GO:0006270	0.0276
positive regulation of protein-containing complex disassembly	Biological Process	GO:0043243	0.0276
axon extension involved in axon guidance	Biological Process	GO:0048846	0.0276
positive regulation of substrate adhesion-dependent cell spreading	Biological Process	GO:1900026	0.0276
neuron projection extension involved in neuron projection guidance	Biological Process	GO:1902284	0.0276
release of sequestered calcium ion into cytosol by endoplasmic reticulum	Biological Process	GO:1903514	0.0276
anaphase-promoting complex-dependent catabolic process	Biological Process	GO:0031145	0.0279
iron ion homeostasis	Biological Process	GO:0055072	0.0279
cAMP-mediated signaling	Biological Process	GO:0019933	0.0285
icosanoid metabolic process	Biological Process	GO:0006690	0.0286
calcium-ion regulated exocytosis	Biological Process	GO:0017156	0.0288
fatty acid derivative metabolic process	Biological Process	GO:1901568	0.0298
renal system process involved in regulation of systemic arterial blood pressure	Biological Process	GO:0003071	0.0298
ionotropic glutamate receptor signaling pathway	Biological Process	GO:0035235	0.0298
cellular response to growth hormone stimulus	Biological Process	GO:0071378	0.0298
negative regulation of synapse organization	Biological Process	GO:1905809	0.0298
telomere maintenance via telomere lengthening	Biological Process	GO:0010833	0.0298
RNA catabolic process	Biological Process	GO:0006401	0.0299
negative regulation of protein-containing complex assembly	Biological Process	GO:0031333	0.03
negative regulation of protein catabolic process	Biological Process	GO:0042177	0.03
DNA unwinding involved in DNA replication	Biological Process	GO:0006268	0.03
hydrogen peroxide-mediated programmed cell death	Biological Process	GO:0010421	0.03
negative regulation of mitochondrial membrane potential	Biological Process	GO:0010917	0.03
skeletal muscle satellite cell proliferation	Biological Process	GO:0014841	0.03
regulation of skeletal muscle cell proliferation	Biological Process	GO:0014857	0.03
prostaglandin secretion	Biological Process	GO:0032310	0.03
negative regulation of interferon-beta production	Biological Process	GO:0032688	0.03
regulation of odontogenesis of dentin-containing tooth	Biological Process	GO:0042487	0.03
negative regulation by host of viral transcription	Biological Process	GO:0043922	0.03
positive regulation of vacuole organization	Biological Process	GO:0044090	0.03
T-helper 2 cell differentiation	Biological Process	GO:0045064	0.03
gamma-delta T cell activation	Biological Process	GO:0046629	0.03

interleukin-1 beta biosynthetic process	Biological Process	GO:0050720	0.03
positive regulation of T cell receptor signaling pathway	Biological Process	GO:0050862	0.03
Golgi localization	Biological Process	GO:0051645	0.03
growth hormone receptor signaling pathway via JAK-STAT	Biological Process	GO:0060397	0.03
positive regulation of cardiac muscle contraction	Biological Process	GO:0060452	0.03
response to interleukin-15	Biological Process	GO:0070672	0.03
negative regulation of calcineurin-NFAT signaling cascade	Biological Process	GO:0070885	0.03
nitrogen cycle metabolic process	Biological Process	GO:0071941	0.03
positive regulation of metanephros development	Biological Process	GO:0072216	0.03
embryonic skeletal joint development	Biological Process	GO:0072498	0.03
programmed cell death in response to reactive oxygen species	Biological Process	GO:0097468	0.03
detection of other organism	Biological Process	GO:0098543	0.03
negative regulation of calcineurin-mediated signaling	Biological Process	GO:0106057	0.03
regulation of postsynaptic membrane organization	Biological Process	GO:1901626	0.03
regulation of DNA catabolic process	Biological Process	GO:1903624	0.03
negative regulation of metallopeptidase activity	Biological Process	GO:1905049	0.03
negative regulation of vascular smooth muscle cell differentiation	Biological Process	GO:1905064	0.03
regulation of unsaturated fatty acid biosynthetic process	Biological Process	GO:2001279	0.03
blastocyst formation	Biological Process	GO:0001825	0.0303
regulation of necrotic cell death	Biological Process	GO:0010939	0.0303
response to food	Biological Process	GO:0032094	0.0303
hippo signaling	Biological Process	GO:0035329	0.0303
early endosome to late endosome transport	Biological Process	GO:0045022	0.0303
regulation of protein export from nucleus	Biological Process	GO:0046825	0.0303
positive regulation of interleukin-1 secretion	Biological Process	GO:0050716	0.0303
positive regulation of neurotransmitter transport	Biological Process	GO:0051590	0.0303
response to growth hormone	Biological Process	GO:0060416	0.0303
cellular defense response	Biological Process	GO:0006968	0.0303
response to fungus	Biological Process	GO:0009620	0.0303
regulation of substrate adhesion-dependent cell spreading	Biological Process	GO:1900024	0.0303
centrosome duplication	Biological Process	GO:0051298	0.0306
action potential	Biological Process	GO:0001508	0.0311
microtubule organizing center organization	Biological Process	GO:0031023	0.0311
transition metal ion homeostasis	Biological Process	GO:0055076	0.0311
cellular response to calcium ion	Biological Process	GO:0071277	0.0313
phosphatidylinositol biosynthetic process	Biological Process	GO:0006661	0.0313

positive regulation of protein polymerization	Biological Process	GO:0032273	0.0327
amine transport	Biological Process	GO:0015837	0.0327
digestive system process	Biological Process	GO:0022600	0.0327
excitatory postsynaptic potential	Biological Process	GO:0060079	0.0327
skeletal muscle cell differentiation	Biological Process	GO:0035914	0.0329
negative regulation of interleukin-6 production	Biological Process	GO:0032715	0.0329
response to dsRNA	Biological Process	GO:0043331	0.0329
negative regulation of peptidyl-tyrosine phosphorylation	Biological Process	GO:0050732	0.0329
regulation of coagulation	Biological Process	GO:0050818	0.0332
endocrine process	Biological Process	GO:0050886	0.0332
neurotrophin signaling pathway	Biological Process	GO:0038179	0.0332
regulation of T cell receptor signaling pathway	Biological Process	GO:0050856	0.0332
C21-steroid hormone biosynthetic process	Biological Process	GO:0006700	0.0332
positive regulation of heart rate	Biological Process	GO:0010460	0.0332
myelination in peripheral nervous system	Biological Process	GO:0022011	0.0332
positive regulation of blood coagulation	Biological Process	GO:0030194	0.0332
melanocyte differentiation	Biological Process	GO:0030318	0.0332
peripheral nervous system axon ensheathment	Biological Process	GO:0032292	0.0332
negative regulation of viral transcription	Biological Process	GO:0032897	0.0332
regulation of water loss via skin	Biological Process	GO:0033561	0.0332
regulation of odontogenesis	Biological Process	GO:0042481	0.0332
amino acid import	Biological Process	GO:0043090	0.0332
embryonic camera-type eye morphogenesis	Biological Process	GO:0048596	0.0332
cardiac myofibril assembly	Biological Process	GO:0055003	0.0332
regulation of necroptotic process	Biological Process	GO:0060544	0.0332
regulation of androgen receptor signaling pathway	Biological Process	GO:0060765	0.0332
regulation of programmed necrotic cell death	Biological Process	GO:0062098	0.0332
global genome nucleotide-excision repair	Biological Process	GO:0070911	0.0332
positive regulation of granulocyte chemotaxis	Biological Process	GO:0071624	0.0332
positive regulation of hemostasis	Biological Process	GO:1900048	0.0332
membrane invagination	Biological Process	GO:0010324	0.034
axo-dendritic transport	Biological Process	GO:0008088	0.035
synaptic vesicle recycling	Biological Process	GO:0036465	0.035
positive regulation of phagocytosis	Biological Process	GO:0050766	0.035
regulation of interleukin-2 production	Biological Process	GO:0032663	0.0355
mRNA catabolic process	Biological Process	GO:0006402	0.0355

germinal center formation	Biological Process	GO:0002467	0.0355
positive regulation of macrophage chemotaxis	Biological Process	GO:0010759	0.0355
skeletal muscle cell proliferation	Biological Process	GO:0014856	0.0355
male sex determination	Biological Process	GO:0030238	0.0355
regulation of granulocyte differentiation	Biological Process	GO:0030852	0.0355
regulation of granulocyte macrophage colony-stimulating factor production	Biological Process	GO:0032645	0.0355
positive regulation of mast cell activation involved in immune response	Biological Process	GO:0033008	0.0355
glucocorticoid receptor signaling pathway	Biological Process	GO:0042921	0.0355
positive regulation of mast cell degranulation	Biological Process	GO:0043306	0.0355
negative regulation of JUN kinase activity	Biological Process	GO:0043508	0.0355
engulfment of apoptotic cell	Biological Process	GO:0043652	0.0355
regulation of interleukin-1 biosynthetic process	Biological Process	GO:0045360	0.0355
locomotor rhythm	Biological Process	GO:0045475	0.0355
negative regulation of membrane potential	Biological Process	GO:0045837	0.0355
positive regulation of membrane potential	Biological Process	GO:0045838	0.0355
negative regulation of bone remodeling	Biological Process	GO:0046851	0.0355
negative regulation of astrocyte differentiation	Biological Process	GO:0048712	0.0355
negative regulation of release of sequestered calcium ion into cytosol	Biological Process	GO:0051280	0.0355
neuroblast division	Biological Process	GO:0055057	0.0355
negative regulation of glial cell proliferation	Biological Process	GO:0060253	0.0355
venous blood vessel development	Biological Process	GO:0060841	0.0355
hepatocyte differentiation	Biological Process	GO:0070365	0.0355
metanephric renal vesicle morphogenesis	Biological Process	GO:0072283	0.0355
epithelial cell-cell adhesion	Biological Process	GO:0090136	0.0355
positive regulation of execution phase of apoptosis	Biological Process	GO:1900119	0.0355
positive regulation of viral release from host cell	Biological Process	GO:1902188	0.0355
mitotic DNA replication	Biological Process	GO:1902969	0.0355
positive regulation of exosomal secretion	Biological Process	GO:1903543	0.0355
regulation of plasma membrane organization	Biological Process	GO:1903729	0.0355
regulation of Wnt signaling pathway, planar cell polarity pathway	Biological Process	GO:2000095	0.0355
protein tetramerization	Biological Process	GO:0051262	0.0357
vesicle organization	Biological Process	GO:0016050	0.036
determination of left/right symmetry	Biological Process	GO:0007368	0.036
positive regulation of TOR signaling	Biological Process	GO:0032008	0.0362
fear response	Biological Process	GO:0042596	0.0362
cellular response to osmotic stress	Biological Process	GO:0071470	0.0362

semaphorin-plexin signaling pathway	Biological Process	GO:0071526	0.0362
regulation of protein localization to cell surface	Biological Process	GO:2000008	0.0362
response to dietary excess	Biological Process	GO:0002021	0.0372
regulation of T-helper 1 type immune response	Biological Process	GO:0002825	0.0372
response to salt stress	Biological Process	GO:0009651	0.0372
positive regulation of skeletal muscle tissue development	Biological Process	GO:0048643	0.0372
positive regulation of coagulation	Biological Process	GO:0050820	0.0372
cellular response to copper ion	Biological Process	GO:0071280	0.0372
metanephric epithelium development	Biological Process	GO:0072207	0.0372
import across plasma membrane	Biological Process	GO:0098739	0.0378
regulation of protein import into nucleus	Biological Process	GO:0042306	0.038
pigment metabolic process	Biological Process	GO:0042440	0.0397
regulation of multi-organism process	Biological Process	GO:0043900	0.0397
cell cycle DNA replication	Biological Process	GO:0044786	0.0397
regulation of gluconeogenesis	Biological Process	GO:0006111	0.0398
negative regulation of potassium ion transport	Biological Process	GO:0043267	0.0398
ruffle assembly	Biological Process	GO:0097178	0.0398
vesicle-mediated transport between endosomal compartments	Biological Process	GO:0098927	0.0398
regulation of phosphatase activity	Biological Process	GO:0010921	0.0401
alcohol biosynthetic process	Biological Process	GO:0046165	0.0401
nuclear export	Biological Process	GO:0051168	0.0409
synaptic vesicle endocytosis	Biological Process	GO:0048488	0.041
histone H3-K4 methylation	Biological Process	GO:0051568	0.041
cell communication involved in cardiac conduction	Biological Process	GO:0086065	0.041
presynaptic endocytosis	Biological Process	GO:0140238	0.041
alcohol metabolic process	Biological Process	GO:0006066	0.0412
defense response to Gram-negative bacterium	Biological Process	GO:0050829	0.0415
gastrulation with mouth forming second	Biological Process	GO:0001702	0.0415
androgen metabolic process	Biological Process	GO:0008209	0.0415
cell proliferation in forebrain	Biological Process	GO:0021846	0.0415
regulation of osteoblast proliferation	Biological Process	GO:0033688	0.0415
response to interferon-beta	Biological Process	GO:0035456	0.0415
regulation of hair cycle	Biological Process	GO:0042634	0.0415
RNA polymerase II preinitiation complex assembly	Biological Process	GO:0051123	0.0415
positive regulation of chromosome segregation	Biological Process	GO:0051984	0.0415
regulation of vascular smooth muscle cell differentiation	Biological Process	GO:1905063	0.0415

ventricular trabecula myocardium morphogenesis	Biological Process	GO:0003222	0.0415
positive regulation of T cell chemotaxis	Biological Process	GO:0010820	0.0415
macrophage cytokine production	Biological Process	GO:0010934	0.0415
negative regulation of phosphatidylinositol 3-kinase signaling	Biological Process	GO:0014067	0.0415
protein autoprocessing	Biological Process	GO:0016540	0.0415
hyaluronan catabolic process	Biological Process	GO:0030214	0.0415
membrane disassembly	Biological Process	GO:0030397	0.0415
negative regulation of B cell proliferation	Biological Process	GO:0030889	0.0415
positive regulation of icosanoid secretion	Biological Process	GO:0032305	0.0415
response to follicle-stimulating hormone	Biological Process	GO:0032354	0.0415
granulocyte macrophage colony-stimulating factor production	Biological Process	GO:0032604	0.0415
negative regulation of interleukin-12 production	Biological Process	GO:0032695	0.0415
peptidyl-threonine dephosphorylation	Biological Process	GO:0035970	0.0415
histone H3-K9 trimethylation	Biological Process	GO:0036124	0.0415
interleukin-1 biosynthetic process	Biological Process	GO:0042222	0.0415
regulation of respiratory system process	Biological Process	GO:0044065	0.0415
negative regulation of T-helper cell differentiation	Biological Process	GO:0045623	0.0415
negative regulation of smooth muscle contraction	Biological Process	GO:0045986	0.0415
positive regulation of striated muscle contraction	Biological Process	GO:0045989	0.0415
negative regulation of glucose import	Biological Process	GO:0046325	0.0415
nuclear envelope disassembly	Biological Process	GO:0051081	0.0415
atrial septum morphogenesis	Biological Process	GO:0060413	0.0415
ureter development	Biological Process	GO:0072189	0.0415
synaptic vesicle clustering	Biological Process	GO:0097091	0.0415
negative regulation of intrinsic apoptotic signaling pathway in response to DNA damage by p53 class mediator	Biological Process	GO:1902166	0.0415
regulation of exosomal secretion	Biological Process	GO:1903541	0.0415
regulation of clathrin-dependent endocytosis	Biological Process	GO:2000369	0.0415
regulation of TOR signaling	Biological Process	GO:0032006	0.0417
regulation of potassium ion transport	Biological Process	GO:0043266	0.0417
positive regulation of striated muscle cell differentiation	Biological Process	GO:0051155	0.0419
regulation of hematopoietic stem cell differentiation	Biological Process	GO:1902036	0.0419
organic anion transport	Biological Process	GO:0015711	0.0427
base-excision repair	Biological Process	GO:0006284	0.043
pituitary gland development	Biological Process	GO:0021983	0.043
ketone biosynthetic process	Biological Process	GO:0042181	0.043

regulation of vacuole organization	Biological Process	GO:0044088	0.043
lymphocyte mediated immunity	Biological Process	GO:0002449	0.0432
regulation of calcium ion transmembrane transporter activity	Biological Process	GO:1901019	0.0437
regulation of cardiac muscle cell apoptotic process	Biological Process	GO:0010665	0.0437
morphogenesis of a polarized epithelium	Biological Process	GO:0001738	0.0437
regulation of defense response to virus	Biological Process	GO:0050688	0.0446
regulation of cardiac conduction	Biological Process	GO:1903779	0.0446
establishment of planar polarity	Biological Process	GO:0001736	0.0453
establishment of tissue polarity	Biological Process	GO:0007164	0.0453
glycosaminoglycan metabolic process	Biological Process	GO:0030203	0.0453
meiotic chromosome segregation	Biological Process	GO:0045132	0.0462
respiratory system process	Biological Process	GO:0003016	0.0462
nitric oxide mediated signal transduction	Biological Process	GO:0007263	0.0462
regulation of cardiac muscle contraction by calcium ion signaling	Biological Process	GO:0010882	0.0462
Schwann cell development	Biological Process	GO:0014044	0.0462
cranial nerve morphogenesis	Biological Process	GO:0021602	0.0462
regulation of syncytium formation by plasma membrane fusion	Biological Process	GO:0060142	0.0462
regulation of ruffle assembly	Biological Process	GO:1900027	0.0462
positive regulation of cardiac muscle cell differentiation	Biological Process	GO:2000727	0.0462
cortical actin cytoskeleton organization	Biological Process	GO:0030866	0.0468
regulation of cyclase activity	Biological Process	GO:0031279	0.0468
regulation of cardiac muscle cell contraction	Biological Process	GO:0086004	0.0468
positive regulation of establishment of protein localization to mitochondrion	Biological Process	GO:1903749	0.0469
regulation of protein import	Biological Process	GO:1904589	0.0469
negative regulation of cytokine secretion	Biological Process	GO:0050710	0.0474
cardiac muscle cell action potential	Biological Process	GO:0086001	0.0474
cardiac muscle cell contraction	Biological Process	GO:0086003	0.0474
negative regulation of sprouting angiogenesis	Biological Process	GO:1903671	0.0474
response to topologically incorrect protein	Biological Process	GO:0035966	0.0482
immune response to tumor cell	Biological Process	GO:0002418	0.0482
lactate metabolic process	Biological Process	GO:0006089	0.0482
DNA damage response, signal transduction by p53 class mediator resulting in transcription of p21 class mediator	Biological Process	GO:0006978	0.0482
regulation of extracellular matrix disassembly	Biological Process	GO:0010715	0.0482
prostaglandin transport	Biological Process	GO:0015732	0.0482
wound healing, spreading of epidermal cells	Biological Process	GO:0035313	0.0482

megakaryocyte development	Biological Process	GO:0035855	0.0482
nodal signaling pathway	Biological Process	GO:0038092	0.0482
copper ion homeostasis	Biological Process	GO:0055070	0.0482
positive regulation of mitotic sister chromatid segregation	Biological Process	GO:0062033	0.0482
cellular response to exogenous dsRNA	Biological Process	GO:0071360	0.0482
regulation of mitophagy	Biological Process	GO:1901524	0.0482
negative regulation of alcohol biosynthetic process	Biological Process	GO:1902931	0.0482
regulation of early endosome to late endosome transport	Biological Process	GO:2000641	0.0482
Wnt signaling pathway, planar cell polarity pathway	Biological Process	GO:0060071	0.0483
chemical synaptic transmission, postsynaptic	Biological Process	GO:0099565	0.0483
transcription regulator complex	Cellular Component	GO:0005667	3.31E-22
membrane raft	Cellular Component	GO:0045121	2.77E-14
membrane microdomain	Cellular Component	GO:0098857	2.77E-14
membrane region	Cellular Component	GO:0098589	9.37E-14
focal adhesion	Cellular Component	GO:0005925	2.78E-13
cell-substrate junction	Cellular Component	GO:0030055	4.78E-13
RNA polymerase II transcription regulator complex	Cellular Component	GO:0090575	1.72E-12
nuclear chromatin	Cellular Component	GO:0000790	2.40E-12
early endosome	Cellular Component	GO:0005769	6.02E-10
protein kinase complex	Cellular Component	GO:1902911	5.58E-09
transferase complex, transferring phosphorus-containing groups	Cellular Component	GO:0061695	1.28E-08
cyclin-dependent protein kinase holoenzyme complex	Cellular Component	GO:0000307	1.24E-07
serine/threonine protein kinase complex	Cellular Component	GO:1902554	1.73E-07
external side of plasma membrane	Cellular Component	GO:0009897	1.73E-07
cell leading edge	Cellular Component	GO:0031252	3.35E-07
heterochromatin	Cellular Component	GO:0000792	6.64E-07
cell-cell junction	Cellular Component	GO:0005911	2.13E-06
extrinsic component of membrane	Cellular Component	GO:0019898	2.78E-06
vesicle lumen	Cellular Component	GO:0031983	2.93E-06
basolateral plasma membrane	Cellular Component	GO:0016323	5.81E-06
caveola	Cellular Component	GO:0005901	6.79E-06
cytoplasmic vesicle lumen	Cellular Component	GO:0060205	6.79E-06
lamellipodium	Cellular Component	GO:0030027	7.41E-06
ruffle	Cellular Component	GO:0001726	1.35E-05
plasma membrane raft	Cellular Component	GO:0044853	1.52E-05
phagophore assembly site membrane	Cellular Component	GO:0034045	1.95E-05

neuronal cell body	Cellular Component	GO:0043025	2.13E-05
platelet alpha granule lumen	Cellular Component	GO:0031093	2.21E-05
secretory granule lumen	Cellular Component	GO:0034774	3.46E-05
phagophore assembly site	Cellular Component	GO:0000407	3.58E-05
collagen-containing extracellular matrix	Cellular Component	GO:0062023	3.67E-05
glutamatergic synapse	Cellular Component	GO:0098978	6.50E-05
PML body	Cellular Component	GO:0016605	7.50E-05
endocytic vesicle	Cellular Component	GO:0030139	7.50E-05
phosphatidylinositol 3-kinase complex	Cellular Component	GO:0005942	7.50E-05
platelet alpha granule	Cellular Component	GO:0031091	0.000119
apical part of cell	Cellular Component	GO:0045177	0.000157
cell-cell contact zone	Cellular Component	GO:0044291	0.000188
mitochondrial outer membrane	Cellular Component	GO:0005741	0.000197
extrinsic component of plasma membrane	Cellular Component	GO:0019897	0.000221
chromosomal region	Cellular Component	GO:0098687	0.000317
outer membrane	Cellular Component	GO:0019867	0.000392
basal plasma membrane	Cellular Component	GO:0009925	0.000392
midbody	Cellular Component	GO:0030496	0.000392
ribonucleoprotein granule	Cellular Component	GO:0035770	5.00E-04
actin-based cell projection	Cellular Component	GO:0098858	0.000518
COP9 signalosome	Cellular Component	GO:0008180	0.000563
cell projection membrane	Cellular Component	GO:0031253	0.000565
ruffle membrane	Cellular Component	GO:0032587	0.000587
cytoplasmic ribonucleoprotein granule	Cellular Component	GO:0036464	0.00062
Schaffer collateral - CA1 synapse	Cellular Component	GO:0098685	0.00062
nuclear matrix	Cellular Component	GO:0016363	0.000663
euchromatin	Cellular Component	GO:0000791	0.000755
organelle outer membrane	Cellular Component	GO:0031968	0.000818
intercalated disc	Cellular Component	GO:0014704	0.000948
basal part of cell	Cellular Component	GO:0045178	0.00109
recycling endosome	Cellular Component	GO:0055037	0.00123
nuclear periphery	Cellular Component	GO:0034399	0.00124
endosome membrane	Cellular Component	GO:0010008	0.00152
early endosome membrane	Cellular Component	GO:0031901	0.00155
nuclear heterochromatin	Cellular Component	GO:0005720	0.00178
apical plasma membrane	Cellular Component	GO:0016324	0.00178

glial cell projection	Cellular Component	GO:0097386	0.00191
leading edge membrane	Cellular Component	GO:0031256	0.00219
lateral plasma membrane	Cellular Component	GO:0016328	0.00227
growth cone	Cellular Component	GO:0030426	0.00227
spindle	Cellular Component	GO:0005819	0.00277
dendritic spine	Cellular Component	GO:0043197	0.00288
fascia adherens	Cellular Component	GO:0005916	0.00288
site of polarized growth	Cellular Component	GO:0030427	0.00301
neuron spine	Cellular Component	GO:0044309	0.00318
cytoplasmic side of membrane	Cellular Component	GO:0098562	0.00334
phagocytic vesicle	Cellular Component	GO:0045335	0.00364
filopodium	Cellular Component	GO:0030175	0.00419
nuclear euchromatin	Cellular Component	GO:0005719	0.00581
beta-catenin destruction complex	Cellular Component	GO:0030877	0.00581
nuclear envelope	Cellular Component	GO:0005635	0.00592
neuron to neuron synapse	Cellular Component	GO:0098984	0.00609
endoribonuclease complex	Cellular Component	GO:1902555	0.00664
chromosome, telomeric region	Cellular Component	GO:0000781	0.00681
secretory granule membrane	Cellular Component	GO:0030667	0.00688
clathrin-coated pit	Cellular Component	GO:0005905	0.00761
mast cell granule	Cellular Component	GO:0042629	0.00783
perinuclear endoplasmic reticulum	Cellular Component	GO:0097038	0.00783
endocytic vesicle membrane	Cellular Component	GO:0030666	0.0112
PcG protein complex	Cellular Component	GO:0031519	0.0117
NuRD complex	Cellular Component	GO:0016581	0.0126
CHD-type complex	Cellular Component	GO:0090545	0.0126
nuclear membrane	Cellular Component	GO:0031965	0.0129
cytoplasmic side of plasma membrane	Cellular Component	GO:0009898	0.0133
nuclear chromosome, telomeric region	Cellular Component	GO:0000784	0.0148
endonuclease complex	Cellular Component	GO:1905348	0.0149
ESC/E(Z) complex	Cellular Component	GO:0035098	0.0152
ficolin-1-rich granule	Cellular Component	GO:0101002	0.0152
ficolin-1-rich granule lumen	Cellular Component	GO:1904813	0.0152
chromosome, centromeric region	Cellular Component	GO:0000775	0.0161
asymmetric synapse	Cellular Component	GO:0032279	0.0197
postsynaptic specialization	Cellular Component	GO:0099572	0.0199

cell division site	Cellular Component	GO:0032153	0.0223
synaptic membrane	Cellular Component	GO:0097060	0.0223
late endosome	Cellular Component	GO:0005770	0.0223
filopodium membrane	Cellular Component	GO:0031527	0.0223
condensed nuclear chromosome	Cellular Component	GO:0000794	0.0229
catenin complex	Cellular Component	GO:0016342	0.0229
cleavage furrow	Cellular Component	GO:0032154	0.0239
DNA repair complex	Cellular Component	GO:1990391	0.0248
clathrin-coated vesicle	Cellular Component	GO:0030136	0.0275
laminin complex	Cellular Component	GO:0043256	0.0275
sarcolemma	Cellular Component	GO:0042383	0.0281
postsynaptic density	Cellular Component	GO:0014069	0.0304
distal axon	Cellular Component	GO:0150034	0.0304
cell cortex	Cellular Component	GO:0005938	0.0341
presynapse	Cellular Component	GO:0098793	0.0341
endoplasmic reticulum lumen	Cellular Component	GO:0005788	0.0349
postsynaptic specialization, intracellular component	Cellular Component	GO:0099091	0.0354
extrinsic component of cytoplasmic side of plasma membrane	Cellular Component	GO:0031234	0.0358
neuromuscular junction	Cellular Component	GO:0031594	0.0373
SWI/SNF superfamily-type complex	Cellular Component	GO:0070603	0.0373
presynaptic membrane	Cellular Component	GO:0042734	0.0385
transcription repressor complex	Cellular Component	GO:0017053	0.0396
spindle midzone	Cellular Component	GO:0051233	0.0396
nucleotide-excision repair complex	Cellular Component	GO:0000109	0.0418
Wnt signalosome	Cellular Component	GO:1990909	0.0418
tight junction	Cellular Component	GO:0070160	0.0418
actin filament	Cellular Component	GO:0005884	0.0418
spindle pole	Cellular Component	GO:0000922	0.0422
ATPase complex	Cellular Component	GO:1904949	0.0468
autophagosome membrane	Cellular Component	GO:0000421	0.0473
immunological synapse	Cellular Component	GO:0001772	0.0473
postsynaptic membrane	Cellular Component	GO:0045211	0.0492
uropod	Cellular Component	GO:0001931	0.0493
cell trailing edge	Cellular Component	GO:0031254	0.0493
clathrin-coated vesicle membrane	Cellular Component	GO:0030665	0.0498
DNA-binding transcription activator activity, RNA polymerase II-specific	Molecular Function	GO:0001228	3.22E-29

DNA-binding transcription activator activity	Molecular Function	GO:0001216	3.22E-29
cytokine receptor binding	Molecular Function	GO:0005126	1.75E-22
DNA-binding transcription factor binding	Molecular Function	GO:0140297	1.56E-16
cytokine activity	Molecular Function	GO:0005125	7.50E-14
ubiquitin-like protein ligase binding	Molecular Function	GO:0044389	7.50E-14
RNA polymerase II-specific DNA-binding transcription factor binding	Molecular Function	GO:0061629	9.71E-14
SMAD binding	Molecular Function	GO:0046332	9.71E-14
ubiquitin protein ligase binding	Molecular Function	GO:0031625	4.52E-13
protein tyrosine kinase activity	Molecular Function	GO:0004713	2.37E-12
kinase regulator activity	Molecular Function	GO:0019207	2.02E-11
protein serine/threonine kinase activity	Molecular Function	GO:0004674	2.02E-11
protease binding	Molecular Function	GO:0002020	2.46E-11
signaling receptor activator activity	Molecular Function	GO:0030546	1.10E-10
growth factor activity	Molecular Function	GO:0008083	3.29E-10
transmembrane receptor protein kinase activity	Molecular Function	GO:0019199	3.29E-10
receptor ligand activity	Molecular Function	GO:0048018	6.18E-10
protein kinase regulator activity	Molecular Function	GO:0019887	3.87E-09
regulatory RNA binding	Molecular Function	GO:0061980	4.02E-09
E-box binding	Molecular Function	GO:0070888	4.02E-09
protein phosphatase binding	Molecular Function	GO:0019903	4.89E-09
DNA-binding transcription repressor activity	Molecular Function	GO:0001217	4.89E-09
DNA-binding transcription repressor activity, RNA polymerase II-specific	Molecular Function	GO:0001227	4.89E-09
integrin binding	Molecular Function	GO:0005178	6.68E-09
growth factor receptor binding	Molecular Function	GO:0070851	8.98E-09
histone deacetylase binding	Molecular Function	GO:0042826	3.08E-08
I-SMAD binding	Molecular Function	GO:0070411	4.13E-08
growth factor binding	Molecular Function	GO:0019838	6.90E-08
transmembrane receptor protein tyrosine kinase activity	Molecular Function	GO:0004714	9.47E-08
phosphatase binding	Molecular Function	GO:0019902	9.47E-08
steroid hormone receptor activity	Molecular Function	GO:0003707	1.60E-07
cyclin-dependent protein serine/threonine kinase regulator activity	Molecular Function	GO:0016538	1.99E-07
cell adhesion molecule binding	Molecular Function	GO:0050839	2.44E-07
activating transcription factor binding	Molecular Function	GO:0033613	2.64E-07
transforming growth factor beta receptor binding	Molecular Function	GO:0005160	3.23E-07
protein C-terminus binding	Molecular Function	GO:0008022	4.22E-07
miRNA binding	Molecular Function	GO:0035198	5.64E-07

beta-catenin binding	Molecular Function	GO:0008013	8.44E-07
disordered domain specific binding	Molecular Function	GO:0097718	1.12E-06
promoter-specific chromatin binding	Molecular Function	GO:1990841	1.12E-06
phosphatidylinositol 3-kinase binding	Molecular Function	GO:0043548	4.41E-06
nuclear receptor activity	Molecular Function	GO:0004879	6.89E-06
ligand-activated transcription factor activity	Molecular Function	GO:0098531	6.89E-06
chemokine receptor binding	Molecular Function	GO:0042379	8.60E-06
transcription coactivator activity	Molecular Function	GO:0003713	1.05E-05
nuclear receptor binding	Molecular Function	GO:0016922	1.21E-05
hormone receptor binding	Molecular Function	GO:0051427	1.49E-05
siRNA binding	Molecular Function	GO:0035197	3.47E-05
insulin receptor substrate binding	Molecular Function	GO:0043560	3.47E-05
transcription coactivator binding	Molecular Function	GO:0001223	4.23E-05
chemokine activity	Molecular Function	GO:0008009	7.08E-05
nuclear hormone receptor binding	Molecular Function	GO:0035257	8.82E-05
cytokine binding	Molecular Function	GO:0019955	9.88E-05
repressing transcription factor binding	Molecular Function	GO:0070491	0.000103
mitogen-activated protein kinase binding	Molecular Function	GO:0051019	0.00011
transcription cofactor binding	Molecular Function	GO:0001221	0.000126
RNA polymerase II activating transcription factor binding	Molecular Function	GO:0001102	0.000142
tumor necrosis factor receptor superfamily binding	Molecular Function	GO:0032813	0.000231
amyloid-beta binding	Molecular Function	GO:0001540	0.000272
insulin receptor binding	Molecular Function	GO:0005158	0.000299
cyclin binding	Molecular Function	GO:0030332	0.000319
BH domain binding	Molecular Function	GO:0051400	0.000352
death domain binding	Molecular Function	GO:0070513	0.000352
steroid hormone receptor binding	Molecular Function	GO:0035258	0.000352
R-SMAD binding	Molecular Function	GO:0070412	0.000381
tumor necrosis factor receptor binding	Molecular Function	GO:0005164	0.000381
protein tyrosine kinase binding	Molecular Function	GO:1990782	0.000381
protein phosphatase 2A binding	Molecular Function	GO:0051721	0.000476
RNA polymerase II transcription factor binding	Molecular Function	GO:0001085	0.000476
apolipoprotein binding	Molecular Function	GO:0034185	0.000524
CXCR chemokine receptor binding	Molecular Function	GO:0045236	0.000552
BMP receptor binding	Molecular Function	GO:0070700	0.000552
G protein-coupled receptor binding	Molecular Function	GO:0001664	0.000564

protein kinase inhibitor activity	Molecular Function	GO:0004860	0.000606
kinase activator activity	Molecular Function	GO:0019209	0.000622
protein serine/threonine/tyrosine kinase activity	Molecular Function	GO:0004712	0.000645
double-stranded RNA binding	Molecular Function	GO:0003725	0.000659
methyl-CpG binding	Molecular Function	GO:0008327	0.000767
cyclin-dependent protein serine/threonine kinase inhibitor activity	Molecular Function	GO:0004861	0.000833
fibronectin binding	Molecular Function	GO:0001968	0.000961
ephrin receptor binding	Molecular Function	GO:0046875	0.000961
kinase inhibitor activity	Molecular Function	GO:0019210	0.00098
non-membrane spanning protein tyrosine kinase activity	Molecular Function	GO:0004715	0.00103
insulin-like growth factor binding	Molecular Function	GO:0005520	0.00119
chromatin DNA binding	Molecular Function	GO:0031490	0.00129
phosphoprotein binding	Molecular Function	GO:0051219	0.00161
transcription corepressor activity	Molecular Function	GO:0003714	0.00163
protein N-terminus binding	Molecular Function	GO:0047485	0.0017
activin binding	Molecular Function	GO:0048185	0.00174
virus receptor activity	Molecular Function	GO:0001618	0.00218
GDP binding	Molecular Function	GO:0019003	0.00218
exogenous protein binding	Molecular Function	GO:0140272	0.00218
glycosaminoglycan binding	Molecular Function	GO:0005539	0.00225
Hsp90 protein binding	Molecular Function	GO:0051879	0.00225
transforming growth factor beta-activated receptor activity	Molecular Function	GO:0005024	0.00229
platelet-derived growth factor receptor binding	Molecular Function	GO:0005161	0.00229
NAD-dependent histone deacetylase activity	Molecular Function	GO:0017136	0.00229
cysteine-type endopeptidase activity involved in apoptotic process	Molecular Function	GO:0097153	0.00229
chemokine binding	Molecular Function	GO:0019956	0.00248
protein serine/threonine phosphatase activity	Molecular Function	GO:0004722	0.00255
cadherin binding	Molecular Function	GO:0045296	0.00278
lipoprotein particle binding	Molecular Function	GO:0071813	0.00292
protein-lipid complex binding	Molecular Function	GO:0071814	0.00292
insulin-like growth factor receptor binding	Molecular Function	GO:0005159	0.00301
NAD-dependent protein deacetylase activity	Molecular Function	GO:0034979	0.00301
coreceptor activity	Molecular Function	GO:0015026	0.00332
nuclear receptor transcription coactivator activity	Molecular Function	GO:0030374	0.00345
receptor serine/threonine kinase binding	Molecular Function	GO:0033612	0.00365
protein self-association	Molecular Function	GO:0043621	0.00372

transmembrane receptor protein serine/threonine kinase activity	Molecular Function	GO:0004675	0.00385
phosphatidylinositol 3-kinase regulator activity	Molecular Function	GO:0035014	0.00385
retinoid X receptor binding	Molecular Function	GO:0046965	0.00385
1-phosphatidylinositol-3-kinase activity	Molecular Function	GO:0016303	0.00385
alpha-catenin binding	Molecular Function	GO:0045294	0.00385
GTPase activity	Molecular Function	GO:0003924	0.00391
MAP kinase kinase kinase activity	Molecular Function	GO:0004709	0.00423
Notch binding	Molecular Function	GO:0005112	0.00423
phosphoprotein phosphatase activity	Molecular Function	GO:0004721	0.00448
iron ion binding	Molecular Function	GO:0005506	0.00448
ion channel binding	Molecular Function	GO:0044325	0.00475
scaffold protein binding	Molecular Function	GO:0097110	0.00497
heparin binding	Molecular Function	GO:0008201	0.00504
type I transforming growth factor beta receptor binding	Molecular Function	GO:0034713	0.00533
phosphatidylinositol 3-kinase activity	Molecular Function	GO:0035004	0.00533
platelet-derived growth factor binding	Molecular Function	GO:0048407	0.00533
steroid hydroxylase activity	Molecular Function	GO:0008395	0.00584
chemoattractant activity	Molecular Function	GO:0042056	0.00584
histone deacetylase activity	Molecular Function	GO:0004407	0.00587
transmembrane receptor protein serine/threonine kinase binding	Molecular Function	GO:0070696	0.00587
oxidoreductase activity, acting on paired donors, with incorporation or reduction of molecular oxygen	Molecular Function	GO:0016705	0.00664
manganese ion binding	Molecular Function	GO:0030145	0.00664
protein deacetylase activity	Molecular Function	GO:0033558	0.00685
histone acetyltransferase binding	Molecular Function	GO:0035035	0.00685
NF-kappaB binding	Molecular Function	GO:0051059	0.00685
enzyme inhibitor activity	Molecular Function	GO:0004857	0.00692
chaperone binding	Molecular Function	GO:0051087	0.00692
low-density lipoprotein particle receptor activity	Molecular Function	GO:0005041	0.00694
insulin-like growth factor I binding	Molecular Function	GO:0031994	0.00694
co-SMAD binding	Molecular Function	GO:0070410	0.00694
RNA polymerase II general transcription initiation factor binding	Molecular Function	GO:0001091	0.00694
protein phosphorylated amino acid binding	Molecular Function	GO:0045309	0.00694
phosphotyrosine residue binding	Molecular Function	GO:0001784	0.00718
cyclin-dependent protein kinase activity	Molecular Function	GO:0097472	0.00774
ribonucleoprotein complex binding	Molecular Function	GO:0043021	0.0079

vascular endothelial growth factor receptor binding	Molecular Function	GO:0005172	0.00931
estrogen receptor binding	Molecular Function	GO:0030331	0.0094
protein kinase activator activity	Molecular Function	GO:0030295	0.0103
collagen binding	Molecular Function	GO:0005518	0.0103
protein serine/threonine kinase inhibitor activity	Molecular Function	GO:0030291	0.0106
MAP kinase activity	Molecular Function	GO:0004707	0.0121
1-phosphatidylinositol-3-kinase regulator activity	Molecular Function	GO:0046935	0.0121
phosphatase activity	Molecular Function	GO:0016791	0.0124
core promoter sequence-specific DNA binding	Molecular Function	GO:0001046	0.0135
RNA polymerase II core promoter sequence-specific DNA binding	Molecular Function	GO:0000979	0.0141
lipoprotein particle receptor activity	Molecular Function	GO:0030228	0.0156
basal transcription machinery binding	Molecular Function	GO:0001098	0.016
basal RNA polymerase II transcription machinery binding	Molecular Function	GO:0001099	0.016
RNA polymerase II repressing transcription factor binding	Molecular Function	GO:0001103	0.016
phosphoric ester hydrolase activity	Molecular Function	GO:0042578	0.0174
mRNA 5'-UTR binding	Molecular Function	GO:0048027	0.0175
cysteine-type endopeptidase activity	Molecular Function	GO:0004197	0.0175
MAP kinase kinase activity	Molecular Function	GO:0004708	0.0189
metalloendopeptidase inhibitor activity	Molecular Function	GO:0008191	0.0189
arachidonic acid epoxygenase activity	Molecular Function	GO:0008392	0.0189
phosphatidylinositol kinase activity	Molecular Function	GO:0052742	0.0189
translation repressor activity	Molecular Function	GO:0030371	0.0201
retinoic acid receptor binding	Molecular Function	GO:0042974	0.0201
Wnt-protein binding	Molecular Function	GO:0017147	0.0201
heat shock protein binding	Molecular Function	GO:0031072	0.0206
sulfur compound binding	Molecular Function	GO:1901681	0.0211
channel inhibitor activity	Molecular Function	GO:0016248	0.0225
mRNA 3'-UTR binding	Molecular Function	GO:0003730	0.0225
death receptor binding	Molecular Function	GO:0005123	0.0225
arachidonic acid monooxygenase activity	Molecular Function	GO:0008391	0.0225
ubiquitin-protein transferase regulator activity	Molecular Function	GO:0055106	0.0225
bHLH transcription factor binding	Molecular Function	GO:0043425	0.0266
p53 binding	Molecular Function	GO:0002039	0.0288
cyclin-dependent protein serine/threonine kinase activity	Molecular Function	GO:0004693	0.0308
protein-hormone receptor activity	Molecular Function	GO:0016500	0.0335
general transcription initiation factor binding	Molecular Function	GO:0140296	0.0356

single-stranded DNA binding	Molecular Function	GO:0003697	0.0357
GTP binding	Molecular Function	GO:0005525	0.0368
SH3 domain binding	Molecular Function	GO:0017124	0.0379
guanyl nucleotide binding	Molecular Function	GO:0019001	0.0379
guanyl ribonucleotide binding	Molecular Function	GO:0032561	0.0379
CCR chemokine receptor binding	Molecular Function	GO:0048020	0.0386
DNA binding, bending	Molecular Function	GO:0008301	0.0386
L-ascorbic acid binding	Molecular Function	GO:0031418	0.0386
protein tyrosine phosphatase activity	Molecular Function	GO:0004725	0.04
purine ribonucleoside binding	Molecular Function	GO:0032550	0.0402
TFIID-class transcription factor complex binding	Molecular Function	GO:0001094	0.0404
ferric iron binding	Molecular Function	GO:0008199	0.0404
calcium channel inhibitor activity	Molecular Function	GO:0019855	0.0404
thioesterase binding	Molecular Function	GO:0031996	0.0404
molecular carrier activity	Molecular Function	GO:0140104	0.0404
deacetylase activity	Molecular Function	GO:0019213	0.0408
androgen receptor binding	Molecular Function	GO:0050681	0.0408
oxidoreductase activity, acting on paired donors, with incorporation or reduction of molecular oxygen, reduced flavin or flavoprotein as one donor, and incorporation of one atom of oxygen	Molecular Function	GO:0016712	0.0421
purine nucleoside binding	Molecular Function	GO:0001883	0.0421
ribonucleoside binding	Molecular Function	GO:0032549	0.0421
hormone binding	Molecular Function	GO:0042562	0.0429
calcium-release channel activity	Molecular Function	GO:0015278	0.0429
cysteine-type peptidase activity	Molecular Function	GO:0008234	0.0435
protein tyrosine/serine/threonine phosphatase activity	Molecular Function	GO:0008138	0.0437
tau protein binding	Molecular Function	GO:0048156	0.0437
heme binding	Molecular Function	GO:0020037	0.0452
copper ion binding	Molecular Function	GO:0005507	0.0456
intracellular ligand-gated ion channel activity	Molecular Function	GO:0005217	0.0459
myosin heavy chain binding	Molecular Function	GO:0032036	0.0481
sialic acid binding	Molecular Function	GO:0033691	0.0481
peroxisome proliferator activated receptor binding	Molecular Function	GO:0042975	0.0481
gamma-catenin binding	Molecular Function	GO:0045295	0.0481
transforming growth factor beta binding	Molecular Function	GO:0050431	0.0481
nucleoside binding	Molecular Function	GO:0001882	0.0488

Phosphatidylinositol Phosphate Metabolism	SMPDB Pathway	SMP0000463	0.0291
mRNA target	TTD Target	mRNA target	3.83E-16
Kinase	TTD Target	Kinase	2.53E-05
Nuclear hormone receptor	TTD Target	Nuclear hormone receptor	0.0413
Basic leucine zipper bZIP	TTD Target	Basic leucine zipper bZIP	0.045
